# Underestimated diversity in high elevations of a global biodiversity hotspot: two new endemic species of *Aethionema* (Brassicaceae) from the alpine zone of Iran

**DOI:** 10.3389/fpls.2023.1182073

**Published:** 2023-05-26

**Authors:** Hamid Moazzeni, Mohammad Mahmoodi, Mohammad Jafari, Gerald M. Schneeweiss, Jalil Noroozi

**Affiliations:** ^1^Herbarium, Department of Botany, Research Center for Plant Science, Ferdowsi University of Mashhad, Mashhad, Iran; ^2^Herbarium, Research Institute of Forests and Rangelands, Tehran, Iran; ^3^Department of Botany and Biodiversity Research, University of Vienna, Vienna, Austria

**Keywords:** Brassicaceae, conservation, endemism, phylogeny, species nova, taxonomy

## Abstract

Although the mountains in South-West Asia are a global biodiversity hotspot, our understanding of their biodiversity, especially in the commonly remote alpine and subnival zones, is still limited. This is well exemplified here by *Aethionema umbellatum* (Brassicaceae), a species considered to have a wide yet disjoint distribution in the Zagros and Yazd-Kerman mountains of western and central Iran. Morphological and molecular phylogenetic data (based on plastid *trn*L-*trn*F and nuclear ITS sequences) show that *A. umbellatum* is restricted to a single mountain range in southwestern Iran (Dena Mts., southern Zagros), whereas populations from central Iran (Yazd-Kerman and central Zagros) and from western Iran (central Zagros) belong to species new to science, *A. alpinum* and *A. zagricum*, respectively. Both new species are phylogenetically and morphologically close to *A. umbellatum*, with which they share unilocular fruits and one-seeded locules. However, they are easily distinguishable by leaf shape, petal size, and fruit characters. This study confirms that the alpine flora of the Irano-Anatolian region is still poorly known. As the proportion of rare and local endemic species in alpine habitats is high, these habitats are of prime interest for conservation efforts.

## 1 Introduction

Mountains are known as biodiversity hotspots with a high proportion of endemic and narrowly distributed species (Hoorn et al., 2013; Vanderplank et al., 2014; Hoorn et al., 2018; Noroozi et al., 2019a; Noroozi et al., 2019b), with alpine habitats being the centers of endemism (Testolin et al., 2021). Tectonic uplift, geographic isolation, climatic oscillations, and strong microhabitat differentiation in high mountains are among the major reasons for allopatric speciation in high elevations (Körner, 1995; Agakbanjanz and Breckle, 2002; Dirnböck et al., 2011; Sandel et al., 2011; Parisod, 2022). A particularly high rate of endemic species is found in the mountains of South-West Asia, a global biodiversity hotspot (Mittermeier et al., 2011; Noroozi et al., 2019a; Noroozi et al., 2019b), where the rate of endemism of the subalpine and alpine flora exceeds 75% (Noroozi et al., 2021). These mountains are characterized by high elevational amplitude, topographic complexity, and strong habitat isolation (Noroozi et al., 2018; Noroozi et al., 2021), likely drivers of the high plant diversity in this region. However, the high elevations of these mountains are floristically poorly explored, and it is estimated that ca. 7% of the alpine flora of the Iranian mountains are still taxonomically undescribed (Noroozi et al., 2016), indicating that the region’s diversity, although already high, is still considerably underestimated. This may be particularly the case for the isolated high mountains in Zagros in western and southwestern Iran, where during the last years, many new species have been described (such as Moazzeni et al., 2014; Moazzeni et al., 2016; Balali et al., 2019; Doostmohammadi and Mirtadzadini, 2019; Pahlevani and Amini Rad, 2019; Noroozi et al., 2020; Alipour et al., 2021).

*Aethionema* Aiton (Brassicaceae) comprises approximately 70 species distributed from the western Mediterranean region *via* South-West Asia to Central Asia, with the majority of species being found in the Irano-Anatolian region in Turkey and Iran (Al-Shehbaz et al., 2006; Mohammadin et al., 2017; Moazzeni et al., 2018). In South-West Asia, *Aethionema* species occur from lowlands up to the high alpine and subnival zones, and approximately one-third of the species are found, often exclusively, in alpine habitats (Hedge, 1965; Hedge, 1968; Moazzeni et al., 2018). The genus comprises perennial herbaceous plants and small shrubs, rarely annuals, with linear or narrowly oblong to ovate or orbicular leaves, pink, lilac, or white flowers, and glabrous mainly angustiseptate dehiscent (rarely indehiscent) silicles with one or two locules (Moazzeni et al., 2018). Morphological characters diagnostic on the species level include features of the filaments (entire, winged, or with teeth), leaves (shape), and fruits (shape, wing characters, number of locules; Moazzeni et al., 2018). Molecular phylogenetic studies confirmed that *Aethionema* is sister group to the rest of Brassicaceae (= core Brassicaceae; Couvreur et al., 2010), established the monophyly of the genus (Lenser et al., 2016; Mohammadin et al., 2017) after transferal of five traditionally recognized *Aethionema* species to *Noccaea* (Al-Shehbaz, 2012), and provided hypotheses on phylogenetic structure within the genus (Lenser et al., 2016; Mohammadin et al., 2017). Being a sister group to all other Brassicaceae, *Aethionema* has become the object for studies on multiple aspects of plant biology, such as dimorphic seeds, seed germination, and seed coat differentiation (Bhattacharya et al., 2019; Mérai et al., 2019; Arshad et al., 2021), as well as phylogeny and taxonomy (Mohammadin et al., 2017; Moazzeni et al., 2018).

In this paper, we focus on *Aethionema umbellatum* (Boiss.) Bornm., which, although known for a long time only from its locus classicus on Dena Mts. in southern Zagros, is currently considered a widely, yet disjointly distributed species (Hedge, 1968; Moazzeni et al., 2018). However, initial morphological investigations of material from different regions including the type locality indicated taxonomically relevant differences in leaf and fruit shape, suggesting that *A*. *umbellatum*, as hitherto understood, contains more than one species. To test this hypothesis, we conducted molecular phylogenetic analyses using nuclear ITS and plastid *trn*L-*trn*F sequences (as for these markers broad data sets across *Aethionema* are already available; Lenser et al., 2016; Mohammadin et al., 2017) and analyzed morphological characters, focusing on those known to be diagnostic in this genus (such as leaf shape and number of locules per fruit; Hedge, 1965; Hedge, 1968; Mohammadin et al., 2017; Moazzeni et al., 2018). Based on the results of these analyses, we taxonomically revise this group, including the description of two new *Aethionema* species.

## 2 Material and methods

### 2.1 Plant material

**Figure 1:**
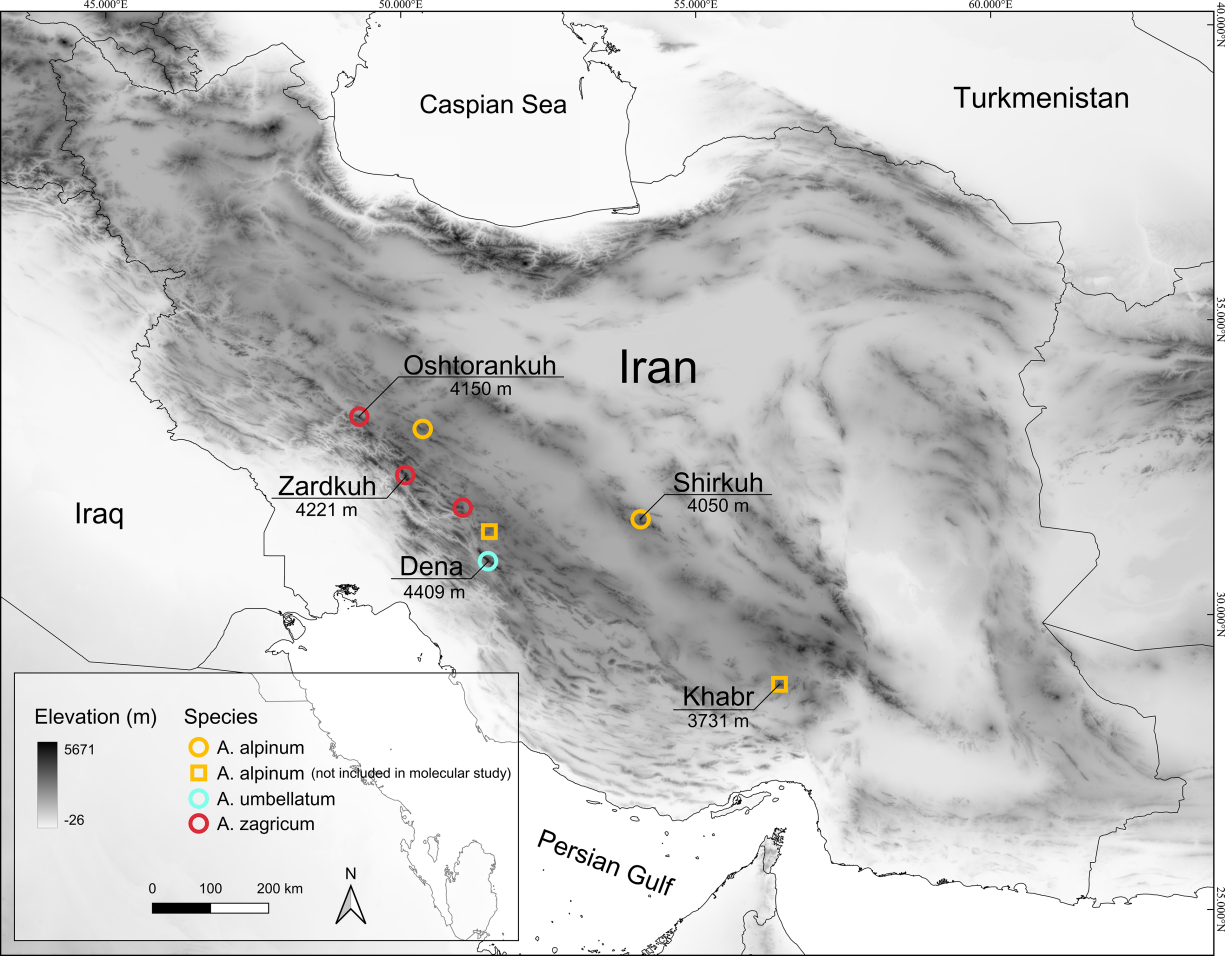
Distribution map of *Aethionema alpinum*, *A*. *umbellatum*, and *A*. *zagricum* in Iran. The highest mountain peaks of the Zagros, Yazd, and the southern Kerman mountains (where the species have been recorded) are presented on the map.

We included nine specimens identified as *A. umbellatum* [two from Shirkuh; one each from Damaneh, Zardkuh, and Oshtorankuh Mts.; and four individuals from the type location of *A*. *umbellatum*; material from the Kerman province (documented by a voucher in Edinburgh herbarium: E0061167) and one from central Zagros (W 0215578) were not available to us for molecular study; see **Figure 1**].

### 2.2 Molecular analyses

Total genomic DNA was extracted from silica-dried material or herbarium specimens using the CTAB protocol (Doyle and Doyle, 1987). Voucher information and GenBank accession numbers for all newly included specimens are listed in **Table S1**.

The internal transcribed spacer (ITS) and the *trn*L-*trn*F region were amplified and sequenced using primers 18F and 26S-82R (Mummenhoff et al., 1997) and c and f (Taberlet et al., 1991), respectively. The standard 25-μl PCR mixtures contained 1 μl of each primer (10 μm, BGI, Hong Kong, China), 12.5 μl of 2× Taq DNA Polymerase Master Mix RED (Amplicon, Denmark), 1 μl of unquantified template DNA, and 1 μl of dimethyl sulfoxide (DMSO, 78.13 g mol, Merck, Darmstadt, Germany); deionized water was added to achieve a final volume of 25 μl. Amplification was done with optimized annealing temperatures (ITS: 38°C; *trn*L-*trn*F: 51°C). Amplification products for both markers were purified using PEG (Joly et al., 2006). Sequencing was performed by BGI (Hong Kong, China) using the PCR primers.

The newly generated sequences (23 sequences) were trimmed and assembled using Geneious 6.1.2 (https://www.geneious.com). Sequences were added to the ITS and *trn*L-*trn*F data sets (including only *Aethionema* samples to avoid alignment issues with too distant outgroups) obtained from Mohammadin et al. (2017) and Lenser et al. (2016), respectively, thus including sequences from jointly 54 accessions (**Table S1**). Based on previous results (Mohammadin et al., 2017 and references therein), *A. spinosum* and *A. lepidioides* were used as outgroups. Both ITS and *trn*L-*trn*F data sets were aligned with MAFFT 1.3 as implemented in Geneious 6.1.2 under default settings. Alignments were manually corrected in Geneious 6.1.2. Using the AIC criterion as implemented in jModelTest 2.1.4 (Darriba et al., 2012), the GTR + G + I and GTR + G models were chosen as best-fit substitution models for the ITS and the *trn*L-*trn*F alignments, respectively. All aligned matrices are available in **Data Sheet 2** of the **Supplementary Material**.

Phylogenetic analyses were performed using maximum likelihood (ML) and Bayesian inference (BI). The ML analysis was carried out on the IQ-TREE web server (http://iqtree.cibiv.univie.ac.at/; Minh et al., 2020) using the default settings, assessing branch support *via* 1,000 bootstrap replicates employing the ultrafast bootstrap approximation (UFBoot; Minh et al., 2013). BI was performed using MrBayes 3.2.7 (Ronquist et al., 2012) at the CIPRES portal (https://www.phylo.org/index.php; Miller et al., 2010) with default prior settings, running the four chains for 10 million MCMC generations each sampling every 1,000 generations. The quality of the analysis was checked by comparing likelihood values and parameter estimates from different runs in Tracer 1.6 (http://tree.bio.ed.ac.uk/software/tracer/) as well as checking the average standard deviation of split frequencies (to be <0.01) and ESS values (to be >200). After discarding the initial 25% of trees as burn-in, the remaining trees were summarized in a 50% majority-rule consensus tree.

After a preliminary phylogenetic analysis, samples with ambiguous phylogenetic positions were removed (**Table S1**). Due to the general morphological similarity among *Aethionema* species (Moazzeni et al., 2018) and/or considerable intraspecific morphological variation (Andersson et al., 1983), misidentification may be the main reason for the dubious phylogenetic placement of some *Aethionema* samples. As we did not have access to the vouchers of these samples and thus could not check their identification, we adopted the strategy to remove them.

### 2.3 Morphological analyses

The evolution of two morphological characters (leaf shape and locus number), which are important features to identify *Aethionema* species, was inferred using ML-based ancestral state reconstructions as implemented in Mesquite 3.70 (Maddison and Maddison, 2021) on the better-resolved phylogenetic hypothesis inferred from *trn*L-*trn*F sequences. To this end, leaf shape was coded as linear or narrowly oblong *versus* ovate, obovate, or spathulate, while locus number was coded as unilocular, bilocular, or both. ML reconstructions were performed under a single-rate Mk likelihood model for discrete morphological characters, described by Lewis (2001). The “trace character over trees” option was used to summarize the ancestral state reconstructions over the set of posterior trees.

## 3 Results

### 3.1 Molecular phylogeny

**Figure 2:**
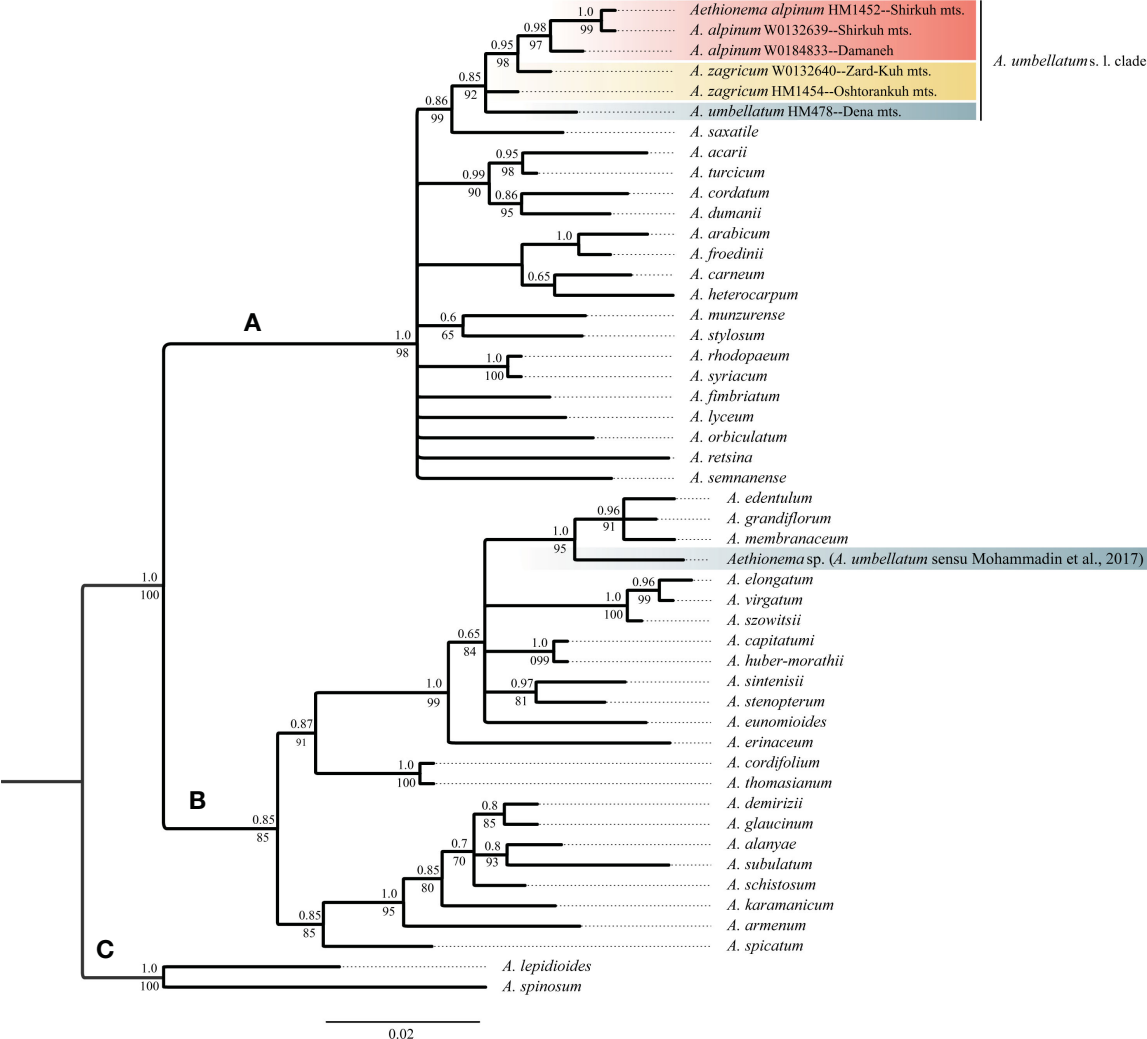
Majority rule consensus tree of Bayesian analysis of nuclear ITS sequences showing the phylogenetic position of *Aethionema alpinum*, *A. zagricum*, and *A. umbellatums*. str. Numbers above the branches indicate posterior probabilities, and those below the branches indicate maximum likelihood bootstrap values; capital letters indicate major clades.

**Figure 3:**
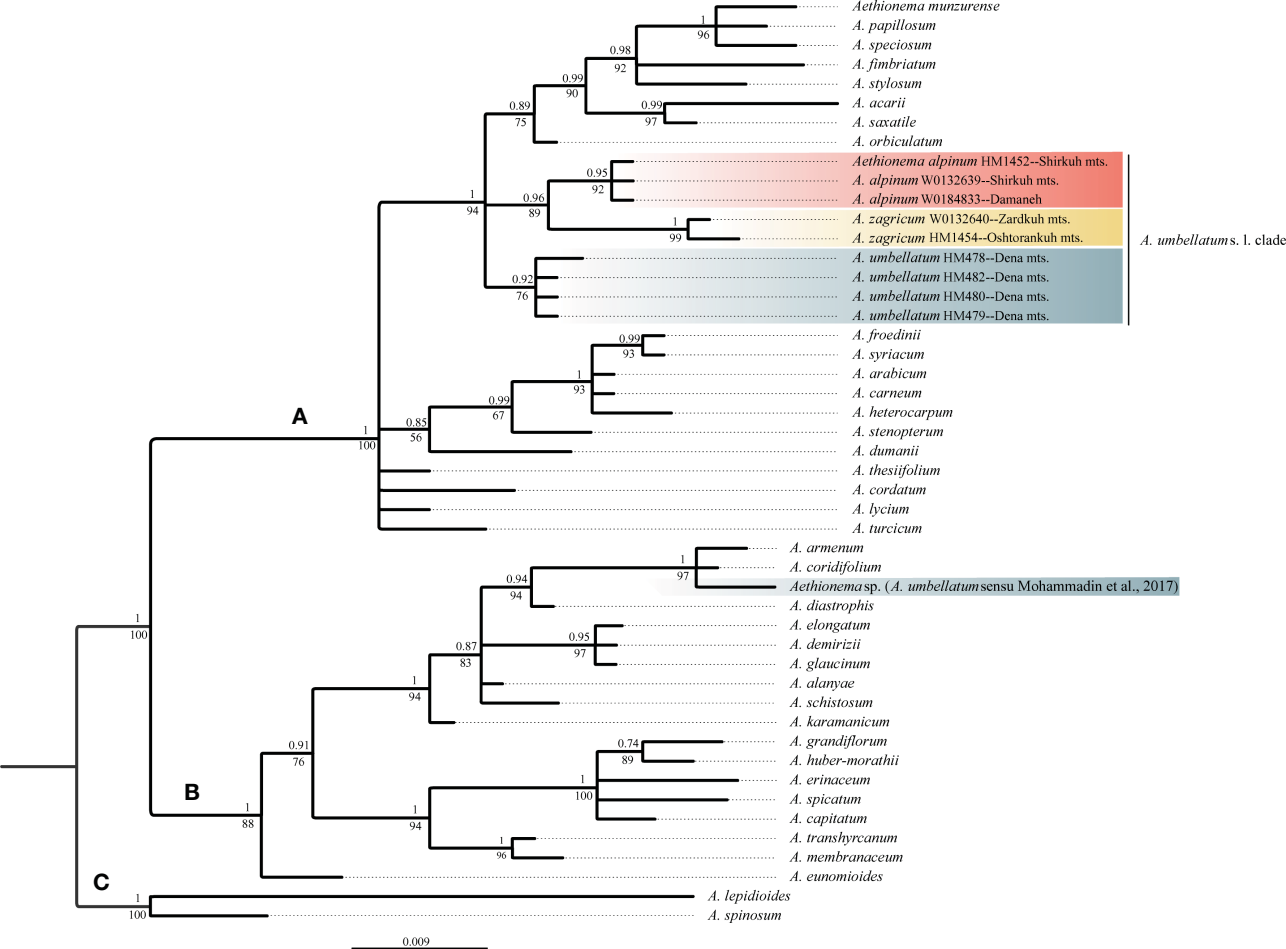
Majority rule consensus tree of Bayesian analysis of plastid trnL-trnF sequences showing the phylogenetic position of *Aethionema alpinum*, *A. zagricum*, and *A. umbellatum* s. str. Numbers above the branches indicate posterior probabilities, and those below the branches indicate maximum likelihood bootstrap values; capital letters indicate major clades.

The newly obtained sequences are available in GenBank under accession numbers OQ695471–OQ695480 for ITS and OQ726488–OQ726500 for *trn*L-*trn*F. Tree topologies obtained *via* maximum likelihood and Bayesian Inference were essentially identical for both markers (**Data Sheet 2** of the **Supplementary Material**). In both data sets, *Aethionema* split into three moderately to well-supported main clades [bootstrap support (BS)/posterior probability (PP) of 85–100/0.85–1.0; **Figures 2**, **3**] in agreement with previous studies based on plastome and rRNA-cistron data (Lenser et al., 2016; Mohammadin et al., 2017). All samples presumably identified as *A*. *umbellatum* were placed within clade A, where they, as inferred from ITS data (**Figure 2**), formed a monophyletic group (BS/PP 92/0.85) or, as inferred from *trn*L-*trn*F data (**Figure 3**), were diphyletic (*A. umbellatum* from Dena Mts.: BS/PP 76/0.92; *A. umbellatum* from elsewhere: BS/PP 89/0.96) in a trichotomy (BS/PP 94/1) with a clade including, among others, *Aethionema saxatile* (BS/PP 75/0.89), the species inferred as a sister (BS/PP 99/0.88) to the *umbellatum* clade by ITS data (**Figure 2**). Whereas the accessions from Damaneh plus Shirkuh and from Dena Mts. formed clades (BS/PP 76–99/0.92–0.98), those from Zardkuh plus Oshtorankuh Mts. were inferred as clade only by the *trn*L-*trn*F data (BS/PP 99/1; **Figure 3**).

### 3.2 Ancestral character state reconstruction

**Figure 4:**
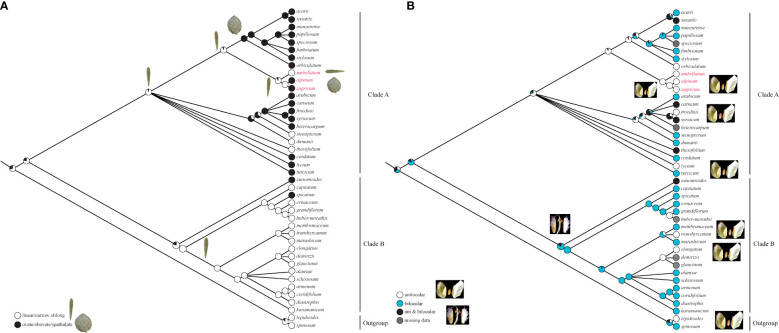
Evolution of **(A)** leaf shape and **(B)** the number of locules per fruit in *Aethionema* inferred using maximum likelihood on the Bayesian tree inferred from plastid *trn*L-*trn*F sequences.

From the ancestral leaf shape, inferred to be linear or narrowly oblong, there were two changes to a broader leaf shape in single species in clade B. In clade A, ovate, obovate, or spathulate leaf shapes dominated, only a few species, including *A. umbellatum* from Dena Mts., retaining the ancestral state of linear to narrowly oblong leaves (**Figure 4A**). Starting from bilocular fruits, estimated to be the ancestral state in *Aethionema*, changes to unilocular fruits were inferred in the outgroup, several times independently in clade B, and on the branch leading to the ancestor of clade A (**Figure 4B**). Within clade A, several reversals to bilocular fruits were inferred, but *A. umbellatum* s. l. retained the ancestral condition of clade A, i.e., unilocular fruits.

### 3.3 Taxonomic treatment

Based on the phylogenetic results and inspection of 13 specimens of members of *A. umbellatum* s. l., we taxonomically revise this group to include three species, two of which are (based on five and three specimens, respectively) described herein as new to science.

**Figure 5:**
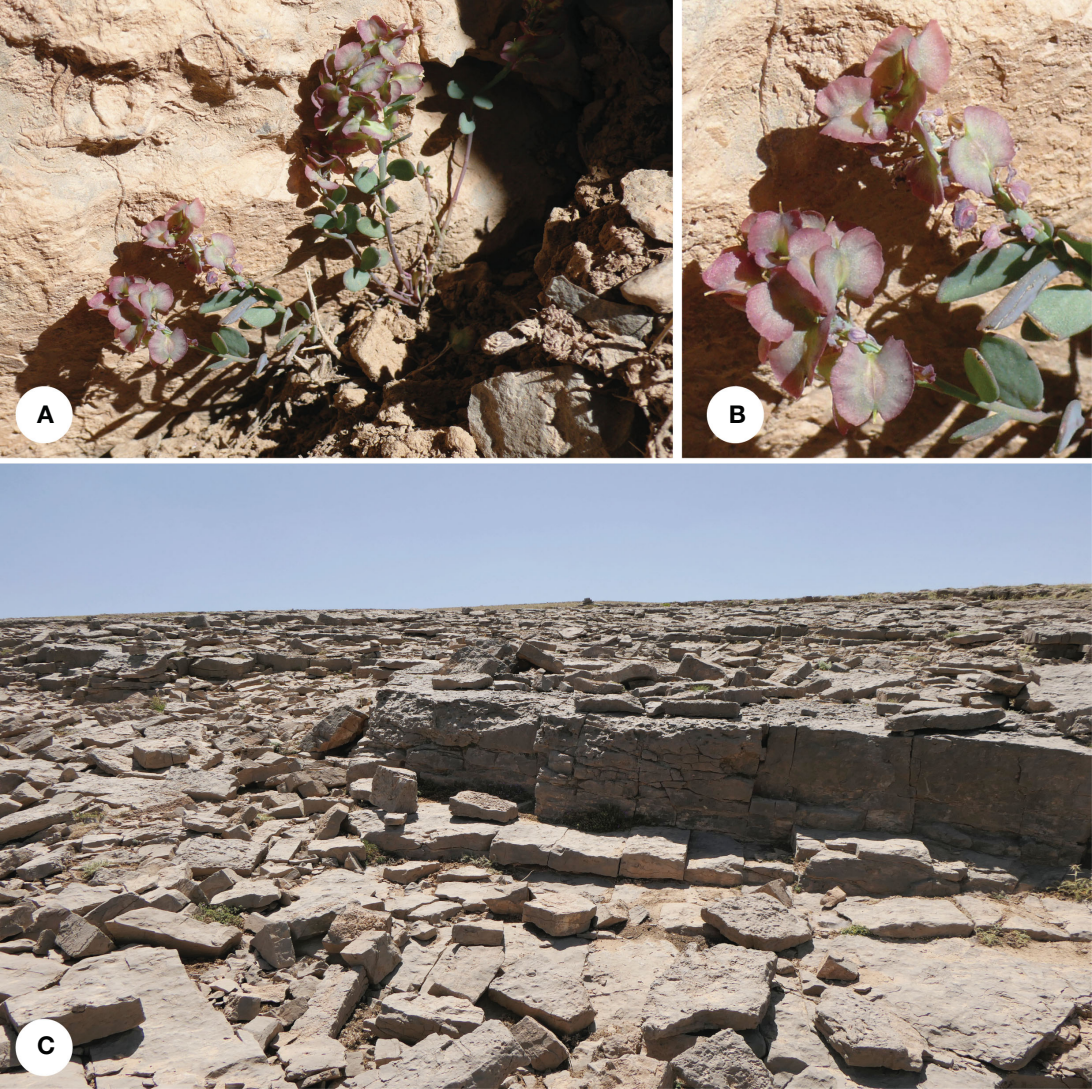
*Aethionema alpinum*: **(A)** plants in their natural habitat, **(B)** close-up of the fruits, and **(C)** habitat (Shirkuh Mt. 3,800 m a.s.l.; photographs by JN).

***Aethionema alpinum*** Moazzeni & Noroozi sp. nov. (**Figure 5**).

**Type**: Iran, Yazd, Shirkuh Mts., 31°37′N; 54°04′E, 3,800 m a.s.l., 2012 July 05, *J. Noroozi & M. Mahmoodi* 2843 (Holotype: W 0132639, Isotype: TARI [98638-TARI])

**Diagnosis: —** Differt ab A. umbellato foliis majoribus et latioribus, foliorum mediorum alternantibus, fructibus minoribus et angustioribus, fructibus alatis undulate.

**Description**: Low-growing perennial herbs, (4–)6.5–7 cm tall. Lower leaves opposite, middle and upper ones alternate, ovate to rarely elliptic, cuneate at the base, rounded at the apex, 4–8 × (1.2–)1.5–2(2.5) mm, entire. Flower characters not known (no flowering material available). Infructescence 10–17 mm long; fruiting pedicels 2–3(–4) mm long, erect. Fruit suborbicular, unilocular, one-seeded; wing undulate, 1.5–2 mm wide, developed over the entire circumference, apical notch ca. 1–1.5 mm deep; locule 3–3.5 × 3–3.5 mm; style 0.6–1 mm long. Seeds ca. 1.5 × 1 mm.

**Phenology**: Flowering probably in June and July (we have not seen flowers) and fruiting in June and July.

**Distribution and ecology**: This plant is known from several collections in high alpine habitats, growing on calcareous bare grounds dominated by rocks and big stones (**Figure 5C**). This species is limited to the high mountains of central Iran (Yazd-Kerman mountains) and the eastern side of Zagros. These mountains have a more continental climate and receive less precipitation compared with the western sides and the central parts of Zagros.

**Etymology**: The epithet “alpinum” refers to the alpine habitats the species is restricted to.

**Conservation status**: Based on the current data, *A*. *alpinum* is restricted to an area of 6.4 ha [extent of occurrence (EOO) = 63,892 km^2^, area of occurrence (AOO) = 16 km^2^]; thus, following the IUCN Red List criteria (IUCN, 2022), it is categorized as Least Concern (LC).

**Additional specimens**: Iran, Esfahan, in montibus prope Damaneh 35 km SE Daran, 115 km NW Esfahan, substr. calc. 1974.06.10, J. Renz 47646 (W 0184833); Iran, Kerman, Kuh-e Khabr, 1977.06.08, Assadi, Edmonson & Miller, 1760 (E00061167); Persia: W: Qashqai: Pashma Kuh, a Semirom 5 km occidentem *versus*, substr. calc., 2,700–3,000 m; 1974 June 06, *K.H. Rechinger* 47450 (W 0215578).

**Figure 6:**
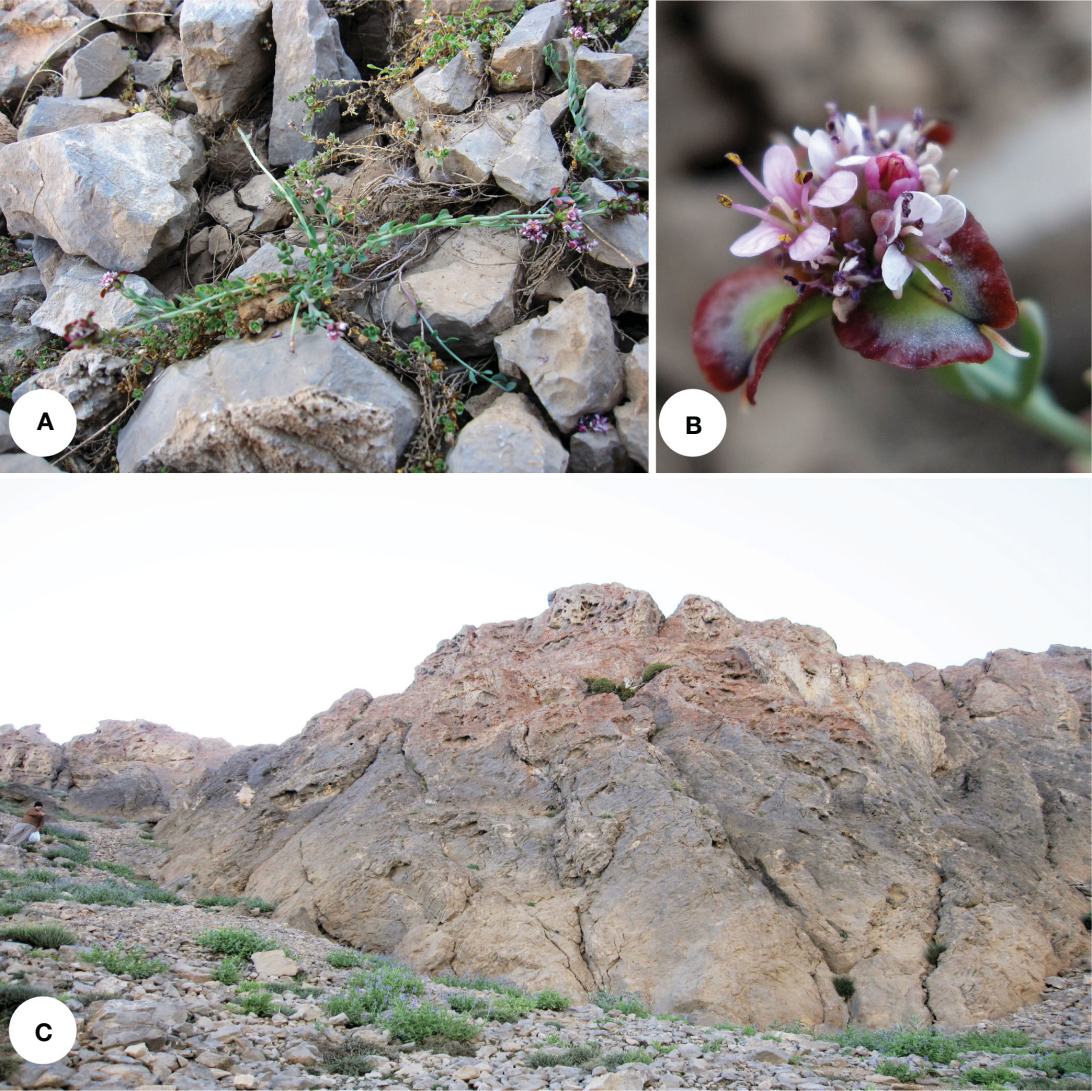
*Aethionema zagricum*: **(A)** plant in its natural habitat, **(B)** close-up of the flowers and fruits, and **(C)** habitat (Oshtorankuh Mt., 3,700 m a.s.l.; photographs by MM).

***Aethionema zagricum*** Moazzeni & Mahmoodi sp. nov. (**Figure 6**).

**Type**: Iran, Lorestan, Azna, Oshtorankuh, between Bidesraneh village and Sanboran summit, 3,700 m a.s.l., 2012.07.23, *Mahmoodi and Hoseini* (Holotype: TARI-98504)

**Diagnosis**: **—** Differt ab A. umbellato et A. alpino inflorescentiis majoribus, stylis longioribus et alis fructuum angustioribus, et a speciebus Turcicis foliis et petalis brevioribus et angustioribus.

**Description**: Low-growing perennial herbs, 7–13 cm tall. Lower leaves opposite, upper and middle alternate, elliptic to oblong to rarely obovate, cuneate at the base, rounded at the apex, 4–7 × 1.5–2 mm, entire. Racemes with up to 15 flowers. Sepals 2–3 mm long; petals spathulate, pink, 3–3.5 × 1–1.3 mm, emarginate at the apex; median filaments 3 mm long, lateral ones 2.5 mm long, entire. Infructescence 10–17 mm long; fruiting pedicels 2–3(–4) mm long, erect. Fruit orbicular to rarely suborbicular, unilocular, one-seeded; wing more or less crenulate, 0.5–1.3 mm wide, developed over the entire circumference, apical notch ca. 0.2 mm deep; locule 3.5–5.5 × 3.5–5.5 mm; style 1–1.5 mm long. Seeds not seen.

**Phenology**: Flowering and fruiting in July and August.

**Distribution and ecology**: Endemic to the alpine and subnival zones of central Zagros, growing on limestone screes above 3,700 m a.s.l. The mountains in this region (Zardkuh and Oshtorankuh) receive a high amount of precipitation (more than 1,500 mm per year), mostly as snow during the wintertime, but the growing season is usually dry (Mediterranean precipitation regime).

**Etymology**: The epithet “zagricum” refers to the Zagros Mountain range, where the new species is restricted to.

**Conservation status**: The new species is known so far only from the highest elevations of Mts. Oshtorankuh and Zardkuh above 3,700 m. Since the highest summits of these mountains are 4,150 and 4,221 m, respectively, the species is strongly restricted elevationally and geographically. As *A. zagricum* is restricted to a small area of 2.8 ha (EOO = 2,857 km^2^, AOO = 12 km^2^), following the IUCN Red List criteria (IUCN 2022), it is categorized as Endangered (EN).

**Additional specimens**: Iran, Chahār Mahāl va Bakhtīārī, In lapide cacum Kellal, 1868.09, Haussknecht, s.n. (JE 00034617); Zardeh-Kuh, above Kurrang Valley: N.E. facing slope in stony clay, immediately below extensive snow fields. 4,265 m, 1966.08.05 Archibald, 2968 (W0132640).

**Figure 7:**
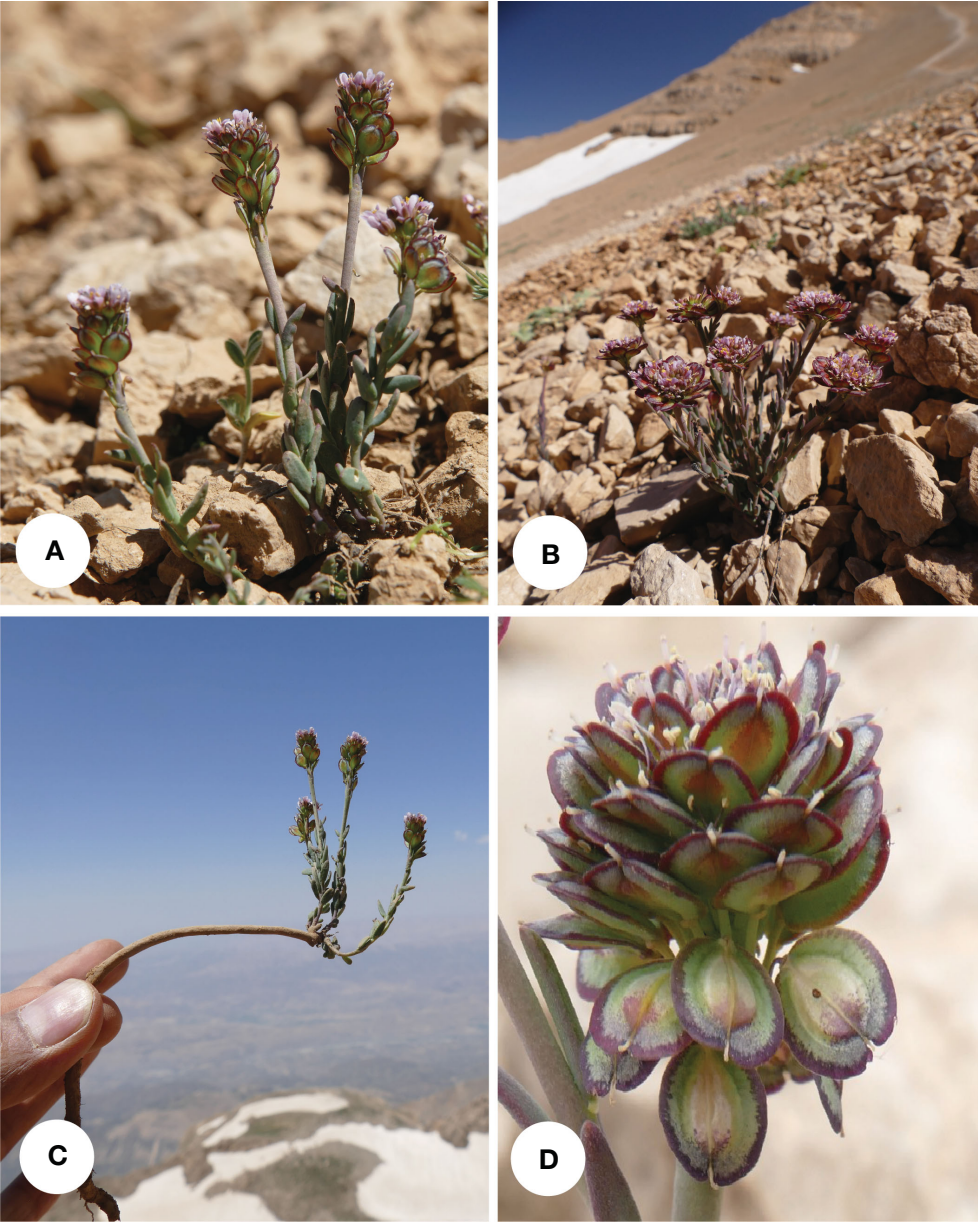
*Aethionema umbellatum*: **(A, B)** plant in its natural habitat, **(C)** habit, and **(D)** close-up of the fruits (Dena Mt., 4,100 m a.s.l.; photographs by JN).

***Aethionema umbellatum*** (Boiss.) Bornmüller (1911c: 535) (**Figure 7**)

≡ *Moriera umbellata* Boissier (1846: 16) ≡ *Crenularia umbellata* (Boiss.) Boissier (1867: 338). — Holotype: IRAN. Persia, in glareosis reg[ionum] summarum alpis Kuh-Daëna [Dena], *Kotschy 780* (G-BOIS [G00154010]; isotypes: B [B100241892], BM [BM001254112], E [E00061169, E00061170], FI [FI005705], G [G00002123, G00002124], K [K000075747], KW [KW000127991], LE [LE00012860], MO [MO2001061, MO2001062], P [P00835122, P00835123, P00835124, P00835126, P00835127], W [W0005554, W18890020458], WAG [WAG0003966]).

Low-growing perennial herbs, (4–)5–13 cm tall, with a pleiocorm, stems glabrous. Leaves sessile, linear-elliptic, cuneate at the base, rounded at the apex, 4–6 × c. 1 mm, entire, lower and middle opposite, upper alternate. Racemes with up to 30 flowers. Sepals 2–3 mm long; petals spathulate, purple, 2.5–3 × 1.5–2 mm, emarginate at the apex; median filaments 1.5 mm long, lateral ones 1 mm long, entire. Infructescence 8–15 mm long; fruiting pedicels 1–3(–4) mm long, erect. Fruit suborbicular to elliptic, unilocular, one-seeded; wing entire, 1 mm wide, developed over the entire circumference, apical notch ca. 0.5 mm deep; locule 2.5–3.5 × 2–3 mm; style 1 mm long. Seeds ca. 1.5 × 1 mm.

**Additional specimens**: Iran: Kohgiluyeh and Boyer-Ahmad Province, Sisakht, Dena Mts., near the summit of Hoz-Dal in southern slopes. 30.9058°N; 51.4787°E, 4,000–4,300 m a.s.l., 2019.07.19, *Jalil Noroozi* 4059 (W! duplicate FUMH)!.

**Phenology**: Flowering and fruiting in July and August.

**Distribution and habitat**: It is a local endemic of Dena Mts., southern Zagros, adapted to limestone screes of the subnival zone (**Figures 7A, B**). The region receives a high amount of annual precipitation as in central Zagros. The accompanying plants of *A. umbellatum* are *Astragalus melanodon* Boiss. (Fabaceae), *Bromus frigidus* Boiss. & Hausskn. (Poaceae), *Chenopodium foliosum* Asch. (Amaranthaceae), *Elymus longearistatus* (Boiss.) Tzvelev (Poaceae), *Euphorbia aucheri* Boiss. (Euphorbiaceae), *Galium pseudokurdicum* (Ehrend.) Schönb.-Tem. (Rubiaceae), *Nepeta lasiocephala* Bwnth. (Lamiaceae), *Physoptychis gnaphalodes* (DC.) Boiss. (Brassicaceae), *Potentilla flaccida* Th.Wolf ex Bornm. (Rosaceae), *Pseudocamelina aphragmodes* (Boiss.) N. Busch (Brassicaceae), *Silene daenensis* Melzh. (Caryophallaceae), and *Zerdana anchonioides* Boiss. (Brassicaceae).

**Conservation status**: *Aethionema umbellatum* is known only from the highest elevations of Dena Mts. above 4,000 m. We estimated the population size to be 20 to 50 individuals. As *A. umbellatum* is restricted to a small area of 4 km^2^ (EOO = incomputable, AOO = 4 km^2^), following the IUCN Red List criteria (IUCN 2022), it is categorized as Critically Endangered (CR).

## 4 Discussion

### 4.1 Underestimated diversity in *Aethionema umbellatum* sensu lato

Our study complements recent studies that the alpine flora in SW Asia, although widely recognized as highly diverse and rich in endemics (Noroozi et al., 2016; Noroozi et al., 2021), is still poorly explored. This is well exemplified here in *A. umbellatum* s. l., where morphological and molecular data uncovered cryptic species diversity allowing three instead of a single species to be recognized.

Whereas molecular data do not reject current taxonomy (i.e., a broadly defined *A. umbellatum*), the geographic structure of phylogenetic lineages as well as morphological data suggest the presence of several distinct species within *A. umbellatum* s. l. (**Table 1**). The combination of ovate leaves (common in species of clade A, but rare in clade B: **Figure 4A**; Mohammadin et al., 2017) and unilocular fruits (relatively common in clade A, but rare in clade B: **Figure 4B**) separates *A. alpinum* and *A. zagricum*, which differ from each other in fruit size and leaf number (**Table 1**), from *A. umbellatum* s. str. (**Figure 4**). Such a discrepancy between diagnostically relevant morphological data (here leaf shape and the number of locules per fruit) and molecular data is seen in many cruciferous genera (Meyer, 1973; Mummenhoff et al., 1997; Moazzeni et al., 2007; Moazzeni et al., 2010), and confirms that morphological characters in Brassicaceae often are homoplaseous, although they can be useful features for species identification (Franzke et al., 2011; Al-Shehbaz, 2012; Huang et al., 2016; Walden et al., 2020).

**Table 1:** Comparison of the morphological characteristics of *Aethionema alpinum*, *A. umbellatum*, *A. zagricum*, and their most similar relatives.

Characters	*A*. *umbellatum*	*A*. *alpinum*	*A*. *zagricum*	*A*. *lycium*	*A. demirizii*	*A. polygaloides*
**Plant height (cm)**	(4–)5–13	up to 10	7–13	up to 10	8–13	6–10
**Leaf shape**	linear	ovate-elliptic	narrowly elliptic to oblong, rarely obovate	obovate to elliptic	spathulate	elliptic
**Leaf length (mm)**	4–6	4–8(10)	4–7	5–7	5–8	c. 5–10
**Leaf width (mm)**	c. 1	(1.2)1.5-2(2.5)	1.5–2	2–3.5	1–1.5	c. 1–3
**Petal color**	pale pink	unknown	pink	pale pink	pale pink	pale pink
**Petal length (mm)**	2.5–3	unknown	3–3.5	c. 4.5	4–4.5	5
**Fruiting pedicel length (mm)**	1–3(4)	1–1.5	2.5–3.5	4–6	n.a.	3–5
**Fruit shape**	suborbicular to elliptic	suborbicular	orbicular, rarely suborbicular	broadly obovate to suborbicular	suborbicular	suborbicular
**Fruit size (mm)**	3–4 × 3–4	3–3.5 × 3–3.5	3.5–5.5 × 3.5–5.5	3 × 3.5	3–3.5 × 1.25	3–4 × 3–5
**Distribution**	SW Iran	SW Iran	SW Iran	SW Turkey	S Turkey	N Turkey

The characters are based on studies of the four known populations of *A. alpinum* (five individuals), one population of *A. umbellatum* (five individuals), and two populations of *A. zagricum* (three individuals).

With respect to habit, fruit size, and shape as well as the number of locules per fruit and seeds per locule, *A. alpinum* and *A. zagricum* resemble, apart from *A. umbellatum* s. str., some species from Turkey, such as *A*. *demirizii*, *A*. *lycium*, and *A*. *polygaloides*. *Aethionema alpinum* can be readily distinguished from *A. umbellatum* s. str. by larger and broader leaves, by alternate phyllotaxis of the middle leaves, and by smaller and narrower fruits that have deeper notches and from the Turkish species by their shorter height and broader fruits (**Table 1**). *Aethionema zagricum* can be distinguished from *A*. *umbellatum* s. str. and from *A. alpinum* by larger inflorescences, longer styles, and narrower fruit wings and from the Turkish species by having shorter and narrower leaves and petals (**Table 1**).

The accession of *A. umbellatum* used by Mohammadin et al. (2017) not only did not group with the other accessions of this species collected at its type locality, but actually fell in a different major clade (clade B instead of clade A; **Figures 2**, **3**). The plants used by Mohammadin et al. (2017) were collected from Chalus road in northern Iran (Lenser et al., 2016), but a voucher specimen of *A*. *umbellatum* is missing (although intended to be stored at the herbarium of Wageningen University: Lenser et al., 2016). Extensive field studies in the Chalus Road area as well as investigation of herbarium specimens located in main Iranian herbaria (such as TARI, TUH, and IRAN) have never shown the presence of *A. umbellatum* s. l. in this region, suggesting that the plant used by Mohammadin et al. (2017) has been misidentified and actually belongs to a different species, whose identity cannot be ascertained without the voucher specimen.

### 4.2 Determination key

Key to the low-growing (up to 30 cm tall) perennial species of *Aethionema* with unilocular fruits and one seed per locus (species marked with an asterisk are endemic to Iran, the remaining ones are endemic to Turkey):

1. Filaments with a conspicuous tooth in the upper third; fruit cymbiform ...............................................................A. sabzevaricum*

- Filaments entire; fruit not cymbiform .......................................2

2. Leaves < 10, fruit broad, 4–5.5 mm wide ............*A*. alpinum*

- Leaves at least 10, fruit 3–3.5 mm wide ....................................3

3. Leaves linear, petals 2–3.5 mm long .........................................4

- Leaves ovate to spathulate, petals 4.5–5.5 mm long ...............5

4. Leaves c. 1 mm wide, raceme with up to 30 flowers, petals pale pink, 2.5–3 mm long, style ≤1 mm long, fruit wing entire ...................................................................................A. umbellatum*

- Leaves 1.5–2 mm wide, raceme with up to 15 flowers, petals lilac, 3–3.5 mm long, style 1–1.5 mm long, fruit wing more or less crenulate ...................................................................A. zagricum*

5. Leaves spathulate, fruit 3 mm wide.........................*A. demirizii*

- Leaves ovate to obovate, fruit 1.5 mm wide ..............................6

6. Lower leaves alternate, ovate; fruit suborbicular *.....................................................................................A. polygaloides*

- Lower leaves opposite, elliptic to obovate to elliptic, fruit broadly obovate ....................................................................*A. lycium*

## 5 Conclusion

Our explorations over the last years of high elevations of the mountains of Iran have resulted in the description of several species new to science (Noroozi et al., 2010; Mahmoodi et al., 2013; Noroozi and Ajani, 2013; Noroozi et al., 2020), confirming that the alpine and subnival flora of the Irano-Anatolian biodiversity hotspot is still insufficiently known. The proportion of rare and local endemic species at high elevations is very high (Noroozi et al., 2021), rendering these habitats very important for conservation biology. This is exacerbated considering that the region is under a high pressure by human activities mainly overgrazing and ongoing climate change, but at the same time suffers from poor conservation management (Jowkar et al., 2016). The three species studied in this manuscript and many other rare and local endemic species restricted to the upper limit of vascular plants of these mountains have no alternative habitats to ascend to under the impact of climate change. Therefore, high attention to the conservation of the biodiversity of these habitats is strongly recommended.

## Data availability statement

The datasets presented in this study can be found in online repositories. The names of the repository/repositories and accession number(s) can be found in the article/**Supplementary Material**.

## Author contributions

HM and JN developed the idea. HM conducted the final morphological and molecular analyses. HM and JN wrote the draft of the manuscript and all authors contributed to the editing process. All authors contributed to the article and approved the submitted version.
