# Supplementary material for: Underestimated diversity in high elevations of a global biodiversity hotspot: two new endemic species of Aethionema (Brassicaceae) from the alpine zone of Iran
Source: Front Plant Sci. 2023 May 26;14:1182073. doi: 10.3389/fpls.2023.1182073 (PMC10250747; doi:10.3389/fpls.2023.1182073)
Supplement: Supplementary file 1 [file DataSheet_1.docx]

**Table S1:** Voucher information and GenBank accession numbers for accessions used for molecular phylogenetic analyses. Sequences taken from Lenser et al. (2016) and Mohammadin et al. (2017) do not have an accession number. The new sequences indicated by asterisk.

| Species | Collector and number | Location | ITS | *trn*L-*trn*F | |
| --- | --- | --- | --- | --- | --- |
| *Aethionema acarii* | Y. Gemici 7694 | Kütahya, Turkey | Mohammadin et al 2017 | Lenser et al. 2016 | |
| *A. alpinum** | Noroozi & Mahmoudi (TARI-98638) | Yazd, Shirkuh mts. | OQ695479 | OQ726499 | |
|  | Noroozi & Mahmoudi (W 0132639) | Yazd, Shirkuh mts. | OQ695480 | OQ726500 | |
|  | J. Renz 47646 (W 0184833) | Esfahan, Damaneh | OQ695478 | OQ726498 | |
| *A. arabicum* | - | Ankara, Turkey | Mohammadin et al 2017 | Lenser et al. 2016 | |
| *A. capitatum* | A.A. Dönmez 4494 | Mersin, Turkey | Mohammadin et al 2017 | Lenser et al. 2016 | |
| *A. carneum* | TNRC 2636 | - | Mohammadin et al 2017 | Lenser et al. 2016 | |
| *A. cordatum* | M.E. Schranz 20.1 | Ankara, Turkey | Mohammadin et al 2017 | Lenser et al. 2016 | |
| *A. coridifolium* | A.A. Dönmez 5371 | Erzincan | Mohammadin et al 2017 | Lenser et al. 2016 | |
| *A. demirizii* | N. Adıgüzel 3010 | İçel, Turkey | Mohammadin et al 2017 | Lenser et al. 2016 | |
| *A. diastrophis* | Ö. İnceoğlu | Ankara, Turkey | Mohammadin et al 2017 | Lenser et al. 2016 | |
| *A. dumanii* | M. Vural 4162 | Ankara, Turkey | Mohammadin et al 2017 | Lenser et al. 2016 | |
| *A. eunomioides* | H.D & Z.A 4300 | Kayseri, Turkey | Mohammadin et al 2017 | Lenser et al. 2016 | |
| *A. fimbriatum* | A.A. Dönmez 9707 | Hakkari, Turkey | Mohammadin et al 2017 | Lenser et al. 2016 | |
| *A. froedinii* | A.A. Dönmez14361 | Diyarbakır, Turkey | Mohammadin et al 2017 | Lenser et al. 2016 | |
| *A. glaucinum* | N. Adıgüzel 3131 | Niğde, Turkey | Mohammadin et al 2017 | Lenser et al. 2016 | |
| *A. grandiflorum** | H. Moazzeni TUH35993 | East Azarbaijan, Mishu Dagh | OQ695474 | OQ726491 | |
| *A. heterocarpum* | A.A. Dönmez 5094 | Şanlıurfa, Turkey | Mohammadin et al 2017 | Lenser et al. 2016 | |
| *A. huber-morathii* | N. Adıgüzel 3007 | Seyhan, Turkey | Mohammadin et al 2017 | Lenser et al. 2016 | |
| *A. karaminicum* | K. Ertuğrul 1215 | Karaman, Turkey | Mohammadin et al 2017 | Lenser et al. 2016 | |
| *A. lepidioides* | N. Adıgüzel 4992 | Sivas, Turkey | Mohammadin et al 2017 | Lenser et al. 2016 | |
| *A. lycium* | N. Adıgüzel 3254 | Antalya, Turkey | Mohammadin et al 2017 | Lenser et al. 2016 | |
| *A. marashicum* | B. Yıldız 1037 | Kahramanmaraş, Turkey | Mohammadin et al 2017 | Lenser et al. 2016 | |
| *A. membranaceum** | M. Joharchi and H. Zangooei 40650 FUMH | Iran, North Khorassan | OQ695473 | OQ726490 | |
| *A. munzurense* | P.H. Davis 31296 | Tunceli, Turkey | Mohammadin et al 2017 | Lenser et al. 2016 | |
| *A. orbiculatum* | D. Phitos 1963 | Greece | Mohammadin et al 2017 | Lenser et al. 2016 | |
| *A. papillosum* | Balls 1096 A.A. | - | Mohammadin et al 2017 | Lenser et al. 2016 | |
| *A. saxatile* | AY122451 | NCBI Genbank | Mohammadin et al 2017 | Lenser et al. 2016 | |
| *A. speciosum* | B. Yıldız 1354 | KahramanMaraş, Turkey | Mohammadin et al 2017 | Lenser et al. 2016 | |
| *A. spicatum* | N. Adıgüzel 3135 | Niğde, Turkey | Mohammadin et al 2017 | Lenser et al. 2016 | |
| *A. spinosum** | LE s.n, | Turkmenistan | OQ695472 | OQ726489 | |
| *A. schistosum* | A.A. Dönmez 13862 | Antalya, Turkey | Mohammadin et al 2017 | Lenser et al. 2016 | |
| *A. stenopterum* | E. Schranz 2.10 | Chalus, Iran | Mohammadin et al 2017 | Lenser et al. 2016 | |
| *A. stylosum* | N. Adıgüzel 3121 | Niğde, Turkey | Mohammadin et al 2017 | Lenser et al. 2016 | |
| *A. syriacum* | A.A. Dönmez 10742 | Siirt, Turkey | Mohammadin et al 2017 | Lenser et al. 2016 | |
| *A. thesiifolium* | N. Adıgüzel 5233 | Burdur, Turkey | Mohammadin et al 2017 | Lenser et al. 2016 | |
| *A. transhyrcanum* | Memariani and Zangooei 39617 FUMH | Iran, North Khorassan | OQ695471 | OQ726488 | |
| *A. turcicum* | N. Adıgüzel 2789 | Ankara, Turkey | Mohammadin et al 2017 | Lenser et al. 2016 | |
| *A. umbellatum** | J. Noroozi, 4059 | Iran, Kohgiluye and Boyer Ahmad | OQ695477 | OQ726494- OQ726497 | |
| *Aethionema* sp. | E. Schranz , s.n. | Iran, Chalus road | Mohammadin et al 2017 |  | |
| *A. zagricum** | Mahmoodi and Hoseini (TARI-98504) | Lorestan, Azna, Oshtorankuh Mts. | OQ695475 | OQ726497 | |
|  | Archibald, 2968 (W0132640). | Lorestan, Zardeh-Kuh Mts. | OQ695476 | W0132640 | |
| *Excluded sequences* | | | | |  |
| *A. coridifolium* |  |  | MT799725 | - |  |
| *A. grandiflorum* |  |  | MW791193 | - |  |
| *A. hubermorathii* |  |  | MT799726 | - |  |
| *A. memebranaceum* |  |  | 33-copen | - |  |
| *A. sp* |  |  | MW791188 | - |  |
| *A. spinosum* |  |  | GQ424545 |  |  |
| *A. thomasianum* |  |  | MF543734^-40^ | - |  |
