# Supplementary material for: Underestimated diversity in high elevations of a global biodiversity hotspot: two new endemic species of Aethionema (Brassicaceae) from the alpine zone of Iran
Source: Front Plant Sci. 2023 May 26;14:1182073. doi: 10.3389/fpls.2023.1182073 (PMC10250747; doi:10.3389/fpls.2023.1182073)
Supplement: Supplementary file 2 [file DataSheet_2.zip › Date Sheet 2/Character-mapping/character_matrix_majority_rule_consensus_tree_NEXUS_format.docx]

#NEXUS

[written Thu Jun 23 16:26:23 IRDT 2022 by Mesquite version 3.70 (build 940) at DrMoazzeni-PC/172.22.36.2]

BEGIN TAXA;

TITLE Taxa;

DIMENSIONS NTAX=42;

TAXLABELS

acarii alanyae arabicum armenum capitatum carneum cordatum coridifolium demirizii diastrophis dumanii elongatum erinaceum eunomioides fimbriatum froedinii glaucinum grandiflorum heterocarpum huber_morathii subalpinae shirkuhense karamanicum lepidioides lycium marashicum membranaceum munzurense orbiculatum papillosum saxatile schistosum speciosum spicatum spinosum stenopterum stylosum syriacum thesiifolium transhyrcanum turcica umbellatum

;

END;

BEGIN CHARACTERS;

TITLE Character_Matrix;

DIMENSIONS NCHAR=4;

FORMAT DATATYPE = STANDARD RESPECTCASE GAP = - MISSING = ? SYMBOLS = " 0 1 2";

CHARSTATELABELS

1 leaf,

2 locus,

3 seed,

4 spine ;

MATRIX

acarii 1110

alanyae 0101

arabicum 1110

armenum 0100

capitatum 0100

carneum 1210

cordatum 1110

coridifolium 0100

demirizii 0?00

diastrophis 0110

dumanii 0000

elongatum 0010

erinaceum 0101

eunomioides 1210

fimbriatum 1110

froedinii 1000

glaucinum 0?10

grandiflorum 0100

heterocarpum 1??0

huber_morathii 0??0

subalpinae 1000

shirkuhense 1000

karamanicum 0100

lepidioides 0011

lycium 1000

marashicum 01?0

membranaceum 0100

munzurense 1110

orbiculatum 10?0

papillosum 1110

saxatile 1220

schistosum 0100

speciosum 1?10

spicatum 1110

spinosum 0101

stenopterum 0000

stylosum 1110

syriacum 1220

thesiifolium 0220

transhyrcanum 0000

turcica 1110

umbellatum 0000

;

END;

BEGIN ASSUMPTIONS;

TYPESET * UNTITLED = unord: 1- 4;

END;

BEGIN MESQUITECHARMODELS;

ProbModelSet * UNTITLED = 'Mk1 (est.)': 1- 4;

END;

BEGIN TREES;

Title 'Trees from "trnL-F_29_5_2022_map_mb1.tre"';

ID 01818634a0331;

LINK Taxa = Taxa;

TRANSLATE

[0] 1 acarii,

[1] 2 alanyae,

[2] 3 arabicum,

[3] 4 armenum,

[4] 5 capitatum,

[5] 6 carneum,

[6] 7 cordatum,

[7] 8 coridifolium,

[8] 9 demirizii,

[9] 10 diastrophis,

[10] 11 dumanii,

[11] 12 elongatum,

[12] 13 erinaceum,

[13] 14 eunomioides,

[14] 15 fimbriatum,

[15] 16 froedinii,

[16] 17 glaucinum,

[17] 18 grandiflorum,

[18] 19 heterocarpum,

[19] 20 huber_morathii,

[20] 21 subalpinae,

[21] 22 shirkuhense,

[22] 23 karamanicum,

[23] 24 lepidioides,

[24] 25 lycium,

[25] 26 marashicum,

[26] 27 membranaceum,

[27] 28 munzurense,

[28] 29 orbiculatum,

[29] 30 papillosum,

[30] 31 saxatile,

[31] 32 schistosum,

[32] 33 speciosum,

[33] 34 spicatum,

[34] 35 spinosum,

[35] 36 stenopterum,

[36] 37 stylosum,

[37] 38 syriacum,

[38] 39 thesiifolium,

[39] 40 transhyrcanum,

[40] 41 turcica,

[41] 42 umbellatum;

TREE con_50_majrule = (13:0.009806,34:0.008965,5:0.004052,((((((42:0.003525,(((1:0.01215,31:0.00221):0.005455,((28:0.005499,30:0.003535,33:0.005474):0.005511,15:0.011559,37:0.007547):0.003511):0.003578,29:0.001562):0.003508,(21:0.009627,22:0.004457):0.004373):0.007368,(((3:0.001523,6:0.001491,(16:0.001481,38:0.001483):0.003484,19:0.005495):0.005555,36:0.005436):0.005722,11:0.009796):0.003477,39:0.003491,7:0.009399,25:0.003492,41:0.007436):0.015875,(24:0.037798,35:0.008052):0.018455):0.007721,14:0.005578):0.003573,(((12:0.001897,9:0.001476,17:0.001445):0.007971,2:0.001477,32:0.005353,((4:0.003464,8:0.001471):0.011388,10:0.001527):0.003447):0.003602,23:0.001702):0.008111):0.008069,(27:0.003469,40:0.001543,26:0.016719):0.005429):0.011652,(18:0.00547,20:0.003557):0.003165);

END;
