# Supplementary material for: Underestimated diversity in high elevations of a global biodiversity hotspot: two new endemic species of Aethionema (Brassicaceae) from the alpine zone of Iran
Source: Front Plant Sci. 2023 May 26;14:1182073. doi: 10.3389/fpls.2023.1182073 (PMC10250747; doi:10.3389/fpls.2023.1182073)
Supplement: Supplementary file 2 [file DataSheet_2.zip › Date Sheet 2/Character-mapping/posterior_trees_for_mapping.docx]

#NEXUS

[ID: 4264414179]

[Param: tree]

begin trees;

translate

1 erinaceum,

2 umbellatum,

3 elongatum,

4 membranaceum,

5 transhyrcanum,

6 grandiflorum,

7 acarii,

8 alanyae,

9 karamanicum,

10 schistosum,

11 armenum,

12 coridifolium,

13 diastrophis,

14 demirizii,

15 glaucinum,

16 huber_morathii,

17 spicatum,

18 eunomioides,

19 capitatum,

20 arabicum,

21 carneum,

22 thesiifolium,

23 orbiculatum,

24 froedinii,

25 syriacum,

26 heterocarpum,

27 cordatum,

28 munzurense,

29 papillosum,

30 lycium,

31 turcica,

32 fimbriatum,

33 speciosum,

34 stylosum,

35 dumanii,

36 stenopterum,

37 saxatile,

38 J1,

39 J2,

40 marashicum,

41 lepidioides,

42 spinosum;

tree gen.0 = [&U] (30:2.000000e-002,((20:2.000000e-002,16:2.000000e-002):2.000000e-002,((37:2.000000e-002,(((33:2.000000e-002,((36:2.000000e-002,34:2.000000e-002):2.000000e-002,22:2.000000e-002):2.000000e-002):2.000000e-002,11:2.000000e-002):2.000000e-002,(6:2.000000e-002,(17:2.000000e-002,(9:2.000000e-002,((31:2.000000e-002,(19:2.000000e-002,((24:2.000000e-002,(32:2.000000e-002,10:2.000000e-002):2.000000e-002):2.000000e-002,8:2.000000e-002):2.000000e-002):2.000000e-002):2.000000e-002,((42:2.000000e-002,18:2.000000e-002):2.000000e-002,((14:2.000000e-002,(27:2.000000e-002,7:2.000000e-002):2.000000e-002):2.000000e-002,((38:2.000000e-002,15:2.000000e-002):2.000000e-002,(26:2.000000e-002,4:2.000000e-002):2.000000e-002):2.000000e-002):2.000000e-002):2.000000e-002):2.000000e-002):2.000000e-002):2.000000e-002):2.000000e-002):2.000000e-002):2.000000e-002,(((28:2.000000e-002,5:2.000000e-002):2.000000e-002,(39:2.000000e-002,2:2.000000e-002):2.000000e-002):2.000000e-002,(23:2.000000e-002,((35:2.000000e-002,(40:2.000000e-002,12:2.000000e-002):2.000000e-002):2.000000e-002,(41:2.000000e-002,(29:2.000000e-002,(21:2.000000e-002,((25:2.000000e-002,13:2.000000e-002):2.000000e-002,3:2.000000e-002):2.000000e-002):2.000000e-002):2.000000e-002):2.000000e-002):2.000000e-002):2.000000e-002):2.000000e-002):2.000000e-002):2.000000e-002,1:2.000000e-002);

tree gen.1000 = [&U] ((((((4:3.026666e-003,5:1.269163e-003):1.243170e-003,3:9.482921e-003):2.118929e-003,((((36:5.338444e-003,((((24:9.409453e-003,(39:1.014081e-002,(((27:4.746687e-003,((21:1.173272e-002,26:4.375645e-003):1.365333e-003,(20:3.839812e-003,25:1.694973e-004):1.038897e-003):1.041151e-002):1.643835e-003,31:6.815616e-003):5.654522e-003,(22:7.949759e-003,30:1.097563e-003):8.876478e-004):2.935718e-003):8.234214e-004):8.891738e-003,35:5.892755e-003):3.765461e-003,34:1.024484e-002):8.711624e-003,(((32:5.732172e-003,((28:4.328004e-003,29:3.408717e-003):1.079756e-004,33:3.142636e-003):2.615848e-003):3.923145e-003,23:8.559236e-004):1.189147e-003,(7:1.196186e-002,37:6.381588e-003):4.505146e-004):1.082831e-003):7.665544e-003):1.323278e-003,(38:5.027813e-003,2:2.286085e-003):1.121185e-003):2.132457e-003,(41:7.904136e-003,42:6.231089e-003):7.849887e-003):2.216322e-003,40:1.077359e-002):2.722136e-003):3.304813e-003,((12:1.725068e-003,11:5.162284e-003):7.317286e-003,((9:5.248074e-003,(((18:6.953963e-003,10:6.606636e-003):4.680439e-003,13:1.278759e-003):2.985584e-004,(15:2.689290e-004,14:1.284492e-003):8.519507e-003):1.580080e-002):8.793327e-004,8:7.739804e-003):1.993284e-004):1.389676e-002):7.039651e-003,((19:3.166650e-003,16:3.482770e-003):1.320198e-003,6:1.553809e-003):2.133498e-003):8.146406e-003,17:9.203345e-003,1:1.352007e-002);

tree gen.2000 = [&U] (((5:2.636972e-003,4:1.036187e-003):2.660861e-003,(((42:7.490171e-003,41:2.894715e-002):6.115598e-003,(40:1.125872e-002,(((36:1.255001e-003,(2:3.369071e-003,((37:4.182237e-003,((32:1.542419e-002,(34:7.555987e-003,(28:9.190050e-003,(29:1.648238e-003,33:2.830733e-003):1.428953e-003):4.922560e-003):3.012908e-003):1.230620e-003,7:9.997378e-003):1.105439e-003):1.814585e-003,23:1.744646e-003):3.700473e-003):1.229768e-003):3.958705e-003,(39:6.012672e-003,38:3.185448e-003):1.088746e-003):1.758401e-003,((((20:2.900100e-003,21:2.236943e-003):1.211295e-003,26:6.159531e-003):1.315658e-003,(24:4.140834e-003,25:1.733483e-003):3.258015e-003):1.712510e-003,(((31:2.023140e-003,30:8.599283e-004):4.324221e-004,35:1.181265e-002):2.977027e-004,(22:2.727001e-003,27:5.456499e-003):5.404507e-004):4.378911e-003):9.750792e-004):6.778960e-004):4.297193e-003):1.722250e-003,(18:1.026441e-003,((((((14:7.416076e-004,15:5.975332e-003):1.454228e-003,3:6.187029e-003):4.607363e-003,10:2.207393e-003):1.488163e-003,13:1.502625e-003):2.171289e-003,(11:5.245949e-003,12:9.787758e-004):3.593209e-003):3.671698e-003,(9:5.792420e-003,8:2.279886e-003):6.091035e-004):3.639676e-003):1.201046e-003):4.076946e-003):3.686001e-003,((17:4.090599e-003,16:3.343989e-003):9.134208e-004,(6:1.809082e-003,19:4.006072e-003):1.128542e-003):4.167169e-003,1:3.551465e-003);

tree gen.3000 = [&U] ((((9:6.588030e-005,(8:2.306589e-003,(10:3.712706e-004,(((13:3.176969e-004,(12:4.860711e-003,11:4.843091e-003):4.796601e-003):5.763209e-003,(14:1.946828e-004,15:1.334438e-003):2.257649e-003):1.403867e-003,3:1.682759e-003):1.169557e-002):4.586937e-003):2.207469e-003):6.062906e-003,(18:1.987351e-003,((41:1.481193e-002,42:1.067772e-003):6.912832e-003,(22:1.023564e-003,(((((((21:2.633923e-004,(20:8.745883e-004,(24:1.189797e-003,25:7.611919e-004):4.263561e-003):6.485503e-004):2.316643e-004,26:4.334335e-003):1.295719e-003,36:2.038819e-003):2.567329e-003,35:5.609647e-003):1.540882e-003,31:5.225972e-003):1.001686e-003,(30:9.427146e-004,((((37:6.295499e-004,7:1.271902e-002):4.903025e-003,(32:5.183942e-003,((33:3.493102e-003,(29:7.202716e-003,28:1.026580e-002):7.403935e-004):2.210526e-003,34:1.915340e-003):1.549413e-003):4.714227e-003):2.382595e-003,23:2.471499e-005):1.052958e-003,(2:1.841740e-004,(38:2.948634e-003,39:3.928601e-003):1.337612e-003):3.568036e-003):4.873701e-003):3.725008e-004):1.220104e-004,27:5.478199e-003):5.366008e-003):9.650383e-003):4.402772e-003):2.636765e-003):4.764190e-003,((40:7.786428e-003,4:2.330076e-003):6.938243e-004,5:2.344504e-003):1.641461e-003):4.928239e-003,(17:7.669854e-003,((16:2.679852e-003,19:7.947014e-003):3.172751e-003,6:3.467054e-003):1.242676e-003):3.159541e-003,1:7.004300e-003);

tree gen.4000 = [&U] (((((41:3.302015e-002,42:4.340163e-003):7.760688e-003,(30:1.159141e-003,(((22:8.586520e-004,31:1.754108e-003):3.516303e-004,(35:7.961649e-003,((36:1.223471e-003,(21:2.330144e-004,((20:2.213381e-003,26:2.379142e-003):9.130692e-004,(25:2.406030e-004,24:6.796035e-003):5.739948e-003):1.610231e-003):3.654390e-003):2.596904e-003,((23:1.100987e-003,((7:6.789303e-003,37:4.788488e-003):1.941029e-003,((28:8.346826e-003,(33:2.649478e-003,29:6.996292e-003):1.689583e-004):1.431060e-003,(32:8.260583e-003,34:2.780589e-003):5.050269e-004):3.405663e-003):4.334034e-003):2.868541e-003,((39:5.555490e-003,38:8.901344e-003):2.776271e-003,2:1.012488e-003):3.350184e-003):1.354470e-003):3.798363e-003):5.909190e-004):2.436676e-003,27:9.447964e-003):2.221615e-003):1.521445e-002):4.266606e-003,(((8:2.292079e-003,((15:6.337313e-004,(14:3.261813e-004,3:8.109819e-003):4.691450e-003):2.262859e-003,(9:4.562537e-004,(13:2.687946e-003,(12:2.238741e-004,11:1.885956e-003):1.793295e-003):1.140808e-002):1.309117e-003):9.831732e-003):2.009814e-003,10:4.498813e-004):8.562088e-003,18:2.189021e-003):3.156788e-003):6.055748e-003,(40:7.207332e-003,(5:2.636912e-003,4:1.102753e-003):4.784475e-004):2.612485e-003):3.658918e-003,(((16:1.795849e-003,6:4.959555e-004):2.202132e-003,17:3.482937e-003):9.087239e-005,19:1.347365e-003):2.474656e-004,1:6.505998e-003);

tree gen.5000 = [&U] (((6:2.327042e-003,16:2.440517e-003):1.616603e-003,19:3.586665e-003):3.001798e-004,((((((8:9.846816e-004,(13:3.276097e-003,(12:2.230883e-004,11:2.665450e-003):6.845710e-003):1.638873e-003):8.781717e-004,(3:4.761813e-003,((14:1.671205e-003,15:1.101970e-003):2.090647e-003,9:1.644612e-003):2.608433e-003):9.858055e-003):1.289592e-003,10:5.845631e-004):4.500175e-003,(((41:3.206309e-002,42:7.395189e-003):1.012450e-002,(30:1.042447e-003,(((((2:3.338224e-003,(39:3.821360e-003,38:6.702159e-003):5.446271e-003):1.630759e-004,(23:2.189995e-003,((7:6.765471e-003,37:3.785485e-003):4.018922e-003,(34:4.119547e-003,((33:3.551853e-003,(28:2.478375e-003,29:2.959453e-003):2.721301e-004):1.417263e-003,32:6.978180e-003):1.396018e-003):1.795695e-003):4.450325e-003):9.249422e-003):2.728273e-004,(36:4.076255e-004,(((20:4.504351e-004,(25:3.480358e-004,24:1.956022e-003):7.726093e-003):6.320550e-004,26:3.087120e-003):7.552168e-004,21:2.304559e-004):9.364994e-003):2.897962e-003):1.247868e-003,35:1.143304e-002):5.820577e-004,(27:2.852582e-003,(31:8.400322e-003,22:7.863617e-004):2.217909e-003):3.105212e-004):1.850838e-003):5.317791e-003):4.836646e-003,18:6.598618e-003):5.415479e-004):5.116105e-003,((5:1.978688e-003,4:1.461558e-003):2.101261e-003,40:1.019123e-002):1.419873e-003):7.921667e-003,17:1.023239e-002):8.934055e-006,1:4.979777e-003);

tree gen.6000 = [&U] ((19:1.601196e-004,17:8.801114e-003):3.566716e-003,((16:1.881879e-003,6:3.108934e-003):6.394644e-004,((((10:2.902458e-003,(((14:1.572339e-003,(15:1.975267e-003,3:6.297372e-004):1.101325e-003):1.087023e-002,(13:6.313931e-004,(12:1.274533e-003,11:2.769132e-003):2.895455e-003):4.132980e-003):6.010704e-003,8:2.810414e-003):1.616915e-003):6.058812e-004,9:9.913865e-005):2.440106e-003,(18:1.494100e-003,((41:3.017341e-002,42:9.266640e-003):1.302042e-002,(22:2.163482e-004,((((((26:1.835780e-003,(24:2.305507e-003,25:1.150764e-003):2.848454e-003):1.387337e-003,(21:1.452184e-003,20:3.429224e-003):1.186317e-003):7.865819e-003,36:2.911808e-003):3.091859e-003,35:8.441732e-003):3.591161e-003,30:1.715120e-003):1.603114e-003,(((((38:6.191405e-003,39:9.435765e-003):1.516184e-003,(((37:5.053538e-004,7:5.860261e-003):6.227092e-003,((32:7.790731e-003,(33:3.635807e-003,(28:5.337688e-003,29:2.304831e-003):7.807325e-005):2.249375e-003):3.601279e-004,34:8.728752e-003):2.788180e-003):5.063597e-003,23:2.926233e-003):1.113808e-003):4.868460e-004,2:6.886811e-004):7.754218e-003,27:1.127440e-002):3.109021e-004,31:3.780507e-003):2.914345e-004):1.150035e-003):1.855663e-002):1.625093e-003):3.830071e-003):4.573571e-003,((5:1.039988e-003,4:3.014336e-004):1.129563e-004,40:1.180953e-002):1.282281e-002):6.050499e-003):8.444972e-004,1:1.007231e-002);

tree gen.7000 = [&U] ((17:1.631208e-003,((6:7.146314e-003,19:1.251217e-002):3.722834e-003,16:4.006560e-003):1.997860e-003):5.407000e-004,((5:1.808943e-003,(40:9.299481e-003,4:3.090308e-003):2.239220e-003):1.155127e-004,((9:1.426010e-003,((13:1.165375e-004,(11:8.093936e-004,12:3.525349e-004):1.253427e-002):1.388519e-003,(((3:4.451860e-004,(15:2.938836e-003,14:1.400716e-003):1.013509e-003):1.563417e-002,10:6.563939e-003):1.200992e-003,8:9.846548e-004):1.273266e-003):3.083622e-003):1.148948e-002,(18:2.999932e-003,((41:4.965297e-002,42:1.914040e-003):7.959302e-003,(((27:6.134139e-003,(((((2:1.561798e-003,(((((33:6.712965e-003,28:4.914433e-003):3.110063e-003,29:1.098233e-002):1.947021e-003,(34:6.388291e-003,32:9.098294e-003):4.223104e-003):3.425637e-003,(7:7.426292e-003,37:9.441256e-004):6.292460e-003):4.267479e-003,23:5.154167e-004):3.612838e-003):2.603693e-004,(39:4.474591e-003,38:3.459058e-003):6.757973e-003):7.031524e-003,22:1.560035e-003):1.221207e-004,30:2.038020e-003):1.191801e-003,35:6.739817e-003):4.297247e-003):2.446528e-003,31:5.778588e-003):2.488363e-004,(36:5.137841e-003,((26:1.547698e-003,(20:2.486503e-003,21:1.365534e-003):2.836763e-004):1.514060e-003,(24:5.636760e-003,25:1.339228e-004):1.488477e-003):6.117540e-003):4.690052e-003):5.841438e-003):6.555531e-003):3.775757e-003):7.473880e-003):7.505784e-003,1:1.161061e-002);

tree gen.8000 = [&U] ((17:2.163960e-003,19:3.762746e-003):3.154293e-003,((40:7.301128e-003,((5:2.663321e-003,(((((((15:6.526003e-004,3:3.472496e-004):4.448400e-003,14:7.531282e-004):2.455122e-003,(13:1.150155e-004,(11:3.825409e-004,12:3.182488e-003):8.209322e-003):1.396194e-003):3.127913e-003,(10:3.178659e-003,8:8.109966e-004):2.073169e-003):3.031901e-003,9:5.019869e-003):6.480350e-003,18:3.090764e-003):8.330592e-004,((41:3.989483e-002,42:3.760637e-003):2.022665e-002,(((((((34:9.987077e-003,32:1.163680e-002):5.585108e-004,((29:5.453229e-003,28:6.019324e-003):4.808497e-003,33:1.172663e-003):3.506444e-003):3.430915e-003,(7:2.421530e-002,37:2.279708e-004):3.716252e-003):2.828480e-003,23:2.530261e-004):1.627387e-003,(2:1.582888e-003,(39:6.587298e-003,38:4.321453e-003):7.770267e-004):3.424298e-003):1.170439e-003,(36:2.228251e-003,((24:1.753343e-003,25:3.649289e-004):2.382445e-003,(26:3.205018e-003,(21:1.336315e-003,20:1.240035e-003):2.698117e-003):7.689972e-004):3.885216e-003):6.729438e-003):4.736864e-003,(22:1.096015e-003,(31:5.553627e-003,((35:1.294762e-002,27:2.198191e-003):3.500374e-003,30:3.910937e-003):1.311620e-003):1.157060e-003):7.664168e-004):1.095001e-002):8.854915e-003):7.607469e-003):2.007936e-005,4:7.574450e-004):8.312205e-004):6.698584e-003,(6:4.195664e-003,16:3.192798e-003):5.690231e-003):3.503902e-004,1:1.061298e-002);

tree gen.9000 = [&U] ((6:2.928895e-003,16:4.180235e-004):1.390034e-003,((((18:3.136546e-003,((41:3.545353e-002,42:5.624599e-003):1.349909e-002,((30:2.859722e-003,(31:8.531362e-003,(((35:5.287045e-003,(36:8.336661e-003,((21:4.080959e-004,(26:1.662227e-003,(24:9.672956e-004,25:1.781444e-004):6.240219e-003):1.688447e-004):2.563658e-003,20:2.220579e-003):2.103616e-003):1.768124e-003):6.456408e-003,27:1.140546e-002):1.995404e-003,(2:1.167287e-003,((((32:1.339637e-002,(((29:3.531094e-003,33:8.574652e-004):2.925967e-003,28:3.653721e-003):1.920054e-003,34:8.135853e-003):5.989345e-004):1.458422e-003,(7:8.809388e-003,37:1.019977e-004):8.644426e-003):1.590091e-003,23:1.756086e-004):4.908825e-003,(39:1.925920e-003,38:1.104958e-002):7.676200e-004):3.996609e-004):3.764877e-003):2.325723e-006):8.957873e-004):1.019924e-003,22:5.258889e-003):1.362628e-002):5.365035e-003):2.824716e-003,(((8:2.067933e-003,(13:1.045936e-003,(11:9.987709e-005,12:3.650508e-003):6.962279e-003):2.162731e-003):2.069812e-003,((14:2.026417e-003,(3:2.087270e-003,15:2.459867e-003):4.041379e-003):7.790206e-003,10:6.599494e-003):1.357714e-003):3.380891e-003,9:4.642097e-003):9.876110e-003):5.594510e-003,(5:3.083753e-004,(4:2.664536e-003,40:5.219089e-003):4.565575e-003):2.985922e-003):7.035095e-003,(19:1.400526e-003,17:7.348861e-003):2.182388e-003):2.313401e-003,1:6.643250e-003);

tree gen.10000 = [&U] (((17:5.998285e-003,19:2.428604e-003):1.286542e-003,6:5.829443e-003):3.336356e-004,((((18:1.729619e-003,((41:2.734006e-002,42:9.972674e-003):1.830857e-002,((((27:6.139401e-003,30:4.197582e-003):1.707046e-003,(((((34:9.499787e-003,32:3.153353e-003):2.386929e-003,((28:6.685786e-003,33:1.406604e-003):2.777411e-004,29:2.477024e-003):1.619834e-003):2.449415e-003,(7:7.909460e-003,37:8.166357e-004):5.085300e-003):2.079482e-003,23:1.271169e-003):2.412963e-003,((39:2.434875e-003,38:3.196686e-003):7.995490e-004,2:9.710859e-004):1.889717e-003):3.926040e-003):1.870465e-003,(31:2.490168e-003,(35:1.328959e-002,(36:5.264514e-003,(((24:2.333705e-004,25:1.875300e-003):8.654753e-004,21:1.404762e-003):4.118926e-004,(20:1.854091e-003,26:1.917901e-003):2.496348e-003):4.364613e-003):3.151989e-003):6.966738e-003):2.187127e-003):4.050645e-004,22:2.222961e-003):1.704677e-002):1.215544e-002):4.882307e-003,(((((13:4.057578e-004,(11:9.728977e-005,12:2.380969e-003):1.061628e-002):4.458652e-003,(3:1.912423e-004,(15:9.141183e-004,14:2.460303e-003):2.099136e-003):2.535148e-003):3.899980e-003,10:4.879518e-003):3.881714e-003,8:3.072943e-004):2.859777e-003,9:5.072338e-004):2.288738e-003):7.343358e-003,((4:2.441105e-003,40:8.526035e-003):1.270962e-004,5:1.890463e-003):4.133958e-003):8.959816e-003,16:1.779587e-003):2.129485e-004,1:8.746354e-003);

tree gen.11000 = [&U] ((6:5.679510e-003,16:2.747675e-003):1.623665e-003,((((18:2.254486e-003,((41:1.993046e-002,42:7.202345e-003):9.094400e-003,((35:6.074047e-003,(36:6.264449e-003,((21:5.118543e-004,20:1.724450e-003):1.297626e-003,((24:4.888829e-004,25:1.029826e-003):7.985167e-004,26:4.282615e-003):5.228899e-003):2.477055e-003):6.362714e-003):2.058178e-003,(((((((32:5.542503e-003,34:7.429148e-003):4.893953e-003,((29:3.981732e-003,28:2.040899e-003):1.376984e-003,33:3.236606e-003):6.056891e-003):3.541056e-003,(7:1.124900e-002,37:5.632569e-004):5.773195e-003):1.131598e-003,23:1.739050e-003):1.216126e-003,(39:1.685791e-003,38:2.255786e-003):8.905839e-004):4.777855e-004,2:1.395416e-003):2.311978e-003,(31:6.505064e-003,((22:6.031398e-003,27:7.387035e-003):2.939439e-003,30:2.985698e-003):2.019626e-004):1.969490e-005):8.330743e-004):1.023010e-002):5.538063e-003):3.998706e-003,(9:2.055749e-003,((14:3.280825e-003,(3:2.995417e-003,15:3.690150e-004):7.870099e-003):8.850838e-003,(((13:5.805280e-004,(11:1.370241e-003,12:1.498966e-003):9.863668e-003):1.592803e-003,10:6.408709e-003):3.250203e-003,8:1.307410e-003):9.448291e-004):3.392756e-003):2.020388e-003):6.753658e-003,((4:3.670964e-004,5:1.179847e-003):2.012214e-003,40:7.329844e-003):1.264876e-003):1.460899e-002,(19:1.160252e-003,17:5.661858e-003):1.023699e-003):8.094462e-004,1:1.183411e-002);

tree gen.12000 = [&U] (((19:4.716911e-003,(16:5.325883e-003,6:3.421154e-003):4.527263e-005):2.049609e-003,17:6.834749e-003):4.676423e-003,(((18:4.317932e-003,((41:2.589800e-002,42:1.434909e-003):1.266758e-002,(((((35:6.261123e-003,(36:4.653990e-003,((20:6.415221e-005,(24:1.455711e-004,25:7.070009e-005):1.164124e-003):2.556505e-003,(26:1.586495e-003,21:1.094572e-003):3.336291e-003):2.654616e-003):3.272021e-003):1.046839e-003,30:3.771822e-003):7.187954e-004,(27:3.831298e-003,31:8.234208e-003):5.630667e-004):1.411456e-004,22:1.042340e-003):2.916596e-004,((2:6.107777e-004,(39:7.257338e-004,38:2.683671e-003):9.776548e-004):1.369804e-004,(((32:1.404750e-002,(34:6.805324e-003,(33:5.601913e-003,(28:1.367365e-003,29:4.062842e-003):1.330392e-004):1.344626e-003):5.179638e-004):7.589443e-004,(7:2.750825e-003,37:2.194297e-003):5.448977e-003):2.027393e-003,23:8.386623e-004):3.980195e-003):1.509956e-003):1.612295e-002):9.836226e-004):6.243602e-003,(9:3.124370e-003,((14:1.981543e-003,(3:2.385678e-003,15:1.081894e-004):1.319398e-003):4.713921e-003,((10:2.282456e-003,8:6.302054e-005):2.620398e-003,(13:3.044621e-003,(11:1.198600e-003,12:5.853685e-004):3.414884e-003):6.213344e-004):1.197339e-003):4.942773e-003):1.980071e-003):3.869606e-003,((4:1.416810e-003,5:1.901401e-003):1.252959e-003,40:4.213614e-003):4.167359e-003):1.215581e-002,1:4.785354e-003);

tree gen.13000 = [&U] ((17:4.065374e-003,((6:5.715284e-003,16:1.786933e-004):4.830310e-003,(((9:4.540209e-004,(((((3:3.951731e-004,14:7.077450e-005):8.776759e-004,15:1.249267e-004):5.991702e-003,(13:3.141704e-003,(11:5.712808e-003,12:2.606106e-003):1.266940e-002):1.362623e-003):4.597164e-004,10:1.202719e-002):8.016384e-003,8:6.793744e-004):8.077946e-003):4.019632e-003,(18:3.651517e-003,(((((30:2.127730e-003,31:6.147433e-003):1.317701e-004,27:1.290371e-002):1.311033e-003,(((39:2.023553e-003,38:8.058035e-003):5.011697e-003,((23:4.218931e-004,(((32:7.145721e-003,(28:1.013258e-002,(33:3.582132e-004,29:1.131592e-002):6.195281e-004):7.742646e-003):1.975777e-003,34:1.313401e-002):2.194674e-003,(37:3.345903e-004,7:9.897241e-003):6.790644e-003):9.829147e-004):2.801637e-003,2:2.359098e-003):2.510993e-004):1.337939e-002,(35:5.852923e-003,(36:4.411362e-003,((((24:4.284378e-004,25:3.118408e-004):1.483994e-003,26:7.423549e-003):1.838572e-003,20:6.013435e-004):2.719362e-004,21:3.721836e-003):8.763697e-003):3.836688e-003):9.157908e-003):1.750763e-003):3.585121e-004,22:2.032019e-003):2.955622e-002,(42:9.199758e-003,41:2.867509e-002):1.152696e-002):4.117338e-003):1.673392e-003):2.034464e-002,((40:1.722746e-002,5:1.189234e-003):5.948700e-003,4:4.320700e-003):3.904321e-003):3.310052e-003):1.581096e-004):1.925547e-003,19:4.726313e-003,1:8.608014e-003);

tree gen.14000 = [&U] (((((18:3.902758e-003,((41:2.848820e-002,42:2.592840e-003):1.788448e-002,((((27:5.093846e-003,30:7.378126e-004):1.055657e-003,((36:5.673874e-003,((21:2.899676e-003,(20:1.005364e-003,26:4.526794e-003):1.867480e-004):6.076281e-004,(24:1.266621e-004,25:1.630634e-004):3.422660e-003):1.445803e-003):5.739930e-003,35:1.121165e-002):3.778373e-003):5.299827e-004,((((((32:5.115520e-003,((29:1.909640e-003,33:2.535489e-003):4.446420e-004,28:9.175313e-003):2.423926e-003):1.747184e-003,34:5.621533e-003):3.377212e-003,(7:7.333849e-003,37:2.937130e-003):4.714178e-003):8.410974e-004,23:1.163267e-003):6.376230e-004,((39:3.056694e-003,38:1.143080e-002):1.189854e-003,2:6.223896e-003):2.635895e-003):1.385981e-003,22:1.287420e-003):5.419929e-003):1.678139e-003,31:4.891743e-003):1.584061e-002):3.268643e-003):3.875191e-004,(9:1.485061e-003,((((15:6.615022e-004,3:9.548853e-004):3.364821e-003,14:3.140003e-003):8.488328e-003,(13:5.978604e-003,(11:1.188502e-003,12:2.331489e-003):7.127989e-003):8.251753e-003):1.136178e-004,(8:1.726516e-003,10:4.620594e-003):8.821011e-004):5.098091e-003):4.134610e-003):1.471696e-002,((5:5.294533e-003,40:1.347912e-002):1.214246e-003,4:2.436266e-003):4.773626e-003):4.674737e-003,17:7.482659e-003):9.421335e-004,((16:3.467978e-004,6:8.229372e-003):2.629398e-003,19:1.037814e-003):1.888890e-003,1:3.369753e-003);

tree gen.15000 = [&U] ((19:2.063854e-003,17:3.382168e-003):4.510451e-003,((((18:4.996621e-003,((41:2.157973e-002,42:1.133545e-002):1.901873e-002,(((22:2.716888e-003,27:5.907424e-003):1.160280e-003,((((36:8.193511e-003,(21:1.393237e-003,(20:2.567351e-003,((24:1.160461e-004,25:1.368939e-003):2.445750e-003,26:4.721053e-003):1.346346e-003):1.071135e-004):3.007664e-003):8.238877e-003,35:6.397226e-003):1.997067e-003,(((((((28:4.289470e-003,33:6.126173e-003):3.162190e-004,29:1.577453e-003):1.423747e-003,(34:4.735254e-003,32:1.626691e-002):1.300035e-004):1.484296e-003,(7:7.434102e-003,37:1.565491e-003):7.484439e-003):1.852603e-003,23:5.321381e-005):4.333720e-003,(39:3.047889e-003,38:1.009097e-002):9.878216e-004):1.760361e-003,2:6.428398e-003):7.767790e-003):8.811843e-004,31:4.502110e-003):9.621081e-004):2.014636e-003,30:1.072217e-003):1.676648e-002):3.500533e-003):3.662217e-003,(9:1.979630e-003,(((10:4.583916e-003,8:5.250743e-004):7.879755e-004,(((14:7.785425e-004,15:3.112974e-005):5.050746e-004,3:3.159782e-004):8.464189e-003,(11:2.016071e-003,12:1.627646e-003):8.276680e-003):3.454466e-003):1.068683e-003,13:7.492830e-003):2.969337e-003):5.202947e-003):2.979490e-003,((5:3.280517e-004,4:2.055110e-003):2.451341e-004,40:9.924501e-003):9.690602e-003):1.865110e-002,(16:2.539079e-003,6:5.321885e-003):3.524475e-003):4.317809e-005,1:3.811023e-003);

tree gen.16000 = [&U] (((19:3.568230e-003,17:5.067928e-003):7.182114e-004,(16:5.505077e-003,6:8.173461e-003):5.845049e-003):7.653886e-006,(((18:5.236313e-003,((41:4.280076e-002,42:9.184346e-003):1.067437e-002,(22:6.823927e-003,(((27:9.058153e-003,((39:8.438305e-003,38:1.021718e-002):7.420006e-003,(((((33:1.009937e-002,(29:2.305892e-003,28:2.254754e-003):3.033483e-003):4.662049e-003,(34:7.225504e-003,32:1.440386e-002):1.203855e-003):3.010688e-003,(7:1.423589e-002,37:2.844672e-003):8.144807e-003):3.217903e-003,23:1.003066e-004):4.353697e-003,2:3.712791e-003):1.945009e-003):9.604326e-003):1.236344e-004,((36:4.679785e-003,((24:1.263543e-004,25:4.139479e-003):2.593334e-003,((26:3.621218e-003,20:4.912429e-003):1.497516e-003,21:1.639387e-003):1.663197e-003):1.273806e-003):2.626543e-003,35:1.696358e-002):7.043920e-003):2.138940e-003,(31:6.733084e-003,30:2.525183e-003):7.174703e-004):3.121852e-003):2.618536e-002):3.255294e-003):3.293941e-004,(9:2.229116e-003,(8:8.354077e-004,((10:1.107566e-002,(3:1.387263e-004,(14:6.832287e-004,15:4.367714e-004):1.779323e-003):2.354642e-003):6.852710e-003,(13:4.884035e-003,(11:7.283407e-003,12:6.439352e-003):1.722711e-002):7.663099e-003):4.514703e-003):4.132689e-003):5.085419e-003):1.139690e-002,(4:2.565757e-003,(40:1.474056e-002,5:4.731601e-005):5.779488e-004):6.389193e-003):8.960617e-003,1:2.347992e-002);

tree gen.17000 = [&U] ((17:9.933665e-003,(16:9.461887e-003,(19:1.964301e-003,6:1.200517e-002):1.383015e-003):2.928490e-006):1.635576e-003,(((18:4.718851e-003,((41:4.409397e-002,42:5.925165e-003):2.318215e-002,(31:1.504781e-002,((2:4.228740e-003,((((((28:5.460134e-003,(29:4.944324e-004,33:8.522637e-003):3.314848e-003):1.109856e-002,34:7.864028e-003):1.422461e-004,32:1.267849e-002):1.003569e-003,(7:1.086471e-002,37:6.512275e-003):6.019597e-003):1.646516e-003,23:1.007580e-003):4.617283e-003,(39:3.245311e-003,38:1.139781e-002):2.169639e-003):1.244599e-003):6.160380e-003,((30:2.278842e-003,(27:1.463385e-002,22:4.414078e-003):2.845228e-003):1.153713e-003,((36:6.041908e-003,((20:1.781502e-003,(21:1.291583e-003,(24:7.073050e-004,25:2.637402e-003):4.864851e-003):2.783562e-004):3.263346e-003,26:1.923012e-003):1.152869e-003):6.353371e-003,35:1.102232e-002):3.309247e-003):4.635505e-003):8.723237e-004):9.650525e-003):9.381912e-003):1.491019e-003,(9:3.324468e-003,((10:7.566880e-003,(((13:2.768718e-003,(11:6.653745e-003,12:5.016188e-004):1.254136e-002):3.870232e-003,(14:1.144676e-004,15:1.482561e-004):8.174390e-003):4.212394e-003,3:1.986667e-003):1.444344e-002):2.649907e-003,8:1.501504e-003):3.805275e-003):5.073049e-003):8.188673e-003,(4:6.906902e-003,(5:7.319932e-004,40:8.537184e-003):3.429169e-005):5.898243e-003):1.151757e-002,1:6.543180e-003);

tree gen.18000 = [&U] (((19:9.357528e-003,((18:4.446674e-003,(((41:3.566952e-002,42:6.123308e-003):2.123967e-002,((((30:6.025995e-003,(31:7.031322e-003,27:2.599719e-003):1.225623e-002):3.957954e-004,((36:4.457038e-003,((21:1.006258e-003,20:4.065623e-003):2.704134e-003,((24:2.524925e-003,25:4.317868e-004):3.530055e-003,26:4.362632e-003):5.289494e-006):3.449536e-003):6.422729e-003,35:1.957281e-003):7.454459e-003):3.765280e-003,22:1.082691e-002):9.246138e-004,(((((34:5.777878e-003,(((28:5.156187e-003,33:6.685841e-003):2.125872e-004,29:2.409607e-003):1.612615e-003,32:1.165005e-002):5.569991e-003):4.336611e-003,(7:6.965899e-003,37:7.772762e-003):1.068491e-002):2.710396e-003,23:4.839282e-004):2.504986e-003,2:6.899150e-003):1.821654e-003,(39:3.953589e-003,38:6.995155e-003):6.024047e-003):4.877808e-003):8.659102e-003):1.412008e-002,(9:1.006297e-003,(10:1.370976e-003,((13:2.577174e-003,(11:5.116409e-003,12:1.676634e-003):9.334030e-003):2.760737e-003,((3:1.891169e-003,(14:1.278253e-003,15:1.741894e-003):4.076429e-004):6.799994e-003,8:1.265292e-003):4.774059e-004):3.286278e-003):1.557711e-003):6.515420e-003):3.002227e-004):9.145911e-003,(4:6.869134e-003,(40:1.160428e-002,5:2.288451e-004):4.635392e-004):8.620818e-003):1.245883e-002):3.186925e-003,17:9.826289e-003):3.426644e-003,(16:9.836570e-004,6:1.967130e-003):6.455244e-003,1:1.447349e-002);

tree gen.19000 = [&U] (((((((10:5.221836e-003,((3:5.914390e-003,(15:1.759633e-003,14:8.404244e-004):3.650061e-004):9.448504e-003,8:1.318001e-003):8.407291e-004):1.317796e-003,(13:1.650278e-003,(11:3.899573e-003,12:4.816087e-003):6.720500e-003):4.141621e-003):5.876530e-003,9:9.833994e-004):8.899422e-003,(18:1.339924e-003,((41:3.146072e-002,42:5.187376e-003):2.318969e-002,(31:1.126854e-002,(30:5.301863e-003,((((36:2.805657e-003,((24:1.789986e-004,25:1.504493e-004):8.238884e-004,((20:4.073801e-004,26:4.638340e-003):6.012185e-004,21:3.863242e-003):2.072746e-004):9.516927e-003):8.229414e-003,35:1.197406e-002):4.419145e-003,(((39:4.066600e-003,38:4.480982e-003):4.420212e-003,2:1.890933e-003):3.324233e-003,(((((33:1.159230e-002,(28:5.498118e-003,29:2.736545e-003):8.745219e-003):2.151631e-003,34:2.360759e-003):3.718821e-003,32:8.031279e-003):3.755241e-003,(7:3.058900e-003,37:4.908960e-003):3.823896e-003):3.604273e-003,23:1.278126e-005):2.597334e-003):6.926529e-003):1.831941e-003,(27:1.272131e-002,22:2.248393e-003):5.276989e-004):3.769922e-003):4.536987e-004):1.463528e-002):8.190031e-003):1.038073e-003):7.023511e-003,(4:1.604556e-003,(5:8.206638e-004,40:1.171667e-002):3.215678e-003):3.652446e-003):1.110870e-002,(6:1.125609e-002,16:4.058485e-003):4.837007e-003):2.754648e-003,(17:6.683219e-003,19:3.654266e-003):2.636843e-004,1:9.349563e-003);

tree gen.20000 = [&U] (((17:3.441916e-003,19:8.252321e-003):4.975606e-003,((((8:8.645899e-004,(10:5.187704e-003,(((14:9.831952e-005,3:2.338480e-003):2.250311e-003,15:1.729531e-003):4.258368e-003,(13:1.587254e-003,(11:2.718890e-003,12:4.206536e-003):1.348246e-002):3.678851e-003):8.569056e-003):4.633996e-004):7.050107e-004,9:9.662347e-004):9.905476e-003,(18:6.830090e-003,((41:3.600077e-002,42:1.341642e-002):6.675348e-003,((31:8.695493e-003,(((30:4.420898e-003,((2:3.786798e-003,((((7:1.545412e-002,37:2.677820e-003):1.013629e-003,32:1.067115e-002):8.289858e-004,(34:4.206173e-003,((29:2.539520e-003,28:8.778985e-003):6.730655e-004,33:5.415778e-003):4.910730e-003):7.444857e-003):3.417723e-003,23:5.025207e-005):4.383013e-003):4.115219e-003,(39:1.139337e-002,38:1.008914e-002):2.991244e-004):1.381565e-002):2.909241e-003,22:6.507633e-004):2.664536e-003,27:1.043245e-002):2.220211e-004):1.011776e-002,((36:4.699882e-003,((24:1.443266e-004,25:3.215397e-004):1.987095e-003,((21:1.943254e-003,20:2.226530e-004):3.863248e-005,26:3.456297e-003):2.732907e-003):4.424767e-003):9.567268e-003,35:8.676150e-003):1.779319e-003):2.028840e-002):3.431798e-003):5.963792e-003):4.011635e-003,(4:4.432871e-003,(40:1.164008e-002,5:1.862852e-003):5.311037e-004):2.596481e-003):8.748240e-003):1.488980e-004,(6:4.434539e-003,16:2.625750e-003):6.734025e-003,1:9.971710e-003);

tree gen.21000 = [&U] ((((((((3:8.008000e-004,(14:1.548009e-003,15:2.302213e-004):1.523405e-003):6.030646e-003,(11:3.539999e-003,12:5.034511e-003):1.154747e-002):2.192755e-003,8:2.360629e-003):9.298205e-004,(13:1.297926e-003,10:2.698146e-003):1.931477e-004):1.230506e-002,9:7.440349e-004):5.837386e-003,(18:7.475654e-003,((41:2.078106e-002,42:1.407158e-002):2.198966e-002,((((2:5.125671e-003,(39:2.051442e-003,38:6.143279e-003):5.326254e-004):6.920902e-003,(((7:1.278489e-002,37:4.495053e-003):3.722678e-003,((((29:9.151671e-003,28:3.632693e-003):4.595667e-004,33:6.231658e-003):5.636378e-003,32:1.701985e-002):4.886248e-004,34:4.976259e-003):5.197763e-003):1.179645e-003,23:2.414403e-003):3.019143e-003):4.697037e-003,(((36:7.269674e-003,(26:2.249158e-003,((21:2.518185e-003,(24:6.251886e-004,25:1.314250e-003):3.392623e-003):7.732095e-003,20:1.887257e-003):2.314896e-004):7.557889e-003):1.795417e-003,35:5.671628e-003):4.184981e-003,31:4.020830e-003):4.687974e-003):3.973326e-004,(22:5.265120e-004,(30:4.515052e-003,27:3.672979e-003):1.237023e-003):3.496460e-003):2.091404e-002):6.163616e-003):2.955837e-003):4.298097e-003,((40:8.679193e-003,4:2.829695e-003):6.813817e-004,5:3.026916e-003):6.096610e-003):1.031587e-002,(17:8.336174e-003,((6:8.596698e-003,19:3.625696e-003):4.430570e-004,16:6.531225e-003):1.200236e-003):3.800995e-003,1:1.046576e-002);

tree gen.22000 = [&U] ((19:2.488374e-003,(17:6.577186e-003,((((41:5.238685e-002,42:5.641048e-003):9.341354e-003,((36:2.746790e-003,(((24:4.728376e-005,25:5.892886e-004):2.287608e-003,21:3.384719e-004):2.104766e-003,(20:2.144748e-003,26:1.156891e-002):1.834638e-003):4.956890e-003):2.141466e-003,((((31:3.578576e-003,35:1.135025e-002):9.878408e-005,30:3.550592e-003):2.882889e-003,27:2.944530e-003):3.309005e-004,(22:4.484976e-003,(((7:9.232981e-003,37:6.362600e-003):5.767506e-003,((32:7.792449e-003,((33:5.093823e-003,28:3.412169e-003):1.593387e-005,29:2.379037e-003):4.548296e-003):9.395277e-004,34:3.091352e-003):3.520212e-003):1.520111e-003,((2:2.920478e-003,(39:7.351895e-003,38:6.869675e-003):2.869491e-004):4.463022e-003,23:5.196618e-003):2.662468e-003):1.367810e-002):2.267944e-003):3.880124e-003):9.713862e-003):5.051185e-003,(((((3:1.225043e-004,14:1.795534e-003):8.402789e-004,15:2.202024e-003):1.374305e-002,((10:6.163266e-003,(13:5.739891e-004,(11:1.019317e-002,12:3.965372e-004):9.365494e-003):5.751388e-003):3.344667e-004,8:1.594238e-003):5.211009e-004):1.382298e-003,9:1.749945e-003):3.876985e-003,18:6.664738e-003):2.551886e-004):5.971259e-003,((4:2.564893e-003,40:1.219732e-002):2.496552e-004,5:9.372792e-004):3.027075e-003):6.187661e-003):2.182555e-004):9.048442e-004,(6:6.824165e-003,16:5.830538e-004):2.848125e-003,1:9.612153e-003);

tree gen.23000 = [&U] ((((((8:1.163785e-003,(((14:3.684238e-003,3:7.113906e-004):1.963113e-003,15:2.702394e-003):1.441163e-002,(13:9.903368e-004,(11:3.704665e-003,12:3.013282e-003):1.940473e-002):6.589300e-003):6.854549e-005):1.009885e-003,10:8.877265e-003):6.109615e-003,9:1.584955e-003):8.133335e-003,(((41:5.991650e-002,42:1.838743e-002):1.352503e-002,(27:4.335530e-003,(((31:1.659895e-002,((((((7:1.394737e-002,37:4.576575e-003):5.286293e-003,(32:7.125837e-003,(34:5.976384e-003,((29:2.966958e-003,28:3.857050e-003):2.736328e-003,33:4.935269e-003):4.144765e-003):1.974597e-003):1.547586e-003):2.697393e-003,(38:1.438877e-002,39:3.500900e-003):4.333050e-003):3.904954e-003,2:4.055400e-003):1.351359e-004,23:7.661724e-003):3.562746e-003,30:4.387471e-003):8.479176e-005):4.194373e-004,((36:1.579187e-003,((21:5.131852e-004,20:2.594093e-003):3.859407e-005,((25:6.261495e-003,24:1.104324e-004):9.928496e-004,26:9.577549e-003):3.082233e-003):6.553340e-003):3.228820e-003,35:5.738482e-003):3.999110e-004):3.639269e-003,22:5.051588e-004):3.010558e-003):1.261977e-002):1.202016e-002,18:3.278481e-003):1.510408e-003):1.228205e-002,((4:6.473880e-003,40:1.347767e-002):1.114297e-003,5:3.429286e-003):7.613422e-003):1.069140e-002,(17:9.443800e-003,((19:6.641786e-003,6:9.317049e-003):9.239381e-004,16:4.531878e-003):4.993043e-003):1.595108e-003,1:6.030012e-003);

tree gen.24000 = [&U] ((((((10:8.926737e-003,(((15:3.914724e-003,(3:5.234779e-004,14:7.380592e-004):2.576912e-004):1.273541e-002,8:1.890272e-003):3.030955e-005,(13:1.793798e-004,(11:4.975253e-004,12:1.254838e-004):1.089195e-002):2.820089e-003):1.092629e-003):1.723477e-003,9:5.980242e-004):5.778960e-003,(((41:3.916941e-002,42:5.607454e-003):1.600234e-002,((27:3.182407e-003,(30:2.145661e-003,(((36:2.724708e-003,(((21:5.149735e-004,20:6.574514e-003):2.035948e-003,(25:4.319723e-004,24:5.282126e-004):2.893454e-003):2.409175e-004,26:1.318800e-003):6.289075e-003):5.385331e-003,35:1.432811e-003):3.648388e-003,(((23:4.885353e-005,((7:1.197673e-002,37:1.122958e-003):1.412661e-002,((32:7.281159e-003,34:3.801287e-003):1.034510e-004,((29:1.972503e-003,33:4.488585e-003):5.595721e-004,28:5.785941e-003):3.515937e-003):1.369081e-003):4.596339e-003):5.155942e-004,(38:7.840577e-003,39:1.814302e-005):4.965893e-003):1.339511e-003,2:3.279999e-003):4.229361e-003):4.897634e-003):3.401508e-003):1.444492e-004,(22:7.313591e-003,31:6.061204e-003):4.577138e-004):1.298871e-002):6.834886e-003,18:7.876643e-003):5.688681e-004):2.434244e-003,((5:5.959059e-004,40:1.212604e-002):1.793095e-003,4:4.658910e-003):5.354829e-003):9.404423e-003,(6:2.205141e-003,16:3.833839e-003):2.132101e-003):3.465313e-004,(17:5.015229e-003,19:1.263459e-003):7.927187e-004,1:5.237745e-003);

tree gen.25000 = [&U] ((((((((((15:3.562287e-003,14:1.798614e-004):1.433542e-003,3:1.775644e-003):1.265203e-002,(10:1.868448e-003,(13:1.159128e-004,(11:2.490687e-003,12:2.972086e-003):1.198562e-002):3.568431e-003):1.877704e-004):1.220937e-003,8:4.542548e-004):3.434576e-003,9:4.405384e-004):5.337926e-003,(((41:3.906280e-002,42:5.399757e-003):1.723702e-002,(31:4.949138e-003,((30:5.689169e-004,22:4.269568e-003):1.618814e-003,((((36:1.372176e-003,(21:2.606168e-004,(((25:1.309779e-004,24:9.799433e-004):2.413775e-003,26:4.951850e-003):4.059387e-003,20:1.540905e-003):5.280956e-004):3.081862e-003):9.893253e-003,35:2.433405e-003):4.501894e-003,27:1.632585e-002):1.212541e-004,(((38:2.201954e-003,39:3.619320e-004):7.452055e-003,2:1.863855e-003):1.066225e-003,(23:3.891146e-003,((7:1.066345e-002,37:1.761702e-003):3.518696e-003,(((29:7.898285e-004,(28:5.513864e-003,33:6.978651e-003):2.294933e-003):2.471414e-003,32:1.392615e-002):5.872976e-004,34:5.741226e-003):1.046076e-003):1.918205e-003):1.129523e-003):5.414045e-003):2.528823e-004):8.460704e-004):1.239761e-002):3.317186e-003,18:4.384064e-003):2.280407e-003):7.768331e-003,(40:8.262082e-003,(5:2.337451e-003,4:5.085865e-003):2.868983e-003):6.948364e-004):4.923873e-003,17:4.905454e-003):1.771890e-003,(6:2.037053e-003,16:8.401000e-004):1.308107e-003):3.759416e-004,19:1.594596e-003,1:3.937132e-003);

tree gen.26000 = [&U] ((17:5.106776e-003,((((((13:9.004194e-004,(11:2.026269e-003,12:8.918472e-004):1.860815e-002):6.273790e-003,(((14:5.633435e-004,15:6.384664e-003):1.239023e-003,3:1.447110e-003):4.511535e-003,10:4.728249e-003):1.758934e-004):2.358101e-004,8:9.403107e-004):6.624729e-003,9:9.475838e-004):5.568736e-003,(((41:2.756344e-002,42:5.405210e-003):1.892665e-002,(27:1.286323e-002,((30:1.285791e-003,((((23:3.261223e-003,((7:1.148976e-002,37:1.475669e-003):5.771506e-004,(34:8.391925e-003,(32:5.463945e-003,(33:1.521751e-002,(29:2.540708e-003,28:6.558360e-003):1.268460e-003):5.724315e-003):2.943019e-004):3.702536e-003):1.614944e-003):9.017834e-004,((38:7.348140e-003,39:3.697529e-003):3.748156e-003,2:2.681492e-003):6.730000e-004):3.652030e-003,((36:1.128803e-003,((21:2.880688e-003,20:3.075722e-003):2.770329e-003,(26:6.062334e-003,(25:9.964876e-005,24:5.647568e-004):4.034338e-003):2.389017e-003):4.714006e-003):2.960397e-003,35:1.074263e-002):1.059595e-003):1.981775e-003,31:1.904952e-003):6.743147e-004):4.410206e-004,22:2.169704e-003):3.897165e-004):1.205009e-002):7.621887e-003,18:4.622226e-003):1.241261e-002):9.848501e-003,((40:1.500240e-002,4:1.338401e-002):4.568804e-003,5:2.715973e-003):3.537609e-003):1.057495e-002):2.702635e-003,(19:1.985642e-003,(6:6.567844e-003,16:8.371365e-004):1.897136e-004):1.243012e-003,1:6.590376e-003);

tree gen.27000 = [&U] (17:6.478784e-003,((16:4.872782e-004,6:7.646031e-003):6.084727e-003,(19:2.311858e-003,(((((10:6.689001e-003,((13:1.001592e-003,(11:3.069523e-003,12:7.571486e-004):8.178829e-003):1.218178e-003,(14:1.203446e-005,(15:4.966400e-004,3:1.643670e-003):1.083990e-004):1.426560e-002):3.365579e-003):5.182117e-004,8:1.509709e-003):1.870781e-003,9:8.195834e-004):5.163324e-003,(((41:2.514963e-002,42:8.197128e-003):1.296421e-002,((36:4.850811e-004,((25:8.149208e-005,24:8.874385e-004):2.950201e-003,(26:3.869250e-003,(20:9.142710e-004,21:1.402745e-003):4.395972e-004):3.567879e-003):2.583373e-003):8.404355e-003,((27:5.511126e-003,((35:2.962590e-003,((23:1.165494e-003,((37:2.504738e-003,((32:4.124882e-003,34:1.049811e-002):2.224859e-003,((33:1.174734e-002,29:2.449378e-003):4.308042e-004,28:3.597707e-003):3.725840e-003):6.650675e-003):1.584061e-003,7:9.937716e-003):1.218828e-003):4.347049e-004,(2:2.919752e-003,(38:1.493278e-002,39:5.332251e-003):1.046366e-002):4.995582e-004):3.307754e-003):5.404075e-004,(31:3.158705e-003,22:7.677783e-003):1.321651e-003):7.013928e-004):8.445911e-005,30:1.141699e-003):3.789160e-004):1.363826e-002):7.834621e-003,18:3.997852e-003):6.300834e-003):4.266644e-003,(40:1.063990e-002,(5:7.103704e-004,4:6.508218e-004):2.801024e-003):1.096477e-002):7.500156e-003):1.322302e-003):2.608395e-003,1:9.480250e-003);

tree gen.28000 = [&U] (((((41:2.487245e-002,42:7.683276e-003):1.387115e-002,(31:4.138886e-003,((((27:6.272736e-003,22:3.266201e-003):3.752569e-003,((36:3.931637e-003,((20:4.997686e-003,21:3.895769e-004):4.461807e-003,((25:2.850590e-003,24:2.238189e-003):6.950019e-004,26:2.514322e-003):2.822686e-003):4.780160e-003):2.998759e-003,35:1.354457e-002):1.898913e-003):1.078890e-003,30:3.391152e-003):1.588337e-003,(((38:4.998093e-003,39:2.395206e-003):2.165115e-003,2:3.106960e-003):9.061041e-004,(23:2.553168e-004,((32:6.354812e-003,(((28:4.078081e-003,33:7.708834e-003):1.729897e-003,29:1.226438e-003):3.228985e-003,34:2.185488e-003):5.068117e-004):2.185307e-003,(7:1.162335e-002,37:1.227141e-003):1.785030e-004):7.024186e-004):7.863179e-004):4.513560e-003):3.733514e-004):8.945433e-003):8.364896e-003,(18:3.121729e-003,(9:4.431098e-004,((((10:2.939090e-003,3:1.551786e-003):1.150067e-003,(13:7.884522e-004,(11:1.251414e-003,12:6.092513e-004):1.322881e-002):1.735401e-003):2.293438e-003,(14:1.561669e-004,15:5.591501e-004):3.661707e-003):7.335818e-003,8:9.846822e-004):2.300614e-003):1.982366e-003):3.535524e-003):1.343557e-002,(5:7.939939e-004,(4:2.552227e-004,40:8.459400e-003):5.549713e-004):6.507354e-003):1.110622e-002,(17:1.164745e-002,((6:3.490211e-003,19:2.127422e-003):5.126406e-003,16:4.293113e-003):1.948422e-003):6.079108e-004,1:1.078542e-002);

tree gen.29000 = [&U] (17:4.292350e-003,((6:3.883175e-003,(((18:1.873499e-003,((41:2.307722e-002,42:1.208982e-002):1.190307e-002,(((30:1.964495e-003,22:2.425880e-003):3.192570e-003,((((2:1.797587e-003,(38:5.782321e-003,39:1.586720e-003):3.749908e-003):6.089046e-003,(23:8.202007e-004,(((34:3.442836e-003,32:7.972409e-003):9.414430e-004,((28:2.924019e-003,33:4.414942e-003):9.612748e-004,29:4.325111e-004):6.820486e-003):2.434375e-003,(7:9.974189e-003,37:1.423070e-003):3.843554e-003):1.687630e-003):6.200804e-003):8.050956e-003,((36:4.513915e-003,((26:7.655502e-003,(25:7.703606e-004,24:1.141190e-003):3.456000e-003):3.809993e-004,(20:1.916305e-003,21:1.701289e-003):3.366765e-004):3.303007e-003):7.748789e-004,35:8.196749e-003):2.531572e-003):2.785669e-004,27:6.193799e-003):2.018815e-004):4.895331e-003,31:4.630547e-003):1.493216e-002):2.811048e-003):6.078402e-003,(9:2.824856e-004,((10:2.430178e-003,((15:3.554709e-003,14:1.664028e-004):2.959239e-003,3:2.691766e-003):1.727959e-003):7.048277e-004,((13:3.666242e-003,(11:1.073860e-003,12:5.228087e-004):7.769737e-003):1.481639e-003,8:3.662129e-004):1.372843e-005):1.862246e-003):2.722697e-003):5.190528e-003,(40:7.626777e-003,(4:4.617559e-004,5:4.390720e-004):6.477787e-004):4.777293e-003):4.851268e-003):4.945887e-004,(19:8.146187e-004,16:1.247751e-003):2.605295e-003):3.051573e-003,1:8.743443e-003);

tree gen.30000 = [&U] (((16:1.305793e-003,(17:6.378263e-003,(((18:4.290449e-003,((41:2.464894e-002,42:5.228930e-003):1.321013e-002,(((31:5.066544e-003,30:1.449593e-003):5.868479e-004,(((36:4.137782e-003,((21:4.817914e-003,(20:5.195055e-004,26:4.956228e-003):2.403124e-003):1.651620e-003,(25:1.718185e-003,24:5.199721e-004):1.092771e-003):2.784331e-003):4.417496e-003,35:8.755007e-003):4.218225e-003,(27:1.246168e-002,22:3.722210e-003):1.251280e-003):3.669729e-004):1.558917e-004,(23:7.932738e-005,(((38:5.790357e-003,39:1.538900e-003):4.301976e-003,2:2.062263e-003):4.701276e-003,(((((29:4.862173e-003,28:3.057319e-003):1.479165e-003,33:4.715631e-003):2.145495e-003,34:1.009094e-002):2.296610e-003,32:6.907491e-003):4.344086e-004,(7:1.065350e-002,37:4.967743e-003):3.699464e-003):3.407003e-003):3.804269e-004):8.894481e-003):2.151603e-002):2.271356e-003):8.462920e-003,(9:2.857871e-003,((((13:1.360329e-003,(11:2.870798e-003,12:1.145875e-005):5.839186e-003):2.112461e-003,10:1.384630e-003):3.456884e-005,((15:3.759686e-004,3:1.204415e-003):7.648570e-003,14:1.471569e-003):3.807362e-004):2.566215e-004,8:2.440633e-003):3.797913e-004):7.494510e-003):3.557138e-003,((5:6.725892e-004,40:8.011915e-003):2.522636e-003,4:2.293589e-003):7.362061e-003):4.743251e-003):3.173525e-003):4.370043e-003,6:1.423736e-003):4.333359e-004,19:1.861608e-003,1:6.690791e-003);

tree gen.31000 = [&U] (((((18:2.734835e-003,((41:2.977675e-002,42:3.991921e-003):2.435379e-002,((((22:1.378818e-003,((36:3.671239e-003,(((20:8.760688e-004,(25:2.825405e-003,24:8.050953e-004):3.648550e-003):3.845956e-003,26:5.139729e-003):1.114380e-003,21:5.327091e-003):1.215876e-003):3.400069e-003,35:5.827558e-003):1.469671e-003):4.800747e-004,(2:3.094427e-003,((38:5.538144e-003,39:1.326855e-003):2.196024e-003,(((32:2.017683e-002,(34:1.007574e-002,((28:1.702046e-003,33:4.830860e-003):4.077872e-004,29:5.858671e-003):1.176203e-003):6.606408e-004):4.184152e-004,(7:1.046022e-002,37:7.427369e-004):2.266076e-003):4.984275e-003,23:4.145484e-003):2.557718e-004):2.506326e-003):7.179522e-003):1.022419e-003,27:6.373088e-003):1.000736e-003,(30:4.794183e-003,31:6.241715e-003):2.638341e-005):4.364820e-003):6.265406e-003):2.005444e-003,(9:2.853102e-003,((((15:1.415127e-004,3:5.731561e-004):2.807430e-003,14:4.052275e-004):1.032263e-002,((13:1.313471e-003,(11:3.064313e-003,12:1.444642e-005):8.400439e-003):9.551067e-004,10:4.374842e-003):1.062557e-003):9.442102e-004,8:4.680470e-004):2.155638e-003):5.023778e-003):4.078147e-003,(4:2.251978e-003,(40:1.406858e-002,5:4.440473e-004):1.255156e-004):6.627292e-003):1.228471e-002,(19:2.655078e-003,17:3.101366e-003):4.247349e-003):2.503897e-003,(6:5.030457e-003,16:1.710403e-003):1.731848e-003,1:6.156306e-003);

tree gen.32000 = [&U] (((6:4.012773e-003,(19:1.326695e-003,16:2.140990e-003):8.153450e-004):2.181945e-003,(((18:9.508300e-003,((41:2.632046e-002,42:2.461054e-003):1.515236e-002,(27:4.377369e-003,((31:6.025718e-003,((((38:7.279443e-003,39:5.264687e-003):5.172500e-003,2:2.686525e-003):1.146680e-003,((((28:4.547208e-003,(29:1.275246e-003,33:1.974767e-003):2.101518e-005):9.301159e-003,(34:1.002928e-002,32:1.821853e-002):1.283268e-003):4.371482e-003,(7:9.635126e-003,37:3.052251e-004):1.986151e-003):4.015294e-004,23:5.071078e-003):2.682365e-003):9.983330e-003,(22:1.615690e-003,30:9.396492e-004):2.969177e-004):9.409582e-004):3.681569e-003,((36:5.237794e-003,(((25:3.582353e-003,24:3.270149e-004):2.842890e-003,20:6.763959e-004):1.080172e-003,(26:2.717981e-003,21:6.674408e-004):6.655452e-004):3.945647e-003):5.876622e-003,35:9.012738e-003):5.874875e-003):1.560226e-003):9.530285e-003):5.617542e-003):1.076881e-003,(9:1.553226e-004,(8:5.981994e-004,(((14:1.614838e-004,(15:1.169830e-003,3:1.680904e-005):2.517522e-003):1.112940e-002,10:4.447298e-004):8.055796e-004,((11:1.252484e-003,12:2.210933e-005):1.015456e-002,13:3.056648e-003):7.882270e-004):1.881962e-003):2.836387e-003):2.537502e-003):6.789726e-003,((5:9.011094e-004,40:1.008421e-002):6.882216e-004,4:6.425300e-004):8.137836e-003):1.318279e-002):3.467547e-003,17:7.425046e-003,1:3.582888e-003);

tree gen.33000 = [&U] ((((6:3.542048e-003,16:1.920687e-003):4.966849e-003,17:8.951782e-003):1.591711e-003,19:7.749677e-004):1.187113e-003,(((18:9.367729e-003,((41:4.187755e-002,42:6.024664e-003):2.410839e-002,(31:8.372872e-003,(((((36:7.400550e-003,((20:1.161291e-003,(25:6.637069e-004,24:8.672509e-004):4.823997e-003):1.854940e-003,(21:1.295178e-003,26:3.432099e-003):3.598477e-003):6.911315e-003):4.484708e-003,35:6.930243e-003):4.339165e-003,22:1.945479e-003):8.605964e-004,(((38:7.337995e-003,39:8.157955e-004):1.581832e-003,(((((28:1.804180e-003,33:6.376861e-003):1.359101e-004,29:6.936311e-003):5.020706e-003,(32:1.426796e-002,34:7.169517e-003):1.240634e-003):6.107099e-003,(7:7.604233e-003,37:3.770912e-004):7.083477e-003):1.657262e-002,23:2.200131e-003):8.671191e-003):2.415282e-003,2:1.153702e-003):9.231865e-003):2.610139e-003,(27:4.896255e-003,30:6.893003e-003):5.956914e-004):4.672406e-004):9.077691e-003):1.492943e-003):1.348898e-003,(9:2.613101e-004,((((11:4.195505e-003,12:2.605614e-003):6.145895e-003,13:4.863324e-003):1.013111e-002,8:7.800032e-004):2.677703e-004,((14:1.261263e-004,(3:2.732257e-003,15:2.417512e-004):7.561151e-003):4.200022e-003,10:6.232427e-003):7.072878e-003):3.470009e-003):6.312627e-003):1.772967e-003,((5:4.530294e-003,40:1.604463e-002):7.953387e-004,4:5.590351e-003):1.881727e-003):1.553359e-002,1:5.842856e-003);

tree gen.34000 = [&U] (((17:1.375492e-002,19:5.931359e-003):9.182254e-004,(((9:3.577448e-005,(((11:7.732716e-003,12:6.562724e-004):7.859259e-003,13:6.780407e-004):2.969301e-003,(((15:1.737689e-003,3:2.025502e-003):6.501231e-003,14:1.828834e-003):8.838862e-003,(8:4.269661e-004,10:7.954713e-003):1.274820e-003):3.741139e-004):3.260390e-003):4.683967e-003,(((41:3.523860e-002,42:8.984684e-003):2.141927e-002,(27:4.851767e-003,(31:3.332561e-003,(22:2.792902e-003,(((36:1.503574e-002,(((25:1.172395e-003,24:6.738221e-004):4.476198e-003,26:3.575506e-003):2.578666e-003,(21:1.489698e-003,20:2.272027e-003):1.081300e-003):2.514085e-003):6.397684e-003,35:6.655562e-003):7.811257e-003,(((2:9.757365e-003,((((34:8.774152e-003,((28:7.489391e-003,33:5.471726e-003):1.497044e-003,29:1.962419e-003):3.629880e-003):3.050887e-003,32:1.329862e-002):8.412816e-003,(7:1.282452e-002,37:1.374184e-003):5.774069e-003):2.405791e-003,23:3.423364e-004):3.342890e-003):2.140358e-003,(38:5.456199e-003,39:7.441375e-004):5.085812e-003):2.431864e-003,30:1.073855e-002):2.247329e-003):2.538473e-003):1.498585e-003):4.098044e-004):1.491285e-002):7.550296e-003,18:5.058383e-003):8.294787e-003):1.465939e-002,((4:2.249537e-003,5:1.167415e-003):1.419435e-003,40:1.449231e-002):1.092506e-002):1.824462e-002):2.767241e-003,(6:3.230921e-003,16:2.625404e-003):4.781417e-003,1:8.928824e-003);

tree gen.35000 = [&U] ((6:3.868793e-003,16:4.048841e-003):6.455927e-003,((19:2.925106e-003,17:1.700650e-002):7.665044e-003,(((9:3.629890e-003,((((11:9.391762e-004,12:5.848287e-003):7.497390e-003,13:3.672439e-003):3.258737e-003,(((3:3.627095e-003,15:1.264167e-003):1.509477e-003,14:3.852269e-003):1.245884e-002,10:5.280088e-003):2.281840e-003):1.694627e-003,8:8.975545e-004):6.350145e-003):4.423401e-003,(((41:3.721994e-002,42:6.621492e-003):1.205349e-002,(30:1.425571e-003,((((((((33:9.351835e-003,29:3.635102e-003):8.747345e-004,28:7.395632e-003):1.187410e-002,(32:6.072079e-003,34:6.566055e-003):2.616358e-003):1.004002e-003,(7:1.004846e-002,37:1.451449e-003):4.854151e-003):5.567433e-003,23:1.766038e-005):3.644466e-003,((38:4.941086e-003,39:2.011605e-004):3.095518e-003,2:4.440738e-003):7.730338e-004):4.386780e-003,22:2.544837e-003):1.332114e-003,(((36:6.343603e-003,(20:2.015519e-003,(26:4.240259e-003,(21:4.270601e-003,(25:1.138919e-004,24:6.023544e-004):1.547021e-003):3.107928e-004):3.228340e-003):1.051405e-003):9.709683e-003,35:7.621121e-003):1.565138e-003,(27:1.654539e-002,31:1.187717e-002):2.145676e-004):2.717287e-003):4.836776e-004):1.654793e-002):6.588049e-003,18:7.962781e-003):8.293466e-004):7.899108e-003,(40:1.882074e-002,(4:1.576791e-003,5:2.584120e-004):3.406716e-003):4.071417e-003):6.462651e-003):8.040130e-004,1:9.491635e-003);

tree gen.36000 = [&U] (((((9:9.225131e-004,((10:4.743349e-003,(((11:2.185626e-003,12:5.770759e-003):2.916117e-003,13:2.337510e-004):1.026669e-002,((15:1.998369e-004,14:1.583235e-003):4.916443e-004,3:4.153856e-004):1.832573e-003):2.694242e-003):7.370473e-003,8:5.442271e-003):1.762735e-003):2.915312e-003,(((41:5.385406e-002,42:5.948394e-003):1.082821e-002,(((31:1.809258e-002,27:1.721153e-002):9.134599e-004,((36:2.460857e-003,((21:7.246920e-004,((25:5.594026e-004,24:1.403874e-003):5.740576e-003,20:2.151332e-004):5.617681e-004):2.796033e-003,26:5.457301e-003):3.797390e-003):4.137389e-003,35:1.362440e-002):4.272882e-003):2.681687e-003,(30:2.910718e-003,((((((34:6.963220e-003,(33:9.132263e-003,(28:8.024604e-003,29:4.365770e-003):5.162284e-004):2.423658e-003):3.379955e-003,32:1.815768e-002):6.244473e-003,(7:1.199327e-002,37:3.572046e-003):2.872816e-003):2.872134e-003,23:3.165097e-003):5.293023e-003,(2:2.246900e-003,(38:7.379868e-003,39:4.645214e-003):5.290445e-003):1.082701e-003):1.044181e-002,22:4.011604e-003):2.988366e-003):1.395393e-003):6.339918e-003):7.435229e-003,18:3.747781e-003):3.139898e-003):1.059436e-002,(4:6.239243e-003,(40:1.639437e-002,5:1.625007e-003):2.794395e-003):5.297075e-003):7.381321e-003,19:9.287757e-003):1.971280e-003,((6:1.952211e-003,16:6.950908e-003):2.019093e-003,17:1.550450e-002):3.323055e-003,1:1.597511e-002);

tree gen.37000 = [&U] ((19:8.776612e-003,(17:5.382802e-003,(6:6.496025e-003,16:4.778842e-003):3.362521e-003):4.615840e-004):7.356339e-004,(((9:1.437802e-003,((10:5.054859e-003,(8:2.727081e-004,(3:1.794595e-004,(14:1.483118e-003,15:2.215163e-004):1.483950e-003):8.602151e-003):2.702027e-004):1.659930e-003,((11:1.492878e-003,12:7.251557e-004):6.695082e-003,13:2.567673e-003):1.678070e-003):2.833454e-003):3.985099e-003,(((41:3.536205e-002,42:5.319632e-003):2.774516e-002,(31:1.073369e-002,(((27:4.006802e-003,((2:6.370565e-003,(38:7.780941e-003,39:3.837367e-003):5.621971e-003):6.348733e-003,(((((29:2.158641e-003,(28:9.117079e-003,33:3.770045e-003):7.199189e-004):5.188419e-003,32:6.310133e-003):1.391097e-003,34:4.690377e-003):3.722530e-003,(7:4.678423e-003,37:2.248604e-003):6.037418e-003):1.482434e-002,23:2.138492e-004):1.034854e-002):7.160356e-003):7.257489e-004,(22:1.092622e-003,30:2.767625e-003):9.336801e-004):8.455982e-004,((36:4.403020e-003,(((25:1.640181e-003,24:3.664112e-004):4.108220e-003,(20:3.366975e-003,21:1.164649e-003):1.371602e-003):4.905748e-003,26:6.466704e-003):4.555410e-003):4.646873e-003,35:1.555422e-002):2.776098e-003):2.080257e-003):8.649139e-003):8.279834e-003,18:9.353084e-003):1.218545e-003):4.070108e-003,(40:2.211593e-002,(5:4.226684e-003,4:3.335234e-003):3.038456e-003):2.659506e-003):1.978910e-002,1:1.343961e-002);

tree gen.38000 = [&U] (((((9:5.706243e-004,((10:4.361050e-003,((11:1.248597e-003,12:6.064980e-004):5.599561e-003,13:5.960483e-004):1.377706e-003):1.066061e-004,(8:4.581512e-003,((3:2.410567e-003,15:1.616219e-003):7.120710e-004,14:3.522180e-003):6.265624e-003):3.748311e-004):3.909481e-003):2.165477e-002,(((41:4.276627e-002,42:2.543553e-002):1.193048e-002,((27:3.166092e-003,(((36:1.546958e-003,(((25:2.317679e-003,24:4.458755e-004):3.047847e-003,(21:7.445115e-004,20:1.891605e-003):1.100604e-002):1.398969e-003,26:5.418040e-003):2.749675e-003):6.809947e-003,35:1.300907e-002):2.067828e-003,(31:8.442142e-003,30:8.197452e-003):1.376800e-003):1.053127e-003):1.262448e-003,(22:3.552635e-004,((2:5.595399e-003,(38:1.325030e-002,39:2.731231e-003):2.717083e-003):1.304865e-003,((((33:2.447249e-003,(29:4.944006e-003,28:6.185125e-003):6.121509e-006):7.159003e-003,34:1.629171e-002):1.426499e-003,((7:1.021522e-002,37:5.445988e-003):3.058773e-003,32:1.688272e-002):2.197675e-003):2.612844e-003,23:3.309942e-005):4.567459e-003):1.235110e-002):3.302510e-003):1.058628e-002):3.140489e-003,18:7.822632e-003):2.229538e-003):9.277565e-003,(40:1.411944e-002,(4:1.139247e-003,5:3.084452e-003):3.801529e-003):9.046393e-003):2.491768e-002,17:3.353594e-003):2.541170e-003,((16:3.959617e-003,19:3.266254e-004):7.232202e-007,6:5.887435e-003):2.538449e-003,1:1.698318e-002);

tree gen.39000 = [&U] (((((9:1.138993e-003,(((15:1.926808e-003,14:1.823689e-003):6.447011e-005,3:3.423848e-003):1.138577e-002,((8:4.765783e-003,10:3.292266e-003):1.775656e-003,((11:7.150064e-003,12:3.685215e-003):1.153495e-002,13:3.502808e-004):4.747451e-003):1.014342e-003):4.080994e-003):2.201511e-003,(((41:3.936791e-002,42:1.744377e-002):1.015852e-002,(31:7.914295e-003,((27:1.266416e-002,(30:2.100533e-003,(((36:2.286793e-003,(26:6.480665e-003,(20:8.671804e-004,((25:8.716696e-004,24:4.609309e-003):2.658809e-003,21:2.459223e-004):7.867986e-006):7.368736e-004):3.731647e-003):2.746180e-003,35:4.758494e-003):3.456918e-003,22:7.247997e-004):5.547464e-004):6.676127e-004):1.206379e-003,((38:1.219738e-002,39:1.174378e-003):2.672960e-003,(2:5.345691e-003,((((((28:2.006029e-003,33:4.473840e-003):2.834788e-004,29:2.784385e-003):1.033719e-002,32:6.669323e-003):2.466322e-003,34:7.389327e-003):2.605540e-003,(7:9.403479e-003,37:3.343398e-003):1.783379e-003):5.607792e-003,23:1.605569e-004):4.610579e-003):6.080820e-003):1.813524e-003):2.646114e-003):1.729524e-002):9.414077e-003,18:9.998578e-003):2.941766e-003):4.506341e-003,((4:2.213864e-003,5:5.113407e-003):2.758341e-004,40:1.242950e-002):1.002495e-002):8.161922e-003,(16:2.416908e-003,(19:5.809991e-003,6:2.990004e-003):2.189847e-003):5.937223e-003):1.472225e-003,17:1.004755e-002,1:1.149693e-002);

tree gen.40000 = [&U] ((((9:1.608793e-003,((((15:9.916466e-004,(14:5.345745e-003,3:1.375916e-003):4.456799e-003):1.217942e-002,10:5.342934e-003):4.103636e-003,8:5.938461e-004):2.844483e-003,((11:1.345941e-002,12:1.391619e-003):1.105053e-002,13:3.487499e-004):3.314846e-003):2.059145e-003):7.331719e-003,(((41:4.338196e-002,42:4.756286e-003):2.282169e-002,((31:5.418892e-004,((((((32:7.349345e-003,(34:9.893756e-003,((28:2.100724e-003,29:4.599109e-003):2.205117e-004,33:4.930004e-003):5.426589e-003):4.630198e-003):6.726937e-003,(7:1.362160e-002,37:5.463205e-003):4.497072e-003):1.907967e-003,23:3.592092e-004):2.361561e-003,2:7.619698e-003):1.770535e-005,(38:9.809243e-003,39:3.554360e-003):8.288679e-003):3.558502e-003,30:8.689368e-003):1.912260e-003):3.180054e-003,(((36:4.794640e-003,((26:4.135894e-003,20:7.006527e-004):5.685454e-003,(21:1.765273e-003,(25:5.378655e-003,24:3.035959e-003):6.839035e-003):7.911366e-003):5.014574e-003):5.794517e-003,35:5.933301e-003):5.521972e-003,(22:2.282555e-003,27:1.363339e-002):1.078361e-003):1.592956e-003):2.848141e-002):1.037396e-002,18:2.999888e-003):1.010611e-003):5.462771e-003,(4:9.130364e-004,(5:6.718288e-003,40:1.460675e-002):2.914838e-003):3.435974e-003):1.474584e-002,((6:1.196167e-003,16:4.999777e-003):4.897251e-003,(19:6.318706e-003,17:1.495126e-002):1.211932e-003):1.209515e-003,1:1.480986e-002);

tree gen.41000 = [&U] ((((19:1.648226e-003,6:1.201001e-003):4.317125e-004,16:5.239211e-003):4.640173e-003,(((9:7.070333e-004,((((3:9.584074e-004,(15:3.858691e-003,14:5.197153e-003):3.716043e-003):1.029246e-002,8:5.620853e-004):2.282640e-003,10:8.107827e-003):1.246468e-003,((11:3.243007e-003,12:3.293207e-004):1.619535e-002,13:1.739691e-004):6.037909e-004):5.457714e-003):1.276731e-002,(((41:3.817508e-002,42:1.675112e-002):7.000129e-003,((27:7.859977e-003,((22:3.702957e-003,((36:1.384150e-002,(((21:2.503775e-003,(25:1.979359e-003,24:4.815214e-004):1.137799e-002):3.070105e-003,20:2.992214e-003):2.100535e-003,26:4.134058e-003):6.326450e-003):1.292647e-003,35:1.712837e-002):7.766760e-003):6.437929e-004,(((2:4.313173e-003,(38:1.419840e-002,39:5.976444e-003):2.931482e-003):6.742566e-005,((((34:6.956849e-003,(29:1.080650e-003,(33:6.397541e-003,28:4.635249e-003):1.732043e-003):3.242210e-003):2.905690e-003,32:9.784731e-003):7.899261e-003,(7:1.198668e-002,37:6.357849e-004):1.150770e-002):7.929101e-003,23:5.104589e-004):1.033975e-003):3.230848e-003,30:5.222721e-003):7.519521e-005):1.170155e-003):9.160898e-004,31:5.139935e-003):1.312752e-002):1.135997e-002,18:4.573930e-003):6.209631e-003):7.661402e-003,((40:3.401110e-002,4:6.076982e-003):2.935907e-003,5:4.874419e-004):6.871747e-003):1.038149e-002):1.855118e-003,17:1.152283e-002,1:9.856402e-003);

tree gen.42000 = [&U] (((((8:2.203145e-003,(10:8.851724e-003,(9:2.697759e-003,((3:6.750774e-004,15:4.216901e-003):1.034335e-003,14:2.247644e-004):1.218683e-002):3.344227e-003):9.848365e-004):1.177264e-003,((11:4.438021e-003,12:3.959318e-004):1.754764e-002,13:1.414824e-004):3.590952e-003):1.196501e-002,(((41:4.707459e-002,42:1.146681e-002):3.427319e-002,((22:9.876482e-003,(27:1.015249e-002,((((((((33:7.236901e-003,29:4.252147e-003):2.102135e-003,28:5.416932e-003):3.485758e-003,32:1.450075e-002):9.966340e-004,34:7.263760e-003):7.387809e-003,(7:1.285786e-002,37:3.287740e-003):8.550194e-003):1.856499e-004,23:7.038271e-004):2.069665e-003,(38:1.640658e-002,39:1.008670e-002):3.776277e-003):5.308257e-003,2:6.248600e-003):2.598363e-003):1.321123e-003):2.389357e-004,(30:2.099037e-003,(31:3.162735e-003,(35:8.135383e-003,(36:1.402405e-002,(((20:5.978318e-006,26:6.109058e-003):3.022532e-003,(25:2.421467e-003,24:3.791453e-003):2.802084e-003):4.761268e-003,21:1.482431e-003):5.256749e-003):1.112516e-002):1.162158e-002):4.010418e-004):3.505489e-004):1.847185e-002):3.245408e-003,18:7.463409e-003):2.322842e-003):6.630889e-003,(5:2.378784e-003,(40:1.960551e-002,4:6.602209e-003):3.559660e-003):8.249705e-003):9.404805e-003,(17:1.503245e-002,(19:7.557925e-003,(6:2.777942e-003,16:5.566073e-003):1.593164e-003):8.530931e-003):1.002648e-003,1:1.359185e-002);

tree gen.43000 = [&U] ((19:2.787451e-003,17:1.020321e-002):1.225734e-003,((((((41:4.192798e-002,42:3.990531e-003):2.353568e-002,(((((32:1.064172e-002,(34:9.847143e-003,((28:3.019294e-003,33:5.176832e-003):2.470573e-003,29:8.051261e-003):1.160789e-002):1.051123e-003):1.120468e-003,(7:8.434692e-003,37:5.007846e-004):9.842339e-003):4.619126e-003,23:3.825323e-003):7.385954e-003,(2:1.352530e-002,(38:5.723928e-003,39:5.181402e-003):1.649164e-002):1.822686e-003):4.510199e-003,(((35:1.531518e-002,(36:1.001713e-002,(((20:4.021154e-006,(25:2.992216e-003,24:2.392958e-003):1.965549e-003):5.388907e-003,26:4.766469e-003):4.967870e-003,21:1.114943e-003):9.875583e-003):1.107003e-002):3.451480e-003,27:7.997171e-003):1.042630e-003,(31:1.379469e-002,(22:3.493493e-003,30:8.863390e-003):2.776844e-004):5.618803e-004):1.974078e-003):1.491694e-002):2.175615e-003,18:8.131990e-003):8.499596e-003,(10:1.229516e-002,(((14:2.352387e-004,3:1.430405e-004):1.483358e-003,15:7.017336e-003):1.014928e-002,(9:1.757345e-002,(8:1.056281e-003,((11:5.157455e-003,12:2.272730e-003):7.740973e-003,13:1.916391e-003):1.863325e-003):8.754462e-005):1.790952e-003):5.396907e-004):9.233457e-003):8.520068e-003,(40:1.829252e-002,(4:5.221747e-003,5:3.596841e-004):3.872083e-003):7.677214e-003):1.402635e-002,(6:1.091316e-002,16:2.402714e-003):2.075179e-003):2.086803e-005,1:1.487496e-002);

tree gen.44000 = [&U] (17:7.307487e-003,(((19:4.896107e-003,16:7.158490e-003):1.637948e-003,6:8.897819e-003):3.993303e-003,(((((41:3.947535e-002,42:9.009558e-003):2.029972e-002,((22:6.930117e-003,(31:1.075273e-002,(30:5.467426e-003,27:7.511573e-003):7.948398e-004):8.987150e-004):7.689983e-004,((35:1.441930e-002,(36:9.431167e-003,((21:4.696914e-003,((26:4.528815e-003,25:4.663412e-004):1.239725e-003,24:9.170328e-004):1.170717e-003):3.352069e-003,20:9.591495e-004):6.427830e-003):1.102187e-002):3.029644e-003,((2:1.258182e-003,(38:1.755691e-002,39:1.029417e-003):5.040065e-003):1.004199e-003,((((32:1.272289e-002,((29:9.186898e-003,33:5.712278e-003):1.774778e-003,28:6.171488e-003):5.302212e-003):2.449877e-003,34:3.161585e-003):8.305583e-003,(7:1.060596e-002,37:1.027431e-002):8.791417e-003):3.196665e-003,23:1.230490e-003):5.209650e-003):8.690981e-003):5.487971e-003):1.255999e-002):4.365649e-003,18:1.288135e-002):2.522404e-003,(((10:9.546150e-003,(((11:1.602628e-003,12:2.139784e-003):1.130020e-002,13:2.155499e-003):8.907363e-003,(3:2.320027e-003,(14:8.522905e-004,15:4.967540e-003):4.833300e-003):5.595422e-003):4.771079e-003):1.227697e-002,8:3.261103e-004):6.765166e-004,9:2.177099e-003):1.365876e-002):6.716684e-003,((40:1.606214e-002,5:1.368700e-003):1.490268e-003,4:7.997200e-004):2.914375e-003):9.781805e-003):2.601079e-003,1:1.377058e-002);

tree gen.45000 = [&U] (17:1.373940e-002,((19:1.398166e-003,(16:1.023026e-002,6:1.070524e-002):2.064347e-003):1.586670e-003,(((((41:3.514655e-002,42:5.326139e-003):2.065939e-002,((((22:8.051739e-003,27:1.224180e-002):1.730058e-003,30:2.300673e-003):3.598108e-004,(31:1.192397e-002,(35:2.125248e-002,(36:5.360140e-003,(((25:5.682726e-004,24:2.589404e-003):1.486926e-003,21:1.377724e-003):7.119208e-004,(26:3.900943e-003,20:4.871645e-003):6.767000e-004):6.917776e-003):7.784234e-003):2.688768e-003):8.080209e-005):2.699688e-003,(((38:1.077640e-002,39:2.926813e-003):4.783113e-003,2:6.159908e-003):8.646923e-004,((((34:1.172400e-002,((28:8.267600e-003,29:2.640229e-003):4.647265e-004,33:6.960851e-003):2.101213e-003):9.032034e-004,32:1.023963e-002):5.806854e-003,(7:6.209055e-003,37:3.320011e-003):1.071302e-002):5.355122e-003,23:1.885984e-003):3.805082e-003):8.970284e-003):1.333789e-002):5.319879e-003,18:3.207670e-003):2.328198e-003,(((14:4.825446e-003,(15:2.559921e-003,3:2.716068e-003):5.261340e-004):6.449774e-003,(10:4.646051e-003,(8:8.173660e-004,(13:1.422477e-003,(11:9.930601e-003,12:8.339713e-004):1.902990e-002):3.349848e-003):1.344945e-004):1.440505e-003):1.109532e-003,9:3.554791e-004):9.474362e-003):1.086272e-002,(40:1.406299e-002,(5:5.632416e-004,4:3.419362e-003):4.837445e-003):9.870677e-003):1.396868e-002):6.363356e-003,1:1.309793e-002);

tree gen.46000 = [&U] ((((((41:2.746770e-002,42:6.569813e-003):1.517526e-002,((22:1.316423e-003,((27:6.754262e-003,(((38:8.915612e-003,39:1.946011e-003):4.241037e-003,(((((32:1.419503e-002,34:1.074864e-002):2.882074e-004,((28:7.115264e-003,33:8.913901e-004):1.024174e-003,29:5.427583e-003):1.427009e-002):3.783458e-003,(7:5.293651e-003,37:6.696568e-004):3.791204e-003):1.825659e-003,23:1.242745e-002):3.718284e-003,2:1.476951e-003):7.955569e-004):9.343578e-003,31:1.016601e-002):2.657929e-003):2.670046e-004,30:2.781388e-003):7.593965e-004):7.192878e-003,(35:8.935771e-003,(36:5.918543e-003,((26:3.300151e-003,(25:2.664377e-003,24:2.207647e-003):7.019560e-004):4.006165e-003,(21:1.309005e-003,20:2.491202e-005):2.164415e-003):5.582390e-003):7.413956e-003):1.431378e-003):2.140102e-002):5.243652e-003,18:7.516053e-003):3.134370e-003,((8:2.230414e-003,((10:1.885872e-002,((11:8.692765e-003,12:1.394305e-005):1.199107e-002,13:1.454002e-003):3.608028e-003):2.342213e-005,(15:2.182511e-003,(3:2.167750e-003,14:8.370055e-004):5.084674e-004):9.269808e-003):3.292229e-003):1.570810e-003,9:1.540001e-003):2.765772e-003):5.298690e-003,((5:5.556603e-003,4:4.392670e-003):6.408160e-004,40:1.281029e-002):9.489698e-003):1.824828e-002,(17:1.318254e-002,(6:1.744217e-003,(19:9.258171e-004,16:8.289036e-003):2.764285e-003):1.942680e-003):2.916920e-003,1:1.113652e-002);

tree gen.47000 = [&U] ((((((41:4.032114e-002,42:1.864636e-002):2.227649e-002,(((((((29:1.110083e-003,33:1.032468e-002):1.413240e-003,28:6.693681e-003):7.927670e-003,(32:4.126538e-003,34:1.769281e-002):1.619160e-003):2.753023e-003,(7:1.040291e-002,37:1.806859e-003):6.805125e-004):1.009493e-002,23:4.304448e-003):1.101020e-002,((38:9.686679e-003,39:4.286189e-003):1.040123e-003,2:4.790411e-003):1.120065e-003):7.989937e-003,(22:2.225866e-003,(27:3.900378e-003,((30:4.019635e-003,(35:4.462157e-003,(36:8.688109e-003,((21:3.596737e-003,(25:2.749366e-003,(26:4.847166e-003,24:4.237925e-003):2.751492e-004):1.138857e-002):4.989228e-003,20:2.610121e-004):9.128083e-003):8.740214e-003):1.084196e-002):1.506025e-003,31:1.369772e-002):2.018328e-003):2.557460e-003):9.795275e-004):2.020350e-002):4.538484e-003,18:4.374338e-003):4.408821e-003,(((8:3.678908e-003,10:1.364140e-002):5.733699e-004,(((11:4.523602e-003,12:4.040705e-004):5.501608e-003,13:1.874228e-003):1.441874e-003,((15:3.203812e-003,3:1.419668e-003):6.383924e-004,14:4.520048e-003):8.654386e-003):4.555781e-003):7.390321e-003,9:1.983973e-003):8.781954e-003):7.778198e-003,((40:2.422097e-002,4:2.158510e-003):2.237193e-005,5:1.194591e-003):4.881615e-003):5.815422e-003,(17:1.779263e-002,((19:1.094047e-002,16:3.930299e-003):1.511445e-004,6:6.181190e-003):1.307979e-002):8.310585e-003,1:9.870345e-003);

tree gen.48000 = [&U] ((((((41:2.962028e-002,42:7.968769e-003):1.800813e-002,(((30:3.722882e-003,22:5.017396e-003):4.992448e-004,((31:3.120336e-003,((2:1.485820e-003,(((34:1.519572e-002,(32:5.769546e-003,(29:8.296373e-003,(28:4.942725e-003,33:2.760384e-003):2.675605e-005):6.563615e-003):9.122501e-004):7.010377e-004,(7:5.917508e-003,37:1.327336e-003):1.186297e-002):3.381963e-003,23:1.283869e-003):3.872216e-003):9.125951e-004,(38:6.130382e-003,39:1.670718e-003):3.131496e-003):5.311665e-003):6.749357e-004,(35:6.592840e-003,(36:1.149204e-002,(((21:2.629872e-003,26:9.961698e-003):9.492858e-004,20:2.131262e-003):4.003211e-003,(25:1.551349e-004,24:3.898588e-004):2.644734e-003):7.595796e-003):8.724137e-003):2.937996e-003):3.160388e-004):8.621508e-005,27:8.918852e-003):1.645705e-002):2.175340e-003,18:7.037616e-003):5.490742e-003,((10:1.002110e-002,(((11:2.522743e-003,12:1.248706e-002):1.148751e-002,13:1.186351e-003):3.600292e-003,(((15:1.761104e-003,14:4.168032e-004):2.015297e-003,3:1.283835e-002):5.928694e-003,8:5.094171e-004):1.452935e-003):3.418665e-005):1.375951e-003,9:2.933196e-004):4.800011e-003):4.337239e-003,((4:8.476482e-003,40:1.679474e-002):5.838949e-004,5:2.010919e-004):8.944150e-003):6.782636e-003,((19:2.294401e-003,(16:1.413794e-002,6:4.196434e-003):2.239408e-003):1.955486e-003,17:1.066085e-002):4.976643e-004,1:8.544840e-003);

tree gen.49000 = [&U] (((((((41:3.200609e-002,42:8.610624e-003):1.039819e-002,((((22:3.954358e-003,(35:8.719716e-003,(36:8.767509e-004,((20:2.080171e-003,((25:4.935641e-004,24:3.753723e-003):9.670126e-003,21:1.914020e-003):4.176734e-003):2.271252e-003,26:6.302983e-003):5.517774e-003):3.610956e-003):2.299643e-003):1.547340e-004,(2:3.016509e-003,((((((33:6.291420e-003,(28:3.345452e-003,29:1.031173e-003):7.912330e-004):5.822330e-003,32:7.622253e-003):1.423135e-003,34:1.240156e-002):1.065252e-003,(7:6.394142e-003,37:4.562180e-004):4.290097e-003):9.826016e-004,23:1.155336e-003):1.836754e-003,(38:1.025841e-002,39:1.284192e-003):5.054564e-003):1.258801e-004):2.453388e-003):2.531899e-003,(31:3.571659e-003,30:1.586623e-003):4.131444e-003):2.058617e-003,27:9.396492e-003):1.707184e-002):5.257999e-003,18:9.238365e-003):6.466047e-003,((8:7.569721e-005,((10:5.462096e-003,(3:1.246029e-002,(14:1.655456e-003,15:1.038218e-003):8.890354e-004):6.740491e-003):5.235595e-004,((11:2.725941e-003,12:4.437044e-004):1.079122e-002,13:5.503302e-003):9.214317e-004):1.051897e-002):2.774067e-004,9:7.963550e-004):3.362810e-003):3.952704e-003,((40:1.805592e-002,4:7.211295e-003):5.357378e-005,5:2.712924e-003):9.410064e-003):1.110234e-002,(16:3.646079e-003,6:8.885575e-003):7.670506e-003):6.463049e-003,(17:1.200720e-002,19:1.895535e-003):1.734533e-003,1:8.261897e-003);

tree gen.50000 = [&U] ((6:7.131666e-003,(16:3.096651e-003,19:5.483282e-003):1.658244e-003):2.277451e-004,(17:9.415331e-003,(((((41:4.116210e-002,42:2.044558e-002):1.196559e-002,(((((38:9.855966e-003,39:7.672006e-004):7.013684e-003,((((34:5.058358e-003,32:7.195319e-003):6.823847e-004,(33:5.910040e-003,(29:7.330379e-004,28:3.324580e-003):3.290721e-003):6.938246e-003):2.501479e-003,(7:1.000535e-002,37:4.684358e-003):2.425237e-003):2.372228e-003,23:8.595334e-004):6.227176e-004):4.650627e-003,2:2.842779e-003):2.214136e-003,(((35:5.643775e-003,(36:8.248288e-004,((21:1.184218e-004,(26:4.216090e-003,(25:6.249243e-003,24:1.368332e-003):7.928239e-003):4.044028e-003):3.970727e-003,20:1.429001e-003):6.025666e-003):1.075041e-003):4.539928e-003,(27:7.812913e-003,31:6.201880e-003):8.679488e-004):2.879976e-003,30:3.315055e-003):2.574644e-003):1.444697e-004,22:3.788212e-003):1.237306e-002):5.047492e-003,18:9.506185e-003):1.692247e-003,(((8:1.905568e-003,(14:6.584569e-003,((15:5.170948e-003,3:1.204926e-003):5.132322e-003,((11:2.637231e-003,12:3.465214e-004):4.756397e-003,13:5.237603e-004):1.964374e-003):4.590424e-003):5.210269e-004):2.964500e-003,10:1.516009e-003):1.879419e-003,9:2.195035e-003):9.479182e-003):7.192384e-003,((4:2.419543e-003,40:7.474283e-003):3.217794e-003,5:5.737047e-004):3.525344e-003):1.575573e-002):7.548417e-003,1:1.097179e-002);

tree gen.51000 = [&U] ((((((41:3.041229e-002,42:3.940060e-003):2.330646e-002,(((30:3.310523e-003,27:1.038265e-002):1.474653e-003,(((((32:6.850089e-003,(34:8.936298e-003,((28:3.265540e-003,29:3.709904e-003):4.330041e-003,33:6.730425e-003):5.047610e-003):7.856492e-004):2.652874e-003,(7:7.712477e-003,37:3.279819e-003):1.876452e-003):6.551415e-003,23:1.256679e-003):7.227747e-003,(2:1.228852e-003,(38:8.120277e-003,39:4.874710e-003):2.011618e-003):4.124263e-005):7.523513e-003,22:5.139261e-003):2.529412e-004):2.113833e-004,((35:8.175225e-003,(36:1.158118e-003,((20:3.490147e-004,(21:2.091628e-003,(25:1.438393e-003,24:1.541984e-004):3.441312e-003):6.720631e-004):2.790512e-004,26:3.435641e-003):4.068399e-003):1.677847e-003):1.669472e-003,31:5.098014e-003):1.058168e-004):7.766008e-003):1.318462e-002,18:3.198198e-003):6.200916e-003,((8:1.650085e-003,(((3:6.912308e-003,(14:2.127744e-003,15:4.790201e-003):2.258133e-004):1.510326e-003,((11:5.608532e-003,12:2.407652e-003):4.330768e-003,13:4.425064e-003):2.470146e-003):2.677474e-003,10:2.185378e-003):4.322226e-003):3.546849e-003,9:1.817609e-003):2.649916e-003):2.877902e-003,((40:1.665060e-002,4:8.009728e-003):1.164720e-003,5:4.630710e-003):7.947254e-003):1.354469e-002,(17:1.400887e-002,((6:4.363951e-003,19:3.984393e-003):5.862901e-004,16:1.954644e-003):8.613758e-003):3.437739e-003,1:7.018120e-003);

tree gen.52000 = [&U] ((6:3.487072e-003,16:3.617118e-003):2.172076e-003,((19:3.249928e-003,(((((41:2.641704e-002,42:1.154167e-002):1.563528e-002,(((31:2.719731e-003,(((((32:7.114619e-003,34:5.378644e-003):3.416673e-003,(29:3.606788e-003,(28:4.492162e-003,33:4.644660e-004):4.811046e-004):3.935844e-003):2.580520e-003,(7:1.319373e-002,37:9.570302e-004):4.862667e-004):2.446765e-003,23:2.466942e-003):1.137727e-003,((38:7.053520e-003,39:3.114225e-003):4.881355e-003,2:4.485956e-003):4.220248e-004):8.079027e-003):1.624444e-003,22:5.513792e-003):2.470839e-003,((35:7.572023e-003,(36:4.108228e-003,((20:4.034845e-003,(21:4.424610e-004,26:6.710619e-003):4.232847e-004):2.002057e-003,(25:3.219232e-004,24:2.843147e-003):9.645358e-004):1.994570e-003):1.764763e-003):4.532933e-003,(30:2.431325e-003,27:8.682200e-003):4.628978e-003):1.962610e-004):1.024811e-002):5.650539e-003,18:2.778052e-003):5.386304e-003,((8:1.140620e-003,(((11:4.923797e-003,12:6.087646e-005):2.693672e-003,13:6.582711e-004):1.760847e-003,(10:2.224501e-003,((3:1.925504e-003,14:4.466790e-004):7.182418e-004,15:5.469195e-004):5.255232e-004):1.962063e-003):9.629719e-003):1.728908e-003,9:1.047678e-003):2.602232e-003):1.760061e-003,((4:5.527209e-003,40:1.878163e-002):3.044608e-004,5:3.980708e-003):6.639211e-003):9.547005e-003):5.292403e-004,17:4.243904e-003):8.852718e-005,1:8.878990e-003);

tree gen.53000 = [&U] ((17:4.241248e-003,19:1.550546e-003):5.733204e-004,((6:4.371494e-003,16:3.635472e-003):1.768539e-003,(((((41:1.669639e-002,42:3.859885e-003):2.269203e-002,((((31:2.733531e-003,(35:6.637957e-003,(36:4.129073e-003,(26:4.488150e-003,(21:2.339856e-003,(20:4.257598e-003,(25:3.210826e-004,24:2.104840e-003):8.554781e-004):2.899716e-004):4.164163e-004):1.464687e-003):1.672545e-003):1.035592e-003):6.002774e-003,27:8.585975e-003):7.273938e-004,((38:6.612408e-003,39:3.168531e-003):2.895218e-003,(2:4.508718e-003,(((34:3.688993e-003,(32:7.150719e-003,(33:5.319430e-004,(29:3.976491e-003,28:1.458323e-003):5.954731e-004):1.723147e-003):3.968789e-004):2.853849e-003,(37:1.134783e-002,7:1.168701e-002):1.184256e-002):4.026969e-003,23:3.978247e-004):5.819589e-003):6.609309e-004):8.176462e-003):1.672655e-003,(30:2.421978e-003,22:5.086066e-003):1.306046e-003):6.624222e-003):2.961982e-003,18:4.551613e-003):1.785533e-003,(((14:1.081959e-003,(3:4.870802e-004,15:7.110322e-004):1.532893e-002):4.894660e-003,((((11:3.196651e-003,12:3.623490e-004):4.107010e-003,13:2.988426e-003):4.659161e-004,10:2.368643e-003):2.331768e-003,8:2.094436e-003):5.221119e-005):8.279008e-004,9:1.265878e-003):2.507868e-003):3.051455e-003,(5:9.414399e-004,(40:1.884900e-002,4:2.174655e-003):1.547464e-003):2.669699e-003):3.330084e-003):1.212463e-003,1:6.355350e-003);

tree gen.54000 = [&U] ((17:1.142712e-003,((((((41:2.392378e-002,42:5.456975e-003):1.037397e-002,(((22:1.515545e-003,(((((38:6.265589e-003,39:8.462677e-004):4.048984e-003,(((32:4.939384e-003,((29:2.653346e-003,(33:1.439286e-003,28:4.062379e-003):1.284406e-003):1.573629e-003,34:2.042076e-003):1.704729e-003):7.195526e-004,(37:1.340725e-004,7:4.623542e-003):6.535275e-003):2.984920e-004,23:3.333490e-004):1.443491e-003):2.147904e-004,2:2.657591e-003):1.014697e-002,(35:6.619075e-003,(36:2.622203e-003,((21:1.739225e-003,26:1.510131e-003):6.420311e-004,(20:1.140278e-003,(25:5.764331e-006,24:7.801817e-004):4.083094e-004):8.933486e-004):2.638402e-003):3.031429e-003):1.902839e-003):1.449145e-003,31:7.560195e-003):2.276178e-003):1.249359e-003,27:3.865129e-003):2.642667e-003,30:3.314404e-003):1.001124e-002):3.178994e-003,18:5.386466e-003):1.858043e-003,(((10:2.930652e-003,(((11:9.271587e-004,12:2.312405e-003):4.003456e-003,13:6.537990e-004):1.174692e-003,((3:3.522948e-003,15:7.641372e-004):1.883385e-003,14:1.323030e-003):2.161626e-003):6.068637e-003):1.641102e-003,8:4.540448e-004):2.597231e-003,9:9.274588e-004):4.506564e-003):6.025846e-003,((40:1.727533e-002,4:2.397345e-003):1.108710e-003,5:3.652356e-004):9.204903e-003):6.446576e-003,(6:3.755932e-003,16:3.162455e-003):1.533320e-003):2.506418e-003):6.541889e-003,19:2.290074e-003,1:4.552374e-003);

tree gen.55000 = [&U] ((16:1.891993e-004,6:2.863117e-003):2.196657e-003,((((((41:2.232777e-002,42:5.733870e-003):1.127500e-002,(((30:4.154367e-003,22:1.590327e-003):5.466019e-004,27:4.050979e-003):1.444778e-003,(((((((28:7.741796e-004,(33:5.050436e-003,29:4.384605e-004):5.497477e-005):2.932446e-003,(32:4.465512e-003,34:4.098447e-003):3.159607e-004):1.912482e-003,(37:7.910496e-004,7:7.940385e-003):5.400836e-003):1.010701e-003,23:3.555154e-004):2.263255e-003,2:2.456924e-003):7.052076e-005,(38:5.573325e-003,39:8.616684e-004):3.366264e-003):8.676257e-003,(31:2.586459e-003,(35:6.739531e-003,(36:2.718533e-003,(20:7.388880e-004,(((25:3.478726e-004,24:6.344926e-004):9.085313e-004,21:9.735008e-004):1.765472e-003,26:9.258506e-004):6.643991e-004):5.534465e-003):9.153711e-004):3.217965e-004):1.272811e-003):1.315985e-004):1.029548e-002):2.634719e-003,18:1.306458e-003):4.436369e-003,((((10:5.510870e-003,(14:3.144509e-005,(15:1.844432e-003,3:7.299757e-004):1.918249e-004):1.345952e-004):8.529253e-003,8:1.028639e-004):1.911651e-003,((11:1.379358e-003,12:1.615695e-003):6.733988e-003,13:2.689362e-004):2.073152e-003):2.630515e-003,9:1.619975e-003):4.503730e-003):5.135692e-003,((40:1.177452e-002,4:2.606934e-003):1.086224e-003,5:1.135851e-003):8.047267e-003):5.362914e-003,(19:3.427401e-003,17:2.696178e-003):2.077341e-003):1.328203e-003,1:4.721894e-003);

tree gen.56000 = [&U] ((((((41:2.912102e-002,42:7.478409e-003):1.533395e-002,(((31:1.134732e-002,22:4.055202e-004):4.485426e-004,(27:5.340061e-003,(30:4.293033e-003,(((((((29:3.818757e-003,(33:7.738891e-003,28:1.457989e-003):3.049783e-004):1.704482e-003,32:5.824151e-003):1.373963e-004,34:5.397751e-003):3.602776e-003,(37:7.299132e-004,7:7.737305e-003):1.558766e-003):3.626622e-003,23:6.894368e-004):1.189513e-003,(38:7.116464e-003,39:1.678008e-002):1.147461e-003):5.364138e-004,2:1.010546e-003):3.923138e-003):1.803477e-003):1.348594e-003):3.918560e-004,(35:3.771971e-003,(36:1.326524e-003,((25:2.592734e-004,24:1.189623e-004):9.870014e-004,(20:3.475087e-004,(21:4.065250e-003,26:4.300358e-003):9.487732e-004):2.451070e-003):3.584646e-003):2.375050e-003):3.743764e-003):1.002082e-002):3.385723e-003,18:1.691683e-003):2.917444e-003,((8:1.529952e-004,((10:7.187561e-003,((15:2.913026e-003,14:1.136831e-003):5.594668e-004,3:9.520719e-004):7.121104e-003):2.634870e-004,((11:1.799030e-003,12:1.841676e-003):9.332307e-003,13:6.350892e-004):1.328579e-003):7.188910e-003):1.445639e-003,9:2.236924e-004):5.873996e-003):4.390046e-003,((4:3.400097e-003,40:7.170329e-003):1.518793e-003,5:3.015669e-004):3.260153e-003):1.553825e-002,(6:3.271555e-003,((19:8.067433e-003,17:8.055170e-003):2.335003e-003,16:1.727037e-003):4.312391e-004):1.304018e-003,1:3.015261e-003);

tree gen.57000 = [&U] (((((((41:2.727278e-002,42:6.812240e-003):1.500625e-002,((22:1.287368e-003,(((30:2.641135e-003,31:1.062713e-002):1.138871e-004,((((32:1.016390e-002,(34:1.397138e-002,(28:1.848886e-003,(29:3.576389e-003,33:7.119244e-003):1.656063e-004):1.233598e-003):9.996869e-004):1.746850e-003,(37:1.408879e-003,7:7.246236e-003):8.608481e-004):3.589265e-003,23:5.707629e-005):2.982914e-003,(2:4.696127e-003,(39:2.441640e-003,38:3.554769e-003):5.304747e-003):1.904228e-004):7.597968e-003):2.234491e-003,27:4.926905e-003):6.112764e-004):5.790034e-004,(35:3.962144e-003,(36:3.836004e-003,(((21:4.952250e-004,(25:3.372348e-004,24:2.106624e-003):5.492477e-003):2.030179e-004,20:2.535078e-003):1.135333e-003,26:1.362062e-003):4.967565e-003):1.020454e-002):3.817879e-003):9.686442e-003):2.418992e-003,18:2.040063e-003):6.766188e-004,(((((3:1.951423e-003,15:1.053282e-003):3.235879e-003,14:1.064679e-003):5.899553e-003,10:3.816755e-003):2.362126e-003,(8:5.645300e-004,((11:1.532284e-003,12:4.921053e-004):1.171944e-002,13:4.873075e-005):1.822263e-003):3.076606e-004):3.897959e-003,9:1.075351e-004):7.210685e-003):3.995465e-003,((40:6.219645e-003,5:1.416232e-004):1.032104e-003,4:3.184301e-003):2.355571e-003):6.310346e-003,(6:6.831169e-003,17:8.800100e-003):2.956507e-003):1.244493e-003,(16:6.432209e-003,19:7.555413e-003):3.388355e-004,1:5.324522e-003);

tree gen.58000 = [&U] ((((((((41:2.058958e-002,42:5.836498e-003):5.041924e-003,((((((32:7.803139e-003,((28:3.575672e-003,29:2.331205e-003):1.030079e-004,33:1.283264e-003):1.359912e-003):1.622181e-004,34:8.279618e-003):1.600565e-003,(37:2.476516e-003,7:3.926967e-003):3.863964e-003):3.804012e-003,23:1.325011e-004):2.091374e-003,((39:4.741704e-003,38:1.578052e-002):3.522434e-003,2:2.061695e-003):5.482885e-004):3.812180e-003,(27:3.910811e-003,(31:2.710884e-003,(((35:4.655348e-003,(36:4.311342e-003,(20:1.034407e-003,((25:6.243171e-004,24:2.496763e-004):1.476933e-003,(26:7.083263e-003,21:5.598886e-005):3.410869e-004):4.931611e-004):4.838953e-003):2.285648e-003):7.430982e-004,22:4.707483e-004):3.487663e-004,30:1.836234e-003):3.981116e-003):2.887003e-003):9.483128e-004):8.527980e-003):6.055336e-004,18:4.919002e-003):1.539352e-003,((8:6.018115e-003,(10:3.270067e-003,(((14:3.868936e-004,3:5.733922e-003):8.349564e-004,15:1.489112e-003):4.460333e-003,((11:2.569696e-003,12:3.880450e-003):1.204566e-002,13:3.345763e-003):3.692631e-003):2.685353e-003):7.141907e-003):3.005306e-003,9:2.192239e-003):2.111568e-003):2.870581e-003,((40:6.276903e-003,5:1.418058e-003):2.268940e-003,4:1.485464e-003):3.183989e-003):1.088635e-002,(6:4.401745e-003,16:9.581383e-004):1.582257e-003):1.754364e-005,19:3.972960e-003):8.618836e-004,17:6.766264e-003,1:5.298567e-003);

tree gen.59000 = [&U] (17:8.566806e-003,((((((41:2.527515e-002,42:1.125527e-002):7.292283e-003,((((((2:4.600670e-003,(((34:4.180439e-003,(((33:2.207498e-003,29:2.792730e-003):1.518696e-003,28:2.761684e-003):2.566364e-003,32:1.000657e-002):2.791129e-003):1.831867e-003,(37:1.411955e-003,7:1.030636e-002):3.438624e-003):3.510162e-003,23:3.758018e-003):3.831002e-003):1.208484e-003,(39:5.506672e-003,38:4.294078e-003):4.327115e-003):3.327321e-003,30:2.185345e-003):4.438730e-003,27:3.756750e-003):6.345252e-004,22:1.465033e-003):9.399562e-004,((35:5.714767e-003,(36:2.655696e-003,((26:2.780961e-003,((25:7.663932e-004,24:4.516077e-005):5.594701e-004,20:1.798143e-003):1.431647e-003):3.868545e-004,21:4.413203e-003):1.553176e-003):4.067768e-003):3.936849e-004,31:2.707865e-003):1.034517e-003):1.322567e-002):4.478834e-003,18:5.574121e-003):1.836359e-003,((10:8.947039e-003,((((14:6.927348e-004,3:2.096340e-003):3.732668e-005,15:1.191078e-003):3.862473e-003,((11:3.154482e-003,12:7.888999e-004):1.420579e-002,13:2.257166e-003):6.061056e-003):4.598375e-003,8:1.963066e-003):3.255764e-003):2.220283e-003,9:2.906668e-003):9.325596e-003):5.087804e-003,((40:1.702168e-002,5:7.607123e-005):2.377376e-003,4:1.108881e-003):7.246631e-003):7.111049e-003,((6:5.403451e-003,16:1.143333e-003):1.572756e-003,19:7.595052e-003):9.274662e-004):1.905362e-003,1:4.134694e-003);

tree gen.60000 = [&U] ((((((41:1.715453e-002,42:8.256269e-003):2.052358e-002,((((((((33:2.252224e-003,29:2.770682e-003):5.377499e-003,28:1.115155e-002):3.181343e-003,34:5.957155e-003):9.081496e-004,32:1.062296e-002):1.868982e-003,(37:3.983434e-004,7:1.041544e-002):1.692960e-003):1.948257e-004,23:6.217443e-004):2.123358e-003,(2:5.345612e-004,(39:5.973600e-003,38:6.489299e-003):1.356824e-003):1.979031e-003):3.832615e-003,((30:3.139575e-003,22:2.051633e-003):1.293596e-003,((31:4.945231e-003,(35:6.007883e-003,(36:2.315652e-003,((26:2.715300e-003,(21:8.303167e-004,(25:1.737507e-003,24:7.829196e-005):2.577129e-004):6.545451e-004):5.333858e-003,20:8.024819e-004):3.034041e-003):6.433865e-003):8.753738e-003):1.222556e-004,27:3.958714e-003):3.719002e-004):7.308590e-004):1.117385e-002):1.748798e-002,18:4.878529e-003):3.006856e-003,((8:5.928452e-004,(((11:6.455028e-003,12:6.637236e-004):5.863964e-003,13:2.359040e-004):1.103807e-003,((14:3.737205e-003,(15:1.322832e-003,3:3.734465e-003):1.741087e-003):2.540076e-003,10:9.538918e-003):6.075431e-004):4.307041e-003):4.278863e-003,9:1.841514e-003):5.450853e-003):2.559571e-003,((4:1.969091e-003,5:1.337318e-003):6.962038e-004,40:1.763023e-002):3.805267e-003):7.336991e-003,((16:4.951280e-003,6:2.155328e-003):3.004185e-003,(19:1.179714e-003,17:5.772501e-003):6.066916e-004):1.378874e-003,1:7.691230e-003);

tree gen.61000 = [&U] ((17:4.581938e-003,19:2.965551e-003):6.052374e-003,((6:4.458076e-003,16:4.291307e-003):2.180800e-003,(((((41:3.516311e-002,42:7.843650e-003):1.567346e-002,(30:1.655049e-003,(((31:4.484678e-003,22:1.183430e-003):4.335668e-004,((39:6.114262e-003,38:5.979735e-003):3.753092e-003,(2:1.855552e-003,(((((33:7.213625e-003,28:3.833148e-003):3.724256e-005,29:4.625811e-003):3.009633e-003,(34:2.653730e-003,32:9.710251e-003):5.498518e-003):7.251915e-004,(37:7.979000e-004,7:9.894910e-003):1.726937e-003):3.503432e-003,23:2.888968e-003):1.687934e-003):4.664327e-005):2.057748e-003):4.005213e-004,(27:3.230271e-003,(35:8.780122e-003,(36:1.144769e-002,((26:2.579599e-003,((24:9.755245e-004,21:3.816285e-003):8.794025e-004,25:1.403022e-003):4.529543e-003):1.076568e-005,20:6.273966e-003):6.629866e-003):3.135775e-003):1.445982e-003):7.647741e-004):1.081826e-003):2.026254e-002):7.172492e-003,18:4.829725e-003):9.828284e-004,(8:2.329129e-004,(10:8.688883e-003,((((15:1.255964e-003,3:1.091588e-004):1.839473e-003,14:2.244443e-004):3.974981e-003,((11:6.132428e-003,12:2.392987e-004):5.570903e-003,13:2.218366e-004):4.116632e-003):3.435693e-003,9:1.733006e-003):1.004525e-002):4.287511e-003):1.306016e-002):1.034187e-002,((5:1.089864e-003,40:1.879309e-002):2.167935e-003,4:2.277348e-003):1.661216e-003):1.065168e-002):3.564047e-004,1:6.059948e-003);

tree gen.62000 = [&U] (((((((41:4.234414e-002,42:9.445483e-003):1.887430e-002,((30:4.233525e-003,((27:4.469262e-003,(((39:1.739271e-003,38:7.039754e-003):5.564530e-003,(2:1.361935e-003,((((((29:1.051949e-002,28:4.648077e-003):1.024833e-003,33:1.488068e-003):9.508125e-003,32:1.098096e-002):8.638682e-004,34:4.339152e-003):4.474552e-003,(37:3.705353e-003,7:9.289739e-003):5.373633e-003):1.982408e-003,23:3.478954e-003):4.487159e-003):8.478953e-004):1.539300e-003,22:1.044478e-003):8.117222e-004):8.986553e-004,31:1.468886e-002):3.118227e-003):1.154061e-003,(35:1.494911e-002,(36:2.611435e-003,(((24:9.026877e-004,25:1.653370e-003):2.072264e-003,26:7.893705e-003):5.346389e-004,(20:2.302930e-003,21:5.309687e-003):6.358605e-003):1.199621e-002):4.505870e-003):6.389355e-003):1.721002e-002):8.639873e-003,18:4.093297e-003):5.710061e-003,(((((10:2.636134e-003,3:1.076480e-003):5.401151e-005,(15:8.848427e-004,14:1.290070e-004):9.016012e-004):9.201525e-003,9:3.559924e-003):3.469117e-004,((11:4.387834e-003,12:1.942206e-004):1.146216e-002,13:1.549830e-004):6.294740e-004):1.253851e-003,8:2.445243e-004):1.690365e-002):1.072138e-002,(40:2.196950e-002,(5:3.213475e-003,4:2.802365e-003):1.783782e-003):1.614821e-003):9.805577e-003,((6:4.944791e-003,16:2.647458e-003):5.908580e-003,17:5.503030e-003):1.528094e-003):1.829596e-003,19:3.155745e-003,1:6.662976e-003);

tree gen.63000 = [&U] ((16:2.095397e-003,6:2.832409e-003):4.326721e-003,((((((41:2.772495e-002,42:5.585144e-003):2.055986e-002,(22:1.822320e-003,((27:6.550425e-003,(((35:6.890845e-003,(36:7.459189e-003,(((20:1.035870e-003,21:1.549246e-003):9.696905e-004,(24:1.967594e-003,25:7.202162e-004):5.341399e-003):2.647626e-003,26:6.036055e-003):2.468198e-003):3.997532e-003):1.689874e-003,((2:8.973760e-004,(39:1.436356e-003,38:7.400204e-003):3.819050e-003):9.780429e-004,(((34:2.672625e-003,((28:8.848484e-003,(29:3.146787e-003,33:1.216553e-003):9.314689e-004):2.087317e-003,32:5.421717e-003):1.095787e-003):2.481243e-003,(37:2.817086e-003,7:7.223101e-003):1.993341e-003):4.490746e-003,23:7.058008e-004):9.120388e-004):1.244025e-003):3.099312e-004,30:1.889523e-003):1.422593e-003):5.301091e-003,31:5.911932e-003):4.357532e-006):1.092072e-002):1.142804e-002,18:1.838699e-003):3.448187e-003,((8:2.449285e-004,(((9:5.858288e-003,(11:3.434174e-003,12:5.310049e-004):1.335561e-002):1.277274e-003,(13:4.349030e-003,(15:2.139181e-003,14:6.977393e-004):6.273968e-003):1.875410e-003):2.388218e-003,3:2.597162e-004):3.526513e-003):3.239902e-003,10:2.162246e-003):1.138060e-002):6.968558e-003,((5:2.920218e-005,4:1.049123e-003):1.796717e-003,40:1.279137e-002):9.451492e-003):6.913581e-003,(17:5.493638e-003,19:1.031508e-003):4.487245e-003):4.197179e-004,1:6.419338e-003);

tree gen.64000 = [&U] ((((((41:2.768812e-002,42:8.210154e-003):1.302453e-002,((2:7.845384e-004,((((32:4.624931e-003,((28:6.516639e-003,(29:5.646567e-003,33:6.535679e-003):1.705431e-004):9.866011e-003,34:2.557998e-003):1.490406e-004):3.649294e-003,(37:5.390141e-004,7:6.161580e-003):5.955985e-003):5.900611e-005,23:7.669257e-004):2.886724e-003,(39:9.188925e-004,38:6.312656e-003):3.372514e-003):4.791431e-004):1.684874e-002,((27:5.730681e-003,(30:1.667046e-003,31:5.127616e-003):3.297566e-003):6.263440e-005,((35:5.878153e-003,(36:2.284864e-003,((((24:3.869193e-003,25:6.796900e-004):5.285127e-004,20:1.693692e-003):5.615097e-003,21:3.621200e-005):6.569173e-004,26:5.221301e-003):5.573243e-003):7.166533e-003):4.525520e-004,22:1.239659e-003):3.437519e-005):4.255938e-004):1.140347e-002):3.216506e-003,18:1.568481e-003):2.948240e-003,((8:2.295833e-005,((3:3.142379e-003,(9:1.804896e-003,(15:6.850867e-004,14:1.385697e-003):3.186604e-003):5.919154e-004):1.085557e-003,(13:7.858714e-004,(11:2.319169e-003,12:4.529674e-004):1.139285e-002):4.963347e-003):3.928403e-003):1.525595e-003,10:1.761966e-003):8.932754e-003):6.391809e-003,((5:2.347277e-004,4:4.373418e-004):8.253216e-004,40:1.123144e-002):2.728055e-003):1.369958e-002,((16:5.355535e-003,6:6.579637e-003):3.569435e-003,(19:2.747420e-003,17:4.875997e-003):2.973749e-004):1.317222e-003,1:8.740723e-003);

tree gen.65000 = [&U] (17:6.640934e-003,((6:6.211826e-003,(16:3.663936e-003,19:2.335577e-003):2.252947e-003):4.501764e-004,(((18:5.574931e-003,((41:3.004879e-002,42:9.626360e-003):7.877748e-003,(((22:2.488408e-003,27:4.308948e-003):1.134001e-003,(((35:4.016965e-003,(36:6.138386e-003,((26:2.080243e-003,(20:1.904350e-003,(24:9.054488e-004,25:1.843528e-004):1.974402e-003):1.006998e-003):2.189045e-003,21:4.245837e-005):2.399131e-003):1.520573e-003):6.230764e-003,30:4.967621e-003):4.023658e-003,31:6.841965e-003):5.228739e-004):4.432365e-004,((39:8.508784e-003,38:7.542382e-003):6.067349e-004,((23:9.682124e-004,((((29:4.514093e-003,(28:2.033564e-003,33:5.812958e-003):1.867613e-003):4.934694e-003,32:5.776446e-003):2.247580e-003,34:2.999238e-003):9.004582e-004,(37:2.606024e-003,7:5.941808e-003):5.499383e-003):1.821542e-003):8.643749e-005,2:7.627268e-004):1.430247e-003):2.140030e-003):1.806561e-002):1.097637e-002):2.608907e-003,((13:9.214299e-004,(11:1.559875e-003,12:2.418602e-003):6.349764e-003):2.064967e-003,(((14:1.683580e-003,(15:5.323689e-004,3:1.822751e-003):1.730696e-004):2.946981e-003,9:3.548813e-004):4.032855e-003,(10:1.684022e-003,8:8.861044e-004):1.494407e-003):1.764355e-003):1.250736e-002):7.515565e-003,(40:5.574955e-003,(4:5.385032e-004,5:2.468417e-004):3.525596e-004):3.215055e-003):1.071423e-002):1.527562e-003,1:5.378559e-003);

tree gen.66000 = [&U] (((6:5.221139e-004,(19:4.503515e-003,16:3.208148e-003):1.197907e-003):2.925017e-003,17:4.648974e-003):6.481865e-004,(((18:7.161881e-003,((41:3.056712e-002,42:7.683242e-003):2.322857e-002,(((35:5.528651e-003,((36:2.911459e-003,(20:9.214852e-004,(((24:3.212775e-003,25:6.629908e-004):3.758331e-003,21:1.508314e-003):9.146012e-005,26:2.860027e-003):1.242780e-004):3.017062e-003):1.968099e-003,((22:1.850469e-003,27:4.049032e-003):1.406307e-003,31:7.889550e-003):3.037260e-004):5.345260e-003):2.999943e-003,30:5.478208e-003):1.016004e-003,((2:9.162793e-004,(39:3.606947e-003,38:8.767868e-003):1.094595e-002):6.542640e-004,(23:3.235310e-003,((((29:1.906270e-003,(33:7.043393e-003,28:4.753284e-003):4.316448e-003):3.043659e-003,32:3.500609e-003):7.927757e-004,34:5.568942e-003):2.299866e-003,(37:2.847853e-003,7:7.582723e-003):1.998743e-003):2.763774e-004):4.869292e-003):4.283515e-003):7.526807e-003):5.989037e-003):2.163905e-003,(((((15:7.666022e-004,14:1.331073e-003):1.217059e-003,3:6.384999e-004):3.365864e-003,10:2.267093e-003):3.378703e-003,(8:7.025078e-004,((11:9.149481e-005,12:3.826337e-007):9.596654e-003,13:1.282776e-003):8.890567e-004):8.732510e-004):2.170096e-004,9:4.172190e-003):9.922717e-003):8.913778e-003,((5:1.216823e-003,4:5.915622e-004):1.278261e-004,40:7.325835e-003):5.983897e-003):6.176390e-003,1:1.159434e-002);

tree gen.67000 = [&U] ((17:4.615356e-003,((6:2.976908e-003,16:4.556241e-003):3.201097e-003,19:6.985986e-003):1.014209e-003):7.422219e-004,(((((41:1.643220e-002,42:8.471291e-003):1.646927e-002,((((31:6.207137e-003,22:1.512534e-003):1.051574e-005,30:7.669088e-004):1.938570e-003,((35:5.729068e-003,(36:4.914615e-003,(((24:1.983599e-003,25:7.157260e-004):3.641016e-003,26:6.857134e-003):5.113171e-004,(20:2.624987e-003,21:2.830327e-004):4.014041e-003):2.864946e-003):3.732038e-003):3.781907e-003,((2:2.028027e-003,(39:3.425089e-003,38:9.315750e-003):6.327405e-003):1.366886e-003,(23:5.825293e-005,((34:5.288163e-003,(((33:6.602877e-003,28:4.513629e-003):6.969111e-004,29:1.450498e-003):3.584596e-003,32:3.324112e-003):5.645711e-003):6.606972e-003,(37:6.581835e-004,7:7.200411e-003):1.882849e-003):6.606031e-004):4.444473e-003):4.405444e-003):1.085193e-003):3.565739e-004,27:7.237594e-003):1.671088e-002):2.573487e-003,18:2.528217e-003):2.983861e-003,((8:6.308039e-004,(10:3.398419e-003,(((11:1.607669e-003,12:7.676435e-006):7.260669e-003,13:1.932669e-003):3.600712e-003,((15:7.134554e-004,14:1.246916e-003):2.060504e-003,3:7.850161e-004):3.451487e-003):1.395818e-003):2.571768e-003):2.177497e-003,9:2.616935e-003):1.050298e-002):7.840011e-003,((5:1.300933e-003,40:1.429239e-002):9.512075e-004,4:2.657497e-003):1.009393e-002):5.471632e-003,1:1.450617e-002);

tree gen.68000 = [&U] ((6:3.246506e-004,16:1.425739e-003):2.205624e-003,((((((41:1.213170e-002,42:9.144110e-004):1.215907e-002,((35:4.037779e-003,(36:7.883984e-003,(26:4.833935e-003,(21:2.087181e-004,((24:1.753958e-003,25:8.736226e-004):2.321948e-003,20:2.132255e-003):7.202668e-004):9.339076e-004):2.942949e-003):3.623260e-003):2.004398e-003,((((30:2.428035e-003,(2:2.179871e-003,((23:6.188246e-004,((34:3.750946e-003,((28:3.542232e-003,(29:5.585027e-004,33:4.874828e-003):3.136807e-003):2.811799e-003,32:2.533021e-003):2.105000e-003):4.521442e-003,(37:1.777189e-005,7:6.023397e-003):1.177255e-003):3.435653e-004):1.602823e-003,(39:4.437392e-003,38:6.877711e-003):3.900601e-003):1.022153e-003):8.578247e-003):2.290991e-004,27:4.641434e-003):2.659229e-004,31:4.488563e-003):9.001415e-005,22:2.083645e-003):1.072169e-003):1.342846e-002):2.543816e-003,18:1.791218e-003):2.930515e-003,((8:1.436597e-003,(((11:1.186924e-003,12:3.704641e-005):6.739303e-003,13:1.426867e-003):1.581041e-003,((14:4.465617e-004,15:1.828397e-003):2.071502e-003,(10:2.509013e-003,3:2.630524e-003):1.547451e-003):1.029053e-003):4.889795e-003):2.590534e-003,9:1.932053e-003):7.857356e-003):7.107026e-003,(40:1.055191e-002,(4:4.884465e-004,5:1.031363e-003):1.069890e-003):1.681212e-003):7.990880e-003,(17:2.818540e-003,19:8.742712e-004):2.360675e-004):2.112851e-003,1:1.106029e-002);

tree gen.69000 = [&U] ((6:1.607628e-003,16:1.769978e-003):1.758690e-003,(17:3.802754e-003,((((((41:4.504678e-002,42:9.404741e-003):1.337550e-002,(((((2:9.631184e-003,(((23:1.574968e-004,((37:1.135941e-004,7:5.040486e-003):1.122363e-002,((34:5.555378e-003,((28:4.654014e-003,33:6.084196e-003):1.661901e-004,29:2.063157e-003):5.065777e-003):9.743764e-004,32:3.219676e-003):6.257493e-003):2.260178e-003):2.129778e-004,39:6.717228e-003):8.554293e-004,38:8.333173e-003):2.478083e-003):4.079545e-003,31:5.743048e-003):1.319698e-003,(35:4.641520e-003,(36:3.797240e-003,((((24:2.467048e-003,25:6.978404e-004):1.012631e-003,20:1.875592e-004):1.972643e-003,26:1.619507e-003):4.809654e-004,21:8.742565e-004):6.879367e-003):3.534540e-003):2.272285e-003):1.127481e-003,27:9.160250e-003):1.775947e-004,(30:1.730328e-003,22:8.867120e-003):7.450796e-004):1.351069e-002):6.718864e-003,18:1.333468e-002):1.740684e-003,((8:3.975909e-004,((3:6.676804e-003,((14:3.670360e-003,15:2.097320e-004):6.953158e-003,((11:1.526044e-003,12:4.488617e-005):8.683773e-003,13:1.728821e-003):3.901110e-003):4.775934e-005):5.173051e-003,10:4.858020e-003):2.919932e-003):1.383494e-003,9:1.985782e-003):3.464526e-003):4.920374e-003,(40:1.219729e-002,(5:1.293725e-003,4:5.180809e-003):9.412690e-004):8.344183e-003):5.304504e-003,19:1.037389e-003):1.519291e-003):2.403588e-004,1:1.563471e-002);

tree gen.70000 = [&U] (((17:5.545157e-003,(((((41:5.236628e-002,42:5.039242e-003):1.305004e-002,((((((2:9.818858e-004,(23:3.172183e-004,((37:4.539484e-003,7:7.393643e-003):2.429370e-003,((29:3.157757e-003,(33:7.465737e-003,28:1.718011e-003):1.754687e-003):2.321454e-003,(32:2.898731e-003,34:2.776376e-003):1.697745e-003):2.039324e-003):3.852875e-003):1.759621e-003):2.861340e-003,(38:1.728530e-002,39:7.966373e-003):1.013466e-003):3.897071e-003,22:6.601918e-003):1.432071e-003,(35:6.369188e-003,(36:7.286712e-003,((20:1.503591e-003,21:9.871641e-004):4.271441e-004,((24:9.363555e-004,25:1.864196e-003):2.888583e-003,26:5.311855e-003):1.389488e-003):8.317129e-003):1.609037e-002):2.765744e-003):8.361924e-003,(30:1.165867e-002,27:7.673109e-003):2.796179e-003):5.341761e-004,31:4.392564e-003):2.564081e-002):6.567324e-003,18:3.867277e-003):2.573301e-003,((((13:2.780811e-003,((10:6.562449e-003,(14:9.918102e-004,15:1.833188e-003):3.742045e-003):2.882670e-003,3:4.608280e-003):3.205935e-003):2.159593e-003,(11:1.042772e-002,12:1.690377e-004):3.702623e-003):2.437689e-003,8:4.021812e-004):1.448033e-003,9:1.153737e-003):6.913930e-003):6.131088e-003,(40:1.417919e-002,(5:1.241570e-003,4:7.664097e-003):4.258604e-004):2.051430e-003):1.155103e-002):1.056593e-006,19:6.863953e-003):2.491464e-003,(6:1.935315e-003,16:2.528164e-003):3.534646e-003,1:3.573858e-003);

tree gen.71000 = [&U] (((((((41:3.014148e-002,42:4.488252e-003):1.075926e-002,(22:1.320184e-003,(((38:1.811774e-002,39:4.465804e-003):5.029855e-003,(2:2.128146e-003,(23:8.069452e-004,((37:2.802298e-003,7:6.585223e-003):1.912564e-003,(34:3.302997e-003,(32:3.724332e-003,(33:2.324952e-003,(29:4.850980e-003,28:1.530164e-003):1.450549e-004):4.503005e-003):1.588523e-003):1.344884e-003):2.239700e-003):7.326978e-003):2.752341e-004):2.218780e-003,(30:1.112661e-002,(((35:4.147588e-003,(36:6.141285e-003,((((24:4.194776e-004,25:6.871138e-003):6.619458e-004,26:7.787082e-003):2.602089e-004,21:1.887529e-003):2.394487e-003,20:2.736490e-003):8.672621e-003):1.634479e-002):2.110911e-003,31:7.329722e-003):1.540866e-004,27:1.214695e-002):1.592151e-003):3.003315e-004):3.139783e-003):2.012998e-002):5.655837e-003,18:5.593214e-003):3.935920e-003,((8:1.223117e-003,((13:1.562857e-003,(11:2.154439e-003,12:7.941719e-004):1.102821e-002):3.365979e-004,(3:5.986758e-004,((15:2.486083e-003,14:4.514464e-004):3.274536e-003,10:3.878774e-003):1.480781e-004):4.832803e-003):6.309825e-003):2.781670e-003,9:1.027587e-003):8.953033e-003):6.907699e-003,(40:1.219763e-002,(5:5.728940e-003,4:4.023420e-003):2.645899e-003):5.163097e-003):1.417080e-002,17:4.591854e-003):1.155953e-004,(6:2.190605e-003,(19:4.697297e-003,16:1.631707e-003):2.982939e-004):9.019021e-004,1:9.186943e-003);

tree gen.72000 = [&U] (17:6.344451e-003,(((16:9.674133e-004,6:2.776901e-003):3.649561e-005,19:1.513699e-003):2.271071e-003,((4:4.939967e-003,(40:1.667841e-002,5:3.721619e-003):1.035513e-003):3.450708e-004,((((41:3.809682e-002,42:5.574798e-003):1.606438e-002,(((22:1.078570e-002,(((23:1.834293e-003,((37:1.926726e-003,7:5.937775e-003):3.584572e-003,(32:6.396751e-003,(34:6.540682e-003,((28:6.018278e-003,33:5.648889e-003):8.128513e-004,29:5.738284e-003):1.021164e-002):3.253915e-004):3.761577e-003):8.053185e-003):3.572878e-003,((38:3.022685e-003,39:5.428068e-003):5.235638e-003,2:2.836192e-003):1.195435e-004):3.167549e-003,((35:2.103432e-002,(36:3.927836e-003,((20:4.281163e-003,26:7.134726e-003):4.672686e-003,(21:1.084742e-003,(24:2.543977e-003,25:1.152139e-003):1.542541e-003):1.731304e-004):9.234390e-003):1.047868e-002):1.404612e-003,31:1.012306e-002):8.787421e-004):1.643051e-003):5.925267e-004,30:4.671316e-004):6.493680e-004,27:7.388802e-003):1.078447e-002):2.567796e-003,18:7.454108e-003):9.291567e-003,(((((15:3.205744e-003,3:4.129343e-003):8.243982e-004,14:8.302083e-004):5.850446e-003,10:5.169264e-003):4.472777e-003,((13:1.007841e-003,(11:2.871234e-003,12:1.058397e-003):8.011847e-003):2.034252e-003,8:1.046662e-003):1.059297e-003):2.249875e-003,9:2.396724e-004):8.682663e-003):7.702034e-003):2.503939e-002):2.557770e-004,1:1.052372e-002);

tree gen.73000 = [&U] ((((16:4.830498e-004,19:1.541881e-003):2.654983e-004,6:7.505593e-003):1.047620e-003,17:5.998376e-003):3.906870e-003,((5:2.732423e-004,(40:1.995253e-002,4:2.198631e-003):4.962380e-004):5.006836e-003,((((41:4.557555e-002,42:7.559831e-003):1.921796e-002,(((2:4.862755e-003,(38:1.627383e-002,39:2.186795e-003):2.186123e-003):5.498196e-003,(23:4.653293e-003,((37:8.562408e-005,7:6.934785e-003):3.676528e-003,((32:9.711585e-003,(29:6.518880e-003,(28:5.868399e-003,33:7.031390e-003):8.910298e-004):9.812574e-003):5.203614e-004,34:7.824675e-003):4.614083e-003):4.579175e-003):4.798695e-003):6.991241e-003,((30:4.056297e-003,31:8.866246e-003):4.388704e-004,(27:6.980689e-003,(22:9.952840e-004,((36:1.825173e-003,((26:8.067327e-003,21:4.273577e-004):2.486868e-003,(20:1.337434e-003,(24:9.975296e-004,25:2.420152e-004):2.255369e-003):1.510213e-004):4.962067e-003):3.951873e-003,35:2.550851e-002):4.614609e-003):3.780269e-003):1.032286e-003):6.287329e-004):1.056928e-002):5.200717e-003,18:8.482392e-003):1.334861e-002,((((14:9.823593e-004,(3:1.185594e-003,15:5.557075e-005):8.012375e-003):1.779829e-002,(10:4.464996e-003,8:3.046497e-003):7.381037e-004):4.241167e-003,(13:1.674788e-003,(11:1.951274e-003,12:1.986863e-003):9.584642e-003):3.411018e-003):7.163925e-003,9:1.247274e-003):5.184479e-003):9.717150e-003):1.060375e-002,1:7.461463e-003);

tree gen.74000 = [&U] ((19:1.795192e-003,((6:8.691275e-003,16:1.304129e-003):2.808703e-003,(((4:5.661554e-003,5:1.449258e-004):7.279064e-004,40:1.335433e-002):1.998867e-003,((((41:4.905205e-002,42:1.110079e-002):1.936029e-002,((((30:3.284162e-003,((36:3.404818e-003,((21:3.991408e-004,(20:7.881710e-005,(24:5.373406e-004,25:2.604762e-004):2.209243e-003):5.461698e-004):1.132598e-003,26:1.004229e-002):7.570026e-003):5.791802e-003,(35:2.623484e-002,(((23:2.379339e-003,((37:2.359394e-003,7:7.463770e-003):8.245089e-003,((32:1.599167e-002,((28:5.165288e-003,33:1.641018e-002):4.809735e-003,29:9.081725e-003):7.356390e-003):5.351337e-004,34:6.126322e-003):5.354852e-003):6.352177e-003):2.076275e-003,(38:1.981727e-002,39:3.837610e-003):4.350956e-003):1.795925e-004,2:3.555870e-003):1.006079e-002):9.544642e-004):1.792818e-003):5.844315e-003,27:7.529075e-003):2.630612e-003,31:6.570918e-003):1.466096e-003,22:9.726348e-004):1.062518e-002):5.343618e-003,18:1.464144e-002):3.244184e-003,(((8:2.948705e-003,(10:4.302373e-003,(14:3.157886e-004,(15:5.928688e-004,3:3.160193e-003):2.724993e-003):3.494562e-003):2.154310e-002):1.013437e-003,(13:3.362774e-003,(11:2.205100e-003,12:4.912575e-003):1.138163e-002):8.210206e-003):3.196740e-003,9:1.342416e-003):2.141481e-002):1.409321e-002):7.139776e-003):4.702639e-003):1.522484e-004,17:8.470367e-003,1:4.638501e-003);

tree gen.75000 = [&U] (17:5.330801e-003,((((40:1.116709e-002,(5:1.524985e-003,4:4.110412e-003):1.359595e-003):8.886684e-003,(((10:8.112667e-003,((8:5.886383e-004,(14:1.026521e-002,(15:4.957656e-004,3:4.426676e-004):6.248069e-003):5.523741e-003):5.571987e-003,(13:3.141823e-003,(11:5.841473e-003,12:7.783450e-003):4.566415e-003):6.511183e-003):2.177491e-003):4.127381e-003,9:3.114252e-003):2.305348e-003,(18:3.752560e-003,((41:4.062052e-002,42:1.105416e-002):1.553594e-002,(27:9.732412e-003,((31:9.120381e-003,22:6.959428e-004):9.217003e-007,(30:7.243915e-004,(((36:8.039767e-003,(20:3.162251e-004,((26:8.190056e-003,21:1.914173e-004):1.309751e-003,(24:2.156108e-003,25:4.405762e-005):9.881633e-003):5.788864e-004):1.308084e-003):8.120478e-003,35:1.940885e-002):4.544650e-003,((2:2.511855e-003,(23:4.447649e-004,((37:2.048376e-003,7:6.241314e-003):1.216094e-003,((((29:7.319213e-004,33:1.751708e-003):7.110359e-004,28:5.550733e-003):3.224747e-003,34:4.818509e-003):1.155222e-003,32:1.103033e-002):5.226529e-003):2.556638e-003):4.139397e-003):1.316060e-003,(38:1.677022e-002,39:2.811485e-003):4.785522e-003):7.134096e-003):2.482192e-005):3.735606e-004):3.868797e-003):1.311132e-002):7.520767e-003):6.066558e-003):6.975201e-003):1.095294e-002,(16:6.002933e-003,6:7.588443e-003):2.307807e-003):1.008175e-002,19:5.820086e-003):1.116133e-005,1:8.630951e-003);

tree gen.76000 = [&U] (((40:8.673295e-003,(4:2.889877e-003,5:1.768741e-004):5.340807e-004):3.323117e-003,((10:5.544020e-003,((8:1.906353e-003,(((15:1.301247e-003,3:5.119570e-003):3.565471e-004,14:3.077442e-003):8.098764e-003,(13:2.650625e-003,(11:3.321660e-003,12:8.853978e-004):9.441887e-003):1.971042e-003):5.273767e-003):3.578079e-004,9:5.426409e-003):1.054911e-003):8.201137e-003,(18:7.510355e-003,((41:4.275015e-002,42:5.792487e-003):2.192177e-002,(((22:1.704978e-003,(30:1.419868e-003,((((38:1.210743e-002,39:4.081320e-003):3.708282e-003,(23:5.516344e-004,((37:2.028224e-003,7:7.909792e-003):1.124551e-003,(((29:1.136388e-003,(33:7.664479e-003,28:5.424435e-003):9.770909e-004):2.853710e-003,32:1.094066e-002):1.056635e-004,34:4.584224e-003):1.908832e-003):2.067007e-003):1.701954e-003):9.889973e-005,2:4.815606e-003):7.396351e-003,((36:7.974410e-003,(((21:3.924859e-004,20:1.059994e-006):1.527485e-003,26:7.894736e-003):3.038549e-004,(24:5.832812e-003,25:8.787276e-004):6.027127e-004):5.782977e-003):6.903765e-003,35:1.226227e-002):2.605309e-003):6.380181e-004):7.672488e-003):6.025926e-003,27:9.731220e-003):4.368935e-005,31:6.504384e-003):1.606676e-002):8.046171e-003):2.395682e-003):6.591360e-003):1.470664e-002,(((16:1.638556e-003,19:4.237722e-003):3.461026e-004,6:7.607042e-003):3.311737e-003,17:1.483075e-002):8.733148e-004,1:1.353583e-002);

tree gen.77000 = [&U] ((((5:2.627680e-003,(40:2.222605e-002,4:2.642664e-003):1.229415e-003):2.453510e-003,((((41:6.646889e-002,42:2.005680e-002):2.356838e-002,(((27:2.017031e-002,(((36:3.035047e-003,((26:1.227491e-002,21:1.534124e-003):4.252809e-003,(20:3.488964e-003,(24:4.346394e-004,25:1.366266e-003):3.007714e-003):2.098729e-003):2.795988e-003):1.271770e-002,35:1.306884e-002):8.958411e-003,31:9.536842e-003):3.111348e-005):4.650691e-003,22:6.199053e-003):4.058357e-004,(((39:9.609187e-003,(2:3.817051e-003,38:1.882491e-002):3.373908e-003):3.396351e-003,(23:8.549147e-004,((37:3.899417e-003,7:1.229832e-002):5.313828e-003,((((29:4.791231e-003,28:2.814344e-003):7.714529e-004,33:3.465689e-003):3.718239e-003,34:3.536472e-003):6.874020e-004,32:1.679234e-002):4.996977e-003):3.768417e-003):6.291611e-003):7.289666e-003,30:6.265114e-003):2.224509e-003):2.719351e-002):2.454464e-002,18:8.172503e-003):1.585829e-003,(9:6.237571e-003,(8:4.157179e-003,(((15:5.438588e-003,14:9.574892e-004):6.514258e-003,(3:2.348760e-003,(13:4.121252e-003,(11:4.174119e-003,12:2.389166e-003):8.869970e-003):1.089086e-002):6.090732e-003):5.671573e-003,10:4.722532e-003):5.342977e-003):4.110189e-003):1.062241e-002):1.936556e-002):1.813147e-002,17:2.206707e-002):9.091787e-004,(19:3.360376e-003,(6:1.274824e-003,16:1.021307e-003):1.102213e-002):7.498039e-004,1:6.401588e-003);

tree gen.78000 = [&U] (19:3.923498e-003,((17:1.404343e-002,(6:8.246685e-004,16:2.222032e-003):3.420293e-003):5.423292e-004,((40:1.047987e-002,(4:2.618184e-004,5:1.217110e-003):3.122431e-004):7.662898e-003,((((41:4.299795e-002,42:1.297451e-002):2.978091e-002,(((30:8.626690e-003,27:1.482154e-002):1.544488e-003,22:2.780786e-003):4.166159e-003,((((36:3.449884e-003,((26:7.908698e-003,(20:5.828173e-004,(24:9.965119e-004,25:2.127181e-003):3.274860e-003):8.294244e-004):2.153295e-003,21:9.038321e-003):5.644461e-003):1.699767e-003,35:8.454076e-003):8.601125e-004,((2:4.723875e-003,(23:3.040945e-004,((37:6.263225e-004,7:6.959042e-003):5.658334e-003,(34:3.657900e-003,(32:1.077227e-002,(28:1.829787e-003,(33:2.133090e-003,29:1.722834e-003):4.703247e-004):3.773955e-003):9.435869e-004):1.838828e-003):6.541993e-003):4.111093e-003):2.779680e-003,(38:9.769516e-003,39:3.840146e-003):6.745363e-003):8.215281e-003):1.498871e-003,31:1.026414e-002):3.029510e-003):1.037859e-002):4.436086e-003,18:3.237500e-003):1.888308e-003,(((10:6.813505e-003,((15:4.145703e-005,14:3.381182e-004):2.399505e-003,3:7.469845e-004):4.182743e-004):2.679853e-003,9:5.313808e-003):1.159106e-003,(((11:1.648571e-003,12:7.649461e-003):1.106623e-002,13:1.149035e-004):1.576205e-003,8:9.042899e-004):1.623350e-004):1.541025e-002):1.258686e-002):7.182906e-003):1.945063e-003,1:6.242408e-003);

tree gen.79000 = [&U] ((((4:7.489909e-003,40:8.986414e-003):6.459363e-004,5:2.358178e-003):3.056617e-003,((((41:3.351410e-002,42:1.318937e-002):1.369064e-002,((((27:1.445745e-002,30:8.455873e-003):3.544707e-003,((2:4.602251e-003,(23:3.111118e-004,((37:2.059476e-003,7:6.581649e-003):6.781793e-003,(32:1.474445e-002,(((29:1.965092e-003,28:2.720279e-003):2.418953e-004,33:2.109869e-003):6.116286e-003,34:4.020757e-003):5.107287e-004):1.578252e-003):9.942269e-004):1.215294e-003):1.843671e-003,(38:9.931305e-003,39:8.648762e-003):8.046548e-004):1.511466e-002):1.569367e-003,((36:2.253617e-003,(21:5.572117e-004,(((24:4.052713e-003,25:2.475401e-003):2.619893e-003,26:8.317373e-003):3.170664e-003,20:3.874999e-004):7.036144e-003):5.477274e-003):5.163298e-003,35:6.543491e-003):9.741992e-004):5.922304e-004,(22:5.632919e-003,31:8.615508e-003):1.085375e-004):1.279671e-002):1.232110e-002,18:2.399700e-003):2.303585e-003,(9:8.842358e-003,((10:6.851355e-003,8:1.511360e-003):8.888414e-004,(((11:5.207268e-003,12:2.484717e-004):1.365564e-002,13:4.434870e-003):7.535528e-004,(3:7.957432e-004,(15:2.624565e-003,14:1.070939e-003):8.623932e-004):1.061702e-002):5.375832e-003):2.532115e-003):1.873306e-002):1.231384e-002):1.437824e-002,((6:1.015478e-002,16:1.103630e-003):2.096763e-003,(19:8.121811e-003,17:1.331682e-002):3.448403e-003):9.458612e-004,1:2.456891e-002);

tree gen.80000 = [&U] ((17:1.999673e-002,(((4:7.310627e-003,5:5.027626e-003):6.136754e-003,40:1.554097e-002):3.214675e-003,((((41:3.410871e-002,42:1.342338e-002):1.971465e-002,((30:8.495913e-003,(((2:2.804814e-003,(23:2.797160e-003,((37:2.230559e-003,7:1.722729e-002):7.199298e-003,((34:5.329106e-003,32:1.703895e-002):3.590040e-003,((29:1.999940e-003,28:1.211855e-002):2.406409e-004,33:1.190566e-002):3.151545e-003):7.412261e-003):8.057660e-003):1.129377e-003):7.921860e-004,(38:9.939349e-003,39:1.328039e-003):1.400740e-002):6.479525e-003,((36:1.869660e-003,(26:4.250457e-003,((20:2.185427e-003,(24:1.469414e-003,25:2.686330e-005):5.861617e-003):4.022407e-003,21:3.538104e-003):7.312191e-003):6.746189e-003):1.361830e-003,35:1.077694e-002):2.181146e-003):3.615521e-003):1.755302e-004,(22:6.059368e-003,(27:1.410331e-002,31:5.683792e-003):5.677424e-004):4.435972e-003):2.514964e-002):2.762305e-003,18:4.435443e-003):1.784240e-003,(9:7.504718e-003,((10:1.246998e-002,((15:3.775366e-003,(14:2.047297e-004,3:1.667996e-003):2.530829e-004):8.600123e-003,((11:5.299657e-003,12:1.142864e-003):1.413954e-002,13:2.410320e-004):2.639454e-003):6.787671e-003):1.614890e-002,8:7.100675e-003):4.626526e-003):1.005820e-002):1.056050e-002):1.117806e-002):2.248836e-003,(19:1.340780e-002,(6:8.250847e-003,16:1.123210e-003):1.910294e-003):1.227222e-003,1:2.516576e-002);

tree gen.81000 = [&U] (((19:5.105375e-003,(6:4.401147e-003,16:1.879814e-002):6.897427e-004):4.216291e-003,17:8.358037e-003):5.930225e-004,((5:7.167552e-004,(4:4.211421e-003,40:1.608602e-002):6.921274e-004):6.011115e-003,((((41:3.800177e-002,42:1.259431e-002):1.889371e-002,(((36:2.183814e-003,(20:1.139255e-003,((21:6.159743e-003,26:5.849485e-003):2.577837e-003,(24:5.055980e-003,25:6.193089e-005):1.168003e-002):7.084686e-004):4.251005e-003):7.845930e-003,(((((2:5.512289e-003,(23:5.737310e-004,((37:1.906464e-003,7:5.784056e-003):1.171749e-003,(34:4.999963e-003,(32:1.436486e-002,(28:8.663209e-003,(29:1.265197e-002,33:3.725063e-003):3.227386e-003):8.751469e-003):2.206361e-003):1.221490e-002):1.003490e-003):4.001921e-003):1.616334e-004,(38:7.065107e-003,39:1.479407e-002):5.207024e-003):8.953701e-003,35:1.003640e-002):1.204194e-003,31:7.405604e-003):4.156998e-003,30:1.777828e-003):5.909999e-004):2.716338e-003,(22:6.012120e-003,27:1.201777e-002):3.789853e-003):2.456248e-002):2.117681e-003,18:1.033699e-002):1.197085e-002,(9:6.089937e-003,((10:7.562012e-003,8:2.196798e-005):5.571188e-004,(((3:1.568625e-003,14:3.216442e-004):4.777285e-003,15:3.447425e-003):6.740247e-003,(13:1.047706e-004,(11:1.632085e-003,12:2.579015e-004):1.494468e-002):7.105593e-003):4.704824e-003):4.035560e-003):9.436970e-003):3.121716e-003):2.116103e-002,1:1.737508e-002);

tree gen.82000 = [&U] ((6:1.483238e-003,16:1.650243e-002):2.386730e-003,((19:8.054984e-004,17:6.594266e-003):2.517615e-004,(((4:8.688617e-003,5:1.826896e-003):4.871150e-003,40:1.653704e-002):6.817056e-003,((18:1.072898e-002,((41:6.042349e-002,42:1.131174e-002):2.949565e-002,(27:5.973809e-003,(22:6.256850e-003,(((((((23:6.654821e-003,(((34:5.872330e-003,32:1.500324e-002):1.330851e-003,((29:9.481960e-003,28:8.843889e-003):1.676415e-003,33:8.459729e-003):9.140572e-003):1.083461e-002,(7:5.696168e-003,37:2.257120e-003):7.977623e-003):2.065388e-003):4.513507e-003,(38:2.907366e-002,39:1.553327e-002):2.677864e-003):2.498632e-003,2:2.555514e-003):1.160612e-002,(36:1.591107e-004,((26:7.259613e-003,(20:3.414097e-003,(24:2.800059e-003,25:1.163669e-002):1.472434e-003):5.158299e-003):1.971819e-003,21:4.591179e-003):2.086996e-002):1.555500e-002):6.465402e-003,30:2.521789e-003):2.674829e-003,31:6.633828e-003):1.950234e-003,35:1.260445e-002):2.066548e-003):1.386962e-003):1.077353e-002):4.166312e-003):8.957890e-003,(9:1.880032e-003,(((13:7.671408e-004,(11:2.154329e-003,12:4.229998e-005):1.359225e-002):2.688725e-003,(14:1.545667e-003,(15:1.757711e-003,3:1.989733e-003):6.028427e-003):5.201786e-003):6.751989e-004,(8:2.252731e-005,10:6.491317e-003):3.804760e-003):2.692355e-003):1.132488e-002):6.154391e-003):9.517482e-003):9.160929e-004,1:2.302077e-002);

tree gen.83000 = [&U] (17:8.692828e-003,(((19:2.576363e-003,6:1.635724e-003):5.514919e-003,16:1.371409e-002):1.215882e-002,((4:1.000883e-003,(40:1.737485e-002,5:5.059920e-003):2.088562e-003):3.539195e-003,((18:3.662163e-003,((41:5.816509e-002,42:1.231791e-002):3.417522e-002,(35:1.460153e-002,((30:1.929890e-003,((27:1.893083e-002,22:6.077638e-003):1.907413e-003,31:6.305158e-003):3.761256e-003):9.251130e-004,(((38:6.124109e-003,39:5.411544e-003):7.024063e-003,((23:1.055832e-003,((34:7.302004e-003,(((28:3.841988e-003,29:1.045677e-002):2.800262e-004,33:7.492740e-003):1.169037e-002,32:1.666250e-002):2.796770e-004):2.995977e-003,(7:2.654781e-003,37:6.475450e-003):6.107990e-003):4.237713e-003):4.176921e-003,2:1.442048e-003):5.073836e-003):2.473428e-003,(36:8.429574e-004,(26:4.098968e-003,(21:2.532837e-003,(20:1.652525e-003,(24:2.660677e-003,25:4.342731e-003):1.507429e-003):4.135865e-003):6.215728e-003):1.076278e-002):8.569032e-003):6.542928e-003):4.920580e-003):1.363105e-002):2.235459e-003):6.334278e-003,(9:2.073312e-003,((((3:2.473317e-003,(14:1.391080e-002,15:1.129241e-003):4.345603e-003):6.669181e-003,(13:1.484003e-003,(11:2.502405e-003,12:6.752243e-003):1.143565e-002):3.995995e-003):5.770188e-003,8:2.030089e-003):1.445634e-003,10:4.256067e-003):6.922120e-003):1.248915e-002):8.687993e-003):1.908217e-002):7.521267e-003,1:2.168885e-002);

tree gen.84000 = [&U] ((((6:1.097014e-002,16:1.078257e-002):1.301115e-002,((40:2.224702e-002,(4:1.374483e-003,5:1.365270e-003):6.854414e-004):6.275767e-003,(((9:1.762442e-003,(8:6.652324e-004,(10:3.752336e-003,((13:1.261493e-003,(11:2.127197e-003,12:2.463732e-003):1.388054e-002):6.306880e-003,((3:1.016819e-004,15:6.568598e-004):3.439635e-003,14:3.419210e-003):7.226685e-003):1.178305e-003):1.755224e-002):1.229616e-002):8.948958e-003,((41:3.956378e-002,42:1.047097e-002):2.212947e-002,(((22:2.947488e-003,(((36:2.773473e-003,((20:5.925697e-003,26:5.339701e-003):8.542863e-004,(21:3.178025e-003,(24:7.321947e-003,25:4.697737e-003):6.053649e-003):4.132182e-005):7.581925e-003):6.335108e-003,35:1.222235e-002):3.714788e-003,30:1.123361e-003):3.228813e-003):1.975142e-003,(27:1.479199e-002,(((38:1.582724e-002,39:3.958131e-003):1.536378e-003,(23:4.522105e-004,(((34:6.207148e-003,32:1.548714e-002):8.612019e-004,((28:6.562597e-003,33:6.477697e-003):6.391503e-003,29:6.079125e-003):1.157831e-002):3.263566e-003,(7:4.757715e-003,37:1.086343e-004):2.314649e-002):7.057675e-003):6.742347e-003):7.052742e-004,2:4.719237e-003):4.611769e-003):2.386559e-003):5.833619e-004,31:2.264921e-003):2.777635e-002):3.983509e-003):8.724723e-004,18:2.997887e-003):4.481933e-003):1.497790e-002):1.725758e-004,17:7.601841e-003):8.222725e-004,19:1.055963e-002,1:1.863986e-002);

tree gen.85000 = [&U] ((6:1.621537e-002,16:1.152165e-002):9.330473e-003,(17:9.506804e-003,(((40:3.084655e-002,(5:2.937563e-003,4:2.957525e-003):7.403927e-004):7.538084e-003,((9:2.365668e-004,((((13:4.393532e-004,(11:2.692073e-003,12:9.096689e-003):7.662501e-003):3.154383e-003,(15:1.997336e-003,(3:7.615435e-003,14:4.712786e-003):4.393635e-005):1.819648e-002):7.154826e-003,10:2.871679e-003):2.217494e-003,8:1.068716e-004):6.969829e-003):1.414383e-002,(((41:5.557695e-002,42:5.896565e-003):2.199883e-002,(27:1.548867e-002,(((23:3.610785e-003,(((28:9.218762e-003,(29:2.039784e-003,33:9.797546e-003):1.611707e-004):6.448127e-003,(34:1.189364e-002,32:2.365116e-002):6.454172e-003):2.908389e-003,(7:8.920013e-003,37:3.937415e-004):4.311047e-003):2.679741e-003):6.070203e-003,((38:2.553936e-002,39:6.304269e-003):4.199906e-003,2:1.574274e-002):1.362934e-003):6.980399e-003,(22:7.720915e-003,(((35:6.422813e-003,(36:5.286164e-003,((24:4.751503e-003,25:6.153491e-003):9.658158e-003,((21:7.451226e-004,26:1.253912e-002):9.172923e-004,20:3.814972e-003):2.278749e-003):1.487556e-003):1.738570e-002):2.893960e-004,30:1.509232e-003):7.767306e-004,31:2.328511e-002):1.610570e-003):6.115994e-003):3.775834e-003):3.890558e-002):1.110571e-002,18:1.538776e-003):1.692144e-003):1.808011e-002):1.044774e-002,19:3.139306e-003):5.462497e-004):5.045080e-004,1:1.742641e-002);

tree gen.86000 = [&U] ((((4:3.324035e-003,(5:6.484788e-004,40:2.600997e-002):2.569583e-003):3.758764e-003,((9:6.247154e-004,((((3:3.388937e-003,15:3.197461e-003):1.115507e-002,14:4.371706e-005):1.783446e-002,(8:5.335380e-003,10:2.507637e-003):1.224597e-003):3.537651e-003,(13:7.456913e-004,(11:1.207410e-003,12:2.486914e-003):1.191679e-002):4.133265e-003):4.829472e-003):2.241291e-002,(((41:4.686276e-002,42:7.552041e-003):2.982044e-002,(27:1.357287e-002,(22:5.715821e-003,((30:5.099414e-003,35:1.811816e-002):2.841232e-004,(((36:4.457319e-003,((24:2.117091e-003,25:2.381526e-003):6.162636e-003,(26:1.357604e-002,(20:8.401749e-003,21:4.610635e-004):1.393716e-004):2.268030e-003):8.863502e-003):6.980531e-003,((23:3.449461e-004,((((28:2.407572e-002,(29:5.698672e-003,33:9.764487e-003):1.274172e-003):4.366674e-003,32:8.520201e-003):5.147652e-003,34:1.002877e-002):5.632873e-003,(7:1.593831e-002,37:1.314970e-003):1.061908e-002):1.584592e-003):2.420475e-003,((38:1.428924e-002,39:5.315790e-003):5.940632e-003,2:1.062521e-002):4.200989e-004):3.867272e-003):1.290712e-002,31:2.003693e-002):2.092624e-003):2.968473e-003):2.177511e-003):1.054115e-002):1.347920e-002,18:4.860324e-003):5.934678e-003):1.589363e-002):2.098533e-002,17:7.578789e-003):5.055406e-003,((16:9.358415e-003,6:3.233589e-003):7.059103e-003,19:1.203504e-002):5.516810e-003,1:1.218308e-002);

tree gen.87000 = [&U] (((17:1.109567e-002,19:1.660449e-002):7.086534e-003,(6:6.128172e-003,16:2.108710e-003):4.938182e-003):1.407191e-003,(((5:1.697782e-003,4:6.964292e-004):8.716339e-003,40:4.056539e-002):6.939215e-003,(18:4.823336e-003,((9:7.222593e-003,(((10:9.501904e-003,8:1.673932e-004):2.128428e-003,(13:1.070168e-002,(11:2.573912e-003,12:3.119525e-003):1.821787e-002):6.332051e-003):3.777793e-003,((3:2.733974e-003,15:2.723103e-003):1.427133e-002,14:8.671735e-003):3.632804e-002):3.554886e-003):2.322373e-002,((41:9.514686e-002,42:1.130186e-002):3.712516e-002,((((30:3.756771e-003,27:2.063598e-002):1.853161e-003,(22:4.031641e-003,((2:2.835570e-003,(23:1.475760e-003,(((((29:8.189125e-003,28:8.116914e-003):3.112961e-003,33:1.472470e-002):2.012767e-002,34:1.445865e-002):7.139149e-003,32:1.235241e-002):2.349186e-003,(7:1.869375e-002,37:1.967893e-003):1.231314e-002):8.064031e-003):3.760263e-003):1.544859e-003,(38:1.483739e-002,39:1.257464e-002):5.681488e-003):7.361111e-003):7.729106e-004):1.525925e-003,31:3.061428e-002):1.479206e-003,(35:2.711437e-002,(36:5.536364e-003,(((24:1.655748e-002,25:7.382615e-004):3.519967e-003,(26:4.505437e-003,20:1.411790e-002):6.749820e-004):6.256288e-003,21:2.760650e-004):2.030469e-003):1.898056e-002):2.070257e-003):1.655987e-002):3.567033e-002):4.149353e-005):1.331942e-002):2.957059e-002,1:1.618714e-002);

tree gen.88000 = [&U] ((((40:3.738917e-002,5:9.155455e-004):3.331221e-003,4:6.383893e-003):2.205619e-002,((9:7.824230e-005,(8:2.396230e-004,((10:5.255237e-003,((14:2.135640e-003,15:3.319711e-003):5.178905e-003,3:1.075025e-002):3.084135e-003):3.238670e-003,(13:2.049159e-003,(11:2.807321e-003,12:3.402413e-003):2.103547e-002):7.666736e-003):4.727462e-003):1.076524e-002):1.436167e-002,(((41:7.858638e-002,42:1.097188e-002):1.975244e-002,(31:3.056329e-002,(27:1.824045e-002,(((2:1.646051e-002,(38:6.291903e-003,39:7.730048e-003):2.430917e-003):3.108800e-003,(23:1.737665e-003,(((((28:1.922834e-002,29:9.825540e-003):1.016714e-002,33:1.605998e-002):1.073716e-002,34:2.192350e-002):9.774869e-004,32:2.158011e-002):6.408003e-003,(7:1.130598e-002,37:6.716128e-004):6.541710e-003):2.363534e-002):1.312110e-002):2.830489e-002,((30:5.447557e-003,22:9.013137e-003):4.358843e-004,(35:3.113619e-002,(36:8.048748e-003,((21:1.220243e-003,26:6.082999e-003):2.713609e-003,((24:1.690171e-002,25:8.052091e-004):1.375151e-002,20:6.817474e-006):1.033377e-003):1.989191e-002):1.509283e-002):3.112472e-003):1.242397e-003):3.771569e-003):4.811918e-004):3.043089e-002):2.006964e-002,18:1.572185e-002):1.937885e-003):1.270520e-002):3.126341e-002,(17:1.155242e-002,(19:4.070829e-003,(16:2.225164e-003,6:7.100220e-003):1.599119e-003):4.596521e-004):2.055019e-003,1:2.271000e-002);

tree gen.89000 = [&U] (19:3.809521e-003,((((4:6.204443e-003,5:5.063819e-003):3.770017e-003,40:4.038949e-002):2.050061e-002,((9:1.209324e-002,(8:2.133541e-004,((13:1.254671e-003,(11:5.474276e-003,12:3.306772e-003):1.571544e-002):1.005731e-002,((10:2.505115e-003,3:1.711730e-003):6.294084e-003,(14:3.945595e-003,15:3.821599e-003):4.398053e-003):6.575453e-003):6.187449e-003):8.782350e-004):1.080614e-002,(((41:7.226916e-002,42:1.066346e-002):3.547279e-002,((27:1.776687e-002,(22:4.772296e-003,((((23:2.845099e-003,(((34:1.820237e-002,32:2.052363e-002):8.390577e-004,((33:1.560853e-002,29:9.565024e-003):3.319188e-003,28:1.861562e-002):1.865950e-002):7.592658e-003,(7:1.098817e-002,37:6.245712e-004):5.752636e-003):8.767279e-003):4.860648e-003,((38:5.478245e-003,39:1.070511e-002):2.781916e-003,2:1.485949e-002):4.647067e-004):4.340323e-003,30:6.860672e-003):8.515710e-004,(35:3.026096e-002,(36:9.170948e-003,(((21:2.471266e-004,26:1.183406e-002):2.255847e-003,20:2.009032e-004):1.444400e-003,(24:4.387343e-004,25:2.572846e-003):2.425011e-003):2.042650e-002):3.715148e-003):4.614593e-003):2.945603e-003):3.053202e-003):1.042238e-003,31:1.982028e-002):4.509605e-002):5.596896e-003,18:4.522510e-003):4.683197e-003):1.195324e-002):6.434216e-003,(17:6.909059e-003,(16:1.714100e-002,6:5.596475e-003):1.551726e-002):2.345624e-003):1.918246e-003,1:1.379097e-002);

tree gen.90000 = [&U] ((6:1.021207e-002,16:1.332047e-002):1.009538e-002,((17:1.146855e-002,((5:7.547841e-004,(40:4.268620e-002,4:6.669889e-003):1.038835e-003):2.113518e-002,((9:1.619358e-003,(10:8.044036e-004,((3:4.650676e-003,((13:2.472301e-003,(11:5.772658e-003,12:3.409130e-003):2.041385e-002):8.482871e-003,(14:3.795974e-003,15:1.345206e-003):5.591914e-003):5.566141e-004):1.517181e-002,8:6.193235e-004):2.768906e-003):5.443416e-003):1.208322e-002,(((41:7.450618e-002,42:1.099354e-002):3.657082e-002,(((22:1.503180e-003,(30:4.725751e-003,(31:6.749294e-003,(((38:9.339870e-003,39:1.648393e-002):1.089547e-003,2:3.825510e-003):1.708790e-003,(23:1.036125e-002,(((32:2.208030e-002,34:6.658142e-003):7.887410e-005,((33:4.492599e-003,29:7.315459e-003):2.379213e-003,28:8.716911e-003):2.091295e-003):8.150390e-003,(7:2.916579e-002,37:6.011232e-004):1.941845e-003):9.345691e-003):7.615066e-003):8.402811e-003):3.071220e-003):6.752738e-004):1.363240e-004,(35:2.499273e-002,(36:9.454826e-003,((26:1.329434e-002,((24:7.556144e-003,25:2.541050e-003):2.739408e-003,20:4.632916e-003):4.185968e-003):6.419088e-003,21:1.138267e-003):8.486038e-003):8.729410e-003):3.694159e-003):1.712974e-003,27:1.981662e-002):2.735523e-002):2.325410e-002,18:1.235477e-002):2.984216e-003):1.161135e-002):1.619572e-002):1.494426e-004,19:6.324859e-003):3.354449e-003,1:1.178698e-002);

tree gen.91000 = [&U] (((6:1.525129e-002,16:1.204329e-002):5.836982e-003,17:1.007901e-002):2.398839e-003,(19:8.521176e-003,((5:2.037248e-003,(40:4.044211e-002,4:1.324576e-002):4.976625e-003):1.270508e-002,((10:4.638776e-003,((((3:3.610130e-003,(15:6.062584e-003,14:2.703704e-003):9.005119e-003):2.469810e-002,9:5.522712e-003):9.733115e-003,(13:9.987671e-003,(11:7.770240e-003,12:6.573805e-003):3.341955e-002):8.304935e-003):3.176755e-004,8:7.166275e-004):2.690491e-003):2.496920e-002,(((41:9.862928e-002,42:2.249815e-002):4.922586e-002,(((27:1.809384e-002,(35:2.090386e-002,(36:1.025834e-002,((24:2.296622e-003,25:1.859161e-003):5.755394e-003,((20:4.393470e-003,26:6.398766e-003):1.119614e-003,21:2.014047e-003):1.992522e-003):1.696149e-002):8.684718e-003):8.238190e-003):6.448055e-003,(22:3.169008e-003,((((38:1.031132e-002,39:1.789873e-002):1.011570e-002,2:1.782239e-003):8.174006e-004,(23:5.108002e-003,((34:7.830469e-003,(32:2.908092e-002,((33:1.377686e-002,28:1.862900e-002):5.214931e-003,29:7.255253e-003):9.053142e-003):8.028715e-003):3.434228e-003,(7:3.075576e-002,37:6.975214e-003):1.785926e-002):5.370554e-003):2.871746e-003):1.085932e-002,30:5.968327e-003):3.848680e-003):1.519544e-003):2.582039e-003,31:1.422190e-002):3.302100e-002):2.946749e-002,18:4.620614e-003):1.276192e-002):3.099478e-002):1.467972e-002):1.246212e-004,1:2.385778e-002);

tree gen.92000 = [&U] ((6:1.766458e-002,(16:7.501339e-004,19:1.449815e-002):5.316041e-003):1.973257e-003,((((40:3.273303e-002,4:5.524677e-003):2.340269e-003,5:1.232933e-004):1.113474e-002,(((41:4.598161e-002,42:1.939210e-002):3.199996e-002,((2:1.646379e-002,((38:9.509231e-003,39:3.550492e-003):1.306798e-002,(23:1.182360e-003,((((28:9.124553e-003,33:1.562831e-002):6.955558e-003,29:6.049449e-003):9.324770e-003,(32:3.387651e-002,34:6.518646e-003):1.583457e-003):9.397322e-003,(7:2.489309e-002,37:1.724968e-002):1.829185e-002):9.311672e-003):5.189474e-004):1.907380e-003):9.288754e-003,(((30:1.819165e-003,((35:1.691916e-002,(36:1.123350e-002,((20:1.250441e-003,21:1.500501e-003):1.228174e-003,((24:2.311144e-003,25:1.504767e-003):1.783229e-003,26:3.510688e-003):1.197127e-003):8.900550e-003):1.635339e-002):5.110895e-003,22:1.250541e-002):3.323477e-003):1.650848e-004,27:8.685183e-003):3.732446e-004,31:1.129164e-002):7.901476e-003):2.752166e-002):1.450186e-002,((10:3.106450e-003,(8:5.838351e-003,(9:1.893611e-003,((13:1.173108e-002,(11:6.289075e-003,12:1.832894e-002):3.088311e-002):5.087698e-003,((3:9.404291e-004,15:9.371171e-003):1.318682e-002,14:3.921663e-003):3.045237e-003):2.031361e-003):1.086204e-003):1.297825e-002):1.518065e-002,18:4.287486e-003):2.752231e-003):3.226015e-002):2.520545e-002,17:1.429228e-002):7.187952e-003,1:2.802621e-002);

tree gen.93000 = [&U] ((19:9.949838e-003,((16:3.310043e-003,6:2.009605e-002):6.851721e-003,(((4:8.775250e-003,5:1.037498e-002):5.872343e-003,40:4.337188e-002):1.338861e-002,((18:6.383069e-003,((41:5.556746e-002,42:2.343480e-002):6.792944e-002,(((35:2.432211e-002,(36:6.456021e-003,(26:2.430576e-002,(20:4.734348e-003,((24:1.042377e-004,25:9.039039e-004):5.191569e-003,21:2.490617e-003):2.300935e-003):4.566987e-003):5.052229e-003):7.008699e-003):7.992984e-003,(((38:1.399811e-002,39:4.290668e-003):1.390417e-002,2:2.466266e-002):1.996513e-003,(23:4.103924e-003,((((33:2.160963e-002,(29:7.310586e-003,28:1.083904e-002):2.629008e-003):1.031015e-002,32:1.715950e-002):1.943513e-003,34:7.447125e-003):1.206088e-002,(7:3.208787e-002,37:2.166997e-004):1.926881e-002):1.229471e-002):4.030200e-003):2.083057e-002):2.386183e-003,((31:2.558209e-002,(27:1.126934e-002,22:1.113271e-002):1.449568e-003):3.117505e-003,30:4.756787e-003):4.694240e-003):2.802066e-002):5.406550e-003):6.898815e-003,((8:2.239492e-004,((13:3.761407e-003,(11:1.299283e-002,12:5.238331e-003):2.659297e-002):3.479378e-003,(10:2.425844e-002,(14:4.822305e-003,(15:2.269820e-003,3:6.262491e-003):1.636289e-003):1.316404e-002):6.035570e-003):3.548741e-003):1.024758e-002,9:2.029545e-003):1.140474e-002):1.513933e-002):3.583909e-002):5.969030e-003):3.014675e-003,17:2.100426e-002,1:3.234832e-002);

tree gen.94000 = [&U] (((((40:2.428387e-002,4:5.119560e-003):5.926255e-003,5:1.667702e-003):3.122388e-002,(((8:1.081866e-002,(((13:3.228948e-004,(11:9.567468e-003,12:4.880334e-003):3.228480e-002):9.305005e-003,(3:6.064632e-003,(14:2.347910e-004,15:1.181866e-004):8.041579e-003):1.767811e-003):2.270874e-003,10:8.954461e-003):1.647748e-002):4.847965e-003,9:5.735932e-003):1.765956e-002,(18:1.124922e-002,((41:5.034132e-002,42:2.123075e-002):5.011063e-002,((((27:1.113635e-002,31:4.582680e-003):4.279779e-003,((23:3.621019e-003,(((((29:8.886335e-003,28:6.951431e-003):1.570575e-004,33:1.888763e-002):1.072542e-002,34:7.127144e-003):2.723918e-004,32:1.967071e-002):7.470922e-003,(7:3.431991e-002,37:3.403344e-003):1.445082e-002):5.967950e-003):1.065438e-003,(2:2.140797e-002,(38:1.580763e-002,39:1.602019e-002):1.211981e-002):4.174494e-003):1.513436e-002):1.043551e-003,((35:1.465824e-002,(36:2.464399e-003,((20:1.428237e-003,((24:1.357291e-003,25:8.188914e-004):1.442357e-002,26:4.378938e-003):4.241262e-003):1.039249e-002,21:2.573039e-003):7.284441e-003):6.578728e-003):3.450692e-003,22:2.371902e-003):5.422645e-003):4.237395e-003,30:4.169689e-003):1.849795e-002):2.608114e-002):2.413655e-003):1.737608e-002):3.612560e-002,(17:9.062832e-003,(16:8.511818e-003,6:7.773165e-003):1.950714e-003):1.067106e-002):7.379471e-004,19:8.473666e-003,1:2.327028e-002);

tree gen.95000 = [&U] (17:9.601718e-003,((19:6.052597e-003,(6:6.075498e-003,16:7.299542e-003):4.651475e-003):4.483268e-003,((((8:9.723261e-004,((((15:3.316123e-004,14:3.852583e-003):5.761098e-003,3:5.023643e-003):1.399017e-002,(13:5.336945e-003,(11:1.627529e-002,12:3.141872e-003):2.645171e-002):3.020479e-003):5.911069e-003,10:7.782049e-003):7.814435e-003):5.456690e-003,9:9.330606e-004):1.697360e-002,(18:1.549561e-002,((41:6.485111e-002,42:1.120339e-002):4.276464e-002,(22:1.574765e-002,((((35:1.199029e-002,(36:8.513910e-003,(((20:3.267209e-003,21:5.845675e-004):1.076401e-002,26:8.994695e-003):6.626731e-003,(24:1.174660e-003,25:3.326881e-003):1.276458e-002):1.090965e-002):3.714693e-003):1.557595e-003,27:7.734845e-003):1.214718e-003,(31:1.492929e-002,((23:2.363552e-003,(((34:3.010009e-003,32:3.245313e-002):6.044285e-003,(33:1.725688e-002,(29:7.583639e-003,28:4.619502e-003):7.158885e-004):6.085447e-003):3.622304e-003,(7:2.109890e-002,37:2.904429e-003):1.431875e-002):1.407927e-002):4.843647e-003,(2:1.255171e-002,(38:2.098587e-002,39:7.454377e-003):5.850584e-003):6.236047e-003):2.986800e-002):1.194099e-003):9.164748e-003,30:3.269445e-003):1.964180e-004):1.231932e-002):8.941002e-003):8.342769e-003):2.635608e-002,((4:7.939913e-003,5:1.001395e-004):4.938735e-003,40:3.012845e-002):7.319817e-003):2.071625e-002):3.867445e-003,1:2.243770e-002);

tree gen.96000 = [&U] (((16:1.434693e-002,6:5.187036e-003):9.309426e-003,((((((11:1.948981e-003,12:1.520691e-002):3.215351e-002,13:5.004239e-003):2.263893e-003,(((15:2.722621e-003,(3:1.164678e-002,14:5.215165e-003):3.021530e-003):1.009724e-002,8:3.171096e-003):1.350447e-003,10:5.964976e-003):2.766197e-003):2.958601e-003,9:8.532832e-004):2.215833e-002,(18:1.312446e-002,((41:5.191854e-002,42:9.489031e-003):3.905789e-002,((((35:1.015552e-002,(36:1.119304e-002,(((21:1.123780e-002,20:4.015145e-003):2.627533e-003,26:7.310794e-003):6.742644e-003,(24:7.128222e-003,25:4.741138e-004):4.053121e-003):3.556901e-003):1.461188e-002):4.297696e-003,(22:5.445392e-003,30:4.408175e-003):1.766772e-003):3.052367e-003,(31:6.432797e-003,27:1.765607e-002):2.268272e-003):2.265316e-003,(((38:1.706399e-002,39:3.179450e-003):7.189230e-003,2:5.383218e-003):6.935814e-003,(23:5.238329e-003,(((32:2.793130e-002,((33:1.502819e-002,28:3.808615e-003):3.702784e-004,29:7.428287e-003):3.010728e-003):8.778110e-004,34:1.207880e-002):3.105026e-003,(7:1.958402e-002,37:1.431245e-005):1.492124e-002):9.037992e-003):1.431017e-002):1.755799e-002):1.089344e-002):8.603383e-003):7.066149e-003):1.153397e-002,((40:2.678819e-002,4:3.336792e-003):5.852642e-003,5:4.025665e-003):1.066943e-002):1.896901e-002):2.945684e-004,(17:7.778959e-003,19:8.419751e-003):4.029270e-003,1:1.935142e-002);

tree gen.97000 = [&U] (17:2.314050e-002,((6:1.367736e-002,16:7.313760e-003):9.496880e-003,(19:1.052951e-002,((((10:2.697254e-002,((8:6.704457e-004,((11:9.261102e-003,12:2.346569e-004):3.633056e-002,13:7.359589e-004):3.012264e-003):3.530201e-003,(14:8.572838e-004,(15:9.210771e-003,3:6.361499e-004):3.965006e-003):3.511420e-002):4.831673e-004):8.230104e-003,9:6.544845e-003):9.834489e-003,(18:6.445665e-003,((41:6.998991e-002,42:2.441252e-002):1.836029e-002,(((27:2.500451e-002,(((38:2.300348e-002,39:4.286126e-003):9.936690e-003,(23:9.433295e-003,((((33:1.162122e-002,28:1.413376e-002):1.603463e-002,29:3.341865e-003):4.395754e-003,(34:1.593833e-002,32:1.347635e-002):2.209952e-004):1.229824e-003,(7:4.095307e-002,37:1.435764e-004):1.435329e-002):1.788128e-002):4.083904e-003):1.701752e-003,2:4.972871e-003):9.732816e-003):3.320902e-003,((35:1.783725e-002,(36:1.508902e-002,(((24:1.781287e-003,25:8.234043e-004):2.199692e-002,(26:6.906571e-003,21:1.643789e-002):3.299073e-003):1.315287e-002,20:3.492245e-003):4.323892e-003):1.244312e-002):1.143956e-002,(22:2.120400e-003,31:7.780731e-003):2.423224e-004):1.767764e-003):4.719164e-003,30:6.167189e-003):4.271957e-002):4.740226e-003):2.128213e-002):3.228537e-002,(40:3.801011e-002,(4:1.270118e-003,5:1.246663e-002):1.152333e-002):7.603888e-003):2.463290e-002):4.600751e-003):3.627336e-004,1:3.894880e-002);

tree gen.98000 = [&U] ((6:1.570486e-003,16:6.254203e-003):5.723522e-003,(17:2.265241e-002,((((((10:7.769010e-003,(((11:9.256535e-003,12:4.046665e-003):2.399182e-002,13:1.471262e-003):4.144840e-003,((15:5.690017e-003,14:9.862211e-004):2.114314e-003,3:3.270110e-003):1.782951e-002):1.210245e-002):1.044273e-002,8:1.743221e-005):8.654089e-004,9:2.469633e-003):9.491161e-003,(18:5.805774e-003,((41:6.995539e-002,42:3.809098e-003):1.835124e-002,(((38:1.586282e-002,39:1.256244e-002):4.473881e-003,((23:7.420408e-003,(((((29:8.781694e-003,33:8.051628e-003):4.987455e-003,28:1.156743e-002):1.289399e-002,34:1.622097e-002):6.678557e-003,32:2.224247e-002):1.030748e-002,(7:1.529046e-002,37:1.788674e-003):1.353480e-002):7.331310e-003):1.374603e-002,2:3.283369e-003):5.369803e-003):8.055558e-003,((((35:2.710721e-002,(36:2.243196e-002,(26:6.879543e-003,(20:1.759218e-003,((24:5.699320e-003,25:8.229982e-004):1.449706e-003,21:4.782989e-003):5.124093e-003):7.575379e-005):5.485076e-003):1.004165e-002):4.940850e-003,(22:1.016840e-002,30:3.540979e-003):5.477430e-003):1.400014e-004,27:2.439040e-002):4.044917e-003,31:1.430054e-002):3.121304e-003):4.475035e-002):1.393456e-002):7.144578e-004):5.973127e-003,((4:2.269761e-002,5:3.938491e-004):2.148200e-003,40:4.478210e-002):1.599736e-002):2.072350e-002,19:8.985902e-003):3.437374e-003):6.472403e-003,1:2.936470e-002);

tree gen.99000 = [&U] (19:3.824746e-003,(17:1.383649e-002,((6:1.763354e-002,16:9.161220e-003):1.017495e-002,((4:1.583036e-002,(5:1.043278e-002,40:6.208681e-002):7.010247e-004):3.128478e-002,(((((11:4.875417e-003,12:5.189472e-003):3.277081e-002,13:1.993423e-003):9.035850e-003,(3:8.929700e-003,(15:4.713782e-003,14:1.846728e-003):4.824542e-003):2.531086e-002):7.366788e-003,((10:8.703656e-003,8:4.237651e-003):2.018687e-002,9:1.350766e-002):3.918564e-003):4.909806e-002,(18:2.304050e-003,((41:9.478301e-002,42:1.155904e-002):3.764317e-002,((35:3.070495e-002,(36:2.448636e-003,((21:4.793285e-004,((24:2.231948e-003,25:3.045850e-004):1.299136e-002,20:3.592662e-003):5.895669e-003):2.729069e-004,26:7.131716e-003):4.759411e-003):1.695107e-002):4.233782e-003,(((2:5.912797e-003,(38:2.081578e-002,39:2.308055e-003):1.695730e-002):9.448394e-003,(23:5.540962e-003,(((32:2.597501e-002,34:1.207522e-002):1.584683e-003,(29:2.981620e-002,(33:1.090920e-002,28:1.588184e-002):5.080851e-003):1.332307e-002):7.113843e-003,(7:2.758520e-002,37:2.905527e-003):2.283761e-002):3.220859e-003):6.531356e-003):1.487049e-002,(30:1.179686e-003,(31:1.146355e-002,(27:3.354711e-002,22:6.551760e-003):1.128037e-002):2.942775e-003):8.081407e-003):1.539185e-003):6.142476e-002):9.019549e-003):9.478441e-003):1.539100e-002):1.876918e-002):1.386276e-003):1.216287e-003,1:4.101007e-002);

tree gen.100000 = [&U] ((6:2.177725e-002,16:2.129751e-002):3.953066e-003,(19:1.629342e-003,(((4:6.567844e-003,(40:7.738550e-002,5:2.245728e-003):2.919240e-004):1.420929e-002,((9:1.170233e-002,(((((11:1.680263e-003,12:3.367756e-002):4.883570e-002,13:4.103591e-005):1.547278e-002,8:4.075832e-004):3.885733e-003,(15:1.247927e-002,(3:9.840283e-003,14:1.134632e-003):1.431420e-002):2.701405e-002):1.372964e-002,10:2.847432e-003):1.230872e-002):5.459043e-002,(18:3.602410e-003,((41:1.080902e-001,42:2.321292e-002):7.086056e-002,(((35:3.653000e-002,(36:1.989198e-002,((20:1.451691e-002,(26:1.541296e-002,21:7.762850e-003):5.544447e-003):2.093284e-003,(24:7.571367e-003,25:5.615076e-004):1.012296e-002):7.137636e-003):1.686168e-002):7.526274e-003,22:8.074973e-003):4.177898e-003,((27:3.607902e-002,31:1.363831e-002):2.943460e-003,(30:9.219826e-003,(((38:3.718605e-002,39:2.412316e-002):3.913016e-002,2:7.034516e-003):4.501462e-003,(23:1.117049e-003,(((29:7.963908e-003,(28:1.113198e-002,33:1.625392e-002):2.207701e-003):6.940115e-003,(32:3.021288e-002,34:1.432832e-002):1.069563e-002):1.353929e-002,(7:5.714626e-002,37:1.291784e-002):3.419672e-002):3.365235e-003):4.826755e-003):1.258651e-002):2.202982e-003):4.541922e-003):3.831391e-002):1.679444e-002):7.671928e-003):4.858484e-002):2.832133e-002,17:5.440332e-002):9.922151e-003):1.026837e-003,1:4.632008e-002);

tree gen.101000 = [&U] (19:2.684447e-003,(((6:1.129087e-002,16:7.970341e-003):7.505342e-003,(((4:5.576461e-003,5:4.177810e-003):2.097089e-004,40:5.796867e-002):8.966386e-003,((9:3.588850e-003,((10:6.737028e-003,(((15:2.170338e-003,3:9.009879e-003):1.960010e-002,14:3.081402e-003):9.069202e-003,((11:1.012981e-002,12:3.951030e-003):3.894659e-002,13:9.976260e-003):5.079593e-003):2.213488e-002):6.714131e-003,8:1.885373e-003):9.949620e-003):1.441793e-002,(18:1.015812e-002,((41:1.026973e-001,42:9.272673e-003):7.276024e-002,((27:1.900323e-002,((35:9.692074e-003,(36:9.390591e-003,((((24:1.336801e-003,25:7.567044e-003):8.479465e-003,26:2.714163e-002):6.749980e-003,21:3.504575e-003):1.904820e-002,20:5.712726e-003):2.606738e-002):1.349614e-002):1.936338e-002,(22:1.637393e-002,((2:6.162988e-003,(38:5.370764e-002,39:1.351966e-002):5.017784e-003):4.893258e-003,(23:2.280564e-004,(((34:1.676078e-002,32:2.564163e-002):9.285203e-004,(33:1.379119e-002,(28:1.615357e-002,29:2.254289e-002):1.587745e-004):7.022693e-003):9.904553e-004,(7:4.849996e-002,37:5.945870e-004):2.870966e-002):1.149585e-002):3.395870e-003):1.121384e-002):1.723400e-003):1.205314e-002):2.990448e-003,(30:6.971798e-003,31:8.865010e-003):5.164842e-003):2.916516e-002):2.594262e-002):3.481303e-003):2.444115e-002):3.010633e-002):5.519058e-003,17:3.769558e-002):1.695482e-003,1:3.997357e-002);

tree gen.102000 = [&U] ((((6:2.384267e-002,19:1.208453e-002):7.301872e-003,16:2.455648e-002):3.259792e-003,17:2.151445e-002):4.244868e-003,(((40:6.062272e-002,4:5.173064e-003):6.055199e-003,5:4.487598e-005):1.417464e-002,((9:2.443480e-003,(((8:2.722987e-003,((15:8.248849e-004,3:5.282770e-003):1.739210e-002,14:8.025883e-003):1.195315e-002):8.738824e-004,10:1.313075e-002):1.156026e-002,((11:3.955993e-002,12:4.834913e-004):2.298655e-002,13:1.174185e-002):8.599738e-003):6.086022e-003):2.782830e-002,(18:1.598426e-002,((41:1.051363e-001,42:3.466938e-002):3.479273e-002,((30:4.190186e-003,22:3.839940e-003):5.094976e-003,(31:8.903127e-003,((27:1.973456e-002,(35:3.184432e-002,(36:7.923805e-003,((25:1.073399e-003,24:1.233417e-002):8.515473e-003,(20:5.889569e-003,(21:6.964202e-003,26:4.397240e-003):6.864767e-003):6.363591e-004):1.765907e-002):1.834790e-003):3.287826e-003):2.524319e-002,((38:9.812970e-003,39:1.384075e-002):1.181790e-002,(2:1.866913e-003,(23:2.339743e-003,((32:2.544290e-002,(34:1.715242e-002,((28:8.823998e-003,29:2.339712e-003):1.281324e-002,33:1.308917e-002):2.500786e-003):6.273641e-003):5.020094e-003,(7:5.564000e-002,37:5.871279e-004):8.669356e-003):1.723625e-002):2.386078e-003):1.643509e-003):2.271121e-002):1.307303e-002):8.754334e-003):3.039562e-002):1.896888e-002):6.454526e-003):3.992276e-002):4.120874e-002,1:3.723098e-002);

tree gen.103000 = [&U] ((16:2.114488e-002,6:3.293875e-002):4.697158e-003,((17:2.255072e-002,19:3.689617e-002):7.824457e-003,(((4:5.321296e-003,5:1.090398e-002):7.509912e-004,40:7.477005e-002):3.792197e-002,((9:3.347579e-003,(10:2.330444e-002,(((3:6.603303e-003,(15:1.999868e-002,14:2.069730e-003):6.329323e-003):5.492723e-002,((11:1.304614e-002,12:6.623852e-004):5.769599e-002,13:9.275731e-003):8.590407e-003):4.051804e-003,8:1.665580e-003):3.301536e-003):1.285558e-002):2.113029e-002,(18:1.440606e-002,((41:1.440372e-001,42:1.961525e-002):4.180198e-002,((((((35:4.659123e-002,(36:1.831184e-002,(((26:6.158722e-003,20:6.288809e-003):1.524910e-003,(25:7.160803e-004,24:3.426161e-002):7.848536e-003):2.962304e-003,21:4.544655e-003):1.921482e-002):1.378142e-002):4.208292e-003,30:1.102706e-002):6.701296e-003,31:2.645396e-002):1.525542e-002,22:1.449275e-002):8.089878e-003,27:6.399955e-002):8.170754e-003,((23:4.321752e-003,(((34:2.449362e-002,32:6.183050e-002):2.583662e-003,(28:3.852914e-002,(29:2.763701e-002,33:1.861747e-002):1.183202e-002):2.186149e-002):8.883461e-003,(7:5.202899e-002,37:4.974216e-003):1.184743e-002):1.187910e-002):1.390009e-002,((38:1.137299e-002,39:1.119381e-002):3.247069e-002,2:8.527977e-003):2.074923e-003):5.077948e-002):4.605414e-002):2.015463e-002):1.512802e-002):2.589802e-002):7.605983e-002):3.638835e-003,1:5.006835e-002);

tree gen.104000 = [&U] (17:2.607165e-002,(((6:2.179248e-002,16:8.737527e-003):9.478326e-003,((4:9.855500e-003,(40:8.234157e-002,5:1.264722e-003):1.497120e-003):1.653321e-003,((9:1.673394e-004,((((((14:7.539186e-003,15:1.930290e-003):9.970271e-004,3:1.284326e-002):3.703866e-002,10:3.517161e-002):5.524550e-002,(11:7.257521e-003,12:4.468424e-003):7.363479e-002):3.211665e-003,13:3.281700e-003):2.239124e-002,8:1.108318e-002):9.108647e-003):2.022421e-002,(18:4.591865e-002,((41:1.665263e-001,42:7.719846e-002):4.932427e-002,((((35:3.740664e-002,(36:1.757157e-002,((21:1.917670e-003,(20:4.820257e-003,26:5.705902e-002):1.134442e-002):7.751304e-003,(25:3.962811e-003,24:8.850107e-003):3.750618e-002):2.306279e-002):7.023209e-002):2.212614e-002,31:3.638875e-002):1.376071e-002,((22:7.181950e-003,(((23:4.229239e-003,(((32:7.187312e-002,34:2.831790e-002):2.020850e-004,(28:1.313014e-002,(33:2.838478e-002,29:2.469019e-002):1.897106e-003):2.466398e-002):1.013938e-002,(7:4.721058e-002,37:1.122384e-002):4.165519e-002):4.049017e-003):2.016715e-002,(39:8.754849e-003,38:9.028892e-002):3.706599e-003):1.056101e-003,2:6.463832e-003):3.636136e-002):2.065806e-003,30:1.185537e-002):4.338859e-003):2.732911e-003,27:7.277642e-002):5.880183e-002):2.868689e-002):1.857696e-002):2.378827e-002):6.695432e-002):1.082869e-004,19:1.994839e-002):3.461065e-003,1:5.494135e-002);

tree gen.105000 = [&U] (((5:9.664858e-004,4:1.082605e-002):3.731448e-002,(40:9.057432e-002,((9:1.289051e-003,((14:9.049939e-003,(15:2.402509e-003,3:1.592637e-002):1.911539e-002):3.197177e-002,((13:5.074284e-004,(11:7.661538e-003,12:7.231529e-003):7.077261e-002):4.088324e-003,(8:2.681867e-003,10:3.712957e-002):3.593799e-003):4.011221e-003):2.962582e-003):1.649002e-002,(18:4.847489e-002,((41:1.757966e-001,42:5.252217e-002):8.081707e-002,(31:2.474109e-002,((((23:1.408946e-002,(((32:2.077729e-002,((33:3.767135e-002,28:1.025527e-002):1.138168e-003,29:6.317412e-003):9.305218e-003):1.351728e-002,34:3.977297e-002):7.057233e-003,(7:4.983873e-002,37:1.462719e-002):3.057634e-002):4.500363e-002):1.018455e-002,2:1.107784e-002):7.250280e-003,(39:3.536000e-002,38:5.162940e-002):1.207227e-002):2.385783e-002,((27:3.148569e-002,(22:1.275211e-002,30:1.142388e-002):4.456290e-003):2.000870e-003,(35:4.067335e-002,(36:2.306879e-002,((26:1.545835e-002,(20:1.545172e-002,(25:9.623850e-003,24:1.096139e-002):9.567841e-003):3.015464e-002):1.779434e-002,21:5.523179e-003):1.624096e-002):2.233109e-002):5.887385e-003):6.925605e-003):1.573677e-003):1.004440e-001):5.525338e-002):1.697183e-002):7.026485e-003):6.439591e-003):5.059218e-002,((6:2.803085e-002,16:1.120600e-002):7.058954e-003,(19:2.325930e-002,17:2.677266e-002):1.607485e-002):1.546263e-002,1:4.769608e-002);

tree gen.106000 = [&U] (((((5:2.729577e-004,4:1.061264e-002):4.671991e-002,((9:1.746920e-002,(((13:2.939957e-003,(11:1.327503e-002,12:2.731857e-003):3.273087e-002):1.796048e-002,(14:1.500313e-004,(15:8.700395e-003,3:1.698360e-003):9.866297e-002):4.419547e-002):6.518334e-003,(10:1.297370e-002,8:7.326786e-003):9.408026e-003):2.068343e-002):1.727923e-002,((40:8.878893e-002,((41:1.450724e-001,42:7.702185e-002):8.064638e-002,((35:3.725724e-002,(((39:1.517212e-002,38:3.306043e-002):1.977187e-002,2:2.858120e-002):1.039735e-002,(23:5.634774e-003,((((29:1.333712e-002,(28:3.515832e-002,33:9.588345e-003):2.797647e-003):1.384088e-002,32:2.710579e-002):6.767103e-003,34:3.898897e-002):5.061075e-003,(7:6.604099e-002,37:1.119505e-002):7.535228e-003):6.120852e-003):2.427478e-002):1.329089e-002):4.906624e-007,(31:2.377421e-002,(30:3.405613e-002,((22:1.525311e-002,27:2.833654e-002):1.078021e-002,(36:2.555925e-002,(((26:2.287595e-002,20:1.551435e-002):5.302208e-003,(25:9.607367e-003,24:3.115290e-002):1.732121e-002):1.677985e-002,21:9.116498e-004):1.138867e-002):3.507972e-002):2.665494e-003):2.740790e-002):8.421467e-004):9.456698e-002):5.054269e-002):2.272347e-004,18:1.425309e-002):2.463825e-002):1.633960e-002):3.528899e-002,(6:2.510354e-002,16:2.393114e-002):1.209548e-002):5.783324e-003,17:3.509213e-002):1.101525e-002,19:2.336329e-002,1:3.510691e-002);

tree gen.107000 = [&U] (((6:8.439949e-003,16:2.112516e-003):3.283167e-002,17:2.287737e-002):3.490264e-002,((((40:9.424450e-002,4:9.994941e-003):2.788854e-003,5:4.220496e-003):2.095057e-002,((9:2.624832e-003,((((15:3.455604e-002,3:2.515732e-002):4.083417e-003,14:5.418995e-005):6.262560e-002,(10:1.067093e-002,8:1.549797e-002):8.853003e-003):6.086037e-003,(13:4.037917e-003,(11:1.992015e-002,12:7.160945e-003):3.304769e-002):2.387685e-002):1.191585e-002):6.770720e-002,(((41:1.040553e-001,42:8.516372e-002):9.826306e-002,(((27:2.055449e-002,30:9.462642e-003):1.395017e-003,(35:1.045219e-001,(((36:2.192399e-002,((25:4.229264e-004,24:1.335998e-002):2.368762e-002,(20:3.031449e-003,(26:2.028992e-002,21:1.464366e-002):2.041657e-003):1.801581e-002):4.474446e-002):2.679212e-002,22:5.303394e-003):2.269872e-002,(2:3.334056e-002,((23:1.210747e-002,((((28:3.411927e-002,33:2.606187e-002):4.493659e-003,29:1.726401e-002):7.268793e-003,(34:2.731620e-002,32:4.205065e-002):3.041295e-003):1.130109e-002,(7:4.485705e-002,37:1.097542e-002):3.666412e-003):4.375203e-003):1.506725e-002,(39:7.827864e-003,38:3.164568e-002):3.037249e-002):4.932115e-003):3.747985e-002):9.887015e-003):3.117040e-003):6.561318e-004,31:2.531225e-002):5.825622e-002):3.233675e-002,18:3.167931e-002):1.011230e-002):1.511431e-002):3.456645e-002,19:4.624526e-002):5.815004e-003,1:2.797938e-002);

tree gen.108000 = [&U] ((((5:6.632537e-004,40:9.545969e-002):2.588210e-003,4:2.928305e-002):1.752978e-002,((9:6.183434e-004,(((13:5.999165e-003,(11:1.810048e-002,12:2.304038e-003):6.164968e-002):1.006860e-002,8:1.248167e-003):1.343415e-002,(((3:2.764571e-004,15:5.486767e-004):2.267482e-002,14:6.976235e-003):3.495682e-002,10:2.946712e-002):7.322543e-003):2.559393e-002):6.292767e-002,(((41:1.059938e-001,42:3.205060e-002):9.214646e-002,((((35:7.679102e-002,(22:2.404472e-002,27:2.804686e-002):1.347754e-002):2.021553e-003,((2:1.888339e-002,((23:4.361880e-003,((34:2.709867e-002,(32:5.528591e-002,((33:2.709231e-002,29:1.706011e-002):6.589931e-003,28:5.165161e-002):9.938875e-003):4.497245e-003):2.407649e-002,(7:4.569268e-002,37:1.984100e-002):2.298385e-002):6.865066e-003):4.210739e-002,(39:2.466107e-002,38:3.933957e-002):2.424750e-002):4.210689e-003):3.663041e-002,(36:2.575815e-002,((20:1.716896e-002,((25:9.468791e-004,24:3.244302e-002):3.094281e-002,21:1.713727e-003):2.139215e-002):9.664064e-003,26:1.966571e-002):4.574588e-002):4.701356e-002):1.315299e-004):1.176761e-002,31:2.774471e-002):5.954868e-003,30:7.738538e-003):6.202632e-002):3.058264e-002,18:1.312420e-002):4.172578e-002):6.260726e-002):1.058227e-001,((19:1.049394e-002,17:2.694810e-002):8.210149e-003,(16:2.145499e-003,6:1.481473e-002):1.958822e-002):2.825980e-003,1:3.017312e-002);

tree gen.109000 = [&U] ((((16:1.047503e-002,6:2.392796e-002):3.288167e-003,((4:1.640837e-002,(5:2.018684e-002,40:1.194935e-001):5.156921e-002):2.400464e-002,((9:3.180676e-003,((13:3.927429e-003,(11:1.312077e-002,12:4.316571e-004):7.162771e-002):1.066087e-002,((8:4.781353e-003,(15:4.684183e-002,(14:1.009796e-002,3:6.133109e-003):5.238630e-003):4.143074e-002):2.132313e-004,10:3.706643e-002):6.450139e-003):1.529364e-002):2.986936e-002,(((41:2.619328e-001,42:9.817681e-002):7.564164e-002,((27:3.061816e-002,((35:7.937173e-002,22:1.551637e-002):6.077068e-003,(((39:2.948744e-002,38:9.513626e-002):5.798685e-003,2:2.052294e-002):5.073285e-003,(23:1.633787e-002,((32:5.446696e-002,(34:4.124592e-002,((29:1.581270e-002,33:4.362992e-002):2.302033e-003,28:3.782777e-002):2.256744e-002):1.869541e-002):2.085885e-002,(7:5.995805e-002,37:3.633255e-002):9.483688e-003):1.472546e-002):6.798195e-003):3.545905e-002):1.050749e-003):9.129779e-003,(((36:4.309988e-002,((25:3.499596e-004,24:3.373667e-002):1.718733e-002,((26:3.314063e-002,21:2.080589e-002):2.992072e-003,20:1.860333e-002):8.441086e-003):4.425249e-002):3.525621e-002,30:4.744042e-002):3.621975e-003,31:2.971845e-002):5.913235e-003):6.426975e-002):7.892799e-002,18:5.356402e-002):4.029478e-002):7.287137e-002):8.566893e-002):1.584627e-002,17:3.749253e-002):2.705136e-003,19:2.287244e-002,1:2.531063e-002);

tree gen.110000 = [&U] ((17:6.446561e-002,19:2.127321e-002):8.208328e-003,((((4:1.567566e-002,5:1.904109e-003):2.719092e-003,40:1.144148e-001):6.224338e-002,((9:7.770618e-003,(8:2.670358e-003,((((15:3.566051e-002,3:1.316826e-002):1.069190e-002,14:3.568353e-003):3.320239e-002,10:1.114185e-002):1.236847e-002,(13:3.438107e-003,(11:1.101363e-002,12:3.771026e-004):3.245611e-002):3.418317e-002):6.979511e-002):1.106551e-002):7.016656e-002,(((41:2.502364e-001,42:2.463731e-002):7.417389e-002,(((35:9.489771e-002,22:9.648759e-003):3.249407e-003,(31:2.660050e-002,(27:5.326200e-002,(2:1.896809e-002,((39:2.817070e-002,38:4.364529e-002):2.652429e-002,(23:7.401878e-003,((32:5.206518e-002,(34:5.770435e-002,((28:1.125497e-002,29:1.500444e-002):1.918902e-003,33:4.220999e-002):3.701438e-002):1.869612e-003):4.266131e-002,(7:3.700525e-002,37:2.772646e-002):9.598511e-003):4.702829e-003):1.573097e-002):1.687056e-002):7.494303e-002):3.076985e-003):3.247840e-004):2.201137e-003,((36:2.645272e-002,((25:3.539218e-003,24:1.462581e-002):2.122771e-002,((26:2.821052e-002,20:6.254364e-003):3.639055e-003,21:1.313775e-002):1.305143e-002):2.870177e-002):6.732704e-002,30:4.640492e-002):8.077199e-003):7.980066e-002):5.080012e-002,18:8.247142e-002):1.777356e-002):3.676779e-002):4.529786e-002,(16:3.794058e-003,6:3.003473e-002):2.645847e-002):1.076100e-002,1:1.321666e-002);

tree gen.111000 = [&U] ((19:3.191855e-002,((16:1.633265e-002,6:2.528932e-002):6.349625e-003,17:4.665973e-002):2.119407e-003):2.558795e-002,((5:5.842796e-003,(40:1.447249e-001,4:2.708652e-003):5.687284e-003):1.254919e-002,((9:7.103122e-003,(((14:2.322047e-002,(15:2.457297e-003,3:2.447710e-002):1.632319e-003):4.854611e-002,((13:1.781506e-002,(11:3.313544e-003,12:1.658239e-002):2.289173e-002):3.590604e-002,8:1.763204e-003):1.184797e-002):3.470860e-002,10:2.797409e-002):1.293612e-002):5.047108e-002,(((41:1.602669e-001,42:8.187643e-002):5.853216e-002,((31:2.893794e-002,(22:6.528769e-003,(((36:4.643937e-002,((25:6.955652e-003,24:1.169195e-002):4.668256e-002,(26:2.220483e-002,(21:7.693489e-004,20:1.378474e-004):1.548851e-002):5.816253e-003):4.565145e-002):2.714198e-002,35:6.688346e-002):8.709281e-003,((2:1.510853e-002,((39:7.506287e-002,38:6.955990e-002):1.523453e-002,(23:3.958371e-003,(((29:4.194976e-003,(28:3.498850e-002,33:3.576153e-002):1.442338e-004):2.992381e-002,(32:4.610139e-002,34:4.871647e-002):1.147566e-002):5.088821e-003,(7:4.183796e-002,37:1.510276e-002):1.961862e-002):9.782409e-003):2.068204e-002):8.970460e-003):3.414306e-002,27:4.564871e-002):2.010228e-003):1.187068e-003):1.491695e-002):3.874261e-002,30:2.891140e-002):8.134383e-002):1.927497e-002,18:2.036605e-002):3.345233e-002):4.908919e-002):4.994200e-002,1:2.604811e-002);

tree gen.112000 = [&U] (((17:7.498921e-002,((5:8.833711e-003,(4:3.224780e-003,40:1.471438e-001):9.927221e-003):8.328101e-003,((9:9.542609e-003,(8:5.364494e-003,(((14:1.370795e-002,(15:1.204207e-002,3:2.780803e-002):1.558821e-002):5.533458e-002,(13:1.341662e-002,(11:2.748042e-002,12:1.079280e-003):7.161649e-002):1.803392e-002):8.438067e-003,10:3.340285e-002):2.093428e-003):2.142047e-002):4.419898e-002,(((41:1.825842e-001,42:3.169400e-002):8.464527e-002,(27:5.360691e-002,(((((23:1.354499e-002,(((((29:7.595899e-003,28:3.871227e-002):1.248072e-002,33:2.385476e-002):2.857096e-002,32:1.448646e-001):9.335569e-003,34:2.727318e-002):1.890671e-002,(7:5.442373e-002,37:1.451727e-003):2.552137e-002):1.169313e-002):1.130876e-002,((39:7.376425e-002,38:4.722830e-002):1.026055e-002,2:1.357835e-002):4.836515e-003):1.776246e-002,30:3.571607e-002):1.146837e-002,((36:6.115783e-002,((21:4.657701e-003,(25:8.607601e-003,24:1.946117e-002):2.441003e-002):4.622126e-003,(26:6.300311e-002,20:1.350418e-003):3.508489e-003):5.200845e-002):1.992821e-002,35:3.477127e-002):1.162064e-002):6.823469e-003,(22:5.863311e-003,31:4.714511e-002):1.143399e-003):5.903197e-003):4.625440e-002):6.584873e-002,18:2.321357e-002):2.354330e-002):4.798080e-002):5.287151e-002):4.896174e-003,19:2.075186e-002):5.915943e-003,(6:2.325168e-002,16:7.139963e-003):7.520493e-003,1:4.062887e-002);

tree gen.113000 = [&U] (19:2.232986e-002,((6:3.480174e-002,16:4.385974e-002):4.617532e-003,((((5:3.067953e-003,40:5.369426e-002):1.426953e-003,4:2.283734e-002):3.256328e-002,((9:7.757454e-003,(10:5.101122e-002,(8:2.716922e-003,(((15:3.718317e-004,3:1.148447e-003):2.428567e-002,14:1.339633e-002):5.333971e-002,(13:8.978742e-003,(11:4.588496e-002,12:1.098839e-002):7.949848e-002):2.523976e-002):5.338127e-003):1.023832e-002):4.893104e-003):2.369132e-002,(((41:1.666043e-001,42:3.380781e-002):7.723708e-002,((((23:4.613758e-003,(((34:2.219937e-002,(29:1.893374e-002,(28:6.956994e-002,33:2.945849e-002):3.984723e-003):2.940347e-002):3.747931e-002,32:9.759871e-002):2.117284e-002,(7:4.754209e-002,37:4.929226e-003):5.826197e-003):1.751272e-002):3.732498e-002,2:1.238577e-002):2.954393e-003,(39:1.794344e-002,38:1.069626e-001):2.773394e-003):3.699128e-002,(((((36:1.453294e-002,((26:6.026220e-002,((25:1.112670e-002,24:2.485139e-003):1.200543e-002,20:1.982284e-003):4.561506e-003):5.878495e-003,21:3.011217e-002):4.766493e-002):2.997118e-002,35:3.747821e-002):4.513756e-003,30:1.374854e-002):2.530690e-002,(22:2.160812e-002,27:4.891520e-002):1.240180e-004):3.000116e-003,31:4.022282e-002):1.895096e-002):6.329935e-002):1.011284e-001,18:1.955632e-002):3.354500e-002):5.368808e-002):5.496143e-002,17:7.826690e-002):3.581133e-003):4.107563e-003,1:5.134975e-002);

tree gen.114000 = [&U] ((17:6.671320e-002,(((4:1.521678e-002,40:4.182546e-002):1.451441e-003,5:1.928491e-002):2.834427e-002,((9:6.089041e-003,((10:1.752963e-002,((14:1.498545e-002,(15:3.034802e-004,3:3.700192e-006):1.710317e-002):2.250738e-002,(13:7.435300e-003,(11:3.468265e-003,12:1.284289e-002):6.240056e-002):2.741791e-002):5.587001e-002):1.014554e-003,8:1.828797e-002):1.453302e-002):2.072922e-002,(18:3.402927e-002,((41:1.307723e-001,42:2.653669e-002):1.239687e-001,(((22:1.078169e-002,(31:3.309381e-002,((27:7.983328e-002,35:5.649476e-002):6.859720e-003,(36:1.741487e-002,((26:4.730147e-002,(20:3.555398e-003,21:2.312621e-002):2.460059e-002):3.779008e-002,(25:8.603937e-003,24:3.152550e-003):2.294408e-003):3.701374e-002):6.813937e-002):2.278917e-002):2.101049e-003):8.808536e-003,((23:4.316788e-003,(((32:4.600878e-002,34:5.709467e-003):9.369445e-003,((33:2.299511e-002,29:1.073353e-002):2.836084e-003,28:2.247169e-002):2.310406e-002):1.949325e-002,(7:6.455759e-002,37:3.268606e-002):1.247707e-002):5.769951e-003):9.998210e-003,(2:2.094309e-002,(39:1.408430e-002,38:8.024797e-002):5.086843e-002):2.295170e-003):1.800511e-002):6.407319e-006,30:4.133476e-003):5.071378e-002):5.460916e-002):1.888369e-002):7.893659e-002):3.229161e-002):2.101801e-002,(16:8.889613e-003,(6:2.731133e-002,19:9.365986e-003):5.584845e-003):1.797276e-002,1:3.631281e-002);

tree gen.115000 = [&U] (17:7.563513e-002,((((5:4.474269e-003,4:9.722032e-003):1.094933e-002,40:4.474727e-002):2.657829e-002,((9:9.837973e-003,(8:1.502362e-002,(10:4.337535e-002,(((15:1.952036e-004,3:5.909497e-003):9.410576e-003,14:1.731989e-002):4.859287e-003,(13:1.722241e-002,(11:3.675029e-002,12:1.161811e-003):4.998175e-002):8.076440e-003):4.073188e-002):2.206232e-002):3.955448e-002):2.898353e-002,(18:1.010242e-001,((41:1.369320e-001,42:3.067058e-002):4.006419e-002,((((39:5.104245e-002,38:2.261643e-002):7.942387e-003,((23:2.530598e-003,(((34:4.017649e-002,((28:3.773673e-002,33:2.618501e-002):5.595748e-003,29:1.358884e-002):2.357442e-002):7.744027e-003,32:5.127509e-002):3.014430e-002,(7:7.461439e-002,37:3.423260e-002):2.059332e-002):1.128144e-002):2.420009e-002,2:4.548809e-002):7.604129e-003):2.913396e-002,(((36:4.757969e-002,(26:5.369455e-002,(((25:9.725823e-003,24:5.149584e-003):1.758288e-002,21:6.731329e-004):4.842898e-003,20:1.058435e-002):2.410228e-003):2.897318e-002):1.532465e-002,35:4.178855e-002):8.700760e-003,31:2.576684e-002):4.147580e-003):8.910576e-003,(30:2.010536e-002,(22:1.780005e-002,27:1.055822e-001):4.092559e-003):1.004064e-002):5.166134e-002):4.578195e-002):2.479838e-002):4.915793e-002):6.665964e-002,((19:2.615026e-002,16:4.487922e-003):4.783923e-003,6:1.581429e-002):3.066819e-002):6.249481e-003,1:2.804748e-002);

tree gen.116000 = [&U] (((((40:3.278379e-002,(5:7.387926e-003,4:1.871889e-002):5.603879e-003):2.766199e-002,((9:8.162545e-003,(10:3.455413e-002,(8:2.791071e-002,(((14:4.109048e-003,15:6.849531e-003):2.089587e-003,3:5.690289e-003):3.491007e-002,(13:8.818088e-003,(11:1.563541e-002,12:5.480814e-004):3.188769e-002):2.337187e-002):6.010498e-002):2.735108e-003):7.685437e-003):2.660235e-002,(18:1.617424e-002,((41:1.176667e-001,42:3.829633e-002):9.063848e-002,((((36:2.670497e-002,((20:3.255432e-003,((25:2.901052e-002,24:5.204299e-003):3.195353e-002,26:2.184391e-002):4.406002e-003):3.171539e-004,21:1.053020e-002):2.775996e-002):2.091548e-002,35:3.685287e-002):1.791069e-002,((2:4.491147e-002,(23:2.454913e-002,(((((28:3.768335e-002,29:1.041786e-002):6.336046e-003,33:1.628869e-002):1.841145e-002,32:5.336546e-002):3.753403e-002,34:5.401861e-002):2.668165e-002,(7:7.540718e-002,37:8.324935e-003):3.258287e-002):2.004276e-003):9.217967e-003):5.350017e-003,(39:1.895352e-002,38:1.897415e-002):1.129529e-002):2.706649e-002):1.457892e-002,((31:3.357360e-002,22:1.904755e-002):8.532452e-003,(30:2.858209e-002,27:1.713920e-002):6.072113e-003):3.936989e-005):4.779756e-002):4.007649e-002):7.689034e-003):5.603688e-002):4.270505e-002,17:7.856393e-002):4.599769e-003,(16:2.141809e-002,6:1.314792e-002):3.455768e-002):9.645986e-003,19:3.158079e-002,1:4.539640e-002);

tree gen.117000 = [&U] (17:1.100493e-001,((16:1.375192e-002,(6:4.147680e-002,19:3.292006e-002):2.953217e-003):2.340979e-002,((4:2.570463e-002,(40:1.041909e-001,5:1.225092e-002):3.544007e-003):4.412939e-002,((9:9.656576e-003,((10:4.602828e-002,(((15:8.953935e-003,14:8.676864e-003):5.954813e-003,3:1.600433e-002):2.765896e-002,(13:5.889159e-003,(11:8.933735e-003,12:3.205065e-003):3.797127e-002):6.144115e-003):7.441521e-003):1.114519e-002,8:1.049166e-002):9.662149e-004):1.419441e-002,(18:2.114350e-002,((41:2.025713e-001,42:1.882174e-002):4.525243e-002,((30:4.050048e-002,((27:1.827389e-002,(2:1.113013e-002,((39:4.151460e-002,38:5.645315e-002):3.244809e-003,(23:1.114777e-003,(((32:7.541988e-002,34:1.121391e-001):1.136599e-002,((33:1.596083e-002,28:2.875060e-002):1.380207e-002,29:1.361858e-002):2.297113e-002):2.697573e-002,(7:2.926936e-002,37:9.391494e-003):3.850178e-003):3.068923e-002):7.330637e-002):2.945503e-003):5.648563e-002):2.205600e-002,(((36:1.613301e-002,(((26:2.207061e-002,(25:1.072793e-002,24:2.786959e-003):1.872506e-002):1.198425e-002,21:4.998825e-003):9.955106e-003,20:1.745556e-002):1.367754e-002):3.491599e-002,35:3.846495e-002):2.752654e-002,22:1.428470e-002):4.200406e-002):2.125548e-002):1.424510e-005,31:3.960082e-002):7.738195e-002):4.874534e-002):1.012284e-002):3.623645e-002):6.184421e-002):3.881938e-002,1:9.042196e-002);

tree gen.118000 = [&U] ((((4:3.256537e-002,5:6.142267e-003):1.819847e-002,40:1.232218e-001):5.148179e-002,((9:9.158743e-003,(8:1.395021e-002,((13:2.827019e-002,(11:2.676618e-002,12:9.324740e-003):5.221358e-002):3.863336e-004,(10:6.084240e-002,(14:6.822421e-003,(3:1.112933e-002,15:4.293399e-002):3.038966e-002):1.675199e-002):5.895261e-002):5.405046e-003):3.182126e-002):3.756340e-002,(18:4.179570e-002,((41:2.582729e-001,42:2.824077e-002):8.370701e-002,(((27:1.895657e-002,31:5.071999e-002):1.486117e-002,((36:2.598606e-002,(20:4.896611e-003,((21:3.070004e-002,26:2.951384e-002):1.811640e-002,(25:2.493358e-002,24:3.553299e-003):7.824087e-003):1.494329e-003):1.777176e-002):2.335515e-002,35:6.307420e-002):3.770405e-002):2.286300e-004,(22:5.248913e-002,(30:2.430665e-002,((39:5.354248e-002,2:4.208656e-002):3.273535e-002,((23:1.534757e-002,((((33:1.909652e-002,28:3.254953e-002):1.007202e-002,29:7.343025e-003):3.242072e-002,(32:9.615833e-002,34:5.684042e-002):3.462715e-002):4.177560e-002,(7:5.181429e-002,37:3.029497e-004):9.911420e-003):5.610560e-003):8.751074e-002,38:1.156798e-001):1.343296e-002):9.370998e-003):1.123456e-002):9.970112e-003):8.167912e-002):6.760185e-002):1.515222e-002):4.063707e-002):6.584939e-002,(((19:6.369070e-002,16:1.870833e-002):4.013714e-003,6:9.069739e-003):8.387436e-003,17:8.348518e-002):8.620050e-003,1:1.978991e-002);

tree gen.119000 = [&U] ((6:1.213184e-002,16:4.072467e-002):1.208425e-002,((17:9.974130e-002,((40:1.320112e-001,(5:1.669093e-003,4:1.084376e-002):3.013358e-002):2.254941e-002,(((8:1.055310e-002,(((13:1.571608e-002,(11:4.298064e-002,12:9.141340e-003):8.519875e-002):2.680378e-002,(14:1.251497e-003,(3:3.153099e-002,15:1.779041e-002):2.455973e-002):3.651377e-002):2.315256e-002,10:7.320567e-002):4.508563e-003):1.324591e-002,9:1.226325e-002):2.263114e-002,(18:9.337279e-002,((41:2.960344e-001,42:6.610742e-002):9.594561e-002,(((((39:7.682568e-003,38:3.640711e-002):4.310493e-002,2:9.020569e-002):6.552710e-003,(23:1.369092e-002,((34:4.540539e-002,(((29:8.700534e-003,28:4.432899e-002):1.041437e-002,33:1.839990e-002):2.480892e-002,32:1.102174e-001):2.969736e-004):5.975723e-002,(7:6.103457e-002,37:1.899957e-003):2.914478e-002):4.805123e-002):2.106447e-002):8.236783e-002,27:3.216880e-002):1.124756e-002,(((30:1.141687e-002,31:9.869477e-003):2.716389e-002,22:3.314223e-002):2.487390e-002,((36:2.224998e-002,((26:3.764829e-003,(21:3.505973e-003,(25:2.574323e-003,24:4.727488e-002):3.509282e-002):1.261192e-002):7.662613e-003,20:5.704558e-003):1.924031e-002):2.999375e-002,35:5.841453e-002):2.031588e-002):1.834791e-003):1.217806e-001):8.530410e-002):2.143268e-002):4.360145e-002):1.137913e-001):2.126847e-003,19:1.099549e-002):2.057084e-002,1:1.249316e-001);

tree gen.120000 = [&U] (((((4:1.351567e-002,5:1.744145e-003):4.843694e-003,40:1.148776e-001):7.671111e-002,((((8:3.207813e-003,((14:4.733863e-003,(3:2.779162e-002,15:1.816693e-002):1.548123e-003):6.882908e-002,(13:1.549115e-002,(11:1.240931e-002,12:2.743819e-002):3.508925e-002):4.447087e-002):2.268532e-002):6.248719e-003,10:3.344404e-002):2.094519e-002,9:1.220434e-002):2.941794e-002,(18:4.698916e-002,((41:2.792867e-001,42:5.885912e-002):1.504990e-001,(((((36:5.118115e-002,(21:3.223330e-003,((20:4.502190e-003,(25:1.368853e-002,24:1.477249e-002):1.376165e-002):1.644484e-002,26:3.863291e-002):2.230766e-002):2.166388e-002):3.819234e-002,35:5.200971e-002):1.631610e-002,(30:4.053938e-002,(((23:1.542908e-002,((((33:1.269407e-002,(29:7.294946e-003,28:3.946857e-002):3.043323e-002):3.362195e-002,32:7.992227e-002):4.867860e-002,34:4.278911e-002):2.063029e-002,(7:5.434248e-002,37:6.944093e-003):2.500852e-002):5.711836e-003):4.091982e-003,2:2.787768e-002):1.877807e-003,(39:2.592561e-002,38:3.241528e-002):4.121406e-002):9.927411e-002):1.960963e-002):8.166812e-004,31:1.430807e-002):1.533714e-002,(27:6.858612e-002,22:8.200854e-003):4.919497e-003):1.252703e-001):7.799966e-002):5.747809e-003):3.947761e-002):3.953434e-002,(6:2.307769e-002,(16:3.953133e-002,19:2.508905e-002):1.382269e-002):2.758981e-002):2.159855e-002,17:8.570989e-002,1:1.097730e-001);

tree gen.121000 = [&U] (17:5.511367e-002,((((5:4.779891e-003,40:7.198611e-002):7.037137e-003,4:2.332540e-002):7.423445e-002,((((10:2.971495e-002,(13:9.389992e-003,(11:6.628607e-003,12:2.283128e-002):5.889960e-002):1.051122e-002):1.787062e-005,(8:2.669216e-003,(14:9.079880e-004,(15:3.507093e-002,3:7.351776e-004):7.272282e-003):6.138750e-002):2.560766e-003):1.809699e-002,9:1.735415e-002):7.766415e-002,(18:3.909962e-002,((41:2.323940e-001,42:4.201380e-002):1.105136e-001,(((27:3.488522e-002,(31:8.326661e-002,30:3.743424e-002):1.875324e-002):6.263637e-003,(((36:2.098617e-002,(((25:9.402934e-003,24:2.500982e-003):1.524096e-002,26:6.004451e-003):1.242684e-002,(20:1.398977e-002,21:1.103086e-002):6.092257e-002):3.473464e-002):3.318588e-002,35:4.327720e-002):3.822290e-002,(2:4.604729e-003,((39:3.597157e-002,38:6.998310e-002):4.934914e-002,(23:3.901286e-003,((((33:1.776865e-002,(29:5.175920e-002,28:4.316533e-002):7.652808e-003):2.380016e-002,34:3.344998e-002):2.229051e-003,32:5.170764e-002):1.922122e-002,(7:5.061168e-002,37:4.665328e-002):4.020799e-002):2.678570e-002):3.497582e-002):5.764186e-003):4.744193e-002):6.312427e-004):2.877314e-003,22:1.226791e-003):1.684821e-001):6.377754e-002):2.552218e-002):3.083836e-002):3.657445e-002,((6:2.605858e-002,16:1.600129e-002):1.223724e-002,19:3.265034e-002):2.336267e-002):5.823784e-003,1:8.226853e-002);

tree gen.122000 = [&U] ((16:1.983098e-002,6:1.908550e-002):5.701058e-002,((((4:1.904543e-002,(5:3.245990e-003,40:1.347887e-001):8.166121e-003):5.337645e-003,((((((15:3.062278e-003,3:2.039192e-002):3.689844e-002,14:5.007106e-003):8.970771e-003,(10:6.881411e-003,((13:4.637491e-002,(11:4.990196e-002,12:1.053785e-002):7.751679e-002):1.064244e-002,8:1.052044e-003):1.785695e-002):1.297056e-002):1.316765e-002,9:5.039637e-002):5.859447e-002,18:3.306872e-002):5.255301e-002,((41:2.112181e-001,42:1.018780e-001):1.424958e-001,(((22:6.368861e-002,(30:1.875547e-002,(((36:6.655420e-003,((25:6.583978e-003,24:5.208894e-003):2.195151e-002,(26:4.242609e-002,(20:6.298668e-004,21:1.820334e-002):2.743747e-003):1.617629e-003):6.665778e-002):2.002498e-002,35:3.500891e-002):1.142071e-002,(((23:7.937581e-003,((34:4.198055e-002,((29:2.152699e-002,(33:2.163340e-002,28:3.349869e-002):4.280337e-003):1.472173e-002,32:3.953748e-002):4.292457e-002):7.043089e-002,(7:6.525851e-002,37:5.889202e-002):3.447834e-002):4.178932e-003):4.109525e-002,(39:4.944582e-003,38:9.023594e-002):3.532984e-002):4.576035e-003,2:1.617721e-002):3.689353e-002):2.516010e-003):1.437845e-002):5.956046e-002,27:1.159540e-001):2.491688e-002,31:9.945456e-002):3.778783e-002):8.912539e-002):3.976287e-002):1.334095e-001,19:9.090219e-002):3.094597e-003,17:6.686169e-002):8.926373e-003,1:5.060882e-002);

tree gen.123000 = [&U] (((4:2.083795e-002,(5:1.121690e-002,40:1.325978e-001):6.136237e-003):6.051581e-002,((18:6.785670e-002,((41:2.232253e-001,42:8.291406e-002):1.696129e-001,(27:9.114491e-002,((((36:9.395892e-003,(21:3.338843e-003,((25:1.956758e-004,24:7.837296e-003):1.304160e-002,(26:1.253110e-001,20:2.681364e-003):4.286453e-004):3.036690e-002):5.548053e-002):3.704369e-002,35:9.165157e-002):1.981514e-002,((31:1.051083e-001,((39:7.811217e-002,38:1.505535e-001):2.056207e-002,((23:4.354143e-002,((34:7.048091e-002,(32:1.636334e-001,(33:1.880145e-002,(28:3.962992e-002,29:3.296705e-003):5.313343e-003):9.732770e-002):2.439363e-002):2.114339e-002,(7:6.864427e-002,37:5.254734e-003):3.025765e-002):2.084008e-002):3.068700e-002,2:1.371176e-002):2.173484e-002):4.147736e-002):2.263941e-002,30:2.227439e-002):3.368563e-002):8.621592e-005,22:1.941045e-002):3.225332e-002):9.508296e-002):5.326111e-002):7.671070e-003,((((15:3.226267e-003,(3:5.668917e-003,14:1.457062e-002):1.558665e-002):7.844109e-002,10:2.780789e-002):3.094939e-002,(8:1.333265e-003,(13:3.378669e-002,(11:2.654089e-002,12:1.113690e-002):8.192342e-002):3.450732e-003):1.927448e-003):1.208170e-002,9:1.416670e-002):6.999405e-002):6.603313e-002):1.355448e-001,(17:8.141430e-002,(19:8.707428e-003,(16:1.014211e-002,6:4.064182e-002):1.717042e-003):4.097444e-002):1.466679e-002,1:4.601187e-002);

tree gen.124000 = [&U] (((6:4.791487e-002,16:2.821743e-003):3.803274e-002,(17:3.668149e-002,19:2.635945e-002):1.148737e-002):1.674187e-002,(((4:2.592877e-002,40:1.039597e-001):1.518137e-002,5:5.004536e-003):1.040893e-001,((18:1.629101e-001,((41:4.074378e-001,42:9.775193e-002):1.371364e-001,((2:8.180375e-003,((23:1.207085e-002,((((29:1.647566e-002,28:6.252040e-002):3.288618e-003,33:8.234913e-003):5.606515e-002,(32:1.582593e-001,34:6.632484e-002):3.598126e-002):8.205986e-002,(7:6.383398e-002,37:4.669465e-003):8.189004e-002):8.088467e-003):1.455959e-002,(39:7.904966e-002,38:1.768270e-001):3.023518e-002):1.201022e-002):6.614334e-002,(30:2.758350e-002,(((36:4.683719e-002,(20:6.500122e-004,((21:1.017525e-003,26:8.383874e-003):1.141907e-002,(25:4.599880e-003,24:2.477468e-002):2.786271e-002):1.258569e-003):2.509740e-002):6.055403e-002,35:5.302114e-002):5.430671e-002,((31:1.648332e-001,27:9.369218e-002):8.796334e-004,22:2.134222e-002):1.154521e-002):1.442247e-002):8.476200e-003):1.215805e-001):1.730952e-001):8.387144e-003,(((((13:2.686276e-003,(11:3.129050e-002,12:1.312990e-002):1.511836e-001):6.177324e-002,8:4.132911e-004):9.810757e-003,((14:2.256884e-002,15:1.017973e-002):1.499304e-003,3:2.558869e-003):8.972104e-002):2.936391e-003,10:5.211710e-002):2.184420e-002,9:9.693199e-004):4.973308e-002):5.530439e-002):1.513879e-001,1:5.696148e-002);

tree gen.125000 = [&U] ((6:3.977209e-002,16:1.543500e-002):1.366485e-002,((17:1.103135e-001,((5:4.594391e-003,(4:1.190026e-002,40:9.779431e-002):1.551008e-002):2.211316e-002,((18:3.078110e-002,((41:3.829117e-001,42:4.166148e-002):1.288814e-001,(30:2.468045e-002,(22:2.353367e-002,(27:7.682725e-002,(31:1.480332e-001,(((2:8.810465e-002,(23:2.427450e-002,(((34:8.843269e-002,((33:7.964698e-003,28:4.591624e-002):1.543807e-003,29:1.163919e-002):7.386469e-002):1.834569e-002,32:1.487327e-001):5.561589e-003,(7:5.357814e-002,37:1.156367e-001):1.082961e-002):2.495061e-002):6.450719e-002):7.214558e-002,(39:2.430468e-002,38:1.661828e-001):8.898446e-002):4.099490e-002,((36:6.548185e-002,((26:2.295074e-002,(21:1.334800e-002,20:3.711446e-003):1.250014e-002):8.501700e-003,(25:1.215858e-003,24:3.023891e-003):5.996770e-003):7.909134e-003):4.553121e-002,35:4.730440e-002):1.654213e-002):7.273585e-003):3.007024e-002):4.412090e-003):2.844104e-002):1.347069e-001):5.507546e-002):1.950497e-002,((((13:2.890979e-002,(11:3.943576e-002,12:2.011205e-002):1.294083e-001):2.053537e-002,8:1.430062e-002):1.177302e-002,(10:6.267540e-002,(14:2.088824e-002,(3:2.738408e-002,15:1.665876e-002):4.758208e-002):3.537224e-002):1.055131e-001):9.878320e-003,9:1.317243e-002):8.713575e-002):6.525467e-002):1.164502e-001):4.207358e-003,19:2.104718e-002):4.937507e-003,1:5.861705e-002);

tree gen.126000 = [&U] ((((4:1.107310e-002,5:1.144701e-002):1.357839e-003,40:1.210165e-001):3.638366e-002,((18:7.753545e-002,((41:3.686722e-001,42:1.167414e-002):1.658706e-001,(30:8.844478e-003,(((31:7.495224e-002,((36:1.178571e-002,((21:2.824733e-003,(25:1.468885e-002,24:1.056860e-002):3.441619e-002):5.906778e-002,(20:3.646563e-003,26:4.790753e-002):2.885540e-003):2.534721e-002):5.047509e-002,35:4.885438e-002):1.698058e-002):4.529500e-003,(27:7.585882e-002,22:1.836702e-002):3.563464e-002):3.053672e-002,(((23:7.943400e-002,(((32:1.432017e-001,(33:3.084716e-002,(28:4.995924e-002,29:3.437317e-003):4.794195e-003):7.464106e-002):7.271716e-003,34:8.585583e-002):2.294922e-002,(7:5.207895e-002,37:2.183317e-002):4.126487e-002):3.553326e-002):1.636073e-002,(38:1.519199e-001,39:5.078392e-002):2.647918e-002):2.193790e-003,2:9.528868e-003):9.673144e-002):6.571925e-003):7.603899e-002):6.309728e-002):6.397151e-002,(((13:3.115212e-002,(11:4.142373e-002,12:5.160811e-003):9.877115e-002):4.484173e-003,((10:1.238102e-001,((3:2.597223e-002,15:5.335537e-002):1.147077e-002,14:1.367514e-002):1.430780e-001):2.636721e-003,8:6.435840e-003):2.693365e-002):2.516518e-002,9:1.268259e-002):7.511328e-002):7.389459e-002):1.019680e-001,(17:1.857924e-002,(19:1.727299e-002,(6:1.997870e-002,16:3.645577e-003):1.314120e-002):1.613427e-003):4.493541e-004,1:3.507114e-002);

tree gen.127000 = [&U] ((((((5:2.091062e-002,40:1.109709e-001):9.138825e-002,4:3.366004e-002):2.658660e-002,((18:2.917356e-003,((41:3.652544e-001,42:1.914619e-001):2.502898e-001,((27:7.552272e-002,31:7.422393e-002):2.005968e-002,(35:6.569496e-002,(((30:6.282792e-002,(((23:3.904044e-002,(((34:8.043632e-002,32:6.255682e-002):5.017897e-004,((28:5.487014e-002,29:1.746145e-002):3.185032e-003,33:5.652802e-002):8.891363e-002):1.690106e-002,(7:9.525835e-002,37:1.407643e-002):2.802749e-002):2.279583e-002):5.715967e-002,2:8.244007e-003):1.019522e-002,(38:9.623631e-002,39:5.031313e-002):4.023768e-002):6.900606e-002):1.341962e-002,(36:5.135742e-002,((25:3.266283e-002,24:1.561039e-002):4.750845e-002,((20:2.190740e-002,21:1.398266e-002):4.289311e-003,26:6.353258e-002):3.690885e-003):2.450280e-002):4.430147e-002):9.745880e-003,22:1.613741e-002):1.405358e-003):9.577504e-003):1.749578e-001):1.124041e-001):4.435213e-003,((10:6.528247e-002,((((14:5.239029e-002,3:2.728256e-002):1.208148e-002,15:2.196530e-002):9.180135e-002,(13:1.511380e-002,(11:9.714587e-002,12:1.714971e-002):5.905070e-002):2.773085e-002):1.068239e-002,8:1.541648e-002):3.694391e-003):1.354439e-002,9:3.353696e-002):6.822397e-002):9.007739e-002):9.828939e-002,19:3.449363e-002):4.239265e-002,17:1.323387e-001):4.603289e-004,(6:2.490626e-002,16:1.527543e-002):3.800594e-002,1:5.998341e-002);

tree gen.128000 = [&U] ((19:3.387489e-002,17:1.399707e-001):2.522372e-002,((((4:1.532277e-002,5:5.453560e-002):3.806346e-003,40:1.337133e-001):1.138843e-001,((18:9.479461e-002,((41:2.344932e-001,42:1.991599e-001):2.260988e-001,((36:7.178115e-002,(26:4.795220e-002,((21:2.941775e-002,20:1.261128e-003):1.407024e-002,(25:1.321670e-002,24:1.623803e-002):4.324376e-002):9.628765e-003):6.319931e-002):9.228256e-002,(31:7.424229e-002,(((((23:3.659003e-003,(((32:6.911545e-002,((29:3.189030e-002,33:5.239288e-002):3.439829e-003,28:1.602808e-002):1.994792e-002):2.360087e-002,34:3.925558e-002):5.674718e-002,(7:1.512047e-001,37:6.227044e-002):1.657966e-002):1.976948e-002):5.894975e-002,(38:1.049194e-001,39:1.939249e-002):3.999461e-002):2.557112e-003,2:2.690838e-002):1.089423e-001,30:3.008692e-002):1.645846e-003,((27:6.104722e-002,35:7.703855e-002):5.255933e-003,22:8.047536e-002):8.617897e-003):1.032890e-002):2.365510e-003):2.138614e-001):6.658121e-002):3.722893e-002,(((13:3.230766e-003,(11:2.265185e-002,12:1.783924e-002):7.433130e-002):6.394953e-002,(((14:5.679327e-003,(15:3.860915e-002,3:8.834139e-003):1.781972e-002):5.855232e-002,10:6.894053e-002):6.072941e-002,8:2.077085e-002):3.878006e-002):2.109399e-002,9:1.824270e-002):8.465594e-002):6.005704e-002):8.306340e-002,(16:1.588960e-002,6:4.843199e-002):1.015190e-002):4.178699e-002,1:1.023871e-001);

tree gen.129000 = [&U] (((19:5.873898e-002,(6:5.826222e-002,16:9.351095e-003):1.129110e-002):4.047535e-002,((5:7.612737e-003,(40:1.619584e-001,4:4.265484e-002):3.156173e-003):7.597780e-003,((18:1.332299e-001,((41:5.646307e-001,42:1.100328e-001):2.909692e-001,((((27:5.887866e-002,22:8.693916e-003):4.263463e-002,((36:4.157844e-002,(21:1.723627e-002,((26:4.760033e-002,(25:1.146607e-002,24:1.848159e-002):3.037716e-002):1.016764e-002,20:1.981686e-002):2.143707e-002):7.327363e-002):8.864775e-002,35:7.879498e-002):1.641396e-002):2.769334e-002,((2:1.609532e-002,(38:9.019430e-002,39:2.416872e-002):3.410015e-002):2.048937e-002,(23:4.440781e-003,(((34:6.160313e-002,32:2.640241e-001):2.840462e-002,(33:6.185038e-002,(28:3.371506e-002,29:4.791945e-002):1.542663e-002):4.196562e-002):3.777906e-002,(7:1.450860e-001,37:9.343106e-002):1.721124e-002):2.554204e-002):4.272061e-002):5.871044e-002):8.863603e-003,(31:1.744081e-001,30:3.496791e-002):1.117123e-002):1.813478e-001):6.523161e-002):5.561552e-002,(((8:2.180458e-002,(13:3.042377e-003,(11:2.299680e-002,12:1.711735e-002):6.118215e-002):4.354016e-002):3.587010e-002,(10:3.235637e-002,(3:1.667828e-002,(14:2.226212e-002,15:3.704678e-002):1.049321e-002):3.959167e-002):8.415936e-002):1.591779e-002,9:5.113266e-002):8.423903e-002):5.762675e-002):1.066226e-001):3.710210e-002,17:1.078922e-001,1:1.238942e-001);

tree gen.130000 = [&U] ((16:3.556632e-002,(19:4.352075e-002,6:7.965956e-002):3.169357e-002):7.815455e-002,(17:2.275191e-001,(((40:1.645778e-001,5:2.153621e-002):9.105708e-004,4:1.255588e-002):5.462426e-002,((18:7.646183e-002,((41:4.088279e-001,42:1.237453e-001):1.528582e-001,((35:9.918061e-002,(36:7.167774e-002,(((25:4.365741e-003,24:8.643644e-003):2.398354e-002,(20:9.324035e-003,26:1.193536e-001):4.364309e-003):5.728381e-002,21:1.810596e-002):5.290284e-002):1.844968e-001):7.270003e-003,(((31:2.959650e-002,(27:9.220315e-002,((2:3.608821e-002,(38:2.415708e-001,39:4.903227e-002):1.614788e-002):2.281062e-002,(23:4.552711e-003,((32:2.768691e-001,(34:7.641139e-002,((28:4.463921e-002,33:5.010412e-002):7.499482e-003,29:6.410970e-002):7.555262e-002):1.260902e-002):4.083463e-002,(7:8.675221e-002,37:9.200743e-002):7.669873e-002):6.721944e-003):3.351634e-002):3.942391e-002):8.040922e-004):5.016437e-002,22:9.976038e-003):5.780005e-003,30:4.414546e-002):5.476781e-002):2.224753e-001):8.864402e-002):1.200225e-002,((8:3.702435e-002,(10:4.127177e-002,((13:9.958502e-003,(11:4.379196e-003,12:2.256041e-002):1.822666e-001):2.588026e-002,(15:2.955210e-002,(14:7.425947e-003,3:5.447708e-002):4.387479e-002):9.242754e-002):4.171643e-002):2.225233e-002):4.992299e-002,9:7.010655e-002):7.207696e-002):2.453241e-001):1.261311e-001):3.359063e-002,1:7.878156e-002);

tree gen.131000 = [&U] ((((5:8.624997e-003,4:6.201677e-002):8.581350e-003,40:2.191942e-001):1.060975e-001,((18:1.018363e-001,((41:5.445006e-001,42:1.479573e-001):2.035853e-001,((((38:1.107114e-001,39:1.773835e-001):1.289202e-002,(2:4.806436e-002,(23:2.684008e-002,((34:9.815836e-002,(32:5.692457e-002,(33:3.671686e-002,(28:5.919898e-002,29:8.975417e-002):7.027907e-002):5.547205e-002):4.620185e-002):1.332141e-002,(7:1.627288e-001,37:5.647085e-003):5.157174e-002):8.968940e-002):2.254096e-002):3.360770e-002):1.077698e-001,(((22:1.129297e-002,31:3.941832e-002):1.952203e-002,(35:1.118844e-001,(36:2.245842e-001,((((25:8.950312e-002,24:1.912654e-002):1.340615e-001,20:2.794389e-002):2.190934e-002,26:1.430544e-001):6.842716e-004,21:4.420935e-004):9.685108e-002):7.325850e-002):3.482359e-002):3.243421e-002,30:3.332092e-002):9.598964e-002):4.127752e-003,27:1.203941e-001):1.236165e-001):1.100823e-001):4.703313e-002,(((14:6.119956e-003,(3:4.302346e-002,15:2.463119e-002):4.384085e-002):7.632330e-002,((9:3.863973e-002,10:9.822233e-002):9.579072e-003,8:2.204367e-002):1.304681e-001):8.251611e-003,(13:2.701807e-003,(11:9.193000e-002,12:2.468189e-003):1.691208e-001):6.960340e-002):6.091316e-002):1.921071e-001):2.938706e-001,((19:4.489994e-002,(6:1.025263e-001,16:1.030166e-002):5.729925e-002):1.845344e-002,17:1.564502e-001):1.856536e-002,1:1.363939e-001);

tree gen.132000 = [&U] (((6:7.237798e-002,16:1.126631e-001):8.172094e-003,(((5:6.906163e-002,40:4.983520e-001):3.325417e-002,4:8.125643e-002):1.146890e-001,((18:1.020929e-001,(9:5.062706e-002,((8:2.888236e-002,(13:9.104968e-003,(11:6.618801e-002,12:4.290444e-002):1.704050e-001):3.867193e-002):3.508257e-002,(10:4.009186e-002,((15:7.150523e-003,3:9.204251e-004):1.340599e-001,14:1.626634e-002):1.422259e-001):2.426690e-002):7.596081e-002):1.300256e-001):6.326305e-002,((41:7.134228e-001,42:2.623764e-001):2.253168e-001,(31:6.739179e-002,(((((38:1.420059e-001,39:1.200269e-002):1.318797e-001,(23:1.014520e-002,((34:2.906567e-001,(((28:7.680480e-002,29:5.575616e-002):2.519940e-002,33:4.805040e-002):1.673270e-001,32:1.749070e-001):1.660418e-003):7.598980e-002,(7:1.911214e-001,37:2.142441e-002):9.289858e-002):5.174127e-002):4.599923e-002):2.539602e-002,2:6.297552e-002):2.959155e-002,30:3.271779e-002):4.872610e-002,(27:1.685845e-001,((35:1.465947e-001,(36:1.688209e-002,(20:7.661814e-002,((21:2.683852e-002,26:1.458175e-001):2.323725e-002,(25:5.440973e-002,24:4.479695e-003):4.129240e-002):3.248335e-002):1.162121e-001):1.198834e-001):9.100170e-003,22:1.171423e-001):1.857702e-001):6.867230e-002):1.520477e-003):2.127378e-001):1.536030e-001):1.346507e-001):9.405128e-002):2.473248e-002,(17:8.432040e-002,19:3.941229e-002):2.568558e-003,1:1.103265e-001);

tree gen.133000 = [&U] (((6:2.540362e-002,16:3.847282e-002):4.271710e-002,19:2.747263e-002):3.773470e-002,(17:2.111429e-001,((5:1.170131e-002,(40:2.724753e-001,4:5.508591e-002):8.925592e-003):9.818893e-002,((18:9.523417e-002,((41:5.846061e-001,42:4.745871e-002):3.099164e-001,((((2:5.160457e-002,(38:1.709597e-001,39:2.977702e-002):8.482913e-002):2.856655e-002,(23:8.313365e-003,((32:1.685643e-001,((33:3.304038e-002,(28:9.055360e-002,29:4.006039e-002):4.012022e-002):1.035624e-001,34:3.148483e-001):1.091913e-002):8.067924e-002,(7:1.332859e-001,37:1.755598e-002):7.048913e-002):5.450033e-002):4.795803e-002):4.780387e-002,(30:2.685988e-002,27:1.381446e-001):1.079615e-002):2.340017e-002,(((35:5.915967e-002,(36:8.483435e-002,(26:1.219959e-001,((20:4.754031e-002,(25:1.042890e-002,24:7.208133e-004):1.247719e-001):6.357554e-003,21:7.379051e-004):2.517212e-003):2.583262e-002):6.801271e-002):3.062257e-002,22:2.449979e-002):1.471826e-001,31:5.146202e-002):2.673872e-002):1.894135e-001):6.543358e-002):1.063968e-001,(9:1.411026e-002,(8:9.603900e-003,((10:1.196375e-001,(13:9.965346e-003,(11:5.423701e-002,12:8.703276e-003):1.396364e-001):7.101309e-002):9.735897e-003,((3:4.608639e-002,15:1.708208e-002):9.847043e-002,14:1.269605e-002):2.158992e-002):1.296019e-001):4.989714e-002):6.080932e-002):9.842485e-002):2.279477e-001):1.784487e-002,1:1.179383e-001);

tree gen.134000 = [&U] (((((5:3.400608e-002,4:4.053529e-002):1.863114e-002,40:1.545009e-001):4.969477e-003,((18:1.067685e-001,((41:5.071723e-001,42:3.543338e-002):2.688665e-001,(((31:4.231586e-002,22:3.357794e-002):2.421943e-002,(((35:3.378747e-002,(36:7.359765e-002,(((20:3.369923e-003,(25:3.857992e-003,24:4.442818e-002):3.677368e-002):1.007546e-002,21:9.883264e-003):8.603763e-002,26:1.093712e-001):7.642276e-002):2.042573e-002):4.140165e-002,(2:1.099152e-002,((38:1.483153e-001,39:5.003323e-002):3.785554e-002,(23:1.035317e-001,(((29:3.367954e-002,(28:4.457005e-002,33:1.029535e-001):7.651104e-002):1.034362e-001,(32:1.471449e-001,34:2.698138e-001):7.263454e-002):4.795724e-003,(7:2.288911e-001,37:2.043615e-002):1.399694e-001):3.493569e-002):3.386963e-002):1.163199e-002):4.774257e-002):2.941715e-002,30:2.263913e-002):2.813428e-002):3.722728e-002,27:1.991193e-001):1.379455e-001):5.745116e-002):3.495273e-002,(9:2.805975e-002,((13:5.216725e-002,(11:1.348888e-001,12:1.001923e-001):9.931890e-002):8.264242e-003,((8:6.274314e-003,((3:5.514835e-002,15:1.538824e-002):1.506807e-001,14:2.439229e-002):5.114646e-002):9.816299e-003,10:1.576203e-001):4.650141e-002):8.128070e-002):1.067386e-001):8.199373e-002):1.267896e-001,(6:2.421799e-002,16:5.719556e-002):8.581166e-002):2.227792e-002,(19:4.131370e-002,17:1.934407e-001):2.860094e-002,1:5.133999e-002);

tree gen.135000 = [&U] (((19:6.143298e-003,((5:2.032511e-002,(40:2.304839e-001,4:7.318200e-002):9.401208e-003):2.576616e-002,((18:2.865786e-002,((41:3.877624e-001,42:1.568632e-001):2.923375e-001,(((((23:9.892746e-003,((34:4.898644e-002,(((28:9.636823e-002,29:2.547945e-002):3.267425e-002,33:1.301510e-001):6.161402e-002,32:1.754593e-001):5.253907e-002):8.709315e-002,(7:2.592124e-001,37:9.746783e-003):1.374702e-001):5.957386e-002):8.240100e-002,(2:1.757139e-001,(38:1.118396e-001,39:1.831283e-001):8.932115e-002):5.926647e-002):1.352254e-001,(27:1.296124e-001,((35:4.028903e-002,(36:6.584900e-002,(((20:3.114681e-002,(25:4.548320e-003,24:4.355060e-003):3.269714e-002):1.278374e-002,26:2.003518e-002):1.110911e-002,21:2.313143e-002):6.037240e-002):3.616810e-002):7.042171e-002,22:5.681736e-002):2.354367e-002):2.839550e-002):4.627569e-002,31:9.661999e-002):1.030661e-002,30:6.695331e-002):2.704354e-001):9.965198e-002):5.817663e-002,(9:3.345916e-002,(((((3:1.759582e-002,15:7.235471e-002):6.384876e-002,14:1.937661e-002):9.603576e-002,10:1.891005e-001):2.783258e-003,(13:6.951268e-004,(11:3.419289e-002,12:6.708962e-002):7.376787e-002):2.795357e-002):5.229105e-002,8:4.265866e-002):7.030565e-002):1.212746e-001):1.003276e-001):1.466610e-001):7.249123e-003,(6:2.887814e-002,16:2.651594e-002):1.260589e-002):6.655671e-003,17:2.322459e-001,1:6.081479e-002);

tree gen.136000 = [&U] (((((4:4.926731e-002,40:1.985137e-001):1.417719e-002,5:4.020553e-003):9.496733e-002,((18:1.325123e-001,((41:3.861194e-001,42:1.561986e-001):2.910989e-001,((27:1.292260e-001,(((35:1.044278e-001,(36:1.012107e-002,(((25:3.023423e-002,24:4.340608e-002):1.905358e-002,26:2.347761e-002):4.889921e-003,(20:3.070807e-003,21:5.290796e-003):5.954449e-002):5.851026e-002):2.999099e-002):3.171602e-002,(2:9.465858e-002,((38:1.000755e-001,39:5.430764e-002):7.898663e-002,(23:2.954823e-003,(((32:2.296947e-001,34:6.270752e-002):2.743132e-002,((28:5.894072e-002,29:3.000260e-002):2.630730e-002,33:1.121298e-001):6.779538e-002):5.841561e-002,(7:2.845633e-001,37:2.444154e-002):9.786957e-002):5.005591e-002):5.007844e-002):4.875732e-002):1.410314e-001):2.568110e-002,(31:6.167208e-002,22:5.245490e-002):5.384507e-002):2.543843e-003):3.131124e-002,30:2.085998e-002):1.278780e-001):8.376568e-002):2.370046e-002,(9:1.796353e-002,(((14:1.395332e-002,(3:1.105002e-001,15:9.881964e-002):5.037753e-003):2.324181e-001,10:1.908238e-001):5.439935e-003,((13:5.841394e-004,(11:2.177530e-002,12:5.043064e-003):1.305241e-001):9.177310e-002,8:7.571350e-002):4.369565e-002):9.581831e-002):1.207607e-001):1.365786e-001):1.653241e-001,((19:2.113100e-002,6:1.036321e-001):1.792523e-002,16:3.131471e-002):1.342233e-002):2.906581e-002,17:1.978311e-001,1:1.296453e-001);

tree gen.137000 = [&U] ((17:1.266307e-001,((19:2.307489e-002,16:5.118379e-002):7.322522e-003,6:1.441039e-001):2.615361e-002):1.234229e-002,((4:1.368230e-001,(40:1.952740e-001,5:9.409150e-004):2.825036e-004):8.523037e-002,((18:3.369625e-002,((41:4.996913e-001,42:1.806102e-001):2.999473e-001,((30:2.611354e-002,((22:2.236172e-001,(((2:8.781955e-003,(23:8.305547e-002,(((32:1.171494e-001,((28:7.537820e-002,33:6.692220e-002):1.099644e-002,29:5.182809e-002):5.091489e-002):1.095485e-001,34:9.462181e-002):3.245506e-002,(7:1.112607e-001,37:1.137884e-002):7.430240e-002):7.967060e-002):5.466160e-002):2.023889e-002,(38:1.090542e-001,39:2.515491e-002):2.613535e-002):1.167906e-001,31:8.555540e-002):2.748163e-002):9.554587e-002,27:7.457586e-002):2.138752e-002):2.948497e-002,((36:1.646458e-002,((21:7.569915e-002,(20:6.961899e-002,(25:6.216887e-002,24:2.151609e-002):6.431367e-002):1.650055e-002):5.176280e-002,26:3.898893e-002):1.279040e-001):1.066283e-001,35:3.939805e-002):4.498065e-002):8.735917e-002):1.074148e-001):7.244364e-002,(9:7.069260e-002,(10:4.109095e-002,((14:4.089687e-002,(15:7.582745e-003,3:1.111989e-001):2.408475e-001):2.906403e-001,((13:4.570653e-003,(11:1.097927e-001,12:2.690752e-002):1.712372e-001):7.129629e-002,8:1.492454e-002):2.424906e-002):2.970660e-003):1.844933e-002):2.240005e-001):1.460279e-001):1.406489e-001,1:1.634155e-001);

tree gen.138000 = [&U] ((19:2.051710e-001,(6:1.953991e-001,16:8.511511e-002):3.802024e-002):6.653493e-002,(17:6.994017e-002,(((5:4.119041e-002,40:6.067750e-001):1.145473e-001,4:1.007600e-001):2.427820e-001,((18:6.301293e-002,((41:8.528992e-001,42:1.526781e-001):3.144947e-001,((((36:2.471603e-002,(20:2.134557e-002,(26:1.407528e-001,((25:1.033825e-001,24:1.503075e-002):7.794302e-002,21:7.847307e-003):3.262417e-002):9.990115e-002):1.689709e-001):1.609188e-001,35:6.551622e-002):1.818986e-001,31:9.358669e-002):3.497197e-002,(30:5.630376e-002,((((38:1.672362e-001,39:5.787926e-002):2.650936e-002,((23:1.926768e-002,((((29:9.466496e-002,(28:1.364736e-001,33:6.844662e-002):2.259946e-002):1.275304e-001,34:3.340024e-002):5.168615e-003,32:1.948114e-001):7.170465e-002,(7:2.691302e-001,37:4.458191e-002):5.342789e-002):5.934335e-002):2.426659e-002,2:4.233083e-002):2.321454e-002):1.715396e-001,22:1.152486e-001):3.883788e-002,27:1.702752e-001):4.572636e-003):6.032323e-003):2.264846e-001):1.529387e-001):5.760916e-003,((8:6.503906e-003,(((15:1.420632e-001,(14:6.784481e-004,3:9.282204e-002):1.102687e-001):3.734342e-002,(13:1.600063e-002,(11:6.596408e-002,12:1.785229e-002):2.847557e-001):6.812040e-002):1.143837e-001,10:2.572521e-001):7.413311e-002):8.858276e-002,9:3.353698e-002):1.870183e-001):5.716551e-002):2.306416e-001):1.352030e-001,1:2.574092e-001);

tree gen.139000 = [&U] ((19:2.822951e-001,17:1.766879e-001):1.081066e-002,((((4:3.994692e-002,5:5.119086e-002):7.738656e-002,40:1.685494e-001):1.169168e-001,((18:3.912401e-001,((41:6.045261e-001,42:1.279889e-001):4.798365e-001,((((27:1.679687e-001,31:1.503928e-001):3.792251e-002,((36:9.215125e-002,((25:1.075035e-001,24:1.033916e-002):7.467705e-002,((26:6.983123e-002,21:2.181741e-002):3.761678e-002,20:1.336939e-002):8.956503e-003):7.027944e-002):1.479599e-001,35:2.479261e-001):8.638112e-002):3.190169e-002,(22:1.472099e-001,30:6.884028e-002):6.177346e-003):9.666894e-003,(2:9.904076e-002,((23:2.709060e-002,((((33:3.675709e-002,(29:6.755434e-002,28:1.892689e-001):6.697128e-002):8.759684e-002,32:2.009927e-001):3.145244e-002,34:3.519763e-002):1.950364e-001,(7:1.612045e-001,37:7.395070e-002):1.646241e-001):2.171987e-001):1.384130e-001,(38:1.622320e-001,39:7.223835e-002):3.033502e-002):6.125157e-002):1.635326e-001):2.672900e-001):1.611689e-001):5.829811e-002,(9:2.038007e-002,((((14:6.403588e-002,3:3.708789e-002):5.947739e-003,15:4.888867e-002):4.456459e-001,(13:1.394301e-001,(11:4.476188e-002,12:7.847992e-003):2.689274e-001):7.435159e-002):1.380860e-001,(10:2.354936e-001,8:9.026515e-003):6.301817e-002):8.350690e-002):1.902221e-001):2.145047e-001):1.922131e-001,(6:8.054535e-002,16:2.531762e-002):1.106582e-001):5.976791e-003,1:2.721306e-001);

tree gen.140000 = [&U] ((17:2.798487e-001,((((5:5.260228e-002,4:2.417695e-002):1.449123e-001,40:2.802138e-001):2.247891e-001,((18:9.206420e-002,((41:6.082598e-001,42:7.997532e-002):4.329808e-001,(((27:2.105358e-001,((22:1.444326e-001,((36:1.014695e-001,(((25:1.419632e-003,24:2.771766e-002):3.390356e-002,20:1.067877e-001):1.530041e-002,(21:7.979197e-003,26:1.057967e-001):4.558853e-002):8.788658e-002):5.416957e-002,35:3.125805e-001):2.829919e-002):1.377990e-002,31:1.224393e-001):3.069178e-002):7.778545e-003,(((23:1.323048e-002,(((((33:1.320999e-001,29:1.087364e-001):2.262555e-003,28:1.279678e-001):1.525863e-001,32:2.589121e-001):1.225819e-002,34:2.905552e-001):3.978547e-003,(7:1.622001e-001,37:3.619306e-002):1.487782e-001):8.378361e-002):3.876188e-002,2:9.032143e-002):1.819802e-002,(38:1.317535e-001,39:6.057577e-002):8.964065e-002):1.468907e-001):6.284554e-002,30:2.321290e-002):2.630933e-001):1.153550e-001):7.726817e-002,(9:9.758371e-003,(((((15:2.356588e-002,14:6.592189e-002):1.773956e-002,3:1.279307e-002):4.657080e-001,8:8.812980e-004):3.676012e-002,10:4.007046e-002):5.903996e-003,(13:4.210944e-002,(11:3.939005e-002,12:1.239377e-002):2.577913e-001):7.481080e-002):1.186844e-002):5.147401e-002):1.397646e-001):2.701276e-001,(6:8.070966e-002,16:1.893207e-001):7.890510e-002):2.762167e-002):2.039107e-001,19:2.214914e-001,1:1.051340e-001);

tree gen.141000 = [&U] (17:2.718308e-001,(((40:1.897307e-001,(5:2.171567e-002,4:4.474948e-002):1.645389e-002):6.551405e-002,((18:8.402442e-002,((41:5.551417e-001,42:1.527583e-001):3.279060e-001,((31:1.441285e-001,(((39:3.146602e-001,38:4.081654e-002):3.413971e-002,(2:3.867398e-002,(23:2.229902e-002,((34:8.548509e-002,(32:8.401147e-002,((29:6.244518e-002,33:1.234090e-001):3.099810e-002,28:9.324494e-002):2.540815e-001):6.078740e-002):3.322066e-002,(7:2.844166e-001,37:2.808130e-001):3.832871e-002):6.542216e-002):5.426515e-002):8.712680e-003):1.302371e-001,((27:1.493300e-001,30:1.013016e-001):2.025452e-005,((36:5.359718e-002,((21:7.012347e-003,26:6.899003e-002):9.257698e-004,(20:9.840871e-003,(25:2.626317e-002,24:6.051348e-002):9.537479e-002):8.619260e-003):1.008717e-001):1.592969e-001,35:6.756241e-002):1.390215e-001):1.185753e-002):9.562031e-002):2.362167e-002,22:3.722923e-002):2.571387e-001):1.538926e-001):6.814507e-002,(9:9.449068e-003,((10:4.313475e-002,(((15:1.866863e-002,3:9.618932e-003):4.944612e-002,14:6.016507e-002):2.617594e-002,(13:9.983426e-003,(11:2.188678e-002,12:4.072303e-003):1.959588e-001):1.490096e-001):1.086093e-002):4.986470e-002,8:2.764234e-002):1.693539e-002):7.610768e-002):1.641805e-001):3.309810e-001,(19:1.483616e-002,(6:4.847675e-002,16:1.200096e-001):4.929532e-003):1.202247e-001):2.061543e-002,1:1.914551e-001);

tree gen.142000 = [&U] ((((((4:3.709789e-002,5:3.514803e-002):7.069894e-004,40:1.036979e-001):5.606218e-002,((18:7.975043e-002,((41:4.313803e-001,42:4.267861e-002):3.297434e-001,((27:8.545737e-002,((30:1.207502e-001,22:1.188937e-001):6.226161e-002,(((23:6.304823e-003,((32:1.334241e-001,(34:6.642734e-002,((28:4.815244e-002,29:1.153591e-001):3.138436e-004,33:9.589663e-002):6.245941e-002):3.239843e-003):3.083562e-002,(7:1.303917e-001,37:1.621261e-001):3.035354e-002):1.135466e-002):4.474400e-002,(39:5.575926e-002,38:1.512944e-001):6.324299e-002):2.118368e-002,2:4.524915e-002):1.597533e-001):4.101679e-003):4.252050e-003,(((36:9.106869e-002,(20:8.148652e-003,(21:5.784425e-002,(26:4.848423e-002,(25:2.673327e-002,24:1.442952e-002):5.160218e-002):1.169460e-002):6.646528e-003):1.395084e-001):8.563303e-002,35:7.111837e-002):4.302106e-002,31:1.136660e-001):8.862086e-003):1.263662e-001):1.483944e-002):1.621798e-001,(9:5.765622e-002,(((((15:4.918304e-002,3:2.024963e-002):1.170496e-002,14:4.733099e-002):2.532283e-002,(13:1.783417e-002,(11:3.812126e-002,12:3.164438e-003):2.867051e-001):2.564372e-002):9.146051e-003,10:9.711633e-002):3.530184e-002,8:1.771870e-002):1.698489e-001):4.193190e-002):1.210158e-001):1.063059e-001,19:1.689638e-001):3.720186e-002,17:1.082850e-001):2.156146e-002,(16:3.099761e-002,6:5.956300e-002):9.647046e-002,1:2.203603e-001);

tree gen.143000 = [&U] ((19:7.799008e-002,((((4:4.337896e-002,5:1.756430e-002):1.295200e-002,40:1.492467e-001):1.053910e-001,((18:9.804277e-002,((41:4.834795e-001,42:6.155281e-002):3.589617e-001,((((36:9.219522e-002,((25:2.442310e-002,24:2.601182e-003):6.603354e-002,((21:2.015926e-003,26:1.238620e-001):2.295099e-002,20:4.322288e-002):7.600525e-002):2.621273e-002):1.742634e-001,35:1.284044e-001):6.055140e-002,((22:7.594125e-002,(31:1.723411e-001,27:1.249676e-001):2.494545e-002):4.752693e-003,((39:8.120082e-002,38:1.530521e-001):1.475556e-001,(2:5.715418e-002,(23:2.061517e-002,((34:9.417663e-002,(32:2.170968e-001,((33:1.564176e-001,29:2.713218e-002):7.042213e-002,28:6.498537e-002):4.582002e-002):3.021289e-002):1.023982e-001,(7:7.491985e-002,37:1.342085e-002):2.497639e-001):3.708635e-002):5.170518e-002):2.491762e-002):1.453337e-001):5.994280e-002):8.105099e-002,30:1.271178e-001):2.316575e-001):2.486421e-001):8.804944e-003,(9:8.014498e-002,(((10:2.918605e-002,8:3.935844e-002):1.068376e-002,(13:5.089584e-002,(11:5.498002e-002,12:5.478462e-004):1.110544e-001):1.178055e-001):5.603092e-003,((3:1.047294e-002,15:5.903891e-002):1.315428e-002,14:3.421088e-002):1.269619e-001):4.841066e-002):1.614545e-001):1.745338e-001):1.276584e-001,(16:4.057059e-002,17:3.186548e-001):2.471801e-002):1.054946e-002):1.379108e-002,6:8.970162e-002,1:4.017147e-001);

tree gen.144000 = [&U] (((5:4.381132e-002,(4:5.380464e-002,40:2.516352e-001):5.339035e-002):6.322145e-002,((18:1.573904e-001,((41:6.974508e-001,42:2.844591e-001):4.635475e-001,(30:1.707230e-001,((31:7.875808e-002,((27:2.209993e-001,22:1.247852e-001):4.394647e-002,((39:5.679737e-002,38:2.540247e-001):5.586344e-002,((23:1.360994e-002,(((((28:1.107413e-001,33:1.148205e-001):9.868207e-003,29:4.836479e-002):5.553999e-002,34:1.507699e-001):4.936210e-002,32:3.774090e-001):1.750562e-001,(7:1.852319e-001,37:2.516062e-001):3.862414e-002):1.272286e-001):1.111925e-001,2:5.481463e-002):4.108165e-002):2.894269e-001):1.746685e-002):2.680828e-003,((36:1.651894e-001,(20:7.330931e-002,((21:3.345886e-003,(25:4.054631e-002,24:2.379376e-003):3.898989e-002):3.697527e-002,26:2.068813e-001):1.088469e-001):4.680640e-002):8.289191e-002,35:2.800910e-001):2.180170e-002):5.786244e-002):5.381044e-001):1.143254e-001):8.963226e-002,(9:1.579807e-002,(((10:6.509535e-003,8:4.458647e-002):4.407729e-002,((15:3.268727e-002,14:3.650915e-002):6.413260e-002,3:8.639298e-003):3.458829e-001):7.377031e-005,(13:4.740511e-002,(11:8.259076e-002,12:1.754315e-001):2.283517e-001):2.510469e-002):7.619448e-002):2.230570e-001):9.101109e-002):1.536624e-001,((17:2.301355e-001,(6:3.797139e-002,16:4.005322e-002):1.186361e-001):1.768864e-002,19:7.178701e-002):1.857213e-002,1:9.128585e-002);

tree gen.145000 = [&U] (17:2.993903e-001,(((6:6.678251e-002,16:1.700707e-002):4.873018e-002,(((5:5.864638e-002,4:1.212044e-001):8.224789e-003,40:2.701203e-001):2.382708e-001,((18:2.289190e-001,((41:7.162385e-001,42:3.092949e-001):4.010617e-001,((31:8.380854e-002,(35:2.888019e-001,(36:8.895358e-002,(20:3.955229e-002,((24:8.166502e-002,25:8.148660e-002):1.414073e-001,(21:1.009721e-002,26:2.099461e-002):9.274571e-003):9.865171e-002):9.473850e-002):1.082322e-001):9.265081e-002):4.841841e-003,((((23:6.430370e-002,((34:1.704719e-001,(((28:1.157668e-001,29:4.624707e-002):1.936234e-002,33:1.099161e-001):5.878500e-002,32:4.117166e-001):6.400421e-002):8.647369e-002,(7:2.014043e-001,37:3.096929e-002):1.807939e-001):3.479905e-002):1.398030e-001,((39:1.954865e-001,38:1.742741e-001):1.687207e-001,2:5.960043e-002):6.969018e-002):1.426195e-001,30:2.829973e-002):1.211634e-001,(27:2.482885e-001,22:1.829941e-002):7.786559e-003):9.700335e-003):6.528953e-001):1.844118e-001):6.494709e-002,(9:1.184275e-002,(10:2.895179e-001,((14:6.342972e-002,(3:4.001622e-003,15:3.477828e-003):8.716603e-002):2.668589e-001,((13:8.331827e-002,(11:8.980166e-002,12:1.849649e-002):1.390471e-001):4.248387e-002,8:1.512696e-002):3.903792e-002):6.083220e-003):2.068695e-001):1.276290e-001):2.042644e-001):1.572703e-001):9.865174e-002,19:8.201303e-002):3.208293e-002,1:1.573302e-001);

tree gen.146000 = [&U] ((((4:1.339313e-001,40:2.625626e-001):7.177135e-002,5:6.376020e-002):8.012577e-003,((18:2.529563e-001,((41:6.645686e-001,42:1.886620e-001):7.141161e-001,(((23:9.371925e-002,((34:1.177898e-001,(((29:1.056153e-001,28:1.280118e-001):5.656319e-002,33:1.941043e-001):1.109609e-001,32:5.722598e-001):2.871337e-002):1.123786e-001,(7:2.842098e-001,37:8.375158e-003):4.628663e-002):1.614026e-002):2.289673e-002,(2:3.363696e-001,(39:3.854664e-002,38:1.535531e-001):6.323974e-002):1.773672e-001):1.476996e-001,((22:1.773549e-002,(27:1.865277e-001,(30:3.391140e-002,31:5.611224e-002):2.184113e-002):4.287847e-002):1.030885e-002,(35:2.604234e-001,(36:1.424473e-001,(((24:4.969974e-002,25:9.004298e-002):3.584740e-002,26:1.537088e-001):1.055261e-002,(21:1.665940e-001,20:3.589641e-001):1.510049e-002):7.234076e-002):2.003774e-001):1.299435e-001):2.996101e-002):5.173047e-001):9.562005e-002):7.176676e-002,(9:5.513004e-002,((8:6.369704e-002,(14:2.182774e-002,(3:8.682001e-003,15:6.117464e-002):1.044993e-001):1.870598e-001):1.142694e-002,((13:2.254624e-002,(11:7.885423e-002,12:4.474577e-002):2.048662e-001):2.761867e-001,10:3.199183e-001):4.936364e-002):1.001498e-001):9.281857e-002):1.916184e-001):3.174212e-001,(17:2.936840e-001,(19:9.207094e-002,(6:9.262309e-002,16:1.254102e-001):7.847390e-002):4.185257e-002):1.788833e-001,1:1.596765e-001);

tree gen.147000 = [&U] (((17:4.492085e-001,(((5:6.062649e-002,4:7.941027e-002):1.032127e-001,40:2.734446e-001):3.098521e-002,((18:1.137457e-001,((41:7.236435e-001,42:1.721135e-001):3.034827e-001,((35:3.076721e-001,(36:8.001679e-002,(((21:4.102591e-003,(24:9.796304e-002,25:7.520547e-002):1.125898e-001):1.683800e-001,26:1.432934e-001):8.348707e-002,20:3.600411e-002):1.938985e-001):1.633755e-001):3.878997e-002,(22:1.491162e-002,(31:1.018647e-001,((30:8.033858e-002,27:2.203694e-001):3.941442e-002,(((23:8.509496e-002,(((32:5.498110e-001,((28:1.492619e-001,33:2.678556e-001):1.663302e-001,29:6.892903e-003):8.901461e-002):6.476077e-004,34:1.155962e-001):2.006439e-001,(7:2.566772e-001,37:1.616407e-001):7.873711e-002):5.822003e-002):3.164498e-002,(39:2.354162e-001,38:6.753709e-001):7.808516e-002):8.119867e-002,2:7.973141e-002):1.114737e-001):1.406565e-001):3.994297e-002):2.266694e-002):4.388148e-001):6.943220e-002):7.465302e-002,(9:4.232716e-002,(10:1.541739e-001,((14:8.578526e-004,(3:9.108265e-003,15:2.483169e-002):1.149825e-001):1.452298e-001,(8:2.726990e-003,(13:2.296186e-002,(11:8.894809e-002,12:6.078035e-002):2.113561e-001):6.842653e-002):2.444622e-002):9.880314e-002):5.265960e-002):2.018575e-001):2.588925e-001):2.036877e-001):1.511471e-002,(6:1.173180e-001,16:3.170647e-002):4.862462e-002):5.797849e-002,19:4.264781e-002,1:2.974540e-001);

tree gen.148000 = [&U] (((((4:6.767567e-002,5:1.156828e-002):6.063337e-002,40:3.787646e-001):9.036201e-002,(((41:6.399218e-001,42:1.566107e-001):2.976195e-001,(((30:6.868531e-002,((27:3.614708e-001,31:1.258179e-001):2.924947e-002,((38:2.170999e-001,39:1.998715e-001):3.264249e-001,(2:4.074896e-002,(23:3.560609e-002,((((29:2.107659e-001,(28:1.310281e-001,33:8.038966e-002):1.049150e-001):5.573302e-002,34:2.196102e-001):1.830943e-002,32:1.867172e-001):1.798813e-001,(7:3.521483e-001,37:6.943589e-002):2.710048e-002):5.345295e-002):2.554326e-002):3.010761e-002):2.613670e-001):1.014210e-001):3.489834e-002,22:2.104529e-001):2.324116e-002,(35:1.405474e-001,(36:1.569457e-001,(((24:4.909887e-003,25:1.344513e-002):8.951965e-002,(20:3.547727e-002,26:1.919770e-001):5.544532e-004):1.647285e-003,21:2.469906e-002):2.873996e-001):2.388413e-001):1.042341e-001):2.022748e-001):5.778867e-002,((10:1.363368e-001,(((13:1.807346e-003,(11:7.865727e-002,12:6.120395e-002):1.077464e-001):2.824773e-002,(((3:1.606404e-002,14:3.178554e-002):1.440680e-002,15:1.113763e-002):1.703418e-001,9:3.993800e-002):9.596501e-002):4.286256e-002,8:1.438872e-001):8.435598e-002):3.183156e-001,18:1.005859e-001):9.850455e-002):2.332859e-001):1.045672e-001,19:1.061550e-001):5.850008e-002,(17:4.167024e-001,(6:1.213796e-001,16:3.657437e-002):1.192805e-002):5.538588e-003,1:1.679539e-001);

tree gen.149000 = [&U] (17:1.322747e-001,(((6:1.368781e-001,19:6.728661e-003):2.677594e-002,16:9.098506e-002):5.490469e-002,(((4:1.632101e-001,40:6.440931e-001):1.261416e-001,5:2.736731e-002):2.033983e-001,((9:2.431548e-003,((((13:1.382020e-001,(3:8.273504e-002,(14:5.902029e-002,15:1.655629e-002):2.206225e-002):2.118676e-001):8.575704e-002,(11:9.151657e-002,12:2.209534e-002):1.541213e-001):1.212537e-001,10:2.710642e-001):1.030126e-001,8:9.970278e-003):1.241319e-001):2.140966e-001,(((41:7.445395e-001,42:2.290008e-001):6.882475e-001,((((35:2.145333e-001,(36:1.500300e-001,(21:3.360601e-003,((24:1.273311e-001,25:4.968547e-002):2.428874e-001,(26:1.341894e-001,20:3.449070e-002):1.774749e-001):1.458012e-002):1.832391e-001):2.512567e-001):2.264696e-001,((31:1.507833e-001,((38:2.294473e-001,39:2.199180e-002):5.554410e-001,(2:2.571375e-002,(23:1.372288e-002,((32:2.236700e-001,(34:2.839042e-001,((33:2.653344e-001,28:8.783467e-002):5.494237e-002,29:6.483513e-002):1.283344e-001):5.751916e-002):6.167979e-002,(7:1.334588e-001,37:1.297215e-001):6.243819e-002):4.447496e-002):4.360095e-002):6.798423e-002):2.132946e-001):6.674406e-002,30:7.784147e-002):2.173023e-002):6.171151e-002,22:1.048601e-001):3.016813e-002,27:2.551086e-001):2.419953e-001):6.723626e-002,18:1.220581e-001):1.126103e-001):2.448564e-001):3.439069e-001):1.302995e-001,1:2.754448e-001);

tree gen.150000 = [&U] ((17:1.940045e-001,(((4:1.955261e-001,40:7.716248e-001):3.509038e-002,5:9.608478e-003):1.120454e-001,((9:2.440522e-002,((10:4.129920e-001,(13:8.549679e-003,(11:1.412844e-001,12:1.048007e-001):1.930607e-001):1.110399e-001):1.654056e-001,((14:3.093757e-002,(15:3.349229e-003,3:4.687398e-002):6.055755e-002):3.014885e-001,8:7.929744e-003):6.917433e-002):9.237922e-002):1.626070e-001,(((41:1.211881e+000,42:2.481364e-001):7.484370e-001,((31:1.746973e-001,(22:1.681784e-001,(2:3.159765e-002,((38:2.850391e-001,39:4.897578e-001):1.958587e-001,(23:1.936868e-002,((((29:1.005931e-001,28:1.023376e-001):2.402400e-002,33:4.918862e-002):2.576583e-001,(32:3.712123e-001,34:3.969753e-001):1.143320e-001):1.791781e-002,(7:2.018671e-001,37:1.976514e-001):8.202154e-002):7.381722e-002):8.155792e-003):4.382489e-003):2.841759e-001):4.557252e-002):1.147549e-002,(27:2.105377e-001,(30:5.661044e-002,(35:2.570114e-001,(36:1.736007e-001,(26:1.359135e-001,(20:1.221358e-002,((24:2.047083e-002,25:5.480814e-002):7.084597e-002,21:1.156015e-001):3.039083e-002):1.909434e-002):1.869479e-001):9.402113e-002):6.695908e-002):1.479408e-001):3.317519e-002):3.237232e-001):1.840225e-001,18:1.462258e-001):1.389784e-002):3.545838e-001):3.122868e-001):6.983002e-002,(6:9.198285e-002,(16:1.080827e-001,19:7.019853e-003):1.830963e-002):1.394288e-001,1:1.872618e-001);

tree gen.151000 = [&U] (19:1.421355e-001,(((4:4.691131e-002,(40:2.086844e-001,5:1.562914e-002):1.428208e-002):9.341600e-002,((9:2.204879e-002,(10:2.094003e-001,((((14:2.184235e-002,15:1.189639e-002):6.584000e-003,3:3.427620e-002):3.390646e-001,(13:5.381309e-003,(11:9.163789e-002,12:5.630515e-002):1.652529e-001):1.857124e-001):2.251355e-002,8:2.727221e-002):1.102196e-002):1.020947e-001):1.841863e-001,(((41:1.094869e+000,42:2.241777e-001):7.010296e-001,(30:1.000545e-001,(31:3.544466e-001,((((23:1.422458e-001,(((28:7.530854e-002,(33:1.906327e-002,29:6.549797e-002):5.083950e-002):1.682323e-001,34:2.985256e-001):9.445289e-002,32:3.067963e-001):1.642508e-001):5.341736e-003,(7:1.424070e-001,37:8.100816e-002):2.928344e-002):7.953634e-002,(2:3.252538e-002,(38:2.585706e-001,39:2.481969e-001):1.726333e-001):1.330612e-001):1.524659e-001,((22:1.220378e-001,27:1.952772e-001):1.209999e-002,(35:2.489804e-001,(36:1.707581e-001,((21:1.544010e-003,((24:1.144815e-002,25:8.112062e-003):1.520977e-001,26:1.937279e-001):1.671796e-002):1.681934e-001,20:9.357027e-003):2.822649e-001):1.577991e-001):1.180493e-001):3.581858e-002):3.535940e-003):5.207294e-002):3.053641e-001):2.501785e-001,18:5.020608e-002):1.326477e-002):1.482367e-001):9.532573e-002,((16:2.524956e-002,6:5.451669e-002):1.926066e-001,17:1.752725e-001):4.604222e-002):1.650021e-002,1:2.208000e-001);

tree gen.152000 = [&U] ((((40:2.508389e-001,(4:5.713055e-002,5:4.473917e-002):4.225353e-002):1.705724e-001,((9:8.874247e-002,(8:2.689951e-003,(((14:3.287045e-002,(15:7.590461e-003,3:2.393156e-002):3.834652e-002):4.130597e-001,10:3.233862e-001):3.076456e-001,(13:5.806866e-002,(11:7.243365e-002,12:7.019816e-002):3.021771e-001):2.748700e-002):2.799210e-001):1.905247e-001):3.492590e-001,(((41:1.830832e+000,42:2.794924e-001):7.318118e-001,((31:2.426869e-001,22:1.298889e-001):8.140153e-002,(((30:5.345761e-002,(35:2.218998e-001,(36:1.525594e-001,((((24:3.535012e-002,25:5.626697e-002):2.602004e-001,21:4.151575e-002):2.824517e-002,20:1.661518e-001):1.138236e-001,26:3.109892e-001):3.545655e-001):1.967352e-001):2.059981e-001):1.409989e-002,27:3.264838e-001):1.959806e-001,((38:7.285821e-002,39:3.443585e-002):1.514943e-001,(2:3.187393e-001,(((34:2.050429e-001,(32:6.741580e-001,(29:1.702741e-001,(28:9.664378e-002,33:1.738213e-001):9.697053e-003):8.009044e-002):5.250030e-002):1.152262e-001,(7:1.775452e-001,37:2.017944e-001):3.094003e-001):2.192003e-001,23:1.906301e-001):1.838169e-001):8.492922e-002):2.587780e-001):4.037596e-003):3.863407e-001):2.357426e-001,18:9.972216e-002):1.129088e-001):1.866031e-001):5.775412e-001,17:2.212508e-001):3.782195e-002,(16:2.036504e-002,(19:1.950369e-002,6:6.843754e-002):1.763396e-002):1.050333e-001,1:3.393447e-001);

tree gen.153000 = [&U] (6:2.590348e-002,(((((5:5.895116e-003,4:4.276084e-002):7.210223e-003,40:3.352576e-001):1.141975e-001,((9:1.110804e-001,(((8:9.214363e-002,10:2.524458e-001):4.612703e-003,(13:2.150650e-002,(11:5.057787e-002,12:4.640163e-002):3.098778e-001):4.735245e-002):5.316076e-003,(3:7.216699e-002,(14:2.136873e-002,15:9.369156e-002):1.060489e-002):2.434948e-001):7.617822e-002):6.779705e-002,(((41:8.366302e-001,42:2.550107e-001):2.604001e-001,(((31:4.514901e-001,((((38:3.825430e-001,39:2.071671e-001):9.119702e-002,((((34:2.133167e-001,32:4.971734e-001):7.038072e-002,(28:1.909245e-001,(33:5.344975e-002,29:9.351237e-002):1.584233e-001):1.450899e-001):9.247111e-002,(7:4.004207e-001,37:1.591986e-001):3.913673e-002):8.979961e-002,23:5.364633e-002):1.081710e-001):8.569208e-002,2:9.654565e-002):2.557439e-001,(27:2.556957e-001,(35:2.105748e-001,(36:1.203563e-001,(20:4.443463e-002,(26:3.040137e-001,(21:4.902230e-002,(24:2.797303e-002,25:2.598828e-002):2.016337e-001):3.675293e-002):5.234123e-002):3.553980e-001):1.153501e-001):5.572272e-002):8.342841e-002):4.227152e-002):3.911796e-002,30:1.737023e-001):5.672818e-003,22:1.029077e-001):3.088102e-001):1.436823e-001,18:1.610467e-001):7.339055e-002):4.938969e-001):2.455089e-001,(16:1.244752e-001,19:1.236868e-001):4.660242e-003):1.763225e-002,17:1.711869e-001):1.669206e-002,1:4.219422e-001);

tree gen.154000 = [&U] (((((5:4.808687e-002,40:3.531666e-001):2.033434e-002,4:4.917348e-002):6.893300e-002,((9:6.835241e-003,((8:1.391290e-001,(14:1.673844e-002,(15:8.886421e-002,3:1.281519e-002):1.040940e-001):3.644528e-001):9.684420e-002,(10:4.159898e-002,(13:2.383504e-002,(11:2.234670e-001,12:4.179367e-002):3.104069e-001):1.030063e-001):4.433762e-002):1.427528e-001):3.558541e-001,(((41:9.272136e-001,42:3.829725e-001):4.583114e-001,((((30:7.269212e-003,(22:7.820397e-002,27:2.999499e-001):2.351924e-002):9.618718e-002,((38:4.172966e-001,(39:1.462886e-001,2:1.101758e-002):1.908557e-001):6.820638e-003,(((((33:5.894340e-002,(28:9.808554e-002,29:4.764305e-002):4.622684e-002):7.058900e-002,34:2.242854e-001):2.106930e-003,32:1.590399e-001):5.794426e-002,(7:1.304220e-001,37:3.706060e-002):2.386168e-001):1.333728e-001,23:1.055500e-001):6.516584e-002):1.311160e-001):4.557249e-003,31:2.572146e-001):1.837707e-002,(35:2.333741e-001,(36:1.333875e-001,((21:5.567741e-002,26:2.833048e-002):3.761956e-002,(20:3.572855e-002,(24:4.555242e-002,25:1.815142e-002):4.435844e-002):1.798016e-001):1.044650e-001):4.346728e-002):1.260331e-001):3.310610e-001):2.387716e-001,18:2.460646e-001):5.362379e-002):2.750002e-001):3.007113e-001,17:1.895783e-001):4.780642e-002,((19:1.447173e-001,16:1.304104e-001):8.024025e-002,6:2.870809e-002):1.145963e-001,1:4.676266e-001);

tree gen.155000 = [&U] ((6:3.108895e-001,(16:2.442680e-001,19:8.481510e-002):1.416559e-002):2.870632e-001,(17:1.872143e-001,(((5:1.795915e-002,4:8.224248e-002):6.212995e-002,40:3.750469e-001):7.266637e-002,((9:3.680636e-002,((10:1.226378e-001,((13:3.725112e-002,(11:1.087920e-001,12:1.599396e-002):3.108035e-001):3.369322e-002,((14:4.119549e-002,3:5.605696e-003):3.999053e-002,15:2.418151e-002):1.269749e-001):2.567731e-001):1.183463e-002,8:1.336919e-001):1.389419e-001):1.874220e-001,(((41:8.222383e-001,42:1.145306e-001):4.295887e-001,((31:3.056674e-001,(30:3.702581e-002,(22:4.707937e-002,((23:4.310549e-002,((7:3.335644e-001,37:1.267185e-001):2.189425e-001,(34:2.336791e-001,((28:1.710582e-001,(29:1.131007e-001,33:5.052363e-002):2.806461e-002):1.181669e-001,32:1.363895e-001):1.145149e-002):1.041872e-001):2.000258e-002):1.641961e-001,(2:1.268993e-001,(38:2.374901e-001,39:2.828501e-001):5.505327e-002):1.055155e-001):1.164807e-001):6.013427e-002):5.858730e-002):2.777772e-002,(27:2.468282e-001,(35:1.980760e-001,(36:1.721678e-001,(21:1.775072e-002,((20:6.381233e-002,26:1.084256e-001):6.181859e-003,(24:1.666909e-006,25:4.519217e-002):1.172800e-001):1.042272e-001):4.021526e-002):3.515280e-002):9.403600e-002):1.637936e-001):2.123429e-001):7.906184e-002,18:2.156241e-001):6.867673e-002):3.609658e-001):5.451593e-001):1.195751e-002,1:2.077881e-001);

tree gen.156000 = [&U] (((4:9.307534e-002,(5:8.063191e-002,40:4.133516e-001):5.797194e-003):3.271238e-001,(((41:9.305423e-001,42:3.465043e-001):5.933566e-001,((2:2.782919e-001,((38:2.687719e-001,39:5.572950e-002):1.879995e-001,(23:2.387187e-002,(((29:1.279981e-001,(33:1.177719e-001,28:1.935897e-001):7.429097e-004):2.232074e-001,(34:2.743263e-001,32:1.958626e-001):1.202320e-002):1.838648e-002,(7:1.751593e-001,37:9.045266e-003):6.137870e-002):1.926919e-001):4.511354e-002):1.777321e-002):2.045509e-001,((35:2.241663e-001,(36:1.123295e-001,(((24:2.417058e-004,25:5.114482e-002):5.891812e-002,(26:2.316791e-002,21:1.124382e-001):5.225961e-002):6.966982e-002,20:5.542192e-002):6.167216e-002):3.943469e-002):1.047926e-001,(30:1.079818e-001,(22:1.306705e-002,(31:3.407551e-001,27:1.921581e-001):1.163063e-001):2.722001e-002):1.570632e-002):5.799110e-002):2.546924e-001):1.548228e-001,(((9:3.496404e-002,((10:7.640630e-002,(13:1.210926e-001,(11:6.458127e-002,12:8.179425e-002):2.520973e-001):4.072468e-002):1.743377e-002,(14:9.339433e-002,(15:3.727756e-002,3:6.417877e-002):4.681579e-002):6.277939e-002):2.567798e-001):3.707328e-002,8:2.824736e-002):2.069934e-001,18:2.457814e-001):1.472084e-001):1.870285e-001):1.642350e-001,(((6:5.115678e-002,16:5.590490e-002):1.537305e-001,19:2.608675e-002):5.778981e-003,17:2.142626e-001):4.520549e-002,1:2.327347e-001);

tree gen.157000 = [&U] ((((19:2.446457e-001,16:6.344237e-002):4.064666e-002,6:8.871188e-002):5.206413e-002,17:2.601964e-001):4.176695e-003,((5:3.617069e-002,(4:1.083226e-001,40:4.378990e-001):1.291309e-001):4.280668e-001,((18:2.345718e-001,((41:1.072941e+000,42:2.924024e-001):6.996883e-001,((35:3.141299e-001,(36:2.784017e-001,(21:1.333087e-001,(26:4.122309e-001,((24:2.294495e-002,25:9.705407e-002):1.916549e-002,20:6.716791e-002):4.251845e-003):3.310728e-002):1.140037e-001):3.973384e-002):1.122522e-001,(((22:1.548337e-002,27:2.237570e-001):8.100393e-003,(31:2.537092e-001,((2:2.547251e-001,(38:1.601953e-001,39:4.942568e-002):1.318045e-002):6.727289e-002,(23:1.388085e-001,((32:2.973870e-001,(34:1.238291e-001,(29:1.178509e-001,(33:1.457090e-001,28:2.168079e-001):4.014936e-002):9.420086e-002):5.585077e-003):9.302255e-002,(7:2.019635e-001,37:4.967940e-003):6.672131e-002):1.432397e-001):9.610242e-002):3.680732e-001):5.411332e-002):1.888242e-002,30:2.251915e-001):1.254936e-001):3.685511e-001):4.936078e-001):1.305655e-001,((9:1.126997e-001,(13:6.049216e-002,(11:6.353768e-002,12:2.345190e-001):2.852395e-001):8.884250e-002):2.087501e-002,((((15:3.291913e-003,3:1.043714e-001):1.574826e-002,14:1.149534e-002):7.571925e-002,10:1.726606e-001):2.995471e-001,8:2.724642e-002):5.214558e-002):4.163791e-001):1.653632e-001):4.004915e-001,1:3.692298e-001);

tree gen.158000 = [&U] (((((5:2.376233e-002,(40:8.266059e-001,4:3.751911e-001):3.389602e-002):2.791097e-001,((18:3.301690e-001,((41:1.437188e+000,42:2.540079e-001):8.032443e-001,((((31:1.865256e-001,((35:4.207722e-001,(36:3.342912e-001,((20:2.016168e-002,21:3.443181e-003):1.135231e-001,(26:2.278873e-001,(24:6.113096e-002,25:4.468300e-002):1.366160e-001):1.244158e-001):1.085349e-001):2.106623e-001):1.644309e-001,30:2.949730e-001):3.362841e-002):2.367985e-003,22:3.838660e-001):6.036176e-003,27:1.705590e-001):1.610511e-002,((38:2.056152e-001,39:1.077948e-001):4.620635e-002,(2:5.829944e-001,(23:5.626131e-003,((((33:1.969623e-001,29:2.072145e-001):1.037612e-003,28:2.187685e-001):1.101050e-001,(32:4.011729e-001,34:4.027857e-001):8.538666e-002):2.031462e-001,(7:2.705270e-001,37:7.227648e-002):6.163738e-002):1.296512e-001):6.321286e-002):1.461920e-002):4.988211e-001):4.212106e-001):5.128825e-002):1.160304e-001,((((((15:8.806076e-002,3:3.310868e-001):5.612960e-001,14:3.408020e-001):3.860086e-002,10:2.366409e-001):1.009220e-002,(13:1.906987e-002,(11:9.147217e-002,12:5.981875e-002):4.714944e-001):3.501422e-001):3.216812e-002,9:1.521993e-001):8.078675e-003,8:1.001579e-001):3.440610e-001):6.074727e-001):5.242336e-001,(6:1.488941e-001,16:3.240815e-002):7.431082e-002):4.298593e-001,17:3.719730e-001):1.378871e-001,19:2.022359e-001,1:3.807099e-001);

tree gen.159000 = [&U] ((17:3.422009e-001,(19:1.192442e-001,(6:3.093416e-001,16:1.286012e-001):1.032177e-001):1.228066e-002):4.602589e-002,(((4:2.445341e-001,5:2.235583e-002):6.209534e-002,40:7.365533e-001):2.507799e-001,((18:3.037428e-001,((41:1.322158e+000,42:4.957951e-001):8.942949e-001,((((30:9.974378e-002,31:1.702036e-001):2.096446e-002,(35:3.870943e-001,(36:2.599393e-001,((26:2.096476e-001,((24:8.696285e-003,25:1.166297e-001):1.427776e-001,20:1.854797e-002):2.729499e-001):6.262767e-002,21:8.081794e-002):1.054432e-001):1.548242e-001):8.344593e-002):4.959848e-002,((((38:1.770820e-001,39:2.991683e-001):1.313759e-001,2:2.687519e-002):3.647156e-001,(23:1.834542e-001,((((33:1.857082e-001,(28:5.861436e-002,29:1.841172e-001):8.570917e-002):1.158758e-001,34:3.465293e-001):4.527804e-002,32:4.042434e-001):2.134207e-001,(7:2.407764e-001,37:8.632415e-002):7.183579e-002):7.414164e-002):7.025350e-002):1.412995e-001,22:2.521549e-001):1.331025e-001):1.096503e-001,27:1.597654e-001):5.497174e-001):1.938005e-001):1.082587e-001,((((13:6.388251e-002,(11:6.900109e-002,12:7.081107e-002):7.338724e-001):6.770775e-003,8:2.861601e-002):6.860687e-002,(((3:3.033452e-001,14:2.006457e-002):1.260788e-002,15:8.353912e-002):3.805577e-001,9:1.223511e-001):3.376032e-001):1.408914e-002,10:7.257238e-002):3.181099e-001):6.136611e-001):4.466354e-001,1:3.710831e-001);

tree gen.160000 = [&U] (19:1.050037e-001,((((5:1.474822e-001,(4:7.980415e-002,40:6.844825e-001):1.817296e-001):2.402575e-001,(((8:2.885535e-002,(9:1.128186e-002,((13:6.182571e-003,(11:1.798859e-002,12:3.392977e-002):2.388778e-001):6.387436e-002,((15:8.246733e-002,3:2.133419e-001):1.035502e-001,14:2.448261e-002):5.065376e-001):5.772397e-002):3.965735e-003):7.110428e-002,10:1.090991e-001):3.097318e-001,(((41:1.272506e+000,42:1.861179e-001):1.060224e+000,((27:1.842366e-001,((35:3.725575e-001,(36:2.501776e-001,((26:2.589476e-001,20:1.456263e-001):1.323111e-002,((24:2.605468e-002,25:1.333710e-002):1.202753e-001,21:2.930951e-002):5.627212e-002):3.109251e-001):1.356889e-001):2.981748e-002,(((((23:2.281938e-002,((((29:1.829456e-001,28:7.849842e-002):2.243022e-002,33:1.818202e-001):1.634734e-001,(32:2.080098e-001,34:3.380732e-001):2.152111e-001):1.997615e-001,(7:5.521189e-001,37:2.544868e-002):1.074140e-001):1.920750e-002):5.294539e-002,(38:4.314147e-001,39:1.288925e-001):6.944713e-002):1.226687e-002,2:3.614713e-002):4.319494e-001,30:8.770230e-002):6.732685e-003,22:1.951109e-001):7.220241e-002):1.922803e-001):1.721155e-001,31:1.009595e-001):7.585751e-001):3.187595e-001,18:4.551443e-002):8.337767e-002):4.537364e-001):5.994061e-001,6:1.455266e-001):8.346319e-002,(17:3.571067e-001,16:2.915181e-001):6.256484e-002):1.278520e-002,1:2.579003e-001);

tree gen.161000 = [&U] (17:3.839759e-001,((((40:8.652730e-001,(5:9.336315e-002,4:5.057308e-002):6.629548e-002):1.897652e-001,((9:1.502395e-002,(((((14:8.573538e-003,15:4.298803e-002):1.160571e-001,3:1.415071e-001):2.092350e-001,10:1.832420e-001):2.988775e-001,(13:1.582173e-002,(11:8.368336e-002,12:5.313410e-003):3.843419e-001):1.179197e-001):4.741722e-002,8:3.505564e-002):7.804133e-002):2.567924e-001,(((41:1.207074e+000,42:7.937277e-002):4.993769e-001,((35:3.750299e-001,(36:4.154437e-001,(20:6.866943e-002,((26:5.271881e-002,(24:1.310450e-002,25:3.669498e-003):1.354397e-001):1.610384e-002,21:1.218769e-001):6.061882e-002):2.976011e-001):1.064419e-001):1.619115e-001,((2:1.214081e-001,((23:7.609852e-002,((32:2.270374e-001,(((28:8.734567e-002,29:1.911471e-001):2.538623e-002,33:6.475832e-002):8.032992e-002,34:2.736526e-001):1.935078e-001):1.638638e-001,(7:6.923191e-001,37:1.058795e-001):2.272265e-001):6.228697e-002):1.449377e-001,(38:4.507552e-001,39:6.715384e-002):1.421225e-001):9.945745e-003):2.688407e-001,((31:2.371088e-001,(30:1.100888e-001,27:4.534729e-001):1.629174e-002):1.176014e-001,22:2.211321e-001):1.815277e-002):1.004006e-001):4.539163e-001):7.824798e-002,18:1.417024e-001):2.923251e-001):2.431414e-001):6.632901e-001,19:1.025423e-001):5.684756e-002,(6:3.053486e-001,16:1.112474e-001):1.402293e-001):1.418864e-001,1:2.618676e-001);

tree gen.162000 = [&U] ((((6:2.520590e-001,16:4.746016e-002):2.285403e-001,(((4:3.645516e-002,5:3.649375e-003):3.116603e-002,40:6.513795e-001):1.545615e-001,(((8:8.418281e-002,(((13:6.760183e-003,(11:1.164998e-001,12:4.976099e-002):1.636501e-001):2.059215e-002,9:1.511089e-002):6.933393e-003,((3:1.536927e-001,15:4.611002e-002):4.189651e-002,14:1.145542e-002):1.069219e-001):1.178042e-001):1.868614e-001,10:1.414159e-001):1.918583e-001,(18:2.441697e-001,((41:9.649606e-001,42:6.125544e-002):4.677728e-001,(31:9.766034e-002,((((2:9.642836e-002,((38:1.902528e-001,39:6.414042e-002):5.677857e-002,(23:5.475811e-002,(((32:3.450986e-001,(28:8.176435e-002,(33:2.665430e-001,29:1.435042e-001):5.185255e-003):2.913461e-001):3.379263e-003,34:2.168963e-001):2.602739e-002,(7:5.342929e-001,37:8.171187e-002):7.075677e-002):1.603483e-001):4.995812e-002):2.137826e-002):2.175485e-001,30:1.304383e-001):7.964535e-002,27:3.416317e-001):4.389078e-003,(22:2.019114e-001,(35:9.129643e-002,(36:2.946073e-001,(26:2.409644e-001,(20:1.666469e-003,(21:1.143251e-001,(24:9.953222e-003,25:3.751808e-002):9.997832e-002):5.237159e-002):4.770775e-002):2.095127e-001):1.045720e-001):4.496811e-002):3.840618e-002):1.317756e-002):5.128247e-001):2.802967e-001):1.017308e-002):4.441741e-001):1.234177e-001):1.477910e-001,19:8.969671e-002):1.089697e-001,17:1.544349e-001,1:2.723007e-001);

tree gen.163000 = [&U] (((16:7.138809e-002,(19:2.247692e-001,6:6.959719e-002):9.553296e-004):9.463695e-002,17:1.917604e-001):2.676468e-002,((5:5.154350e-002,(40:4.130464e-001,4:3.784206e-002):4.928600e-002):1.041514e-001,(((((((13:8.640692e-003,(11:1.895841e-001,12:1.240109e-002):1.630688e-001):1.706794e-001,3:6.419031e-003):5.109776e-002,(15:9.123699e-003,14:8.920449e-002):9.918645e-002):3.618891e-001,8:1.008123e-001):5.851470e-002,10:9.271326e-002):5.279424e-002,9:7.511801e-002):2.511831e-001,(((41:1.005984e+000,42:3.390250e-001):3.316947e-001,(((36:1.217238e-001,((21:1.484892e-002,((24:1.430735e-002,25:6.295357e-002):8.664486e-002,26:3.412481e-002):4.291226e-003):1.959826e-002,20:5.683188e-002):1.627999e-001):1.121992e-001,(35:2.184819e-001,27:3.529587e-001):9.155023e-002):1.148827e-001,((((23:4.745868e-002,(((((28:8.228701e-002,29:7.453597e-002):7.362258e-002,33:7.860357e-002):3.262963e-001,32:2.734288e-001):4.293110e-002,34:2.158065e-001):1.118996e-001,(7:5.570074e-001,37:1.314062e-001):1.570557e-001):2.873874e-001):1.543381e-001,((38:1.947105e-001,39:6.256637e-002):7.507275e-002,2:8.350725e-002):9.254255e-002):1.583917e-001,22:3.820458e-002):1.368866e-002,(31:3.163070e-001,30:3.756040e-002):8.671131e-003):1.991005e-003):9.933763e-001):1.347009e-001,18:1.323760e-001):1.533364e-002):3.337496e-001):7.161736e-002,1:2.735966e-001);

tree gen.164000 = [&U] (((5:1.131597e-002,(4:2.416608e-001,40:4.515360e-001):3.912615e-002):8.160510e-002,((18:1.160548e-001,(((((10:7.382013e-002,(15:3.051942e-002,14:4.867132e-002):1.454764e-001):1.191397e-002,3:9.019893e-002):1.476465e-001,(13:4.960280e-002,(11:1.156125e-001,12:2.127845e-002):2.405177e-001):3.605757e-001):3.358105e-001,8:4.577229e-002):7.256372e-002,9:5.493420e-002):3.172115e-001):4.396698e-002,((41:1.100560e+000,42:5.244509e-001):5.503134e-001,(30:1.942890e-002,((((36:1.757443e-001,((21:1.348093e-001,(20:1.293257e-002,(24:9.653051e-002,25:3.383896e-002):9.957188e-002):1.893020e-002):6.347944e-002,26:3.737989e-002):1.810941e-001):5.749864e-002,35:2.024940e-001):1.787944e-001,(((((23:5.192042e-002,(((((28:1.245536e-001,33:8.062737e-002):3.703736e-003,29:1.575085e-001):1.713041e-001,34:2.275648e-001):1.018783e-001,32:8.392319e-001):8.426442e-002,(7:5.046099e-001,37:3.366526e-001):8.577642e-002):2.082950e-002):1.416747e-001,(38:2.130159e-001,39:5.662140e-003):7.429140e-002):6.494100e-002,2:9.310639e-002):2.102969e-001,27:2.910845e-001):3.194264e-002,31:3.602573e-001):1.004922e-001):5.101240e-002,22:2.481222e-001):9.130763e-003):3.085214e-001):1.381452e-001):2.845645e-001):2.667859e-001,(17:2.186474e-001,((19:1.816935e-001,6:2.103941e-001):1.077801e-001,16:5.432618e-002):2.773183e-001):6.200539e-002,1:1.997043e-001);

tree gen.165000 = [&U] ((((4:1.105408e-001,40:5.010892e-001):1.328580e-002,5:4.828990e-002):3.271174e-001,((18:4.072284e-001,((41:1.111964e+000,42:1.118431e-001):5.560157e-001,(((36:1.711712e-001,((24:8.881620e-002,25:3.523473e-002):6.991512e-002,((21:5.138491e-002,20:3.399653e-002):6.145034e-004,26:1.757066e-001):5.422049e-002):2.598631e-001):1.264488e-001,35:1.693537e-001):1.354943e-001,(30:1.265106e-001,(22:7.456435e-002,(((((23:5.245841e-002,((32:2.490935e-001,(34:1.369021e-001,((29:1.004514e-001,33:9.100358e-002):2.114178e-001,28:9.485299e-002):6.197952e-002):6.344086e-002):7.693063e-002,(7:3.217276e-001,37:7.424783e-002):7.176743e-002):1.173600e-001):1.204251e-001,2:1.463925e-001):2.042462e-002,(38:2.050599e-001,39:2.429045e-001):3.430153e-002):4.717631e-001,31:1.603145e-001):1.569156e-002,27:2.821547e-001):6.665657e-003):2.646148e-002):1.914202e-002):2.432724e-001):1.862894e-001):3.332286e-002,(((((11:1.166841e-001,12:2.707802e-002):6.108855e-001,13:6.919409e-003):5.516976e-002,8:1.017365e-001):4.552739e-002,(10:3.020773e-002,((14:5.592901e-002,15:9.436644e-003):1.612870e-001,3:9.113356e-002):4.258480e-001):2.159756e-002):1.231061e-002,9:2.024015e-001):1.891346e-001):4.165579e-001):2.608794e-001,(((16:1.362464e-001,6:9.309544e-002):4.039360e-002,19:1.280456e-001):1.817167e-001,17:1.502336e-001):1.273249e-001,1:2.017736e-001);

tree gen.166000 = [&U] ((17:1.029027e-001,19:1.604661e-001):1.584019e-001,(((40:8.153211e-001,(4:3.274855e-002,5:1.264449e-001):3.844588e-002):1.041089e-002,((18:6.507345e-002,((41:7.478020e-001,42:9.398268e-002):4.339817e-001,((30:1.563209e-001,(((31:1.885253e-001,((36:4.346677e-002,((24:3.320738e-002,25:8.794671e-003):2.791616e-002,((26:5.503970e-002,21:7.055283e-004):5.108508e-002,20:1.620400e-002):4.433845e-002):4.193410e-001):1.425206e-001,35:2.086250e-001):1.146070e-001):5.957182e-002,27:3.475834e-001):2.777654e-004,((23:2.178612e-001,((32:3.183960e-001,((33:1.344894e-001,(28:2.902937e-001,29:1.015614e-001):4.438841e-002):2.568564e-001,34:1.387661e-001):8.385258e-002):6.918340e-002,(7:3.963328e-001,37:2.269922e-001):1.015811e-001):6.175046e-002):7.373407e-002,((38:9.879270e-002,39:1.611586e-001):2.862607e-001,2:1.471201e-001):1.361251e-002):3.782780e-001):9.515888e-003):1.367058e-002,22:8.812270e-002):2.861172e-001):8.557561e-002):2.241849e-001,(((8:5.533792e-002,10:1.576837e-001):5.657981e-002,(((3:1.122665e-001,15:9.504046e-003):2.408686e-003,14:2.180307e-002):3.661231e-001,((11:5.675270e-002,12:5.881922e-002):2.638936e-001,13:5.452467e-002):3.539418e-002):2.554784e-002):3.827278e-002,9:1.120601e-002):2.616583e-001):1.389270e-001):2.698993e-001,(6:4.648333e-001,16:1.665550e-001):1.281872e-001):3.081082e-004,1:2.754954e-001);

tree gen.167000 = [&U] (17:1.360346e-001,(((6:2.402793e-001,16:2.258316e-002):1.068169e-001,19:9.178926e-002):2.170124e-001,(((40:4.145694e-001,5:7.517476e-002):5.166503e-003,4:1.706810e-002):2.132237e-001,((18:1.968105e-001,((41:7.800432e-001,42:9.803471e-002):4.526927e-001,(((30:5.143944e-002,(31:1.813216e-001,((36:6.289136e-002,(21:5.004345e-002,((20:1.819233e-002,(25:1.333156e-002,24:3.973173e-002):2.529803e-002):2.021431e-001,26:1.511335e-001):4.264274e-002):4.220483e-002):8.609709e-002,35:2.439184e-001):5.577652e-002):9.555924e-002):1.023607e-001,22:1.592749e-001):4.936745e-002,(27:2.516004e-001,((38:4.713873e-001,39:1.266478e-001):3.156796e-002,((23:2.382353e-002,(((29:7.780149e-002,(28:2.600257e-001,33:1.295068e-001):9.738583e-003):8.487555e-002,(34:1.488837e-001,32:2.764084e-001):2.869442e-002):2.290793e-001,(7:2.465320e-001,37:1.559860e-001):1.030624e-001):6.985545e-002):1.229057e-001,2:1.753442e-001):1.934101e-002):4.401134e-001):4.963600e-002):2.550337e-001):1.500061e-001):2.308465e-001,(((((14:6.461850e-004,(15:5.638996e-002,3:1.843737e-002):5.699220e-003):3.912357e-001,10:2.095353e-001):6.923042e-002,8:1.259696e-002):7.379039e-002,((11:3.474450e-001,12:6.135518e-002):3.760292e-001,13:1.678250e-002):8.097667e-002):1.303529e-001,9:1.033011e-001):2.296497e-001):1.182380e-001):2.331998e-001):1.305471e-001,1:3.364365e-001);

tree gen.168000 = [&U] ((((40:4.438035e-001,4:3.172010e-002):2.576791e-002,5:4.254264e-002):1.387574e-001,((18:2.355783e-001,((41:8.806134e-001,42:2.419819e-001):4.290322e-001,((30:8.603798e-002,27:2.734264e-001):2.815181e-002,(((2:1.380089e-001,(38:5.321627e-001,39:2.602885e-001):8.375043e-003):1.503470e-002,(23:8.440577e-002,((32:3.163094e-001,(34:3.064905e-001,((33:1.438371e-001,29:5.254242e-002):6.449044e-002,28:7.772178e-002):3.120918e-001):6.526636e-002):1.056136e-001,(7:1.155309e-001,37:2.004791e-002):1.939120e-001):1.119555e-001):4.457091e-001):2.550331e-001,((22:1.765676e-001,31:1.940591e-001):8.807059e-002,((36:9.511692e-002,((24:1.780229e-001,25:1.257518e-002):9.571496e-003,((21:1.658882e-001,20:6.711983e-002):7.620167e-002,26:1.672330e-001):1.135505e-002):1.609193e-001):8.864215e-002,35:2.753665e-001):6.316179e-002):1.707675e-002):3.685663e-002):3.257176e-001):9.896984e-002):2.723425e-002,((((((11:1.099635e-002,12:6.554462e-002):4.300413e-001,13:6.598261e-002):6.780193e-002,(15:2.497902e-002,(14:6.176097e-002,3:1.771353e-001):6.001832e-002):1.466385e-001):2.980595e-001,8:2.898164e-002):1.694246e-002,10:2.008500e-001):6.012682e-002,9:1.829514e-003):4.470869e-001):1.390400e-001):4.386674e-001,((19:1.109004e-001,17:1.604347e-001):4.199922e-002,(6:6.349490e-002,16:6.827558e-002):3.941258e-002):7.737651e-002,1:2.114671e-001);

tree gen.169000 = [&U] (17:9.418245e-002,(((16:8.906192e-002,6:9.453869e-002):7.693808e-002,19:2.073574e-001):5.436215e-002,((40:3.344738e-001,(5:1.793726e-002,4:2.397640e-002):3.214618e-002):1.122871e-001,(((9:3.606421e-002,((10:7.057525e-002,((11:8.430110e-002,12:9.141154e-003):3.085029e-001,13:1.731316e-002):2.390435e-002):1.917506e-002,((15:1.149591e-002,14:4.212421e-002):4.384069e-003,3:1.097127e-002):1.273766e-001):4.852265e-002):1.070515e-001,8:7.418308e-002):3.226007e-001,(((41:6.317343e-001,42:1.263953e-001):4.744071e-001,((30:6.170834e-002,((36:6.471215e-002,(26:1.171662e-001,(21:3.845744e-002,((24:1.086656e-001,25:1.103663e-002):1.619737e-001,20:6.624415e-002):3.329782e-002):9.754885e-002):5.174465e-002):6.641968e-002,35:1.927527e-001):8.559857e-002):4.430846e-002,(31:1.932868e-001,((27:1.948027e-001,((2:1.985231e-002,((((34:2.289064e-001,32:2.269140e-001):1.047921e-001,(28:7.612906e-002,(29:7.866285e-002,33:1.674844e-001):2.349644e-003):8.650923e-002):5.827831e-002,(7:1.456941e-001,37:5.234341e-002):7.164371e-002):4.657718e-002,(38:3.885050e-001,39:1.771900e-001):4.495138e-002):1.364541e-002):1.102162e-002,23:6.200659e-002):2.401073e-001):6.327520e-003,22:1.326398e-001):2.694228e-002):6.921192e-003):2.800795e-001):1.262645e-001,18:1.160649e-001):4.947287e-002):2.373918e-001):7.742071e-002):5.943237e-002,1:2.984704e-001);

tree gen.170000 = [&U] (17:2.355686e-001,((6:8.669855e-002,16:2.641506e-002):5.473489e-002,(19:1.554489e-001,((4:1.383074e-001,(5:2.082532e-002,40:1.836397e-001):7.903323e-003):1.856782e-001,(18:1.467602e-001,(((41:5.512006e-001,42:1.102824e-001):2.194108e-001,((22:9.021475e-003,30:2.975927e-002):5.964784e-002,((31:2.104804e-001,(((36:3.377496e-002,(26:4.099971e-002,((20:5.886940e-002,(24:9.481286e-002,25:3.508968e-002):7.460935e-002):3.021120e-002,21:2.942322e-002):1.251996e-001):1.877367e-001):1.556722e-002,35:2.151899e-001):1.003232e-001,(((23:9.531516e-002,(((((28:1.081085e-001,33:1.413492e-001):1.048145e-001,29:1.144594e-002):5.022453e-002,32:2.756884e-001):2.701579e-002,34:2.004214e-001):5.566331e-003,(7:3.884172e-001,37:8.475456e-002):1.449352e-001):7.153199e-002):1.958827e-002,2:1.751724e-002):3.078517e-004,(38:3.327714e-001,39:1.503877e-001):9.350897e-002):1.780182e-001):7.541934e-002):4.084193e-002,27:1.797743e-001):4.398400e-003):2.465273e-001):2.515423e-001,((8:3.316769e-002,(((3:7.026234e-002,(15:1.385297e-002,14:3.769325e-002):2.217294e-002):7.812278e-002,9:3.027679e-002):6.491563e-002,((11:3.580816e-002,12:3.630962e-003):2.010885e-001,13:3.726265e-002):9.405486e-002):1.798183e-002):7.993881e-002,10:4.008805e-002):2.955787e-001):2.741931e-002):3.165253e-001):2.711301e-001):4.050356e-003):3.605102e-002,1:8.910999e-002);

tree gen.171000 = [&U] (((40:3.839589e-001,(4:8.398215e-002,5:2.078334e-002):2.164672e-002):1.604759e-001,(((((14:5.090055e-002,(3:6.561580e-003,15:8.353656e-003):2.508877e-002):1.970216e-001,(13:5.365197e-002,(11:1.157930e-001,12:4.706768e-003):4.452609e-001):5.111163e-002):1.781025e-001,(9:1.617177e-002,8:1.011287e-002):3.653327e-002):8.912398e-002,10:1.447476e-002):1.896529e-001,(18:1.902433e-001,((41:1.034544e+000,42:8.952133e-002):5.122337e-001,((30:6.530866e-003,27:1.352640e-001):2.966941e-002,(((((36:8.528559e-002,(21:3.927357e-002,(26:6.751781e-002,(20:1.318846e-001,(24:3.781959e-003,25:1.948775e-002):7.170859e-002):1.313413e-002):1.107692e-002):1.174965e-001):2.762583e-001,35:1.701566e-001):4.303036e-003,31:2.533444e-001):5.403042e-002,((2:2.983800e-002,(38:1.274403e-001,39:1.949456e-001):2.054513e-001):3.072256e-002,(23:1.274528e-002,(((34:1.103231e-001,(29:2.946313e-002,(33:1.763832e-001,28:1.726990e-001):3.887684e-002):6.888638e-002):5.306464e-003,32:1.778082e-001):6.177121e-002,(7:1.664151e-001,37:1.363190e-002):1.961099e-002):4.705940e-002):3.203996e-002):1.842197e-001):1.401779e-002,22:8.958391e-002):1.883567e-002):2.075917e-001):2.000571e-001):7.482879e-002):2.597694e-001):3.619216e-001,(((6:1.336880e-001,19:1.978617e-001):1.290917e-002,16:3.354510e-002):2.226305e-002,17:2.370694e-001):3.689087e-002,1:2.477345e-001);

tree gen.172000 = [&U] (((16:9.146207e-002,(6:7.384430e-002,19:1.409250e-001):1.135688e-001):7.919743e-002,17:2.548536e-001):3.382061e-002,(((4:7.717490e-002,5:1.198933e-001):5.039756e-002,40:2.469993e-001):1.495545e-001,((18:1.155808e-001,(((9:5.858689e-002,8:2.241394e-003):4.931961e-002,((13:7.714014e-003,(11:1.710874e-001,12:1.307743e-001):3.542157e-001):3.591558e-002,((3:2.260741e-003,15:1.922665e-002):2.876445e-003,14:1.367354e-002):2.450183e-001):4.070700e-003):1.895415e-002,10:9.064419e-002):1.468963e-001):9.889342e-002,((41:6.939820e-001,42:3.104030e-001):2.082038e-001,(((38:2.618438e-001,39:9.687688e-002):9.336780e-002,((23:3.400999e-002,((32:2.187042e-001,(34:1.017883e-001,((29:2.156573e-002,33:4.400003e-002):1.459202e-003,28:8.450622e-002):5.965531e-002):2.509405e-003):5.441352e-002,(7:1.535409e-001,37:2.919803e-002):1.201763e-001):2.960572e-002):1.172370e-001,2:3.393160e-002):7.972788e-002):1.535511e-001,((27:2.157820e-001,31:2.337451e-001):1.899362e-004,((22:8.191346e-002,30:4.820185e-002):1.695556e-003,((36:1.184502e-001,((20:2.343329e-002,21:4.255462e-002):3.287333e-002,(26:4.672510e-002,(24:9.650833e-002,25:1.438802e-002):4.330718e-002):2.203472e-003):8.897502e-002):1.830698e-001,35:1.569929e-001):5.061749e-002):2.290094e-003):3.742462e-002):4.177218e-001):4.988237e-002):2.357957e-001):3.034839e-001,1:2.508213e-001);

tree gen.173000 = [&U] ((((4:6.921543e-002,5:6.476404e-002):6.933402e-002,40:1.803155e-001):1.618679e-001,((18:1.990464e-001,((41:7.799320e-001,42:1.138094e-001):2.339899e-001,(22:9.108554e-002,(((((2:4.835115e-002,(23:7.845380e-002,((32:1.172798e-001,(34:1.872136e-001,((33:2.926140e-002,29:1.983207e-001):1.707894e-002,28:9.189395e-002):3.983532e-002):9.363111e-002):8.522670e-002,(7:2.648980e-001,37:2.252545e-003):8.591705e-002):5.328159e-002):5.026025e-002):3.193331e-002,(38:1.203463e-001,39:6.560020e-002):2.374502e-001):1.038543e-001,31:1.750067e-001):3.283946e-002,(((36:3.439315e-002,((24:5.706545e-002,25:9.593406e-003):1.621802e-001,(21:5.366431e-002,(20:1.137326e-003,26:1.597040e-001):1.210068e-001):8.638545e-002):1.040803e-001):2.120773e-001,35:1.838433e-001):5.697432e-002,30:5.414883e-002):2.537840e-004):1.427759e-003,27:2.750054e-001):6.730323e-004):4.821893e-001):2.945148e-001):5.692869e-002,(9:7.634419e-002,((10:1.019778e-001,((14:6.564058e-002,3:2.290178e-002):1.922185e-002,15:4.880100e-002):4.368786e-001):1.487403e-002,(8:1.455792e-002,(13:8.669398e-003,(11:1.082182e-001,12:2.240788e-002):3.712005e-001):5.044056e-002):6.252898e-002):1.343420e-001):2.479033e-001):3.282361e-001):3.410705e-001,(17:4.456098e-001,(19:2.322160e-001,(16:3.169223e-002,6:2.018555e-001):4.622701e-002):5.219372e-001):3.869117e-002,1:1.698634e-001);

tree gen.174000 = [&U] (((16:8.659845e-002,6:2.170799e-001):5.229607e-002,19:4.535775e-002):1.177411e-001,((((5:9.420516e-002,4:6.300356e-002):7.398827e-002,40:1.610478e-001):4.857577e-002,((18:1.769596e-001,((41:5.618254e-001,42:1.495935e-002):2.610942e-001,((31:1.029690e-001,((22:3.824328e-002,30:3.313879e-002):1.249284e-002,(((36:1.306246e-001,((((24:4.970451e-002,25:2.118645e-002):4.178590e-002,26:1.485883e-001):2.483018e-003,21:2.571018e-002):1.152127e-002,20:3.650891e-002):4.373768e-002):1.930474e-001,((38:1.245618e-001,39:3.131548e-002):1.182803e-001,((23:6.373510e-002,(((33:2.601446e-002,(28:2.040794e-001,29:2.095217e-002):4.498471e-002):1.339101e-001,(34:1.721853e-001,32:1.053766e-001):7.122135e-002):7.175618e-002,(7:1.932617e-001,37:1.437936e-002):1.013381e-001):4.514719e-002):5.027558e-002,2:2.443848e-002):7.924333e-002):1.075385e-001):3.551840e-002,35:1.660667e-001):1.643253e-004):4.473201e-002):5.124233e-002,27:4.125965e-002):4.160086e-001):7.364456e-002):9.587967e-002,(9:2.016906e-002,(((13:5.165949e-003,(11:8.306415e-002,12:6.285250e-002):4.145030e-001):9.346172e-002,8:1.113564e-001):5.227735e-002,(((15:9.554990e-003,3:3.604428e-002):3.804991e-002,14:5.024656e-003):8.128562e-002,10:4.639704e-002):2.876463e-001):8.111616e-002):2.203951e-001):2.982817e-001):2.536479e-001,17:3.775192e-001):6.859384e-002,1:1.859092e-001);

tree gen.175000 = [&U] ((((6:9.434873e-002,16:5.575047e-002):5.858661e-002,(((18:2.428539e-001,((41:4.822256e-001,42:3.924968e-002):3.854201e-001,(((30:9.393562e-002,(((23:6.899390e-002,(((((33:1.847749e-002,29:4.172285e-002):4.783238e-002,28:5.816818e-002):1.716705e-001,32:1.237295e-001):5.424495e-002,34:1.991303e-001):8.227824e-002,(7:3.027329e-001,37:1.112832e-002):1.158648e-001):5.014443e-002):2.755893e-002,(2:2.430385e-002,(38:1.876541e-001,39:3.761714e-002):8.832715e-002):9.772509e-002):6.515408e-002,(35:1.860004e-001,(36:1.147983e-002,(((21:8.006931e-002,26:1.053323e-001):1.057789e-003,20:4.103827e-002):4.898282e-002,(24:3.854795e-002,25:1.135589e-003):3.547387e-002):9.945329e-002):2.019385e-001):5.841188e-002):2.705736e-002):1.126011e-002,(22:3.482545e-002,31:3.762631e-001):2.028418e-002):1.897674e-002,27:1.144175e-001):6.090859e-001):9.064051e-002):1.596555e-002,(9:2.332529e-002,(10:6.493906e-002,((((15:3.640179e-002,3:1.049249e-001):4.818551e-002,14:5.643592e-002):1.502650e-001,(13:5.852296e-002,(11:6.348374e-002,12:2.507951e-002):1.070161e-001):3.788969e-002):1.834899e-001,8:1.962870e-001):2.844319e-002):1.482400e-001):7.526494e-002):3.467713e-001,((5:1.504072e-001,4:4.866442e-002):1.061729e-002,40:1.862500e-001):2.530098e-002):3.127905e-001):9.863844e-002,19:5.233316e-002):5.049402e-002,17:4.192868e-001,1:1.875918e-001);

tree gen.176000 = [&U] (17:3.946496e-001,(19:1.292060e-001,((6:1.654603e-001,16:8.635776e-003):2.728662e-002,(((18:2.008520e-002,((41:4.531381e-001,42:4.304637e-002):3.723293e-001,((((35:1.578702e-001,(31:6.981358e-002,27:1.001418e-001):2.212624e-002):2.919597e-002,30:6.273903e-002):1.875253e-002,((36:6.641535e-002,((26:6.068816e-002,(24:6.972578e-002,25:8.730195e-003):5.338052e-002):3.023858e-003,(20:2.421640e-002,21:7.491943e-002):2.499846e-002):1.447464e-001):1.687343e-001,22:4.620705e-002):4.810025e-002):3.413157e-002,((38:2.008049e-001,39:1.686692e-002):9.790058e-002,((23:9.238000e-004,(((((33:1.422138e-001,29:1.092755e-002):4.618066e-002,28:6.122703e-002):1.007185e-001,32:1.127023e-001):5.405253e-002,34:1.776907e-001):6.271011e-002,(7:3.038363e-001,37:2.097496e-003):7.808803e-002):1.786220e-001):4.734120e-002,2:1.325453e-002):1.094146e-001):9.967902e-002):2.547969e-001):7.197889e-002):2.670626e-002,(9:2.351094e-002,(((10:1.270953e-001,((15:2.089762e-002,14:4.112922e-003):1.499520e-002,3:6.548320e-002):1.380989e-001):7.938468e-002,(13:5.785927e-003,(11:5.782586e-002,12:2.675552e-002):8.801983e-002):6.819586e-002):6.388912e-002,8:3.648003e-002):2.276886e-001):2.416359e-001):1.818910e-001,(40:2.809963e-001,(5:3.985291e-002,4:5.556403e-002):1.525378e-001):2.506515e-002):2.389432e-001):2.137211e-002):5.081205e-002,1:1.085025e-001);

tree gen.177000 = [&U] ((((((41:3.715728e-001,42:8.627278e-002):2.281196e-001,(((30:4.744886e-002,((2:3.351462e-002,(23:5.885754e-004,((34:4.804767e-002,(32:9.090707e-002,((33:1.550990e-001,28:1.453881e-001):6.960996e-003,29:5.581860e-002):1.191501e-001):4.476422e-002):7.149972e-002,(7:2.515713e-001,37:2.982331e-003):2.899621e-002):1.336176e-002):1.056742e-002):2.073318e-002,(38:1.428824e-001,39:6.318327e-002):4.331639e-002):4.745177e-002):1.114748e-001,(((36:5.061143e-002,((20:1.019435e-002,((24:2.037341e-002,25:6.696096e-003):3.411927e-002,21:2.120704e-002):1.111003e-002):4.600019e-002,26:5.983899e-002):5.246570e-002):4.233663e-002,35:1.169575e-001):5.051322e-002,(22:1.244981e-001,27:8.587720e-002):7.461890e-003):4.765678e-002):2.125950e-002,31:5.513564e-002):2.216336e-001):6.247051e-002,18:6.331718e-002):4.812636e-002,(9:2.472287e-002,(8:4.708329e-002,((10:1.382705e-001,(13:4.790649e-003,(11:7.198704e-002,12:1.808627e-002):5.118142e-002):1.523570e-002):9.835029e-003,(15:1.255232e-003,(3:6.849023e-003,14:5.919609e-002):1.581688e-002):1.513409e-001):5.195677e-002):4.447617e-002):8.739801e-002):2.297427e-001,(5:4.712945e-002,(4:3.231282e-002,40:1.812152e-001):3.836298e-002):4.956091e-002):1.436047e-001,(17:1.223711e-001,((6:3.602531e-002,16:7.150275e-003):2.628030e-002,19:1.287494e-001):5.194576e-002):2.849390e-003,1:1.248102e-001);

tree gen.178000 = [&U] (((((((41:3.810805e-001,42:7.604261e-002):2.581778e-001,((((22:1.122905e-001,((2:7.406440e-002,((38:1.468831e-001,39:1.284116e-002):9.985900e-002,(23:1.732559e-002,(((28:2.196096e-002,(29:1.222099e-001,33:1.038410e-001):1.066130e-002):4.381629e-002,(32:6.347418e-002,34:1.126432e-001):6.677346e-003):4.469308e-002,(7:2.432392e-001,37:1.532152e-003):6.360557e-002):6.546314e-002):1.601242e-002):5.544694e-003):5.671322e-002,30:4.746998e-002):4.250062e-003):7.968562e-002,27:9.265993e-002):1.711039e-002,((36:2.828358e-002,(((24:6.380894e-003,25:8.355398e-003):3.796621e-002,(20:3.922960e-003,21:8.382406e-002):2.583555e-002):3.644302e-002,26:6.618297e-003):5.925273e-002):6.736300e-002,35:1.055158e-001):5.941517e-002):7.110844e-003,31:2.918943e-002):1.661608e-001):1.061388e-001,18:4.744814e-002):9.480178e-002,(9:9.154065e-003,(((10:1.150579e-001,(13:3.792814e-003,(11:7.382902e-002,12:2.640555e-002):9.614180e-002):2.827473e-002):1.124503e-002,(15:6.939953e-002,(3:7.639373e-003,14:1.894950e-002):2.509871e-002):5.576122e-002):8.765337e-004,8:3.275351e-003):3.224450e-002):9.404239e-002):5.572399e-002,(40:1.632857e-001,(4:2.773510e-002,5:1.244406e-002):2.379435e-002):5.116308e-002):1.403161e-001,19:1.473739e-001):2.593381e-002,(17:1.262954e-001,(16:8.140532e-003,6:1.410056e-001):2.943428e-002):1.599452e-002,1:8.395779e-002);

tree gen.179000 = [&U] (((((((41:3.600134e-001,42:3.941909e-002):8.066819e-002,((((27:4.806826e-002,((36:2.672000e-002,((20:1.390491e-003,21:7.386268e-002):1.953456e-003,(26:3.931221e-003,(24:3.517826e-003,25:1.469639e-003):4.621268e-002):2.762466e-002):5.651525e-002):8.293720e-002,35:9.968262e-002):4.505341e-002):1.649585e-003,(30:1.988350e-002,31:2.985243e-002):2.183172e-002):3.832354e-003,((23:4.940037e-002,((34:1.162185e-001,(((28:5.479996e-002,29:1.141034e-001):4.172517e-003,33:6.407645e-002):1.758407e-002,32:5.996518e-002):7.764054e-003):7.859273e-002,(7:2.835103e-001,37:1.141902e-001):1.660574e-001):2.458616e-002):7.388565e-002,(2:6.827047e-002,(38:1.387630e-001,39:8.775725e-002):1.182070e-002):8.954138e-003):6.638682e-002):6.550556e-003,22:2.122213e-002):1.564608e-001):8.590527e-002,18:3.826835e-002):3.930616e-002,(9:3.162836e-003,(10:1.080239e-001,((((14:3.373318e-002,3:7.508694e-003):2.847215e-002,15:9.177254e-002):4.110228e-002,(13:3.150892e-002,(11:4.921858e-002,12:2.516979e-003):1.199213e-001):8.283908e-003):1.028242e-001,8:8.619403e-004):1.601677e-002):5.867860e-002):3.277573e-002):8.138455e-002,((40:1.542589e-001,5:3.486868e-003):3.915069e-002,4:5.482693e-002):4.643144e-002):2.788098e-001,((19:1.436492e-001,6:8.693966e-002):9.408714e-004,16:3.558544e-002):4.422430e-002):1.735124e-002,17:1.193135e-001,1:8.057519e-002);

tree gen.180000 = [&U] ((((6:3.214671e-002,16:3.480386e-002):1.292651e-002,19:1.692508e-001):7.021580e-002,(((((41:4.110248e-001,42:5.049963e-002):8.662930e-002,(((36:2.900210e-002,((21:2.002147e-002,(20:7.837708e-002,(24:4.347848e-003,25:3.478871e-002):7.224918e-003):4.971387e-002):9.769551e-003,26:6.672194e-002):9.955325e-002):1.128826e-001,35:2.217314e-001):3.402147e-002,((31:1.235738e-001,(27:6.028784e-002,(22:3.388029e-002,(2:1.957089e-002,((23:4.199319e-003,(((32:1.652794e-001,34:1.368666e-001):7.242978e-004,((28:2.895035e-002,33:1.177536e-001):2.942582e-003,29:7.719732e-002):1.373488e-001):4.285664e-002,(7:3.224130e-001,37:6.961302e-002):6.618405e-002):4.951309e-002):3.815428e-002,(38:1.092801e-001,39:6.778245e-002):3.138078e-002):6.117915e-002):8.285691e-002):1.703580e-002):3.252016e-003):1.503797e-002,30:4.867099e-002):1.897198e-003):1.832294e-001):3.444410e-002,18:4.496890e-002):1.695805e-002,(9:2.588129e-002,(10:4.837463e-002,(8:1.953050e-002,((15:5.478143e-003,(14:4.468735e-002,3:7.001213e-002):8.335875e-003):1.362133e-001,(13:3.714682e-003,(11:6.415080e-002,12:1.436237e-002):1.177756e-001):8.560443e-003):8.240133e-002):4.773690e-003):5.403636e-002):5.253513e-002):9.919486e-002,(40:1.687583e-001,(4:1.174115e-002,5:2.238154e-002):5.332457e-002):2.844816e-002):3.124430e-001):7.315130e-003,17:1.252570e-001,1:1.377807e-001);

tree gen.181000 = [&U] (((6:1.343691e-001,(19:6.284590e-002,16:2.751966e-002):3.592031e-002):6.122423e-002,(((((41:5.651603e-001,42:7.052077e-002):1.045247e-001,(((30:1.403456e-001,35:3.191652e-001):1.120022e-002,31:1.319998e-001):4.835369e-002,(27:1.558077e-001,(((36:9.000910e-002,((20:4.315177e-002,21:7.488855e-003):1.996252e-002,((24:1.045442e-001,25:8.828725e-002):6.694460e-002,26:3.446548e-002):5.440190e-002):1.146233e-001):9.187768e-002,22:5.211052e-002):2.171787e-002,((2:5.870147e-002,(38:2.735720e-001,39:2.734020e-002):1.060969e-001):1.188724e-002,(23:1.233881e-002,((((33:1.427399e-001,29:5.660034e-002):3.803759e-002,28:5.586982e-002):1.269479e-001,7:2.414738e-001):1.428607e-002,(37:8.029638e-002,(34:7.212876e-002,32:1.925524e-001):8.649189e-002):8.156714e-003):7.231261e-002):1.487505e-001):7.010745e-002):3.315121e-002):1.487971e-002):1.917280e-001):3.255227e-001,18:5.387192e-002):2.744988e-002,(9:3.914081e-002,(8:8.550535e-003,(10:1.352773e-002,(((14:2.247433e-002,15:4.139132e-004):1.132570e-003,3:1.813314e-001):6.910194e-002,(13:3.868464e-003,(11:2.284154e-002,12:1.626204e-002):1.775463e-001):4.820454e-002):8.340525e-002):3.091048e-002):6.262480e-003):7.343497e-002):5.043166e-002,((40:2.005384e-001,5:8.096988e-002):2.608131e-002,4:1.916070e-001):2.018140e-002):1.818287e-001):1.013334e-002,17:1.016810e-001,1:1.563100e-001);

tree gen.182000 = [&U] ((((16:5.076944e-002,6:5.195454e-002):7.844397e-002,19:7.745279e-002):4.337671e-002,17:1.159831e-001):3.337546e-002,(((((41:4.504951e-001,42:7.537079e-002):2.759869e-001,((31:1.086539e-001,((22:6.713231e-002,(35:1.801103e-001,((2:4.654061e-002,(38:2.192701e-001,39:4.401186e-002):8.669889e-002):1.404173e-002,(23:2.790736e-002,(((((29:4.812339e-002,28:1.136816e-002):4.473182e-003,33:2.437310e-002):1.712824e-001,32:1.510674e-001):2.125492e-002,34:6.039360e-002):5.356377e-002,(37:7.054559e-002,7:1.937897e-001):2.296198e-002):1.153986e-001):3.700458e-002):5.102929e-002):2.257237e-002):1.231096e-002,((36:7.214299e-002,(((21:4.859405e-002,26:6.404053e-002):1.340607e-002,(25:3.378027e-002,24:3.620611e-002):5.664782e-002):3.176872e-002,20:8.731766e-003):9.404044e-002):3.555121e-002,30:1.174050e-001):4.093759e-003):3.856248e-002):3.203240e-002,27:6.610411e-002):3.024398e-001):1.006551e-001,18:4.924027e-002):3.719467e-002,(9:6.620944e-003,((((3:1.216940e-001,(15:2.073361e-003,14:2.851012e-002):5.004921e-002):2.818296e-002,10:1.084258e-002):6.243153e-002,(13:7.441436e-003,(11:1.830767e-002,12:4.158939e-003):1.422805e-001):4.958705e-002):4.176550e-002,8:9.732783e-003):3.967713e-002):1.422874e-001):4.042136e-002,((40:1.231722e-001,4:2.111082e-002):1.088385e-002,5:1.107503e-002):6.301618e-002):1.457371e-001,1:9.506813e-002);

tree gen.183000 = [&U] ((((((41:6.194996e-001,42:8.114354e-002):2.077855e-001,(((((((23:6.779542e-002,(((32:1.779854e-001,((29:5.433843e-002,28:1.261339e-002):2.245531e-003,33:3.165241e-002):4.866217e-002):4.251029e-002,34:6.501922e-002):2.884467e-002,(37:7.123980e-002,7:1.231987e-001):1.719604e-001):4.154329e-002):2.040334e-002,2:1.058293e-001):2.074323e-002,(38:2.295579e-001,39:6.610480e-002):1.997828e-001):1.155116e-001,27:7.202739e-002):2.439243e-002,30:6.642364e-002):3.674506e-002,((35:1.452843e-001,(36:3.497533e-002,(((25:5.333756e-002,24:2.190996e-003):5.236005e-002,21:5.629619e-003):1.756989e-002,(20:8.710529e-004,26:7.264396e-002):3.267646e-002):8.064071e-002):5.498671e-002):3.632165e-003,22:6.688870e-002):4.599564e-002):1.219600e-002,31:1.450283e-001):2.635025e-001):8.499954e-002,18:3.669004e-002):1.039095e-001,(9:1.148302e-003,(((13:1.429866e-002,(11:4.801672e-002,12:4.629377e-002):1.592758e-001):2.209892e-002,(10:2.136174e-001,((3:7.385904e-002,14:1.945023e-003):1.172830e-002,15:4.018280e-002):1.233343e-001):2.864107e-002):8.959544e-002,8:4.952661e-002):6.690375e-002):5.238712e-002):9.060036e-002,(40:1.320285e-001,(4:6.659177e-002,5:5.609724e-003):2.826160e-002):9.891275e-002):1.736015e-001,((17:1.607917e-001,19:2.601657e-002):2.246123e-002,(6:1.654623e-002,16:1.122517e-002):1.105216e-001):1.240590e-002,1:1.449247e-001);

tree gen.184000 = [&U] (17:1.467935e-001,((19:5.762272e-002,(((((41:5.362569e-001,42:8.325020e-002):2.807325e-001,(22:3.708537e-002,((35:1.791132e-001,27:1.594988e-001):1.386718e-002,((31:1.272320e-001,(((38:2.477450e-001,39:1.573800e-001):4.677380e-002,((23:6.116907e-002,(((((33:1.053180e-001,28:1.511952e-002):7.118673e-002,29:3.544181e-002):4.234479e-002,32:2.226361e-001):6.046320e-002,34:8.400901e-002):4.114034e-002,(37:7.973604e-004,7:1.192132e-001):5.665277e-002):1.600291e-001):7.346512e-002,2:1.304713e-001):2.529002e-002):4.146705e-002,(36:4.935840e-002,((21:1.653331e-002,(26:7.666166e-002,20:3.124358e-003):7.332500e-002):2.783828e-002,(25:6.586094e-002,24:1.591171e-002):1.824315e-002):1.191901e-001):1.223848e-001):1.256592e-001):3.089474e-002,30:7.443286e-002):5.751700e-002):9.850781e-003):1.417185e-001):1.636186e-001,18:1.926399e-001):2.835976e-002,(9:2.967805e-003,(((14:7.033793e-002,3:7.760020e-002):5.452445e-002,15:3.951924e-002):1.787635e-001,(((13:3.809941e-003,(11:5.919723e-002,12:2.470340e-002):1.121623e-001):7.604515e-002,8:2.837289e-003):4.029469e-002,10:1.220288e-001):3.971682e-002):5.901163e-003):1.004288e-001):1.380593e-001,((5:4.743331e-002,4:5.776785e-002):7.511724e-002,40:1.629293e-001):1.006373e-001):2.130137e-001):1.382980e-002,(6:5.677543e-002,16:4.163366e-002):4.059338e-002):3.851599e-002,1:3.207853e-001);

tree gen.185000 = [&U] ((19:2.221222e-001,17:1.702365e-001):7.715840e-003,((6:1.448559e-001,(((((41:6.660230e-001,42:2.081473e-001):2.618506e-001,((2:4.388587e-002,((38:1.593899e-001,39:1.719645e-001):1.401972e-001,(23:6.231585e-003,(((34:9.026305e-002,32:2.432679e-001):4.773633e-002,((29:5.277726e-003,33:3.382834e-002):4.924426e-002,28:7.022838e-002):2.617194e-001):4.761020e-002,(37:6.972835e-002,7:1.058430e-001):1.053805e-001):2.517360e-002):1.564348e-002):1.563188e-003):2.289034e-001,((31:1.453427e-001,22:4.665066e-002):2.682017e-002,(((27:1.678800e-001,30:9.888015e-002):3.118459e-002,35:2.985057e-001):5.051218e-003,(36:5.393246e-002,(((25:2.496112e-002,24:9.308616e-003):9.035368e-003,20:8.630421e-003):2.168819e-002,(26:6.299875e-002,21:1.304758e-002):2.281362e-002):1.200994e-001):1.454187e-001):3.887598e-002):5.218680e-002):4.462761e-001):1.048808e-001,18:8.826514e-002):3.303928e-002,((((15:4.230335e-002,(14:3.745896e-002,3:4.215596e-002):3.475625e-002):2.691348e-001,(10:1.284846e-001,8:3.346406e-003):6.763956e-003):5.983424e-003,(13:1.038075e-001,(11:6.468307e-002,12:2.699267e-002):1.604943e-001):3.521132e-002):1.910828e-002,9:1.715292e-002):1.425071e-001):8.588470e-002,(40:2.212503e-001,(5:6.452718e-002,4:8.303448e-003):7.835550e-002):6.044220e-002):2.551602e-001):1.214055e-002,16:4.549187e-002):1.964717e-002,1:2.138028e-001);

tree gen.186000 = [&U] ((6:7.979095e-002,16:4.449515e-002):8.482032e-002,(19:1.707232e-001,(17:1.214210e-001,(((((41:6.204642e-001,42:8.532125e-002):2.578362e-001,(((30:7.175802e-002,((39:6.062785e-002,2:4.164958e-002):2.868365e-002,(38:2.553383e-001,(23:4.528784e-002,(((29:5.782747e-003,(28:6.368644e-002,33:2.600937e-002):2.619336e-002):2.893804e-002,(34:3.010696e-001,32:2.369175e-001):6.676064e-003):1.491148e-002,(37:1.000624e-001,7:1.235797e-001):8.516971e-002):5.573133e-002):1.876562e-002):6.318244e-003):6.121745e-002):1.249640e-001,(36:4.503016e-002,(((20:7.170347e-003,21:1.249311e-002):1.210532e-002,(25:6.546946e-003,24:1.984442e-003):5.518900e-002):1.045675e-002,26:5.103848e-002):1.915170e-001):1.236684e-001):1.196118e-001,(((31:1.270670e-001,35:1.146035e-001):1.562757e-003,22:4.165259e-002):6.436056e-003,27:3.521605e-001):4.738918e-002):4.090441e-001):8.728692e-002,18:1.156590e-001):4.388546e-002,((8:3.234115e-002,(((13:3.224805e-003,(11:3.153896e-002,12:4.728066e-003):1.493290e-001):7.344208e-002,10:1.532200e-001):2.684155e-004,((15:3.800082e-002,14:6.850496e-003):3.591008e-002,3:4.425496e-003):2.482829e-001):9.759154e-003):3.191431e-002,9:1.597959e-002):1.373584e-001):6.989315e-002,(40:2.546244e-001,(4:5.079851e-002,5:4.555476e-002):3.374358e-003):1.736174e-001):2.229502e-001):3.399466e-002):1.314291e-001,1:1.264595e-001);

tree gen.187000 = [&U] ((19:1.061932e-001,(6:9.669586e-002,16:1.012254e-001):5.268009e-002):1.399212e-003,((((((41:4.975393e-001,42:2.035783e-001):1.479414e-001,((30:4.031021e-002,((35:1.246865e-001,(36:6.826598e-002,(20:9.569483e-002,(((25:1.799607e-002,24:5.091241e-003):4.724992e-002,26:6.176974e-002):2.693084e-003,21:1.889385e-002):8.032503e-003):1.351383e-001):9.185826e-002):3.512022e-002,22:6.922394e-002):6.279910e-003):8.797934e-002,((27:2.740915e-001,(38:2.003274e-001,((2:1.134917e-001,(23:1.155903e-002,((((28:1.544275e-001,(29:1.002393e-002,33:2.180266e-002):9.419404e-003):1.136559e-001,34:1.671046e-001):5.063918e-002,32:1.874842e-001):1.498698e-002,(37:1.025001e-001,7:9.501884e-002):8.433811e-002):7.963852e-003):6.073241e-002):1.687063e-002,39:8.595121e-002):1.994257e-002):2.215318e-001):1.884157e-003,31:8.322031e-002):7.021236e-002):3.231838e-001):9.302988e-002,18:3.824774e-002):9.497177e-002,(((13:3.296097e-002,(11:5.659061e-002,12:6.796618e-002):7.381695e-002):7.466795e-002,(10:1.305292e-001,(((3:6.575272e-002,15:2.716442e-002):1.563623e-003,14:1.286622e-002):1.255878e-001,8:1.181424e-002):1.344404e-002):1.653675e-002):3.562742e-002,9:1.745835e-002):1.459147e-001):1.164887e-001,((4:4.136453e-002,40:2.931551e-001):2.486977e-002,5:3.686871e-002):3.755183e-002):1.787798e-001,17:1.723945e-001):2.688718e-002,1:1.305225e-001);

tree gen.188000 = [&U] (((((((41:7.035557e-001,42:1.024482e-001):2.302771e-001,(31:1.272016e-001,(((((2:1.690186e-002,38:2.588913e-001):3.520324e-002,39:6.322731e-002):1.439057e-001,(23:5.135524e-002,((32:2.050107e-001,(34:1.069069e-001,((28:1.422422e-001,29:2.287368e-002):1.662089e-002,33:1.766819e-001):2.384790e-001):3.605202e-002):4.044183e-002,(37:9.772477e-002,7:4.574328e-001):2.038976e-002):6.529397e-002):7.198615e-002):9.197199e-002,((22:5.609495e-002,(35:1.182710e-001,(36:9.653290e-002,((((25:2.357536e-002,24:1.442927e-002):1.309199e-001,21:1.379864e-001):1.346010e-002,26:6.896923e-002):7.737146e-003,20:5.095251e-003):1.012192e-001):8.604855e-002):1.425101e-001):1.116470e-002,27:1.632659e-001):4.100710e-002):3.288386e-002,30:1.535895e-002):5.790675e-003):2.503081e-001):1.576184e-001,18:8.100942e-002):1.327789e-001,((((13:9.429614e-005,(11:8.002311e-002,12:5.877569e-002):1.644797e-001):2.303158e-002,(3:2.688934e-002,(14:4.092818e-002,15:1.701885e-002):8.311306e-004):1.374208e-001):1.240399e-002,(10:6.930867e-002,8:2.366938e-003):3.111869e-002):5.042251e-002,9:4.619095e-002):7.702424e-002):2.421087e-001,((4:5.549106e-002,5:1.643760e-002):5.069517e-002,40:2.776669e-001):1.184329e-001):2.528073e-001,17:1.560966e-001):9.354764e-003,((6:1.061505e-001,16:1.941438e-002):5.399203e-002,19:7.402107e-002):1.647174e-001,1:2.012839e-001);

tree gen.189000 = [&U] (((((((41:3.958206e-001,42:1.346908e-001):2.373961e-001,(31:9.357095e-002,(((2:1.657905e-002,(39:1.128193e-001,38:1.382936e-001):4.225332e-002):6.865911e-004,(23:3.421373e-005,((32:1.852740e-001,((33:2.500831e-002,(29:1.063253e-001,28:1.239306e-001):1.833901e-002):9.463351e-002,34:1.160786e-001):2.914502e-002):9.007508e-002,(37:9.355774e-002,7:6.579587e-002):2.555092e-002):8.407180e-002):3.495080e-002):6.614984e-002,((35:1.528674e-001,(36:9.241669e-002,(26:1.160349e-001,((20:7.657261e-003,(25:2.401191e-002,24:2.247688e-003):1.809460e-001):5.239031e-003,21:8.223501e-003):9.586432e-002):3.194167e-001):4.443045e-002):8.984949e-002,(30:4.464423e-002,(27:1.801160e-001,22:4.922156e-002):7.599452e-003):4.487500e-002):2.563157e-002):7.088614e-002):2.472831e-001):9.767546e-002,18:8.255672e-002):4.115848e-002,((8:5.558356e-002,((13:6.632194e-003,(11:7.059710e-002,12:5.626947e-002):1.781634e-001):2.205052e-002,(((15:3.536788e-002,14:1.007858e-002):7.351186e-002,3:1.592740e-002):1.002285e-001,10:4.604915e-002):1.667589e-001):7.440234e-002):1.773927e-001,9:1.246322e-002):2.864459e-002):4.874621e-002,((4:7.155970e-002,40:2.688141e-001):1.142626e-001,5:7.273815e-003):2.149251e-001):2.587252e-001,17:1.435975e-001):7.500838e-003,(6:9.551248e-002,(16:2.259765e-002,19:9.238197e-002):4.365125e-003):1.072518e-001,1:1.428354e-001);

tree gen.190000 = [&U] (17:1.523403e-001,((((((41:5.341181e-001,42:2.614358e-001):4.189694e-001,(30:7.098134e-002,(((((23:4.992115e-003,(((((29:6.527441e-002,28:6.445212e-002):1.935378e-002,33:4.361562e-002):1.131984e-001,34:2.003629e-001):1.468725e-002,32:1.686269e-001):5.743149e-002,(37:4.523957e-002,7:1.797979e-001):1.748541e-001):2.724550e-002):7.177624e-002,(39:9.947860e-002,38:1.356126e-001):5.976848e-002):2.569584e-002,2:2.094694e-002):1.853694e-001,31:9.405450e-002):4.533772e-002,((35:1.655896e-001,22:7.358572e-002):3.735693e-002,((36:1.151397e-001,((21:7.886353e-003,((25:1.046914e-002,24:1.047397e-001):2.262802e-001,20:1.070361e-001):1.169276e-002):4.239899e-002,26:1.080007e-001):8.261656e-002):5.408953e-002,27:2.171044e-001):5.414916e-002):5.880162e-002):7.532711e-003):3.303865e-001):9.479572e-002,18:1.302308e-001):1.084778e-001,(((((13:1.675334e-003,(11:2.037501e-001,12:5.338606e-002):2.192176e-001):5.704813e-002,(3:9.105816e-002,(14:2.426686e-003,15:4.818339e-002):1.692495e-002):1.636821e-001):1.386975e-002,8:7.277983e-002):1.326300e-001,10:1.460376e-001):4.844803e-002,9:1.486967e-003):1.327901e-001):6.380586e-002,(40:4.226992e-001,(4:1.046824e-002,5:2.781216e-002):1.122734e-002):8.184027e-002):4.979859e-001,((16:2.537061e-001,6:1.189966e-001):4.857908e-002,19:5.407673e-002):7.074064e-002):1.635154e-002,1:7.013516e-002);

tree gen.191000 = [&U] (((((((41:5.533970e-001,42:3.094208e-001):4.141299e-001,(27:9.252327e-002,(((30:8.210304e-002,31:2.112383e-001):1.970876e-003,(22:1.760799e-001,(((23:2.327615e-002,(((33:5.177679e-002,(28:4.487253e-002,29:1.491090e-001):1.939402e-002):1.422348e-001,(34:2.351447e-001,32:1.412859e-001):1.816338e-002):5.469598e-002,(37:3.583583e-002,7:3.424876e-001):1.859406e-001):2.465378e-002):4.079121e-002,(39:2.637878e-001,38:4.932358e-001):4.406202e-002):2.297584e-002,2:1.332684e-001):1.872026e-001):4.369107e-002):5.265858e-002,(36:2.087474e-001,(21:8.065605e-003,((25:4.539097e-002,24:6.741739e-002):1.071642e-001,(20:2.634695e-002,(26:1.259720e-001,35:3.327884e-001):5.719293e-002):5.127942e-004):4.329504e-002):1.442941e-001):1.397841e-001):1.375482e-002):4.066330e-001):1.048581e-001,18:1.541338e-001):1.042304e-001,(((10:1.637481e-001,(13:2.028564e-001,(11:1.019871e-001,12:2.584129e-003):3.606764e-001):1.240889e-001):2.402273e-002,(((15:4.483179e-002,14:7.425359e-002):2.852588e-002,3:2.932241e-002):1.978237e-001,8:5.170013e-002):1.265541e-003):1.251108e-001,9:4.472815e-002):4.597372e-001):2.973037e-001,((5:9.286673e-002,40:5.505618e-001):3.748274e-003,4:6.631749e-002):2.093755e-001):2.735517e-001,(19:8.058748e-003,(16:8.428477e-003,6:1.302227e-001):4.325811e-002):1.975434e-002):5.788218e-002,17:1.708151e-001,1:8.204698e-002);

tree gen.192000 = [&U] (17:1.229355e-001,((((((41:4.107221e-001,42:2.296470e-001):3.610049e-001,((22:4.714270e-002,((((36:4.908771e-002,(26:9.201579e-002,(20:3.274168e-002,((25:1.123376e-002,24:1.251530e-002):2.149509e-002,21:6.066013e-002):2.373567e-003):7.724687e-003):6.715434e-002):2.248222e-001,35:2.469901e-001):4.523887e-002,31:1.954994e-001):6.410403e-003,30:2.427133e-002):2.509615e-002):5.265498e-002,(27:1.426974e-001,(((23:3.742346e-002,((((33:2.132337e-002,28:1.433647e-001):3.373074e-002,29:2.001089e-001):1.016253e-001,(34:1.745205e-001,32:1.427586e-001):1.086995e-003):9.858044e-002,(37:5.845055e-003,7:3.523129e-001):1.251114e-001):6.221292e-002):1.406969e-001,(38:2.256399e-001,39:5.775523e-002):3.030374e-001):1.145247e-002,2:1.300740e-001):1.291863e-001):8.064878e-002):2.638102e-001):1.682529e-001,18:5.536970e-002):9.649281e-002,(((3:7.469144e-002,(15:9.173743e-002,14:1.290817e-001):2.051510e-002):1.094480e-001,(10:7.606530e-002,((13:5.284421e-004,(11:5.108898e-002,12:1.890737e-002):3.178166e-001):1.482222e-002,8:9.136020e-004):1.802848e-002):2.201257e-002):3.311370e-002,9:1.221760e-001):6.883454e-002):3.162711e-001,(5:1.177161e-002,(40:2.052917e-001,4:4.045765e-002):5.995881e-002):1.436656e-001):2.378437e-001,(19:3.861126e-002,(16:1.222003e-001,6:7.449134e-002):1.366694e-001):4.147117e-002):4.412326e-002,1:9.063967e-002);

tree gen.193000 = [&U] ((19:1.462763e-001,((16:9.693031e-002,17:1.832921e-001):2.041882e-002,6:1.050358e-001):1.312203e-001):1.481669e-002,(((((41:5.791348e-001,42:3.532442e-001):3.479861e-001,(((23:5.228321e-003,((((29:2.821616e-001,33:1.243051e-001):2.821942e-002,28:2.014956e-001):1.274119e-001,(34:2.412560e-001,32:2.941050e-001):1.241129e-002):8.430875e-002,(37:4.014886e-002,7:3.486742e-001):1.551380e-001):1.311986e-001):9.616893e-002,((38:1.365856e-001,39:1.288654e-001):1.277887e-001,2:1.190328e-001):6.538979e-002):3.524315e-001,((22:6.621229e-002,27:2.060376e-001):2.935675e-002,(31:2.537502e-001,(30:4.644883e-002,((36:2.849753e-001,((21:4.690459e-002,(20:1.606337e-002,26:1.653445e-001):1.287551e-002):2.739869e-002,(25:2.065373e-001,24:1.448850e-002):1.184748e-001):1.643058e-001):5.871054e-002,35:2.788915e-001):1.572441e-001):2.178107e-002):2.732894e-002):4.492053e-002):4.600420e-001):8.849944e-002,18:1.756944e-001):3.239574e-002,(((8:2.313469e-002,((3:5.114184e-002,15:4.579743e-002):3.739821e-002,14:2.236740e-002):4.091389e-001):1.271313e-001,((13:7.058021e-004,(11:9.725581e-002,12:2.175209e-002):3.199566e-001):2.580031e-002,10:1.643872e-001):6.501863e-002):4.152170e-002,9:6.932422e-002):1.223651e-001):1.682081e-001,((5:4.626707e-002,4:1.723549e-002):3.132078e-002,40:2.801252e-001):8.025938e-002):3.442968e-001,1:2.543285e-001);

tree gen.194000 = [&U] (17:1.572723e-001,((((((41:5.878653e-001,42:1.715597e-001):3.003948e-001,(31:6.690788e-002,(27:2.472994e-001,(30:2.511640e-002,(((36:1.020476e-001,(21:1.919674e-002,(26:4.184871e-002,((25:2.937582e-005,24:7.018113e-002):4.802936e-002,20:8.446993e-003):4.501215e-002):4.266020e-003):1.304108e-001):1.866781e-001,35:2.340602e-001):1.286259e-001,(22:9.600687e-002,((23:4.247975e-002,(((((29:3.048686e-001,33:5.431440e-002):7.096528e-003,28:5.808474e-002):1.244099e-001,32:2.637295e-001):1.590528e-002,34:1.831240e-001):2.569486e-002,(37:3.026051e-002,7:2.465133e-001):8.648100e-002):1.703281e-001):6.839462e-002,((38:1.260933e-001,39:7.659881e-002):2.280028e-002,2:9.733101e-002):4.624906e-002):2.678021e-001):4.081120e-002):1.400783e-001):4.938066e-003):5.259699e-002):4.005074e-001):2.063953e-001,18:1.198134e-001):7.645002e-002,(((((3:5.242274e-002,15:1.430714e-002):5.666169e-002,14:1.627515e-002):1.428691e-001,10:2.183583e-001):1.137285e-001,(8:5.636121e-002,(13:3.068979e-002,(11:7.652939e-002,12:5.878439e-002):2.249661e-001):4.025939e-002):1.036204e-001):6.387420e-002,9:3.696242e-003):6.093923e-002):6.856188e-002,(40:3.162130e-001,(4:6.176961e-002,5:6.415332e-002):4.124688e-002):1.365999e-001):2.851866e-001,((19:7.641979e-002,16:6.182896e-002):1.200777e-002,6:5.565173e-002):8.997283e-002):4.312048e-002,1:2.363041e-001);

tree gen.195000 = [&U] (17:1.476941e-001,(((16:1.937000e-002,6:1.215429e-001):7.156506e-002,19:7.713958e-002):1.704746e-002,(((((41:5.668009e-001,42:1.659044e-001):3.598774e-001,(((31:6.480354e-002,27:9.599273e-002):3.191619e-002,((22:3.121479e-002,((38:3.318198e-001,39:2.097450e-001):1.061442e-001,((23:6.933345e-002,((32:3.465950e-001,(((33:5.028481e-002,28:1.045731e-001):2.037903e-002,29:1.522270e-001):6.324584e-002,34:1.960042e-001):4.431337e-002):5.832493e-002,(37:8.831397e-002,7:2.049106e-001):8.336901e-002):6.941590e-002):4.074546e-002,2:9.725701e-002):1.333556e-001):1.478938e-001):7.085241e-002,((36:8.930497e-002,(21:7.381320e-003,((20:2.173594e-002,(25:8.750397e-002,24:4.835133e-002):1.484188e-001):1.990787e-002,26:2.085414e-001):6.397906e-003):7.596867e-002):1.082807e-001,35:3.752039e-001):4.175296e-002):1.457614e-003):2.075003e-002,30:8.186879e-002):4.243732e-001):1.528391e-001,18:9.668040e-002):1.621677e-001,((8:6.547620e-003,((13:7.320340e-002,(11:7.378719e-002,12:2.186258e-003):2.909766e-001):3.534579e-002,(10:8.041092e-002,((15:1.175428e-002,3:1.402055e-003):2.681352e-002,14:3.590467e-003):3.290573e-001):6.956229e-002):6.984196e-002):2.757102e-002,9:1.978152e-002):1.776730e-001):2.407618e-001,(40:4.807592e-001,(4:4.887783e-002,5:6.017339e-002):1.165296e-001):2.878524e-002):4.922408e-001):4.258444e-002,1:1.966170e-001);

tree gen.196000 = [&U] (((19:1.789885e-002,6:1.905446e-001):9.893147e-002,16:4.279543e-002):3.436841e-002,(17:1.403276e-001,(((((41:6.405544e-001,42:1.587736e-001):2.620663e-001,((22:6.109397e-002,((((2:9.307676e-002,(38:2.702578e-001,39:2.007299e-001):1.412248e-001):2.518437e-004,(23:3.223449e-002,(((28:2.172912e-001,(33:8.591181e-002,29:1.460228e-002):2.256129e-002):5.416690e-002,(34:1.330690e-001,32:3.316978e-001):3.297362e-002):2.760920e-002,(37:1.944718e-002,7:1.644025e-001):4.627472e-002):1.419246e-001):4.233643e-002):1.266554e-001,30:3.984348e-002):6.608007e-002,((36:1.051751e-002,(20:3.439803e-002,(26:8.719937e-002,((25:4.149724e-002,24:3.091218e-002):4.834279e-002,21:7.132417e-003):1.381874e-002):2.217430e-002):7.166815e-002):3.126438e-001,35:3.102873e-001):4.163627e-002):8.134437e-003):1.372974e-002,(27:2.436073e-001,31:6.245668e-002):8.210738e-002):1.678857e-001):1.342797e-001,18:4.201084e-002):1.474591e-002,((((13:1.184821e-002,(11:3.208847e-002,12:2.229709e-002):2.304799e-001):7.955388e-002,8:4.810273e-002):1.569205e-002,(((3:7.694126e-004,15:4.012601e-002):4.642870e-002,14:1.320690e-001):1.392870e-001,10:7.360803e-002):1.628082e-002):2.701455e-002,9:4.122187e-002):2.224582e-001):1.544943e-001,(4:7.341226e-002,(40:2.389458e-001,5:4.315658e-002):8.294945e-003):1.398581e-001):1.457889e-001):4.823049e-002,1:6.871415e-002);

tree gen.197000 = [&U] ((((((41:7.368700e-001,42:1.744127e-001):1.988228e-001,((22:5.581328e-002,((31:5.846964e-002,((36:5.109461e-002,(((21:2.480160e-002,(25:3.708374e-002,24:2.526294e-002):2.980681e-001):7.120937e-002,20:1.456831e-003):6.803769e-002,26:4.639351e-002):1.045944e-001):5.992597e-002,35:1.768575e-001):1.088350e-001):5.069287e-002,(((38:2.287928e-001,39:6.442537e-002):1.489658e-001,2:6.778569e-002):1.315866e-001,(23:2.782901e-002,(((32:4.175827e-001,((33:1.091438e-001,29:6.177696e-002):1.467808e-002,28:2.647948e-001):4.501000e-002):5.285767e-002,34:2.146250e-001):7.595695e-002,(37:6.969261e-003,7:2.153320e-001):1.161896e-001):5.826261e-002):4.461365e-002):2.609000e-001):7.815828e-002):3.902640e-002,(30:1.567300e-001,27:3.407615e-002):1.223411e-002):9.676368e-002):1.518701e-001,18:5.502518e-002):9.294348e-002,((8:2.763056e-002,((((15:6.108328e-003,3:7.353077e-002):6.436377e-003,14:1.771539e-001):2.895669e-001,10:9.856189e-002):1.109081e-001,(13:7.290345e-003,(11:3.828493e-002,12:7.703160e-002):3.767349e-001):2.046374e-001):2.663565e-003):1.779732e-001,9:1.055196e-001):1.972113e-001):2.066800e-001,((5:3.250633e-002,4:9.849620e-002):1.143428e-001,40:2.895567e-001):1.604321e-001):3.221094e-001,((19:2.377037e-002,17:1.332115e-001):1.277272e-001,(16:6.091607e-002,6:2.390699e-001):7.678689e-002):5.675501e-002,1:3.493122e-001);

tree gen.198000 = [&U] (17:2.466982e-001,((6:2.001150e-001,16:2.707280e-002):7.418580e-002,((((((41:6.790142e-001,42:6.152258e-002):5.849664e-001,((30:8.790697e-002,(27:2.602480e-001,(22:2.063480e-002,((((38:9.723994e-002,39:1.231970e-001):1.029088e-001,2:3.343020e-002):4.775589e-002,(23:2.168852e-002,((34:1.621323e-001,(32:2.150270e-001,(28:2.090974e-001,(33:1.167235e-001,29:1.030268e-001):5.160024e-003):1.385134e-001):1.847818e-001):3.779551e-002,(37:2.847269e-003,7:4.321907e-002):1.326285e-001):2.625812e-002):5.042198e-002):2.037990e-001,31:7.054640e-002):1.013827e-002):1.513023e-001):2.470344e-002):6.016651e-002,((36:3.831009e-002,((20:3.898101e-003,26:1.990039e-001):2.744449e-002,(21:1.931484e-002,(24:6.113975e-003,25:5.567873e-002):1.275242e-001):6.159299e-002):1.085591e-001):3.927375e-002,35:1.311133e-001):5.838017e-002):1.200474e-001):1.952521e-001,18:1.127379e-001):1.487376e-001,(((13:8.704450e-002,(11:2.983733e-002,12:1.941497e-002):4.481080e-001):3.403240e-002,(((3:2.399823e-002,(14:3.220999e-002,15:1.303085e-003):9.678996e-002):4.993457e-002,10:4.408968e-002):1.182490e-001,8:3.859159e-002):6.075429e-002):8.422740e-002,9:1.024904e-001):2.096429e-001):1.316789e-001,((40:2.078126e-001,4:1.938473e-002):1.688673e-003,5:7.911365e-003):2.188222e-001):2.081437e-001,19:1.852541e-002):2.363595e-002):3.844248e-002,1:2.292546e-001);

tree gen.199000 = [&U] (17:1.935927e-001,((19:1.348539e-002,(((((41:8.237780e-001,42:4.530100e-001):4.187677e-001,(((27:2.104010e-001,(((23:2.166797e-003,((34:1.901350e-001,(32:8.750709e-002,((29:1.673841e-001,28:2.367985e-001):2.390694e-002,33:1.367828e-001):6.472650e-002):5.414747e-002):1.862222e-001,(37:1.611156e-002,7:5.064639e-002):1.304352e-001):9.505947e-002):4.408167e-002,2:7.640778e-002):3.502913e-002,(38:2.066032e-001,39:4.169037e-002):1.394470e-001):1.497120e-001):8.725116e-002,((30:2.869420e-002,31:8.080603e-002):1.811495e-002,((36:1.547066e-002,(((24:7.605141e-003,25:2.905272e-002):2.163426e-002,21:3.384744e-002):3.715260e-002,(20:1.120080e-002,26:1.395586e-001):5.643576e-002):7.055457e-002):3.461189e-002,35:1.133291e-001):9.748870e-002):3.527962e-002):4.992251e-002,22:3.652697e-002):2.632927e-001):1.746353e-001,18:2.198564e-001):8.608812e-002,((8:2.321994e-002,(10:9.339880e-002,(((15:4.793109e-003,3:7.781721e-002):5.769335e-002,14:3.774538e-002):5.147955e-002,(13:5.032776e-002,(11:1.238053e-001,12:1.021791e-001):4.942801e-001):5.658637e-002):2.071860e-002):2.035543e-001):9.999147e-002,9:7.957640e-002):1.879214e-001):8.194875e-002,((5:6.394224e-002,4:3.398704e-002):6.116029e-003,40:2.709580e-001):5.538790e-002):2.224885e-001):4.025759e-002,(16:1.002827e-001,6:9.765760e-002):1.367745e-001):5.995271e-002,1:3.371783e-001);

tree gen.200000 = [&U] ((19:9.984343e-002,(((((41:1.090610e+000,42:9.410930e-002):6.811305e-001,(((23:1.090546e-002,(((32:7.958635e-002,(28:2.631407e-001,(29:1.148561e-001,33:1.857990e-001):2.038866e-002):1.424964e-001):6.078587e-003,34:2.177207e-001):1.563111e-001,(37:4.930617e-002,7:1.090955e-001):2.159165e-001):1.270273e-001):8.569061e-002,((38:2.245040e-001,39:6.401164e-002):4.896255e-002,2:7.452879e-002):2.815541e-002):1.132190e-001,(30:3.484034e-002,((22:5.241879e-002,(27:2.319767e-001,((36:4.747574e-002,((26:1.084423e-001,(20:3.476363e-002,(24:1.674752e-002,25:3.418302e-002):5.082438e-002):6.137864e-002):2.804257e-002,21:1.411781e-002):1.092611e-001):6.458637e-002,35:2.005399e-001):1.065781e-001):3.598617e-002):4.669766e-002,31:2.556726e-001):1.427023e-002):9.637308e-002):3.174657e-001):6.642399e-002,18:1.738072e-001):1.531412e-001,((8:7.122004e-002,((13:4.973237e-002,(11:1.230850e-001,12:5.822638e-002):1.274303e-001):8.051953e-003,(((15:1.569319e-002,14:8.786550e-003):2.950728e-002,3:1.148914e-002):1.238066e-001,10:1.207394e-001):7.798141e-002):9.946301e-002):5.491864e-002,9:3.303763e-003):1.027560e-001):1.929528e-001,((5:1.152234e-004,40:4.258084e-001):4.640460e-003,4:3.926616e-002):1.990969e-001):5.501135e-001):1.157005e-001,((16:1.124685e-001,6:8.203136e-002):1.263779e-001,17:2.093611e-001):4.563374e-002,1:3.750623e-001);

tree gen.201000 = [&U] ((((((41:1.387674e+000,42:5.365451e-001):5.630676e-001,(((30:3.749762e-002,(((38:2.685462e-001,39:2.623134e-001):1.804570e-002,(2:9.181284e-002,(23:1.144687e-002,((((29:4.564269e-002,28:3.436662e-001):4.020119e-003,33:1.487861e-001):1.681892e-001,(32:2.854936e-001,34:1.023432e-001):4.714497e-002):7.067413e-002,(37:1.422645e-002,7:1.388113e-001):7.756424e-002):6.004644e-002):6.070336e-002):3.771726e-002):1.679071e-001,27:2.888532e-001):7.250693e-002):3.964721e-002,31:2.984405e-001):1.845256e-002,(((36:1.612304e-001,((21:1.827759e-002,(24:3.273967e-002,25:4.200793e-002):1.368605e-001):1.371344e-002,(26:2.037420e-001,20:3.763750e-003):2.126209e-002):2.194949e-001):8.904927e-002,35:1.295070e-001):2.294966e-002,22:4.147123e-002):1.141049e-001):4.054196e-001):1.745924e-001,18:1.587838e-001):1.122801e-001,(((((14:5.050914e-002,(3:2.941677e-002,15:1.744328e-002):8.424200e-002):4.531475e-001,(13:2.199618e-002,(11:1.129358e-001,12:3.143627e-002):2.437563e-001):1.575598e-002):3.250675e-002,10:1.609744e-001):8.169908e-002,8:5.418254e-002):4.737834e-003,9:1.603402e-003):3.706570e-001):1.448935e-001,(40:5.395953e-001,(5:5.044058e-002,4:3.101022e-002):8.194677e-003):1.845829e-001):2.015948e-001,(17:4.904102e-001,((16:1.452403e-001,6:7.433887e-002):1.769445e-001,19:1.800897e-001):2.819576e-002):6.845710e-002,1:4.988974e-001);

tree gen.202000 = [&U] (((((((41:1.546977e+000,42:4.429960e-001):3.724135e-001,(27:4.531860e-001,(((((36:1.797394e-001,(((24:6.742345e-002,25:7.083919e-003):5.398976e-002,26:2.274182e-001):2.810506e-002,(20:2.517410e-002,21:3.980294e-002):2.407961e-002):1.460963e-001):2.583525e-001,35:1.083865e-001):1.256573e-001,30:1.902035e-002):3.658611e-002,((((38:3.068665e-001,39:2.274084e-001):4.366833e-002,2:8.144073e-002):4.823669e-003,(23:1.276095e-002,(((34:7.357983e-002,((33:1.656154e-001,28:2.353641e-001):2.369690e-002,29:2.645586e-002):1.931455e-001):2.903076e-002,32:3.182679e-001):8.964698e-002,(37:6.997297e-002,7:3.387476e-001):3.622171e-002):4.724548e-002):2.526196e-001):2.908964e-001,31:1.889245e-001):1.378256e-002):6.892786e-003,22:8.534988e-002):1.952489e-002):3.090428e-001):1.533901e-001,18:2.058273e-001):8.837096e-002,((8:5.859340e-002,(((14:4.598643e-002,(3:4.598361e-002,15:7.785850e-002):4.340997e-001):9.267098e-002,10:1.132755e-001):6.212994e-002,(13:3.707846e-002,(11:9.557140e-002,12:3.504511e-002):2.846334e-001):3.099598e-002):1.864232e-001):5.314369e-002,9:3.374934e-002):1.335084e-001):3.307926e-001,((40:1.977465e-001,4:3.213031e-002):7.761288e-002,5:3.840440e-003):1.137383e-001):3.446071e-001,(19:1.400491e-001,(6:1.404522e-001,16:1.557198e-001):2.019452e-001):3.084285e-002):2.133404e-002,17:7.920565e-002,1:1.481305e-001);

tree gen.203000 = [&U] (((((((41:1.471142e+000,42:3.747266e-001):1.084652e+000,(22:8.687253e-002,((30:1.908700e-001,((2:5.543927e-002,(39:1.787972e-001,38:1.825286e-001):5.993940e-002):8.740522e-003,(23:8.302545e-002,(((32:4.136783e-001,34:3.081752e-001):3.103365e-002,(28:2.312382e-001,(33:2.872400e-001,29:2.425981e-002):4.877496e-002):7.899923e-002):3.187443e-001,(37:1.039727e-001,7:2.971381e-001):2.013810e-001):6.207602e-002):3.443225e-002):1.847900e-001):1.374040e-001,(27:2.514097e-001,(31:2.543633e-001,((36:2.336217e-001,((((24:8.901431e-002,25:7.705980e-002):1.586908e-001,26:3.401745e-001):9.844459e-003,21:4.913213e-002):4.951411e-003,20:7.911163e-002):2.519782e-001):2.825092e-001,35:1.408786e-001):1.577695e-001):2.944237e-002):8.584535e-002):2.911700e-002):4.465314e-001):6.089373e-001,18:1.249306e-001):1.437530e-002,((((14:9.974472e-002,(3:1.456448e-002,15:2.260665e-002):7.807896e-002):2.423510e-001,(13:2.348747e-002,(11:3.358184e-001,12:2.518913e-002):3.211704e-001):1.003243e-001):1.725033e-001,(8:2.352202e-002,10:3.636919e-002):8.119874e-002):8.951291e-002,9:6.806835e-002):2.382361e-001):1.899530e-001,((4:4.473877e-002,40:4.413215e-001):3.022754e-001,5:5.909725e-003):3.311733e-001):5.612663e-001,17:9.124589e-001):2.958733e-001,((19:1.297956e-001,16:9.374257e-002):1.948077e-002,6:1.821306e-001):1.916177e-001,1:2.993045e-001);

tree gen.204000 = [&U] (17:1.624373e-001,((((((41:8.784909e-001,42:2.641890e-001):3.820530e-001,((((36:7.923120e-002,((21:1.451215e-002,(24:1.385405e-001,25:1.443887e-002):6.318403e-002):1.981266e-002,(26:2.739114e-001,20:3.462067e-002):5.206189e-002):1.930612e-001):1.987511e-002,35:4.341702e-001):8.437916e-002,((((((39:9.563309e-002,38:4.976728e-002):1.069778e-001,2:4.310347e-002):2.406407e-002,(23:6.317911e-004,(((((29:1.818739e-002,33:1.620015e-001):6.772789e-002,28:1.290642e-001):1.809628e-001,32:3.288542e-001):1.831517e-002,34:2.729054e-001):2.603066e-001,(37:1.588433e-002,7:2.387552e-001):1.451440e-001):6.768489e-002):8.303606e-002):8.556312e-002,30:1.001404e-001):1.043625e-001,27:1.339432e-001):1.125920e-001,31:1.793726e-001):7.756230e-003):2.763708e-002,22:6.242934e-002):5.132684e-001):2.548516e-001,18:6.479249e-002):2.420016e-001,((8:1.596740e-001,((3:2.926360e-002,(14:3.387179e-003,15:1.176308e-002):8.154126e-002):1.030007e-001,(10:1.128329e-001,(13:4.651726e-002,(11:1.621294e-001,12:1.410353e-001):2.776321e-001):1.288779e-001):2.329006e-002):1.066434e-001):1.837910e-001,9:4.209412e-002):1.742708e-001):1.082831e-001,(40:3.111396e-001,(4:4.461284e-001,5:1.768110e-001):9.026889e-003):2.632993e-001):2.116970e-001,((16:5.893446e-002,6:7.473144e-002):9.695937e-003,19:2.330735e-001):1.329976e-001):1.562497e-002,1:3.624059e-001);

tree gen.205000 = [&U] (((((((41:1.270821e+000,42:3.973782e-001):8.232646e-001,(27:1.901410e-001,(((((36:7.829213e-002,((24:2.381174e-001,25:3.861996e-003):3.504666e-001,((21:4.192756e-002,20:1.171565e-001):9.486422e-003,26:1.443983e-001):1.428673e-002):8.717208e-002):5.614754e-002,35:6.988826e-001):1.166357e-001,((2:4.054035e-001,(39:1.438459e-001,38:1.183329e-001):1.386241e-001):2.920329e-001,(23:8.480955e-003,(((32:7.983985e-001,34:5.772263e-001):3.108376e-002,(29:3.898420e-001,(28:9.789583e-002,33:2.038061e-001):1.229588e-001):4.000492e-001):1.076758e-001,(37:5.653323e-003,7:3.591221e-001):6.637814e-002):1.277002e-001):1.271696e-001):8.606800e-002):1.082516e-001,31:3.254633e-001):3.919532e-002,(22:2.000342e-001,30:1.494409e-001):1.494563e-003):2.039569e-002):7.926319e-001):9.522218e-002,18:1.937293e-001):7.802472e-002,(((8:9.175472e-002,(((14:1.535376e-002,3:9.353598e-002):3.438044e-002,15:2.166802e-001):4.152531e-001,(13:1.128746e-001,(11:2.438658e-001,12:1.018436e-001):4.175985e-001):2.453108e-002):1.051156e-001):3.905498e-002,10:1.633408e-001):1.588324e-001,9:2.886693e-002):2.564682e-001):1.628733e-001,(40:3.160742e-001,(4:7.981023e-002,5:3.486320e-001):1.316815e-001):1.811832e-001):1.047983e+000,((19:1.238712e-001,6:3.343572e-001):5.490873e-002,16:1.323589e-001):2.692663e-003):5.123695e-002,17:3.863411e-001,1:1.625381e-001);

tree gen.206000 = [&U] (((16:7.654195e-002,6:1.809553e-001):1.700282e-001,(19:1.426336e-002,17:3.093082e-001):3.195219e-002):6.022133e-003,(((((41:9.450807e-001,42:1.314438e-001):5.727399e-001,((((2:1.862047e-001,(39:6.809937e-002,38:2.255743e-001):2.166693e-001):6.287901e-003,(23:1.125138e-001,(((34:3.092805e-001,32:5.937510e-001):2.606305e-003,(29:1.456076e-001,(28:3.179635e-001,33:1.157316e-001):7.475962e-002):2.538652e-001):2.169745e-001,(37:7.363276e-002,7:1.728828e-001):9.672189e-002):8.966747e-002):7.756137e-002):2.623200e-001,22:2.072143e-001):9.921512e-003,((30:5.587604e-002,(27:1.742165e-001,((36:1.267477e-001,(((20:5.339550e-002,21:6.526474e-002):9.714396e-002,(24:5.978231e-002,25:1.940301e-001):1.696859e-001):2.249280e-002,26:1.118958e-001):5.436548e-002):1.457294e-001,35:3.310666e-001):5.156994e-002):3.429824e-002):1.738770e-002,31:2.121109e-001):2.988902e-002):5.775033e-001):1.435879e-001,18:5.127261e-002):1.426376e-001,((8:5.890101e-002,(((14:6.775264e-003,15:6.896932e-002):1.465618e-001,3:1.042246e-001):3.702809e-001,((13:2.473419e-003,(11:8.386737e-002,12:3.091912e-002):1.996334e-001):8.443206e-002,10:9.395841e-002):4.325751e-002):1.133446e-002):1.122822e-001,9:1.324965e-001):1.859677e-001):1.262785e-001,(4:1.549810e-001,(40:2.540634e-001,5:4.719865e-003):8.089407e-002):2.441392e-001):7.532340e-001,1:1.610451e-001);

tree gen.207000 = [&U] (19:6.086028e-002,((((((41:8.734887e-001,42:3.081260e-001):5.293535e-001,((27:4.018658e-001,((((23:5.686883e-002,((32:3.433233e-001,((28:1.527089e-001,(33:1.012914e-001,29:1.145075e-001):1.009475e-003):1.022907e-001,34:2.357570e-001):1.840501e-001):2.072228e-001,(37:2.701813e-002,7:3.134561e-001):4.149305e-002):8.520363e-002):1.095510e-001,2:1.491542e-001):2.070176e-002,(39:1.701139e-001,38:2.084865e-001):1.122674e-001):2.702262e-001,((31:1.675666e-001,30:5.939553e-002):1.037003e-001,22:2.209687e-002):5.547103e-005):1.138804e-002):1.496841e-002,((36:1.199197e-001,((24:6.967559e-002,25:7.328264e-002):1.856698e-001,(21:4.211994e-003,(20:9.186696e-002,26:2.926988e-001):6.034125e-002):2.223270e-003):7.279377e-002):1.482647e-001,35:3.059875e-001):3.496559e-002):2.989740e-001):1.316375e-001,18:8.850124e-002):1.478932e-001,((((8:4.790501e-002,((3:2.622391e-002,15:1.193369e-001):2.140167e-001,14:5.915840e-003):1.522940e-001):1.663401e-001,(13:2.446039e-002,(11:3.332427e-002,12:1.443652e-002):1.845108e-001):1.403079e-002):1.662410e-001,10:8.509833e-002):2.559149e-002,9:6.574073e-002):1.581470e-001):2.578600e-001,((5:7.662743e-002,4:5.206768e-002):3.521687e-003,40:4.947950e-001):4.714787e-002):5.246349e-001,(17:2.886618e-001,(16:7.982757e-002,6:1.757165e-001):1.055701e-001):1.407851e-001):3.052115e-003,1:3.747441e-001);

tree gen.208000 = [&U] ((((6:1.511893e-001,16:1.066847e-002):1.598032e-001,(((((41:1.190575e+000,42:3.640744e-001):5.640133e-001,(31:1.461938e-001,((30:1.084726e-001,22:3.220817e-002):8.959392e-002,((((23:2.146800e-002,(((28:4.685125e-002,(29:1.995964e-003,33:1.372941e-001):1.217836e-001):2.508158e-001,(32:4.868651e-001,34:1.348134e-001):6.365521e-002):3.766186e-002,(37:9.615991e-002,7:4.483285e-001):1.394927e-001):1.138855e-001):1.451504e-001,(39:1.833479e-001,38:3.061507e-001):3.256141e-002):7.228161e-003,2:3.557544e-002):3.292662e-001,(27:4.324097e-001,((36:1.507660e-001,(26:2.386718e-002,(((24:9.221145e-002,25:2.362135e-002):9.090633e-002,20:1.348604e-001):5.817169e-002,21:1.298173e-001):2.149239e-002):5.142712e-002):2.653210e-001,35:3.199324e-001):1.138237e-001):4.231799e-002):1.329433e-002):2.970557e-003):4.525150e-001):2.555818e-001,18:1.213298e-001):1.489199e-001,((10:4.167121e-001,((((3:1.165337e-001,15:3.746353e-002):9.161717e-002,14:8.286700e-003):1.712625e-001,(13:1.095986e-001,(11:5.126855e-002,12:1.085946e-002):2.532744e-001):8.374156e-003):1.349193e-001,8:1.595594e-002):4.852119e-002):1.883737e-001,9:1.928322e-002):3.602405e-001):3.688107e-001,((40:6.276361e-001,5:2.021815e-002):3.345133e-002,4:8.029423e-002):1.426501e-001):7.335386e-001):2.232230e-001,17:2.606435e-001):1.250069e-002,19:1.498720e-002,1:4.456777e-001);

tree gen.209000 = [&U] (((19:2.907274e-002,17:1.839575e-001):1.755130e-001,(16:9.462986e-002,6:2.226837e-001):6.331165e-002):7.873767e-003,(((((41:1.043355e+000,42:3.359911e-001):6.911592e-001,(22:2.741285e-002,((((((36:1.664458e-001,(((24:3.598327e-002,25:1.975730e-002):7.357220e-002,(26:2.476278e-002,20:4.663913e-002):8.409027e-002):3.218294e-002,21:7.356981e-002):6.062895e-002):1.965304e-001,35:2.967878e-001):3.620237e-002,((39:1.167245e-001,38:2.516162e-001):2.896397e-001,((23:2.865173e-002,(((32:4.120704e-001,34:1.988243e-001):2.139152e-002,((29:1.197203e-001,33:1.924572e-001):5.183410e-002,28:2.358272e-001):1.109418e-001):2.845759e-002,(37:1.298526e-001,7:3.799108e-001):3.917105e-001):1.751597e-001):6.699223e-002,2:2.027792e-002):1.814483e-002):2.828115e-001):5.398194e-002,31:4.120657e-001):7.860775e-003,27:1.436233e-001):3.795562e-002,30:2.632218e-002):1.056635e-002):4.194304e-001):2.793787e-001,18:2.595813e-001):1.449485e-001,((8:4.438489e-002,((13:8.720094e-002,(11:5.898257e-002,12:4.277037e-002):3.649485e-001):1.345638e-001,((3:2.422878e-001,(15:2.956429e-002,14:6.927230e-002):1.375957e-001):2.812413e-001,10:9.593896e-002):2.314245e-001):7.022452e-002):3.201157e-002,9:9.498224e-002):3.313513e-001):4.035327e-001,((40:5.143788e-001,4:2.186134e-002):4.653564e-002,5:7.923385e-004):1.560040e-001):5.770800e-001,1:4.121927e-001);

tree gen.210000 = [&U] ((17:1.984392e-001,(((((41:7.306475e-001,42:2.352901e-001):3.610837e-001,((31:1.306731e-001,(((((23:8.135943e-004,(((34:2.055933e-001,32:2.819527e-001):5.277668e-003,(33:5.846874e-002,(29:2.234071e-001,28:6.352415e-002):2.062623e-002):7.635653e-002):8.806336e-002,(37:1.063035e-002,7:2.660465e-001):6.572316e-002):2.914845e-001):2.100255e-001,(39:1.588726e-001,38:3.316179e-001):1.269159e-001):5.701366e-002,2:9.579977e-002):1.193545e-001,22:3.665925e-002):3.395518e-002,27:1.068829e-001):2.126188e-002):5.521858e-002,(((36:1.927876e-001,((26:4.047641e-002,(20:1.561719e-003,(24:1.610254e-002,25:6.768840e-002):9.072842e-002):8.268175e-002):2.436767e-002,21:2.602298e-001):2.341719e-001):2.843014e-001,35:2.078366e-001):8.075270e-002,30:3.630214e-002):1.848950e-002):3.706463e-001):2.204683e-001,18:1.932300e-001):1.670476e-001,((((13:5.283047e-003,(11:1.689831e-002,12:1.555410e-003):3.457787e-001):9.988414e-002,((15:1.546886e-002,14:3.389925e-002):8.988006e-002,3:1.312841e-001):8.499775e-002):3.322835e-001,(8:2.912588e-002,10:6.718477e-002):4.122970e-002):3.411983e-002,9:1.270674e-002):2.320409e-001):2.890527e-001,((4:5.302723e-002,40:3.364905e-001):3.058043e-002,5:1.414742e-002):3.813277e-001):2.554191e-001):8.416945e-002,((6:6.443710e-002,16:3.260237e-001):7.547080e-002,19:1.205302e-001):5.707989e-002,1:2.563459e-001);

tree gen.211000 = [&U] ((((((41:1.002266e+000,42:5.267711e-001):5.104659e-001,((31:1.663962e-001,((22:1.528068e-001,(27:6.380647e-001,((39:4.881676e-002,38:3.052012e-001):1.936393e-001,(2:2.441762e-002,(23:5.808312e-003,(((((29:2.367632e-001,33:1.446581e-001):3.315773e-002,28:1.230596e-001):1.677933e-001,34:2.089557e-001):1.136498e-003,32:3.930755e-001):1.502566e-001,(37:4.652590e-002,7:3.370400e-001):3.032749e-002):8.174089e-002):1.418401e-001):1.547406e-002):1.808745e-001):3.398139e-002):3.779734e-002,30:4.130293e-002):6.807141e-002):1.752201e-002,((36:3.874402e-001,(26:1.153901e-001,(((24:2.068469e-002,25:5.973479e-002):7.245050e-002,21:8.033301e-002):1.157567e-002,20:1.768905e-002):1.777592e-002):1.417826e-001):1.846393e-001,35:1.970752e-001):1.486267e-001):5.082727e-001):2.334391e-001,18:3.854654e-001):3.464357e-002,(((((15:3.308189e-001,14:4.251235e-002):6.826440e-002,3:2.961040e-001):2.364918e-001,(13:2.261099e-002,(11:2.151793e-002,12:4.736658e-004):4.403067e-001):1.326196e-001):2.285427e-001,(10:8.730750e-002,8:2.984481e-002):1.013465e-001):1.975966e-001,9:1.757024e-001):2.469121e-001):3.267739e-001,((40:3.752274e-001,5:1.865065e-002):7.727311e-002,4:1.965753e-002):1.950163e-001):3.258652e-001,(17:4.923079e-001,(16:2.516172e-001,(19:6.699900e-002,6:4.054781e-001):6.185321e-002):2.549041e-001):7.421649e-002,1:3.512083e-001);

tree gen.212000 = [&U] ((((16:2.303167e-001,19:9.490983e-002):3.870199e-002,6:4.381700e-002):4.274192e-002,(((((41:1.525643e+000,42:4.320277e-001):5.586785e-001,(((31:1.378104e-001,(27:5.565888e-001,22:8.132473e-002):1.125274e-001):8.073216e-002,(30:7.326385e-002,((36:1.597948e-001,(((20:7.536860e-003,26:1.490700e-001):1.382027e-003,21:1.659778e-002):8.975120e-003,(24:8.947312e-003,25:4.899108e-002):2.340128e-001):8.379724e-002):2.953239e-001,35:1.616299e-001):1.470448e-001):3.087795e-002):3.838275e-002,((39:2.685190e-002,38:2.503087e-001):1.023023e-001,((23:8.618859e-002,((((29:7.462343e-002,(28:9.898452e-002,33:1.164822e-001):2.565038e-002):7.548380e-002,34:1.512024e-001):2.406926e-002,32:6.844651e-001):2.381879e-001,(37:2.188404e-002,7:3.902509e-001):2.679041e-001):2.215014e-002):3.073123e-001,2:2.032072e-002):1.563224e-002):3.916517e-001):8.107457e-001):3.390983e-001,18:2.759351e-001):2.001374e-001,(((10:5.830305e-002,(((15:1.893287e-001,3:2.215069e-002):2.686534e-001,14:1.926488e-002):2.630755e-001,(13:2.164803e-002,(11:3.523188e-002,12:1.486267e-002):3.381354e-001):5.963143e-002):4.095763e-002):3.627607e-002,8:2.866903e-002):2.603725e-002,9:8.975904e-003):2.025033e-001):4.304129e-001,(5:8.655729e-002,(4:1.795547e-001,40:8.329878e-001):2.206737e-002):9.480781e-002):4.460753e-001):4.782574e-002,17:3.964671e-001,1:3.430937e-001);

tree gen.213000 = [&U] (((((((41:1.370130e+000,42:3.879900e-001):6.854483e-001,((31:1.733306e-001,(((((36:1.435065e-001,(((21:9.676458e-003,26:1.354446e-001):3.750543e-003,20:6.682069e-003):4.150508e-003,(24:1.480620e-002,25:1.016492e-001):6.733548e-002):1.445738e-001):1.585998e-001,35:1.992495e-001):1.921132e-001,30:2.951623e-001):4.060672e-002,((39:5.694806e-002,38:4.181203e-001):1.199111e-001,(((((32:8.139512e-001,((33:1.046089e-001,28:9.069732e-002):5.978093e-002,29:7.819196e-002):2.977513e-001):3.653846e-002,34:1.578959e-001):1.765409e-001,(37:6.514971e-002,7:4.001994e-001):8.301683e-002):2.981553e-002,23:2.513999e-003):9.102878e-002,2:4.985434e-002):9.332176e-002):2.720973e-001):9.182192e-002,22:7.245690e-002):8.725913e-003):3.026791e-002,27:4.691372e-001):7.535205e-001):8.334699e-002,18:3.388943e-001):1.010614e-002,((8:1.729521e-002,(((15:1.700299e-001,3:2.288890e-002):4.865543e-001,14:2.377820e-002):4.448928e-002,((13:1.575384e-002,(11:2.474364e-002,12:1.035303e-001):3.036683e-001):2.322284e-001,10:1.242206e-001):6.132444e-002):9.873459e-003):7.012404e-002,9:1.003123e-001):6.697711e-002):1.558210e-001,(5:4.592911e-003,(4:7.247977e-002,40:5.566374e-001):1.044970e-001):1.241760e-001):2.838543e-001,17:1.595900e-001):5.897473e-003,((6:3.882451e-002,16:5.316510e-002):6.211298e-003,19:2.406138e-001):1.438134e-001,1:3.388170e-001);

tree gen.214000 = [&U] ((((((41:1.687452e+000,42:2.196669e-001):1.045235e+000,(((((30:4.456117e-002,((36:1.604600e-001,((21:3.519256e-002,(26:1.543192e-001,20:1.169297e-001):7.461932e-002):2.606831e-002,(24:1.294804e-002,25:9.778863e-002):2.590231e-001):2.204625e-001):3.182269e-001,35:2.680772e-001):1.883939e-001):2.995728e-002,27:5.222788e-001):8.668020e-002,(2:8.308217e-002,((39:1.944570e-001,38:5.500289e-001):1.688135e-001,(((((33:1.718504e-001,28:4.047111e-001):5.250410e-002,29:8.088967e-002):2.201825e-001,(32:9.463754e-001,34:1.949152e-001):4.934647e-002):1.546943e-001,(37:7.284635e-002,7:4.474780e-001):2.822548e-001):1.257815e-001,23:4.835482e-003):1.090238e-001):4.294393e-002):2.617834e-001):1.426646e-002,22:7.940250e-002):4.131973e-002,31:1.911199e-001):8.020190e-001):2.330588e-001,18:2.009848e-001):4.199050e-002,(((13:8.137018e-003,(11:5.637709e-002,12:2.692670e-002):3.338255e-001):1.428310e-002,((15:7.177002e-002,(3:5.754600e-002,14:4.605134e-003):4.612332e-002):4.822297e-001,(8:5.322708e-002,10:8.302042e-002):5.240913e-002):1.090628e-003):5.923295e-002,9:1.975701e-001):2.454534e-001):2.466160e-001,((40:6.141387e-001,5:2.663873e-002):3.188679e-002,4:8.326679e-002):2.384091e-001):2.420545e-001,((16:2.065387e-001,(6:1.612850e-001,19:2.618186e-001):1.478682e-001):1.899920e-001,17:5.632712e-001):6.036273e-003,1:1.614354e-001);

tree gen.215000 = [&U] (19:1.258716e-001,((((((41:1.327033e+000,42:1.727487e-001):4.418618e-001,(((((((((33:1.286466e-001,28:3.139056e-001):1.477955e-002,29:8.928221e-002):1.770713e-001,32:7.517664e-001):8.056303e-002,34:1.611457e-001):2.339759e-001,(37:1.165687e-001,7:3.519020e-001):3.223171e-001):3.350219e-001,23:3.036134e-002):4.505479e-002,2:2.271308e-001):8.757084e-002,(39:3.308461e-001,38:4.027715e-001):5.032786e-002):1.549809e-001,(((36:2.432795e-001,((26:1.309202e-001,(21:1.879038e-002,20:3.265132e-002):3.077949e-001):2.861006e-002,(24:1.724469e-004,25:6.962652e-002):3.883918e-002):1.627152e-001):1.367167e-001,35:2.108191e-001):8.697650e-002,(30:8.585031e-002,((27:4.268934e-001,22:5.986917e-002):1.308319e-002,31:1.466312e-001):3.381501e-002):1.206918e-001):7.567839e-002):6.519349e-001):1.832802e-001,18:2.248009e-001):8.240023e-002,((8:1.194020e-002,((13:3.924100e-002,(11:4.433561e-002,12:2.284169e-003):2.625244e-001):5.405676e-002,(((15:3.970691e-002,3:2.238519e-004):2.004328e-001,14:1.471046e-003):2.030757e-001,10:1.932588e-001):2.624122e-001):2.665993e-001):1.176559e-001,9:1.825918e-001):2.718998e-001):6.261610e-001,(4:1.371047e-001,(5:7.154262e-002,40:4.912998e-001):2.900771e-002):1.423019e-001):1.919859e-001,((6:1.230432e-001,16:9.206182e-002):1.104080e-001,17:2.787549e-001):5.120849e-003):1.501100e-002,1:2.313931e-001);

tree gen.216000 = [&U] (((((((41:1.248438e+000,42:2.931743e-001):5.077399e-001,(27:4.697206e-001,((30:7.533778e-002,((36:2.795505e-001,((20:3.751937e-002,(24:1.718626e-001,25:7.849380e-002):5.136828e-002):1.406085e-001,(26:2.263147e-001,21:3.135979e-002):4.883668e-002):2.431776e-001):8.329576e-002,35:2.226951e-001):2.082978e-001):7.474819e-002,((22:1.132072e-001,(2:3.675075e-002,((39:3.960784e-001,38:2.284500e-001):1.118516e-001,((((32:2.122499e-001,((28:3.907473e-001,33:8.191775e-002):1.888152e-002,29:1.025935e-001):2.833412e-001):8.117946e-002,34:1.851712e-001):4.166230e-002,(37:1.443428e-001,7:5.237908e-001):1.335709e-001):1.191914e-001,23:2.168684e-001):8.786435e-002):8.583489e-003):2.065878e-001):1.262032e-001,31:3.838735e-001):5.850012e-003):1.393612e-001):7.569779e-001):1.487977e-001,18:1.346965e-001):1.428294e-001,((10:3.210390e-001,(((13:3.083204e-002,(11:5.094570e-002,12:3.704602e-002):3.129873e-001):1.494308e-001,(15:4.963865e-002,(14:7.583101e-002,3:4.534645e-002):3.867957e-002):1.997470e-001):2.743536e-001,8:2.622105e-002):2.746147e-001):3.468769e-002,9:9.669342e-002):3.662080e-001):2.764621e-001,(4:7.850249e-002,(40:5.411823e-001,5:1.556773e-001):4.813268e-002):1.164547e-001):9.496343e-001,((19:2.979900e-001,16:8.781048e-002):2.300821e-002,6:1.510677e-001):8.298080e-002):5.717617e-002,17:3.229394e-001,1:5.253665e-001);

tree gen.217000 = [&U] (((16:2.338485e-001,6:7.253717e-002):1.658758e-001,(19:9.598874e-002,17:3.429130e-001):2.012894e-002):1.082581e-001,(((((41:1.396735e+000,42:2.248735e-001):4.737374e-001,((((31:2.621687e-001,(30:1.696945e-001,22:3.298012e-002):8.079831e-002):1.437533e-002,((36:1.250833e-001,(21:7.148123e-002,(((24:7.491482e-002,25:5.121648e-002):1.901812e-001,26:2.365899e-001):3.329196e-004,20:3.840869e-002):1.133121e-002):2.153010e-001):1.871032e-001,35:2.392790e-001):7.404576e-002):2.853461e-001,27:2.618450e-001):2.574171e-001,(((((32:2.556727e-001,34:1.898982e-001):8.678042e-002,(33:1.563062e-001,(28:3.998418e-002,29:3.653213e-001):4.081840e-001):1.793790e-001):6.041685e-002,(37:5.252092e-002,7:5.627971e-001):1.383645e-001):1.201789e-001,23:2.603057e-002):2.420705e-002,((39:1.603425e-001,38:8.256566e-001):6.673433e-002,2:4.704880e-002):4.398229e-002):2.123110e-001):9.945164e-001):1.598786e-001,18:2.192818e-001):1.394082e-001,(((10:1.038424e-001,8:5.311840e-002):8.073385e-004,(((14:3.866649e-002,15:8.625590e-003):1.888860e-001,3:2.008530e-002):5.533480e-001,(13:2.701418e-002,(11:1.723062e-001,12:3.603674e-002):2.648574e-001):2.602602e-001):5.786899e-002):1.126393e-001,9:3.305833e-002):2.850998e-001):3.099870e-001,(5:1.672705e-001,(4:1.681307e-001,40:7.186513e-001):7.912242e-002):2.083434e-001):4.373326e-001,1:4.735775e-001);

tree gen.218000 = [&U] ((17:2.594936e-001,(((((41:1.006893e+000,42:1.621092e-001):4.544663e-001,(31:6.268141e-002,(((22:2.062750e-001,(((((34:2.487446e-001,(32:1.881280e-001,((28:1.287584e-001,29:9.449013e-002):1.093283e-001,33:1.360374e-001):3.190204e-001):1.552719e-002):1.532328e-001,(37:4.722209e-003,7:3.652795e-001):9.934088e-002):1.024676e-001,23:1.317019e-001):1.178699e-001,(39:7.691801e-002,(38:4.112864e-001,2:3.917707e-002):1.756790e-002):8.974094e-002):1.557962e-001,((36:1.071900e-001,(20:9.054523e-002,((24:3.214488e-002,25:5.567989e-002):1.074819e-001,(21:2.541602e-002,26:1.049074e-001):5.324985e-002):4.217507e-002):2.857442e-001):2.914395e-001,35:2.007630e-001):1.948594e-001):1.499801e-001):8.928846e-002,27:1.894517e-001):1.629342e-002,30:1.223312e-001):2.305070e-001):3.146329e-001):4.047116e-001,18:1.331968e-001):3.157334e-002,(((14:9.787131e-002,(15:2.556603e-002,3:7.566725e-004):3.611213e-001):1.379790e-001,((10:5.609994e-002,(11:5.650687e-002,12:1.813008e-002):1.879985e-001):2.223385e-002,(8:8.964140e-003,13:1.143281e-001):1.602707e-002):2.255255e-002):1.129716e-001,9:2.383144e-002):2.561715e-001):2.279263e-001,(40:5.150338e-001,(5:1.191058e-001,4:1.282154e-001):3.108379e-003):2.219803e-001):4.038806e-001):1.091169e-001,((16:2.538590e-001,6:3.243087e-002):1.410733e-002,19:3.396859e-002):2.014751e-002,1:2.220972e-001);

tree gen.219000 = [&U] (((19:1.278219e-001,(16:4.394152e-001,6:7.448245e-002):4.081449e-002):3.311565e-001,(((((41:1.071201e+000,42:3.930138e-001):4.834916e-001,((((((((33:1.573652e-001,28:1.311537e-001):8.617680e-003,29:1.074819e-001):9.003594e-002,(32:1.906427e-001,34:4.371864e-001):3.934435e-002):1.587370e-001,(37:9.998617e-004,7:5.771058e-001):2.622738e-001):1.912654e-002,23:5.376236e-002):5.027202e-002,(2:4.165931e-002,(39:1.260667e-001,38:3.528621e-001):1.121864e-001):3.242032e-002):1.567327e-001,(31:4.665265e-002,((27:2.070291e-001,22:7.543896e-002):1.836203e-001,((36:1.318818e-001,(26:2.249328e-002,((21:3.132273e-003,20:3.668812e-002):9.096851e-002,(24:1.838218e-002,25:1.125560e-001):5.701443e-003):1.204188e-001):1.850745e-001):2.363346e-001,35:2.135851e-001):2.122028e-001):8.911771e-002):1.920056e-002):2.001026e-002,30:3.141032e-001):3.536869e-001):9.079051e-002,18:2.047956e-001):1.750078e-001,((8:1.208932e-001,((14:1.604240e-001,(15:6.485070e-002,3:4.984580e-002):1.431861e-001):8.719353e-002,((13:5.670784e-002,10:4.066890e-001):1.726771e-001,(11:6.021137e-002,12:2.871882e-001):3.657800e-001):3.175905e-002):1.008377e-001):4.006454e-002,9:1.183117e-001):2.514745e-001):5.256648e-001,(40:5.227082e-001,(4:1.111542e-001,5:1.464444e-001):2.561156e-002):1.538012e-001):3.353325e-001):1.440659e-001,17:2.759833e-001,1:3.978039e-001);

tree gen.220000 = [&U] (((((41:1.570992e+000,42:8.369265e-002):5.390242e-001,(((36:1.372011e-001,((26:9.191025e-002,(20:1.033989e-002,21:2.502907e-002):8.512897e-002):1.251616e-001,(24:1.425137e-002,25:1.615336e-003):3.827087e-002):1.095858e-001):2.917916e-001,35:2.784097e-001):1.803853e-001,(((31:5.649046e-002,30:2.647134e-001):1.194790e-002,(22:1.957846e-001,(((((34:3.449184e-001,32:3.058594e-001):1.036399e-002,((29:2.279776e-002,33:1.918902e-001):3.653045e-002,28:2.162797e-001):1.504535e-001):3.524830e-002,(37:1.135624e-002,7:6.003828e-001):1.944442e-001):3.184619e-002,23:3.963445e-002):5.660873e-002,((39:1.907778e-001,38:6.280525e-001):9.779648e-002,2:2.245009e-002):6.977393e-002):2.282611e-001):6.023013e-002):3.484577e-002,27:2.001594e-001):1.460922e-002):5.716773e-001):6.095967e-001,(((10:1.917300e-001,((((15:1.435718e-003,3:7.119445e-002):3.078387e-002,14:2.948234e-002):5.321684e-001,8:1.596452e-001):4.429899e-003,(13:1.298495e-002,(11:8.606418e-002,12:4.643769e-003):4.092031e-001):4.203856e-002):4.089458e-002):1.322163e-001,9:2.788742e-002):2.848976e-001,18:1.125580e-001):2.231233e-002):2.821539e-001,(4:4.774956e-002,(5:1.768714e-002,40:6.131727e-001):7.164041e-002):2.822566e-001):3.382261e-001,((6:2.272374e-001,16:2.326324e-001):2.593725e-001,(19:1.912313e-001,17:4.235983e-001):1.092922e-001):7.502697e-002,1:5.290960e-001);

tree gen.221000 = [&U] ((((((((14:5.386934e-002,(3:2.434702e-002,15:4.510116e-002):4.844860e-002):1.825647e-001,10:2.932098e-001):2.529038e-001,8:4.067985e-002):6.363676e-002,(13:2.695412e-003,(11:7.817181e-002,12:7.665030e-002):2.598899e-001):5.136853e-002):9.706039e-002,9:6.603462e-002):1.577292e-001,(18:2.856365e-001,((41:1.300712e+000,42:3.912117e-001):8.574028e-001,((((22:1.617686e-001,((36:1.560218e-001,((20:8.003085e-002,26:2.720970e-001):4.331564e-002,(21:3.359670e-002,(24:9.072874e-004,25:1.679029e-003):5.099864e-002):5.758650e-002):9.040612e-002):8.899859e-002,35:1.629508e-001):6.728413e-002):6.039817e-002,(27:1.725708e-001,31:5.299790e-001):4.598669e-002):2.774601e-002,((((((32:2.565390e-001,((29:2.906165e-001,33:1.075727e-001):2.079760e-003,28:9.784147e-002):2.318580e-002):8.323844e-002,34:1.348327e-001):9.558338e-002,(37:9.525021e-003,7:2.461527e-001):2.873151e-001):1.128516e-001,23:1.644096e-002):8.592461e-002,2:1.882997e-002):2.003889e-002,(39:2.600030e-001,38:3.008339e-001):7.559367e-002):1.750218e-001):1.669870e-001,30:7.270981e-002):5.858007e-001):1.669924e-001):1.006463e-001):2.404900e-001,((4:2.002793e-002,5:8.705001e-002):9.667436e-002,40:5.076030e-001):1.748009e-001):2.985603e-001,(17:2.212587e-001,(6:6.753485e-002,(16:9.064617e-002,19:2.458356e-001):2.011112e-002):6.573542e-002):2.177817e-002,1:2.996722e-001);

tree gen.222000 = [&U] ((((((10:7.441573e-002,((8:3.995660e-002,(13:1.728897e-003,(11:4.657084e-002,12:5.006109e-002):4.207206e-001):3.313077e-002):6.509845e-002,((15:1.415833e-003,3:6.588578e-002):1.434652e-002,14:5.585563e-002):4.509032e-001):8.068268e-002):1.339234e-001,9:7.112202e-002):2.739125e-001,(18:1.838773e-001,((41:1.386131e+000,42:4.691484e-001):7.693404e-001,(31:1.338740e-001,(22:3.517976e-002,(((((((((34:1.976525e-001,32:2.439541e-001):2.977538e-002,((29:6.741538e-002,33:2.550999e-001):7.050870e-002,28:1.185891e-001):7.061920e-002):8.784114e-002,(37:9.355674e-003,7:2.614869e-001):1.893396e-001):3.351640e-002,23:1.344393e-001):9.435454e-002,2:1.931487e-002):2.201188e-002,(39:2.678452e-001,38:1.871658e-001):1.281550e-001):3.274377e-001,27:3.105417e-001):4.126368e-003,30:1.131274e-002):1.668867e-002,((36:6.935423e-002,(26:7.469171e-002,((20:9.250284e-002,21:1.728879e-002):6.190215e-002,(24:7.737618e-005,25:5.939141e-002):1.796329e-002):2.820169e-005):4.924611e-001):1.232832e-001,35:1.224489e-001):1.060398e-001):4.613200e-002):8.396825e-002):4.901880e-001):9.778185e-002):8.537266e-002):4.249203e-001,((40:4.768759e-001,4:1.094126e-001):1.072023e-002,5:5.100794e-002):1.735542e-001):4.067669e-001,(6:1.742673e-001,16:2.166793e-001):1.888145e-001):2.978598e-002,(19:1.149585e-001,17:1.243136e-001):3.143847e-001,1:1.927426e-001);

tree gen.223000 = [&U] (((((10:2.860141e-001,(((13:6.757117e-002,(11:2.523511e-001,12:2.329446e-003):1.010164e+000):1.371186e-001,(15:1.032086e-002,(3:6.608674e-002,14:5.576276e-002):3.886650e-002):3.451502e-001):5.760892e-002,8:1.580924e-002):1.044643e-001):1.251490e-001,9:2.938225e-002):3.489143e-001,(18:1.314875e-001,((41:9.482633e-001,42:7.949725e-002):6.460574e-001,((2:6.052365e-002,(((((32:2.435485e-001,(29:1.058175e-001,(28:2.557543e-001,33:4.504869e-001):5.481423e-003):4.284169e-001):2.576221e-002,34:2.371769e-001):1.236090e-001,(37:1.227668e-001,7:3.597956e-001):1.168745e-001):3.900233e-002,23:5.396682e-002):9.773569e-002,(39:1.441251e-001,38:2.439963e-001):2.457581e-001):4.482810e-002):6.064574e-002,(((36:9.086770e-002,(26:2.242356e-001,(((24:3.078878e-005,25:5.929266e-002):4.553218e-001,20:8.489610e-002):5.804436e-003,21:3.599278e-002):3.966144e-002):2.170633e-001):2.812766e-001,35:3.247323e-001):6.618496e-002,((30:2.853192e-001,(22:1.171361e-001,27:3.119074e-001):4.616194e-002):5.227671e-003,31:3.914813e-001):3.048173e-001):1.819856e-002):6.578654e-001):3.425177e-001):3.537719e-001):1.368132e-001,(4:1.234069e-001,(5:5.467979e-002,40:4.760830e-001):9.424085e-003):1.221494e-001):4.060906e-001,((19:1.138207e-001,(6:7.471849e-002,16:2.674405e-001):4.162224e-002):1.208203e-001,17:5.323885e-001):4.495464e-003,1:2.046039e-001);

tree gen.224000 = [&U] (19:1.229222e-001,(((6:3.996696e-002,16:3.557770e-002):3.595707e-002,17:3.219742e-001):1.500133e-001,(((((13:4.805314e-002,(11:9.420619e-002,12:1.845435e-003):1.127358e-001):2.126857e-001,((((15:4.219424e-003,3:7.892037e-002):2.402441e-003,14:1.850982e-002):1.476796e-001,10:1.557618e-001):1.485231e-001,8:1.763622e-002):2.758430e-002):8.336577e-002,9:4.848759e-002):1.030359e-001,(18:5.097010e-002,((41:7.512338e-001,42:1.274525e-001):6.059291e-001,(31:4.948985e-001,(30:1.944432e-001,((((((34:1.970478e-001,(32:2.803484e-001,(28:1.910990e-001,(29:4.819022e-002,33:3.568850e-001):2.848198e-002):8.134604e-002):5.842883e-002):9.068527e-002,(37:6.581153e-002,7:1.971291e-001):1.525887e-001):1.389678e-001,23:4.275363e-002):1.660434e-002,(39:1.141789e-001,38:1.872230e-001):6.352036e-002):1.974941e-002,2:2.016108e-002):1.488193e-001,(22:9.049031e-002,(((36:1.237509e-001,(((26:2.621368e-001,20:4.102979e-002):1.392031e-001,21:6.767662e-002):1.731032e-002,(24:5.146662e-004,25:1.234724e-001):1.686259e-001):6.118858e-002):2.252737e-001,35:2.572597e-001):1.013212e-001,27:2.546743e-001):2.748320e-002):1.592110e-002):5.794667e-002):1.100245e-002):2.844477e-001):1.857710e-001):2.731763e-002):1.063978e-001,(4:3.040896e-002,(40:1.993277e-001,5:1.620345e-002):1.755163e-003):1.079597e-001):2.709323e-001):2.238344e-002,1:2.035443e-001);

tree gen.225000 = [&U] (((6:1.615149e-001,16:1.081652e-001):1.116338e-001,19:8.830703e-002):2.813023e-003,((((((10:2.155738e-001,((13:4.616080e-002,(11:1.590238e-001,12:8.865247e-002):8.671909e-002):8.764539e-002,8:1.869584e-001):2.808449e-002):3.787332e-002,(14:6.145529e-002,(15:2.466382e-002,3:8.196709e-002):5.165713e-002):1.401998e-001):2.320878e-002,9:3.213631e-002):5.993864e-002,(18:1.729473e-001,((41:1.011041e+000,42:1.420497e-001):3.858544e-001,((36:1.749497e-001,(((24:9.960142e-003,25:7.526575e-002):4.071283e-002,26:1.864844e-001):9.746069e-002,(20:9.122711e-003,21:3.128730e-002):5.871111e-003):7.006460e-002):4.951499e-002,((((22:1.920768e-001,31:3.824328e-001):2.079770e-002,((2:2.770172e-001,(39:6.949832e-002,38:1.168192e-001):1.710520e-001):9.768973e-002,(((34:1.921567e-001,((28:1.934819e-001,(29:5.304068e-002,33:3.347003e-002):1.422904e-002):7.274653e-002,32:3.034668e-001):2.223032e-002):4.693257e-002,(7:2.198389e-001,37:1.985672e-001):3.228794e-002):3.258722e-002,23:7.526155e-003):5.490124e-002):1.672493e-001):1.075253e-001,(30:1.873049e-001,27:2.370762e-001):1.161325e-001):3.214364e-002,35:4.073612e-001):3.214251e-002):7.515503e-001):9.842668e-002):1.052045e-002):1.942405e-001,((5:5.300265e-003,4:1.744071e-001):4.113987e-002,40:4.328362e-001):2.183222e-001):2.922154e-001,17:3.337085e-001):2.192194e-002,1:2.034022e-001);

tree gen.226000 = [&U] (19:1.794434e-001,(((16:6.331450e-002,6:1.257642e-001):6.146700e-002,17:2.727240e-001):1.371017e-001,((((((13:4.888198e-002,(11:1.572028e-001,12:8.366438e-002):2.561897e-001):8.661238e-003,(((15:2.240502e-002,3:8.417513e-002):6.929615e-003,14:1.766418e-002):1.107547e-001,10:2.902962e-001):5.080199e-002):9.565684e-002,8:3.004882e-002):1.709845e-001,9:8.818207e-003):7.911957e-002,(18:1.096738e-001,((41:9.994635e-001,42:1.904311e-001):2.139037e-001,(30:6.718429e-002,(35:4.057608e-001,((22:4.655379e-002,31:3.625812e-001):5.169587e-002,((27:1.183911e-001,(36:1.215174e-001,(21:3.620344e-003,((24:1.009426e-002,25:1.436268e-001):8.913661e-003,(20:1.810882e-002,26:1.172702e-001):5.508591e-003):5.728588e-002):2.120070e-001):1.535374e-001):1.354632e-002,(((39:1.717941e-001,38:1.033803e-001):1.825128e-001,(((7:2.470761e-001,37:1.802357e-001):2.410293e-002,((34:6.091301e-002,(33:4.281267e-002,(29:5.362540e-002,28:5.228991e-002):2.627228e-002):1.323104e-001):1.543357e-002,32:3.058295e-001):1.102845e-001):2.550954e-002,23:1.127196e-001):1.194706e-002):6.521746e-002,2:9.268107e-002):4.164807e-001):9.577068e-002):1.753568e-002):5.399624e-002):2.898048e-001):2.282593e-001):8.069505e-002):1.285384e-001,(4:2.448139e-001,(40:4.251670e-001,5:1.962984e-003):1.641040e-002):1.394567e-001):2.217019e-001):7.224401e-002,1:2.010730e-001);

tree gen.227000 = [&U] ((19:1.173125e-001,(((((8:2.191058e-002,(14:4.651529e-002,(15:1.491095e-001,3:9.923974e-002):2.069064e-001):5.149101e-001):1.491326e-002,(10:8.280273e-002,(13:1.214735e-001,(11:1.304377e-001,12:4.601213e-003):3.144380e-001):4.389061e-002):3.292199e-002):1.988537e-001,9:4.202216e-003):4.595211e-001,(18:7.698676e-002,((41:1.178335e+000,42:3.375486e-001):3.466694e-001,(31:2.820310e-001,(30:7.766568e-002,(27:4.429405e-001,((22:1.915512e-001,35:5.738814e-001):1.332642e-002,((36:4.042583e-001,(((24:6.643234e-002,25:8.517334e-002):3.122950e-002,21:8.298755e-004):1.283168e-001,(20:1.033970e-002,26:1.173691e-001):8.261957e-002):2.077047e-001):2.634438e-001,((((7:5.094335e-001,37:6.679525e-002):9.888753e-002,(32:4.799567e-001,(34:1.271635e-001,(28:2.969094e-001,(33:1.763894e-001,29:4.923986e-002):6.556766e-003):6.397673e-002):1.018155e-001):2.003672e-001):1.464858e-001,23:1.794267e-002):1.948041e-001,((39:2.708942e-001,38:1.454023e-001):1.620898e-001,2:8.702248e-002):6.237207e-002):4.000909e-001):3.100266e-002):6.146551e-002):1.076789e-001):1.386957e-002):3.696013e-001):7.336024e-002):5.711961e-002):1.769685e-001,((5:2.922973e-003,4:4.255840e-002):1.179564e-001,40:8.530854e-001):2.417241e-001):2.033025e-001):2.070233e-001,(17:4.038650e-001,(16:7.929723e-002,6:6.594557e-002):8.815028e-002):2.346801e-002,1:1.927216e-001);

tree gen.228000 = [&U] ((((((((11:1.134513e-001,12:3.985277e-003):2.816598e-001,13:3.617011e-002):4.833226e-002,(8:2.315482e-002,((14:1.249073e-001,(15:1.450081e-001,3:2.225159e-002):1.243836e-001):2.466714e-001,10:1.969662e-001):2.524607e-001):7.765886e-002):2.482991e-002,9:1.494397e-002):2.947755e-001,(18:1.463791e-001,((41:1.020598e+000,42:1.612340e-001):2.099219e-001,((22:7.297809e-002,(36:1.610795e-001,(20:2.900923e-002,((21:6.934461e-003,26:1.079223e-001):4.404723e-002,(24:8.748365e-003,25:7.377171e-002):3.517053e-002):9.210048e-002):7.645992e-002):2.310926e-001):3.367163e-002,(31:2.451997e-001,((30:1.396690e-001,((2:1.735163e-001,(39:1.313910e-001,38:2.251950e-001):3.355776e-002):3.156150e-002,(((7:5.412636e-001,37:1.512026e-002):1.794975e-001,(32:4.134335e-001,(((29:3.435303e-002,28:1.765128e-002):2.310994e-002,33:1.863536e-001):1.291947e-001,34:1.486281e-001):2.986431e-002):1.075020e-001):3.991285e-002,23:2.867310e-002):1.154980e-001):3.498320e-001):1.356122e-001,(35:4.220483e-001,27:3.965330e-001):2.227394e-002):4.139855e-002):8.131410e-002):2.205455e-001):2.086255e-001):6.731133e-002):4.019515e-001,(5:1.872866e-002,(4:2.581260e-002,40:7.213763e-001):1.138590e-001):1.184761e-001):2.205745e-001,(17:2.853469e-001,19:1.025396e-001):4.737465e-002):4.334211e-002,(16:2.484411e-001,6:7.217165e-002):8.330759e-002,1:1.944310e-001);

tree gen.229000 = [&U] ((((6:1.446225e-001,17:2.842532e-001):1.185505e-001,16:1.374952e-001):8.576586e-002,((((((10:2.119469e-001,(14:5.210222e-002,(15:7.053681e-002,3:7.171054e-002):1.243849e-001):5.094324e-001):1.708766e-001,((11:3.177627e-002,12:4.696898e-002):3.645772e-001,13:2.639736e-001):1.830270e-002):2.387873e-002,8:9.063608e-003):1.130185e-001,9:1.086749e-002):3.403259e-001,(18:1.867626e-002,((41:1.484446e+000,42:1.233582e-001):4.360632e-001,((35:5.462944e-001,((22:2.059072e-001,(2:2.145852e-001,((39:9.863709e-002,38:2.898737e-001):1.132806e-001,(((7:5.782293e-001,37:1.996303e-002):2.725342e-001,(((29:6.686768e-002,(28:2.360321e-002,33:5.095936e-002):6.934944e-003):1.156903e-001,32:3.745108e-001):5.044784e-002,34:3.653425e-001):9.547867e-002):2.217261e-001,23:2.959882e-002):9.513929e-002):2.054332e-003):5.738750e-001):5.731475e-002,(27:3.159302e-001,31:3.304555e-001):1.733001e-002):1.405235e-001):1.499318e-001,(30:1.065046e-001,(36:2.464971e-001,((26:4.965335e-002,((24:2.955614e-002,25:1.280896e-002):4.388764e-003,21:1.050184e-001):1.020697e-002):2.601225e-003,20:1.436953e-001):8.327915e-002):2.864152e-001):1.199205e-002):1.276633e+000):2.700424e-001):1.917043e-001):2.655483e-001,(5:5.245805e-002,(4:5.095087e-002,40:9.337411e-001):5.993806e-002):7.524818e-002):5.408959e-001):8.195066e-003,19:1.551598e-001,1:2.997885e-001);

tree gen.230000 = [&U] ((((16:3.346172e-002,6:1.277353e-001):8.430662e-002,17:3.091337e-001):1.097995e-001,((((((((11:1.240863e-001,12:4.185884e-002):3.329040e-001,13:5.868324e-003):7.276457e-002,8:2.353646e-003):1.126604e-001,((15:1.477821e-001,3:6.773391e-002):1.768670e-001,14:4.288597e-002):4.513182e-001):9.782268e-002,10:3.930935e-001):1.927000e-001,9:9.685124e-003):2.492203e-001,(18:4.761554e-001,((41:1.322941e+000,42:7.762706e-001):6.006492e-001,(35:4.951579e-001,(31:2.879439e-001,(((36:2.670491e-001,((21:4.365539e-002,(24:2.360036e-002,25:4.367715e-003):2.163163e-001):9.928127e-002,(20:2.706408e-002,26:6.938732e-002):6.794648e-002):5.072290e-002):1.879952e-001,((((39:8.790554e-002,38:3.274108e-001):4.437719e-002,(2:8.418805e-002,(((7:3.802138e-001,37:6.305745e-002):1.872743e-001,(32:4.442049e-001,(34:1.879634e-001,((28:1.397132e-001,33:6.245711e-001):5.018285e-003,29:7.034754e-002):1.050471e-001):1.526957e-001):1.798488e-001):5.669007e-001,23:7.345559e-002):2.894874e-001):2.801522e-002):3.464865e-001,30:7.613316e-002):5.151144e-003,27:3.369389e-001):2.016121e-001):4.477100e-002,22:1.268118e-001):6.663509e-002):1.942331e-003):9.781519e-001):9.888954e-002):2.462750e-001):1.499919e-001,((5:2.773414e-002,4:5.729401e-002):2.226606e-001,40:8.616774e-001):1.738380e-001):3.008163e-001):2.521778e-002,19:1.471355e-001,1:2.107950e-001);

tree gen.231000 = [&U] (19:1.595082e-001,((17:3.584115e-001,(16:4.485692e-001,6:8.497337e-002):5.544245e-002):2.947844e-002,((((((11:2.166932e-001,12:4.668441e-002):3.785068e-001,13:6.529601e-002):9.768915e-002,((10:1.392201e-001,(14:2.275765e-002,(3:4.239758e-002,15:2.244746e-002):6.024130e-002):3.963300e-001):8.846992e-002,8:1.429893e-001):1.087801e-001):2.067930e-001,9:8.816647e-004):4.707037e-001,(18:1.496969e-001,((41:1.346788e+000,42:2.767574e-001):1.207620e+000,((((((((7:4.110345e-001,37:5.949704e-002):5.785677e-002,((34:2.096323e-001,(33:2.653982e-001,(29:1.774938e-001,28:1.836349e-001):1.527445e-003):2.669362e-001):1.354950e-002,32:4.190107e-001):1.701014e-002):8.161548e-002,23:2.016202e-002):1.119076e-001,(39:1.109743e-001,38:3.746969e-001):4.004827e-002):8.338873e-002,2:5.312575e-002):4.555212e-001,(31:2.118377e-001,27:3.613421e-001):1.666139e-002):1.702296e-002,(((36:1.593982e-001,(20:1.937427e-002,((24:3.708081e-002,25:4.871234e-003):1.891998e-001,(26:8.199476e-002,21:2.652225e-001):1.022698e-001):9.005858e-003):8.597339e-002):2.675430e-001,35:5.522407e-001):7.070384e-002,22:1.531271e-001):6.813464e-002):7.659933e-002,30:1.529171e-001):9.622117e-001):2.576551e-001):1.015485e-001):1.720899e-001,((4:2.198812e-001,5:1.227131e-002):5.577836e-002,40:9.534375e-001):2.352016e-001):3.431221e-001):1.206177e-001,1:6.546239e-001);

tree gen.232000 = [&U] ((17:5.821683e-001,((((((3:1.831483e-002,(14:6.445250e-003,15:6.760977e-002):2.607364e-002):5.023757e-001,((11:1.743271e-001,12:7.704442e-002):4.034141e-001,13:6.689619e-002):1.011719e-001):1.761435e-001,(10:2.004741e-001,8:8.492802e-002):1.091795e-001):1.689929e-001,9:1.446760e-001):3.227483e-001,(18:2.699289e-001,((41:1.446882e+000,42:1.043347e-001):5.023103e-001,((((((39:1.117635e-001,38:3.729842e-001):8.160289e-002,2:1.093453e-001):5.902858e-002,(((7:5.048341e-001,37:8.026936e-002):5.377593e-001,(((33:4.098089e-001,28:1.829209e-001):2.589480e-003,29:3.304794e-001):8.874248e-002,(32:4.346775e-001,34:2.269862e-001):1.092383e-002):5.120673e-003):5.956813e-002,23:3.820200e-002):5.301396e-002):3.508629e-001,31:2.156047e-001):2.248092e-001,(22:1.515397e-001,30:8.426039e-002):5.765644e-002):3.143526e-002,(((36:5.722511e-001,(((26:4.195171e-001,(24:8.787192e-002,25:3.006851e-001):1.101745e-001):6.508563e-003,21:7.281287e-003):1.403254e-002,20:1.352923e-001):7.100657e-002):6.822355e-002,35:5.561679e-001):1.138548e-001,27:3.129042e-001):2.148157e-002):4.560936e-001):3.976464e-001):1.555333e-001):4.810496e-001,((40:9.856683e-001,5:1.756707e-004):2.034718e-002,4:2.282813e-001):5.845186e-001):3.942725e-001):9.118017e-002,((16:1.426505e-001,6:8.748798e-002):5.663348e-002,19:2.288883e-001):1.837133e-002,1:1.988412e-001);

tree gen.233000 = [&U] ((((((8:9.701610e-002,10:1.058597e-001):8.185506e-003,(((3:1.533539e-002,14:4.799234e-002):5.556737e-002,15:5.868864e-002):4.573477e-001,((11:1.977140e-001,12:6.451090e-002):3.377870e-001,13:1.083169e-001):3.787749e-002):1.880512e-001):9.368550e-002,9:1.461213e-002):2.659747e-001,(18:2.260171e-001,((41:1.211504e+000,42:8.038318e-002):4.205949e-001,((30:6.923618e-002,(((2:2.354960e-001,(((7:3.898538e-001,37:1.342784e-001):1.698488e-001,(((33:3.401080e-001,(29:2.526630e-001,28:1.422377e-001):4.962212e-002):1.443154e-001,34:1.966187e-001):1.066059e-001,32:1.735722e-001):2.152255e-001):5.254628e-002,23:3.198733e-002):2.949045e-001):5.325388e-002,(39:9.336221e-002,38:1.875457e-001):5.626900e-002):2.288415e-001,((36:7.832875e-002,(21:2.691102e-002,(20:2.112912e-002,(26:3.591066e-001,(24:1.424143e-002,25:8.259619e-003):3.123304e-002):1.271525e-001):1.967260e-003):6.461789e-002):1.900297e-001,35:4.884107e-001):2.647972e-002):1.333823e-001):9.243115e-002,(27:2.725750e-001,(31:2.201519e-001,22:4.979856e-002):3.264837e-004):1.116514e-002):3.802934e-001):1.566850e-001):1.339345e-001):1.786362e-001,((4:1.911447e-001,5:9.370903e-004):1.068593e-001,40:3.435715e-001):1.008480e-001):2.101457e-001,(17:3.443406e-001,((16:5.106881e-002,6:1.368027e-001):1.470650e-001,19:1.927634e-001):6.606426e-004):3.980908e-003,1:3.350875e-001);

tree gen.234000 = [&U] (((16:9.354242e-003,(6:1.290345e-001,19:6.108325e-002):1.001522e-002):1.912864e-001,17:4.186254e-001):7.192826e-002,((((((11:2.376961e-002,12:2.323285e-002):4.759309e-001,13:1.450260e-002):2.394029e-001,(8:1.313575e-001,(((3:2.116386e-003,14:5.363914e-002):8.131003e-002,15:1.076436e-001):5.317023e-001,10:9.646864e-002):1.515879e-001):1.345263e-001):1.062446e-003,9:1.611284e-002):7.837260e-002,(18:1.951323e-001,((41:1.252242e+000,42:3.328405e-001):4.637913e-001,(30:6.422016e-002,(((22:1.651099e-001,(((39:9.898581e-002,38:1.448048e-001):1.370107e-001,(((7:4.298930e-001,37:5.500645e-002):1.715244e-001,(((28:6.743339e-002,29:5.324963e-002):1.131522e-002,33:5.134262e-002):1.818862e-001,(34:1.979172e-001,32:7.784876e-001):1.013891e-001):7.496814e-002):7.164008e-002,23:4.989918e-002):1.797900e-001):1.367913e-001,2:3.895763e-001):3.120298e-001):2.290934e-002,(27:4.169262e-001,((36:3.193445e-001,((21:3.090756e-001,(24:1.325049e-001,25:6.341402e-002):5.866015e-002):8.249680e-002,(26:2.489490e-001,20:2.380012e-002):8.584249e-003):3.463419e-002):3.481357e-001,35:5.385720e-001):2.808757e-002):1.116580e-001):3.180639e-002,31:2.544316e-001):7.491573e-002):4.110251e-001):1.463638e-001):9.287300e-002):3.224642e-001,(40:2.650932e-001,(4:9.181199e-002,5:2.364552e-001):8.119381e-002):5.977171e-002):2.564621e-001,1:3.583332e-001);

tree gen.235000 = [&U] (((((((((15:1.339297e-001,3:7.129974e-002):9.306549e-002,14:1.436322e-001):4.621374e-001,8:1.143295e-001):2.429064e-002,(((11:3.193024e-001,12:2.898143e-002):6.817049e-001,13:1.809103e-002):1.460979e-001,10:1.203431e-001):1.344965e-001):1.042608e-001,9:1.203580e-002):3.285553e-001,(18:1.686308e-001,((41:1.562089e+000,42:3.187771e-001):5.805728e-001,((((((7:4.299252e-001,37:7.603953e-002):1.084395e-001,((34:1.947720e-001,((28:1.565666e-001,29:7.019424e-002):1.373514e-002,33:1.907005e-001):1.034898e-001):7.112502e-002,32:4.034979e-001):2.761593e-001):1.472028e-001,23:2.562082e-003):6.774921e-002,((39:1.189667e-001,38:2.374598e-001):8.931997e-002,2:4.570310e-001):1.701984e-001):3.620369e-001,31:5.017956e-001):9.867237e-002,(22:2.081512e-001,((((36:1.889742e-001,(20:1.303516e-003,((24:1.033316e-001,25:4.177288e-002):1.831881e-001,(26:4.430336e-001,21:3.366670e-001):7.128689e-002):8.890693e-002):2.416020e-001):3.397535e-001,35:5.609208e-001):7.985673e-002,27:3.106708e-001):1.272796e-002,30:1.083910e-001):1.001098e-002):2.585756e-002):2.517219e-001):4.527116e-001):5.843529e-002):3.077738e-001,((5:7.868613e-002,40:1.020186e+000):9.593574e-002,4:7.848402e-002):1.787165e-001):3.090986e-001,17:5.081570e-001):5.428958e-003,((6:1.648191e-001,16:1.423745e-001):1.098292e-001,19:9.082238e-002):2.740223e-001,1:4.300176e-001);

tree gen.236000 = [&U] ((((6:1.913898e-001,((((8:9.431820e-002,(((14:1.463716e-001,(15:1.345288e-001,3:3.659085e-001):6.081910e-002):4.784392e-001,10:1.077414e-001):1.677495e-001,((11:8.928414e-002,12:2.518656e-003):2.078707e-001,13:9.227017e-002):8.852900e-002):1.609370e-001):5.852032e-002,9:1.178616e-002):5.073293e-002,(18:4.686960e-001,((41:1.230492e+000,42:3.121652e-001):5.587307e-001,(((((39:5.741641e-002,38:2.235113e-001):2.411788e-001,(2:8.274201e-002,(((7:3.460608e-001,37:7.807212e-002):1.947704e-001,((34:4.841608e-001,(33:4.280511e-001,(28:1.474778e-001,29:1.798730e-001):1.934965e-001):2.282783e-001):1.212256e-001,32:3.421202e-001):7.778717e-002):1.441496e-001,23:1.977792e-001):8.664107e-003):1.564930e-002):1.349344e-001,(30:5.401813e-002,((36:3.922868e-001,((((24:1.050178e-001,25:1.438800e-001):9.941806e-002,26:6.872522e-001):4.675701e-002,20:2.494050e-002):5.602951e-002,21:9.207489e-003):2.267172e-001):7.747015e-002,35:5.846534e-001):1.036029e-001):7.728356e-002):3.244846e-001,(31:4.448922e-001,22:1.404668e-001):4.557223e-002):1.126227e-001,27:4.248781e-001):2.615365e-001):4.164127e-001):4.979904e-003):3.818475e-001,(5:3.352420e-003,(4:4.099920e-001,40:4.633769e-001):2.596804e-001):2.170121e-001):4.305496e-001):5.997245e-002,16:1.394214e-001):9.802302e-002,17:5.137877e-001):1.576662e-003,19:3.403590e-001,1:6.035693e-001);

tree gen.237000 = [&U] (17:3.870532e-001,((6:4.730966e-001,(16:2.609498e-001,(((18:2.216935e-001,((41:1.266692e+000,42:4.602860e-001):1.251311e+000,((30:4.397242e-002,((36:6.145085e-001,(21:4.835601e-002,(((24:2.945680e-002,25:2.121503e-001):1.406842e-001,20:3.961890e-002):1.411679e-001,26:4.160635e-001):1.492127e-001):2.981753e-001):8.387137e-002,35:8.555194e-001):2.595384e-001):3.123523e-002,(((((((7:6.347876e-001,37:1.339306e-002):1.791749e-001,(32:4.728712e-001,(((28:1.812128e-001,29:2.568765e-001):2.407573e-001,33:2.804048e-001):3.737112e-001,34:7.550338e-001):2.017574e-001):1.735499e-001):3.007088e-001,23:7.832298e-002):2.439700e-001,((39:9.417774e-002,38:3.275638e-001):2.670537e-001,2:1.261620e-001):9.231697e-002):4.696413e-001,22:3.924852e-001):1.069305e-001,27:7.869236e-001):2.812640e-002,31:2.456778e-001):1.091068e-001):5.421724e-001):2.336692e-001):4.267943e-002,(((8:1.792400e-002,10:2.900942e-001):3.066505e-002,(((15:2.262950e-001,3:2.584295e-001):1.006682e-001,14:2.020879e-001):1.732985e-001,((11:1.648735e-001,12:1.790909e-001):3.065043e-001,13:1.163989e-001):4.791310e-002):5.384412e-001):6.081685e-001,9:5.534550e-002):2.232294e-001):4.616959e-001,(40:9.795956e-001,(5:4.740413e-002,4:1.352548e-002):3.579200e-002):1.254331e-001):8.350330e-001):3.333261e-003):1.117981e-001,19:4.989216e-001):5.030801e-002,1:7.508270e-001);

tree gen.238000 = [&U] (17:2.976386e-001,(19:3.223224e-001,((((18:2.140133e-001,((41:1.117726e+000,42:4.591258e-001):1.388049e+000,(30:1.774238e-001,((22:6.989463e-002,(((36:6.007037e-001,(((21:5.636179e-002,20:5.128694e-002):1.256131e-001,26:3.517620e-001):2.072939e-001,(24:6.249789e-002,25:1.877901e-001):1.376176e-001):5.713039e-002):1.736913e-001,35:4.239093e-001):5.624429e-002,(31:4.063714e-001,((2:1.452570e-001,(((7:6.213588e-001,37:1.573334e-002):1.459750e-001,((34:4.143861e-001,32:3.943184e-001):3.365970e-001,(33:1.908787e-001,(28:2.256119e-001,29:3.498136e-001):1.173930e-001):1.715235e-001):3.079717e-001):2.416430e-001,23:2.098048e-002):1.956141e-001):1.863630e-002,(39:9.118242e-003,38:3.173116e-001):3.211171e-001):4.904466e-001):2.197507e-002):3.302959e-002):7.459902e-003,27:4.518450e-001):2.753896e-002):7.728001e-001):2.122410e-001):1.060464e-001,((((((15:7.425653e-002,3:2.140886e-002):1.303737e-001,14:8.032423e-002):4.095541e-001,10:1.756340e-001):1.378627e-001,((11:2.257212e-001,12:1.242760e-002):4.052109e-001,13:1.997741e-002):4.327154e-002):2.759424e-001,8:1.496023e-003):8.997923e-002,9:6.003941e-002):1.969771e-001):2.870793e-001,(40:9.570749e-001,(4:1.732250e-001,5:8.687310e-002):5.130860e-002):1.143932e-001):3.288011e-001,(6:4.090163e-001,16:4.366247e-002):2.429128e-001):1.595943e-001):2.753682e-001,1:6.463265e-001);

tree gen.239000 = [&U] (((16:3.362415e-001,6:5.182690e-001):3.084387e-002,19:4.113485e-001):6.912813e-002,((((18:2.711786e-001,((41:1.416282e+000,42:1.066090e+000):1.433458e+000,(30:1.020722e-001,(((27:5.968572e-001,((36:1.064225e+000,((((24:8.651315e-002,25:5.929051e-002):1.742348e-001,21:7.077999e-003):4.895074e-002,20:1.604664e-002):3.204359e-002,26:1.488091e-001):2.589298e-001):2.184973e-001,35:4.690489e-001):1.277547e-001):2.503152e-002,((((7:6.814096e-001,37:1.993588e-002):3.867420e-002,((33:2.371940e-001,(28:2.416870e-001,29:2.009330e-001):2.426325e-001):3.278817e-001,(34:2.177604e-001,32:7.306373e-001):8.017264e-002):1.420569e-001):4.843253e-001,23:9.803992e-002):1.326963e-001,((39:3.418226e-001,38:6.064764e-001):2.978494e-002,2:2.486468e-001):2.948017e-002):2.954566e-001):7.074631e-002,(31:4.861225e-001,22:3.506750e-002):6.821587e-002):8.706818e-002):6.933445e-001):5.148109e-001):2.804080e-001,((((((11:4.891593e-001,12:2.867130e-001):5.433660e-001,13:5.283211e-002):7.099221e-002,10:5.953056e-001):3.701451e-001,8:7.142984e-004):2.270688e-001,((15:1.670515e-002,3:7.357550e-003):1.644106e-001,14:5.515911e-002):3.469477e-001):8.653209e-002,9:5.393859e-002):4.398066e-001):1.919233e-001,((40:5.711422e-001,4:8.350808e-002):7.038579e-002,5:2.329070e-001):3.255174e-001):4.229198e-001,17:7.528623e-001):2.643899e-001,1:5.386999e-001);

tree gen.240000 = [&U] ((17:4.665840e-001,(((18:4.949729e-001,((41:2.314937e+000,42:1.780394e-001):8.640643e-001,(((27:7.302401e-001,31:2.474125e-001):3.507693e-001,22:3.250878e-001):1.240262e-001,(30:3.262821e-001,(((36:3.803944e-001,(26:3.172074e-001,((24:3.261902e-002,25:7.065383e-002):2.101645e-001,(21:5.191792e-002,20:1.794684e-002):1.454489e-001):3.491684e-002):4.750669e-001):9.355037e-001,35:6.264290e-001):1.531526e-001,(2:2.646155e-001,((39:3.199250e-001,38:3.543245e-001):2.283083e-001,(((7:8.078579e-001,37:1.054863e-001):5.647896e-001,((32:7.064251e-001,34:5.333724e-001):2.451825e-001,((29:1.729722e-001,33:2.663836e-001):3.428603e-002,28:2.825219e-001):3.657706e-001):1.922572e-002):1.435028e-001,23:1.061970e-001):2.413513e-001):4.890177e-004):5.934642e-001):6.191610e-002):1.840014e-001):5.957449e-001):7.752242e-001):1.079130e-001,(((((11:2.604457e-002,12:2.797928e-001):3.781963e-001,13:3.153484e-002):3.846405e-001,(((15:1.121156e-001,3:6.357395e-002):3.893678e-002,14:4.179736e-002):6.999168e-001,10:1.494209e-001):2.296346e-001):1.265472e-001,8:4.320198e-002):2.126932e-001,9:2.671041e-002):2.663734e-001):2.287064e-001,((40:1.621578e+000,5:2.529821e-002):1.422107e-003,4:1.178956e-001):1.469953e-001):1.235306e+000):2.435476e-001,(19:1.724227e-001,(16:6.137349e-001,6:3.630177e-001):9.243872e-003):1.176951e-001,1:6.525955e-001);

tree gen.241000 = [&U] (((19:1.226024e-001,(16:1.405253e-001,6:2.707879e-001):7.624069e-002):1.257058e-002,(((18:3.648912e-001,((41:2.052724e+000,42:1.902379e-001):5.731977e-001,(30:2.224234e-001,((((36:1.908427e-001,(26:7.820735e-001,((20:3.937061e-001,(24:1.193945e-003,25:6.420522e-003):1.392397e-001):8.960741e-002,21:9.265814e-002):1.599085e-001):2.766231e-001):2.999261e-001,35:5.402289e-001):1.929133e-001,((39:1.918209e-001,38:3.632530e-001):2.588030e-001,((((7:2.721699e-001,37:3.251398e-001):3.827639e-001,(((28:4.886960e-001,33:2.471397e-001):4.160314e-002,29:6.574957e-002):2.503547e-001,(34:3.107901e-001,32:5.207726e-001):7.345653e-002):8.194686e-002):2.060217e-001,23:7.548707e-002):2.090901e-001,2:1.953093e-001):2.431032e-002):2.913797e-001):1.021176e-001,(27:3.107610e-001,(22:2.435586e-001,31:2.010154e-001):4.874350e-001):8.299946e-002):2.283461e-001):1.045901e+000):1.186763e-001):3.022267e-001,((10:7.032851e-001,((((11:8.473938e-002,12:2.062617e-001):1.420535e+000,(3:6.318786e-002,(15:1.611482e-002,14:1.063411e-001):1.488998e-002):6.710442e-001):8.108472e-002,13:3.478160e-001):1.183740e-001,8:1.994392e-002):4.315283e-001):1.350091e-001,9:1.969076e-002):2.419568e-001):1.686011e-001,((40:9.781612e-001,5:3.090958e-002):1.081195e-002,4:9.036963e-002):4.057708e-001):6.510568e-001):2.966031e-001,17:3.050712e-001,1:5.124497e-001);

tree gen.242000 = [&U] (16:1.880329e-001,(((19:3.307589e-001,(((18:5.698735e-001,((41:3.205868e+000,42:5.008666e-001):4.656060e-001,(((((((7:4.250648e-001,37:2.015908e-001):1.253382e-001,((29:4.572633e-001,(28:2.642687e-001,33:2.194592e-001):2.946689e-001):3.266298e-001,(34:9.084945e-001,32:8.133232e-001):2.593013e-001):1.805520e-001):4.347568e-001,23:5.016630e-002):1.375367e-001,((39:2.995787e-001,38:7.050110e-001):3.138068e-001,2:1.195406e-001):3.689493e-002):9.110517e-001,31:6.828071e-001):3.869700e-002,((30:3.582845e-001,((36:2.980511e-001,((20:2.257585e-001,(24:1.323236e-001,25:1.002733e-002):2.397702e-001):8.169608e-003,(26:2.684307e-001,21:4.490488e-002):6.154417e-002):6.809620e-001):2.258186e-001,35:6.240483e-001):5.760026e-001):6.915655e-003,22:1.901964e-001):2.470412e-001):4.066960e-002,27:4.638851e-001):1.746185e+000):1.039165e-001):1.431112e-001,((10:6.866876e-001,(((13:2.654325e-002,(11:3.249742e-001,12:1.614720e-002):6.633755e-001):4.428489e-002,(3:5.400354e-002,(14:3.579852e-002,15:1.182085e-002):2.494359e-001):1.030010e+000):6.145266e-002,8:9.927835e-002):2.600732e-003):1.583489e-001,9:5.901240e-002):4.544289e-001):7.011343e-001,((5:1.127309e-001,4:5.017089e-001):1.136782e-002,40:1.504180e+000):4.386278e-001):1.029273e+000):1.122767e-001,6:2.989954e-001):1.449991e-001,17:4.801817e-001):1.046290e-001,1:8.009410e-001);

tree gen.243000 = [&U] (((16:4.267979e-001,17:5.760239e-001):1.227789e-001,19:3.331815e-001):9.593271e-002,(6:2.873102e-001,(((18:3.620828e-001,((41:2.663717e+000,42:5.373207e-001):6.498997e-001,((30:5.781812e-001,((22:1.782284e-001,(((36:4.184078e-001,(20:3.444126e-001,(26:2.444785e-001,((24:1.818924e-001,25:3.361578e-001):4.568079e-001,21:5.303821e-002):1.875505e-001):1.908829e-001):4.632589e-002):6.772333e-001,35:3.473264e-001):2.652243e-001,(((39:7.925301e-001,38:6.677239e-001):1.490413e-001,(((7:4.146586e-001,37:1.358293e-001):1.617722e-001,((34:8.087189e-001,32:4.537330e-001):3.128942e-002,((33:1.275062e-001,29:4.238263e-001):5.107537e-002,28:2.727922e-001):2.308734e-001):1.987438e-001):1.731401e-001,23:5.209026e-001):5.902067e-002):2.626054e-001,2:3.031523e-001):7.132340e-001):9.085797e-002):3.869643e-003,27:3.686532e-001):5.687582e-002):1.526281e-001,31:6.584743e-001):6.203048e-001):2.844561e-001):2.633873e-001,((8:1.206753e-003,((((3:2.717635e-002,15:2.382304e-002):1.806439e-001,14:3.276369e-002):6.265869e-001,10:6.434782e-001):1.965627e-001,(13:1.926135e-002,(11:1.250299e-001,12:2.725945e-001):1.037821e+000):4.052531e-002):2.436214e-001):3.159157e-001,9:8.157483e-002):5.828710e-001):6.546508e-001,(4:2.402451e-001,(5:4.557730e-001,40:1.394019e+000):2.112024e-001):2.116397e-001):1.206700e+000):2.631020e-002,1:1.052710e+000);

tree gen.244000 = [&U] ((((6:4.113830e-001,16:2.817309e-001):4.139084e-003,19:3.843293e-002):1.041957e-001,17:3.885810e-001):1.093308e-001,(((18:8.890708e-002,((41:1.132568e+000,42:3.588670e-001):8.039801e-001,((((((36:2.612549e-001,((24:1.554073e-003,25:4.227244e-002):3.296336e-002,((26:7.336925e-002,21:4.754987e-002):1.794228e-001,20:2.633153e-001):1.414165e-001):2.902642e-001):4.511750e-001,35:3.641406e-001):1.442867e-001,30:3.092172e-001):2.398109e-002,22:1.253114e-001):1.653542e-001,(27:2.467638e-001,((39:2.031258e-001,38:4.459609e-001):8.029376e-002,((((7:2.594116e-001,37:4.485182e-002):1.522627e-001,((32:2.865469e-001,((33:4.272613e-001,28:4.590148e-001):8.086367e-002,29:1.431735e-001):1.722826e-001):6.280283e-002,34:5.401290e-001):9.328455e-002):4.866049e-002,23:5.429689e-002):1.402947e-001,2:1.592707e-001):4.868242e-002):2.869636e-001):1.200821e-001):1.712518e-001,31:3.727417e-001):3.764402e-001):2.258690e-001):1.947068e-001,(((13:2.084583e-001,(11:2.788237e-002,12:4.086639e-002):7.999448e-001):3.507758e-001,(8:6.736990e-002,(10:2.922557e-001,(14:9.471769e-002,(3:5.966485e-002,15:1.169929e-002):7.631010e-002):5.924788e-001):1.933209e-001):1.040920e-002):1.345411e-001,9:9.364059e-002):2.134121e-001):5.267514e-001,((5:9.564275e-002,4:8.687049e-002):1.331353e-001,40:2.991636e-001):4.537399e-001):7.519175e-001,1:4.886847e-001);

tree gen.245000 = [&U] ((17:3.411810e-001,(6:4.498962e-002,16:8.587848e-002):2.167305e-001):8.524566e-002,(19:1.928794e-001,(((18:1.579996e-001,((41:1.493647e+000,42:2.999346e-001):4.780792e-001,(31:1.752840e-001,(22:3.848630e-001,(((((36:2.183522e-001,((20:7.752299e-003,26:2.335389e-001):7.279924e-002,(21:6.152436e-002,(24:4.124999e-003,25:3.533055e-002):6.841299e-002):1.249938e-002):1.436511e-001):3.079445e-001,35:2.370848e-001):3.092440e-001,30:7.195822e-002):1.081367e-002,((((7:4.641800e-001,37:3.737125e-002):4.461615e-001,(34:4.342179e-001,(((33:1.252375e-002,29:2.975478e-001):2.891279e-002,28:1.292356e-001):3.390518e-001,32:2.436511e-001):6.124176e-003):6.374188e-002):1.152121e-001,23:4.538037e-002):1.217588e-001,(2:4.335701e-001,(39:7.667661e-002,38:3.879311e-001):1.762673e-001):2.573745e-003):2.506745e-001):2.387410e-002,27:1.591566e-001):5.100877e-002):1.350252e-001):4.692088e-001):2.347521e-001):5.352043e-001,((((14:8.953519e-002,(15:1.059947e-001,3:6.240173e-002):2.309998e-001):4.532642e-001,(10:1.284640e-001,(13:1.545736e-002,(11:8.482701e-002,12:1.847892e-003):5.527184e-001):3.190604e-001):5.094149e-003):5.113888e-002,8:5.604744e-002):1.077900e-001,9:7.174473e-002):2.078996e-001):4.594015e-001,((40:1.903419e-001,4:8.595284e-002):5.753682e-001,5:1.928159e-002):1.670252e-001):4.435022e-001):2.984450e-002,1:3.119521e-001);

tree gen.246000 = [&U] ((19:1.798861e-001,17:2.812168e-001):7.688037e-002,((((18:2.176575e-001,((41:2.057621e+000,42:2.875570e-001):1.036817e+000,((((((((29:5.210241e-002,28:1.494235e-001):4.317168e-002,33:1.307052e-001):1.906963e-001,32:4.883549e-001):1.351620e-001,(7:5.503578e-001,37:5.148197e-002):4.054315e-001):5.903662e-002,34:3.125571e-001):1.207483e-001,23:2.410371e-002):2.448245e-001,((39:6.812760e-002,38:3.117828e-001):1.295919e-001,2:2.570520e-001):3.388761e-001):4.826659e-001,((((((36:3.007980e-001,((20:7.116055e-002,((24:1.854513e-001,25:1.241456e-001):5.594026e-002,21:5.396876e-002):8.411856e-002):2.711260e-002,26:1.056862e-001):1.339554e-001):2.634212e-001,35:3.266038e-001):4.529896e-002,22:6.024032e-001):5.248000e-002,30:1.021668e-001):9.015405e-003,31:3.308958e-001):4.810359e-003,27:2.068726e-001):3.515827e-002):5.333164e-001):2.605479e-001):5.125436e-002,((((13:2.223072e-001,(11:1.168562e-001,12:6.645775e-002):7.614150e-001):1.175560e-001,(10:1.283111e-001,(3:1.527389e-002,(15:6.323389e-003,14:1.279859e-001):5.698883e-002):1.365523e-001):2.326865e-002):4.424029e-001,8:2.915829e-004):9.943828e-002,9:8.947148e-003):2.137526e-001):3.687090e-001,((4:1.179805e-001,5:9.243043e-002):5.825179e-002,40:2.749074e-001):5.339798e-001):3.308990e-001,(6:3.966889e-002,16:7.297939e-002):1.299206e-001):4.857185e-001,1:2.753740e-001);

tree gen.247000 = [&U] ((19:1.674121e-001,17:2.977945e-001):6.246624e-002,((((18:2.492123e-001,(((13:2.268052e-001,(11:3.121129e-001,12:1.021582e-001):3.560273e-001):3.740125e-002,((10:1.309073e-001,((15:8.277362e-002,3:1.248899e-001):3.626024e-002,14:5.505871e-002):7.286248e-001):7.283585e-002,8:3.161089e-002):1.801807e-002):6.824110e-002,9:5.218175e-002):3.622270e-001):1.068344e-001,((41:6.821295e-001,42:1.774816e-001):9.509184e-001,(31:3.622191e-001,((22:4.667927e-002,30:9.988035e-002):4.843809e-002,(((36:3.068842e-001,((((24:7.424337e-003,25:4.262194e-002):2.043395e-001,20:1.433014e-001):7.810223e-003,21:6.978614e-002):2.705096e-002,26:5.098021e-001):1.775918e-001):2.687511e-001,35:3.367778e-001):3.548534e-002,(27:2.033362e-001,((39:2.043585e-002,38:6.810116e-001):8.881165e-002,(2:6.197242e-002,(((32:5.844425e-001,(((33:1.972223e-001,29:1.762649e-001):9.955729e-003,28:4.157138e-001):4.865006e-002,34:2.866216e-001):8.442345e-002):3.378690e-002,(7:7.877070e-002,37:2.078265e-002):6.471923e-001):2.516710e-001,23:1.769127e-001):7.024639e-002):7.087553e-002):2.388696e-001):1.494633e-003):1.882474e-002):4.900427e-002):3.427936e-001):1.720724e-001):3.737030e-001,(4:2.098098e-002,(40:5.688779e-001,5:6.923879e-002):1.825622e-002):2.329874e-001):6.238562e-001,(6:6.724259e-002,16:1.029728e-001):1.114478e-001):4.612398e-002,1:4.252631e-001);

tree gen.248000 = [&U] (((((18:2.860409e-001,((41:1.276061e+000,42:5.534027e-001):9.227568e-001,(((36:2.000713e-001,((26:6.877969e-001,((24:6.021543e-002,25:7.647183e-002):1.660796e-001,20:7.367344e-003):3.333958e-002):8.986217e-002,21:5.289766e-002):3.005570e-001):1.820536e-001,35:3.397993e-001):1.112382e-001,((30:1.125569e-001,31:6.452363e-001):1.814231e-002,((27:6.013838e-001,((38:4.680543e-001,39:9.827854e-002):7.214800e-002,(2:9.427773e-002,(((((33:2.370051e-001,29:4.554608e-002):5.278995e-002,28:7.456658e-002):1.633453e-001,(34:1.168528e-001,32:8.105219e-001):9.859510e-002):2.074832e-002,(7:5.057964e-001,37:1.033210e-002):1.416272e-001):3.538276e-002,23:1.078191e-001):3.978023e-002):9.836154e-003):3.752538e-001):8.134645e-002,22:1.037795e-001):1.922785e-001):1.257806e-001):4.040427e-001):3.387714e-001):2.126726e-001,(((10:1.125835e-001,(((3:1.667079e-002,15:1.138239e-001):4.771175e-002,14:1.618130e-003):1.906316e-001,((11:3.861610e-001,12:1.420184e-002):6.459852e-001,13:3.242254e-001):3.439918e-003):5.078576e-002):4.471938e-001,8:8.427585e-002):1.488275e-001,9:5.734993e-002):4.684424e-001):4.462255e-001,(40:7.356893e-001,(4:1.053763e-001,5:5.105975e-002):9.326731e-002):6.758950e-001):5.531040e-001,((6:6.473999e-002,16:9.834197e-002):7.780867e-002,17:5.470899e-001):1.298897e-002):5.548799e-002,19:8.194980e-002,1:5.583594e-001);

tree gen.249000 = [&U] ((19:9.014591e-002,17:1.855495e-001):1.248621e-001,((16:8.465901e-002,6:1.129672e-001):3.798963e-002,(((18:1.237219e-001,((41:1.041261e+000,42:4.515744e-001):6.265300e-001,((((36:2.085623e-002,((24:5.722770e-002,(20:2.305481e-002,(21:7.599787e-004,26:1.468652e-001):2.890551e-002):1.440934e-001):6.537084e-002,25:6.240071e-002):2.563892e-001):1.956872e-001,35:4.304863e-001):8.479015e-002,((((38:4.677651e-001,39:1.612895e-001):1.327741e-001,((((((33:1.993021e-001,29:3.203256e-001):9.975468e-002,28:7.438929e-002):5.363944e-001,(34:2.701993e-001,32:2.829213e-001):1.758984e-002):1.694670e-002,(7:2.798397e-001,37:8.430955e-003):2.587121e-001):8.456827e-002,23:8.248709e-003):2.136005e-002,2:1.290488e-001):5.550639e-003):2.309337e-001,31:1.244887e-001):1.913219e-002,30:1.426866e-001):1.403723e-001):8.482216e-002,(22:3.269392e-001,27:4.090657e-001):3.383859e-002):6.670029e-001):3.347794e-001):1.735400e-001,((((13:3.166215e-002,(11:2.656063e-001,12:1.033647e-001):5.518589e-001):5.962187e-002,8:1.187694e-002):9.290507e-004,(((15:1.271195e-001,3:1.269988e-001):2.512546e-001,14:6.778141e-002):3.833204e-001,10:2.458872e-001):1.618621e-001):1.222128e-001,9:4.679731e-002):3.822471e-001):4.602800e-001,(40:6.110011e-001,(5:5.902545e-002,4:1.451109e-001):6.896424e-002):3.135313e-001):2.328948e-001):4.706270e-001,1:6.077651e-001);

tree gen.250000 = [&U] (17:2.614201e-001,((19:6.595850e-002,(16:6.172385e-002,6:8.557165e-002):1.351132e-001):1.019627e-001,(((18:3.431900e-001,((41:7.887459e-001,42:1.059090e-001):4.104239e-001,(((36:2.508626e-001,(((24:7.814329e-003,25:1.466033e-001):1.940246e-001,(20:3.523773e-002,26:1.425184e-001):5.235488e-002):8.978790e-002,21:3.349854e-001):2.636820e-001):8.424216e-002,35:6.053994e-002):6.721697e-002,(((2:1.615402e-001,((((((33:1.003712e-001,28:5.477989e-002):2.503765e-002,29:9.139265e-002):3.410973e-001,(34:2.812949e-001,32:5.086988e-001):1.010186e-001):2.646357e-001,(7:1.794963e-001,37:1.361393e-001):1.268554e-001):4.035596e-001,23:4.174353e-002):1.407140e-003,(38:3.543278e-001,39:7.432600e-002):1.439035e-001):1.782524e-001):1.793911e-001,(27:2.959154e-001,22:1.275191e-001):6.584192e-002):1.706622e-001,(30:1.014026e-001,31:1.985392e-001):1.784755e-003):9.891688e-003):5.263203e-001):2.535924e-001):8.464115e-002,(((13:3.295898e-002,(11:3.798261e-002,12:9.450248e-002):4.987129e-001):1.109766e-001,(((15:5.339378e-002,(14:5.179540e-002,3:1.441116e-002):1.150759e-002):2.903617e-001,10:8.772998e-002):1.743935e-001,8:2.131681e-003):1.209932e-002):3.741654e-002,9:4.794525e-002):5.666275e-002):5.555934e-001,(40:5.333161e-001,(4:2.940834e-002,5:4.655603e-002):5.027124e-002):1.387490e-001):1.484571e-001):3.387318e-002,1:5.448578e-001);

tree gen.251000 = [&U] ((((16:2.617888e-001,6:7.287090e-002):1.188050e-001,17:2.595929e-001):3.934164e-002,19:8.803242e-002):4.781302e-002,(((18:3.819160e-001,((41:8.777490e-001,42:2.035034e-001):3.540425e-001,(((36:2.131859e-001,(21:5.454743e-002,((20:4.294767e-002,26:1.651613e-001):1.895711e-002,(24:2.852642e-002,25:1.016345e-001):4.765701e-002):2.739936e-001):2.163881e-001):2.755996e-002,35:6.826348e-002):5.939586e-002,((31:1.874030e-001,27:3.203127e-001):5.111173e-002,(30:2.447715e-002,(22:7.272218e-002,((((((34:3.182781e-001,32:5.944102e-001):2.724696e-001,((29:1.075804e-001,33:1.113283e-001):2.078157e-002,28:9.151783e-002):5.094306e-002):2.789377e-001,(7:1.997509e-001,37:6.728488e-002):8.892987e-002):1.360595e-001,23:1.083006e-002):2.149169e-002,2:1.797686e-001):1.029793e-003,(38:4.434052e-001,39:2.359250e-001):6.165244e-002):3.428950e-001):2.426898e-002):1.507086e-003):1.092426e-001):4.640396e-001):3.001270e-001):3.829076e-002,((8:2.910916e-002,(10:1.797280e-001,((((14:4.915098e-002,15:9.587438e-003):9.236966e-002,3:4.563425e-002):3.185660e-001,13:1.113295e-001):3.547705e-002,(11:1.135577e-001,12:1.051663e-001):3.809539e-001):1.404022e-001):2.077517e-001):1.854567e-001,9:1.163576e-002):1.303741e-001):1.797190e-001,(40:5.934963e-001,(5:1.716422e-002,4:3.254006e-002):1.055900e-001):1.723075e-002):3.268810e-001,1:5.956840e-001);

tree gen.252000 = [&U] ((((6:7.361400e-002,16:1.905370e-001):5.481474e-002,19:1.081610e-001):2.246004e-001,(((18:3.452689e-001,((41:8.331147e-001,42:2.197707e-001):6.100343e-001,(31:9.691934e-002,(22:1.016226e-001,((((((38:5.142193e-001,39:8.882897e-002):1.805679e-001,2:1.423000e-001):6.606845e-003,(((32:2.503579e-001,(34:3.128885e-001,((29:6.817519e-002,33:1.435723e-001):6.874935e-002,28:9.740638e-002):1.339057e-001):8.885928e-002):1.752770e-001,(7:2.157183e-001,37:2.050280e-001):2.547643e-002):1.176711e-001,23:1.066759e-002):7.774291e-002):4.054539e-001,27:3.570607e-001):5.704433e-003,((36:4.087312e-001,((26:4.255641e-001,21:1.079442e-001):3.634436e-002,(20:1.336618e-001,(24:2.635111e-002,25:2.593968e-002):1.634810e-001):8.154917e-002):8.851662e-002):1.378919e-001,35:8.414372e-002):5.086909e-002):2.301730e-001,30:1.132918e-001):2.051639e-002):9.647547e-002):2.299806e-001):2.896611e-001):2.808859e-002,(((10:8.812143e-002,((14:6.473615e-002,15:7.111802e-002):6.348488e-002,3:2.909662e-001):3.949526e-001):1.899398e-001,(13:8.615418e-002,(11:9.953786e-002,12:7.233737e-002):4.885879e-001):1.101728e-001):1.544849e-001,(8:4.701230e-002,9:2.005403e-001):1.489412e-001):3.986642e-001):8.206932e-002,(4:1.862545e-001,(40:5.174925e-001,5:4.228775e-002):2.516747e-002):9.431810e-002):3.809117e-001):1.141904e-001,17:2.832204e-001,1:6.158915e-001);

tree gen.253000 = [&U] (17:2.298218e-001,((((9:8.829670e-002,(8:1.073173e-002,(((11:7.982896e-002,12:5.801428e-002):3.861318e-001,(13:1.696345e-001,((14:5.679252e-003,3:1.171520e-002):1.587269e-001,15:8.324122e-002):1.451253e-001):1.518727e-001):1.414826e-001,10:9.389870e-002):1.166282e-001):1.100937e-001):7.487306e-002,(18:3.212838e-001,((41:6.681546e-001,42:2.842090e-001):4.319530e-001,(30:1.356344e-001,(27:3.633852e-001,((2:2.621158e-001,(((((32:1.992963e-001,((33:4.247516e-002,28:1.090997e-001):7.024566e-002,29:1.315001e-001):9.881346e-002):1.005368e-001,34:3.218877e-001):3.034130e-002,(7:6.558947e-001,37:3.681340e-002):2.544677e-001):1.427767e-002,23:8.312433e-002):1.201925e-002,(38:3.115912e-001,39:7.392740e-002):6.836041e-002):6.539564e-003):1.498488e-001,(31:5.061434e-001,(((36:7.950572e-002,((20:3.773012e-001,21:2.071734e-002):4.166234e-002,(26:2.142017e-001,(24:5.205242e-002,25:1.697988e-002):8.755583e-002):3.654059e-002):1.073777e-001):3.296916e-002,35:5.376222e-001):1.368875e-002,22:9.632639e-002):5.690147e-002):5.117656e-003):6.928077e-002):4.141506e-002):3.603028e-001):2.690614e-001):1.321178e-001):1.708267e-001,(40:2.864327e-001,(4:3.773031e-002,5:5.989299e-002):1.080554e-001):1.578237e-001):2.373969e-001,((16:1.194194e-001,6:6.520216e-002):1.536090e-001,19:8.955977e-002):6.549222e-002):4.543630e-002,1:3.334379e-001);

tree gen.254000 = [&U] ((((9:6.635424e-002,(((10:1.735763e-001,(13:1.037492e-002,(11:2.575041e-001,12:6.348253e-002):5.122006e-001):8.888787e-002):3.595763e-003,8:8.226171e-002):8.397178e-002,((3:2.752665e-002,15:4.770076e-002):1.687831e-001,14:4.169463e-002):2.459458e-001):1.467594e-001):2.026737e-001,(18:4.108184e-001,((41:1.590563e+000,42:5.007094e-001):3.175839e-001,((2:7.670425e-002,((((34:1.817056e-001,((28:2.704588e-001,(29:6.933398e-002,33:8.804173e-002):4.440483e-003):2.043759e-001,32:1.013863e-001):1.457605e-001):9.899528e-002,(7:3.435094e-001,37:3.882204e-002):1.957908e-001):3.133055e-002,23:2.549656e-002):9.944913e-002,(38:2.703452e-001,39:1.540988e-001):1.577114e-001):3.658741e-001):1.559550e-001,(22:1.063165e-001,(27:5.489017e-001,((31:3.142881e-001,((36:1.588954e-001,(26:1.916001e-001,(21:3.388044e-002,((24:2.756056e-001,25:7.582101e-003):1.124058e-001,20:7.713099e-003):3.070570e-002):3.603544e-002):9.715660e-002):5.770491e-002,35:1.251154e-001):1.168911e-001):1.232044e-002,30:3.986254e-001):1.437679e-001):1.401215e-002):3.538631e-002):6.005012e-001):2.019486e-001):9.114208e-002):1.656555e-001,(5:6.837094e-004,(40:2.818875e-001,4:2.227759e-001):2.337765e-002):2.600615e-001):1.800652e-001,(((16:3.882380e-002,6:6.032707e-002):1.363636e-001,17:2.643224e-001):1.085281e-001,19:2.824227e-002):4.981618e-001,1:4.824032e-001);

tree gen.255000 = [&U] ((17:2.940363e-001,(6:3.482819e-001,(19:1.110833e-001,16:3.536979e-002):5.748097e-002):1.042092e-001):3.122112e-002,(((9:5.576568e-002,(((15:2.236954e-002,(14:3.374819e-002,3:1.080735e-001):1.710844e-002):2.783251e-001,(13:1.988085e-002,(11:2.345948e-001,12:3.227013e-002):4.034916e-001):9.382798e-002):1.446976e-001,(10:1.617344e-001,8:2.720400e-003):6.977095e-002):1.890696e-001):2.067867e-001,(18:2.844038e-001,((41:1.449056e+000,42:1.052371e-001):6.416313e-001,((((((38:2.462936e-001,39:3.007823e-001):3.393153e-002,(((((28:2.278592e-001,(29:1.127422e-002,33:8.732409e-002):1.555111e-002):1.345190e-001,34:1.660352e-001):5.048598e-002,32:1.857890e-001):8.565394e-002,(7:3.129485e-001,37:2.421006e-002):1.458832e-001):8.193258e-002,23:4.482068e-002):7.491497e-002):4.194789e-004,2:1.078860e-001):2.867668e-001,30:4.805892e-002):1.991709e-001,31:2.911521e-001):4.123235e-002,(22:1.114838e-001,(27:9.992209e-002,((36:2.254398e-001,(26:1.304620e-001,((20:9.813986e-004,(24:9.458061e-002,25:1.143216e-002):6.087586e-002):6.414545e-002,21:7.891079e-003):4.529950e-003):2.215291e-001):1.089102e-001,35:3.501452e-001):2.703196e-002):2.821389e-002):1.978636e-002):3.762364e-001):2.431696e-001):6.851406e-002):1.242840e-001,((40:3.472313e-001,4:1.290021e-001):1.341646e-002,5:8.684953e-003):8.014324e-002):3.908051e-001,1:1.980228e-001);

tree gen.256000 = [&U] (((17:2.799204e-001,(((9:1.931982e-002,((8:1.570976e-002,(10:2.009624e-001,((14:3.928246e-002,15:5.215884e-003):1.104603e-001,3:8.342967e-002):1.922975e-001):2.055526e-001):2.479287e-002,(13:1.361670e-002,(11:9.174895e-002,12:2.144775e-002):4.240329e-001):1.212285e-001):1.017838e-001):1.443534e-001,(18:1.579365e-001,((41:1.388257e+000,42:1.364536e-001):4.022541e-001,(((31:3.134171e-001,(22:1.094201e-001,(30:1.480791e-001,27:5.947558e-001):1.717402e-002):2.197581e-002):4.073220e-002,((36:1.228964e-001,((26:1.256327e-001,20:7.026090e-002):9.525337e-002,(21:2.211935e-001,(24:1.799752e-002,25:8.961368e-002):8.912951e-003):3.040169e-002):6.475523e-002):3.310604e-002,35:2.215661e-001):1.790187e-001):3.849307e-002,((38:4.058543e-001,39:2.185728e-001):6.665072e-002,(2:1.523069e-001,(((7:2.311937e-001,37:2.614961e-001):1.346286e-001,(((29:6.833844e-002,33:8.639809e-002):3.304794e-002,28:1.275610e-001):1.303941e-001,(32:2.361140e-001,34:1.328456e-001):8.046767e-002):1.005699e-001):1.524932e-001,23:7.286177e-002):8.686191e-002):2.265388e-002):1.998766e-001):3.684955e-001):1.410457e-001):8.226615e-002):4.035339e-001,((5:9.666668e-002,4:1.374473e-001):1.171233e-001,40:8.421491e-001):8.740743e-002):3.861761e-001):3.864534e-003,(6:1.171466e-001,16:6.763263e-002):6.611273e-002):8.692070e-003,19:1.642146e-001,1:2.950380e-001);

tree gen.257000 = [&U] (((6:8.844041e-002,16:4.507310e-003):3.771189e-002,(19:8.306665e-002,17:2.554607e-001):3.814155e-003):1.001767e-001,(((9:1.590043e-002,(((((11:4.708521e-002,12:4.067679e-002):4.095292e-001,13:5.637733e-002):3.914869e-002,10:1.605341e-001):2.493584e-002,((14:1.529833e-002,15:6.293427e-002):9.261947e-002,3:3.703477e-002):3.882402e-001):2.226778e-003,8:3.486076e-003):5.888659e-002):1.951832e-001,(18:2.087084e-001,((41:9.602575e-001,42:1.038334e-001):3.240405e-001,(30:1.884920e-001,((((27:5.187694e-001,((36:1.562488e-001,((20:6.514389e-002,(24:4.124372e-002,25:6.655122e-002):8.169378e-002):1.258397e-002,(26:6.146888e-002,21:1.182874e-001):9.216262e-002):1.206832e-001):2.206099e-003,35:2.458123e-001):4.952978e-002):3.284319e-002,22:8.296538e-003):1.327715e-002,(((38:3.829064e-001,39:1.179841e-001):2.395912e-002,2:1.253503e-001):4.508020e-002,((((((29:1.675998e-001,33:1.064089e-001):1.112543e-002,28:2.235404e-001):1.074118e-001,32:1.869428e-001):1.866248e-002,34:1.083035e-001):9.054633e-002,(37:2.702421e-002,7:4.906273e-001):2.458536e-001):3.019531e-002,23:2.161526e-002):1.904385e-001):1.186471e-001):2.704371e-002,31:3.763862e-001):4.525640e-002):3.329105e-001):1.504128e-001):1.516515e-001):2.177638e-001,((40:6.668237e-001,5:1.189821e-001):5.752799e-003,4:2.996720e-002):1.603428e-001):1.944840e-001,1:2.473987e-001);

tree gen.258000 = [&U] ((17:2.915776e-001,(((18:1.020669e-001,((41:1.027388e+000,42:3.302822e-001):3.639784e-001,((30:2.817964e-002,(((38:2.576220e-001,39:1.048418e-001):1.606760e-001,(2:8.176961e-003,((((33:8.414216e-002,(29:1.782489e-001,28:7.202281e-002):2.060482e-001):2.904358e-001,(32:1.876662e-001,34:1.955952e-001):6.993208e-003):3.447139e-001,(37:2.842340e-002,7:5.434216e-001):1.161367e-001):4.657761e-002,23:3.417413e-002):1.687542e-001):4.564979e-002):1.543769e-001,(31:2.597316e-001,(22:4.973815e-002,((36:1.304026e-001,((21:1.616698e-003,(20:1.295228e-001,(24:1.995311e-002,25:5.389813e-003):1.201440e-001):5.897363e-003):4.372682e-002,26:3.795366e-002):1.081683e-001):8.118109e-002,35:1.043182e-001):2.337686e-002):1.825780e-002):4.186978e-002):3.707693e-002):6.233290e-004,27:1.308759e-001):4.244051e-001):1.796300e-001):3.291702e-002,(9:3.812132e-002,(13:1.232456e-001,((10:2.195429e-001,8:3.369862e-002):5.294771e-002,(((14:1.597051e-002,3:4.008607e-003):3.240834e-002,15:4.251087e-002):2.896779e-001,(11:1.620213e-001,12:2.338073e-002):3.912069e-001):1.376325e-002):1.060971e-001):4.252388e-002):1.596626e-001):2.671657e-001,(5:5.366046e-002,(4:1.388347e-001,40:7.123060e-001):1.200362e-001):3.334807e-001):5.522150e-001):9.213871e-002,((16:1.266616e-001,19:1.468456e-001):1.307048e-002,6:6.783012e-002):1.454281e-002,1:2.911855e-001);

tree gen.259000 = [&U] (((5:8.050386e-002,(40:8.209059e-001,4:9.790393e-002):6.557243e-002):1.061868e-001,((18:2.362417e-001,((41:9.278755e-001,42:2.364912e-001):6.427204e-001,(((((36:1.462533e-001,(21:6.199607e-002,(((24:4.127979e-003,25:5.318339e-002):1.093978e-001,26:1.789229e-001):4.770282e-002,20:2.807640e-002):3.825980e-002):9.634391e-002):2.490071e-002,35:4.525240e-001):6.880517e-002,((22:1.088232e-001,(((((32:1.782487e-001,34:2.456378e-001):1.996414e-001,(33:1.480941e-001,(29:1.022148e-001,28:5.573768e-002):1.036263e-002):1.092976e-001):1.272289e-001,(37:1.245250e-001,7:5.437138e-001):1.627092e-001):2.054541e-001,2:2.034980e-002):4.121894e-003,(23:2.076262e-002,(39:1.044416e-001,38:1.981910e-001):1.299795e-001):2.003146e-001):3.069551e-001):1.872932e-001,27:4.023294e-001):3.321964e-002):5.258570e-002,31:4.125930e-001):5.241303e-003,30:1.090071e-001):4.628717e-001):2.402087e-001):4.177210e-002,(9:2.068156e-002,((13:1.447764e-001,((14:2.866286e-002,(15:3.407612e-002,3:3.496437e-002):1.304629e-001):4.599128e-001,((11:2.488981e-001,12:8.600067e-002):5.215589e-001,10:2.126669e-001):5.784850e-002):3.450430e-001):5.740965e-002,8:1.387628e-001):1.224832e-001):7.404223e-002):3.661548e-001):5.297275e-001,(17:3.025749e-001,(6:7.843465e-002,(16:2.290316e-001,19:9.354193e-002):3.648726e-002):9.702519e-003):4.897444e-002,1:4.054992e-001);

tree gen.260000 = [&U] (((5:4.576787e-002,4:1.807517e-001):1.954211e-001,(((9:9.255726e-002,((8:2.776915e-002,10:1.153107e-001):3.014843e-002,(((15:1.639502e-002,14:1.895218e-002):1.405403e-002,3:2.311880e-002):3.171008e-001,(13:2.724202e-003,(11:2.835252e-001,12:5.686453e-002):2.572913e-001):1.185568e-001):7.249395e-002):2.472324e-002):1.002761e-001,(18:1.439774e-001,((41:5.700626e-001,42:1.563704e-001):3.760487e-001,((((36:2.733625e-001,((20:1.859806e-002,26:1.073710e-001):1.844224e-002,((24:5.822419e-002,25:5.981799e-003):4.131647e-002,21:2.132509e-002):6.742569e-003):1.016695e-001):6.553335e-002,35:2.992135e-001):6.286789e-002,22:6.849510e-002):2.276432e-002,(27:1.312720e-001,(30:4.843337e-002,(((23:3.733059e-003,(((32:1.406101e-001,34:1.670194e-001):5.633275e-002,((28:9.932932e-002,33:9.788743e-002):8.718569e-003,29:6.758546e-002):1.465874e-001):2.357815e-002,(37:3.548939e-002,7:2.591049e-001):2.648575e-001):1.267829e-001):1.853155e-002,((39:1.931984e-001,38:2.079089e-001):8.583513e-002,2:3.210402e-002):3.941130e-002):2.960164e-001,31:3.392189e-001):2.831237e-002):3.906901e-002):2.094452e-002):4.701540e-001):5.355671e-002):3.025613e-002):1.579568e-002,40:5.577403e-001):1.062586e-001):2.762076e-001,(((6:4.133090e-002,16:5.061804e-002):9.418185e-002,19:6.627470e-002):2.684258e-002,17:1.478011e-001):2.742804e-002,1:2.639631e-001);

tree gen.261000 = [&U] ((((40:5.757298e-001,(((((41:5.286722e-001,42:1.450168e-001):2.530063e-001,((31:5.663858e-002,((2:1.793568e-002,(39:5.497413e-002,38:1.265556e-001):9.723842e-002):7.784933e-002,(23:2.311085e-002,(((((28:1.300523e-001,29:1.853448e-001):3.301975e-002,33:1.038345e-001):1.763727e-002,32:1.381862e-001):1.434635e-002,34:7.724411e-002):5.075618e-002,(37:3.430933e-002,7:3.117004e-001):1.444656e-001):9.804015e-002):1.968922e-002):2.858482e-001):4.409992e-002,(((27:5.435136e-001,22:9.091943e-002):1.942995e-002,30:4.824672e-002):9.450689e-003,((36:1.364260e-002,(((20:1.095251e-002,21:1.986538e-002):2.858883e-002,(24:2.648516e-003,25:1.800702e-002):5.213585e-002):8.613567e-003,26:9.941008e-002):5.977220e-002):3.137496e-001,35:1.430494e-001):2.115724e-002):2.814855e-002):4.601813e-001):1.312410e-001,(9:7.119261e-003,(((((14:5.979499e-002,15:4.229611e-002):3.586814e-002,10:6.277448e-002):3.485694e-003,3:9.250642e-003):4.338164e-002,(13:4.689936e-002,(11:6.459338e-002,12:9.014218e-004):1.629331e-001):4.311589e-002):4.125366e-001,8:7.298752e-002):1.943646e-001):2.008741e-001):2.948293e-002,18:1.355078e-001):5.758083e-002,(5:2.060591e-002,4:1.676279e-001):1.621032e-001):3.393991e-002):3.109755e-001,(6:1.409855e-001,16:1.250660e-002):2.449658e-003):7.157086e-002,17:1.396620e-001):1.039843e-001,19:7.223502e-002,1:1.070780e-001);

tree gen.262000 = [&U] (17:1.621547e-001,((16:5.671635e-002,(6:7.387251e-002,(((5:8.416211e-003,4:7.771899e-002):8.081887e-002,40:2.989032e-001):1.814981e-001,(((9:7.351358e-003,(((10:6.500419e-002,(3:1.062323e-001,(13:1.397235e-002,(11:8.533466e-002,12:1.892051e-002):1.687212e-001):1.385720e-001):5.227792e-003):1.580660e-002,(14:5.921904e-002,15:8.188395e-003):7.969593e-002):3.615065e-001,8:1.095328e-001):1.116801e-001):1.105490e-001,18:1.708262e-001):2.494639e-002,((41:6.383492e-001,42:1.497446e-001):2.297302e-001,((27:4.840110e-002,((((39:4.155582e-002,38:1.714775e-001):3.998570e-002,(23:1.074946e-002,((32:1.009060e-001,((28:1.959593e-001,(33:1.220405e-001,29:5.381564e-002):1.823186e-003):4.182432e-002,34:1.489421e-001):3.500204e-002):2.774297e-002,(37:4.964141e-002,7:3.218622e-001):7.305206e-002):2.028607e-002):7.689880e-002):3.932838e-002,2:8.364532e-002):3.110564e-001,(((36:1.057651e-001,(26:1.076560e-001,((24:7.796495e-002,25:3.115504e-002):1.504233e-001,(21:1.212636e-001,20:4.441001e-002):8.618489e-003):5.803143e-002):1.886160e-001):1.566051e-001,35:2.590082e-001):1.399732e-001,22:5.241738e-002):4.906039e-002):1.733257e-002):6.099001e-003,(30:5.056912e-002,31:5.377843e-002):6.322508e-002):2.144897e-001):2.892689e-002):2.308222e-001):2.765234e-001):1.567710e-001):1.440450e-002,19:7.174146e-002):1.751372e-001,1:1.059922e-001);

tree gen.263000 = [&U] (((16:6.468407e-002,(19:2.630785e-001,6:8.508833e-002):8.970141e-002):7.717643e-002,((40:3.480869e-001,(5:2.195742e-003,4:1.488804e-001):1.032504e-001):1.690242e-001,(((10:6.590110e-002,9:1.231911e-001):4.708283e-002,(8:1.817268e-002,((14:2.780624e-002,(3:5.711792e-002,15:5.822377e-003):8.674571e-002):1.244866e-001,(13:1.296829e-002,(11:9.829075e-002,12:1.706681e-002):1.943376e-001):5.517164e-002):1.218111e-001):1.169178e-001):2.513048e-001,(18:1.243743e-001,((41:7.581555e-001,42:3.049237e-001):2.830993e-001,(31:1.306524e-001,(30:1.108224e-001,(((2:1.031136e-001,(39:4.737859e-002,38:2.224165e-001):1.193978e-002):1.338820e-001,(23:1.374105e-001,((((29:3.720571e-002,28:5.193670e-002):2.833206e-002,33:1.405695e-001):4.970535e-002,(32:1.992525e-001,34:1.554366e-001):4.770448e-003):4.018033e-002,(37:5.975057e-002,7:2.986826e-001):1.103644e-001):1.480065e-001):5.669640e-002):5.209303e-002,((35:3.052154e-001,(36:7.533351e-002,(20:5.269071e-003,((26:1.273064e-001,21:3.860597e-002):2.681385e-001,(24:4.347322e-002,25:1.028422e-002):7.099157e-002):4.973294e-002):3.702059e-002):3.273557e-001):2.541909e-002,(22:1.914462e-002,27:2.954537e-001):6.217177e-002):8.995787e-002):3.996435e-002):4.895813e-002):6.057745e-001):2.113583e-001):5.184463e-002):3.096753e-001):1.061082e-001):7.073587e-002,17:2.475933e-001,1:1.593149e-001);

tree gen.264000 = [&U] (17:9.682125e-002,((6:8.341202e-002,16:3.848664e-002):9.868913e-002,(19:4.497175e-002,((40:3.337284e-001,(5:1.620056e-002,4:1.122871e-001):1.238791e-001):6.427601e-002,((((13:1.544464e-002,(((15:3.779315e-002,3:8.363378e-002):3.512590e-002,14:1.502009e-002):7.643622e-002,(8:7.537153e-002,(9:4.710291e-002,(11:1.022265e-001,12:3.261764e-002):2.795451e-001):1.549535e-001):1.208137e-002):7.663375e-002):1.016607e-002,10:1.080639e-001):8.783206e-002,18:1.170313e-001):8.373051e-002,((41:5.445809e-001,42:1.050222e-001):3.982040e-001,(((((36:9.113244e-002,(26:1.306491e-001,(20:1.467241e-001,(21:1.712823e-002,(24:7.294288e-002,25:9.970826e-002):1.158349e-001):4.414238e-002):5.582670e-002):2.520041e-001):2.588397e-002,35:1.995700e-001):1.736603e-001,22:5.060098e-002):1.104240e-002,((((23:1.538269e-002,((34:1.083080e-001,((33:1.298823e-001,(29:5.986865e-002,28:1.359907e-001):2.309079e-002):4.558352e-002,32:2.239826e-001):6.973329e-002):9.906215e-002,(37:5.490283e-002,7:2.744496e-001):7.878532e-002):1.214982e-002):1.440185e-002,(38:2.043712e-001,(39:1.133297e-001,2:8.085512e-002):1.216421e-001):6.769884e-002):6.045512e-002,30:2.107388e-002):3.468614e-002,27:2.656223e-001):2.639755e-002):5.446077e-003,31:2.327018e-001):1.619956e-001):7.285043e-002):1.936344e-001):1.638255e-001):3.744061e-002):9.665280e-003,1:3.164592e-001);

tree gen.265000 = [&U] ((19:7.663575e-002,17:1.023641e-001):5.775254e-002,((6:6.012661e-002,16:4.111133e-002):4.262470e-002,((40:3.574021e-001,(5:2.319888e-002,4:1.684901e-001):3.376585e-003):3.380897e-001,((9:1.524121e-002,((((11:1.544962e-001,12:1.505231e-001):2.693663e-001,13:4.325661e-002):9.300615e-002,10:1.921518e-001):2.584473e-002,(8:5.635533e-002,(14:1.604443e-002,(3:4.888105e-002,15:3.102692e-002):3.896978e-002):1.284915e-001):4.082521e-002):1.726387e-002):3.277360e-001,(18:7.373366e-002,((41:5.817201e-001,42:2.549632e-001):2.642287e-001,((22:1.542379e-002,((27:2.837371e-001,(((36:1.130273e-001,(21:1.879970e-002,((26:1.339467e-001,20:3.066171e-002):3.324435e-002,(24:9.014659e-002,25:1.330487e-002):7.585326e-002):8.359064e-003):1.141935e-001):3.936616e-002,35:2.506145e-001):8.633713e-002,31:1.804646e-001):1.016494e-001):4.509958e-002,30:4.734007e-002):3.214244e-002):3.894045e-002,((38:2.251308e-001,39:6.969112e-002):9.867376e-002,(2:7.535653e-002,(23:1.716806e-002,(((34:1.395919e-001,(33:9.601465e-002,(29:6.395156e-002,28:8.918178e-002):5.384526e-002):6.398979e-002):8.989536e-004,32:2.851918e-001):7.270139e-002,(37:8.686888e-002,7:1.870908e-001):2.235525e-002):1.449365e-001):6.205789e-002):6.433527e-002):1.794388e-001):5.377190e-001):2.849330e-001):4.633350e-002):1.131422e-001):2.649922e-001):1.908095e-001,1:2.149756e-001);

tree gen.266000 = [&U] (((((4:1.399126e-001,5:5.371220e-002):2.808451e-002,40:5.289395e-001):1.650360e-001,((9:7.449398e-002,(((11:1.312117e-001,12:1.278374e-001):2.605574e-001,13:5.797293e-002):2.707783e-002,((14:1.032679e-002,(3:5.165907e-002,15:4.139203e-004):1.507273e-001):1.003317e-001,(8:6.175851e-003,10:8.054336e-002):5.646289e-002):4.932388e-002):7.420852e-002):3.169632e-001,(18:1.032286e-001,((41:3.437928e-001,42:1.041403e-001):4.622983e-001,((((36:1.058255e-001,(((26:2.145197e-001,21:1.028527e-001):1.755054e-002,20:1.314745e-001):2.524641e-002,(24:2.735871e-002,25:2.987833e-002):1.140517e-001):9.769939e-002):1.708196e-002,35:2.685530e-001):1.283631e-001,(30:3.528449e-002,((2:4.711923e-002,((23:7.038481e-002,((32:2.448560e-001,(((28:1.841273e-001,33:5.457566e-002):2.962741e-002,29:7.219752e-002):1.546472e-001,34:1.185537e-001):1.210475e-002):6.617159e-002,(37:1.170664e-001,7:1.588940e-001):1.700932e-001):1.160197e-001):3.973038e-002,(38:1.336236e-001,39:7.230292e-002):1.012785e-001):4.610637e-002):1.405796e-001,31:1.532664e-001):1.540773e-002):1.120422e-001):8.382877e-002,(27:2.278033e-001,22:1.254495e-002):1.844674e-002):5.230374e-001):6.176825e-002):4.836160e-002):1.231632e-001):1.670602e-001,((16:1.235460e-001,19:1.685893e-002):2.297712e-002,6:2.625593e-001):3.496488e-002):7.239760e-002,17:1.290388e-001,1:1.531945e-001);

tree gen.267000 = [&U] (((19:2.499681e-001,16:9.438629e-002):1.660112e-002,6:1.489008e-001):1.511957e-001,(((40:4.926043e-001,(5:1.409043e-002,4:1.312388e-001):1.117422e-001):1.096002e-001,((((41:2.955310e-001,42:1.664866e-001):3.918072e-001,((((30:2.377754e-002,((36:9.180164e-002,((26:1.238352e-001,((24:2.012871e-002,25:4.747952e-005):1.251930e-002,20:1.062388e-001):5.450349e-002):7.125138e-002,21:9.691979e-002):4.943585e-002):1.070003e-001,35:1.781280e-001):8.228336e-002):9.462808e-004,(31:9.300501e-002,(((38:1.159160e-001,39:3.171572e-002):1.671670e-001,2:1.937471e-002):7.031572e-003,(23:3.651867e-002,((((29:6.857008e-002,(28:8.111080e-002,33:5.163896e-002):3.869304e-002):8.878202e-002,34:9.501909e-002):5.480558e-002,32:2.145721e-001):8.785425e-002,(37:1.529920e-002,7:1.377820e-001):1.533399e-001):7.025240e-002):7.310161e-002):1.366196e-001):4.059139e-002):9.258300e-002,22:6.847700e-002):3.025845e-002,27:1.875951e-001):1.418885e-001):1.041094e-001,18:2.533629e-002):6.385683e-002,((8:5.357437e-003,((11:5.675044e-003,12:7.540729e-002):1.645098e-001,13:6.357017e-002):4.271505e-002):2.931245e-002,((9:6.252977e-002,((3:6.325848e-003,15:2.500797e-003):5.115131e-002,14:1.212191e-002):1.343495e-001):2.643089e-002,10:6.107636e-002):6.662645e-002):1.017909e-001):1.040458e-001):1.548712e-001,17:1.416053e-001):6.185797e-003,1:1.359888e-001);

tree gen.268000 = [&U] (17:1.089483e-001,(19:2.401964e-001,((6:1.827725e-001,16:1.325744e-001):4.992231e-002,(((4:3.130702e-002,5:6.053120e-002):2.000479e-002,40:4.703334e-001):6.841373e-002,((((41:8.030638e-001,42:1.908682e-001):2.537449e-001,((22:6.580010e-002,(30:2.394090e-002,31:9.613594e-002):2.292118e-002):3.388781e-002,(((38:8.349147e-002,39:1.655282e-001):4.137063e-002,(2:1.156802e-001,(23:3.816648e-002,(((32:1.989096e-001,34:9.130462e-002):1.809444e-002,(29:1.067539e-001,(33:9.853624e-002,28:6.000475e-002):9.545381e-002):1.837829e-001):6.482776e-002,(37:3.822053e-002,7:1.835635e-001):4.995017e-002):3.129163e-002):7.567618e-002):2.018958e-002):7.494128e-002,(27:1.797910e-001,((36:8.820687e-002,((21:1.845651e-002,((24:4.355985e-003,25:2.935168e-003):3.610657e-002,26:1.189942e-001):2.366988e-002):5.881858e-003,20:2.645858e-002):9.433282e-002):1.004433e-001,35:1.711646e-001):3.144170e-002):3.654418e-002):1.126455e-001):1.476018e-001):1.332390e-001,18:8.236989e-002):1.194554e-001,(9:1.334634e-003,((14:7.285107e-003,(15:7.112797e-003,3:3.943755e-002):3.368139e-002):1.510731e-001,((8:2.347609e-002,((11:1.259164e-002,12:2.415088e-002):1.271334e-001,13:2.160472e-002):1.266285e-001):7.129000e-003,10:5.766676e-002):1.641499e-002):5.127430e-003):9.604209e-002):6.162094e-002):1.973534e-001):2.666891e-002):7.060687e-002,1:1.983847e-001);

tree gen.269000 = [&U] (((((40:3.272504e-001,5:9.675170e-002):3.744468e-002,4:1.130103e-001):9.361945e-002,((((41:1.061943e+000,42:8.277979e-002):4.025898e-001,(35:1.886987e-001,((((27:2.467732e-001,30:1.077227e-001):4.727649e-002,22:1.899564e-001):4.645147e-003,((36:1.203473e-001,((20:3.238268e-002,21:1.082443e-002):9.246748e-002,((24:2.831151e-002,25:1.725854e-002):4.900402e-002,26:3.598263e-002):9.264177e-002):4.580717e-002):7.843344e-002,31:9.273211e-002):8.514839e-002):2.122346e-003,((2:2.075566e-001,(39:8.043431e-002,38:1.095366e-001):4.882834e-003):5.456914e-003,(23:1.559118e-002,((34:2.153764e-001,(32:2.630309e-001,(28:7.934813e-002,(29:1.508576e-002,33:1.540775e-001):3.301066e-002):1.655449e-001):1.105097e-001):5.866550e-002,(37:5.843029e-002,7:2.352755e-001):9.870271e-002):2.209941e-002):9.931289e-002):2.001799e-001):1.361951e-002):3.536728e-001):1.485036e-001,18:5.874054e-002):2.079177e-001,(9:6.133059e-002,(8:2.778148e-002,(((14:2.010802e-002,(15:2.326494e-002,3:1.366461e-002):4.056882e-002):1.331718e-001,10:1.977821e-001):2.609327e-002,((11:1.220115e-001,12:2.882279e-002):2.379257e-001,13:1.225242e-001):5.488686e-002):1.913652e-002):1.577192e-002):1.791726e-001):1.800717e-001):2.609731e-001,17:8.847947e-002):3.991252e-002,(19:1.472432e-001,(6:5.751656e-002,16:1.164026e-001):2.996716e-002):1.556214e-002,1:2.843336e-001);

tree gen.270000 = [&U] ((((40:2.187635e-001,4:1.637410e-001):3.732203e-002,5:3.883742e-002):4.581687e-002,((((41:6.389529e-001,42:1.258460e-001):3.301292e-001,(((30:5.084246e-002,(((35:1.095713e-001,(36:1.041750e-001,((21:9.226205e-003,26:1.011894e-001):8.339295e-003,(20:1.986653e-002,(24:2.616252e-002,25:1.316933e-002):3.595403e-002):6.952118e-002):2.004475e-001):2.523638e-001):5.396181e-003,(((39:6.841293e-002,38:6.848030e-002):2.552374e-002,(23:1.284664e-002,((32:2.318442e-001,(((33:1.227606e-001,29:1.249290e-001):2.974140e-003,28:4.862498e-002):5.700778e-002,34:1.079821e-001):3.120296e-002):4.292474e-003,(37:2.222948e-001,7:1.938596e-001):4.433719e-002):3.773776e-002):1.254302e-001):6.121035e-003,2:6.684189e-002):1.501712e-001):5.620937e-003,27:2.064115e-001):1.224559e-002):1.685229e-003,22:5.606361e-003):9.073656e-002,31:1.512277e-001):2.567607e-001):4.277624e-001,18:1.587344e-001):3.799099e-002,(9:1.779002e-002,((((11:6.162111e-002,12:4.516725e-003):2.479315e-001,((14:1.338322e-002,15:8.700212e-003):9.518751e-002,3:7.300490e-002):6.228686e-002):8.913663e-002,(13:1.391993e-002,10:1.099662e-001):1.653319e-002):1.181688e-001,8:7.007425e-003):8.182561e-002):7.149671e-002):1.505996e-001):1.343928e-001,(17:1.134208e-001,(19:8.951559e-003,(16:8.090434e-002,6:6.748971e-002):6.534914e-003):1.133863e-001):4.150607e-002,1:1.780715e-001);

tree gen.271000 = [&U] ((((4:6.009752e-002,(40:2.071955e-001,5:1.348930e-003):4.614409e-002):7.645078e-002,((((41:3.603530e-001,42:1.154165e-001):2.844925e-001,((22:1.294034e-001,30:5.851395e-002):1.047634e-002,(((35:1.728721e-001,(36:9.510616e-002,(20:1.318026e-002,((24:9.756222e-003,25:3.655221e-002):4.914757e-002,(21:9.065198e-003,26:8.953728e-002):7.488067e-003):5.569694e-002):1.932261e-001):1.138968e-001):2.164147e-001,(27:7.467940e-002,31:1.418786e-001):1.129337e-001):2.178015e-002,((23:4.840148e-002,(((29:5.061964e-002,(28:3.216171e-002,33:8.687236e-002):1.947572e-002):3.425882e-002,(32:2.060457e-001,34:9.609388e-002):5.260943e-002):3.909703e-002,(37:6.906753e-002,7:1.777933e-001):1.680665e-002):4.010905e-002):1.024577e-001,(2:6.130232e-002,(39:6.487597e-002,38:1.005611e-001):7.363646e-002):2.293783e-003):4.094051e-002):1.098267e-002):2.273983e-001):1.355219e-001,18:4.515484e-002):2.347532e-002,(9:1.631566e-002,(((((15:4.471593e-002,3:9.374291e-002):9.294697e-002,14:1.678841e-002):6.659386e-002,8:4.129772e-002):6.983143e-003,10:5.459065e-002):2.470370e-002,((11:8.199607e-003,12:2.112121e-002):1.814881e-001,13:9.088443e-002):2.513698e-002):1.476185e-002):7.044387e-002):8.038672e-002):1.878548e-001,(16:6.391229e-002,6:1.125716e-001):6.552233e-002):6.855187e-002,(17:1.353456e-001,19:7.786306e-003):3.245133e-002,1:1.718089e-001);

tree gen.272000 = [&U] ((((19:3.051153e-002,16:6.475697e-002):6.067650e-003,6:1.301782e-001):7.282463e-002,(((4:7.184946e-002,5:9.807514e-002):9.473376e-002,40:2.669230e-001):7.294963e-002,((((41:6.283632e-001,42:4.825734e-002):1.953311e-001,((((27:6.629590e-002,(30:7.349686e-002,(35:1.885801e-001,(36:1.389513e-001,((24:2.485643e-002,25:2.553236e-002):5.471505e-002,(26:1.036587e-001,(20:4.087104e-003,21:9.892704e-003):2.408730e-002):9.786162e-003):2.271417e-002):9.083171e-002):2.184016e-001):1.303493e-002):5.805503e-003,22:1.453636e-001):7.261294e-003,(((23:2.261155e-002,(((32:1.804655e-001,34:5.732113e-002):6.989238e-002,((28:6.233121e-002,33:1.171770e-001):2.693666e-002,29:1.296463e-001):3.748935e-002):4.547747e-002,(37:1.145735e-001,7:2.615330e-001):1.025939e-001):1.013103e-001):5.596062e-002,38:1.162299e-001):5.748047e-002,(39:9.075276e-002,2:6.444593e-002):5.966721e-002):2.091893e-001):3.203912e-002,31:1.668357e-001):3.040625e-001):1.480762e-001,18:1.140101e-001):1.013794e-001,(9:6.321585e-002,(((15:2.296917e-002,3:3.581387e-002):2.183872e-001,14:2.302966e-002):3.871527e-002,(((11:1.443970e-001,12:5.875264e-003):2.031815e-001,13:6.566832e-003):3.108106e-002,(10:5.795811e-002,8:4.719466e-002):3.995685e-002):2.506290e-002):1.721289e-001):6.056391e-002):8.454769e-002):1.986598e-001):5.047974e-002,17:9.919557e-002,1:2.330533e-001);

tree gen.273000 = [&U] (((19:3.373089e-002,(16:6.344443e-002,6:1.022369e-001):8.532203e-002):7.103695e-002,((4:6.541797e-003,(5:2.227633e-002,40:2.919438e-001):1.466137e-002):1.156686e-001,((((41:7.021958e-001,42:6.364500e-002):4.047973e-001,((27:7.549336e-002,((35:1.161646e-001,(36:1.519763e-001,(20:6.754825e-003,(26:8.388417e-002,((24:7.748931e-003,25:2.792571e-002):1.188935e-001,21:6.089674e-002):1.346190e-002):1.058141e-001):5.667128e-002):9.501091e-002):1.635729e-002,((2:6.578920e-002,((23:1.691601e-002,((32:2.325937e-001,34:7.754928e-002):2.014349e-001,((37:6.209688e-002,7:4.075279e-001):3.481379e-002,((33:1.281609e-001,29:2.398045e-002):5.762742e-003,28:4.743888e-002):2.997593e-002):6.745075e-004):5.611324e-002):8.070758e-002,(38:1.544112e-001,39:1.351723e-001):5.364752e-002):7.529191e-002):1.815617e-001,(22:4.303288e-002,30:1.487209e-001):2.616437e-003):1.885432e-002):7.221501e-003):4.290491e-002,31:1.781286e-001):2.253408e-001):1.369786e-001,18:2.811472e-001):7.079839e-002,(9:4.055872e-002,(8:3.195321e-002,(((11:2.208394e-001,12:1.168972e-002):2.175759e-001,13:3.419260e-002):1.214961e-001,(((15:4.525316e-002,3:2.912265e-002):1.592184e-001,14:5.837284e-002):2.788162e-002,10:3.698507e-002):3.965845e-002):7.290482e-002):1.877208e-002):1.194682e-001):1.461533e-001):3.940663e-001):1.027695e-002,17:3.419744e-002,1:1.358492e-001);

tree gen.274000 = [&U] (((((40:3.763258e-001,4:3.813759e-002):1.570190e-002,5:3.299336e-002):6.677505e-002,((((41:1.062499e+000,42:8.170677e-002):3.163078e-001,(30:1.774488e-001,(((23:4.410610e-002,((37:7.904436e-002,7:5.231799e-001):1.214406e-001,(32:1.326302e-001,(((33:1.678941e-001,29:4.693083e-002):1.781257e-003,28:5.495073e-002):6.540226e-002,34:9.948965e-002):6.885027e-002):2.280224e-001):8.979025e-002):6.390041e-003,(2:8.553657e-002,(38:4.592320e-001,39:1.716672e-001):3.198012e-001):4.746282e-002):3.536871e-001,(22:2.293739e-001,((31:7.901240e-002,27:1.997409e-001):5.298534e-002,(35:2.794456e-001,(36:1.240233e-001,((24:9.338009e-003,25:3.296450e-002):1.682634e-001,((20:1.055005e-001,21:4.175092e-002):1.366101e-002,26:1.817675e-001):4.961902e-002):8.876068e-002):1.589389e-001):2.308279e-002):3.396910e-002):1.387186e-002):1.460929e-003):2.808582e-001):1.549538e-001,18:8.593225e-002):1.500599e-001,(9:1.059166e-002,((((3:9.393400e-003,15:1.180700e-001):6.789740e-002,14:6.442651e-002):3.070369e-001,(10:2.045591e-001,((11:1.166868e-001,12:3.572649e-002):4.333088e-001,13:3.020510e-002):1.723493e-001):3.596418e-002):6.888421e-002,8:1.998916e-002):1.158996e-001):1.262215e-001):1.534713e-001):2.842245e-001,17:2.472484e-001):6.179943e-002,(19:4.330335e-002,(6:1.288812e-001,16:3.061299e-002):7.403049e-004):3.241187e-002,1:1.288482e-001);

tree gen.275000 = [&U] (17:2.196197e-001,((((5:1.104033e-002,(4:1.690536e-001,40:4.200744e-001):3.589343e-002):2.395151e-001,((((41:9.369745e-001,42:1.369847e-001):3.609968e-001,((30:5.251134e-002,(31:1.391558e-001,((36:1.077689e-001,(21:2.390572e-003,(26:1.413393e-001,(20:1.921512e-001,(24:9.012469e-002,25:2.266616e-002):1.957809e-001):1.828294e-002):1.021531e-002):1.645831e-001):1.086180e-001,35:2.224810e-001):2.282486e-002):4.685500e-002):7.267751e-002,((((38:1.365531e-001,39:9.776877e-002):3.568346e-002,(2:1.400510e-001,(23:3.539849e-002,((37:6.540993e-002,7:5.968487e-001):5.156877e-002,((32:3.319684e-001,34:1.633254e-001):1.905104e-002,(28:6.211205e-002,(33:9.899959e-002,29:3.499795e-002):1.958734e-002):2.677121e-001):4.311858e-002):4.353240e-001):6.849462e-002):1.035704e-002):2.610573e-001,27:1.306139e-001):3.476950e-002,22:2.677814e-001):3.414491e-002):3.335294e-001):1.266316e-001,18:3.507217e-002):6.071082e-002,(9:6.179902e-002,((10:1.540746e-001,(((14:5.349807e-002,15:1.537904e-003):6.884268e-002,3:7.012955e-002):2.841123e-001,((11:1.608689e-001,12:4.075712e-002):3.517927e-001,13:2.099857e-002):1.429006e-001):2.461501e-002):1.332470e-001,8:1.936962e-002):1.615759e-001):1.210341e-001):1.507679e-001):2.055326e-001,(16:4.771320e-002,6:1.493865e-001):6.904595e-002):3.617960e-002,19:1.839828e-001):3.864122e-002,1:2.770166e-001);

tree gen.276000 = [&U] (((((5:2.118062e-002,4:1.640775e-001):7.702257e-002,40:4.305698e-001):1.190750e-001,((((41:8.018096e-001,42:3.450879e-001):3.317881e-001,(31:1.135279e-001,(35:1.936612e-001,(30:3.471809e-003,(22:2.316297e-002,((36:6.067514e-002,(26:1.136835e-001,((24:3.727077e-002,25:5.455986e-002):1.200126e-001,(21:3.015295e-002,20:1.062977e-001):6.403232e-002):4.514497e-002):1.543078e-001):1.073917e-001,(27:1.182616e-001,((23:2.133417e-002,((37:9.271597e-002,7:2.389489e-001):2.790582e-002,((((33:5.360400e-002,29:1.617689e-001):4.174762e-002,28:1.230223e-001):5.459802e-002,34:1.270661e-001):1.784118e-001,32:2.840797e-001):1.183468e-001):1.024844e-001):4.389170e-003,(2:5.131787e-002,(38:1.202369e-001,39:2.093122e-002):3.037346e-002):5.706155e-002):2.602431e-001):3.400211e-002):5.859723e-002):1.401828e-001):3.019353e-002):3.009626e-002):3.241782e-001):3.167298e-001,18:4.339774e-002):5.565094e-002,(9:5.288409e-002,(((10:8.458464e-002,(15:2.331625e-002,(3:1.498436e-002,14:6.877161e-003):2.198682e-002):1.676592e-001):2.047065e-001,8:1.261829e-002):6.430985e-002,((11:6.302955e-002,12:7.301075e-002):3.010443e-001,13:1.959543e-001):8.198977e-002):1.328945e-002):2.627883e-001):7.150565e-002):1.477750e-001,((6:2.548944e-001,19:6.075878e-002):3.152116e-002,16:4.297949e-002):7.487612e-002):3.316697e-003,17:1.696048e-001,1:2.828368e-001);

tree gen.277000 = [&U] (17:1.803554e-001,(((((40:4.717322e-001,5:8.257516e-002):2.639810e-002,4:6.257051e-002):1.864289e-001,((((41:8.635561e-001,42:3.716628e-001):6.601895e-001,((30:9.757740e-002,((((36:3.257881e-001,(((24:4.595261e-002,25:1.013092e-002):9.833054e-002,20:1.126453e-001):3.261731e-002,(21:6.516889e-003,26:6.618513e-002):1.429449e-002):7.317954e-002):1.131036e-001,35:1.475195e-001):4.629273e-003,22:6.520722e-002):1.226989e-003,(((((37:2.470265e-002,7:2.373893e-001):1.202585e-001,(((33:5.117765e-002,28:3.295372e-002):1.544329e-002,29:8.833107e-002):2.837208e-002,(34:1.115932e-001,32:3.027800e-001):7.973736e-003):7.168980e-002):1.272093e-001,23:2.200034e-002):2.470967e-001,2:1.860003e-001):9.210086e-003,(38:3.281995e-001,39:2.926182e-002):2.926645e-001):2.857477e-001):3.186131e-002):5.453445e-004,(31:9.188941e-002,27:1.404624e-001):9.800079e-003):2.313752e-001):1.493158e-001,18:6.603644e-002):5.907644e-002,(9:2.808826e-002,(10:1.196234e-001,(8:1.525901e-002,((14:3.768015e-003,(15:4.434474e-002,3:1.378159e-001):8.406692e-002):4.168447e-001,((11:2.116998e-001,12:5.420115e-002):1.920916e-001,13:3.226858e-002):3.800476e-002):8.167584e-002):2.284566e-002):8.381216e-002):1.758581e-001):1.999557e-001):3.549587e-001,19:9.050395e-002):2.791986e-002,(6:2.545442e-001,16:9.997408e-002):8.393585e-002):3.783733e-003,1:3.060588e-001);

tree gen.278000 = [&U] ((16:4.130045e-002,(6:1.352121e-001,19:2.417054e-001):9.754801e-003):3.367937e-002,(17:1.837245e-001,((4:1.323643e-001,(5:3.923761e-002,40:4.542798e-001):9.839552e-002):1.266153e-001,((((41:1.332097e+000,42:9.168200e-002):3.642766e-001,(((31:1.808147e-001,22:6.461182e-002):2.891000e-002,(((36:6.725001e-002,(((24:2.411488e-002,25:2.303496e-003):1.177605e-001,(20:1.493092e-002,26:2.183453e-001):3.164667e-002):3.371798e-002,21:2.041465e-001):2.257211e-001):9.541891e-002,35:9.588935e-002):1.760818e-001,27:3.958262e-001):5.414687e-002):3.862569e-002,(30:4.321439e-002,((38:2.056170e-001,39:5.049657e-002):3.033005e-001,(2:4.859036e-002,(((37:7.793133e-003,7:3.183076e-001):1.771926e-001,(34:1.098995e-001,(((28:2.622985e-001,33:5.996148e-002):4.431604e-003,29:7.700510e-002):2.874991e-002,32:3.715558e-001):1.112354e-001):7.121098e-002):1.025757e-001,23:4.390769e-003):4.228927e-002):4.588450e-002):2.814516e-001):4.419051e-002):1.845662e-001):2.517054e-001,18:2.357627e-001):3.735457e-002,(9:2.074533e-002,((8:2.737545e-003,((11:2.084868e-001,12:2.706077e-002):1.974697e-001,13:8.527920e-002):1.674139e-001):4.740074e-002,((14:4.990702e-002,(3:7.351655e-002,15:2.609824e-002):6.323034e-002):3.042471e-001,10:3.272583e-002):6.573105e-002):1.061026e-001):1.589681e-001):1.096333e-001):2.662838e-001):4.078400e-002,1:2.536659e-001);

tree gen.279000 = [&U] (((16:7.162022e-002,6:4.615854e-001):7.556662e-002,(17:2.186158e-001,19:2.778966e-001):7.525441e-002):9.197348e-004,(((5:3.675097e-002,4:3.120258e-002):1.468511e-001,40:4.332788e-001):3.820576e-002,((((41:8.582976e-001,42:8.391620e-002):7.739773e-001,((22:8.836839e-002,(27:2.931538e-001,(((((((34:1.048190e-001,32:3.525730e-001):2.071030e-002,((29:6.608109e-002,28:2.114789e-001):1.059636e-003,33:1.463886e-001):5.672167e-002):9.646205e-002,(37:3.013619e-002,7:1.826525e-001):5.857995e-002):1.531389e-001,23:2.355639e-002):8.355003e-002,((38:3.228737e-001,39:1.808316e-001):2.782057e-002,2:4.634407e-002):1.756404e-001):1.381703e-001,31:1.278003e-001):2.523339e-002,30:1.188650e-001):7.299036e-002):2.146652e-002):4.127333e-002,((36:6.248942e-002,((((24:6.514129e-002,25:5.426555e-002):9.269067e-002,20:4.865666e-002):3.506234e-002,26:1.240098e-001):9.319303e-003,21:1.112687e-001):2.207767e-001):7.743210e-002,35:1.708080e-001):1.082530e-001):4.569347e-001):4.282632e-001,18:1.447929e-001):4.601476e-002,(9:2.786789e-003,(8:2.781615e-002,((((11:1.988486e-001,12:1.150385e-001):1.396942e-001,13:1.442536e-003):2.696456e-001,((3:2.883390e-002,15:3.382612e-003):5.224072e-003,14:8.679865e-002):2.175807e-001):6.219761e-003,10:1.248027e-001):1.998588e-001):4.797951e-002):1.133445e-001):1.711283e-001):2.938222e-001,1:2.333393e-001);

tree gen.280000 = [&U] ((19:1.783936e-001,(6:3.453967e-002,16:6.637773e-002):2.168787e-002):4.589175e-001,(17:2.971078e-001,((40:2.723471e-001,(5:1.973602e-002,4:4.186118e-002):3.495644e-002):1.552641e-001,((((41:1.298333e+000,42:2.848990e-001):8.193866e-001,((27:2.074397e-001,(31:1.749089e-001,22:9.476339e-002):3.574292e-001):7.281015e-002,(((36:5.621656e-002,(((24:1.314518e-002,25:5.812168e-002):2.326249e-002,(26:2.232626e-001,21:1.029083e-001):8.505168e-002):2.110622e-001,20:7.014083e-002):1.429576e-001):2.947560e-001,35:2.281689e-001):1.262506e-001,(((38:4.264874e-001,39:3.638279e-001):1.262945e-001,(((((34:1.949860e-001,32:4.271393e-001):7.970382e-002,(29:8.732581e-002,(33:8.204387e-002,28:1.028545e-001):1.548121e-002):8.078538e-002):5.581460e-002,(37:2.294246e-002,7:2.439910e-001):4.681704e-001):1.711337e-001,23:1.540712e-002):4.836292e-002,2:5.550611e-002):5.378220e-003):1.499636e-001,30:1.516275e-001):4.567891e-002):7.534618e-002):3.975638e-001):8.630199e-002,18:8.350442e-002):6.538140e-002,(9:5.866620e-003,(8:4.876298e-002,(((11:2.656261e-001,12:2.315766e-001):2.981705e-001,13:2.490307e-002):7.706427e-002,((3:1.394815e-001,(14:1.345898e-001,15:7.575762e-002):1.682980e-002):2.176951e-001,10:4.587029e-002):5.682798e-002):8.946903e-002):6.927216e-002):2.047508e-001):4.396622e-001):3.959498e-001):2.901453e-002,1:1.844208e-001);

tree gen.281000 = [&U] (((16:5.345457e-002,(19:1.191805e-001,6:5.768043e-002):2.023156e-002):1.439793e-002,17:2.656624e-001):8.798922e-003,(((4:4.300590e-002,40:5.385025e-001):4.963245e-003,5:2.898325e-002):2.318437e-001,((18:1.288225e-001,((41:1.365531e+000,42:2.630929e-001):4.031046e-001,((22:8.389666e-002,((31:1.247796e-001,(((36:1.056051e-001,((20:1.447243e-002,21:7.165744e-003):1.547268e-001,((24:3.974922e-002,25:8.086749e-003):2.331980e-002,26:2.078677e-001):1.131048e-001):1.294958e-001):4.430603e-002,35:3.184221e-001):1.874629e-001,30:6.492535e-002):4.754597e-002):3.248598e-002,27:2.452745e-001):1.115541e-001):3.915510e-002,((2:5.074357e-002,((((32:4.252270e-001,(29:7.506201e-002,(28:5.190244e-002,33:8.698736e-002):3.686616e-002):2.619990e-001):7.992511e-003,34:8.974179e-002):1.433786e-001,(37:2.931798e-001,7:1.481019e-001):6.099644e-002):8.104271e-002,23:3.577387e-003):2.507736e-002):7.291667e-002,(39:2.920540e-001,38:1.502944e-001):3.988122e-003):3.430916e-001):3.117804e-001):4.117712e-001):1.040004e-001,(9:1.801354e-002,(8:6.040573e-002,((((11:2.073208e-001,12:1.160383e-002):1.682422e-001,13:1.849992e-001):4.578429e-003,10:1.088310e-001):8.210563e-002,(15:6.137497e-004,(14:6.303329e-002,3:3.217206e-002):1.348401e-002):3.215845e-001):5.006920e-002):4.245728e-002):3.462231e-001):1.267193e-001):4.390982e-001,1:1.502422e-001);

tree gen.282000 = [&U] ((19:1.399693e-001,17:1.594220e-001):1.006704e-002,((((5:1.796112e-003,40:2.590188e-001):4.331993e-002,4:6.070956e-002):5.029497e-002,((((41:7.296139e-001,42:2.608505e-001):4.212997e-001,((((31:8.120491e-002,((36:7.854031e-002,((26:6.844143e-002,((24:2.253404e-002,25:4.803754e-003):3.305893e-002,21:2.464027e-002):3.789016e-002):9.040526e-002,20:6.476608e-003):8.273467e-002):2.417607e-002,35:1.891516e-001):8.669082e-002):1.337164e-001,22:4.591217e-002):1.317566e-003,30:8.298882e-003):4.202914e-002,(27:2.802357e-001,(((((32:2.739912e-001,34:2.189618e-001):6.244336e-002,((28:1.056227e-001,33:8.197054e-002):1.455488e-001,29:3.822065e-002):7.524145e-002):1.114958e-001,(37:7.074266e-002,7:2.214349e-001):4.876472e-002):9.114733e-002,23:5.967255e-002):1.489665e-002,((39:2.006910e-001,38:1.085416e-001):1.847644e-001,2:2.201213e-001):3.691337e-002):1.838191e-001):3.373432e-002):2.209488e-001):2.255606e-001,18:7.652417e-002):6.265984e-002,(9:3.863939e-002,((3:1.901902e-002,(14:3.750226e-002,15:9.202342e-005):2.498625e-002):1.656979e-001,(8:3.247992e-002,(10:1.160616e-001,((11:5.049543e-002,12:2.809742e-002):1.802619e-001,13:1.483514e-001):5.301134e-002):2.930045e-002):4.863846e-002):1.089405e-001):7.522012e-002):2.019858e-001):1.253216e-001,(6:3.334389e-002,16:3.550622e-002):4.521137e-002):1.163606e-002,1:6.463194e-002);

tree gen.283000 = [&U] (((5:8.185332e-003,(40:2.874809e-001,4:4.932932e-002):4.955186e-002):3.475249e-002,((((41:6.321642e-001,42:2.689470e-001):4.474412e-001,((27:2.204573e-001,(22:2.063424e-001,((36:7.823494e-002,((21:2.420996e-002,(26:1.039959e-001,20:1.182771e-001):4.883263e-003):3.575185e-003,(24:3.476068e-003,25:7.613382e-005):7.556336e-002):8.099492e-002):1.374799e-001,35:1.884162e-001):1.945517e-002):3.867339e-002):1.228125e-001,((31:7.363655e-002,30:4.079009e-002):8.915863e-003,(((39:1.045258e-001,38:1.933149e-001):2.502829e-002,((((32:3.112822e-001,34:2.513975e-001):4.912798e-002,((29:4.777883e-002,33:1.044163e-001):5.316091e-003,28:1.262764e-001):9.268837e-002):2.240628e-001,(37:3.552388e-002,7:1.046289e-001):7.214366e-002):4.002242e-002,23:1.593328e-002):6.436337e-002):8.315827e-002,2:1.704721e-002):2.318715e-001):4.573396e-002):3.101854e-001):1.102472e-001,18:5.894483e-002):6.329863e-002,(9:8.817076e-002,(8:3.235363e-002,(10:9.979266e-002,(((3:1.056421e-002,15:3.597442e-003):5.323951e-002,14:3.309794e-002):8.455207e-002,((11:1.342835e-001,12:1.229423e-002):4.855419e-001,13:8.201369e-003):1.094109e-001):1.922517e-002):1.983323e-001):2.445275e-002):6.999132e-002):2.217078e-001):2.879823e-001,((19:1.281396e-002,17:2.155490e-001):3.807646e-002,(6:5.338684e-002,16:1.030080e-001):4.568639e-002):5.145613e-002,1:1.790438e-001);

tree gen.284000 = [&U] (((17:2.922998e-001,(6:1.355980e-001,16:1.516644e-001):1.586688e-001):3.539654e-003,(((40:3.246910e-001,5:8.833097e-002):8.462016e-002,4:9.849906e-002):1.987963e-001,((((41:9.688911e-001,42:1.373834e-001):4.165668e-001,((((36:1.322496e-001,(21:7.426653e-004,(((24:2.196086e-002,25:5.132678e-002):6.625239e-002,26:1.422146e-001):1.573462e-002,20:1.543321e-001):8.472165e-003):8.021735e-002):1.206192e-001,35:2.032949e-001):6.693994e-002,((((((((33:1.367898e-001,29:1.224376e-002):3.239644e-002,28:5.703277e-002):1.471963e-001,34:1.921011e-001):1.714768e-002,32:4.120542e-001):1.973753e-001,(37:7.918448e-002,7:1.270023e-001):9.570108e-002):5.046311e-002,23:1.452106e-002):1.240520e-001,((39:3.061045e-001,38:2.213686e-001):1.565720e-002,2:3.222764e-002):3.700968e-002):1.315624e-001,31:3.468577e-001):3.626287e-002):1.656515e-002,((22:8.135932e-002,30:9.362514e-002):1.574294e-002,27:1.870839e-001):1.957791e-002):3.939309e-001):2.024331e-001,18:2.062331e-001):7.369579e-003,(9:3.522287e-002,(8:1.276019e-002,((((11:7.217314e-002,12:1.730887e-003):6.506259e-001,13:5.769338e-002):9.782921e-002,((14:7.472318e-003,15:1.004840e-002):3.893244e-004,3:7.982743e-002):1.174379e-001):9.270810e-002,10:1.407237e-001):2.474591e-001):1.479841e-002):1.203202e-001):3.481072e-001):4.141836e-001):3.058631e-002,19:4.134508e-002,1:1.158703e-001);

tree gen.285000 = [&U] (19:3.892762e-002,(((16:6.071285e-003,6:3.393967e-001):6.492327e-002,((5:1.493757e-001,(40:5.991945e-001,4:9.100117e-002):9.086679e-003):2.233833e-001,((((41:9.047254e-001,42:2.779453e-001):3.249601e-001,(31:3.855261e-001,(((36:1.076975e-001,(((20:6.274938e-002,21:9.693828e-002):1.798115e-001,(24:1.733123e-002,25:2.744416e-002):1.264214e-001):7.825846e-002,26:1.577383e-001):1.898736e-001):2.154846e-002,35:1.898315e-001):4.505211e-002,((((((39:8.507240e-002,38:1.847671e-001):1.092932e-001,2:3.009334e-002):5.602038e-003,((((32:5.391726e-001,34:2.325301e-001):1.581584e-001,((29:1.004543e-001,33:1.314285e-001):6.194587e-002,28:1.026618e-001):6.784697e-002):5.383683e-002,(37:9.159128e-002,7:2.014285e-001):1.174584e-001):4.897603e-002,23:1.115940e-001):2.308312e-001):2.493700e-001,27:1.780971e-001):4.008406e-002,30:9.537791e-002):2.813835e-002,22:5.315352e-002):5.254865e-002):1.865023e-002):3.611083e-001):1.261882e-001,18:2.566818e-001):2.246585e-001,(9:3.082652e-002,(((((14:2.287500e-002,3:5.240172e-002):1.111005e-003,15:1.185599e-001):7.758741e-002,10:2.039952e-002):8.771932e-002,((11:7.831656e-002,12:1.724538e-001):3.268340e-001,13:5.387259e-002):6.622527e-002):3.900313e-001,8:3.616125e-002):1.158696e-001):1.697272e-001):1.575668e-001):1.494254e-001):2.100299e-002,17:3.381452e-001):1.059529e-001,1:2.268770e-001);

tree gen.286000 = [&U] (((6:3.798868e-001,19:4.763645e-003):2.541647e-003,16:8.701665e-002):2.296490e-001,(17:1.815020e-001,((4:1.721414e-001,(5:3.895461e-002,40:6.777330e-001):2.428673e-002):8.619574e-002,((((41:1.012659e+000,42:2.100409e-001):2.751988e-001,((22:8.755586e-002,27:3.966015e-001):7.117589e-002,(((30:2.031799e-002,((39:1.028404e-001,38:5.293886e-002):1.470499e-001,(((((32:5.602874e-001,34:3.872480e-001):1.128483e-001,(29:1.124385e-001,(33:4.216261e-002,28:7.360832e-002):3.789211e-002):8.523775e-002):2.231784e-002,(37:1.263418e-001,7:7.133814e-001):8.712076e-002):9.477159e-002,23:1.312023e-001):5.884441e-002,2:5.190294e-002):3.417473e-002):2.682999e-001):3.120158e-001,31:3.473352e-001):5.202971e-002,((36:2.825555e-001,(((26:2.018377e-001,21:1.263685e-002):5.419049e-003,20:6.279391e-002):3.616823e-002,(24:5.591575e-002,25:3.519601e-002):5.079548e-002):2.784438e-001):7.489673e-002,35:5.532899e-001):1.715463e-001):6.876121e-002):2.925627e-001):3.412473e-001,18:2.284794e-001):2.568996e-001,(8:4.441226e-002,((11:8.765974e-002,12:1.930276e-001):3.571401e-001,(((15:1.642761e-001,14:2.864169e-002):2.085589e-002,(3:2.101301e-002,(13:5.987878e-002,10:7.283440e-002):1.598888e-002):7.597260e-002):1.046618e-001,9:4.803004e-002):1.980992e-001):1.325307e-001):4.616758e-001):2.960264e-001):1.706339e-001):3.159662e-004,1:1.912864e-001);

tree gen.287000 = [&U] (17:3.785957e-001,((((5:2.007226e-002,4:8.123804e-002):6.513991e-003,40:5.614134e-001):7.327419e-002,((((41:1.173355e+000,42:6.509046e-001):3.906904e-001,(22:2.463445e-001,((31:2.805121e-001,30:1.948303e-002):3.288440e-002,(((2:1.641600e-001,(((((32:3.660359e-001,(33:3.568480e-002,(29:9.754894e-002,28:4.861274e-002):7.595049e-003):3.104436e-002):3.507266e-002,34:2.807406e-001):1.109152e-001,(37:5.297968e-002,7:5.838590e-001):3.754192e-001):2.676124e-001,23:3.601214e-003):3.948386e-002,(39:2.697155e-001,38:8.974188e-001):5.658345e-002):4.018776e-002):2.037389e-001,27:8.183278e-001):7.895373e-003,((36:1.872226e-001,((((24:1.696614e-001,25:1.402806e-001):2.902751e-002,26:3.131579e-001):4.627089e-003,21:1.356046e-001):2.759768e-003,20:1.887098e-001):1.310184e-001):3.332717e-001,35:4.528340e-001):8.931266e-002):8.816230e-002):8.091743e-002):2.867266e-001):4.045337e-001,18:1.089524e-001):1.281842e-001,((8:1.102229e-002,(10:5.779934e-002,(((15:2.193394e-001,(14:9.835799e-003,3:7.229645e-002):3.645558e-002):2.818891e-001,(11:9.606542e-002,12:9.294534e-002):5.582505e-001):6.295829e-002,13:5.104739e-002):1.593390e-001):3.941690e-003):6.844552e-002,9:5.527703e-002):2.557265e-001):1.595366e-001):3.497598e-001,(16:1.580216e-001,(6:2.872655e-001,19:2.873824e-001):2.894560e-002):2.875980e-002):2.115870e-004,1:3.679324e-001);

tree gen.288000 = [&U] ((17:1.294750e-001,((5:5.791266e-002,(4:1.818409e-001,40:4.787388e-001):1.072138e-001):5.215239e-002,((((41:9.492930e-001,42:1.219917e-001):5.698888e-001,(31:4.353770e-001,((30:8.970170e-002,(((((((34:2.262143e-001,32:1.009393e-001):7.796907e-002,(28:3.908831e-001,(29:8.317868e-002,33:1.064774e-001):1.807047e-002):9.183602e-002):1.202417e-001,(37:4.624181e-002,7:3.289636e-001):2.837094e-001):1.002671e-002,23:4.738684e-002):8.047276e-002,(39:1.434924e-001,38:5.809600e-001):3.364250e-001):2.633647e-001,2:1.425464e-001):2.209937e-001,(27:6.620610e-001,22:2.824408e-002):3.949714e-002):5.409650e-002):7.749936e-003,((36:1.128773e-001,(21:4.145628e-002,((24:4.890371e-002,25:3.008369e-003):1.092887e-001,(20:1.969690e-001,26:1.644522e-001):4.055418e-002):5.359443e-002):1.922527e-001):2.768221e-001,35:3.173482e-001):4.132866e-002):1.311584e-001):4.313389e-001):3.272845e-001,18:3.518068e-001):1.475620e-001,(((((13:5.207756e-001,((14:1.535987e-003,15:3.670805e-002):4.242554e-002,3:5.624884e-002):1.995355e-001):1.214204e-002,(11:1.418070e-001,12:7.519663e-002):3.972538e-001):3.483608e-002,10:8.165683e-002):1.511812e-001,8:7.846682e-002):1.216237e-001,9:4.619510e-002):2.553981e-001):1.211370e-001):4.361383e-001):3.726617e-002,(19:8.755977e-002,(16:4.530496e-002,6:1.077757e-001):3.815231e-002):4.147612e-002,1:3.470340e-001);

tree gen.289000 = [&U] ((19:1.385989e-001,(17:1.070805e-001,((40:5.233844e-001,(4:2.950433e-002,5:7.602781e-002):4.210180e-002):1.178032e-001,((((41:1.536395e+000,42:2.598228e-001):3.639135e-001,((30:2.347672e-001,(2:2.328900e-001,(((((34:1.255393e-001,(33:6.044182e-002,(29:6.952072e-002,28:3.267000e-001):2.666109e-001):3.595563e-002):7.525438e-003,32:1.725335e-001):3.043218e-002,(37:3.864889e-002,7:1.414617e-001):2.359877e-001):1.263141e-001,23:3.211590e-002):1.798446e-001,(39:1.290278e-001,38:4.855662e-001):9.954899e-002):9.836361e-003):1.890250e-001):3.699227e-002,((((36:9.948762e-002,((24:3.560564e-002,25:1.018714e-002):2.043931e-001,((21:1.896859e-002,26:2.895036e-001):2.900249e-002,20:5.972023e-002):3.550328e-003):1.627221e-001):1.305533e-001,35:2.082322e-001):9.029253e-002,(31:2.605464e-001,27:3.226457e-001):6.655360e-002):9.567162e-003,22:7.306302e-002):4.657397e-002):4.427441e-001):1.856484e-001,18:2.777180e-001):6.969170e-002,((((13:1.183326e-001,(11:1.737013e-001,12:1.579229e-001):2.605378e-001):5.310913e-002,((3:1.992717e-002,(14:2.411173e-002,15:1.472962e-002):4.827018e-002):8.650892e-002,10:2.279212e-001):9.170179e-002):2.632501e-001,8:1.686331e-001):1.177886e-001,9:2.847855e-003):2.134616e-001):2.187989e-001):2.927224e-001):4.202068e-002):7.220004e-004,(16:1.224384e-001,6:1.502604e-001):3.650011e-002,1:1.045502e-001);

tree gen.290000 = [&U] ((19:1.799749e-001,(17:1.213611e-001,(16:5.562519e-002,6:1.285907e-001):1.122913e-001):1.950945e-002):1.445531e-002,((40:6.880850e-001,(4:1.282648e-001,5:2.329104e-001):1.114236e-001):1.908690e-001,((((41:1.522025e+000,42:3.520018e-001):9.338209e-001,(31:2.006384e-001,(((36:1.347835e-001,(21:5.935535e-002,(((24:5.829896e-003,25:9.963442e-003):1.156201e-001,26:3.280858e-001):9.204455e-002,20:2.231545e-001):2.056954e-002):2.213771e-001):1.484266e-001,35:2.821081e-001):1.340334e-001,(30:5.360538e-002,((((((((34:1.240168e-001,(29:2.357834e-002,(33:7.273459e-002,28:1.410250e-001):8.087017e-002):2.189819e-001):1.629987e-001,32:1.646660e-001):2.711203e-001,(37:1.388248e-001,7:1.916490e-001):2.198559e-001):6.421885e-002,23:3.145771e-002):1.267370e-001,(39:1.475883e-001,38:2.798602e-001):2.910184e-001):2.341897e-002,2:3.306800e-001):3.896740e-001,27:1.424221e-001):7.894286e-002,22:8.164722e-002):2.463457e-002):1.622909e-001):2.484971e-002):7.474877e-001):2.057649e-001,18:8.609853e-002):6.378584e-002,(((10:1.200091e-001,(((14:2.871209e-001,3:2.013943e-002):3.979596e-002,15:4.699858e-002):1.974583e-001,(13:3.619600e-002,(11:1.149875e-001,12:1.393413e-002):4.506020e-001):8.327741e-002):1.469742e-002):8.611636e-002,8:3.291706e-002):2.590560e-001,9:1.218603e-001):2.752769e-001):4.947535e-001):4.051638e-001,1:3.003719e-001);

tree gen.291000 = [&U] ((17:2.114077e-001,(((4:2.390582e-001,5:1.132252e-001):2.883960e-001,40:4.214131e-001):1.237582e-001,(((41:1.353712e+000,42:4.622918e-001):7.520108e-001,(((22:4.557312e-002,(((((34:1.033952e-001,(((28:2.086467e-001,33:7.658724e-002):3.639575e-002,29:8.449970e-002):2.654604e-001,32:4.933719e-001):9.211283e-003):5.163019e-002,(37:4.306763e-002,7:1.704555e-001):7.903993e-002):1.073468e-001,23:2.797896e-002):1.061321e-001,(39:2.147915e-001,38:3.210132e-001):2.044709e-001):9.045051e-002,2:5.575107e-002):1.493890e-001):1.045312e-002,(27:1.264346e-001,(31:1.875769e-001,((36:2.376276e-001,(26:1.241647e-001,((25:1.113564e-002,24:2.999040e-003):9.573665e-002,(21:1.432561e-002,20:1.484001e-002):1.244828e-002):4.107291e-002):2.820512e-001):1.296416e-001,35:2.668532e-001):1.013091e-001):6.842992e-002):1.278822e-002):1.092981e-002,30:4.641590e-002):6.383747e-001):1.975420e-001,(18:7.772571e-002,(10:1.067379e-001,(8:3.064338e-002,((13:1.902274e-002,(11:1.022717e-001,12:5.727782e-003):5.420263e-001):9.000302e-002,(((14:5.236701e-002,15:4.150983e-003):2.145053e-001,3:3.531843e-002):3.255291e-001,9:5.278593e-002):2.964210e-002):3.035241e-002):5.056231e-002):7.571741e-002):3.066684e-001):2.558857e-001):1.659137e-001):1.933450e-002,(6:9.443624e-002,(19:1.600724e-001,16:8.364140e-002):2.713559e-002):1.194729e-002,1:2.526926e-001);

tree gen.292000 = [&U] ((6:6.471738e-002,16:3.255412e-002):1.304533e-003,((17:2.550564e-001,19:2.163668e-001):1.798508e-003,((5:1.136618e-002,(40:4.606970e-001,4:7.562895e-002):5.918433e-002):1.127788e-001,((((((8:3.033506e-002,(13:1.800251e-004,(11:2.010963e-001,12:2.623981e-002):1.831467e-001):1.134660e-001):1.315372e-001,((14:3.098792e-003,15:9.936047e-002):2.097861e-002,3:1.991631e-002):3.057880e-001):2.716071e-004,10:1.733220e-001):2.348734e-001,9:2.588615e-002):1.523996e-001,18:2.337217e-001):2.130354e-001,((41:1.393372e+000,42:1.889668e-001):6.534372e-001,(((((((39:3.310445e-002,38:2.153681e-001):3.004615e-001,2:8.850546e-002):4.929628e-002,((((((28:2.261298e-001,29:1.120399e-001):3.583291e-002,33:1.888956e-001):1.270613e-001,34:1.225457e-001):9.891776e-002,32:4.797637e-001):2.536085e-002,(37:1.418213e-003,7:2.040144e-001):4.509365e-002):1.006502e-001,23:2.161106e-002):2.460438e-002):8.604344e-002,(22:1.799535e-001,30:7.900129e-002):8.608781e-003):2.549972e-001,31:1.813594e-001):1.684053e-002,35:3.884206e-001):1.235468e-002,(27:3.216153e-001,(36:1.028620e-001,(((21:7.596672e-002,20:1.647944e-001):1.414355e-001,26:1.409081e-001):4.800914e-002,(25:2.803417e-002,24:3.136639e-003):1.129637e-001):8.507590e-002):2.665723e-001):1.048294e-002):3.812851e-001):2.208516e-001):9.379029e-002):1.747645e-001):1.599991e-001,1:3.465265e-001);

tree gen.293000 = [&U] (17:3.057856e-001,((((40:5.387957e-001,5:6.069871e-002):1.013666e-003,4:1.805230e-001):7.442177e-002,((((((13:3.004372e-002,(11:1.493705e-001,12:1.532540e-001):3.858825e-001):3.592670e-002,10:1.973119e-001):3.645231e-002,((15:1.793076e-002,14:3.094532e-003):1.162371e-001,3:1.018771e-001):3.192511e-001):3.389597e-003,8:1.831267e-003):3.920921e-001,9:7.640149e-002):1.209108e-001,(18:2.943553e-001,((41:1.646032e+000,42:2.039098e-001):7.442304e-001,(((27:2.865833e-001,(((22:1.791287e-002,(36:5.698081e-002,((20:1.340022e-002,21:3.009339e-002):6.889868e-003,(26:1.862223e-001,(25:8.938219e-002,24:1.233448e-002):1.913652e-001):6.125342e-003):4.203029e-001):2.499185e-001):1.927967e-002,((39:1.406358e-001,38:4.240879e-001):1.623492e-002,((((((33:1.954672e-001,28:3.106835e-001):4.828787e-002,29:3.916484e-002):2.418161e-001,(34:1.774082e-001,32:5.989497e-001):1.164002e-001):6.368079e-002,(37:1.572002e-001,7:7.242696e-001):1.928095e-001):1.708610e-002,23:3.769231e-002):1.127354e-001,2:6.328718e-002):5.282738e-002):2.625226e-001):8.843320e-003,30:7.477060e-002):4.386316e-004):2.554758e-002,35:4.610908e-001):5.880715e-002,31:1.497502e-001):3.353963e-001):3.464171e-001):6.522188e-002):1.062249e-001):2.827836e-001,((6:5.704836e-002,19:2.558023e-001):2.371312e-001,16:3.904806e-002):6.348412e-002):2.000807e-001,1:5.940276e-001);
[truncated: 19,961,699 more chars]
