# Supplementary material for: Underestimated diversity in high elevations of a global biodiversity hotspot: two new endemic species of Aethionema (Brassicaceae) from the alpine zone of Iran
Source: Front Plant Sci. 2023 May 26;14:1182073. doi: 10.3389/fpls.2023.1182073 (PMC10250747; doi:10.3389/fpls.2023.1182073)
Supplement: Supplementary file 2 [file DataSheet_2.zip › Date Sheet 2/ITS/Aethionema_ITS_ML_IQ-TREE_output.docx]

IQ-TREE 1.6.12 built Aug 15 2019

Input file name: ITS_Jalil_16_1_2023.fasta

Type of analysis: ModelFinder + tree reconstruction + ultrafast bootstrap (1000 replicates)

Random seed number: 765437

REFERENCES

----------

To cite ModelFinder please use:

Subha Kalyaanamoorthy, Bui Quang Minh, Thomas KF Wong, Arndt von Haeseler,

and Lars S Jermiin (2017) ModelFinder: Fast model selection for

accurate phylogenetic estimates. Nature Methods, 14:587–589.

https://doi.org/10.1038/nmeth.4285

To cite IQ-TREE please use:

Lam-Tung Nguyen, Heiko A. Schmidt, Arndt von Haeseler, and Bui Quang Minh

(2015) IQ-TREE: A fast and effective stochastic algorithm for estimating

maximum likelihood phylogenies. Mol Biol Evol, 32:268-274.

https://doi.org/10.1093/molbev/msu300

Since you used ultrafast bootstrap (UFBoot) please also cite:

Diep Thi Hoang, Olga Chernomor, Arndt von Haeseler, Bui Quang Minh,

and Le Sy Vinh (2017) UFBoot2: Improving the ultrafast bootstrap

approximation. Mol Biol Evol, in press.

https://doi.org/10.1093/molbev/msx281

SEQUENCE ALIGNMENT

------------------

Input data: 49 sequences with 629 nucleotide sites

Number of constant sites: 446 (= 70.9062% of all sites)

Number of invariant (constant or ambiguous constant) sites: 446 (= 70.9062% of all sites)

Number of parsimony informative sites: 105

Number of distinct site patterns: 195

ModelFinder

-----------

Best-fit model according to BIC: TIM3e+G4

List of models sorted by BIC scores:

Model LogL AIC w-AIC AICc w-AICc BIC w-BIC

TIM3e+G4 -2766.7078 5731.4155 + 0.4361 5768.8446 + 0.5915 6171.3845 + 0.8596

TIM3e+I+G4 -2766.0862 5732.1724 + 0.2987 5770.4300 + 0.2677 6176.5855 + 0.0638

TNe+G4 -2772.6058 5741.2117 - 0.0033 5777.8230 - 0.0066 6176.7365 + 0.0592

TIM2e+G4 -2771.8080 5741.6161 - 0.0027 5779.0452 - 0.0036 6181.5851 - 0.0052

TNe+I+G4 -2772.0349 5742.0699 - 0.0021 5779.4990 - 0.0029 6182.0388 - 0.0042

SYM+G4 -2765.8683 5733.7367 + 0.1366 5772.8335 + 0.0805 6182.5939 - 0.0032

TIMe+G4 -2772.5388 5743.0776 - 0.0013 5780.5068 - 0.0017 6183.0466 - 0.0025

TIM3e+I -2773.7167 5745.4334 - 0.0004 5782.8625 - 0.0005 6185.4024 - 0.0008

K2P+G4 -2780.6203 5755.2405 - 0.0000 5791.0447 - 0.0000 6186.3213 - 0.0005

TIM2e+I+G4 -2771.2098 5742.4196 - 0.0018 5780.6772 - 0.0016 6186.8328 - 0.0004

SYM+I+G4 -2765.2303 5734.4606 + 0.0951 5774.4073 - 0.0366 6187.7620 - 0.0002

TIMe+I+G4 -2771.9466 5743.8931 - 0.0009 5782.1507 - 0.0008 6188.3062 - 0.0002

TNe+I -2779.4928 5754.9856 - 0.0000 5791.5969 - 0.0000 6190.5105 - 0.0001

K2P+I+G4 -2779.7464 5755.4929 - 0.0000 5792.1042 - 0.0000 6191.0177 - 0.0000

TVMe+G4 -2773.9080 5747.8161 - 0.0001 5786.0736 - 0.0001 6192.2292 - 0.0000

K3P+G4 -2780.5538 5757.1077 - 0.0000 5793.7190 - 0.0000 6192.6326 - 0.0000

TIM3+F+G4 -2767.6691 5739.3382 - 0.0083 5779.2850 - 0.0032 6192.6396 - 0.0000

TIM2e+I -2778.8478 5755.6956 - 0.0000 5793.1247 - 0.0000 6195.6646 - 0.0000

TIMe+I -2779.4329 5756.8658 - 0.0000 5794.2949 - 0.0000 6196.8348 - 0.0000

TVMe+I+G4 -2772.9994 5747.9988 - 0.0001 5787.0955 - 0.0001 6196.8560 - 0.0000

SYM+I -2773.0488 5748.0976 - 0.0001 5787.1943 - 0.0001 6196.9548 - 0.0000

K3P+I+G4 -2779.6695 5757.3389 - 0.0000 5794.7680 - 0.0000 6197.3079 - 0.0000

TIM3+F+I+G4 -2767.0325 5740.0649 - 0.0058 5780.8725 - 0.0014 6197.8104 - 0.0000

K2P+I -2787.2322 5768.4645 - 0.0000 5804.2686 - 0.0000 6199.5452 - 0.0000

TN+F+G4 -2774.6617 5751.3234 - 0.0000 5790.4202 - 0.0000 6200.1807 - 0.0000

TPM3+F+G4 -2775.3532 5752.7064 - 0.0000 5791.8032 - 0.0000 6201.5636 - 0.0000

TPM3u+F+G4 -2775.3533 5752.7065 - 0.0000 5791.8033 - 0.0000 6201.5638 - 0.0000

GTR+F+G4 -2766.4477 5740.8954 - 0.0038 5782.5748 - 0.0006 6203.0851 - 0.0000

TIM2+F+G4 -2773.4830 5750.9660 - 0.0000 5790.9127 - 0.0000 6204.2673 - 0.0000

TN+F+I+G4 -2774.0893 5752.1786 - 0.0000 5792.1254 - 0.0000 6205.4800 - 0.0000

TVMe+I -2780.6772 5761.3544 - 0.0000 5799.6120 - 0.0000 6205.7676 - 0.0000

K3P+I -2787.1726 5770.3451 - 0.0000 5806.9564 - 0.0000 6205.8700 - 0.0000

TPM3u+F+I+G4 -2774.4805 5752.9610 - 0.0000 5792.9078 - 0.0000 6206.2624 - 0.0000

TPM3+F+I+G4 -2774.4815 5752.9630 - 0.0000 5792.9098 - 0.0000 6206.2644 - 0.0000

TIM+F+G4 -2774.6109 5753.2217 - 0.0000 5793.1685 - 0.0000 6206.5231 - 0.0000

TIM3+F+I -2774.8336 5753.6672 - 0.0000 5793.6140 - 0.0000 6206.9686 - 0.0000

GTR+F+I+G4 -2765.7932 5741.5864 - 0.0027 5784.1485 - 0.0003 6208.2202 - 0.0000

HKY+F+G4 -2782.4408 5764.8815 - 0.0000 5803.1391 - 0.0000 6209.2947 - 0.0000

TIM2+F+I+G4 -2772.8820 5751.7639 - 0.0000 5792.5715 - 0.0000 6209.5094 - 0.0000

TIM+F+I+G4 -2774.0179 5754.0357 - 0.0000 5794.8433 - 0.0000 6211.7812 - 0.0000

TVM+F+G4 -2774.1512 5754.3024 - 0.0000 5795.1100 - 0.0000 6212.0479 - 0.0000

TPM2+F+G4 -2781.2778 5764.5555 - 0.0000 5803.6523 - 0.0000 6213.4128 - 0.0000

TPM2u+F+G4 -2781.2778 5764.5555 - 0.0000 5803.6523 - 0.0000 6213.4128 - 0.0000

HKY+F+I+G4 -2781.5712 5765.1425 - 0.0000 5804.2392 - 0.0000 6213.9997 - 0.0000

TN+F+I -2781.6636 5765.3272 - 0.0000 5804.4240 - 0.0000 6214.1845 - 0.0000

TPM3+F+I -2782.0819 5766.1638 - 0.0000 5805.2606 - 0.0000 6215.0211 - 0.0000

TPM3u+F+I -2782.0820 5766.1639 - 0.0000 5805.2607 - 0.0000 6215.0212 - 0.0000

K3Pu+F+G4 -2782.3912 5766.7825 - 0.0000 5805.8793 - 0.0000 6215.6397 - 0.0000

TVM+F+I+G4 -2773.2310 5754.4619 - 0.0000 5796.1413 - 0.0000 6216.6516 - 0.0000

GTR+F+I -2773.8661 5755.7323 - 0.0000 5797.4117 - 0.0000 6217.9219 - 0.0000

TPM2u+F+I+G4 -2780.3699 5764.7398 - 0.0000 5804.6866 - 0.0000 6218.0412 - 0.0000

TPM2+F+I+G4 -2780.3713 5764.7425 - 0.0000 5804.6893 - 0.0000 6218.0439 - 0.0000

TIM2+F+I -2780.7146 5765.4292 - 0.0000 5805.3760 - 0.0000 6218.7306 - 0.0000

K3Pu+F+I+G4 -2781.5110 5767.0219 - 0.0000 5806.9687 - 0.0000 6220.3233 - 0.0000

TIM+F+I -2781.6169 5767.2338 - 0.0000 5807.1806 - 0.0000 6220.5352 - 0.0000

HKY+F+I -2789.0979 5778.1958 - 0.0000 5816.4534 - 0.0000 6222.6089 - 0.0000

TVM+F+I -2781.1069 5768.2138 - 0.0000 5809.0215 - 0.0000 6225.9594 - 0.0000

TPM2+F+I -2788.1433 5778.2867 - 0.0000 5817.3834 - 0.0000 6227.1439 - 0.0000

TPM2u+F+I -2788.1433 5778.2867 - 0.0000 5817.3835 - 0.0000 6227.1440 - 0.0000

K3Pu+F+I -2789.0521 5780.1041 - 0.0000 5819.2009 - 0.0000 6228.9614 - 0.0000

TIM3e -2828.0397 5852.0794 - 0.0000 5888.6908 - 0.0000 6287.6043 - 0.0000

TNe -2832.5112 5859.0225 - 0.0000 5894.8266 - 0.0000 6290.1032 - 0.0000

TIM2e -2831.8763 5859.7526 - 0.0000 5896.3640 - 0.0000 6295.2775 - 0.0000

TIMe -2832.4505 5860.9010 - 0.0000 5897.5123 - 0.0000 6296.4258 - 0.0000

SYM -2827.3915 5854.7830 - 0.0000 5893.0406 - 0.0000 6299.1962 - 0.0000

K2P -2843.7050 5879.4101 - 0.0000 5914.4176 - 0.0000 6306.0467 - 0.0000

TIM3+F -2829.6826 5861.3653 - 0.0000 5900.4621 - 0.0000 6310.2225 - 0.0000

K3P -2843.6440 5881.2880 - 0.0000 5917.0921 - 0.0000 6312.3687 - 0.0000

TN+F -2834.8901 5869.7802 - 0.0000 5908.0378 - 0.0000 6314.1933 - 0.0000

TVMe -2838.5693 5875.1386 - 0.0000 5912.5677 - 0.0000 6315.1076 - 0.0000

TIM2+F -2834.0068 5870.0135 - 0.0000 5909.1103 - 0.0000 6318.8708 - 0.0000

TIM+F -2834.8379 5871.6757 - 0.0000 5910.7725 - 0.0000 6320.5330 - 0.0000

GTR+F -2828.7887 5863.5774 - 0.0000 5904.3851 - 0.0000 6321.3230 - 0.0000

TPM3u+F -2840.6385 5881.2769 - 0.0000 5919.5345 - 0.0000 6325.6901 - 0.0000

TPM3+F -2840.6385 5881.2770 - 0.0000 5919.5346 - 0.0000 6325.6901 - 0.0000

HKY+F -2845.8634 5889.7269 - 0.0000 5927.1560 - 0.0000 6329.6959 - 0.0000

JC+G4 -2855.8234 5903.6468 - 0.0000 5938.6543 - 0.0000 6330.2834 - 0.0000

TPM2u+F -2844.9833 5889.9666 - 0.0000 5928.2242 - 0.0000 6334.3798 - 0.0000

TPM2+F -2844.9837 5889.9673 - 0.0000 5928.2249 - 0.0000 6334.3804 - 0.0000

JC+I+G4 -2855.0813 5904.1625 - 0.0000 5939.9667 - 0.0000 6335.2433 - 0.0000

K3Pu+F -2845.8109 5891.6217 - 0.0000 5929.8793 - 0.0000 6336.0349 - 0.0000

TVM+F -2839.7471 5883.4943 - 0.0000 5923.4410 - 0.0000 6336.7957 - 0.0000

JC+I -2862.2221 5916.4442 - 0.0000 5951.4518 - 0.0000 6343.0808 - 0.0000

F81+F+G4 -2858.0518 5914.1037 - 0.0000 5951.5328 - 0.0000 6354.0727 - 0.0000

F81+F+I+G4 -2857.3107 5914.6215 - 0.0000 5952.8791 - 0.0000 6359.0346 - 0.0000

F81+F+I -2864.4687 5926.9374 - 0.0000 5964.3665 - 0.0000 6366.9064 - 0.0000

JC -2917.5851 6025.1702 - 0.0000 6059.3916 - 0.0000 6447.3627 - 0.0000

F81+F -2920.0010 6036.0020 - 0.0000 6072.6133 - 0.0000 6471.5269 - 0.0000

AIC, w-AIC : Akaike information criterion scores and weights.

AICc, w-AICc : Corrected AIC scores and weights.

BIC, w-BIC : Bayesian information criterion scores and weights.

Plus signs denote the 95% confidence sets.

Minus signs denote significant exclusion.

SUBSTITUTION PROCESS

--------------------

Model of substitution: TIM3e+G4

Rate parameter R:

A-C: 0.4506

A-G: 4.0429

A-T: 1.0000

C-G: 0.4506

C-T: 1.9857

G-T: 1.0000

State frequencies: (equal frequencies)

Rate matrix Q:

A -1.23 0.1009 0.9055 0.224

C 0.1009 -0.6466 0.1009 0.4447

G 0.9055 0.1009 -1.23 0.224

T 0.224 0.4447 0.224 -0.8927

Model of rate heterogeneity: Gamma with 4 categories

Gamma shape alpha: 0.3795

Category Relative_rate Proportion

1 0.01387 0.25

2 0.1663 0.25

3 0.7081 0.25

4 3.112 0.25

Relative rates are computed as MEAN of the portion of the Gamma distribution falling in the category.

MAXIMUM LIKELIHOOD TREE

-----------------------

Log-likelihood of the tree: -2764.1871 (s.e. 124.9620)

Unconstrained log-likelihood (without tree): -2147.8650

Number of free parameters (#branches + #model parameters): 99

Akaike information criterion (AIC) score: 5726.3742

Corrected Akaike information criterion (AICc) score: 5763.8033

Bayesian information criterion (BIC) score: 6166.3432

Total tree length (sum of branch lengths): 0.5567

Sum of internal branch lengths: 0.1965 (35.3013% of tree length)

WARNING: 12 near-zero internal branches (<0.0016) should be treated with caution

Such branches are denoted by '**' in the figure below

NOTE: Tree is UNROOTED although outgroup taxon 'HM1454_Aethionema_S1865' is drawn at root

Numbers in parentheses are SH-aLRT support (%) / ultrafast bootstrap support (%)

+--HM1454_Aethionema_S1865

|

| +--Umbellatum_Archibold_J1

+--| (78.3/98)

| | +**Shirkuh_J2

| | +--| (90.9/99)

| | | +**HM1452_yazd_S1863

| +--| (79.6/97)

| +--W_0184833_Aethionema_ITS

|

| +--S658_Sp_nova_HM478

+**| (0/67)

| +---acarii_37

| +--| (75.1/98)

| | +**turcicum_1

| +--| (83.8/89)

| | | +---cordatum_6

| | +--| (73.3/95)

| | +--dumanii_9

| +**| (0/40)

| | +----------retsina_38

| +--| (81.3/49)

| | | +--arabicum

| | | +--| (87.7/97)

| | | | +--froedinii_11

| | +--| (96.4/98)

| | | +--carneum

| | +--| (37.5/69)

| | +---heterocarpum_15

| +--| (75.9/92)

| | | +--alanyae_39

| | | +--| (78.4/93)

| | | | +--subulatum_20

| | | +--| (74.8/69)

| | | | | +--demirizii_40

| | | | | +--| (76.5/85)

| | | | | | +-----glaucinum_41

| | | | +**| (0/50)

| | | | +--schistosum_21

| | | +--| (74.6/77)

| | | | +---karamanicum_43

| | | +--| (90.7/95)

| | | | +------armenum

| | | +--| (82.2/83)

| | | | +---spicatum_35

| | | +---| (54.8/84)

| | | | | +**capitatumi_4

| | | | | +--| (93.5/99)

| | | | | | +**huber-morathii_42

| | | | | +**| (0/40)

| | | | | | | +--elongatum_12

| | | | | | | +--| (82.8/99)

| | | | | | | | +**virgatum_26

| | | | | | +----| (99.6/100)

| | | | | | +**Szowitsii_51

| | | | | +**| (0/50)

| | | | | | | +--edentulum_53

| | | | | | | +**| (0/45)

| | | | | | | | +--grandiflorum_14

| | | | | | | +--| (86.1/91)

| | | | | | | | +--membranaceum_33Copen

| | | | | | | +--| (93.6/94)

| | | | | | | | +---umbellatum_32

| | | | | | +**| (0/24)

| | | | | | +-----eunomioides_10

| | | | | +--| (72.2/84)

| | | | | | | +--sintenisii_48

| | | | | | +--| (81.4/81)

| | | | | | +--stenopterum_31

| | | | | +----| (97.1/99)

| | | | | | +--------erinaceum

| | | | +--| (75.3/91)

| | | | | +**coridifolium_7

| | | | +--| (95.6/100)

| | | | +**thomasianum_54

| | | +---------| (98.5/99)

| | | | | +------lepidioides_18

| | | | +-------------------------------| (100/100)

| | | | +-------------spinosum_23

| | | +**| (0/9)

| | | | | +---munzurense_46

| | | | | +--| (80.7/64)

| | | | | | +---stylosum_19

| | | | | +**| (0/40)

| | | | | | +-------semnanense_50

| | | | +--| (75/45)

| | | | | +**rhodopaeum_24

| | | | +--| (97.2/100)

| | | | +**syriacum_27

| | +**| (0/17)

| | | +---fimbriatum_44

| | | +**| (52.4/76)

| | | | +----Lycium_13

| | +**| (0/52)

| | +------orbiculatum_33

+--| (77/92)

+---saxatile_34

Tree in newick format:

(HM1454_Aethionema_S1865:0.0016862456,(Umbellatum_Archibold_J1:0.0016916181,((Shirkuh_J2:0.0000026877,HM1452_yazd_S1863:0.0000023284)90.9/99:0.0034090014,W_0184833_Aethionema_ITS:0.0016867830)79.6/97:0.0016970005)78.3/98:0.0016863328,(S658_Sp_nova_HM478:0.0072131705,((((((acarii_37:0.0103428628,turcicum_1:0.0000020806)75.1/98:0.0016830260,(cordatum_6:0.0085412231,dumanii_9:0.0068596810)73.3/95:0.0016960048)83.8/89:0.0051270554,retsina_38:0.0241262774)0/40:0.0000020740,((arabicum:0.0051359161,froedinii_11:0.0016790089)87.7/97:0.0037630515,(carneum:0.0054421759,heterocarpum_15:0.0101202990)37.5/69:0.0017249857)96.4/98:0.0082696847)81.3/49:0.0017678694,(((((((((alanyae_39:0.0017065275,subulatum_20:0.0017160466)78.4/93:0.0017028905,((demirizii_40:0.0034153592,glaucinum_41:0.0139861207)76.5/85:0.0017273309,schistosum_21:0.0034281685)0/50:0.0000021895)74.8/69:0.0016598944,karamanicum_43:0.0087411383)74.6/77:0.0018090504,armenum:0.0148391200)90.7/95:0.0065188479,spicatum_35:0.0091471980)82.2/83:0.0031058440,((((((capitatumi_4:0.0000022857,huber-morathii_42:0.0000022486)93.5/99:0.0051489181,((elongatum_12:0.0017053760,virgatum_26:0.0000026826)82.8/99:0.0017066221,Szowitsii_51:0.0000022566)99.6/100:0.0121592956)0/40:0.0000022457,((((edentulum_53:0.0034446018,grandiflorum_14:0.0017177550)0/45:0.0000025532,membranaceum_33Copen:0.0034344087)86.1/91:0.0033123046,umbellatum_32:0.0088491278)93.6/94:0.0070932227,eunomioides_10:0.0139367018)0/24:0.0000024809)0/50:0.0000020966,(sintenisii_48:0.0069698969,stenopterum_31:0.0051822116)81.4/81:0.0034756050)72.2/84:0.0020050832,erinaceum:0.0194163269)97.1/99:0.0106174144,(coridifolium_7:0.0000025532,thomasianum_54:0.0000021046)95.6/100:0.0083858710)75.3/91:0.0020259362)54.8/84:0.0084960087,(lepidioides_18:0.0151634412,spinosum_23:0.0305835003)100/100:0.0692723310)98.5/99:0.0228551951,(((munzurense_46:0.0099460518,stylosum_19:0.0097070160)80.7/64:0.0028364215,semnanense_50:0.0174318221)0/40:0.0000026060,(rhodopaeum_24:0.0000026552,syriacum_27:0.0000020450)97.2/100:0.0071501179)75/45:0.0017346031)0/9:0.0000026728,((fimbriatum_44:0.0104209661,Lycium_13:0.0121792880)52.4/76:0.0015888255,orbiculatum_33:0.0157923938)0/52:0.0000022780)0/17:0.0000020281)75.9/92:0.0017144832,saxatile_34:0.0086631492)77/92:0.0016599182)0/67:0.0000023701);

CONSENSUS TREE

--------------

Consensus tree is constructed from 1000bootstrap trees

Log-likelihood of consensus tree: -2764.189537

Robinson-Foulds distance between ML tree and consensus tree: 2

Branches with support >0.000000% are kept (extended consensus)

Branch lengths are optimized by maximum likelihood on original alignment

Numbers in parentheses are bootstrap supports (%)

+--HM1454_Aethionema_S1865

|

| +--Umbellatum_Archibold_J1

+--| (98)

| | +--Shirkuh_J2

| | +--| (99)

| | | +--HM1452_yazd_S1863

| +--| (97)

| +--W_0184833_Aethionema_ITS

|

| +--S658_Sp_nova_HM478

+--| (67)

| +---acarii_37

| +--| (98)

| | +--turcicum_1

| +--| (89)

| | | +---cordatum_6

| | +--| (95)

| | +--dumanii_9

| +--| (40)

| | +----------retsina_38

| +--| (49)

| | | +--arabicum

| | | +--| (97)

| | | | +--froedinii_11

| | +--| (98)

| | | +--carneum

| | +--| (69)

| | +---heterocarpum_15

| +--| (92)

| | | +--alanyae_39

| | | +--| (93)

| | | | +--subulatum_20

| | | +--| (69)

| | | | | +--demirizii_40

| | | | | +--| (85)

| | | | | | +-----glaucinum_41

| | | | +--| (50)

| | | | +--schistosum_21

| | | +--| (77)

| | | | +---karamanicum_43

| | | +--| (95)

| | | | +------armenum

| | | +--| (83)

| | | | +---spicatum_35

| | | +---| (84)

| | | | | +--capitatumi_4

| | | | | +--| (99)

| | | | | | +--huber-morathii_42

| | | | | +--| (40)

| | | | | | | +--elongatum_12

| | | | | | | +--| (99)

| | | | | | | | +--virgatum_26

| | | | | | +----| (100)

| | | | | | +--Szowitsii_51

| | | | | +--| (50)

| | | | | | | +--edentulum_53

| | | | | | | +--| (45)

| | | | | | | | +--grandiflorum_14

| | | | | | | +--| (91)

| | | | | | | | +--membranaceum_33Copen

| | | | | | | +--| (94)

| | | | | | | | +---umbellatum_32

| | | | | | +--| (24)

| | | | | | +-----eunomioides_10

| | | | | +--| (84)

| | | | | | | +--sintenisii_48

| | | | | | +--| (81)

| | | | | | +--stenopterum_31

| | | | | +----| (99)

| | | | | | +--------erinaceum

| | | | +--| (91)

| | | | | +--coridifolium_7

| | | | +--| (100)

| | | | +--thomasianum_54

| | | +---------| (99)

| | | | | +------lepidioides_18

| | | | +-------------------------------| (100)

| | | | +-------------spinosum_23

| | +--| (17)

| | | +---fimbriatum_44

| | | +--| (76)

| | | | +----Lycium_13

| | | +--| (52)

| | | | +------orbiculatum_33

| | +--| (12)

| | | +---munzurense_46

| | | +--| (64)

| | | | +---stylosum_19

| | | +--| (40)

| | | | +-------semnanense_50

| | +--| (45)

| | | +--rhodopaeum_24

| | +--| (100)

| | +--syriacum_27

+--| (92)

+---saxatile_34

Consensus tree in newick format:

(HM1454_Aethionema_S1865:0.0016856589,(Umbellatum_Archibold_J1:0.0016910263,((Shirkuh_J2:0.0000025532,HM1452_yazd_S1863:0.0000025532)99:0.0034090571,W_0184833_Aethionema_ITS:0.0016862002)97:0.0016964311)98:0.0016857536,(S658_Sp_nova_HM478:0.0072133411,((((((acarii_37:0.0103428607,turcicum_1:0.0000025532)98:0.0016823708,(cordatum_6:0.0085404377,dumanii_9:0.0068599201)95:0.0016956092)89:0.0051272380,retsina_38:0.0241255564)40:0.0000021679,((arabicum:0.0051362158,froedinii_11:0.0016791113)97:0.0037632583,(carneum:0.0054423594,heterocarpum_15:0.0101202722)69:0.0017252029)98:0.0082688099)49:0.0017680992,((((((((alanyae_39:0.0017067478,subulatum_20:0.0017162659)93:0.0017030671,((demirizii_40:0.0034145933,glaucinum_41:0.0139859235)85:0.0017274040,schistosum_21:0.0034274015)50:0.0000025532)69:0.0016601230,karamanicum_43:0.0087410095)77:0.0018092773,armenum:0.0148390084)95:0.0065183310,spicatum_35:0.0091477465)83:0.0031059781,((((((capitatumi_4:0.0000025532,huber-morathii_42:0.0000025532)99:0.0051489598,((elongatum_12:0.0017058315,virgatum_26:0.0000025532)99:0.0017070777,Szowitsii_51:0.0000025532)100:0.0121593701)40:0.0000025532,((((edentulum_53:0.0034446705,grandiflorum_14:0.0017171818)45:0.0000025532,membranaceum_33Copen:0.0034344754)91:0.0033115094,umbellatum_32:0.0088492758)94:0.0070933537,eunomioides_10:0.0139367931)24:0.0000025532)50:0.0000025532,(sintenisii_48:0.0069700394,stenopterum_31:0.0051821292)81:0.0034757695)84:0.0020061392,erinaceum:0.0194165120)99:0.0106172596,(coridifolium_7:0.0000025532,thomasianum_54:0.0000025532)100:0.0083851490)91:0.0020255464)84:0.0084959880,(lepidioides_18:0.0151650536,spinosum_23:0.0305822145)100:0.0692729609)99:0.0228555735,(((fimbriatum_44:0.0104208537,Lycium_13:0.0121791153)76:0.0015885282,orbiculatum_33:0.0157921545)52:0.0000025532,(((munzurense_46:0.0099457608,stylosum_19:0.0097067447)64:0.0028366020,semnanense_50:0.0174315138)40:0.0000025532,(rhodopaeum_24:0.0000025532,syriacum_27:0.0000025532)100:0.0071501465)45:0.0017353051)12:0.0000025532)17:0.0000025532)92:0.0017138643,saxatile_34:0.0086633660)92:0.0016593334)67:0.0000025532);

TIME STAMP

----------

Date and time: Mon Jan 16 08:46:39 2023

Total CPU time used: 22.944 seconds (0h:0m:22s)

Total wall-clock time used: 24.85512209 seconds (0h:0m:24s)
