# Supplementary material for: Underestimated diversity in high elevations of a global biodiversity hotspot: two new endemic species of Aethionema (Brassicaceae) from the alpine zone of Iran
Source: Front Plant Sci. 2023 May 26;14:1182073. doi: 10.3389/fpls.2023.1182073 (PMC10250747; doi:10.3389/fpls.2023.1182073)
Supplement: Supplementary file 2 [file DataSheet_2.zip › Date Sheet 2/ITS/Aethionema_ITS_MrBayes_consensus_trees.docx]

#NEXUS

[ID: 2362539896]

begin taxa;

dimensions ntax=49;

taxlabels

HM1454_Aethionema_S1865

Umbellatum_Archibold_J1

S658_Sp_nova_HM478

Shirkuh_J2

HM1452_yazd_S1863

W_0184833_Aethionema_ITS

acarii_37

alanyae_39

arabicum

armenum

capitatumi_4

carneum

cordatum_6

coridifolium_7

demirizii_40

dumanii_9

edentulum_53

elongatum_12

erinaceum

eunomioides_10

fimbriatum_44

froedinii_11

glaucinum_41

grandiflorum_14

heterocarpum_15

huber-morathii_42

karamanicum_43

lepidioides_18

Lycium_13

membranaceum_33Copen

munzurense_46

orbiculatum_33

retsina_38

rhodopaeum_24

saxatile_34

schistosum_21

semnanense_50

sintenisii_48

spicatum_35

spinosum_23

stenopterum_31

stylosum_19

subulatum_20

syriacum_27

Szowitsii_51

thomasianum_54

turcicum_1

umbellatum_32

virgatum_26

;

end;

begin trees;

translate

1 HM1454_Aethionema_S1865,

2 Umbellatum_Archibold_J1,

3 S658_Sp_nova_HM478,

4 Shirkuh_J2,

5 HM1452_yazd_S1863,

6 W_0184833_Aethionema_ITS,

7 acarii_37,

8 alanyae_39,

9 arabicum,

10 armenum,

11 capitatumi_4,

12 carneum,

13 cordatum_6,

14 coridifolium_7,

15 demirizii_40,

16 dumanii_9,

17 edentulum_53,

18 elongatum_12,

19 erinaceum,

20 eunomioides_10,

21 fimbriatum_44,

22 froedinii_11,

23 glaucinum_41,

24 grandiflorum_14,

25 heterocarpum_15,

26 huber-morathii_42,

27 karamanicum_43,

28 lepidioides_18,

29 Lycium_13,

30 membranaceum_33Copen,

31 munzurense_46,

32 orbiculatum_33,

33 retsina_38,

34 rhodopaeum_24,

35 saxatile_34,

36 schistosum_21,

37 semnanense_50,

38 sintenisii_48,

39 spicatum_35,

40 spinosum_23,

41 stenopterum_31,

42 stylosum_19,

43 subulatum_20,

44 syriacum_27,

45 Szowitsii_51,

46 thomasianum_54,

47 turcicum_1,

48 umbellatum_32,

49 virgatum_26

;

tree con_50_majrule = [&U] (1[&prob=1.00000000e+00,prob_stddev=0.00000000e+00,prob_range={1.00000000e+00,1.00000000e+00},prob(percent)="100",prob+-sd="100+-0"]:4.225744e-03[&length_mean=7.94045692e-03,length_median=4.22574400e-03,length_95%HPD={1.39058000e-05,2.62689200e-02}],3[&prob=1.00000000e+00,prob_stddev=0.00000000e+00,prob_range={1.00000000e+00,1.00000000e+00},prob(percent)="100",prob+-sd="100+-0"]:1.189607e-02[&length_mean=2.06343828e-02,length_median=1.18960700e-02,length_95%HPD={1.13777600e-03,6.44642900e-02}],(2[&prob=1.00000000e+00,prob_stddev=0.00000000e+00,prob_range={1.00000000e+00,1.00000000e+00},prob(percent)="100",prob+-sd="100+-0"]:4.304662e-03[&length_mean=8.12037386e-03,length_median=4.30466200e-03,length_95%HPD={9.44434400e-07,2.73289000e-02}],((4[&prob=1.00000000e+00,prob_stddev=0.00000000e+00,prob_range={1.00000000e+00,1.00000000e+00},prob(percent)="100",prob+-sd="100+-0"]:1.779697e-03[&length_mean=3.96899496e-03,length_median=1.77969700e-03,length_95%HPD={4.33576000e-08,1.42435800e-02}],5[&prob=1.00000000e+00,prob_stddev=0.00000000e+00,prob_range={1.00000000e+00,1.00000000e+00},prob(percent)="100",prob+-sd="100+-0"]:1.762156e-03[&length_mean=3.91159744e-03,length_median=1.76215600e-03,length_95%HPD={3.36547400e-08,1.38919200e-02}])[&prob=9.99902783e-01,prob_stddev=6.99019910e-05,prob_range={9.99833343e-01,1.00000000e+00},prob(percent)="100",prob+-sd="100+-0"]:6.672484e-03[&length_mean=1.18664064e-02,length_median=6.67248400e-03,length_95%HPD={1.41942200e-04,3.84910000e-02}],6[&prob=1.00000000e+00,prob_stddev=0.00000000e+00,prob_range={1.00000000e+00,1.00000000e+00},prob(percent)="100",prob+-sd="100+-0"]:4.241437e-03[&length_mean=8.06424201e-03,length_median=4.24143700e-03,length_95%HPD={3.52763400e-06,2.67027700e-02}])[&prob=9.68085106e-01,prob_stddev=1.67856729e-03,prob_range={9.66612966e-01,9.70501639e-01},prob(percent)="97",prob+-sd="97+-0"]:4.289379e-03[&length_mean=8.12110388e-03,length_median=4.28937900e-03,length_95%HPD={6.28867400e-08,2.70498600e-02}])[&prob=9.30434420e-01,prob_stddev=1.41876482e-03,prob_range={9.29615021e-01,9.32559302e-01},prob(percent)="93",prob+-sd="93+-0"]:4.208111e-03[&length_mean=7.89071014e-03,length_median=4.20811100e-03,length_95%HPD={2.45221200e-06,2.64439500e-02}],((((7[&prob=1.00000000e+00,prob_stddev=0.00000000e+00,prob_range={1.00000000e+00,1.00000000e+00},prob(percent)="100",prob+-sd="100+-0"]:1.624476e-02[&length_mean=2.79150505e-02,length_median=1.62447600e-02,length_95%HPD={2.14023200e-03,8.64378800e-02}],47[&prob=1.00000000e+00,prob_stddev=0.00000000e+00,prob_range={1.00000000e+00,1.00000000e+00},prob(percent)="100",prob+-sd="100+-0"]:1.839312e-03[&length_mean=4.06285617e-03,length_median=1.83931200e-03,length_95%HPD={7.34353900e-08,1.45649500e-02}])[&prob=9.32600967e-01,prob_stddev=2.99618764e-03,prob_range={9.28115105e-01,9.34336981e-01},prob(percent)="93",prob+-sd="93+-0"]:4.377953e-03[&length_mean=8.31754518e-03,length_median=4.37795300e-03,length_95%HPD={1.31273400e-07,2.79178000e-02}],(13[&prob=1.00000000e+00,prob_stddev=0.00000000e+00,prob_range={1.00000000e+00,1.00000000e+00},prob(percent)="100",prob+-sd="100+-0"]:1.385675e-02[&length_mean=2.39429051e-02,length_median=1.38567500e-02,length_95%HPD={1.53441500e-03,7.48013100e-02}],16[&prob=1.00000000e+00,prob_stddev=0.00000000e+00,prob_range={1.00000000e+00,1.00000000e+00},prob(percent)="100",prob+-sd="100+-0"]:1.156875e-02[&length_mean=2.01426042e-02,length_median=1.15687500e-02,length_95%HPD={9.90375600e-04,6.44263400e-02}])[&prob=8.56855175e-01,prob_stddev=2.49115302e-03,prob_range={8.53952558e-01,8.59507805e-01},prob(percent)="86",prob+-sd="86+-0"]:4.243937e-03[&length_mean=8.03613959e-03,length_median=4.24393700e-03,length_95%HPD={2.62191500e-06,2.70684600e-02}])[&prob=9.93389256e-01,prob_stddev=8.37596643e-04,prob_range={9.92611522e-01,9.94444753e-01},prob(percent)="99",prob+-sd="99+-0"]:9.415286e-03[&length_mean=1.65151906e-02,length_median=9.41528600e-03,length_95%HPD={2.43389500e-04,5.26675000e-02}],(((((((8[&prob=1.00000000e+00,prob_stddev=0.00000000e+00,prob_range={1.00000000e+00,1.00000000e+00},prob(percent)="100",prob+-sd="100+-0"]:4.318305e-03[&length_mean=8.10818334e-03,length_median=4.31830500e-03,length_95%HPD={3.16321000e-06,2.68237000e-02}],43[&prob=1.00000000e+00,prob_stddev=0.00000000e+00,prob_range={1.00000000e+00,1.00000000e+00},prob(percent)="100",prob+-sd="100+-0"]:4.329194e-03[&length_mean=8.21011153e-03,length_median=4.32919400e-03,length_95%HPD={2.37964400e-05,2.75228500e-02}])[&prob=7.83400922e-01,prob_stddev=4.27068076e-03,prob_range={7.77123493e-01,7.86122993e-01},prob(percent)="78",prob+-sd="78+-0"]:3.961850e-03[&length_mean=7.63452571e-03,length_median=3.96185000e-03,length_95%HPD={1.88088300e-07,2.56208400e-02}],(15[&prob=1.00000000e+00,prob_stddev=0.00000000e+00,prob_range={1.00000000e+00,1.00000000e+00},prob(percent)="100",prob+-sd="100+-0"]:7.121207e-03[&length_mean=1.27011716e-02,length_median=7.12120700e-03,length_95%HPD={1.30278200e-04,4.06826600e-02}],23[&prob=1.00000000e+00,prob_stddev=0.00000000e+00,prob_range={1.00000000e+00,1.00000000e+00},prob(percent)="100",prob+-sd="100+-0"]:2.117976e-02[&length_mean=3.62866005e-02,length_median=2.11797600e-02,length_95%HPD={3.67360000e-03,1.11266900e-01}])[&prob=7.76429087e-01,prob_stddev=7.04699368e-03,prob_range={7.70401644e-01,7.86622965e-01},prob(percent)="78",prob+-sd="78+-1"]:4.315296e-03[&length_mean=8.26435067e-03,length_median=4.31529600e-03,length_95%HPD={2.09304800e-06,2.78967600e-02}],36[&prob=1.00000000e+00,prob_stddev=0.00000000e+00,prob_range={1.00000000e+00,1.00000000e+00},prob(percent)="100",prob+-sd="100+-0"]:6.675626e-03[&length_mean=1.19844215e-02,length_median=6.67562600e-03,length_95%HPD={1.67500200e-04,3.91267200e-02}])[&prob=5.93828121e-01,prob_stddev=8.50146405e-03,prob_range={5.82467641e-01,6.03022054e-01},prob(percent)="59",prob+-sd="59+-1"]:4.171234e-03[&length_mean=7.98860922e-03,length_median=4.17123400e-03,length_95%HPD={1.05503600e-07,2.68588000e-02}],27[&prob=1.00000000e+00,prob_stddev=0.00000000e+00,prob_range={1.00000000e+00,1.00000000e+00},prob(percent)="100",prob+-sd="100+-0"]:1.481968e-02[&length_mean=2.56633098e-02,length_median=1.48196800e-02,length_95%HPD={1.87456500e-03,8.05322100e-02}])[&prob=8.21482140e-01,prob_stddev=9.04277492e-03,prob_range={8.10343870e-01,8.32342648e-01},prob(percent)="82",prob+-sd="82+-1"]:5.056152e-03[&length_mean=9.45748820e-03,length_median=5.05615200e-03,length_95%HPD={4.91752300e-07,3.18250300e-02}],10[&prob=1.00000000e+00,prob_stddev=0.00000000e+00,prob_range={1.00000000e+00,1.00000000e+00},prob(percent)="100",prob+-sd="100+-0"]:2.306710e-02[&length_mean=3.95398006e-02,length_median=2.30671000e-02,length_95%HPD={4.25769500e-03,1.23507800e-01}])[&prob=9.95416921e-01,prob_stddev=8.27091511e-04,prob_range={9.94278096e-01,9.96055775e-01},prob(percent)="100",prob+-sd="100+-0"]:1.047347e-02[&length_mean=1.82412632e-02,length_median=1.04734700e-02,length_95%HPD={2.15695100e-04,5.84404600e-02}],39[&prob=1.00000000e+00,prob_stddev=0.00000000e+00,prob_range={1.00000000e+00,1.00000000e+00},prob(percent)="100",prob+-sd="100+-0"]:1.420210e-02[&length_mean=2.45402403e-02,length_median=1.42021000e-02,length_95%HPD={1.37951400e-03,7.64900300e-02}])[&prob=8.38536748e-01,prob_stddev=2.29430129e-03,prob_range={8.35786901e-01,8.40564413e-01},prob(percent)="84",prob+-sd="84+-0"]:6.003404e-03[&length_mean=1.11105942e-02,length_median=6.00340400e-03,length_95%HPD={7.30540800e-07,3.66021000e-02}],((((11[&prob=1.00000000e+00,prob_stddev=0.00000000e+00,prob_range={1.00000000e+00,1.00000000e+00},prob(percent)="100",prob+-sd="100+-0"]:1.782464e-03[&length_mean=3.96123111e-03,length_median=1.78246400e-03,length_95%HPD={7.08533600e-08,1.40399600e-02}],26[&prob=1.00000000e+00,prob_stddev=0.00000000e+00,prob_range={1.00000000e+00,1.00000000e+00},prob(percent)="100",prob+-sd="100+-0"]:1.788738e-03[&length_mean=3.99294906e-03,length_median=1.78873800e-03,length_95%HPD={1.08900600e-08,1.42151200e-02}])[&prob=1.00000000e+00,prob_stddev=0.00000000e+00,prob_range={1.00000000e+00,1.00000000e+00},prob(percent)="100",prob+-sd="100+-0"]:9.039944e-03[&length_mean=1.59352436e-02,length_median=9.03994400e-03,length_95%HPD={3.97064500e-04,5.13823500e-02}],((17[&prob=1.00000000e+00,prob_stddev=0.00000000e+00,prob_range={1.00000000e+00,1.00000000e+00},prob(percent)="100",prob+-sd="100+-0"]:6.607178e-03[&length_mean=1.18712078e-02,length_median=6.60717800e-03,length_95%HPD={1.83241900e-04,3.84993100e-02}],24[&prob=1.00000000e+00,prob_stddev=0.00000000e+00,prob_range={1.00000000e+00,1.00000000e+00},prob(percent)="100",prob+-sd="100+-0"]:4.225992e-03[&length_mean=7.90593120e-03,length_median=4.22599200e-03,length_95%HPD={2.07562000e-05,2.63810800e-02}],30[&prob=1.00000000e+00,prob_stddev=0.00000000e+00,prob_range={1.00000000e+00,1.00000000e+00},prob(percent)="100",prob+-sd="100+-0"]:6.635371e-03[&length_mean=1.19247591e-02,length_median=6.63537100e-03,length_95%HPD={2.30006000e-04,3.83963500e-02}])[&prob=9.58974501e-01,prob_stddev=9.92198828e-04,prob_range={9.57946781e-01,9.60279984e-01},prob(percent)="96",prob+-sd="96+-0"]:6.428902e-03[&length_mean=1.16311018e-02,length_median=6.42890200e-03,length_95%HPD={1.51451900e-05,3.76675500e-02}],48[&prob=1.00000000e+00,prob_stddev=0.00000000e+00,prob_range={1.00000000e+00,1.00000000e+00},prob(percent)="100",prob+-sd="100+-0"]:1.421476e-02[&length_mean=2.46312881e-02,length_median=1.42147600e-02,length_95%HPD={1.49156100e-03,7.66191500e-02}])[&prob=1.00000000e+00,prob_stddev=0.00000000e+00,prob_range={1.00000000e+00,1.00000000e+00},prob(percent)="100",prob+-sd="100+-0"]:1.176541e-02[&length_mean=2.03864141e-02,length_median=1.17654100e-02,length_95%HPD={9.48595300e-04,6.43669300e-02}],((18[&prob=1.00000000e+00,prob_stddev=0.00000000e+00,prob_range={1.00000000e+00,1.00000000e+00},prob(percent)="100",prob+-sd="100+-0"]:4.212499e-03[&length_mean=7.95981713e-03,length_median=4.21249900e-03,length_95%HPD={1.67770800e-06,2.67683200e-02}],49[&prob=1.00000000e+00,prob_stddev=0.00000000e+00,prob_range={1.00000000e+00,1.00000000e+00},prob(percent)="100",prob+-sd="100+-0"]:1.799554e-03[&length_mean=4.03345688e-03,length_median=1.79955400e-03,length_95%HPD={1.64278000e-08,1.44382100e-02}])[&prob=9.63779790e-01,prob_stddev=1.60173611e-03,prob_range={9.61891006e-01,9.65724126e-01},prob(percent)="96",prob+-sd="96+-0"]:4.181056e-03[&length_mean=7.91243996e-03,length_median=4.18105600e-03,length_95%HPD={1.48298600e-07,2.65510500e-02}],45[&prob=1.00000000e+00,prob_stddev=0.00000000e+00,prob_range={1.00000000e+00,1.00000000e+00},prob(percent)="100",prob+-sd="100+-0"]:1.872821e-03[&length_mean=4.14345550e-03,length_median=1.87282100e-03,length_95%HPD={2.06528000e-08,1.50780600e-02}])[&prob=1.00000000e+00,prob_stddev=0.00000000e+00,prob_range={1.00000000e+00,1.00000000e+00},prob(percent)="100",prob+-sd="100+-0"]:1.865275e-02[&length_mean=3.22268649e-02,length_median=1.86527500e-02,length_95%HPD={2.68484000e-03,9.98150400e-02}],20[&prob=1.00000000e+00,prob_stddev=0.00000000e+00,prob_range={1.00000000e+00,1.00000000e+00},prob(percent)="100",prob+-sd="100+-0"]:2.110886e-02[&length_mean=3.62369956e-02,length_median=2.11088600e-02,length_95%HPD={3.55155700e-03,1.11975200e-01}],(38[&prob=1.00000000e+00,prob_stddev=0.00000000e+00,prob_range={1.00000000e+00,1.00000000e+00},prob(percent)="100",prob+-sd="100+-0"]:1.166357e-02[&length_mean=2.03546813e-02,length_median=1.16635700e-02,length_95%HPD={1.06171300e-03,6.54064800e-02}],41[&prob=1.00000000e+00,prob_stddev=0.00000000e+00,prob_range={1.00000000e+00,1.00000000e+00},prob(percent)="100",prob+-sd="100+-0"]:9.000596e-03[&length_mean=1.59477153e-02,length_median=9.00059600e-03,length_95%HPD={3.89347900e-04,5.12877700e-02}])[&prob=9.66474085e-01,prob_stddev=3.68953723e-03,prob_range={9.63279818e-01,9.70612744e-01},prob(percent)="97",prob+-sd="97+-0"]:6.708496e-03[&length_mean=1.20079337e-02,length_median=6.70849600e-03,length_95%HPD={9.09560400e-05,3.86464500e-02}])[&prob=6.28145659e-01,prob_stddev=6.59015192e-03,prob_range={6.21687684e-01,6.36409088e-01},prob(percent)="63",prob+-sd="63+-1"]:4.760986e-03[&length_mean=8.93016496e-03,length_median=4.76098600e-03,length_95%HPD={8.27062500e-07,2.99880000e-02}],19[&prob=1.00000000e+00,prob_stddev=0.00000000e+00,prob_range={1.00000000e+00,1.00000000e+00},prob(percent)="100",prob+-sd="100+-0"]:2.897670e-02[&length_mean=4.98678995e-02,length_median=2.89767000e-02,length_95%HPD={5.93035300e-03,1.54924300e-01}])[&prob=1.00000000e+00,prob_stddev=0.00000000e+00,prob_range={1.00000000e+00,1.00000000e+00},prob(percent)="100",prob+-sd="100+-0"]:1.737700e-02[&length_mean=3.00681004e-02,length_median=1.73770000e-02,length_95%HPD={1.95392800e-03,9.30247100e-02}],(14[&prob=1.00000000e+00,prob_stddev=0.00000000e+00,prob_range={1.00000000e+00,1.00000000e+00},prob(percent)="100",prob+-sd="100+-0"]:1.757918e-03[&length_mean=3.93599044e-03,length_median=1.75791800e-03,length_95%HPD={3.80290100e-08,1.38771100e-02}],46[&prob=1.00000000e+00,prob_stddev=0.00000000e+00,prob_range={1.00000000e+00,1.00000000e+00},prob(percent)="100",prob+-sd="100+-0"]:1.783295e-03[&length_mean=3.96894657e-03,length_median=1.78329500e-03,length_95%HPD={8.88830200e-08,1.41433200e-02}])[&prob=1.00000000e+00,prob_stddev=0.00000000e+00,prob_range={1.00000000e+00,1.00000000e+00},prob(percent)="100",prob+-sd="100+-0"]:1.367144e-02[&length_mean=2.38694508e-02,length_median=1.36714400e-02,length_95%HPD={1.46235800e-03,7.57459700e-02}])[&prob=8.69757236e-01,prob_stddev=3.06647618e-03,prob_range={8.66451864e-01,8.73507027e-01},prob(percent)="87",prob+-sd="87+-0"]:4.973938e-03[&length_mean=9.47286996e-03,length_median=4.97393800e-03,length_95%HPD={2.61086600e-06,3.20864400e-02}])[&prob=8.53841453e-01,prob_stddev=4.56319944e-03,prob_range={8.49397256e-01,8.58285651e-01},prob(percent)="85",prob+-sd="85+-0"]:1.491848e-02[&length_mean=2.59803132e-02,length_median=1.49184800e-02,length_95%HPD={1.54009300e-06,8.23737100e-02}],(28[&prob=1.00000000e+00,prob_stddev=0.00000000e+00,prob_range={1.00000000e+00,1.00000000e+00},prob(percent)="100",prob+-sd="100+-0"]:2.301105e-02[&length_mean=3.97248709e-02,length_median=2.30110500e-02,length_95%HPD={2.19352100e-03,1.23675200e-01}],40[&prob=1.00000000e+00,prob_stddev=0.00000000e+00,prob_range={1.00000000e+00,1.00000000e+00},prob(percent)="100",prob+-sd="100+-0"]:4.214922e-02[&length_mean=7.19166084e-02,length_median=4.21492200e-02,length_95%HPD={8.09160800e-03,2.21311200e-01}])[&prob=1.00000000e+00,prob_stddev=0.00000000e+00,prob_range={1.00000000e+00,1.00000000e+00},prob(percent)="100",prob+-sd="100+-0"]:8.952926e-02[&length_mean=1.54313646e-01,length_median=8.95292600e-02,length_95%HPD={2.56049300e-02,4.72207200e-01}])[&prob=1.00000000e+00,prob_stddev=0.00000000e+00,prob_range={1.00000000e+00,1.00000000e+00},prob(percent)="100",prob+-sd="100+-0"]:3.319399e-02[&length_mean=5.70088397e-02,length_median=3.31939900e-02,length_95%HPD={5.55532400e-03,1.77660100e-01}],((9[&prob=1.00000000e+00,prob_stddev=0.00000000e+00,prob_range={1.00000000e+00,1.00000000e+00},prob(percent)="100",prob+-sd="100+-0"]:9.007832e-03[&length_mean=1.59616759e-02,length_median=9.00783200e-03,length_95%HPD={4.07655100e-04,5.03347300e-02}],22[&prob=1.00000000e+00,prob_stddev=0.00000000e+00,prob_range={1.00000000e+00,1.00000000e+00},prob(percent)="100",prob+-sd="100+-0"]:4.157590e-03[&length_mean=7.89118075e-03,length_median=4.15759000e-03,length_95%HPD={1.02604800e-06,2.63477700e-02}])[&prob=9.99666685e-01,prob_stddev=1.01424469e-04,prob_range={9.99555580e-01,9.99777790e-01},prob(percent)="100",prob+-sd="100+-0"]:7.407836e-03[&length_mean=1.32786518e-02,length_median=7.40783600e-03,length_95%HPD={2.14626800e-04,4.30493000e-02}],(12[&prob=1.00000000e+00,prob_stddev=0.00000000e+00,prob_range={1.00000000e+00,1.00000000e+00},prob(percent)="100",prob+-sd="100+-0"]:9.977862e-03[&length_mean=1.74483591e-02,length_median=9.97786200e-03,length_95%HPD={5.76695400e-04,5.51835300e-02}],25[&prob=1.00000000e+00,prob_stddev=0.00000000e+00,prob_range={1.00000000e+00,1.00000000e+00},prob(percent)="100",prob+-sd="100+-0"]:1.548763e-02[&length_mean=2.68658455e-02,length_median=1.54876300e-02,length_95%HPD={2.14481000e-03,8.46329100e-02}])[&prob=6.11507694e-01,prob_stddev=4.43756681e-03,prob_range={6.08799511e-01,6.18132326e-01},prob(percent)="61",prob+-sd="61+-0"]:4.261445e-03[&length_mean=8.02523584e-03,length_median=4.26144500e-03,length_95%HPD={6.62370600e-07,2.62977900e-02}])[&prob=1.00000000e+00,prob_stddev=0.00000000e+00,prob_range={1.00000000e+00,1.00000000e+00},prob(percent)="100",prob+-sd="100+-0"]:1.371380e-02[&length_mean=2.37132226e-02,length_median=1.37138000e-02,length_95%HPD={1.19502200e-03,7.50024200e-02}],21[&prob=1.00000000e+00,prob_stddev=0.00000000e+00,prob_range={1.00000000e+00,1.00000000e+00},prob(percent)="100",prob+-sd="100+-0"]:1.732463e-02[&length_mean=3.00478555e-02,length_median=1.73246300e-02,length_95%HPD={2.35448000e-03,9.35750300e-02}],29[&prob=1.00000000e+00,prob_stddev=0.00000000e+00,prob_range={1.00000000e+00,1.00000000e+00},prob(percent)="100",prob+-sd="100+-0"]:1.943147e-02[&length_mean=3.35191280e-02,length_median=1.94314700e-02,length_95%HPD={2.89779500e-03,1.04946900e-01}],(31[&prob=1.00000000e+00,prob_stddev=0.00000000e+00,prob_range={1.00000000e+00,1.00000000e+00},prob(percent)="100",prob+-sd="100+-0"]:1.600200e-02[&length_mean=2.78308557e-02,length_median=1.60020000e-02,length_95%HPD={1.75846700e-03,8.73139300e-02}],42[&prob=1.00000000e+00,prob_stddev=0.00000000e+00,prob_range={1.00000000e+00,1.00000000e+00},prob(percent)="100",prob+-sd="100+-0"]:1.558566e-02[&length_mean=2.71995456e-02,length_median=1.55856600e-02,length_95%HPD={1.55342900e-03,8.51839400e-02}])[&prob=5.64385312e-01,prob_stddev=9.35752722e-03,prob_range={5.57469029e-01,5.78190101e-01},prob(percent)="56",prob+-sd="56+-1"]:5.999469e-03[&length_mean=1.11041431e-02,length_median=5.99946900e-03,length_95%HPD={7.39720800e-07,3.73896200e-02}],32[&prob=1.00000000e+00,prob_stddev=0.00000000e+00,prob_range={1.00000000e+00,1.00000000e+00},prob(percent)="100",prob+-sd="100+-0"]:2.302964e-02[&length_mean=3.98254144e-02,length_median=2.30296400e-02,length_95%HPD={4.35624300e-03,1.22592100e-01}],33[&prob=1.00000000e+00,prob_stddev=0.00000000e+00,prob_range={1.00000000e+00,1.00000000e+00},prob(percent)="100",prob+-sd="100+-0"]:3.290137e-02[&length_mean=5.62707545e-02,length_median=3.29013700e-02,length_95%HPD={7.11083600e-03,1.73180700e-01}],(34[&prob=1.00000000e+00,prob_stddev=0.00000000e+00,prob_range={1.00000000e+00,1.00000000e+00},prob(percent)="100",prob+-sd="100+-0"]:1.735958e-03[&length_mean=3.87612229e-03,length_median=1.73595800e-03,length_95%HPD={1.46488400e-08,1.40320100e-02}],44[&prob=1.00000000e+00,prob_stddev=0.00000000e+00,prob_range={1.00000000e+00,1.00000000e+00},prob(percent)="100",prob+-sd="100+-0"]:1.741449e-03[&length_mean=3.89501804e-03,length_median=1.74144900e-03,length_95%HPD={5.23282800e-08,1.39825500e-02}])[&prob=1.00000000e+00,prob_stddev=0.00000000e+00,prob_range={1.00000000e+00,1.00000000e+00},prob(percent)="100",prob+-sd="100+-0"]:1.186552e-02[&length_mean=2.06201922e-02,length_median=1.18655200e-02,length_95%HPD={9.59209000e-04,6.45988000e-02}],37[&prob=1.00000000e+00,prob_stddev=0.00000000e+00,prob_range={1.00000000e+00,1.00000000e+00},prob(percent)="100",prob+-sd="100+-0"]:2.540766e-02[&length_mean=4.37841249e-02,length_median=2.54076600e-02,length_95%HPD={4.80624500e-03,1.36193300e-01}])[&prob=8.63327037e-01,prob_stddev=2.44530797e-03,prob_range={8.60729959e-01,8.66229654e-01},prob(percent)="86",prob+-sd="86+-0"]:4.535066e-03[&length_mean=8.45977344e-03,length_median=4.53506600e-03,length_95%HPD={2.53436400e-06,2.80047100e-02}],35[&prob=1.00000000e+00,prob_stddev=0.00000000e+00,prob_range={1.00000000e+00,1.00000000e+00},prob(percent)="100",prob+-sd="100+-0"]:1.453219e-02[&length_mean=2.50549252e-02,length_median=1.45321900e-02,length_95%HPD={1.56044900e-03,7.89732600e-02}])[&prob=8.51383256e-01,prob_stddev=2.90696576e-03,prob_range={8.47675129e-01,8.54230321e-01},prob(percent)="85",prob+-sd="85+-0"]:4.416584e-03[&length_mean=8.37312503e-03,length_median=4.41658400e-03,length_95%HPD={3.97767600e-07,2.81285800e-02}]);

end;
