# Supplementary material for: Underestimated diversity in high elevations of a global biodiversity hotspot: two new endemic species of Aethionema (Brassicaceae) from the alpine zone of Iran
Source: Front Plant Sci. 2023 May 26;14:1182073. doi: 10.3389/fpls.2023.1182073 (PMC10250747; doi:10.3389/fpls.2023.1182073)
Supplement: Supplementary file 2 [file DataSheet_2.zip › Date Sheet 2/ITS/Aethionema_ITS_MrBayes_input_NEXUS.docx]

#NEXUS

BEGIN DATA;

DIMENSIONS NTAX=49 NCHAR=629;

FORMAT MISSING=? DATATYPE=DNA interleave=yes gap=- missing=?;

MATRIX

HM1454_Aethionema_S1865 CATCGAAAAATTTGGGTCATGTTTAAAGCTCACGGACAAGAAGAGCGACCCGACTATAACAAGAGGCTTTT-GTTCACCACCGCTAGTCGGGACAGTCTTGCTGCCCTTGGCTCGGATTTAGGCCAACCGCGTGCGGTACCACACGGGAGACCAACTTCCGTCCCT---TATCCAAGAT-GGATGGGGGGACG----ACGTTTTGTGACACCCAGGCAGACGTGCCCTCGGCCCTAAGGCTTGAGGCGCAACTTGCGTTCAAAGACTCGATGGTTCACGGGATTCTGCAATTCACACCAAGTATCGCATTTCGCTACGTTCTTCATCGATGCGAGAGCCGAGATATCCGTTGCCGAGAGTCGTTTAGACTTTAGATACTGGCACC-ACGTATGCGCACACCGTCTCCGGGTTGCAAACGCGGACCATTTCGTTCAATGTTCCTTGGCACTTTTAATGCCGGGGTTTTGTGATATCCAAGGAGAGCCGATCACG-ATACAATCCAGAGAGAACCCTGAAAAGGACCGTGCGCGGATCCATGGAGTTGGTGAA-CGCAAAACCGGTTAAGGCCCTGGCTCACCGCGAGTGATGATCAA-TTGTTCACGGGTCATTCTGTTTTGGACAGGTT

Umbellatum_Archibold_J1 CATCGAAAAATTTGGGTCATGTT-AAAGCTCGCGGACAAGAAGAGCGACCCGACTATAACAAGAGGCTTTT-GTTCACCACCGCTAGTCGGGACAGTCTTGCTGCCCTTGGCTCGGATTTAGGCCAACCGCGTGCGGTACCACACGGGAGACCAACTTCCGTCCCT---TATCCAAGAT-GGATGGGGGGACG----ACGTTTTGTGACACCCAGGCAGACGTGCCCTCGGCCCGAAGGCTTGAGGCGCAACTTGCGTTCAAAGACTCGATGGTTCACGGGATTCTGCAATTCACACCAAGTATCGCATTTCGCTACGTTCTTCATCGATGCGAGAGCCGAGATATCCGTTGCCGAGAGTCGTTTAGACTTTAGATACTGGCACC-ACGTATGCGCACACCGTCTCCGGGTTGCAAACGCGGACCATTTCGTTCAATGTTCCTTGGCACTTTTAATGCCGGGGTTTTGTGATATCCAAGGAGAGCCGATCACG-ATACAATCCAGAGAGAACCCTGAAAAGGACCGTGCGCGGATCCATGGAGTTGGTGAA-CGCAAAACCGATTAAGGCCCTGGCTCACCGCGAGTGATGATCAA-TTGTTCACGGGTCATTCTGTTTTGGACAGGTT

S658_Sp_nova_HM478 CATCGAAAAATTTGGGTCATGTT-AAAGCTCACGGACAAGAAGAGCGACCCGACTATAACAAGAGGCTTTT-GTTCACCACCGCTAGTCGGGACAGTCTTGCTGCCCTTGGCTCGGATTTAGGCCAACCGCGTGCGGTACCACACGGGAAACCAACTTCCGTCCCT---TATCCAAGAT-GGATGGGGGGACG----ACGTTTTGTGACACCCAGGCAGACGTGCCCTCGGCCCGAAGGCTTGAGGCGCAACTTGCGTTCAAAGACTCGATGGTTCACGGGATTCTGCAATTCACACCAAGTATCGCATTTCGCTACGTTCTTCATCGATGCGAGAGCCGAGATATCCGTTGCCGAGAGTCGTTTAGACTTTAGATACTGGTACC-ACGTATGCGCACACCGTCTCCGGGTTGCAAACGCGGACCATTTCGTTCAATGTTCCTTGGCACTTTTAATGCCGGGGTTTTGTGATATCCAAGGAGAGCCGATCACG-ATTCAATCCAGAGAGAACCCTGAAAAGGACCGTGCACGGATCCATGGAGTTGGTGAA-CGCAAAACCGGTTAAGGCCCTGGCTCACCGCGAGTGATGAT------------------------------------

Shirkuh_J2 CATCGAAAAATTTGGGTCATGTT-AAAGCTCGCGGACAAGAAGAGCGACCCGACTATAACAAGAGGCTTTT-GTTCACCACCGCTAGTCGGGACAGTCTTGCTGCCCTTGGCTCGGATTTAGGCCAACCGCGTGCGGTACCACACGGGAGACCAACTTCCGTCCCT---TATCCAAGAT-GGATGGGGG-ACG----ACGTTTTGTGACACCCAGGCAGACGTGCCCTCGGCCCGAAGGCTTGAGGCGCAACTTGCGTTCAAAGACTCGATGGTTCACGGGATTCTGCAATTCACACCAAGTATCGCATTTCGCTACGTTCTTCATCGATGCGAGAGCCGAGATATCCGTTGCCGAGAGTCGTTTAGACTTTAGATACTGGCACC-ACGTATGCGCACACCGTCTCCGGGTTGCAAACGCGGACCATTTCGTTCAATGTTCCTTGGCACTTTTAATGCCGGGGTTTTGTGATATCCAAGGAGAGCCGATCACG-ATACAATCCAGAGAGAACCCTGAAAAGGACCGTGCGCGGATCCATGGAGTTGGTGAA-CACAAAACCGGTTAAGGTCCTGGCTCACCCCGAGTGATGATCAA-TTGTTCACGGGTCATTCTGTTTTGGACAGGTT

HM1452_yazd_S1863 CATCGAAAAATTTGGGTCATGTT-AAAGCTCGCGGACAAGAAGAGCGACCCGACTATAACAAGAGGCTTTT-GTTCACCACCGCTAGTCGGGACAGTCTTGCTGCCCTTGGCTCGGATTTAGGCCAACCGCGTGCGGTACCACACGGGAGACCAACTTCCGTCCCT---TATCCAAGAT-GGATGGGGG-ACG----ACGTTTTGTGACACCCAGGCAGACGTGCCCTCGGCCCGAAGGCTTGAGGCGCAACTTGCGTTCAAAGACTCGATGGTTCACGGGATTCTGCAATTCACACCAAGTATCGCATTTCGCTACGTTCTTCATCGATGCGAGAGCCGAGATATCCGTTGCCGAGAGTCGTTTAGACTTTAGATACTGGCACC-ACGTATGCGCACACCGTCTCCGGGTTGCAAACGCGGACCATTTCGTTCAATGTTCCTTGGCACTTTTAATGCCGGGGTTTTGTGATATCCAAGGAGAGCCGATCACG-ATACAATCCAGAGAGAACCCTGAAAAGGACCGTGCGCGGATCCATGGAGTTGGTGAA-CACAAAACCGGTTAAGGTCCTGGCTCACCCCGAGTGATGATCAA-TTGTTCACGGGTCATTCTGTTTTGGACAGGTT

W_0184833_Aethionema_ITS CATCGAAAAATTTGGGTCATGTT-AAAGCTCGCGGACGAGAAGAGCGACCCGACTATAACAAGAGGCTTTT-GTTCACCACCGCTAGTCGGGACAGTCTTGCTGCCCTTGGCTCGGATTTAGGCCAACCGCGTGCGGTACCACACGGGAGACCAACTTCCGTCCCT---TATCCAAGAT-GGATGGGGGGACG----ACGTTTTGTGACACCCAGGCAGACGTGCCCTCGGCCCGAAGGCTTGAGGCGCAACTTGCGTTCAAAGACTCGATGGTTCACGGGATTCTGCAATTCACACCAAGTATCGCATTTCGCTACGTTCTTCATCGATGCGAGAGCCGAGATATCCGTTGCCGAGAGTCGTTTAGACTTTAGATACTGGCACC-ACGTATGCGCACACCGTCTCCGGGTTGCAAACGCGGACCATTTCGTTCAATGTTCCTTGGCACTTTTAATGCCGGGGTTTTGTGATATCCAAGGAGAGCCGATCACG-ATACAATCCAGAGAGAACCCTGAAAAGGACCGTGCGCGGATCCATGGAGTTGGTGAA-CGCAAAACCGGTTAAGGTCCTGGCTCACCGCGAGTGATGATCAA-TTGTTCACGGGTCATTCTGTTTTGGACAGGTT

acarii_37 CATCGAAAAAGTTGGGTCATGTT-AAAGCTCACGGACAAGAAGAGCAACCCGACTATAACAAGAGGCTTTT-GTTCACCACCGCTAGTCGGGACAGTCTTGCTGCCCTTGGCTCGGATTTAGGCCAACCGCGTGCGGTACCACACGGGAGACCAACTTCCGTCCCT---TATCCAAGAT-GGATGGGGGGACG----ACATTTTGTGACACCCAGGCAGACGTGCCCTCGGCCCGAAGGCTTGAGGCGCAACTTGCGTTCAAAGACTCGATGGTTCACGGGATTCTGCAATTCACACCAAGTATCGCATTTCGCTACGTTCTTCATCGATGCGAGAGCCGAGATATCCGTTGCCGAGAGTCGTTTAGACTTTACATACGGGCACC-ACGTATGCGCACACCGTCTCCGGGTTGCAAACGTGGACCATTTCGTTCAATGATCCTTGGCACTTTTAATGCCGGGGTTTTGTGATATCCAAGGAGAGCCGATCACG-ATACAGGCCAGAGAGAACCCTGAAAAGGACCGTGCGCGGATCCATGGAGTCGGTGAA-CGCAAAACCGGTTAAGGCTCCGGCTCACCGCGAGTGATGATCAC-TTGTTCACGGGTCATTCTGTTTTGGACAGGTT

alanyae_39 CATCGAAAAATTTGGGTCATCTT-AAAGCTCACGGACAAGAAGAGCAGCCCGACTATAACAAGAGGCTTTT-GTTCACCACCGCTAGTCGGGACACTCTTGCTGCCCTTGGCTCGGATTTAGGCCAACCGCGTGCGGTACCACACGGGAGACCAACTTCCGTCCCG---TTTCCAAGAT-GGATGGGGGGACG----ACGTTTTGTGACACCCAGGCAGACGTGCCCTCGGCCCGAAGGCTTGAGGCGCAACTTGCGTTCAAAGACTCGATGGTTCACGGGATTCTGCAATTCACACCAAGTATCGCATTTCGCTACGTTCTTCATCGATGCGAGAGCCGAGATATCCGTTGCCGAGAGTCGTTTAGACTTTAGATACTGGCACC-ACGTTTGCATACACCGTCTCCGGGTCGCAAACGCAGACCATTTCGTTCAATGTTCCTTGGCACTTTTAATGCCGGGGTTTTGTGATATCCAAGGAAGGCCGATCACG-ATCCAACCCAAAGATAACTCTGAGAAGGACCGTGCACGGATCAATGGAGTCGGTGAA-CACAGGACCGGTTAAAGCCCTGGCTCACCGCGAGTGATGATCAA-TCGTTCACGGGTCATTCTGTTTTGGACAGGTT

arabicum CATCGAAAACTTTGGGGCATGTT-AAAGCTCACGGACAAGAAGACCAACCCGACTATAACAAGAGGCTTTT-GTTCACCACCGCTAGTCGGGACAGTCTTGCTGCCCTTGGCTCGGATTTAGGCCAACCGCGTGCGGTACCACACGGGAGACCAATTTCCGTCCCT---TATCCAAGAT-GGATGGGGGGACG----ACGTTTTGTGACACCCAGGCAGACGTGCCCTCGGCCCGAAGGCTTGAGGCGCAACTTGCGTTCAAAGACTCGATGGTTCACGGGATTCTGCAATTCACACCAAGTATCGCATTTCGCTACGTTCTTCATCGATGCGAGAGCTGAGATATCCGTTGCCGAGAGTCGTTTAGACTTTAGATACTGGCACC-ACGTATGCACACACCGTCTCCGGGTTGCAAACGCGAACCATTTCGTTCAATGTTCCTTGGCACTTTTAATGCCGGGGTTTCGTGATATCCAAGGAGAGCCGATCACG-TTACAATCCAGAGAGAACCCTGAAAAGGACCGTGCGCGGATCCATGGAGTCGGTGAA-CGCAAAACCGGTTAAGGCTCTGGCTCACCGCGAGTGGTGATCAA-TTGTTCACGGGTCATTCTGTTTTGGACAGGTT

armenum CATCGAAAAATTTGGGTCATCTT-AAAGCTCACGGACAAGAAGAGCAGCCCAACTATAACAAGAGGCTTTT-GTTCACCACCGCTAGTCGGGACACTCTTGCTGCCCTTGGCTCGGATTTAGGCCAACCGCGTGCGGTACCACACGGGAGACCAACTTCCGTCCCG---TTTCCAAGAT-GGATGGGGGGACG----ACGTTTTGTGACACCCAGGCAGACGTGCCCTCGGCCCGAAGGCTTGAGGCGCAACTTGCGTTCAAAGACTCGATGGTTCACGGGATTCTGCAATTCACACCAAGTATCGCATTTCGCTACGTTCTTCATCGATGCGAGAGCCGAGATATCCGTTGCCGAGAGTCGTTTAGACTTTAGATACTGGCACC-ACGTTTGCGTACACCGTCTCCGGGTCACAAACGCAGACCATTTCGTTTAATATTCCTTGGCACTTTTAATGCCGGGGTTTTGTGATATCCAAGGAGAACCGACCACGAATCCAACCCAAAGATAACTCTGAGAAGGACCGTGCACGGATCCATGGAGTCGGTGAA-CACAGGTCCGGTTAGAGCCCTGGCTCACCGCGAGTGATGAT------------------------------------

capitatumi_4 CATCGGAAATTTTGGGTCATCTT-AAAGCTCACGGACAAGAAGAGCAACCCGACTATAACAAGAGGCTTTT-GTTCACCACCGCTAGTCGGGACACTCTTGCTGCCCTTGGCTCGGATTTAGGCCAACCGCGTGCGGTACCACACGGGAGACCAACTTCCGTCCCG---TATCCAAGAT-GGATGGGGGGACG----ACGTTTTGTGACACCCAGGCAGACGTGCCCTCGGCCCGAAGGCTTGAGGCGCAACTTGCGTTCAAAGACTCGATGGTTCACGGGATTCTGCAATTCACACCAAGTATCGCATTTCGCTACGTTCTTCATCGATGCGAGAGCCGAGATATCCGTTGCCGAGAGTCGTTTAGACTTTAGATACTGGTACC-ACGTTTGCATACACCGTCTCCGGGTCGCAAACACAGACCATTTCGTTCAATGTTCCTTGGCACTTTTAATGCCGGGGTTTTGTGATATCCAAGGAAAGCCGATCACA-ATCCAACCCAAAGAGAACTCTGAGAAGGAC-GTTCGCGGATCCTTGGAGTCAGTGAA-CACAGGACCGGTTAAAGCCCTGGTTCACCGCGAGTGATGATCAA-TCGTTCACGGGTCATTCTGTTTTGGACAGGTT

carneum NATCCAAAACTTTGGGGCATGTT-AAAGCTCACGGGCAAGAAGAGCAACCCGACTATAACAAGAGGCTTTT-GTTCACCACCGCTAGTCGGGACAGTCTTGCTGCCCTTGGCTCGGATTTAGGCCAACCGCGTACGGTAACACACGGGAGACCAACTTCCGTCCCT---TATCCAAGAT-GGATGGGGGGACG----ACGTTTTGTGACACCCAGGCAGACGTGCCCTCGGCCCGAAGGCTTGAGGCGCAACTTGCGTTCAAAGACTCGATGGTTCACGGGATTCTGCAATTCACACCAAGTATCGCATTTCGCTACGTTCTTCATCGATGCGAGAGCCGAGATATCCGTTGCCGAGAGTCGTTTAGACTTTAGATACTGGCACC-ACGTATGCACACACCGTCTCCGGGTTGCAAACGCGAACCATTTCGTTCAATGTTCCTTGGCACTTTTAATGCCGGGGTTTTGTGATATCCAAGGAGAGCCGATCACG-TTACAATCCAGAGAGAACCCTGAAAAGGACCGTGCGCGGATCCATGGAGTCGGTGAA-CGCAAAACCGGTTAAGGCTCTGGCTCACCGCGAGTGATGATCAA-TTGTTCACGGGTCATTCTGTTTTGGACAGGTT

cordatum_6 CATCGAAAAAGTTGGGTCATGTTTAAAGCTCACGGACAAGAAGAACAACCCGACTATAACAAGAGGCTTTT-GTTCACCACCGCTAGTCGGGACAGTCTTGCTGCCCTTGGCTCGGATTTAGGCCAACCGCGTGCGGTACCACACGGGAGACCAACTTCCGTCCCA---TATCCAAGAT-GGATGGGGGGACG----ACGTTTTGTGACACCCAGGCAGACGTGCCCTCGGCCCGAAGGCTTGAGGCGCAACTTGCGTTCAAAGACTCGATGGTTCACGGGATTCTGCAATTCACACCAAGTATCGCATTTCGCTACGTTCTTCATCGATGCGAGAGCCAAGATATCCGTTGCCGAGAGTCGTTTAGACTTCAGATACTGGCACC-ACGTATGCGCACACCGTCTCCGGGTTACAAACGCGGACCATTTCGTTCAATGTTCCTTGGCACTTTTAATGCCGGGGTTTTGTGATATCCAAGGAGAGCCGATCACG-ATACAGTCCAGAGAGAACCCTGAAAAGGACCGCGCGCGGATCCATGGAGTCGGTGAA-CGCAAAACCGGTTAAGGCTCTGGCTCACCGCGAGTGATGATCAC-TTGTTCACGGGTCATTCTGTTTTGGACAGGTT

coridifolium_7 CATCGAAAAATTTGGGTCATCTT-AAAGCTCACGGACAAGAAGAGGAACCCGACTATAACAAGAGGCTTTT-GTTCACCACCGCTAGTCGGGACACTCTTGTTGCCCTTTGCTCGGATTTAGGCCAACCGCGTGCGGTACCACACGGGAGACCAACTTCCGTCCCG---TATCCAAGAT-GGATGGGGGGACG----ACGTTTTGTGACACCCAGGCAGACGTGCCCTCGGCCCGAAGGCTTGAGGCGCAACTTGCGTTCAAAGACTCGATGGTTCACGGGATTCTGCAATTCACACCAAGTATCGCATTTCGCTACGTTCTTCATCGATGCGAGAGCCGAGATATCCGTTGCCGAGAGTCGTTTAGACTTTAGATACTGGCACC-ACGTTTGCATACACCGTCTCCGGGTCACAAACGCAGACCATTTCGTTCAATGTTCCTTGGCACTTTTAATGCCGGGGTTTTGTGATATCCAAGGAGAGCCGATCATG-ATCCAACCCAAAGAGAACTCTGAGAAGGACCGTGCGCGGATCCATGGAGTCGGTGAA-CACAAGACCGGTTAAAGCCCTGGTTCACCGCGAGTGATGATCAA-TCGTTCACGGGTCATTCTGTTTTGGACAGGTT

demirizii_40 CATCGAAAAATTTGGGTCATCTT-AAAGCTCACAGACAAGAAGAGCAGCCCGACTATAACAAGAGGCTTTT-GTTCACCACCGCTAGTCGGGACACTCTTGCTGCCCTTGGCTCGGATTTAGGCCAACCGCGTGCAGTACCACACGGGAGACCAACTTCCGTCCCG---TTTCCAAGAT-GGATGGGGGGACG----ACGTTTTGTGACACCCAGGCAGACGTGCCCTCGGCCCGAAGGCTTGAGGCGCAACTTGCGTTCAAAGACTCGATGGTTCACGGGATTCTGCAATTCACACCAAGTATCGCATTTCGCTACGTTCTTCATCGATGCGAGAGCCGAGATATCCGTTGCCGAGAGTCGTTTAGACTTTAGATACTGGCACC-ACGTTTGCATACACCGTCTCCGGGTCACAAACGCAGACCATTCCGTTCAATGTTCCTTGGCACTTTTAATGCCGGGGTTTTGTGATATCCAAGGAGGGCCGATCACG-ATCCAACCCAAAGATAACTCTGAGAAGGACCGTGCACGGATCAATGGAGTCGGTGAA-CACAGGACCGGTTAAAGCCCTGGCTCACCGCGAGTGATGATCAA-TCGTTCACGGGTCATTCTGTTTTGGACAGGTT

dumanii_9 CATCGAAAAAGGTGGGTCATGTT-AAAGCTCACGGACAAGAAGAGCAACCCGACTATAACAAGAGGCTTTT-GTTCACCACCGCTAGTCGGGACAGTCTTGCTGCCCTTGGCTCGGATTTAGGCCAACCGCGTGCGGTACCACACGGGAGACCAACTTCCGTCCCA---CATCCAAGAT-GGATGGGGGGACG----ACGTTTTGTGACACCCAGGCAGACGTGCCCTCGGCCCGAAGGCTTGAGGCGCAACTTGCGTTCAAAGACTCGATGGTTCACGGGATTCTGCAATTCACACCAAGTATCGCATTTCGCTACGTTCTTCATCGATGCGAGAGCCGAGATATCCGTTGCCGAGAGTCGTTTAGACTTTAGATACTGGCACC-ACGTATGCGCACACCGTCTCCGGGTTGCAAACGCGGACCATTTCGTTCAATGTTCCTTGGCACTTTTAATGCCGGGGTTTTGTGATATCCAAGGAGGGCCGAACACG-ATACAGTCCAGAGAGAACCCTGAAAAGGACCGTGCGCGGATCCATGGAGTCGGTGAA-CGCAAAACCGGTTAAGGCTCTGGCTCACCGCGAGTGATGATCAC-TTGTTCACGGGTCATTCTGTTTTGGACAGGTT

edentulum_53 CATCGAAAATTTTGGGTCATCTT-AAAGCTCACAGACAAAAAGAGCAACCCGACTATAACAAGAGGCTTTT-GTTCACCACCGCTAGTCGGGACACTCTTGCTGCCCTTGGCTCGGATTTAGGCCAACCGCGTGCGGTACCACACGGGAGACCAACTTCCGTCCCG---TATCCAAGATGGGATGGGGGGACG----ACGTTTTGTGACACCCAGGCAGACGTGCCCTCGGCCCGAAGGCTTGAGGCGCAACTTGCGTTCAAAGACTCGATGGTTCACGGGATTCTGCAATTCACACCAAGTATCGCATTTCGCTACGTTCTTCATCGATGCGAGAGCCAAGATATCCGTTGCCGAGAGTCGTTTAGACTTTAGATACTGGCACC-GCGTTTGCATACACCGTCTCCGGGTCGCAAACGCAGACCATTTCGTTCAATGTTCCTTGGCACTTTTAATGCCGGGGTTTTGTGATATCCAAGGAAA-CCGATCACA-ATCCAACCCAAATATAACTCTGAGAAGGAC-GATCACGGATCCTTGGAGTCAGTGAA-CACAGGACCGGTTAAAGCCCTGGTTCACCGCGAGTGATGATCAA-TCGTTCACGGGTCATTCTGTTTTGGACAGGTT

elongatum_12 CATCAAAAATTTTGGGTCATCTT-AAAGCTCACGGACAAGAAGAGCAACCCGACTATAACAAGAGGCTTTT-GTTCACCACCGCTTGTCGGGACACTCTTGCTGCCCTTGGCTTGGATTTAGGCCAACCGCATGCGGTACCACACGGGAGACCAACTTCCGTCCCA---TATCCAAGAT-GGATGGGGGGACG----ACGTTTTGTGACACCCAGGCAGACGTGCCCTCGGCCCGAAGGCTTAAGGCGCAACTTGCGTTCAAAGACTCGATGGTTCACGGGATTCTGCAATTCACACCAAGTATCGCATTTCGCTACGTTCTTCATCGATGCGAGAGCCGAGATATCCGTTGCCGAGAGTCGTTTAGACTTTAGATACTGGCACC-ACGTTTGCATACACCGTCTCCGGGTCGCAAATGCAGACCATTTCGTTCAATGTTCCTTGGCACTTTTAATGCCGGGGTTTTGTGATATCCAAGGAAAGCCGATCACA-ATCCAACCCAAAGAGAACTCTGAGAAGGAC-GTTCGCGGATCCTTGGAGTCAGTGAA-CACAGGACCGGTTAAAGCCCTGGTTCACCGCAAGTGATGATCAA-TTGTTCACGGGTCATTCTGTTTTGGACAGGTT

erinaceum CATCGAAAATTTTGGGTCATCTT-AAAGCTCACGGACAAGAAGAGCAACCCGACTATAACAAGAGGCTTCT-ATTCACCACCGCTAGTCGGGACACTCTTGCTGCCCTTGGCTCGGATTTAGGCCAACCGCGTGCGGTACCACACGGGAGACCAACTTCCGTCCCG---TATCCAAGAT-GGATGGGGGGACG----ACGTTTTGTGACACCCAGGCAGACGTGCCCTCGGCCCGAAGGCTTGAGGCGCAACTTGCGTTCAAAGACTCGATGGTTCACGGGATTCTGCAATTCACACCAAGTATCGCATTTCGCTACGTTCTTCATCGATGCGAGAGCCGAGATATCCGTTGCCGAGAGTCGTTTAGACTTTAGATACTGGCACC-ACGTTTGCATACACCGTCTCCGGGGCACAAACGCAGACCATTT-GTTCAATGTTCCTTGGCACTTTTAATGCCGGGGTTTTGTGATATCCTACGAAAACCGATCACA-ATCCGACCCAAAGAGAACTCTGAGAAGGAC-GTTCGCGGATCCATGGAGTCAGTGAA-CACGGGGCCGGTTAAAGCCCTGGTTCACCGCTAGTGATGATCAA-TCGTTCACGGGTCATTCTGTTTTGGACAGGTT

eunomioides_10 CATCGAAAATTTTGGGTCATCTT-AAAGCTCACGGACAAGAAGAGCAACCCGACTATAACAAGAGGCTTTT-GTTCACCACCGCTAGTCGGGACACTCTTGCTGCCCTTGGCTCGGATTTAGGCCAACCGCGTGCGGTACCACACGGGAGACCAACTTCTGTCCCG---TATCCAAGAT-GGATGGGGGGACA----ACGTTTTGTGACACCCAGGCAGACGTGCCCTCGGCCCGAAGGCTTGAGGCGCAACTTGCGTTCAAAGACTCGATGGTTCACGGGATTCTGCAATTCACACCAAGTATCGCATTTCGCTACGTTCTTCATCGATGCGAGAGCCGAGATATCCGTTGCCGAGAGTCGTTTAGACTTTAGATACTGGCACC-ACGTTTGCATACACCGTCTCCGGGTTGCAAACGCAGACCGTTTCGTTCAATGTTCCTTGGCACTTTTAATGCCGGGGTTTTGTGATATCCAAGGAAAGACGATCACA-ATCCAACCCAAAGAGAACTCTGAGAAGGAT-GTTCGCGGATCCTTGGAGTCAGTGAA-CACAGGACCGGTTAAAGCCCTGGTTCACTGCGAGTGATGATCAA-TCGTTCACAGGTCATTCTGTTTTGGACAGGTT

fimbriatum_44 CATCGAAAAATTTGGGTCATGTT-AAAGCTCACGGACAAGAAGAGCAACCCGACTATAACAAGAGGCTTTT-GTTCACCACCGCTAGTCGGGACAGTCTTGCTGCCCTTGGCTCAGATTTAGGCCAACCGCGTGCGGTACCACACGGGAGACCAACTTCCGTCCCT---TATCCAAGAT-GGATGGGGGGACG----ACGTTTTGTGACACCCAGGCAGACGTGCCCTCGGCCCGAAGGCTTGAGGCGCAACTTGCGTTCAAAGACTCGATGGTTCACGGGATTCTGCAATTCACACCAAGTATCGCATTTCGCTACGTTCTTCATCGATGCGAGAGCCGAGATATCCGTTGCCGAGAGTCGTTTAGACTTTAGATACTGGCACC-ACGTATGCGCACACCGTCTCCGGGTTGCAAACGCGGACCATTTTGTTCAATATTCCTTGGCACTTTTAATGCCGGGGTTTTGTGATATCCAAAGAGAGCCGATCACG-ATACAATCCAAAGAGAACCCTGAAAAGCACCGTGCGCGGATCCATGGAGTCGGTGAA-CGCAAAACTGGTTAAGGCCCTGGCTCACCGCGAGTGATGATCAA-TTGTTCACGGGTCATTCTGTTTTGGACAGGTT

froedinii_11 CATCGAAAACTTTGGGGCATGTT-AAAGCTCACGGACAAGAAGACCAACCCGACTATAACAAGAGGCTTTT-GTTCACCACCGCTAGTCGGGACAGTCTTGCTGCCCTTGGCTCGAATTTAGGCCAACCGCGTGCGGTACCACACGGGAGACCAACTTCCGTCCCT---TATCCAAGAT-GGATGGGGGGACG----ACGTTTTGTGACACCCAGGCAGACGTGCCCTCGGCCCGAAGGCTTGAGGCGCAACTTGCGTTCAAAGACTCGATGGTTCACGGGATTCTGCAATTCACACCAAGTATCGCATTTCGCTACGTTCTTCATCGATGCGAGAGCCGAGATATCCGTTGCCGAGAGTCGTTTAGACTTTAGATACTGGCACC-ACGTATGCACACACCGTCTCCGGGTTGCAAACGCGAACCATTTCGTTCAATGTTCCTTGGCACTTTTAATGCCGGGGTTTTGTGATATCCAAGGAGAGCCGATCACG-TTACAATCCAGAGAGAACCCTGAAAAGGACCGTGCGCGGATCCATGGAGTCGGTGAA-CGCAAAACCGGTTAAGGCTCTGGCTCACCGCGAGTGGTGATCAA-TTGTTCACGGGTCATTCTGTTTTGGACAGGTT

glaucinum_41 CATCGAAAAATTTGGGTCATCTT-GAAGCTCACGGACAAGAAGAGCGACCCGACCATAACAAGAGGCTTTT-GTTCACCACCGCTAGTCGGGACACTCTTGTTGCCCTTGGCTCGGATTTAGGCCAACCGCGTGCGGTACCACACGGGAGACCAACTTCCGTCCCG---TATCCAAGAT-GGATGGGGGGACG----ACGTTTTGTGACACCCAGGCAGACGTGCCCTTGGCCCGAAGGCTTGAGGCGCAACTTGCGTTCAAAGACTCGATGGTTCACGGGATTCTGCAATTCACACCAAGTATCGCATTTCGCTACGTTCTTCATCGATGCGAGAGCCGAGATATCCGTTGCCGAGAGTCGTTTAGACTTTAGATACTGGCACC-ACGTTTGCATACACCGTCTCCGGGTCACAAACGCAGACCATTCCGTTCAATGTTCCTTGGCACTTTTAATGCCGGGGTTTTGTGATATCCAAGGAGGGCCGATCACG-ATCCAACCCAAAGATAACTCTGAGAAGGACCGTGCACGGATCAATGGAATCGGTGAA-CACAGGACCGGTTAAAGCCCTGGCTCACCGCGAGTGATGATCAA-TCGTTCACGGGTCATTCTGTTTTGGACAGGTT

grandiflorum_14 CATCGAAAATTTTGGGTCATCTT-AAAGCTCACAGACAAAAAGAGCAACCCGACTATAACAAGAGGCTTTT-GTTCACCACCGCTAGTCGGGACACTCTTGCTGCCCTTGGCTCGGATTTAGGCCAACCGCGTGCGGTACCACACGGGAGACCAACTTCCGTCCCG---TATCCAAGATGGGATGGGGGGACG----ACGTTTTGTGACACCCAGGCAGACGTGCCCTCGGCCCGAAGGCTTGAGGCGCAACTTGCGTTCAAAGACTCGATGGTTCACGGGATTCTGCAATTCACACCAAGTATCGCATTTCGCTACGTTCTTCATCGATGCGAGAGCCGAGATATCCGTTGCCGAGAGTCGTTTAGACTTTAGATACTGGCACC-ACGTTTGCATACACCGTCTCCGGGTTGCAAACGCAGACCATTTCGTTCAATGTTCCTTGGCACTTTTAATGCCGGGGTTTTGTGATATCCAAGGAAA-CCGATCACA-ATCCAACCCAAATATAACTCTGAGAAGGAC-GATCACGGATCCTTGGAGTCAGTGAA-CACAGGACCGGTTAAAGCCCTGGTTCACCGCGAGTGATGATCAA-TCGTTCACGGGTCATTCTGTTTTGGACAGGTT

heterocarpum_15 CATCGAAAAA-TTGGGGCATGTT-AAAGCACACGGACAACAAAGGCAACCCGACAATAACAAGAGGCTTTT-GTTCACCACCGCTAGTCGGGACAGTCTTGCTGCCCTTGGCTCGGATTTAGGCCAACCGCGTGCGGTAACACACGGGAGACCAACTTCCGTCCCT---TATCCAAGAT-GGATGGGGGGACG----ACGTTTTGTGACACCCAGGCAGACGTGCCCTCGGCCCGAAGGCTTGAGGCGCAACTTGCGTTCAAAGACTCGATGGTTCACGGGATTCTGCAATTCACACCAAGTATCGCATTTCGCTACGTTCTTCATCGATGCGAGAGCCGAGATATCCGTTGCCGAGAGTCGTTTAGACTTTAGATACTGGCACC-ACGTATGCACACACCGTCTCCGGGTTGCAAACGCGAACCATTTCGTTCAATGTTCCTTGGCACTTTTAATGCCGGGGTTTTGTGATATCCAAGGAGAGCCGATCACG-TTACAATCCAGAGAGAACCCTGAAAAGGACCGTGCGCGGATCCATGGAGTCGGTGAA-CGCAAAACCGGTTAAGGCTCTGGCTCACCGCGAGTGATGATCAA-TTGTTCACGGGTCATTCTGTTTTGGACAGGTT

huber-morathii_42 CATCGGAAATTTTGGGTCATCTT-AAAGCTCACGGACAAGAAGAGCAACCCGACTATAACAAGAGGCTTTT-GTTCACCACCGCTAGTCGGGACACTCTTGCTGCCCTTGGCTCGGATTTAGGCCAACCGCGTGCGGTACCACACGGGAGACCAACTTCCGTCCCG---TATCCAAGAT-GGATGGGGGGACG----ACGTTTTGTGACACCCAGGCAGACGTGCCCTCGGCCCGAAGGCTTGAGGCGCAACTTGCGTTCAAAGACTCGATGGTTCACGGGATTCTGCAATTCACACCAAGTATCGCATTTCGCTACGTTCTTCATCGATGCGAGAGCCGAGATATCCGTTGCCGAGAGTCGTTTAGACTTTAGATACTGGTACC-ACGTTTGCATACACCGTCTCCGGGTCGCAAACACAGACCATTTCGTTCAATGTTCCTTGGCACTTTTAATGCCGGGGTTTTGTGATATCCAAGGAAAGCCGATCACA-ATCCAACCCAAAGAGAACTCTGAGAAGGAC-GTTCGCGGATCCTTGGAGTCAGTGAA-CACAGGACCGGTTAAAGCCCTGGTTCACCGCGAGTGATGATCAA-TCGTTCACGGGTCATTCTGTTTTGGACAGGTT

karamanicum_43 CATCGAAAAATTTGGGTCATCTT-ATAGCTCACGGACAAGAAGAGCAGCCCGACTATAACAAGAGGCTTTT-GTTCACCACCGCTAGTCGGGACACTCTTGCTGCCCTTGGCTCGGATTTAGGCCAACCGCGTGCGGTACCACACGGGAGACCAACTTCCGTCCCG---TTTCCAAGAT-GGATGGGGGGACG----ACGTTTTGTGACACCCAGGCAGACGTGCCCTCGGCCCGAAGGCTTGAGGCGCAACTTGCGTTCAAAGACTCGATGGTTCACGGGATTCTGCAATTCACACCAAGTATCGCATTTCGCTACGTTCTTCATCGATGCGAGAGCCGAGATATCCGTTGCCGAGAGTCGTTTAGACTTTAGATACTGGCACC-ACGTTTGCATACACCGTCTCCGGGCCACAAACGCAGACCATTTCGTTCAATGTTCCTTGGCACTTTTAATGCCGGGGTTTTGTGATATCCAAGGAGAGCCGATGACG-ATCCAAACCAAAGATAACTCTGAGAAGGACCGTGCACGGATCAATGGAGTCGGTGAA-CACAGGACCGGTTAAAGCCCTTGCTCACCGCGAGTGATGATCAA-TCGTTCACGGGTCATTCTGTTTTGGACAGGTT

lepidioides_18 CATTGAAAACATTGGGTCATCTT-GAAGCTCACGGACAAGA---GCGAACCGACTGTAACAAGAGGTTTTT-GTTCACCACCGCTAGTCGGGACGCTCTTGCTGCCCTTGGCTCGGATTTAGGCCAACCGCGTACGGTACCACACGGGAGACCAGCTTCCGTCCCACTCTATCCAAGAT-GGATGGGGGGGGGGACGACGTTTTGTGACACCCAGGCAGACGTGCCCTCGGCCCGAAGGCTTTAGGCGCAACTTGCGTTCAAAGACTCGATGGTTCACGGGATTCTGCAATTCACACCAAGTATCGCATTTCGCTACGTTCTTCATCGATGCGAGAGCCGAGATATCCGTTGCCGAGAGTCGTTTAGACTTTACGTAGTGGCACC-GCGTTCGCACACACCGTCTCCGAGTTGCAAACGCGAGCCATTTAGTTGAATGTTCCTTGGCACTTTTAATGCCGGGGTTTTGTGATATCCA-TGCGACCCGGCCACG-ATCCAGCCCGGA---------GAGAAGGACCGAGCACGGATCCATGGAATCGGTGAAACACAAGACCGGTTAAGGCCCTGGCTCACCGCGAGTGATGATCTA-TCGTTCACGGGTCGTTCTGTTTGGGACAGGTA

Lycium_13 CATCGAAAAATTTGGGTCATGTT-AAAGCTCACAGACAAGAAGAGCAACCCGACTACAACAAGAGGCTTTT-GTTCACCACCGCTAGTCGGGACAGTCTTGCTGTCCTTGGCTCGGATTTAGGCCAACCGCGTGCGGTACCACACGGGAGACCAACTTCCGTCCCT---TATCCAAGAT-GGATGGGGGGACG----ACGTTTTGTGACACCCAGGCAGACGTGCCCTCGGCCCGAAGGCTTGAGGCGCAACTTGCGTTCAAAGACTCGATGGTTCACGGGATTCTGCAATTCACACCAAGTATCGCATTTCGCTACGTTCTTCATCGATGCGAGAGCCGAGATATCCGTTGCCGAGAGTCGTTTAGACTTTAGATACTGGCACC-ACGTATGCGCACACCGTCTCCGGGTTGCAAACGCGGACCATTTTGTTCAATGTTCCTTGGCACTTTTAATGCCGGGGTTTTGTGATATCCAAGGAGAACCGATCACG-ATACAATCCAGAGAGAACCCTGAAATGGACCGTACGCGGATCCATGGAGTCGGTGAA-CGCAAAACCGGTTAAGGCCCTGGCTTACCGCGAGTGATGATCAA-TTGTTCACGGGTCATTCTGTTTTGGACAGGTT

membranaceum_33Copen CATCGAAAATTTTGGGTCATCTT-AAAGCTCACAGACAAAAAGAGCAACCCGACTATAACAAGAGGCTTTT-GTTCACCACCGCTAGTCGGGACACTCTTGCTGCCCTTGTCTCGGATTTAGGCCAACCGCGTGCGGTACCACACGGGAGACCAACTTCCGTCCCG---TATCCAAGATGGGAT-GGGGGACG----ACGTTTTGTGACACCCAGGCAGACGTGCCCTCGGCCCGAAGGCTTGAGGCGCAACTTGCGTTCAAAGACTCGATGGTTCACGGGATTCTGCAATTCACACCAAGTATCGCATTTCGCTACGTTCTTCATCGATGCGAGAGCCGAGATATCCGTTGCCGAGAGTCGTTTAGACTTTAGATACTGGCACC-ACGTTTGCATACACCGTCTCCGGGTCGCAAACGCAGACCATTTCGTTCAATGTTCCTTGGCACTTTTAATGCCGGGGTTTTGTGATATCCAAGGAAAGCCGATCACA-ATCCAACCCAAATATAACTTTGAGAAGGAC-GATCACGGATCCTTGGAGTCAGTGAA-CACAGGACCGGTTAAAGCCCTGGTTCACCGCGAGTGATGATCAA-TCGTTCACGGGTCATTCTGTTTTGGACAGGTT

munzurense_46 CATCGAAAAAGTTGGGTCATGTT-AAAGCTGACGGACAAGAAGAGCAACCCGACTATAACAAGAGGCTTTT-GTTCACCACCGCTAGTCGGGACAGTCTTGCTGCCCTTGGCTCGGATTTAGGCCAACCGCGTGCGGTACCACACGGGAGACCAACTTCCGTCCCA---TATCCAAGAT-GGATGGGGGGACG----ACGTTTTGTGACACCCAGGCAGACGTGCCCTCGGCCCGAAGGCTTGAGGCGCAACTTGCGTTCAAAGACTCGATGGTTCACGGGATTCTGCAATTCACACCAAGTATCGCATTTCGCTACGTTCTTCATCGATGCGAGAGCCGAGATATCCGTTGCCGAGAGTCGTTTAGACTTTAGATACTGGCACC-ACGTATGCGCACACCGTCTCCGGGTTGCAAACGCGGACCATTTCGTTCAATGTTCCTTGGCACTTTTAATGCCGGGGTTTTGTGATATCCAAGGAGTGCCGATCACG-ATACAATCCAGAGAGAACCCTGAAATGGACCGTGCGCGGATCCATGGAGTCGGTGAA-CGCAAAACCGGTTAAGGCCTTGGCTCACCGCGAGTGATGATCAG-TTGTTCACGGGTCATTCTTTTTTGGACAGGTT

orbiculatum_33 -----AAATATTTGGGTCAAGTC-AAAGCTCACGGACAAGAAGAGCAACCCGACTATAACAAGAGGTTTTT-GTTCACCACCGCTAGTCGGGACAGTCTTGCTGCCCTTGGCTCGGATTTAGGCCAGCCGCGTGCGGTACCACACGGGAGACCAACTTCCGTCCCT---TATCCAAGAT-GGATGGGGGGACG----ACGTTTTGTGACACCCAGGCAGACGTGCCCTCGGCCCGAAGGCTTGAGGCGCAACTTGCGTTCAAAGACTCGATGGTTCACGGGATTCTGCAATTCACACCAAGTATCGCATTTCGCTACGTTCTTCATCGATGCGAGAGCCGAGATATCCGTTGCCGAGAGTCGTTTAGACTTTAGATACTGGCACC-ACGTATGCGCACACCGTCTCCGGGTTGCAAACGCGGGCCATTTCGTTCAATGTTCCTTGGCACTTTTAATGCCGGGGTTTTGTGATATCCAAGGAGAACCGATCACG-ATACAATCCAGAGAGAACCCTGAAAAGGACCGTGTGCGGATCCCTGGAGTCGGTGAA-CGCAAAACCGGTTAAGGCCCTGGCTCACCGCGAGTGATGATCAA-TTGTTCACGGGTCATTCTGTTTTGGACAGGTT

retsina_38 CATCGAAAAATTTGGGTCATGTT-GAAGCACACGGACAAGAAGAGCAACCCGACTATAACAAGAGGCTTTTTGTTCACCACCGCTAGTCGGGACAGTCTTGCTGCCCTTGGCTCGGATTTAGGCCAACCGCGTGCGGTACCACACGGGAGACCAACTTCCGTCCCA---TATCCAAGAT-GGATGGGGGGACG----ACGTTTTGTGACACCCAGGCAGACGTGCCCTCGGCCCGAAGGCTTGAGGCGCAACTTGCGTTCAAAGACTCGATGGTTCACGGGATTCTGCAATTCACACCAAGTATCGCATTTCGCTACGTTCTTCATCGATGCGAGAGCCGAGATATCCGTTGCCGAGAGTCGTTTAGACTTTAGATACTGGCACC-ACGTCTGCGCACACCATCTCCGGGTTGCAAACGTAGACCATTTCGTTCAATGTTCCTTGGCACTTTTAATGCCGGGGTTTTGTGATATCCAAGGAGAGCCGATCACG-ATACAATCCAGAGAGAACCCTGAAAAGGACCGTGCGCGGATCCACGGAGTCGGTGAA-CGCAAAACCGGTTAAGGATTTGGCTCACCACGAGTGATGATTAATTAGTTCACGGGTCATTCTGTTTTGGACAGGTT

rhodopaeum_24 CATCGAAAAATTTGGGTCATGTT-AAAGCTCGCGGACAAGAAGAGCAACCCGACTATAACAAGAGGCTTTT-GTTCACCACCGCTAGTCGGGACAGTCTTGCTGCCCTTGGCTCGGATTTAGGCCAACCGCATGCGGTACCACACGGGAGACCAACTTCCGTCCCT---TATCCAAGAT-GGATGGGGGGACG----ACGTTTTGTGACACCCAGGCAGACGTGCCCTCGGCCCGAAGGCTTGAGGCGCAACTTGCGTTCAAAGACTCGATGGTTCACGGGATTCTGCAATTCACACCAAGTATCGCATTTCGCTACGTTCTTCATCGATGCGAGAGCCGAGATATCCGTTGCCGAGAGTCGTTTAGACTTTAGATACTGGCACC-ACGTATGCGCACACCGTCTCCGGGTTGCAAACGCGGACCATTTCGTTCAATGTTCCTTGGCACTTTTAATGCCGGGGTTTTGTGATATCCAAGGAGAGCCGATCACG-ATACAATCCAGAGAGAACCCTGAAAAGGACCGTGCGCGGATCCATGGAGTCGGTGAA-CGCAAAACCGGTTAAGGCCAGGGCTCACCGCGAGTGATGATCAAATTGTTCACGGGTCATTCTGTTTTGGACAGGTT

saxatile_34 CATCGAAAA-TTTGGGTCATGTT-AAAGCTCACGGACAAGTAGAGCAACCCGACTATAACAAGAGGCTTTT-GTTCACCACCGCTAGTCGGGACAGTCTTGCTGCCCTTGGCTCGGATTTAGGCCAACCGCGTGCGGTACCACACGGGAGACCAACTTCCGTCCCT---TATCCAAGAT-AGATGGGGAGACG----ACGTTTTGTGACACCCAGGCAGACGTGCCCTCGGCCCGAAGGCTTGAGGCGCAACTTGCGTTCAAAGACTCGATGGTTCACGGGATTCTGCAATTCACACCAAGTATCGCATTTCGCTACGTTCTTCATCGATGCGAGAGCCGAGATATCCGTTGCCGAGAGTCGTTTAGACTTTAGATACTGGCACC-ACGTATGCGCACACCGTCTCCGGGTTGCAAACGCGGACCATTTCGTTCAATTTTCCTTGGCACTTTTAATGCCGGGGTTTTGTGATATCCAAGGAGAGCCGATCACG-ATACAATCCAGAGAGAACCCTGAAAAGGACCGTGCGCGGATCCGTGGAGTTGGTGAA-CGCAAAACCGGTTAAGGCCCTGGCTCACCGCGAGTGATGATCAA-TTGTTCACGGGTCATTCTGTTTTGGACAGGTT

schistosum_21 CATCGAAAAATTTGGGTCATCTT-AAAGCTCACGGACAAGAAGAGCAGCCCGACTATAACAAGAGGCTTTT-GTTCACCACCGCTAGTCGGGACACTCTTGCTGCCCTTGGCTCGGATTTAGGCCAACCGCGTGCGGTACCACACGGGAGACCAACTTCCGTCCCG---TTTCCAAGAT-GGATGGGGG-ACG----ACGTTTTGTGACACCCAGGCAGACGTGCCCTCGGCCCGAAGGCTTGAGGCGCAACTTGCGTTCAAAGACTCGATGGTTCACGGGATTCTGCAATTCACACCAAGTATCGCATTTCGCTACGTTCTTCATCGATGCGAGAGCCGAGATATCCGTTGCCGAGAGTCGTTTAGACTTTAGATACTGGCACC-ACGTTTGCATACACCGTCTCCGGGTCACAAACGTAGACCATTTCGTTCAATGTTCCTTGGCACTTTTAATGCCGGGGTTTTGTGATATCCAAGGAGGGCCGATCACG-ATCCAACCCAAAGATAACTCTGAGAAGGACCGTGCACGGATCAATGGAGTCGGTGAA-CACAGGACCGGTTAAAGCCCTGGCTCACCTCGAGTGATGATCAA-TCGTTCACGGGTCATTCTGTTTTGGACAGGTT

semnanense_50 CATCGAAAAATTTGGGTCATGTT-GAAGCTCACAGACAAGAAGAGCAACCCGACTATAACAAGAGGCGTTT-GTTCACCACCGCTAGTCGGGACAGTCTTGCTGCCCTTGGCTCGGATTTAGGCCAACCGCGTGCGGTACCACACGGGAGACCAACTTCCGTCCCT---TATCCAAGAT-GGATGGGGGGACG----ACGTTTTGTGACACCCAGGCAGACGTGCCCTCGGCCCGAAGGCTTGAGGCGCAACTTGCGTTCAAAGACTCGATGGTTCACGGGATTCTGCAATTCACACCAAGTATCGCATTTCGCTACGTTCTTCATCGATGCGAGAGCCGAGATATCCGTTGCCGAGAGTCGTTTAGACTTTAGATACTGGCACC-ACGTATGTGCACTCCGTCTCCGGGTTGCAAACGC-GACCATTTCGTTCAATGTTCCTTGGCACTTTTAATGCCGGGGTTTTGTGATATCCAAGGAGAGTCAACCACG-ATACAATCCAGAGAGAACCCTGAAAAGGACCGTGCATGGATCCATGGAGTCGGTGAA-CGCAAAACCGGTTAAGGCCTTGGCTCACCGCGAGTGATGATCAA-TTGTTCACGGGTCATTCTGTTTTGGACAGGTT

sintenisii_48 CATCGAAAATTTTGGGTCATCTT-AAAGCTCACAGACAAAAAGAGCAACCCGACTATAACAAGAGGCTTTT-GTTCACCACCGCTAGTCGGGACACTCTTGCTGCCCTTGTCTCGGATTTAGGCCAACCGCGTGCGGTACCACACGGGAGACCAACTTCCGTCCCG---TATCCAAGATGGGAT-GGGGGACG----ACGTTTTGTGACACCCAGGCAGACGTGCCCTCGGCCCGAAGGCTTGAGGCGCAACTTGCGTTCAAAGACTCGATGGTTCACGGGATTCTGCAATTCACACCAAGTATCGCATTTCGCTACGTTCTTCATCGATGCGAGAGCCGAGATATCCGTTGCCGAGAGTCGTTTAGACTTTAGATACTGGCACC-ACGTTTGCATACACCGTCTCCGGGTCGCAAACGCAGACCATTTCGTTCAATGTTCCTTGGCACTTTTAATGCCGGGGTTTTGTGATATCCAAGGAAATCCGATCACA-ATCCAACCCAAAGAGGACTCTGAGAAGGAC-GTTCGCGGATCCTTGGAGTCAGTGAA-CACAGGACCGGTTAAAGCCTTGGTTCACCGCGAGTGATGATCAA-TCGTTCACGGGTCATTCTGTTTTGGACAGGTT

spicatum_35 CATCGAAAAATTTGGGTCATCTT-GAAGCTCACGGACAAGAAGAGTAACCCGACTATAACAAGAGGCTTTT-GTTCACCACCGCTAGTCGGGACACTCTTGCTGCCCTTGGCTCGGATTTAGGCCAACCGCGTGCGGTACCACACGGGAGACCAACTTCCGTCCCG---TATCCAAGAT-GGATGGGGGGACG----ACGTTTTGTGACACCCAGGCAGACGTGCCCTCGGCCCGAAGGCTTAAGGCGCAACTTGCGTTCAAAGACTCGATGGTTCACGGGATTCTGCAATTCACACCAAGTATCGCATTTCGCTACGTTCTTCATCGATGCGAGAGCCGAGATATCCGTTGCCGAGAGTCGTTTAGACTTTAGATACTGGCACC-ACGTTTGCATACACCGTCTCCGGGTCGCAAACGCAGACCATTTCGTTCAATGTTCCTTGGCACTTTTAATGCCGGGGTTTTGTGATATCCAAGGAGAGCCGATCACG-ATCCAACCCAAAGATAACTCTGAGGAGGACCGTGCACGGATCCATGGAGTCGGTGAA-CACAAGACCGGTTAAAGCCTTGGCTCACCGCGAGTGATGATCAA-TCGTTCACGGGTCATTCTGTTTTGGACAGGTT

spinosum_23 CATTGAAGACTTTGGGTCATCTT-AAAGCTCACGAACAAGA---GCGAACCGACCGTAACAAGAGGTTTTT-GTTCACCACCGCTAGTCGGGACGCTCTTGCTGCCCTTGGCTCGGATTTAGGCCAACCGCGTGCGGTACCACACGGGAGACCAGCTTCCGTCCCT---TATCCGAGAT-GGATGGGGGGGGGGACGACGTTTTGTGACACCCAGGCAGACGTGCCCTCGGCCCGAAGGCTTTAGGCGCAACTTGCGTTCAAAGACTCGATGGTTCACGGGATTCTGCAATTCACACCAAGTATCGCATTTCGCTACGTTCTTCATCGATGCGAGAGCCGAGATATCCGTTGCCGAGAGTCGTTTAGACTTTACATACTGGCATACGCATTTGCGCACACCGTCTCCGGGTTGCAAACGCGAGCCATTTAGTTGAATGTTCCTTGGCACTTTTAATGCCGGGGTTTTGTGATATCCA-TGCGACCCAGCCACA-ATCCAGCCCGTA---------GAGAAGGACCGAGCACGGATCCATGGACTCGGTGAA-CGCAAGACCGGTTAAAGCCCTGGCACACCGCGAGTGATGATCTA-TCGTTCACGGGTCGTGCTGTTTGGGACAGGTA

stenopterum_31 CATCGAAAATTTTGGGTCATCTT-AAAGCTCATGGACAAGAAGAGCAACCCGACTATAACAAGAGGCTTTT-GTTCACCACCGCTAGTCGGGACACTCTTGCTGCCCTTGGCTCGGATTTAGGCCAACCGCGTGCGGTACCACACGGGAGACCAACTTCCGTCCCG---TATCCAAGAT-GGATGGGGGGACG----ACGTTTTGTGACACCCAGGCAGACGTGCCCTCGGCCCGAAGGCTTGAGGCGCAACTTGCGTTCAAAGACTCGATGGTTCACGGGATTCTGCAATTCACACCAAGTATCGCATTTCGCTACGTTCTTCATCGATGCGAGAGCCGAGATATCCGTTGCCGAGAGTCGTTTAGACTTTAGATACTGGCACC-ACGTTTGCATACACCGTCTCCGGGTCACAAACGCAGACCATTTCGTTCAATGTTCCTTGGCACTTTTAATGCCGGGGTTTTGTGATATCCAAGGAAATCCGATCACA-ATCCAACCCAAAGAGGACTCTGAGAAGGAC-GTTCGCGGATCCTTGGAGTCAGTGAA-CACAAGACCGGTTAAAGCCCTGGTTCACCGCGAGTGATGATCAA-TCGTTCACGGGTCATTCTGTTTTGGACAGGTT

stylosum_19 CATCGAAAAAATTGGGTCATGTT-AAAGCTCACGGACAAGAAGAGCAACCCGACTATAACAAGAGGCTTTT-GTTCACCACCGCTAGTCGGGACAGTCTTGCTGCCCTTGGCTCGGATTTAGGCCAACCGCGTGCGGTACCACACGGGAGACCAACTTCCGTCCCT---TGTCCAAGAT-GGATGGGGGGACG----ACGTTTTGTGACACCCAGGCAGACGTGCCCTCGGCCCGAAGGCTTGAGGCGCAACTTGCGTTCAAAGACTCGATGGTTCACGGGATTCTGCAATTCACACCAAGTATCGCATTTCGCTACGTTCTTCATCGATGCGAGAGCCGAGATATCCGTTGCCGAGAGTCGTTTAGACTTTAGATACTAGCACC-ACGTATGCGCACACCGTCTCCGGGTTGCAAACGCAGACCATTTCGTTCAATGTTCCTTGGCACTTTTAATGCCGGGGTTTTGTGATATCCAAAGAGAGCCGATCACG-ATACAGTCCAGAGAGAACCCTGAAAAGGACCGTGCGCGGATCCATGGAGTCGGTGAA-CGCAAAACCGGTTAAGGCCTTGGCTCACCGCGAGTGATGATCAG-TTGTTCACGGGTCATTCTGTTTTGGACAGGTT

subulatum_20 CATCGAAAAATTTGGGTCATCTT-AAAGCTCACGGACAAGAAGAGCAGCCCGACTATAACAAGAGGCTTTT-GTTCACCACCGCTAGTCGGGACACTCTTGCTGCCCTTGGCTCGGATTTAGGCCAACCGCGTGCGGTACCACACGGGAGACCAACTTCCGTCCCG---TTTCCAAGAT-GGATGGGGGGACG----ACGTTTTGTGACACCCAGGCAGACGTGCCCTCGGCCCGAAGGCTTGAGGCGCAACTTGCGTTCAAAGACTCGATGGTTCACGGGATTCTGCAATTCACACCAAGTATCGCATTTCGCTACGTTCTTCATCGATGCGAGAGCCGAGATATCCGTTGCCGAGAGTCGTTTAGACTTTAGATACTGGCACC-ACGTTTGCATACACCGTCTCCGGGTCGCAAACGCAGACCATTTCGTTCAATGTTCCTTGGCACTTTTAATGCCGGGGTTTTGTGATATCCAAGGAGGGCCGATCACG-ATCCAACCCAAAGATAACTCTGAGAAGGACCGTGCACGGATCAATGGAGTCGGTGAA-CACAGGACCGGTTAAAGCCCTGGCTCACCGCGAGTGATGATCAA-TCGTTCACGGGTCATTCTGTTTTGGACAGGCT

syriacum_27 CATCGAAAAATTTGGGTCATGTT-AAAGCTCGCGGACAAGAAGAGCAACCCGACTATAACAAGAGGCTTTT-GTTCACCACCGCTAGTCGGGACAGTCTTGCTGCCCTTGGCTCGGATTTAGGCCAACCGCATGCGGTACCACACGGGAGACCAACTTCCGTCCCT---TATCCAAGAT-GGATGGGGGGACG----ACGTTTTGTGACACCCAGGCAGACGTGCCCTCGGCCCGAAGGCTTGAGGCGCAACTTGCGTTCAAAGACTCGATGGTTCACGGGATTCTGCAATTCACACCAAGTATCGCATTTCGCTACGTTCTTCATCGATGCGAGAGCCGAGATATCCGTTGCCGAGAGTCGTTTAGACTTTAGATACTGGCACC-ACGTATGCGCACACCGTCTCCGGGTTGCAAACGCGGACCATTTCGTTCAATGTTCCTTGGCACTTTTAATGCCGGGGTTTTGTGATATCCAAGGAGAGCCGATCACG-ATACAATCCAGAGAGAACCCTGAAAAGGACCGTGCGCGGATCCATGGAGTCGGTGAA-CGCAAAACCGGTTAAGGCCAGGGCTCACCGCGAGTGATGATCAAATTGTTCACGGGTCATTCTGTTTTGGACAGGTT

Szowitsii_51 CATCAAAAATTTTGGGTCATCTT-AAAGCTCACGGACAAGAAGAGCAACCCGACTATAACAAGAGGCTTTT-GTTCACCACCGCTTGTCGGGACACTCTTGCTGCCCTTGGCTTGGATTTAGGCCAACCGCATGCGGTACCACACGGGAGACCAACTTCCGTCCCG---TATCCAAGAT-GGATGGGGGGACG----ACGTTTTGTGACACCCAGGCAGACGTGCCCTCGGCCCGAAGGCTTAAGGCGCAACTTGCGTTCAAAGACTCGATGGTTCACGGGATTCTGCAATTCACACCAAGTATCGCATTTCGCTACGTTCTTCATCGATGCGAGAGCCGAGATATCCGTTGCCGAGAGTCGTTTAGACTTTAGATACTGGCACC-ACGTTTGCATACACCGTCTCCGGGTCGCAAATGCAGACCATTTCGTTCAATGTTCCTTGGCACTTTTAATGCCGGGGTTTTGTGATATCCAAGGAAAGCCGATCACA-ATCCAACCCAAAGAGAACTCTGAGAAGGAC-GTTCGCGGATCCTTGGAGTCAGTGAA-CACAGGACCGGTTAAAGCCCTGGTTCACCGCAAGTGATGATCAA-TCGTTCACGGGTCATTCTGTTTTGGACAGGTT

thomasianum_54 CATCGAAAAATTTGGGTCATCTT-AAAGCTCACGGACAAGAAGAGGAACCCGACTATAACAAGAGGCTTTT-GTTCACCACCGCTAGTCGGGACACTCTTGTTGCCCTTTGCTCGGATTTAGGCCAACCGCGTGCGGTACCACACGGGAGACCAACTTCCGTCCCG---TATCCAAGAT-GGATGGGGGGACG----ACGTTTTGTGACACCCAGGCAGACGTGCCCTCGGCCCGAAGGCTTGAGGCGCAACTTGCGTTCAAAGACTCGATGGTTCACGGGATTCTGCAATTCACACCAAGTATCGCATTTCGCTACGTTCTTCATCGATGCGAGAGCCGAGATATCCGTTGCCGAGAGTCGTTTAGACTTTAGATACTGGCACC-ACGTTTGCATACACCGTCTCCGGGTCACAAACGCAGACCATTTCGTTCAATGTTCCTTGGCACTTTTAATGCCGGGGTTTTGTGATATCCAAGGAGAGCCGATCATG-ATCCAACCCAAAGAGAACTCTGAGAAGGACCGTGCGCGGATCCATGGAGTCGGTGAA-CACAAGACCGGTTAAAGCCCTGGTTCACCGCGAGTGATGATCAA-TCGTTCACGGGTCATTCTGTTTTGGACAGGTT

turcicum_1 CATCGAAAAAGTTGGGTCATGTT-AAAGCTCACGGACAAGAAGAGCAACCCGACTATAACAAGAGGCTTTT-GTTCACCACCGCTAGTCGGGACAGTCTTGCTGCCCTTGGCTCGGATTTAGGCCAACCGCGTGCGGTACCACACGGGAGACCAACTTCCGTCCCT---TATCCAAGAT-GGATGGGGGGACG----ACGTTTTGTGACACCCAGGCAGACGTGCCCTCGGCCCGAAGGCTTGAGGCGCAACTTGCGTTCAAAGACTCGATGGTTCACGGGATTCTGCAATTCACACCAAGTATCGCATTTCGCTACGTTCTTCATCGATGCGAGAGCCGAGATATCCGTTGCCGAGAGTCGTTTAGACTTTAGATACTGGCACC-ACGTATGCGCACACCGTCTCCGGGTTGCAAACGTGGACCATTTCGTTCAATGTTCCTTGGCACTTTTAATGCCGGGGTTTTGTGATATCCAAGGAGAGCCGATCACG-ATACAGTCCAGAGAGAACCCTGAAAAGGACCGTGCGCGGATCCATGGAGTCGGTGAA-CGCAAAACCGGTTAAGGCTCTGGCTCACCGCGAGTGATGATCAC-TTGTTCACGGGTCATTCTGTTTTGGACAGGTT

umbellatum_32 CATCGAAAATTTTGGGTCATCTT-AAAGCTCACGGACAAGAAGAGCAACCCGACTATAACAAGAGGCTTTT-GTTCACCACCGCTAGTCGGGACACTCTTGCTGCCCTTGGCTCGGATTTAGGCCAACCGCGTGCGGTACCACACGGGAGACCAACTTCCGTCCCG---TATCCACGATGGGATGGGGGGACG----ACGTTTTGTGACACCCAGGCAGACGTGCCCTCGGCCCGAAGGCTTGAGGCGCAACTTGCGTTCAAAGACTCGATGGTTCACGGGATTCTGCAATTCACACCAAGTATCGCATTTCGCTACGTTCTTCATCGATGCGAGAGCCGAGATATCCGTTGCCGAGAGTCGTTTAGACTTTAGATACTGGCACA-ACGTTTGCATACACCGTCTCCGGGTCGCAAACGTAGACCATTTCGTTCAATGTTCCTTGGCACTTTTAATGCCGGGGTTTTGTGATATCCAAGGAAAGCCGATCACA-ATCCAGCCCAAATATAACTCTGAGAAGGAC-GATCACGGATCCTTGGAGTCAGTGAA-CACAGGACCGGTTAAAGCCCTGGTTCACTGCGAGTGATGATCAA-TCGTTCACGGGTCATTCTGTTTTGGACAGGTT

virgatum_26 CATCAAAAATTTTGGGTCATCTT-AAAGCTCACGGACAAGAAGAGCAACCCGACTATAACAAGAGGCTTTT-GTTCACCACCGCTTGTCGGGACACTCTTGCTGCCCTTGGCTTGGATTTAGGCCAACCGCATGCGGTACCACACGGGAGACCAACTTCCGTCCCG---TATCCAAGAT-GGATGGGGGGACG----ACGTTTTGTGACACCCAGGCAGACGTGCCCTCGGCCCGAAGGCTTAAGGCGCAACTTGCGTTCAAAGACTCGATGGTTCACGGGATTCTGCAATTCACACCAAGTATCGCATTTCGCTACGTTCTTCATCGATGCGAGAGCCGAGATATCCGTTGCCGAGAGTCGTTTAGACTTTAGATACTGGCACC-ACGTTTGCATACACCGTCTCCGGGTCGCAAATGCAGACCATTTCGTTCAATGTTCCTTGGCACTTTTAATGCCGGGGTTTTGTGATATCCAAGGAAAGCCGATCACA-ATCCAACCCAAAGAGAACTCTGAGAAGGAC-GTTCGCGGATCCTTGGAGTCAGTGAA-CACAGGACCGGTTAAAGCCCTGGTTCACCGCAAGTGATGATCAA-TTGTTCACGGGTCATTCTGTTTTGGACAGGTT

;

end;

begin mrbayes;

set autoclose=yes nowarn=yes;

lset nst=6 rates=gamma;

unlink statefreq=(all) revmat=(all) shape=(all) pinvar=(all);

prset applyto=(all) ratepr=variable;

mcmcp ngen=20000000 nruns=4 nchains=4 temp=0.2 samplefreq=1000 savebrlens=yes printfreq=1000;

mcmc;

sump burninfrac=0.1;

sumt burninfrac=0.1 contype=halfcompat conformat=Figtree outputname=Aethionema_16_1_2023_ITS.tre;

end;
