# Supplementary material for: Underestimated diversity in high elevations of a global biodiversity hotspot: two new endemic species of Aethionema (Brassicaceae) from the alpine zone of Iran
Source: Front Plant Sci. 2023 May 26;14:1182073. doi: 10.3389/fpls.2023.1182073 (PMC10250747; doi:10.3389/fpls.2023.1182073)
Supplement: Supplementary file 2 [file DataSheet_2.zip › Date Sheet 2/ITS/JModeltest_output.docx]

-------------------------- jModeltest 2.1.10 v20160303 --------------------------

(c) 2011-onwards D. Darriba, G.L. Taboada, R. Doallo and D. Posada,

(1) Department of Biochemistry, Genetics and Immunology

University of Vigo, 36310 Vigo, Spain.

(2) Department of Electronics and Systems

University of A Coruna, 15071 A Coruna, Spain.

e-mail: ddarriba@udc.es, dposada@uvigo.es

--------------------------------------------------------------------------------

Thu Dec 29 23:03:50 PST 2022

Linux 4.18.0-348.23.1.el8_5.x86_64, arch: amd64, bits: 64, numcores: 32

--------------------------------------------------------------------------------

Citation: Darriba D, Taboada GL, Doallo R and Posada D. 2012.

"jModelTest 2: more models, new heuristics and parallel computing".

Nature Methods 9(8), 772.

--------------------------------------------------------------------------------

jModelTest 2.1.10 v20160303

Copyright (C) 2011 D. Darriba, G.L. Taboada, R. Doallo and D. Posada

This program comes with ABSOLUTELY NO WARRANTY

This is free software, and you are welcome to redistribute it under certain

conditions

Notice: This program may contain errors. Please inspect results carefully.

Arguments = -tr 32 --set-property log-dir=./ -d infile.phy -o output.txt -s 11 -f -i -g 4 -t ML -AIC -AICc -BIC -DT -S BEST -p -a -w

Reading data file "infile.phy"... OK.

number of sequences: 61

number of sites: 636

---------------------------------------------------------------

* *

* COMPUTATION OF LIKELIHOOD SCORES WITH PHYML *

* *

---------------------------------------------------------------

::Settings::

Phyml version = 20130103

Phyml binary = PhyML_3.0_linux64

Phyml path = /expanse/projects/ngbt/opt/expanse/jmodeltest2/2.1.10/exe/phyml/

Candidate models = 88

number of substitution schemes = 11

including models with equal/unequal base frequencies (+F)

including models with/without a proportion of invariable sites (+I)

including models with/without rate variation among sites (+G) (nCat = 4)

Optimized free parameters (K) = substitution parameters + 119 branch lengths + topology

Base tree for likelihood calculations = ML tree

Tree topology search operation = BEST

::Progress::

Model Exec. Time Total Time -lnL

-------------------------------------------------------------------------

F81+G 00h:00:33:03 00h:00:33:03 3076.8102 (1/88)

TrN+G 00h:00:34:02 00h:00:34:03 2980.3871 (2/88)

TPM1uf+G 00h:00:34:03 00h:00:34:03 2986.9305 (3/88)

TPM3uf+G 00h:00:34:04 00h:00:34:04 2986.5251 (4/88)

TPM2uf+G 00h:00:34:06 00h:00:34:06 2981.9063 (5/88)

TIM2+G 00h:00:35:03 00h:00:35:04 2977.3869 (6/88)

TIM3+G 00h:00:36:07 00h:00:36:07 2981.7701 (7/88)

TVM+G 00h:00:36:06 00h:00:36:08 2981.4184 (8/88)

HKY+G 00h:00:44:05 00h:00:44:06 2986.9719 (9/88)

TIM1+G 00h:00:53:01 00h:00:53:02 2979.6701 (10/88)

TPM1+I+G 00h:00:59:04 00h:00:59:04 2988.4352 (11/88)

K80+I+G 00h:01:00:06 00h:01:00:07 2988.4582 (12/88)

TPM3uf+I+G 00h:01:04:01 00h:01:04:02 2984.4766 (13/88)

JC+I+G 00h:01:04:06 00h:01:04:07 3078.5135 (14/88)

TIM3+I+G 00h:01:04:08 00h:01:04:08 2979.1939 (15/88)

TPM2uf+I+G 00h:01:04:08 00h:01:04:08 2980.1215 (16/88)

TPM2+I+G 00h:01:05:00 00h:01:05:00 2982.2050 (17/88)

TPM3+I+G 00h:01:05:08 00h:01:05:09 2986.6360 (18/88)

JC+G 00h:00:32:01 00h:01:06:04 3078.6302 (19/88)

K80+G 00h:00:33:06 00h:01:07:09 2989.2683 (20/88)

HKY+I+G 00h:01:07:09 00h:01:08:00 2985.9674 (21/88)

TPM1+G 00h:00:33:04 00h:01:08:01 2989.2390 (22/88)

TPM1uf+I+G 00h:01:08:02 00h:01:08:02 2985.9339 (23/88)

TPM2+G 00h:00:33:07 00h:01:09:01 2983.8497 (24/88)

TIM2ef+I+G 00h:01:13:01 00h:01:13:02 2975.3564 (25/88)

TIM3ef+I+G 00h:01:13:08 00h:01:13:09 2980.1693 (26/88)

TrNef+I+G 00h:01:14:03 00h:01:14:03 2980.9603 (27/88)

TIM1ef+I+G 00h:01:14:04 00h:01:14:04 2981.1262 (28/88)

TVMef+I+G 00h:01:14:08 00h:01:14:08 2981.6594 (29/88)

TrNef+G 00h:00:43:01 00h:01:17:05 2979.8109 (30/88)

TPM3+G 00h:00:41:05 00h:01:18:02 2988.5907 (31/88)

TrN+I+G 00h:01:18:04 00h:01:18:05 2980.0677 (32/88)

F81+I 00h:00:14:05 00h:01:18:06 3090.4189 (33/88)

TIM1ef+G 00h:00:42:06 00h:01:19:04 2979.7881 (34/88)

HKY+I 00h:00:15:01 00h:01:19:08 3000.8585 (35/88)

TPM1uf+I 00h:00:15:04 00h:01:20:02 3000.8217 (36/88)

SYM+I+G 00h:01:21:00 00h:01:21:01 2974.6703 (37/88)

TIM1+I 00h:00:15:00 00h:01:21:04 2996.0835 (38/88)

TPM3uf+I 00h:00:15:08 00h:01:21:07 3000.6350 (39/88)

TVM+I+G 00h:01:21:07 00h:01:21:08 2979.4793 (40/88)

TPM2uf+I 00h:00:17:05 00h:01:22:06 2995.7711 (41/88)

TrN+I 00h:00:18:01 00h:01:23:00 2996.2350 (42/88)

F81+I+G 00h:01:23:03 00h:01:23:03 3073.2145 (43/88)

TIM3+I 00h:00:15:04 00h:01:23:04 2995.9998 (44/88)

GTR+I+G 00h:01:23:05 00h:01:23:05 2972.4569 (45/88)

TVM+I 00h:00:15:04 00h:01:23:05 2995.5184 (46/88)

TIM2ef+G 00h:00:39:08 00h:01:24:03 2977.6013 (47/88)

TIM2+I 00h:00:17:05 00h:01:25:05 2991.3452 (48/88)

GTR+I 00h:00:17:04 00h:01:25:06 2987.7100 (49/88)

TIM1+I+G 00h:01:25:07 00h:01:25:07 2980.3571 (50/88)

TIM2+I+G 00h:01:26:02 00h:01:26:03 2975.1922 (51/88)

GTR+G 00h:00:53:00 00h:01:26:04 2972.7904 (52/88)

JC+I 00h:00:20:00 00h:01:29:01 3091.9818 (53/88)

K80+I 00h:00:16:02 00h:01:29:04 3003.0489 (54/88)

TPM1+I 00h:00:15:02 00h:01:29:05 3003.3245 (55/88)

TrNef+I 00h:00:16:02 00h:01:30:01 2996.1016 (56/88)

TPM2+I 00h:00:16:02 00h:01:30:06 2997.9754 (57/88)

TPM3+I 00h:00:16:01 00h:01:31:00 3002.9178 (58/88)

TrN 00h:00:09:09 00h:01:31:00 3058.5267 (59/88)

HKY 00h:00:11:01 00h:01:31:03 3066.3507 (60/88)

TIM1ef+I 00h:00:14:01 00h:01:31:06 2996.9258 (61/88)

F81 00h:00:12:01 00h:01:31:09 3157.1132 (62/88)

TPM2uf 00h:00:10:09 00h:01:32:06 3062.8133 (63/88)

TPM1uf 00h:00:11:04 00h:01:32:09 3066.1534 (64/88)

TIM3ef+I 00h:00:14:05 00h:01:33:00 2996.3908 (65/88)

TIM3ef+G 00h:00:39:09 00h:01:33:01 2980.4769 (66/88)

TIM1 00h:00:11:01 00h:01:33:07 3058.3324 (67/88)

TPM3uf 00h:00:12:00 00h:01:33:08 3066.0681 (68/88)

GTR 00h:00:10:02 00h:01:33:08 3054.7024 (69/88)

TIM2ef+I 00h:00:16:00 00h:01:34:02 2991.8204 (70/88)

TIM2 00h:00:11:06 00h:01:34:06 3055.1310 (71/88)

TIM3 00h:00:11:02 00h:01:34:06 3058.1977 (72/88)

TVM 00h:00:11:06 00h:01:35:00 3062.4265 (73/88)

JC 00h:00:12:03 00h:01:35:08 3161.4404 (74/88)

TVMef+I 00h:00:17:02 00h:01:35:09 2997.5092 (75/88)

SYM+I 00h:00:16:06 00h:01:36:00 2990.3462 (76/88)

TVMef+G 00h:00:36:09 00h:01:36:03 2983.1337 (77/88)

TIM1ef 00h:00:11:01 00h:01:37:05 3063.3669 (78/88)

K80 00h:00:13:02 00h:01:37:05 3075.5721 (79/88)

TPM1 00h:00:12:07 00h:01:38:03 3075.5464 (80/88)

TPM2 00h:00:12:06 00h:01:38:03 3071.3384 (81/88)

TPM3 00h:00:12:07 00h:01:38:09 3075.2342 (82/88)

TrNef 00h:00:13:06 00h:01:39:01 3064.8778 (83/88)

TIM3ef 00h:00:11:01 00h:01:40:05 3062.9247 (84/88)

SYM 00h:00:10:09 00h:01:41:00 3059.4516 (85/88)

TVMef 00h:00:12:09 00h:01:42:04 3070.9952 (86/88)

TIM2ef 00h:00:13:03 00h:01:42:04 3060.6676 (87/88)

SYM+G 00h:00:53:08 00h:01:54:05 2972.8048 (88/88)

Model = JC

partition = 000000

-lnL = 3161.4404

K = 120

Model = JC+I

partition = 000000

-lnL = 3091.9818

K = 121

p-inv = 0.5880

Model = JC+G

partition = 000000

-lnL = 3078.6302

K = 121

gamma shape = 0.3390

Model = JC+I+G

partition = 000000

-lnL = 3078.5134

K = 122

p-inv = 0.1960

gamma shape = 0.5130

Model = F81

partition = 000000

-lnL = 3157.1132

K = 123

freqA = 0.2453

freqC = 0.2548

freqG = 0.2183

freqT = 0.2816

Model = F81+I

partition = 000000

-lnL = 3090.4189

K = 124

freqA = 0.2427

freqC = 0.2563

freqG = 0.2153

freqT = 0.2857

p-inv = 0.5900

Model = F81+G

partition = 000000

-lnL = 3076.8102

K = 124

freqA = 0.2426

freqC = 0.2581

freqG = 0.2145

freqT = 0.2848

gamma shape = 0.3340

Model = F81+I+G

partition = 000000

-lnL = 3073.2145

K = 125

freqA = 0.2402

freqC = 0.2593

freqG = 0.2142

freqT = 0.2863

p-inv = 0.2030

gamma shape = 0.5170

Model = K80

partition = 010010

-lnL = 3075.5721

K = 121

kappa = 4.5180 (ti/tv = 2.2590)

Model = K80+I

partition = 010010

-lnL = 3003.0489

K = 122

kappa = 4.6460 (ti/tv = 2.3230)

p-inv = 0.5920

Model = K80+G

partition = 010010

-lnL = 2989.2683

K = 122

kappa = 4.7690 (ti/tv = 2.3845)

gamma shape = 0.3290

Model = K80+I+G

partition = 010010

-lnL = 2988.4582

K = 123

kappa = 4.6843 (ti/tv = 2.3421)

p-inv = 0.2120

gamma shape = 0.5180

Model = HKY

partition = 010010

-lnL = 3066.3507

K = 124

freqA = 0.2446

freqC = 0.2545

freqG = 0.2197

freqT = 0.2812

kappa = 4.5511 (ti/tv = 2.2927)

Model = HKY+I

partition = 010010

-lnL = 3000.8585

K = 125

freqA = 0.2469

freqC = 0.2528

freqG = 0.2243

freqT = 0.2760

kappa = 4.6684 (ti/tv = 2.3449)

p-inv = 0.5900

Model = HKY+G

partition = 010010

-lnL = 2986.9719

K = 125

freqA = 0.2480

freqC = 0.2551

freqG = 0.2238

freqT = 0.2731

kappa = 4.6881 (ti/tv = 2.3547)

gamma shape = 0.3330

Model = HKY+I+G

partition = 010010

-lnL = 2985.9674

K = 126

freqA = 0.2460

freqC = 0.2568

freqG = 0.2233

freqT = 0.2740

kappa = 4.6015 (ti/tv = 2.3144)

p-inv = 0.2080

gamma shape = 0.5200

Model = TrNef

partition = 010020

-lnL = 3064.8778

K = 122

R(a) [AC] = 1.0000

R(b) [AG] = 3.1522

R(c) [AT] = 1.0000

R(d) [CG] = 1.0000

R(e) [CT] = 5.8907

R(f) [GT] = 1.0000

Model = TrNef+I

partition = 010020

-lnL = 2996.1015

K = 123

R(a) [AC] = 1.0000

R(b) [AG] = 3.3745

R(c) [AT] = 1.0000

R(d) [CG] = 1.0000

R(e) [CT] = 6.0166

R(f) [GT] = 1.0000

p-inv = 0.5790

Model = TrNef+G

partition = 010020

-lnL = 2979.8108

K = 123

R(a) [AC] = 1.0000

R(b) [AG] = 3.1763

R(c) [AT] = 1.0000

R(d) [CG] = 1.0000

R(e) [CT] = 6.1648

R(f) [GT] = 1.0000

gamma shape = 0.3520

Model = TrNef+I+G

partition = 010020

-lnL = 2980.9603

K = 124

R(a) [AC] = 1.0000

R(b) [AG] = 3.3291

R(c) [AT] = 1.0000

R(d) [CG] = 1.0000

R(e) [CT] = 6.2115

R(f) [GT] = 1.0000

p-inv = 0.2110

gamma shape = 0.5490

Model = TrN

partition = 010020

-lnL = 3058.5267

K = 125

freqA = 0.2641

freqC = 0.2384

freqG = 0.2357

freqT = 0.2619

R(a) [AC] = 1.0000

R(b) [AG] = 3.2076

R(c) [AT] = 1.0000

R(d) [CG] = 1.0000

R(e) [CT] = 5.8979

R(f) [GT] = 1.0000

Model = TrN+I

partition = 010020

-lnL = 2996.2350

K = 126

freqA = 0.2625

freqC = 0.2402

freqG = 0.2357

freqT = 0.2617

R(a) [AC] = 1.0000

R(b) [AG] = 3.3528

R(c) [AT] = 1.0000

R(d) [CG] = 1.0000

R(e) [CT] = 5.8271

R(f) [GT] = 1.0000

p-inv = 0.5780

Model = TrN+G

partition = 010020

-lnL = 2980.3871

K = 126

freqA = 0.2639

freqC = 0.2416

freqG = 0.2364

freqT = 0.2582

R(a) [AC] = 1.0000

R(b) [AG] = 3.3025

R(c) [AT] = 1.0000

R(d) [CG] = 1.0000

R(e) [CT] = 6.1403

R(f) [GT] = 1.0000

gamma shape = 0.3530

Model = TrN+I+G

partition = 010020

-lnL = 2980.0677

K = 127

freqA = 0.2639

freqC = 0.2416

freqG = 0.2364

freqT = 0.2581

R(a) [AC] = 1.0000

R(b) [AG] = 3.3084

R(c) [AT] = 1.0000

R(d) [CG] = 1.0000

R(e) [CT] = 6.1867

R(f) [GT] = 1.0000

p-inv = 0.1990

gamma shape = 0.5350

Model = TPM1

partition = 012210

-lnL = 3075.5464

K = 122

R(a) [AC] = 1.0000

R(b) [AG] = 4.6234

R(c) [AT] = 1.0470

R(d) [CG] = 1.0470

R(e) [CT] = 4.6234

R(f) [GT] = 1.0000

Model = TPM1+I

partition = 012210

-lnL = 3003.3245

K = 123

R(a) [AC] = 1.0000

R(b) [AG] = 4.8623

R(c) [AT] = 1.0499

R(d) [CG] = 1.0499

R(e) [CT] = 4.8623

R(f) [GT] = 1.0000

p-inv = 0.5920

Model = TPM1+G

partition = 012210

-lnL = 2989.2389

K = 123

R(a) [AC] = 1.0000

R(b) [AG] = 4.8962

R(c) [AT] = 1.0535

R(d) [CG] = 1.0535

R(e) [CT] = 4.8962

R(f) [GT] = 1.0000

gamma shape = 0.3290

Model = TPM1+I+G

partition = 012210

-lnL = 2988.4352

K = 124

R(a) [AC] = 1.0000

R(b) [AG] = 4.7918

R(c) [AT] = 1.0460

R(d) [CG] = 1.0460

R(e) [CT] = 4.7918

R(f) [GT] = 1.0000

p-inv = 0.2090

gamma shape = 0.5140

Model = TPM1uf

partition = 012210

-lnL = 3066.1534

K = 125

freqA = 0.2447

freqC = 0.2544

freqG = 0.2196

freqT = 0.2813

R(a) [AC] = 1.0000

R(b) [AG] = 4.8563

R(c) [AT] = 1.1344

R(d) [CG] = 1.1344

R(e) [CT] = 4.8563

R(f) [GT] = 1.0000

Model = TPM1uf+I

partition = 012210

-lnL = 3000.8217

K = 126

freqA = 0.2469

freqC = 0.2527

freqG = 0.2243

freqT = 0.2761

R(a) [AC] = 1.0000

R(b) [AG] = 4.8057

R(c) [AT] = 1.0594

R(d) [CG] = 1.0594

R(e) [CT] = 4.8057

R(f) [GT] = 1.0000

p-inv = 0.5900

Model = TPM1uf+G

partition = 012210

-lnL = 2986.9305

K = 126

freqA = 0.2480

freqC = 0.2550

freqG = 0.2238

freqT = 0.2732

R(a) [AC] = 1.0000

R(b) [AG] = 4.8365

R(c) [AT] = 1.0638

R(d) [CG] = 1.0638

R(e) [CT] = 4.8365

R(f) [GT] = 1.0000

gamma shape = 0.3330

Model = TPM1uf+I+G

partition = 012210

-lnL = 2985.9339

K = 127

freqA = 0.2461

freqC = 0.2567

freqG = 0.2232

freqT = 0.2740

R(a) [AC] = 1.0000

R(b) [AG] = 4.7305

R(c) [AT] = 1.0560

R(d) [CG] = 1.0560

R(e) [CT] = 4.7305

R(f) [GT] = 1.0000

p-inv = 0.2080

gamma shape = 0.5200

Model = TPM2

partition = 010212

-lnL = 3071.3384

K = 122

R(a) [AC] = 1.7978

R(b) [AG] = 6.2770

R(c) [AT] = 1.7978

R(d) [CG] = 1.0000

R(e) [CT] = 6.2770

R(f) [GT] = 1.0000

Model = TPM2+I

partition = 010212

-lnL = 2997.9754

K = 123

R(a) [AC] = 2.0235

R(b) [AG] = 6.9915

R(c) [AT] = 2.0235

R(d) [CG] = 1.0000

R(e) [CT] = 6.9915

R(f) [GT] = 1.0000

p-inv = 0.5940

Model = TPM2+G

partition = 010212

-lnL = 2983.8497

K = 123

R(a) [AC] = 2.0488

R(b) [AG] = 7.0805

R(c) [AT] = 2.0488

R(d) [CG] = 1.0000

R(e) [CT] = 7.0805

R(f) [GT] = 1.0000

gamma shape = 0.3250

Model = TPM2+I+G

partition = 010212

-lnL = 2982.2050

K = 124

R(a) [AC] = 2.0268

R(b) [AG] = 7.0479

R(c) [AT] = 2.0268

R(d) [CG] = 1.0000

R(e) [CT] = 7.0479

R(f) [GT] = 1.0000

p-inv = 0.2220

gamma shape = 0.5230

Model = TPM2uf

partition = 010212

-lnL = 3062.8133

K = 125

freqA = 0.2378

freqC = 0.2547

freqG = 0.2261

freqT = 0.2814

R(a) [AC] = 1.7353

R(b) [AG] = 6.2041

R(c) [AT] = 1.7353

R(d) [CG] = 1.0000

R(e) [CT] = 6.2041

R(f) [GT] = 1.0000

Model = TPM2uf+I

partition = 010212

-lnL = 2995.7710

K = 126

freqA = 0.2334

freqC = 0.2534

freqG = 0.2359

freqT = 0.2772

R(a) [AC] = 2.0684

R(b) [AG] = 6.8642

R(c) [AT] = 2.0684

R(d) [CG] = 1.0000

R(e) [CT] = 6.8642

R(f) [GT] = 1.0000

p-inv = 0.5920

Model = TPM2uf+G

partition = 010212

-lnL = 2981.9063

K = 126

freqA = 0.2333

freqC = 0.2558

freqG = 0.2371

freqT = 0.2737

R(a) [AC] = 2.1045

R(b) [AG] = 7.0607

R(c) [AT] = 2.1045

R(d) [CG] = 1.0000

R(e) [CT] = 7.0607

R(f) [GT] = 1.0000

gamma shape = 0.3270

Model = TPM2uf+I+G

partition = 010212

-lnL = 2980.1215

K = 127

freqA = 0.2331

freqC = 0.2555

freqG = 0.2364

freqT = 0.2750

R(a) [AC] = 2.0809

R(b) [AG] = 7.0280

R(c) [AT] = 2.0809

R(d) [CG] = 1.0000

R(e) [CT] = 7.0280

R(f) [GT] = 1.0000

p-inv = 0.2150

gamma shape = 0.5200

Model = TPM3

partition = 012012

-lnL = 3075.2342

K = 122

R(a) [AC] = 0.8487

R(b) [AG] = 4.1661

R(c) [AT] = 1.0000

R(d) [CG] = 0.8487

R(e) [CT] = 4.1661

R(f) [GT] = 1.0000

Model = TPM3+I

partition = 012012

-lnL = 3002.9178

K = 123

R(a) [AC] = 0.8220

R(b) [AG] = 4.3037

R(c) [AT] = 1.0000

R(d) [CG] = 0.8220

R(e) [CT] = 4.3037

R(f) [GT] = 1.0000

p-inv = 0.5930

Model = TPM3+G

partition = 012012

-lnL = 2988.5907

K = 123

R(a) [AC] = 0.7786

R(b) [AG] = 4.2055

R(c) [AT] = 1.0000

R(d) [CG] = 0.7786

R(e) [CT] = 4.2055

R(f) [GT] = 1.0000

gamma shape = 0.3280

Model = TPM3+I+G

partition = 012012

-lnL = 2986.6360

K = 124

R(a) [AC] = 0.7534

R(b) [AG] = 4.1399

R(c) [AT] = 1.0000

R(d) [CG] = 0.7534

R(e) [CT] = 4.1399

R(f) [GT] = 1.0000

p-inv = 0.2190

gamma shape = 0.5230

Model = TPM3uf

partition = 012012

-lnL = 3066.0681

K = 125

freqA = 0.2447

freqC = 0.2565

freqG = 0.2198

freqT = 0.2790

R(a) [AC] = 0.8565

R(b) [AG] = 4.2235

R(c) [AT] = 1.0000

R(d) [CG] = 0.8565

R(e) [CT] = 4.2235

R(f) [GT] = 1.0000

Model = TPM3uf+I

partition = 012012

-lnL = 3000.6350

K = 126

freqA = 0.2470

freqC = 0.2550

freqG = 0.2244

freqT = 0.2736

R(a) [AC] = 0.8648

R(b) [AG] = 4.3477

R(c) [AT] = 1.0000

R(d) [CG] = 0.8648

R(e) [CT] = 4.3477

R(f) [GT] = 1.0000

p-inv = 0.5900

Model = TPM3uf+G

partition = 012012

-lnL = 2986.5251

K = 126

freqA = 0.2481

freqC = 0.2586

freqG = 0.2240

freqT = 0.2693

R(a) [AC] = 0.8090

R(b) [AG] = 4.2231

R(c) [AT] = 1.0000

R(d) [CG] = 0.8090

R(e) [CT] = 4.2231

R(f) [GT] = 1.0000

gamma shape = 0.3320

Model = TPM3uf+I+G

partition = 012012

-lnL = 2984.4766

K = 127

freqA = 0.2476

freqC = 0.2589

freqG = 0.2236

freqT = 0.2698

R(a) [AC] = 0.7831

R(b) [AG] = 4.1562

R(c) [AT] = 1.0000

R(d) [CG] = 0.7831

R(e) [CT] = 4.1562

R(f) [GT] = 1.0000

p-inv = 0.2180

gamma shape = 0.5310

Model = TIM1ef

partition = 012230

-lnL = 3063.3669

K = 123

R(a) [AC] = 1.0000

R(b) [AG] = 3.1107

R(c) [AT] = 1.0228

R(d) [CG] = 1.0228

R(e) [CT] = 5.8566

R(f) [GT] = 1.0000

Model = TIM1ef+I

partition = 012230

-lnL = 2996.9258

K = 124

R(a) [AC] = 1.0000

R(b) [AG] = 3.2406

R(c) [AT] = 1.0222

R(d) [CG] = 1.0222

R(e) [CT] = 5.9470

R(f) [GT] = 1.0000

p-inv = 0.5770

Model = TIM1ef+G

partition = 012230

-lnL = 2979.7881

K = 124

R(a) [AC] = 1.0000

R(b) [AG] = 3.2486

R(c) [AT] = 1.0458

R(d) [CG] = 1.0458

R(e) [CT] = 6.3040

R(f) [GT] = 1.0000

gamma shape = 0.3520

Model = TIM1ef+I+G

partition = 012230

-lnL = 2981.1262

K = 125

R(a) [AC] = 1.0000

R(b) [AG] = 3.3040

R(c) [AT] = 1.0463

R(d) [CG] = 1.0463

R(e) [CT] = 6.2728

R(f) [GT] = 1.0000

p-inv = 0.1870

gamma shape = 0.5170

Model = TIM1

partition = 012230

-lnL = 3058.3324

K = 126

freqA = 0.2641

freqC = 0.2383

freqG = 0.2356

freqT = 0.2620

R(a) [AC] = 1.0000

R(b) [AG] = 3.4266

R(c) [AT] = 1.1344

R(d) [CG] = 1.1344

R(e) [CT] = 6.2969

R(f) [GT] = 1.0000

Model = TIM1+I

partition = 012230

-lnL = 2996.0835

K = 127

freqA = 0.2613

freqC = 0.2424

freqG = 0.2364

freqT = 0.2600

R(a) [AC] = 1.0000

R(b) [AG] = 3.2520

R(c) [AT] = 1.0299

R(d) [CG] = 1.0299

R(e) [CT] = 5.9268

R(f) [GT] = 1.0000

p-inv = 0.5770

Model = TIM1+G

partition = 012230

-lnL = 2979.6701

K = 127

freqA = 0.2630

freqC = 0.2430

freqG = 0.2373

freqT = 0.2567

R(a) [AC] = 1.0000

R(b) [AG] = 3.3254

R(c) [AT] = 1.0555

R(d) [CG] = 1.0555

R(e) [CT] = 6.3632

R(f) [GT] = 1.0000

gamma shape = 0.3470

Model = TIM1+I+G

partition = 012230

-lnL = 2980.3571

K = 128

freqA = 0.2631

freqC = 0.2434

freqG = 0.2366

freqT = 0.2570

R(a) [AC] = 1.0000

R(b) [AG] = 3.3044

R(c) [AT] = 1.0560

R(d) [CG] = 1.0560

R(e) [CT] = 6.2757

R(f) [GT] = 1.0000

p-inv = 0.1900

gamma shape = 0.5250

Model = TIM2ef

partition = 010232

-lnL = 3060.6676

K = 123

R(a) [AC] = 1.7908

R(b) [AG] = 4.3738

R(c) [AT] = 1.7908

R(d) [CG] = 1.0000

R(e) [CT] = 8.1706

R(f) [GT] = 1.0000

Model = TIM2ef+I

partition = 010232

-lnL = 2991.8204

K = 124

R(a) [AC] = 2.0147

R(b) [AG] = 4.9606

R(c) [AT] = 2.0147

R(d) [CG] = 1.0000

R(e) [CT] = 8.6221

R(f) [GT] = 1.0000

p-inv = 0.5800

Model = TIM2ef+G

partition = 010232

-lnL = 2977.6013

K = 124

R(a) [AC] = 2.0522

R(b) [AG] = 4.9321

R(c) [AT] = 2.0522

R(d) [CG] = 1.0000

R(e) [CT] = 8.9679

R(f) [GT] = 1.0000

gamma shape = 0.3440

Model = TIM2ef+I+G

partition = 010232

-lnL = 2975.3564

K = 125

R(a) [AC] = 2.0742

R(b) [AG] = 4.8202

R(c) [AT] = 2.0742

R(d) [CG] = 1.0000

R(e) [CT] = 9.1920

R(f) [GT] = 1.0000

p-inv = 0.1940

gamma shape = 0.5180

Model = TIM2

partition = 010232

-lnL = 3055.1310

K = 126

freqA = 0.2561

freqC = 0.2388

freqG = 0.2428

freqT = 0.2623

R(a) [AC] = 1.7177

R(b) [AG] = 4.3657

R(c) [AT] = 1.7177

R(d) [CG] = 1.0000

R(e) [CT] = 7.9819

R(f) [GT] = 1.0000

Model = TIM2+I

partition = 010232

-lnL = 2991.3452

K = 127

freqA = 0.2492

freqC = 0.2412

freqG = 0.2469

freqT = 0.2627

R(a) [AC] = 2.0034

R(b) [AG] = 4.9853

R(c) [AT] = 2.0034

R(d) [CG] = 1.0000

R(e) [CT] = 8.4984

R(f) [GT] = 1.0000

p-inv = 0.5810

Model = TIM2+G

partition = 010232

-lnL = 2977.3869

K = 127

freqA = 0.2494

freqC = 0.2438

freqG = 0.2481

freqT = 0.2588

R(a) [AC] = 2.0453

R(b) [AG] = 4.9527

R(c) [AT] = 2.0453

R(d) [CG] = 1.0000

R(e) [CT] = 8.8585

R(f) [GT] = 1.0000

gamma shape = 0.3450

Model = TIM2+I+G

partition = 010232

-lnL = 2975.1922

K = 128

freqA = 0.2486

freqC = 0.2445

freqG = 0.2491

freqT = 0.2578

R(a) [AC] = 2.0789

R(b) [AG] = 4.8572

R(c) [AT] = 2.0789

R(d) [CG] = 1.0000

R(e) [CT] = 9.1126

R(f) [GT] = 1.0000

p-inv = 0.1950

gamma shape = 0.5200

Model = TIM3ef

partition = 012032

-lnL = 3062.9247

K = 123

R(a) [AC] = 0.8281

R(b) [AG] = 2.8048

R(c) [AT] = 1.0000

R(d) [CG] = 0.8281

R(e) [CT] = 5.2812

R(f) [GT] = 1.0000

Model = TIM3ef+I

partition = 012032

-lnL = 2996.3908

K = 124

R(a) [AC] = 0.8051

R(b) [AG] = 2.8822

R(c) [AT] = 1.0000

R(d) [CG] = 0.8051

R(e) [CT] = 5.2858

R(f) [GT] = 1.0000

p-inv = 0.5780

Model = TIM3ef+G

partition = 012032

-lnL = 2980.4769

K = 124

R(a) [AC] = 0.7611

R(b) [AG] = 2.9004

R(c) [AT] = 1.0000

R(d) [CG] = 0.7611

R(e) [CT] = 5.3801

R(f) [GT] = 1.0000

gamma shape = 0.3500

Model = TIM3ef+I+G

partition = 012032

-lnL = 2980.1693

K = 125

R(a) [AC] = 0.7407

R(b) [AG] = 2.7866

R(c) [AT] = 1.0000

R(d) [CG] = 0.7407

R(e) [CT] = 5.2841

R(f) [GT] = 1.0000

p-inv = 0.1910

gamma shape = 0.5200

Model = TIM3

partition = 012032

-lnL = 3058.1977

K = 126

freqA = 0.2642

freqC = 0.2403

freqG = 0.2358

freqT = 0.2597

R(a) [AC] = 0.8464

R(b) [AG] = 2.9598

R(c) [AT] = 1.0000

R(d) [CG] = 0.8464

R(e) [CT] = 5.4522

R(f) [GT] = 1.0000

Model = TIM3+I

partition = 012032

-lnL = 2995.9998

K = 127

freqA = 0.2626

freqC = 0.2424

freqG = 0.2358

freqT = 0.2592

R(a) [AC] = 0.8572

R(b) [AG] = 3.1085

R(c) [AT] = 1.0000

R(d) [CG] = 0.8572

R(e) [CT] = 5.4069

R(f) [GT] = 1.0000

p-inv = 0.5780

Model = TIM3+G

partition = 012032

-lnL = 2981.7701

K = 127

freqA = 0.2639

freqC = 0.2467

freqG = 0.2363

freqT = 0.2531

R(a) [AC] = 0.7926

R(b) [AG] = 3.0095

R(c) [AT] = 1.0000

R(d) [CG] = 0.7926

R(e) [CT] = 5.4108

R(f) [GT] = 1.0000

gamma shape = 0.3490

Model = TIM3+I+G

partition = 012032

-lnL = 2979.1939

K = 128

freqA = 0.2642

freqC = 0.2463

freqG = 0.2366

freqT = 0.2529

R(a) [AC] = 0.7723

R(b) [AG] = 2.9105

R(c) [AT] = 1.0000

R(d) [CG] = 0.7723

R(e) [CT] = 5.4339

R(f) [GT] = 1.0000

p-inv = 0.2050

gamma shape = 0.5420

Model = TVMef

partition = 012314

-lnL = 3070.9952

K = 124

R(a) [AC] = 1.5326

R(b) [AG] = 5.8583

R(c) [AT] = 1.8318

R(d) [CG] = 0.8701

R(e) [CT] = 5.8583

R(f) [GT] = 1.0000

Model = TVMef+I

partition = 012314

-lnL = 2997.5092

K = 125

R(a) [AC] = 1.6741

R(b) [AG] = 6.4662

R(c) [AT] = 2.0875

R(d) [CG] = 0.8549

R(e) [CT] = 6.4662

R(f) [GT] = 1.0000

p-inv = 0.5940

Model = TVMef+G

partition = 012314

-lnL = 2983.1337

K = 125

R(a) [AC] = 1.6055

R(b) [AG] = 6.3844

R(c) [AT] = 2.1293

R(d) [CG] = 0.8138

R(e) [CT] = 6.3844

R(f) [GT] = 1.0000

gamma shape = 0.3240

Model = TVMef+I+G

partition = 012314

-lnL = 2981.6594

K = 126

R(a) [AC] = 1.5385

R(b) [AG] = 6.3174

R(c) [AT] = 2.1315

R(d) [CG] = 0.7961

R(e) [CT] = 6.3174

R(f) [GT] = 1.0000

p-inv = 0.2250

gamma shape = 0.5200

Model = TVM

partition = 012314

-lnL = 3062.4265

K = 127

freqA = 0.2380

freqC = 0.2567

freqG = 0.2261

freqT = 0.2793

R(a) [AC] = 1.5143

R(b) [AG] = 6.0947

R(c) [AT] = 1.8971

R(d) [CG] = 0.9647

R(e) [CT] = 6.0947

R(f) [GT] = 1.0000

Model = TVM+I

partition = 012314

-lnL = 2995.5184

K = 128

freqA = 0.2336

freqC = 0.2556

freqG = 0.2360

freqT = 0.2748

R(a) [AC] = 1.7943

R(b) [AG] = 6.4971

R(c) [AT] = 2.1281

R(d) [CG] = 0.8924

R(e) [CT] = 6.4971

R(f) [GT] = 1.0000

p-inv = 0.5920

Model = TVM+G

partition = 012314

-lnL = 2981.4184

K = 128

freqA = 0.2334

freqC = 0.2594

freqG = 0.2373

freqT = 0.2698

R(a) [AC] = 1.7151

R(b) [AG] = 6.5131

R(c) [AT] = 2.1949

R(d) [CG] = 0.8466

R(e) [CT] = 6.5131

R(f) [GT] = 1.0000

gamma shape = 0.3260

Model = TVM+I+G

partition = 012314

-lnL = 2979.4793

K = 129

freqA = 0.2333

freqC = 0.2597

freqG = 0.2367

freqT = 0.2703

R(a) [AC] = 1.6411

R(b) [AG] = 6.4158

R(c) [AT] = 2.1881

R(d) [CG] = 0.8295

R(e) [CT] = 6.4158

R(f) [GT] = 1.0000

p-inv = 0.2190

gamma shape = 0.5210

Model = SYM

partition = 012345

-lnL = 3059.4516

K = 125

R(a) [AC] = 1.5118

R(b) [AG] = 3.8619

R(c) [AT] = 1.8075

R(d) [CG] = 0.8183

R(e) [CT] = 7.4538

R(f) [GT] = 1.0000

Model = SYM+I

partition = 012345

-lnL = 2990.3462

K = 126

R(a) [AC] = 1.6041

R(b) [AG] = 4.5260

R(c) [AT] = 2.0550

R(d) [CG] = 0.8448

R(e) [CT] = 8.0447

R(f) [GT] = 1.0000

p-inv = 0.5810

Model = SYM+G

partition = 012345

-lnL = 2972.8048

K = 126

R(a) [AC] = 1.5334

R(b) [AG] = 4.1209

R(c) [AT] = 2.0914

R(d) [CG] = 0.7541

R(e) [CT] = 8.0137

R(f) [GT] = 1.0000

gamma shape = 0.3460

Model = SYM+I+G

partition = 012345

-lnL = 2974.6703

K = 127

R(a) [AC] = 1.5465

R(b) [AG] = 4.3887

R(c) [AT] = 2.1252

R(d) [CG] = 0.7983

R(e) [CT] = 8.2128

R(f) [GT] = 1.0000

p-inv = 0.2160

gamma shape = 0.5420

Model = GTR

partition = 012345

-lnL = 3054.7024

K = 128

freqA = 0.2564

freqC = 0.2406

freqG = 0.2428

freqT = 0.2602

R(a) [AC] = 1.4805

R(b) [AG] = 4.2572

R(c) [AT] = 1.8743

R(d) [CG] = 0.9520

R(e) [CT] = 7.7939

R(f) [GT] = 1.0000

Model = GTR+I

partition = 012345

-lnL = 2987.7100

K = 129

freqA = 0.2482

freqC = 0.2440

freqG = 0.2484

freqT = 0.2595

R(a) [AC] = 1.6614

R(b) [AG] = 4.3989

R(c) [AT] = 2.0483

R(d) [CG] = 0.8276

R(e) [CT] = 7.9459

R(f) [GT] = 1.0000

p-inv = 0.5790

Model = GTR+G

partition = 012345

-lnL = 2972.7904

K = 129

freqA = 0.2488

freqC = 0.2490

freqG = 0.2496

freqT = 0.2526

R(a) [AC] = 1.5573

R(b) [AG] = 4.1766

R(c) [AT] = 2.1013

R(d) [CG] = 0.7641

R(e) [CT] = 8.0405

R(f) [GT] = 1.0000

gamma shape = 0.3460

Model = GTR+I+G

partition = 012345

-lnL = 2972.4569

K = 130

freqA = 0.2486

freqC = 0.2492

freqG = 0.2500

freqT = 0.2523

R(a) [AC] = 1.5584

R(b) [AG] = 4.1799

R(c) [AT] = 2.1184

R(d) [CG] = 0.7590

R(e) [CT] = 8.0562

R(f) [GT] = 1.0000

p-inv = 0.1980

gamma shape = 0.5220

Best-fit models should be reoptimized for comparison with unconstrained likelihood

Computation of likelihood scores completed. It took 00h:01:54:05.

---------------------------------------------------------------

* *

* AKAIKE INFORMATION CRITERION (AIC) *

* *

---------------------------------------------------------------

Model selected:

Model = SYM+G

partition = 012345

-lnL = 2972.8048

K = 126

R(a) [AC] = 1.5334

R(b) [AG] = 4.1209

R(c) [AT] = 2.0914

R(d) [CG] = 0.7541

R(e) [CT] = 8.0137

R(f) [GT] = 1.0000

gamma shape = 0.3460

--

PAUP* Commands Block:

If you want to load the selected model and associated estimates in PAUP*,

attach the next block of commands after the data in your PAUP file:

[!

Likelihood settings from best-fit model (SYM+G) selected by AIC

with jModeltest 2.1.10 v20160303 on Thu Dec 29 23:05:44 PST 2022]

BEGIN PAUP;

Lset base=equal nst=6 rmat=(1.5334 4.1209 2.0914 0.7541 8.0137) rates=gamma shape=0.3460 ncat=4 pinvar=0;

END;

--

Tree for the best AIC model = (lepidioides_18:0.01544422,spinosum_23:0.03159457,(((((MW791191_armenum:0.00180655,armenum_3:0.00536430):0.01267078,((MT799724_karamanicum:0.00174003,karamanicum_43:0.00538075):0.00355727,(schistosum_21:0.00350440,(demirizii_40:0.00524652,(subulatum_20:0.00175326,alanyae_39:0.00174289):0.00173898):0.00000008):0.00178055):0.00180448):0.00463002,(spicatum_35:0.00950546,(DQ518386_elongatum:0.01795077,glaucinum_41:0.00993762):0.00574526):0.00166900):0.00359495,((FM180113:0.01954692,((((Szowitsii_51:0.00000012,(elongatum_12:0.00174291,virgatum_26:0.00000010):0.00174457):0.01245227,(sintenisii_48:0.00531323,(MW791193_grandiflorum:0.00000013,stenopterum_31:0.00527945):0.00175032):0.00531550):0.00000009,(eunomioides_10:0.01428690,(huber-morathii_42:0.00000001,(capitatumi_4:0.00000001,MT799726_huber-morathii:0.00000009):0.00000001):0.00527492):0.00000009):0.00000011,(MT799723_grandiflorum:0.00928952,(umbellatum_32:0.01030578,(grandiflorum_14:0.00175559,(edentulum_53:0.00352265,membranaceum_33Copen:0.00351540):0.00000010):0.00213468):0.00366546):0.00550212):0.00245751):0.00974053,(coridifolium_7:0.00000001,(thomasianum_54:0.00000001,MT799725_cordifolium:0.00000010):0.00000001):0.00929935):0.00201655):0.01044620,((saxatile_34:0.00889173,((HM1454_Aethionema_S1865:0.00172735,(Shirkuh_J2:0.00350245,W_0184833_Aethionema_ITS:0.00172939):0.00347489):0.00000008,(S658_Sp_nova_HM478:0.00739050,Umbellatum_Archibold_J1:0.00215194):0.00000008):0.00169740):0.00175608,((Lycium_13:0.01248811,fimbriatum_44:0.01069286):0.00161348,((semnanense_50:0.01797502,(munzurense_46:0.01030141,stylosum_19:0.00979347):0.00297990):0.00171050,((((froedinii_11:0.00173227,(AY254539_arabicum:0.00348633,arabicum_2:0.00173269):0.00174396):0.00384222,(heterocarpum_15:0.01036516,EU938532_carneum_ITS1:0.00557516):0.00176182):0.00847206,(retsina_38:0.02475761,((turcicum_1:0.00000011,(MW791189_turcicum:0.00000001,acarii_37:0.00698982):0.00349528):0.00172296,(cordatum_6:0.00873089,(MT799718_dumanii:0.00364716,(MW791190_dumanii:0.00000001,dumanii_9:0.00000009):0.00348188):0.00354569):0.00173769):0.00525929):0.00000016):0.00181357,((MF543734_thomasianum:0.00193798,orbiculatum_33:0.01432966):0.00337070,(GQ284853_saxatile:0.01043301,(rhodopaeum_24:0.00000001,syriacum_27:0.00000001):0.00503052):0.00232487):0.00000010):0.00000011):0.00000008):0.00000011):0.02351291):0.07286205);

* AIC MODEL SELECTION : Selection uncertainty

Model -lnL K AIC delta weight cumWeight

-------------------------------------------------------------------------

SYM+G 2972.80480 126 6197.609600 0.000000 0.685560 0.685560

TIM2ef+I+G 2975.35643 125 6200.712860 3.103260 0.145272 0.830832

TIM2ef+G 2977.60125 124 6203.202500 5.592900 0.041837 0.872670

SYM+I+G 2974.67032 127 6203.340640 5.731040 0.039045 0.911715

GTR+G 2972.79037 129 6203.580740 5.971140 0.034628 0.946343

GTR+I+G 2972.45694 130 6204.913880 7.304280 0.017780 0.964123

TrNef+G 2979.81085 123 6205.621700 8.012100 0.012481 0.976604

TIM2+I+G 2975.19220 128 6206.384400 8.774800 0.008524 0.985128

TIM1ef+G 2979.78812 124 6207.576240 9.966640 0.004697 0.989824

TIM2+G 2977.38690 127 6208.773800 11.164200 0.002581 0.992405

TIM3ef+G 2980.47692 124 6208.953840 11.344240 0.002359 0.994764

TrNef+I+G 2980.96028 124 6209.920560 12.310960 0.001455 0.996219

TIM3ef+I+G 2980.16928 125 6210.338560 12.728960 0.001180 0.997399

TIM1ef+I+G 2981.12615 125 6212.252300 14.642700 0.000453 0.997852

TPM2+I+G 2982.20498 124 6212.409960 14.800360 0.000419 0.998271

TrN+G 2980.38711 126 6212.774220 15.164620 0.000349 0.998621

TIM1+G 2979.67010 127 6213.340200 15.730600 0.000263 0.998884

TPM2+G 2983.84973 123 6213.699460 16.089860 0.000220 0.999104

TrN+I+G 2980.06770 127 6214.135400 16.525800 0.000177 0.999280

TPM2uf+I+G 2980.12149 127 6214.242980 16.633380 0.000168 0.999448

TIM3+I+G 2979.19389 128 6214.387780 16.778180 0.000156 0.999604

TVMef+I+G 2981.65942 126 6215.318840 17.709240 9.78e-005 0.999702

TPM2uf+G 2981.90626 126 6215.812520 18.202920 7.64e-005 0.999778

TVMef+G 2983.13374 125 6216.267480 18.657880 6.09e-005 0.999839

TIM1+I+G 2980.35709 128 6216.714180 19.104580 4.87e-005 0.999888

TVM+I+G 2979.47930 129 6216.958600 19.349000 4.31e-005 0.999931

TIM3+G 2981.77005 127 6217.540100 19.930500 3.22e-005 0.999963

TVM+G 2981.41844 128 6218.836880 21.227280 1.69e-005 0.999980

TPM3+I+G 2986.63599 124 6221.271980 23.662380 4.99e-006 0.999985

K80+G 2989.26834 122 6222.536680 24.927080 2.65e-006 0.999987

K80+I+G 2988.45824 123 6222.916480 25.306880 2.19e-006 0.999990

TPM3uf+I+G 2984.47662 127 6222.953240 25.343640 2.15e-006 0.999992

TPM3+G 2988.59069 123 6223.181380 25.571780 1.92e-006 0.999994

HKY+I+G 2985.96738 126 6223.934760 26.325160 1.32e-006 0.999995

HKY+G 2986.97194 125 6223.943880 26.334280 1.31e-006 0.999996

TPM1+G 2989.23895 123 6224.477900 26.868300 1.00e-006 0.999997

TPM1+I+G 2988.43516 124 6224.870320 27.260720 8.25e-007 0.999998

TPM3uf+G 2986.52508 126 6225.050160 27.440560 7.54e-007 0.999999

TPM1uf+G 2986.93052 126 6225.861040 28.251440 5.03e-007 0.999999

TPM1uf+I+G 2985.93394 127 6225.867880 28.258280 5.01e-007 1.000000

TIM2ef+I 2991.82038 124 6231.640760 34.031160 2.79e-008 1.000000

SYM+I 2990.34617 126 6232.692340 35.082740 1.65e-008 1.000000

GTR+I 2987.71004 129 6233.420080 35.810480 1.15e-008 1.000000

TIM2+I 2991.34522 127 6236.690440 39.080840 2.24e-009 1.000000

TrNef+I 2996.10155 123 6238.203100 40.593500 1.05e-009 1.000000

TIM3ef+I 2996.39079 124 6240.781580 43.171980 2.89e-010 1.000000

TIM1ef+I 2996.92577 124 6241.851540 44.241940 1.69e-010 1.000000

TPM2+I 2997.97542 123 6241.950840 44.341240 1.61e-010 1.000000

TPM2uf+I 2995.77105 126 6243.542100 45.932500 7.28e-011 1.000000

TrN+I 2996.23495 126 6244.469900 46.860300 4.58e-011 1.000000

TVMef+I 2997.50920 125 6245.018400 47.408800 3.48e-011 1.000000

TIM3+I 2995.99983 127 6245.999660 48.390060 2.13e-011 1.000000

TIM1+I 2996.08350 127 6246.167000 48.557400 1.96e-011 1.000000

TVM+I 2995.51843 128 6247.036860 49.427260 1.27e-011 1.000000

K80+I 3003.04891 122 6250.097820 52.488220 2.74e-012 1.000000

HKY+I 3000.85853 125 6251.717060 54.107460 1.22e-012 1.000000

TPM3+I 3002.91776 123 6251.835520 54.225920 1.15e-012 1.000000

TPM1+I 3003.32454 123 6252.649080 55.039480 7.66e-013 1.000000

TPM3uf+I 3000.63496 126 6253.269920 55.660320 5.62e-013 1.000000

TPM1uf+I 3000.82171 126 6253.643420 56.033820 4.66e-013 1.000000

TIM2 3055.13104 126 6362.262080 164.652480 1.21e-036 1.000000

GTR 3054.70242 128 6365.404840 167.795240 2.51e-037 1.000000

TrN 3058.52666 125 6367.053320 169.443720 1.10e-037 1.000000

TIM2ef 3060.66761 123 6367.335220 169.725620 9.56e-038 1.000000

TIM3 3058.19774 126 6368.395480 170.785880 5.63e-038 1.000000

TIM1 3058.33235 126 6368.664700 171.055100 4.92e-038 1.000000

SYM 3059.45164 125 6368.903280 171.293680 4.37e-038 1.000000

TIM3ef 3062.92466 123 6371.849320 174.239720 1.00e-038 1.000000

TIM1ef 3063.36690 123 6372.733800 175.124200 6.43e-039 1.000000

TrNef 3064.87776 122 6373.755520 176.145920 3.86e-039 1.000000

TPM2uf 3062.81331 125 6375.626620 178.017020 1.51e-039 1.000000

TVM 3062.42654 127 6378.853080 181.243480 3.02e-040 1.000000

HKY 3066.35066 124 6380.701320 183.091720 1.20e-040 1.000000

TPM3uf 3066.06808 125 6382.136160 184.526560 5.84e-041 1.000000

TPM1uf 3066.15339 125 6382.306780 184.697180 5.36e-041 1.000000

TPM2 3071.33843 122 6386.676860 189.067260 6.03e-042 1.000000

TVMef 3070.99524 124 6389.990480 192.380880 1.15e-042 1.000000

K80 3075.57205 121 6393.144100 195.534500 2.38e-043 1.000000

TPM3 3075.23417 122 6394.468340 196.858740 1.23e-043 1.000000

TPM1 3075.54644 122 6395.092880 197.483280 8.98e-044 1.000000

F81+I+G 3073.21446 125 6396.428920 198.819320 4.60e-044 1.000000

JC+G 3078.63020 121 6399.260400 201.650800 1.12e-044 1.000000

JC+I+G 3078.51345 122 6401.026900 203.417300 4.62e-045 1.000000

F81+G 3076.81016 124 6401.620320 204.010720 3.43e-045 1.000000

JC+I 3091.98176 121 6425.963520 228.353920 1.78e-050 1.000000

F81+I 3090.41888 124 6428.837760 231.228160 4.22e-051 1.000000

F81 3157.11316 123 6560.226320 362.616720 1.24e-079 1.000000

JC 3161.44041 120 6562.880820 365.271220 3.30e-080 1.000000

-------------------------------------------------------------------------

-lnL: negative log likelihod

K: number of estimated parameters

AIC: Akaike Information Criterion

delta: AIC difference

weight: AIC weight

cumWeight: cumulative AIC weight

* AIC MODEL SELECTION : Confidence interval

There are 88 models in the 100% confidence interval: [ SYM+G TIM2ef+I+G TIM2ef+G SYM+I+G GTR+G GTR+I+G TrNef+G TIM2+I+G TIM1ef+G TIM2+G TIM3ef+G TrNef+I+G TIM3ef+I+G TIM1ef+I+G TPM2+I+G TrN+G TIM1+G TPM2+G TrN+I+G TPM2uf+I+G TIM3+I+G TVMef+I+G TPM2uf+G TVMef+G TIM1+I+G TVM+I+G TIM3+G TVM+G TPM3+I+G K80+G K80+I+G TPM3uf+I+G TPM3+G HKY+I+G HKY+G TPM1+G TPM1+I+G TPM3uf+G TPM1uf+G TPM1uf+I+G TIM2ef+I SYM+I GTR+I TIM2+I TrNef+I TIM3ef+I TIM1ef+I TPM2+I TPM2uf+I TrN+I TVMef+I TIM3+I TIM1+I TVM+I K80+I HKY+I TPM3+I TPM1+I TPM3uf+I TPM1uf+I TIM2 GTR TrN TIM2ef TIM3 TIM1 SYM TIM3ef TIM1ef TrNef TPM2uf TVM HKY TPM3uf TPM1uf TPM2 TVMef K80 TPM3 TPM1 F81+I+G JC+G JC+I+G F81+G JC+I F81+I F81 JC ]

* AIC MODEL SELECTION : Parameter importance

Parameter Importance

----------------------

fA 0.0648

fC 0.0648

fG 0.0648

fT 0.0648

kappa 0.0000

titv 0.0000

rAC 0.9801

rAG 1.0000

rAT 0.9818

rCG 0.7864

rCT 1.0000

rGT 1.0000

pinv(I) 0.0000

alpha(G) 0.7852

pinv(IG) 0.2148

alpha(IG) 0.2148

----------------------

Values have been rounded.

(I): considers only +I models.

(G): considers only +G models.

(IG): considers only +I+G models.

* AIC MODEL SELECTION : Best Model's command line

phyml -i /tmp/jmodeltest13117230865493229200.phy -d nt -n 1 -b 0 --run_id SYM+G -m 012345 -f 0.25,0.25,0.25,0.25 -c 4 -a e --no_memory_check -o tlr -s BEST

---------------------------------------------------------------

* *

* MODEL AVERAGED PHYLOGENY *

* *

---------------------------------------------------------------

Selection criterion: . . . . AIC

Confidence interval: . . . . 1.00

Consensus type:. . . . . . . 50% majority rule

Using 88 models in the 1.00 confidence interval = SYM+G TIM2ef+I+G TIM2ef+G SYM+I+G GTR+G GTR+I+G TrNef+G TIM2+I+G TIM1ef+G TIM2+G TIM3ef+G TrNef+I+G TIM3ef+I+G TIM1ef+I+G TPM2+I+G TrN+G TIM1+G TPM2+G TrN+I+G TPM2uf+I+G TIM3+I+G TVMef+I+G TPM2uf+G TVMef+G TIM1+I+G TVM+I+G TIM3+G TVM+G TPM3+I+G K80+G K80+I+G TPM3uf+I+G TPM3+G HKY+I+G HKY+G TPM1+G TPM1+I+G TPM3uf+G TPM1uf+G TPM1uf+I+G TIM2ef+I SYM+I GTR+I TIM2+I TrNef+I TIM3ef+I TIM1ef+I TPM2+I TPM2uf+I TrN+I TVMef+I TIM3+I TIM1+I TVM+I K80+I HKY+I TPM3+I TPM1+I TPM3uf+I TPM1uf+I TIM2 GTR TrN TIM2ef TIM3 TIM1 SYM TIM3ef TIM1ef TrNef TPM2uf TVM HKY TPM3uf TPM1uf TPM2 TVMef K80 TPM3 TPM1 F81+I+G JC+G JC+I+G F81+G JC+I F81+I F81 JC

Species in order:

1. lepidioides_18

2. spinosum_23

3. MW791191_armenum

4. armenum_3

5. MT799724_karamanicum

6. karamanicum_43

7. schistosum_21

8. demirizii_40

9. subulatum_20

10. alanyae_39

11. spicatum_35

12. DQ518386_elongatum

13. glaucinum_41

14. FM180113

15. Szowitsii_51

16. elongatum_12

17. virgatum_26

18. sintenisii_48

19. MW791193_grandiflorum

20. stenopterum_31

21. eunomioides_10

22. huber-morathii_42

23. capitatumi_4

24. MT799726_huber-morathii

25. MT799723_grandiflorum

26. umbellatum_32

27. grandiflorum_14

28. edentulum_53

29. membranaceum_33Copen

30. coridifolium_7

31. thomasianum_54

32. MT799725_cordifolium

33. saxatile_34

34. HM1454_Aethionema_S1865

35. Shirkuh_J2

36. W_0184833_Aethionema_ITS

37. S658_Sp_nova_HM478

38. Umbellatum_Archibold_J1

39. Lycium_13

40. fimbriatum_44

41. semnanense_50

42. munzurense_46

43. stylosum_19

44. froedinii_11

45. AY254539_arabicum

46. arabicum_2

47. heterocarpum_15

48. EU938532_carneum_ITS1

49. retsina_38

50. turcicum_1

51. MW791189_turcicum

52. acarii_37

53. cordatum_6

54. MT799718_dumanii

55. MW791190_dumanii

56. dumanii_9

57. MF543734_thomasianum

58. orbiculatum_33

59. GQ284853_saxatile

60. rhodopaeum_24

61. syriacum_27

Bipartitions included in the consensus tree

1234567891111111111222222222233333333334444444444555555555566

0123456789012345678901234567890123456789012345678901

************************************************************* ( 1.0 )

--*********************************************************** ( 1.0 )

--******************************----------------------------- ( 0.99974 )

--***********------------------------------------------------ ( 0.95491 )

--********--------------------------------------------------- ( 1.0 )

--**--------------------------------------------------------- ( 1.0 )

----******--------------------------------------------------- ( 1.0 )

----**------------------------------------------------------- ( 1.0 )

------****--------------------------------------------------- ( 1.0 )

-------***--------------------------------------------------- ( 0.78517 )

--------**--------------------------------------------------- ( 1.0 )

----------***------------------------------------------------ ( 0.95491 )

-----------**------------------------------------------------ ( 1.0 )

-------------*******************----------------------------- ( 1.0 )

-------------****************-------------------------------- ( 1.0 )

--------------***************-------------------------------- ( 1.0 )

--------------**********------------------------------------- ( 1.0 )

--------------******----------------------------------------- ( 0.73525 )

--------------***-------------------------------------------- ( 1.0 )

---------------**-------------------------------------------- ( 1.0 )

-----------------***----------------------------------------- ( 1.0 )

------------------**----------------------------------------- ( 1.0 )

--------------------****------------------------------------- ( 0.72277 )

---------------------***------------------------------------- ( 1.0 )

----------------------**------------------------------------- ( 1.0 )

------------------------*****-------------------------------- ( 1.0 )

-------------------------****-------------------------------- ( 1.0 )

--------------------------***-------------------------------- ( 1.0 )

---------------------------**-------------------------------- ( 1.0 )

-----------------------------***----------------------------- ( 1.0 )

------------------------------**----------------------------- ( 0.81265 )

--------------------------------***************************** ( 1.0 )

--------------------------------******----------------------- ( 1.0 )

---------------------------------*****----------------------- ( 1.0 )

---------------------------------***------------------------- ( 0.78517 )

----------------------------------**------------------------- ( 1.0 )

------------------------------------**----------------------- ( 0.78517 )

--------------------------------------*********************** ( 0.68557 )

--------------------------------------**--------------------- ( 1.0 )

----------------------------------------********************* ( 0.72019 )

----------------------------------------***------------------ ( 0.72019 )

-----------------------------------------**------------------ ( 0.99959 )

-------------------------------------------****************** ( 0.68557 )

-------------------------------------------*************----- ( 0.72019 )

-------------------------------------------*****------------- ( 1.0 )

-------------------------------------------***--------------- ( 1.0 )

--------------------------------------------**--------------- ( 1.0 )

----------------------------------------------**------------- ( 1.0 )

------------------------------------------------********----- ( 0.72019 )

-------------------------------------------------*******----- ( 1.0 )

-------------------------------------------------***--------- ( 1.0 )

--------------------------------------------------**--------- ( 1.0 )

----------------------------------------------------****----- ( 1.0 )

-----------------------------------------------------***----- ( 1.0 )

------------------------------------------------------**----- ( 1.0 )

--------------------------------------------------------***** ( 0.68792 )

--------------------------------------------------------**--- ( 1.0 )

----------------------------------------------------------*** ( 1.0 )

-----------------------------------------------------------** ( 1.0 )

+--61 glaucinum_41

+-115

| +----60 DQ518386_elongatum

+-116

| +--59 spicatum_35

+-117

| | +--58 karamanicum_43

| | +-112

| | | +--57 MT799724_karamanicum

| | +-113

| | | | +--56 alanyae_39

| | | | +-109

| | | | | +--55 subulatum_20

| | | | +-110

| | | | | +--54 demirizii_40

| | | +-111

| | | +--53 schistosum_21

| +-114

| | +--52 armenum_3

| +-108

| +--51 MW791191_armenum

+-118

| | +--50 MT799726_huber-morathii

| | +-101

| | | +--49 capitatumi_4

| | +-102

| | | +--48 huber-morathii_42

| | +-103

| | | +--47 eunomioides_10

| | +-104

| | | | +--46 virgatum_26

| | | | +--98

| | | | | +--45 elongatum_12

| | | | +--99

| | | | | +--44 Szowitsii_51

| | | +-100

| | | | +--43 stenopterum_31

| | | | +--96

| | | | | +--42 MW791193_grandiflorum

| | | +--97

| | | +--41 sintenisii_48

| | +-105

| | | | +--40 membranaceum_33Copen

| | | | +--92

| | | | | +--39 edentulum_53

| | | | +--93

| | | | | +--38 grandiflorum_14

| | | | +--94

| | | | | +--37 umbellatum_32

| | | +--95

| | | +--36 MT799723_grandiflorum

| | +-106

| | | +----35 FM180113

| +-107

| | +--34 MT799725_cordifolium

| | +--90

| | | +--33 thomasianum_54

| +--91

| +--32 coridifolium_7

+----------------------119

| | +--31 orbiculatum_33

| | +--84

| | | +--30 MF543734_thomasianum

| | +--85

| | | | +--29 syriacum_27

| | | | +--82

| | | | | +--28 rhodopaeum_24

| | | +--83

| | | +--27 GQ284853_saxatile

| | +--86

| | | | +--26 dumanii_9

| | | | +--76

| | | | | +--25 MW791190_dumanii

| | | | +--77

| | | | | +--24 MT799718_dumanii

| | | | +--78

| | | | | +--23 cordatum_6

| | | | +--79

| | | | | | +--22 acarii_37

| | | | | | +--74

| | | | | | | +--21 MW791189_turcicum

| | | | | +--75

| | | | | +--20 turcicum_1

| | | | +--80

| | | | | +------19 retsina_38

| | | +--81

| | | | +--18 EU938532_carneum_ITS1

| | | | +--72

| | | | | +--17 heterocarpum_15

| | | +--73

| | | | +--16 arabicum_2

| | | | +--70

| | | | | +--15 AY254539_arabicum

| | | +--71

| | | +--14 froedinii_11

| | +--87

| | | | +--13 stylosum_19

| | | | +--68

| | | | | +--12 munzurense_46

| | | +--69

| | | +----11 semnanense_50

| | +--88

| | | | +--10 fimbriatum_44

| | | +--67

| | | +---9 Lycium_13

| +------89

| | +---8 W_0184833_Aethionema_ITS

| | +--63

| | | +---7 Shirkuh_J2

| | +--64

| | | +---6 HM1454_Aethionema_S1865

| | +--65

| | | | +---5 Umbellatum_Archibold_J1

| | | +--62

| | | +---4 S658_Sp_nova_HM478

| +--66

| +---3 saxatile_34

|

+---------2 spinosum_23

|

+----1 lepidioides_18

(lepidioides_18:0.0154442200,spinosum_23:0.0315945700,(((saxatile_34:0.0088917300,((S658_Sp_nova_HM478:0.0073905000,Umbellatum_Archibold_J1:0.0021519400):0.7852:0.0000000800,(HM1454_Aethionema_S1865:0.0017273500,(Shirkuh_J2:0.0035024500,W_0184833_Aethionema_ITS:0.0017293900):1.0000:0.0034748900):0.7852:0.0000000800):1.0000:0.0016974000):1.0000:0.0017560800,((Lycium_13:0.0124881100,fimbriatum_44:0.0106928600):1.0000:0.0016134800,((semnanense_50:0.0179750200,(munzurense_46:0.0103014100,stylosum_19:0.0097934700):0.9996:0.0029799000):0.7202:0.0017105000,((((froedinii_11:0.0017322700,(AY254539_arabicum:0.0034863300,arabicum_2:0.0017326900):1.0000:0.0017439600):1.0000:0.0038422200,(heterocarpum_15:0.0103651600,EU938532_carneum_ITS1:0.0055751600):1.0000:0.0017618200):1.0000:0.0084720600,(retsina_38:0.0247576100,((turcicum_1:0.0000001100,(MW791189_turcicum:0.0000000100,acarii_37:0.0069898200):1.0000:0.0034952800):1.0000:0.0017229600,(cordatum_6:0.0087308900,(MT799718_dumanii:0.0036471600,(MW791190_dumanii:0.0000000100,dumanii_9:0.0000000900):1.0000:0.0034818800):1.0000:0.0035456900):1.0000:0.0017376900):1.0000:0.0052592900):0.7202:0.0000001600):0.7202:0.0018135700,((GQ284853_saxatile:0.0104330100,(rhodopaeum_24:0.0000000100,syriacum_27:0.0000000100):1.0000:0.0050305200):1.0000:0.0023248700,(MF543734_thomasianum:0.0019379800,orbiculatum_33:0.0143296600):1.0000:0.0033707000):0.6879:0.0000001000):0.6856:0.0000001100):0.7202:0.0000000800):0.6856:0.0000001100):1.0000:0.0235129100,(((coridifolium_7:0.0000000100,(thomasianum_54:0.0000000100,MT799725_cordifolium:0.0000001000):0.8126:0.0000000100):1.0000:0.0092993500,(FM180113:0.0195469200,((MT799723_grandiflorum:0.0092895200,(umbellatum_32:0.0103057800,(grandiflorum_14:0.0017555900,(edentulum_53:0.0035226500,membranaceum_33Copen:0.0035154000):1.0000:0.0000001000):1.0000:0.0021346800):1.0000:0.0036654600):1.0000:0.0055021200,(((sintenisii_48:0.0053132300,(MW791193_grandiflorum:0.0000001300,stenopterum_31:0.0052794500):1.0000:0.0017503200):1.0000:0.0053155000,(Szowitsii_51:0.0000001200,(elongatum_12:0.0017429100,virgatum_26:0.0000001000):1.0000:0.0017445700):1.0000:0.0124522700):0.7352:0.0000000900,(eunomioides_10:0.0142869000,(huber-morathii_42:0.0000000100,(capitatumi_4:0.0000000100,MT799726_huber-morathii:0.0000000900):1.0000:0.0000000100):1.0000:0.0052749200):0.7228:0.0000000900):1.0000:0.0000001100):1.0000:0.0024575100):1.0000:0.0097405300):1.0000:0.0020165500,(((MW791191_armenum:0.0018065500,armenum_3:0.0053643000):1.0000:0.0126707800,((schistosum_21:0.0035044000,(demirizii_40:0.0052465200,(subulatum_20:0.0017532600,alanyae_39:0.0017428900):1.0000:0.0017389800):0.7852:0.0000000800):1.0000:0.0017805500,(MT799724_karamanicum:0.0017400300,karamanicum_43:0.0053807500):1.0000:0.0035572700):1.0000:0.0018044800):1.0000:0.0046300200,(spicatum_35:0.0095054600,(DQ518386_elongatum:0.0179507700,glaucinum_41:0.0099376200):1.0000:0.0057452600):0.9549:0.0016690000):0.9549:0.0035949500):0.9997:0.0104462000):1.0000:0.0728620500);

Note: this tree is unrooted. Branch lengths are the expected number of substitutions per site. Labels next to parentheses represent phylogenetic uncertainty due to model selection (see documentation)

---------------------------------------------------------------

* *

* CORRECTED AKAIKE INFORMATION CRITERION (AICc) *

* *

---------------------------------------------------------------

Sample size: 636.0

Model selected:

Model = SYM+G

partition = 012345

-lnL = 2972.8048

K = 126

R(a) [AC] = 1.5334

R(b) [AG] = 4.1209

R(c) [AT] = 2.0914

R(d) [CG] = 0.7541

R(e) [CT] = 8.0137

R(f) [GT] = 1.0000

gamma shape = 0.3460

--

PAUP* Commands Block:

If you want to load the selected model and associated estimates in PAUP*,

attach the next block of commands after the data in your PAUP file:

[!

Likelihood settings from best-fit model (SYM+G) selected by AICc

with jModeltest 2.1.10 v20160303 on Thu Dec 29 23:05:45 PST 2022]

BEGIN PAUP;

Lset base=equal nst=6 rmat=(1.5334 4.1209 2.0914 0.7541 8.0137) rates=gamma shape=0.3460 ncat=4 pinvar=0;

END;

--

Tree for the best AICc model = (lepidioides_18:0.01544422,spinosum_23:0.03159457,(((((MW791191_armenum:0.00180655,armenum_3:0.00536430):0.01267078,((MT799724_karamanicum:0.00174003,karamanicum_43:0.00538075):0.00355727,(schistosum_21:0.00350440,(demirizii_40:0.00524652,(subulatum_20:0.00175326,alanyae_39:0.00174289):0.00173898):0.00000008):0.00178055):0.00180448):0.00463002,(spicatum_35:0.00950546,(DQ518386_elongatum:0.01795077,glaucinum_41:0.00993762):0.00574526):0.00166900):0.00359495,((FM180113:0.01954692,((((Szowitsii_51:0.00000012,(elongatum_12:0.00174291,virgatum_26:0.00000010):0.00174457):0.01245227,(sintenisii_48:0.00531323,(MW791193_grandiflorum:0.00000013,stenopterum_31:0.00527945):0.00175032):0.00531550):0.00000009,(eunomioides_10:0.01428690,(huber-morathii_42:0.00000001,(capitatumi_4:0.00000001,MT799726_huber-morathii:0.00000009):0.00000001):0.00527492):0.00000009):0.00000011,(MT799723_grandiflorum:0.00928952,(umbellatum_32:0.01030578,(grandiflorum_14:0.00175559,(edentulum_53:0.00352265,membranaceum_33Copen:0.00351540):0.00000010):0.00213468):0.00366546):0.00550212):0.00245751):0.00974053,(coridifolium_7:0.00000001,(thomasianum_54:0.00000001,MT799725_cordifolium:0.00000010):0.00000001):0.00929935):0.00201655):0.01044620,((saxatile_34:0.00889173,((HM1454_Aethionema_S1865:0.00172735,(Shirkuh_J2:0.00350245,W_0184833_Aethionema_ITS:0.00172939):0.00347489):0.00000008,(S658_Sp_nova_HM478:0.00739050,Umbellatum_Archibold_J1:0.00215194):0.00000008):0.00169740):0.00175608,((Lycium_13:0.01248811,fimbriatum_44:0.01069286):0.00161348,((semnanense_50:0.01797502,(munzurense_46:0.01030141,stylosum_19:0.00979347):0.00297990):0.00171050,((((froedinii_11:0.00173227,(AY254539_arabicum:0.00348633,arabicum_2:0.00173269):0.00174396):0.00384222,(heterocarpum_15:0.01036516,EU938532_carneum_ITS1:0.00557516):0.00176182):0.00847206,(retsina_38:0.02475761,((turcicum_1:0.00000011,(MW791189_turcicum:0.00000001,acarii_37:0.00698982):0.00349528):0.00172296,(cordatum_6:0.00873089,(MT799718_dumanii:0.00364716,(MW791190_dumanii:0.00000001,dumanii_9:0.00000009):0.00348188):0.00354569):0.00173769):0.00525929):0.00000016):0.00181357,((MF543734_thomasianum:0.00193798,orbiculatum_33:0.01432966):0.00337070,(GQ284853_saxatile:0.01043301,(rhodopaeum_24:0.00000001,syriacum_27:0.00000001):0.00503052):0.00232487):0.00000010):0.00000011):0.00000008):0.00000011):0.02351291):0.07286205);

* AICc MODEL SELECTION : Selection uncertainty

Model -lnL K AICc delta weight cumWeight

-------------------------------------------------------------------------

SYM+G 2972.80480 126 6260.485828 0.000000 0.572823 0.572823

TIM2ef+I+G 2975.35643 125 6262.477566 1.991738 0.211602 0.784425

TIM2ef+G 2977.60125 124 6263.867862 3.382034 0.105590 0.890014

TrNef+G 2979.81085 123 6265.199825 4.713997 0.054249 0.944263

SYM+I+G 2974.67032 127 6267.340640 6.854812 0.018600 0.962863

TIM1ef+G 2979.78812 124 6268.241602 7.755774 0.011854 0.974718

TIM3ef+G 2980.47692 124 6269.619202 9.133374 0.005953 0.980670

GTR+G 2972.79037 129 6269.865325 9.379497 0.005264 0.985934

TrNef+I+G 2980.96028 124 6270.585922 10.100094 0.003671 0.989605

TIM2+I+G 2975.19220 128 6271.520495 11.034667 0.002301 0.991906

TIM3ef+I+G 2980.16928 125 6272.103266 11.617438 0.001719 0.993625

GTR+I+G 2972.45694 130 6272.359425 11.873597 0.001513 0.995138

TIM2+G 2977.38690 127 6272.773800 12.287972 0.001229 0.996367

TPM2+I+G 2982.20498 124 6273.075322 12.589494 0.001057 0.997425

TPM2+G 2983.84973 123 6273.277585 12.791757 0.000956 0.998380

TIM1ef+I+G 2981.12615 125 6274.017006 13.531178 0.000660 0.999041

TrN+G 2980.38711 126 6275.650448 15.164620 0.000292 0.999333

TIM1+G 2979.67010 127 6277.340200 16.854372 0.000125 0.999458

TVMef+G 2983.13374 125 6278.032186 17.546358 8.87e-005 0.999547

TrN+I+G 2980.06770 127 6278.135400 17.649572 8.42e-005 0.999631

TVMef+I+G 2981.65942 126 6278.195068 17.709240 8.18e-005 0.999713

TPM2uf+I+G 2980.12149 127 6278.242980 17.757152 7.98e-005 0.999792

TPM2uf+G 2981.90626 126 6278.688748 18.202920 6.39e-005 0.999856

TIM3+I+G 2979.19389 128 6279.523875 19.038047 4.21e-005 0.999898

K80+G 2989.26834 122 6281.039604 20.553776 1.97e-005 0.999918

TIM3+G 2981.77005 127 6281.540100 21.054272 1.54e-005 0.999933

TIM1+I+G 2980.35709 128 6281.850275 21.364447 1.31e-005 0.999947

TPM3+I+G 2986.63599 124 6281.937342 21.451514 1.26e-005 0.999959

K80+I+G 2988.45824 123 6282.494605 22.008777 9.53e-006 0.999969

TPM3+G 2988.59069 123 6282.759505 22.273677 8.34e-006 0.999977

TVM+I+G 2979.47930 129 6283.243185 22.757357 6.55e-006 0.999984

TVM+G 2981.41844 128 6283.972975 23.487147 4.55e-006 0.999988

TPM1+G 2989.23895 123 6284.056025 23.570197 4.36e-006 0.999993

TPM1+I+G 2988.43516 124 6285.535682 25.049854 2.08e-006 0.999995

HKY+G 2986.97194 125 6285.708586 25.222758 1.91e-006 0.999996

HKY+I+G 2985.96738 126 6286.810988 26.325160 1.10e-006 0.999998

TPM3uf+I+G 2984.47662 127 6286.953240 26.467412 1.02e-006 0.999999

TPM3uf+G 2986.52508 126 6287.926388 27.440560 6.30e-007 0.999999

TPM1uf+G 2986.93052 126 6288.737268 28.251440 4.20e-007 1.000000

TPM1uf+I+G 2985.93394 127 6289.867880 29.382052 2.39e-007 1.000000

TIM2ef+I 2991.82038 124 6292.306122 31.820294 7.05e-008 1.000000

SYM+I 2990.34617 126 6295.568568 35.082740 1.38e-008 1.000000

TrNef+I 2996.10155 123 6297.781225 37.295397 4.56e-009 1.000000

GTR+I 2987.71004 129 6299.704665 39.218837 1.74e-009 1.000000

TIM2+I 2991.34522 127 6300.690440 40.204612 1.07e-009 1.000000

TIM3ef+I 2996.39079 124 6301.446942 40.961114 7.30e-010 1.000000

TPM2+I 2997.97542 123 6301.528965 41.043137 7.01e-010 1.000000

TIM1ef+I 2996.92577 124 6302.516902 42.031074 4.28e-010 1.000000

TPM2uf+I 2995.77105 126 6306.418328 45.932500 6.08e-011 1.000000

TVMef+I 2997.50920 125 6306.783106 46.297278 5.07e-011 1.000000

TrN+I 2996.23495 126 6307.346128 46.860300 3.82e-011 1.000000

K80+I 3003.04891 122 6308.600744 48.114916 2.04e-011 1.000000

TIM3+I 2995.99983 127 6309.999660 49.513832 1.01e-011 1.000000

TIM1+I 2996.08350 127 6310.167000 49.681172 9.33e-012 1.000000

TPM3+I 3002.91776 123 6311.413645 50.927817 5.00e-012 1.000000

TVM+I 2995.51843 128 6312.172955 51.687127 3.42e-012 1.000000

TPM1+I 3003.32454 123 6312.227205 51.741377 3.33e-012 1.000000

HKY+I 3000.85853 125 6313.481766 52.995938 1.78e-012 1.000000

TPM3uf+I 3000.63496 126 6316.146148 55.660320 4.69e-013 1.000000

TPM1uf+I 3000.82171 126 6316.519648 56.033820 3.89e-013 1.000000

TIM2 3055.13104 126 6425.138308 164.652480 1.01e-036 1.000000

TIM2ef 3060.66761 123 6426.913345 166.427517 4.16e-037 1.000000

TrN 3058.52666 125 6428.818026 168.332198 1.60e-037 1.000000

GTR 3054.70242 128 6430.540935 170.055107 6.78e-038 1.000000

SYM 3059.45164 125 6430.667986 170.182158 6.36e-038 1.000000

TIM3 3058.19774 126 6431.271708 170.785880 4.70e-038 1.000000

TIM3ef 3062.92466 123 6431.427445 170.941617 4.35e-038 1.000000

TIM1 3058.33235 126 6431.540928 171.055100 4.11e-038 1.000000

TrNef 3064.87776 122 6432.258444 171.772616 2.87e-038 1.000000

TIM1ef 3063.36690 123 6432.311925 171.826097 2.80e-038 1.000000

TPM2uf 3062.81331 125 6437.391326 176.905498 2.21e-039 1.000000

HKY 3066.35066 124 6441.366682 180.880854 3.02e-040 1.000000

TVM 3062.42654 127 6442.853080 182.367252 1.44e-040 1.000000

TPM3uf 3066.06808 125 6443.900866 183.415038 8.51e-041 1.000000

TPM1uf 3066.15339 125 6444.071486 183.585658 7.81e-041 1.000000

TPM2 3071.33843 122 6445.179784 184.693956 4.49e-041 1.000000

K80 3075.57205 121 6450.583789 190.097961 3.01e-042 1.000000

TVMef 3070.99524 124 6450.655842 190.170014 2.90e-042 1.000000

TPM3 3075.23417 122 6452.971264 192.485436 9.13e-043 1.000000

TPM1 3075.54644 122 6453.595804 193.109976 6.68e-043 1.000000

JC+G 3078.63020 121 6456.700089 196.214261 1.41e-043 1.000000

F81+I+G 3073.21446 125 6458.193626 197.707798 6.70e-044 1.000000

JC+I+G 3078.51345 122 6459.529824 199.043996 3.44e-044 1.000000

F81+G 3076.81016 124 6462.285682 201.799854 8.66e-045 1.000000

JC+I 3091.98176 121 6483.403209 222.917381 2.25e-049 1.000000

F81+I 3090.41888 124 6489.503122 229.017294 1.07e-050 1.000000

JC 3161.44041 120 6619.269170 358.783342 7.07e-079 1.000000

F81 3157.11316 123 6619.804445 359.318617 5.41e-079 1.000000

-------------------------------------------------------------------------

-lnL: negative log likelihod

K: number of estimated parameters

AICc: Corrected Akaike Information Criterion

delta: AICc difference

weight: AICc weight

cumWeight: cumulative AICc weight

* AICc MODEL SELECTION : Confidence interval

There are 88 models in the 100% confidence interval: [ SYM+G TIM2ef+I+G TIM2ef+G TrNef+G SYM+I+G TIM1ef+G TIM3ef+G GTR+G TrNef+I+G TIM2+I+G TIM3ef+I+G GTR+I+G TIM2+G TPM2+I+G TPM2+G TIM1ef+I+G TrN+G TIM1+G TVMef+G TrN+I+G TVMef+I+G TPM2uf+I+G TPM2uf+G TIM3+I+G K80+G TIM3+G TIM1+I+G TPM3+I+G K80+I+G TPM3+G TVM+I+G TVM+G TPM1+G TPM1+I+G HKY+G HKY+I+G TPM3uf+I+G TPM3uf+G TPM1uf+G TPM1uf+I+G TIM2ef+I SYM+I TrNef+I GTR+I TIM2+I TIM3ef+I TPM2+I TIM1ef+I TPM2uf+I TVMef+I TrN+I K80+I TIM3+I TIM1+I TPM3+I TVM+I TPM1+I HKY+I TPM3uf+I TPM1uf+I TIM2 TIM2ef TrN GTR SYM TIM3 TIM3ef TIM1 TrNef TIM1ef TPM2uf HKY TVM TPM3uf TPM1uf TPM2 K80 TVMef TPM3 TPM1 JC+G F81+I+G JC+I+G F81+G JC+I F81+I JC F81 ]

* AICc MODEL SELECTION : Parameter importance

Parameter Importance

----------------------

fA 0.0110

fC 0.0110

fG 0.0110

fT 0.0110

kappa 0.0000

titv 0.0000

rAC 0.9290

rAG 1.0000

rAT 0.9339

rCG 0.6188

rCT 1.0000

rGT 1.0000

pinv(I) 0.0000

alpha(G) 0.7585

pinv(IG) 0.2415

alpha(IG) 0.2415

----------------------

Values have been rounded.

(I): considers only +I models.

(G): considers only +G models.

(IG): considers only +I+G models.

* AICc MODEL SELECTION : Best Model's command line

phyml -i /tmp/jmodeltest13117230865493229200.phy -d nt -n 1 -b 0 --run_id SYM+G -m 012345 -f 0.25,0.25,0.25,0.25 -c 4 -a e --no_memory_check -o tlr -s BEST

---------------------------------------------------------------

* *

* MODEL AVERAGED PHYLOGENY *

* *

---------------------------------------------------------------

Selection criterion: . . . . AICc

Confidence interval: . . . . 1.00

Consensus type:. . . . . . . 50% majority rule

Using 88 models in the 1.00 confidence interval = SYM+G TIM2ef+I+G TIM2ef+G TrNef+G SYM+I+G TIM1ef+G TIM3ef+G GTR+G TrNef+I+G TIM2+I+G TIM3ef+I+G GTR+I+G TIM2+G TPM2+I+G TPM2+G TIM1ef+I+G TrN+G TIM1+G TVMef+G TrN+I+G TVMef+I+G TPM2uf+I+G TPM2uf+G TIM3+I+G K80+G TIM3+G TIM1+I+G TPM3+I+G K80+I+G TPM3+G TVM+I+G TVM+G TPM1+G TPM1+I+G HKY+G HKY+I+G TPM3uf+I+G TPM3uf+G TPM1uf+G TPM1uf+I+G TIM2ef+I SYM+I TrNef+I GTR+I TIM2+I TIM3ef+I TPM2+I TIM1ef+I TPM2uf+I TVMef+I TrN+I K80+I TIM3+I TIM1+I TPM3+I TVM+I TPM1+I HKY+I TPM3uf+I TPM1uf+I TIM2 TIM2ef TrN GTR SYM TIM3 TIM3ef TIM1 TrNef TIM1ef TPM2uf HKY TVM TPM3uf TPM1uf TPM2 K80 TVMef TPM3 TPM1 JC+G F81+I+G JC+I+G F81+G JC+I F81+I JC F81

Species in order:

1. lepidioides_18

2. spinosum_23

3. MW791191_armenum

4. armenum_3

5. MT799724_karamanicum

6. karamanicum_43

7. schistosum_21

8. demirizii_40

9. subulatum_20

10. alanyae_39

11. spicatum_35

12. DQ518386_elongatum

13. glaucinum_41

14. FM180113

15. Szowitsii_51

16. elongatum_12

17. virgatum_26

18. sintenisii_48

19. MW791193_grandiflorum

20. stenopterum_31

21. eunomioides_10

22. huber-morathii_42

23. capitatumi_4

24. MT799726_huber-morathii

25. MT799723_grandiflorum

26. umbellatum_32

27. grandiflorum_14

28. edentulum_53

29. membranaceum_33Copen

30. coridifolium_7

31. thomasianum_54

32. MT799725_cordifolium

33. saxatile_34

34. HM1454_Aethionema_S1865

35. Shirkuh_J2

36. W_0184833_Aethionema_ITS

37. S658_Sp_nova_HM478

38. Umbellatum_Archibold_J1

39. Lycium_13

40. fimbriatum_44

41. semnanense_50

42. munzurense_46

43. stylosum_19

44. froedinii_11

45. AY254539_arabicum

46. arabicum_2

47. heterocarpum_15

48. EU938532_carneum_ITS1

49. retsina_38

50. turcicum_1

51. MW791189_turcicum

52. acarii_37

53. cordatum_6

54. MT799718_dumanii

55. MW791190_dumanii

56. dumanii_9

57. MF543734_thomasianum

58. orbiculatum_33

59. GQ284853_saxatile

60. rhodopaeum_24

61. syriacum_27

Bipartitions included in the consensus tree

1234567891111111111222222222233333333334444444444555555555566

0123456789012345678901234567890123456789012345678901

************************************************************* ( 1.0 )

--*********************************************************** ( 1.0 )

--******************************----------------------------- ( 0.99988 )

--***********------------------------------------------------ ( 0.8919 )

--********--------------------------------------------------- ( 1.0 )

--**--------------------------------------------------------- ( 1.0 )

----******--------------------------------------------------- ( 1.0 )

----**------------------------------------------------------- ( 1.0 )

------****--------------------------------------------------- ( 1.0 )

-------***--------------------------------------------------- ( 0.75855 )

--------**--------------------------------------------------- ( 1.0 )

----------***------------------------------------------------ ( 0.8919 )

-----------**------------------------------------------------ ( 1.0 )

-------------*******************----------------------------- ( 1.0 )

-------------****************-------------------------------- ( 1.0 )

--------------***************-------------------------------- ( 1.0 )

--------------**********------------------------------------- ( 1.0 )

--------------******----------------------------------------- ( 0.63926 )

--------------***-------------------------------------------- ( 1.0 )

---------------**-------------------------------------------- ( 1.0 )

-----------------***----------------------------------------- ( 1.0 )

------------------**----------------------------------------- ( 1.0 )

--------------------****------------------------------------- ( 0.58501 )

---------------------***------------------------------------- ( 1.0 )

----------------------**------------------------------------- ( 1.0 )

------------------------*****-------------------------------- ( 1.0 )

-------------------------****-------------------------------- ( 1.0 )

--------------------------***-------------------------------- ( 1.0 )

---------------------------**-------------------------------- ( 1.0 )

-----------------------------***----------------------------- ( 1.0 )

------------------------------**----------------------------- ( 0.76817 )

--------------------------------***************************** ( 1.0 )

--------------------------------******----------------------- ( 1.0 )

---------------------------------*****----------------------- ( 1.0 )

---------------------------------***------------------------- ( 0.75855 )

----------------------------------**------------------------- ( 1.0 )

------------------------------------**----------------------- ( 0.75855 )

--------------------------------------*********************** ( 0.57283 )

--------------------------------------**--------------------- ( 1.0 )

----------------------------------------********************* ( 0.57809 )

----------------------------------------***------------------ ( 0.57809 )

-----------------------------------------**------------------ ( 0.99895 )

-------------------------------------------****************** ( 0.57283 )

-------------------------------------------*************----- ( 0.57809 )

-------------------------------------------*****------------- ( 1.0 )

-------------------------------------------***--------------- ( 1.0 )

--------------------------------------------**--------------- ( 1.0 )

----------------------------------------------**------------- ( 1.0 )

------------------------------------------------********----- ( 0.57809 )

-------------------------------------------------*******----- ( 1.0 )

-------------------------------------------------***--------- ( 1.0 )

--------------------------------------------------**--------- ( 1.0 )

----------------------------------------------------****----- ( 1.0 )

-----------------------------------------------------***----- ( 1.0 )

------------------------------------------------------**----- ( 1.0 )

--------------------------------------------------------***** ( 0.57878 )

--------------------------------------------------------**--- ( 1.0 )

----------------------------------------------------------*** ( 1.0 )

-----------------------------------------------------------** ( 1.0 )

+--61 glaucinum_41

+-115

| +----60 DQ518386_elongatum

+-116

| +--59 spicatum_35

+-117

| | +--58 karamanicum_43

| | +-112

| | | +--57 MT799724_karamanicum

| | +-113

| | | | +--56 alanyae_39

| | | | +-109

| | | | | +--55 subulatum_20

| | | | +-110

| | | | | +--54 demirizii_40

| | | +-111

| | | +--53 schistosum_21

| +-114

| | +--52 armenum_3

| +-108

| +--51 MW791191_armenum

+-118

| | +--50 MT799726_huber-morathii

| | +-101

| | | +--49 capitatumi_4

| | +-102

| | | +--48 huber-morathii_42

| | +-103

| | | +--47 eunomioides_10

| | +-104

| | | | +--46 virgatum_26

| | | | +--98

| | | | | +--45 elongatum_12

| | | | +--99

| | | | | +--44 Szowitsii_51

| | | +-100

| | | | +--43 stenopterum_31

| | | | +--96

| | | | | +--42 MW791193_grandiflorum

| | | +--97

| | | +--41 sintenisii_48

| | +-105

| | | | +--40 membranaceum_33Copen

| | | | +--92

| | | | | +--39 edentulum_53

| | | | +--93

| | | | | +--38 grandiflorum_14

| | | | +--94

| | | | | +--37 umbellatum_32

| | | +--95

| | | +--36 MT799723_grandiflorum

| | +-106

| | | +----35 FM180113

| +-107

| | +--34 MT799725_cordifolium

| | +--90

| | | +--33 thomasianum_54

| +--91

| +--32 coridifolium_7

+----------------------119

| | +--31 dumanii_9

| | +--80

| | | +--30 MW791190_dumanii

| | +--81

| | | +--29 MT799718_dumanii

| | +--82

| | | +--28 cordatum_6

| | +--83

| | | | +--27 acarii_37

| | | | +--78

| | | | | +--26 MW791189_turcicum

| | | +--79

| | | +--25 turcicum_1

| | +--84

| | | +------24 retsina_38

| | +--85

| | | | +--23 EU938532_carneum_ITS1

| | | | +--76

| | | | | +--22 heterocarpum_15

| | | +--77

| | | | +--21 arabicum_2

| | | | +--74

| | | | | +--20 AY254539_arabicum

| | | +--75

| | | +--19 froedinii_11

| | +--86

| | | | +--18 orbiculatum_33

| | | | +--72

| | | | | +--17 MF543734_thomasianum

| | | +--73

| | | | +--16 syriacum_27

| | | | +--70

| | | | | +--15 rhodopaeum_24

| | | +--71

| | | +--14 GQ284853_saxatile

| | +--87

| | | | +--13 stylosum_19

| | | | +--68

| | | | | +--12 munzurense_46

| | | +--69

| | | +----11 semnanense_50

| | +--88

| | | | +--10 fimbriatum_44

| | | +--67

| | | +---9 Lycium_13

| +------89

| | +---8 W_0184833_Aethionema_ITS

| | +--63

| | | +---7 Shirkuh_J2

| | +--64

| | | +---6 HM1454_Aethionema_S1865

| | +--65

| | | | +---5 Umbellatum_Archibold_J1

| | | +--62

| | | +---4 S658_Sp_nova_HM478

| +--66

| +---3 saxatile_34

|

+---------2 spinosum_23

|

+----1 lepidioides_18

(lepidioides_18:0.0154442200,spinosum_23:0.0315945700,(((saxatile_34:0.0088917300,((S658_Sp_nova_HM478:0.0073905000,Umbellatum_Archibold_J1:0.0021519400):0.7585:0.0000000800,(HM1454_Aethionema_S1865:0.0017273500,(Shirkuh_J2:0.0035024500,W_0184833_Aethionema_ITS:0.0017293900):1.0000:0.0034748900):0.7585:0.0000000800):1.0000:0.0016974000):1.0000:0.0017560800,((Lycium_13:0.0124881100,fimbriatum_44:0.0106928600):1.0000:0.0016134800,((semnanense_50:0.0179750200,(munzurense_46:0.0103014100,stylosum_19:0.0097934700):0.9989:0.0029799000):0.5781:0.0017105000,(((GQ284853_saxatile:0.0104330100,(rhodopaeum_24:0.0000000100,syriacum_27:0.0000000100):1.0000:0.0050305200):1.0000:0.0023248700,(MF543734_thomasianum:0.0019379800,orbiculatum_33:0.0143296600):1.0000:0.0033707000):0.5788:0.0000001000,(((froedinii_11:0.0017322700,(AY254539_arabicum:0.0034863300,arabicum_2:0.0017326900):1.0000:0.0017439600):1.0000:0.0038422200,(heterocarpum_15:0.0103651600,EU938532_carneum_ITS1:0.0055751600):1.0000:0.0017618200):1.0000:0.0084720600,(retsina_38:0.0247576100,((turcicum_1:0.0000001100,(MW791189_turcicum:0.0000000100,acarii_37:0.0069898200):1.0000:0.0034952800):1.0000:0.0017229600,(cordatum_6:0.0087308900,(MT799718_dumanii:0.0036471600,(MW791190_dumanii:0.0000000100,dumanii_9:0.0000000900):1.0000:0.0034818800):1.0000:0.0035456900):1.0000:0.0017376900):1.0000:0.0052592900):0.5781:0.0000001600):0.5781:0.0018135700):0.5728:0.0000001100):0.5781:0.0000000800):0.5728:0.0000001100):1.0000:0.0235129100,(((coridifolium_7:0.0000000100,(thomasianum_54:0.0000000100,MT799725_cordifolium:0.0000001000):0.7682:0.0000000100):1.0000:0.0092993500,(FM180113:0.0195469200,((MT799723_grandiflorum:0.0092895200,(umbellatum_32:0.0103057800,(grandiflorum_14:0.0017555900,(edentulum_53:0.0035226500,membranaceum_33Copen:0.0035154000):1.0000:0.0000001000):1.0000:0.0021346800):1.0000:0.0036654600):1.0000:0.0055021200,(((sintenisii_48:0.0053132300,(MW791193_grandiflorum:0.0000001300,stenopterum_31:0.0052794500):1.0000:0.0017503200):1.0000:0.0053155000,(Szowitsii_51:0.0000001200,(elongatum_12:0.0017429100,virgatum_26:0.0000001000):1.0000:0.0017445700):1.0000:0.0124522700):0.6393:0.0000000900,(eunomioides_10:0.0142869000,(huber-morathii_42:0.0000000100,(capitatumi_4:0.0000000100,MT799726_huber-morathii:0.0000000900):1.0000:0.0000000100):1.0000:0.0052749200):0.5850:0.0000000900):1.0000:0.0000001100):1.0000:0.0024575100):1.0000:0.0097405300):1.0000:0.0020165500,(((MW791191_armenum:0.0018065500,armenum_3:0.0053643000):1.0000:0.0126707800,((schistosum_21:0.0035044000,(demirizii_40:0.0052465200,(subulatum_20:0.0017532600,alanyae_39:0.0017428900):1.0000:0.0017389800):0.7585:0.0000000800):1.0000:0.0017805500,(MT799724_karamanicum:0.0017400300,karamanicum_43:0.0053807500):1.0000:0.0035572700):1.0000:0.0018044800):1.0000:0.0046300200,(spicatum_35:0.0095054600,(DQ518386_elongatum:0.0179507700,glaucinum_41:0.0099376200):1.0000:0.0057452600):0.8919:0.0016690000):0.8919:0.0035949500):0.9999:0.0104462000):1.0000:0.0728620500);

Note: this tree is unrooted. Branch lengths are the expected number of substitutions per site. Labels next to parentheses represent phylogenetic uncertainty due to model selection (see documentation)

---------------------------------------------------------------

* *

* BAYESIAN INFORMATION CRITERION (BIC) *

* *

---------------------------------------------------------------

Sample size: 636.0

Model selected:

Model = TrNef+G

partition = 010020

-lnL = 2979.8108

K = 123

R(a) [AC] = 1.0000

R(b) [AG] = 3.1763

R(c) [AT] = 1.0000

R(d) [CG] = 1.0000

R(e) [CT] = 6.1648

R(f) [GT] = 1.0000

gamma shape = 0.3520

--

PAUP* Commands Block:

If you want to load the selected model and associated estimates in PAUP*,

attach the next block of commands after the data in your PAUP file:

[!

Likelihood settings from best-fit model (TrNef+G) selected by BIC

with jModeltest 2.1.10 v20160303 on Thu Dec 29 23:05:45 PST 2022]

BEGIN PAUP;

Lset base=equal nst=6 rmat=(1.0000 3.1763 1.0000 1.0000 6.1648) rates=gamma shape=0.3520 ncat=4 pinvar=0;

END;

--

Tree for the best BIC model = (lepidioides_18:0.01536945,spinosum_23:0.03064658,((((FM180113:0.01909245,(((huber-morathii_42:0.00000001,(capitatumi_4:0.00000001,MT799726_huber-morathii:0.00000009):0.00000001):0.00518876,(eunomioides_10:0.01406435,((Szowitsii_51:0.00000012,(elongatum_12:0.00172236,virgatum_26:0.00000010):0.00172259):0.01226745,(sintenisii_48:0.00523374,(MW791193_grandiflorum:0.00000020,stenopterum_31:0.00520235):0.00172542):0.00522605):0.00000008):0.00000011):0.00000008,(MT799723_grandiflorum:0.00911218,(umbellatum_32:0.01014471,(grandiflorum_14:0.00173214,(edentulum_53:0.00347258,membranaceum_33Copen:0.00346666):0.00000012):0.00209835):0.00362416):0.00539583):0.00248093):0.00953813,(coridifolium_7:0.00000001,(thomasianum_54:0.00000001,MT799725_cordifolium:0.00000008):0.00000001):0.00907465):0.00198368,(((MW791191_armenum:0.00177314,armenum_3:0.00525924):0.01244372,((MT799724_karamanicum:0.00170716,karamanicum_43:0.00528337):0.00348592,(schistosum_21:0.00344208,(demirizii_40:0.00514873,(subulatum_20:0.00171901,alanyae_39:0.00171025):0.00170651):0.00000008):0.00175023):0.00176174):0.00453376,(spicatum_35:0.00934700,(DQ518386_elongatum:0.01765430,glaucinum_41:0.00970779):0.00563991):0.00164203):0.00352052):0.01035878,(((stylosum_19:0.00952752,munzurense_46:0.01011889):0.00301044,(retsina_38:0.02335540,semnanense_50:0.01673657):0.00090868):0.00194118,((((MF543734_thomasianum:0.00207126,orbiculatum_33:0.01399396):0.00324670,(saxatile_34:0.00873864,((HM1454_Aethionema_S1865:0.00169752,(Shirkuh_J2:0.00343523,W_0184833_Aethionema_ITS:0.00169989):0.00341515):0.00000009,(S658_Sp_nova_HM478:0.00726266,Umbellatum_Archibold_J1:0.00210719):0.00000011):0.00166789):0.00172600):0.00000010,((((froedinii_11:0.00170176,(AY254539_arabicum:0.00342521,arabicum_2:0.00170184):0.00171413):0.00376470,(heterocarpum_15:0.01018063,EU938532_carneum_ITS1:0.00547010):0.00172796):0.00838862,((turcicum_1:0.00000011,(MW791189_turcicum:0.00000001,acarii_37:0.00685440):0.00342198):0.00168955,(cordatum_6:0.00854845,(MT799718_dumanii:0.00356523,(MW791190_dumanii:0.00000001,dumanii_9:0.00000009):0.00341411):0.00346189):0.00171784):0.00520864):0.00166413,(Lycium_13:0.01229780,fimbriatum_44:0.01050213):0.00158988):0.00000012):0.00000010,(GQ284853_saxatile:0.01021771,(rhodopaeum_24:0.00000001,syriacum_27:0.00000001):0.00493764):0.00229003):0.00000016):0.02282971):0.07001802);

* BIC MODEL SELECTION : Selection uncertainty

Model -lnL K BIC delta weight cumWeight

-------------------------------------------------------------------------

TrNef+G 2979.81085 123 6753.611123 0.000000 0.600745 0.600745

TIM2ef+G 2977.60125 124 6755.647122 2.035999 0.217059 0.817804

TIM2ef+I+G 2975.35643 125 6757.612680 4.001557 0.081239 0.899043

SYM+G 2972.80480 126 6758.964619 5.353496 0.041323 0.940366

TIM1ef+G 2979.78812 124 6760.020862 6.409739 0.024369 0.964735

TIM3ef+G 2980.47692 124 6761.398462 7.787339 0.012237 0.976973

TPM2+G 2983.84973 123 6761.688883 8.077760 0.010583 0.987556

TrNef+I+G 2980.96028 124 6762.365182 8.754059 0.007547 0.995103

TPM2+I+G 2982.20498 124 6764.854582 11.243459 0.002174 0.997277

K80+G 2989.26834 122 6766.070905 12.459781 0.001183 0.998460

TIM3ef+I+G 2980.16928 125 6767.238380 13.627257 0.000660 0.999120

SYM+I+G 2974.67032 127 6769.150858 15.539734 0.000254 0.999374

TIM1ef+I+G 2981.12615 125 6769.152120 15.540997 0.000254 0.999627

K80+I+G 2988.45824 123 6770.905903 17.294780 0.000105 0.999733

TPM3+G 2988.59069 123 6771.170803 17.559680 9.24e-005 0.999825

TPM1+G 2989.23895 123 6772.467323 18.856200 4.83e-005 0.999873

TVMef+G 2983.13374 125 6773.167300 19.556177 3.41e-005 0.999907

TPM3+I+G 2986.63599 124 6773.716602 20.105479 2.59e-005 0.999933

TrN+G 2980.38711 126 6774.129239 20.518116 2.10e-005 0.999954

TIM2+G 2977.38690 127 6774.584018 20.972894 1.68e-005 0.999971

TIM2+I+G 2975.19220 128 6776.649816 23.038693 5.97e-006 0.999977

TVMef+I+G 2981.65942 126 6776.673859 23.062736 5.90e-006 0.999983

TPM2uf+G 2981.90626 126 6777.167539 23.556416 4.61e-006 0.999988

TPM1+I+G 2988.43516 124 6777.314942 23.703819 4.28e-006 0.999992

GTR+G 2972.79037 129 6778.301355 24.690231 2.61e-006 0.999994

TIM1+G 2979.67010 127 6779.150418 25.539294 1.71e-006 0.999996

TrN+I+G 2980.06770 127 6779.945618 26.334494 1.15e-006 0.999997

TPM2uf+I+G 2980.12149 127 6780.053198 26.442074 1.09e-006 0.999998

HKY+G 2986.97194 125 6780.843700 27.232577 7.33e-007 0.999999

TIM3+G 2981.77005 127 6783.350318 29.739194 2.09e-007 0.999999

TIM2ef+I 2991.82038 124 6784.085382 30.474259 1.45e-007 0.999999

GTR+I+G 2972.45694 130 6784.089693 30.478570 1.45e-007 1.000000

TIM3+I+G 2979.19389 128 6784.653196 31.042073 1.09e-007 1.000000

HKY+I+G 2985.96738 126 6785.289779 31.678656 7.94e-008 1.000000

TrNef+I 2996.10155 123 6786.192523 32.581400 5.06e-008 1.000000

TPM3uf+G 2986.52508 126 6786.405179 32.794056 4.55e-008 1.000000

TIM1+I+G 2980.35709 128 6786.979596 33.368473 3.41e-008 1.000000

TPM1uf+G 2986.93052 126 6787.216059 33.604936 3.03e-008 1.000000

TPM3uf+I+G 2984.47662 127 6788.763458 35.152334 1.40e-008 1.000000

TVM+G 2981.41844 128 6789.102296 35.491173 1.18e-008 1.000000

TPM2+I 2997.97542 123 6789.940263 36.329140 7.76e-009 1.000000

TPM1uf+I+G 2985.93394 127 6791.678098 38.066974 3.26e-009 1.000000

TVM+I+G 2979.47930 129 6791.679215 38.068091 3.25e-009 1.000000

TIM3ef+I 2996.39079 124 6793.226202 39.615079 1.50e-009 1.000000

K80+I 3003.04891 122 6793.632045 40.020921 1.23e-009 1.000000

SYM+I 2990.34617 126 6794.047359 40.436236 9.96e-010 1.000000

TIM1ef+I 2996.92577 124 6794.296162 40.685039 8.79e-010 1.000000

TPM3+I 3002.91776 123 6799.824943 46.213820 5.54e-011 1.000000

TPM1+I 3003.32454 123 6800.638503 47.027380 3.69e-011 1.000000

TVMef+I 2997.50920 125 6801.918220 48.307097 1.95e-011 1.000000

TIM2+I 2991.34522 127 6802.500658 48.889534 1.45e-011 1.000000

TPM2uf+I 2995.77105 126 6804.897119 51.285996 4.39e-012 1.000000

TrN+I 2996.23495 126 6805.824919 52.213796 2.76e-012 1.000000

GTR+I 2987.71004 129 6808.140695 54.529571 8.66e-013 1.000000

HKY+I 3000.85853 125 6808.616880 55.005757 6.83e-013 1.000000

TIM3+I 2995.99983 127 6811.809878 58.198754 1.38e-013 1.000000

TIM1+I 2996.08350 127 6811.977218 58.366094 1.27e-013 1.000000

TPM3uf+I 3000.63496 126 6814.624939 61.013816 3.39e-014 1.000000

TPM1uf+I 3000.82171 126 6814.998439 61.387316 2.81e-014 1.000000

TVM+I 2995.51843 128 6817.302276 63.691153 8.88e-015 1.000000

TIM2ef 3060.66761 123 6915.324643 161.713520 4.60e-036 1.000000

TrNef 3064.87776 122 6917.289745 163.678621 1.72e-036 1.000000

TIM3ef 3062.92466 123 6919.838743 166.227620 4.82e-037 1.000000

TIM1ef 3063.36690 123 6920.723223 167.112100 3.10e-037 1.000000

TIM2 3055.13104 126 6923.617099 170.005976 7.28e-038 1.000000

TrN 3058.52666 125 6923.953140 170.342017 6.16e-038 1.000000

SYM 3059.45164 125 6925.803100 172.191977 2.44e-038 1.000000

TIM3 3058.19774 126 6929.750499 176.139376 3.39e-039 1.000000

TIM1 3058.33235 126 6930.019719 176.408596 2.97e-039 1.000000

TPM2 3071.33843 122 6930.211085 176.599961 2.69e-039 1.000000

K80 3075.57205 121 6932.223126 178.612003 9.85e-040 1.000000

TPM2uf 3062.81331 125 6932.526440 178.915317 8.47e-040 1.000000

HKY 3066.35066 124 6933.145942 179.534819 6.21e-040 1.000000

GTR 3054.70242 128 6935.670256 182.059133 1.76e-040 1.000000

TPM3 3075.23417 122 6938.002565 184.391441 5.48e-041 1.000000

JC+G 3078.63020 121 6938.339426 184.728303 4.63e-041 1.000000

TPM1 3075.54644 122 6938.627105 185.015981 4.01e-041 1.000000

TPM3uf 3066.06808 125 6939.035980 185.424857 3.27e-041 1.000000

TPM1uf 3066.15339 125 6939.206600 185.595477 3.00e-041 1.000000

TVMef 3070.99524 124 6942.435102 188.823979 5.97e-042 1.000000

JC+I+G 3078.51345 122 6944.561125 190.950001 2.06e-042 1.000000

TVM 3062.42654 127 6944.663298 191.052174 1.96e-042 1.000000

F81+I+G 3073.21446 125 6953.328740 199.717617 2.57e-044 1.000000

F81+G 3076.81016 124 6954.064942 200.453819 1.78e-044 1.000000

JC+I 3091.98176 121 6965.042546 211.431423 7.36e-047 1.000000

F81+I 3090.41888 124 6981.282382 227.671259 2.19e-050 1.000000

JC 3161.44041 120 7097.504648 343.893524 1.27e-075 1.000000

F81 3157.11316 123 7108.215743 354.604620 5.99e-078 1.000000

-------------------------------------------------------------------------

-lnL: negative log likelihod

K: number of estimated parameters

BIC: Bayesian Information Criterion

delta: BIC difference

weight: BIC weight

cumWeight: cumulative BIC weight

* BIC MODEL SELECTION : Confidence interval

There are 88 models in the 100% confidence interval: [ TrNef+G TIM2ef+G TIM2ef+I+G SYM+G TIM1ef+G TIM3ef+G TPM2+G TrNef+I+G TPM2+I+G K80+G TIM3ef+I+G SYM+I+G TIM1ef+I+G K80+I+G TPM3+G TPM1+G TVMef+G TPM3+I+G TrN+G TIM2+G TIM2+I+G TVMef+I+G TPM2uf+G TPM1+I+G GTR+G TIM1+G TrN+I+G TPM2uf+I+G HKY+G TIM3+G TIM2ef+I GTR+I+G TIM3+I+G HKY+I+G TrNef+I TPM3uf+G TIM1+I+G TPM1uf+G TPM3uf+I+G TVM+G TPM2+I TPM1uf+I+G TVM+I+G TIM3ef+I K80+I SYM+I TIM1ef+I TPM3+I TPM1+I TVMef+I TIM2+I TPM2uf+I TrN+I GTR+I HKY+I TIM3+I TIM1+I TPM3uf+I TPM1uf+I TVM+I TIM2ef TrNef TIM3ef TIM1ef TIM2 TrN SYM TIM3 TIM1 TPM2 K80 TPM2uf HKY GTR TPM3 JC+G TPM1 TPM3uf TPM1uf TVMef JC+I+G TVM F81+I+G F81+G JC+I F81+I JC F81 ]

* BIC MODEL SELECTION : Parameter importance

Parameter Importance

----------------------

fA 0.0001

fC 0.0001

fG 0.0001

fT 0.0001

kappa 0.0013

titv 0.0013

rAC 0.3657

rAG 0.9987

rAT 0.3774

rCG 0.0793

rCT 0.9987

rGT 0.9987

pinv(I) 0.0000

alpha(G) 0.9077

pinv(IG) 0.0923

alpha(IG) 0.0923

----------------------

Values have been rounded.

(I): considers only +I models.

(G): considers only +G models.

(IG): considers only +I+G models.

* BIC MODEL SELECTION : Best Model's command line

phyml -i /tmp/jmodeltest13117230865493229200.phy -d nt -n 1 -b 0 --run_id TrNef+G -m 010020 -f 0.25,0.25,0.25,0.25 -c 4 -a e --no_memory_check -o tlr -s BEST

---------------------------------------------------------------

* *

* MODEL AVERAGED PHYLOGENY *

* *

---------------------------------------------------------------

Selection criterion: . . . . BIC

Confidence interval: . . . . 1.00

Consensus type:. . . . . . . 50% majority rule

Using 88 models in the 1.00 confidence interval = TrNef+G TIM2ef+G TIM2ef+I+G SYM+G TIM1ef+G TIM3ef+G TPM2+G TrNef+I+G TPM2+I+G K80+G TIM3ef+I+G SYM+I+G TIM1ef+I+G K80+I+G TPM3+G TPM1+G TVMef+G TPM3+I+G TrN+G TIM2+G TIM2+I+G TVMef+I+G TPM2uf+G TPM1+I+G GTR+G TIM1+G TrN+I+G TPM2uf+I+G HKY+G TIM3+G TIM2ef+I GTR+I+G TIM3+I+G HKY+I+G TrNef+I TPM3uf+G TIM1+I+G TPM1uf+G TPM3uf+I+G TVM+G TPM2+I TPM1uf+I+G TVM+I+G TIM3ef+I K80+I SYM+I TIM1ef+I TPM3+I TPM1+I TVMef+I TIM2+I TPM2uf+I TrN+I GTR+I HKY+I TIM3+I TIM1+I TPM3uf+I TPM1uf+I TVM+I TIM2ef TrNef TIM3ef TIM1ef TIM2 TrN SYM TIM3 TIM1 TPM2 K80 TPM2uf HKY GTR TPM3 JC+G TPM1 TPM3uf TPM1uf TVMef JC+I+G TVM F81+I+G F81+G JC+I F81+I JC F81

Species in order:

1. lepidioides_18

2. spinosum_23

3. FM180113

4. huber-morathii_42

5. capitatumi_4

6. MT799726_huber-morathii

7. eunomioides_10

8. Szowitsii_51

9. elongatum_12

10. virgatum_26

11. sintenisii_48

12. MW791193_grandiflorum

13. stenopterum_31

14. MT799723_grandiflorum

15. umbellatum_32

16. grandiflorum_14

17. edentulum_53

18. membranaceum_33Copen

19. coridifolium_7

20. thomasianum_54

21. MT799725_cordifolium

22. MW791191_armenum

23. armenum_3

24. MT799724_karamanicum

25. karamanicum_43

26. schistosum_21

27. demirizii_40

28. subulatum_20

29. alanyae_39

30. spicatum_35

31. DQ518386_elongatum

32. glaucinum_41

33. stylosum_19

34. munzurense_46

35. retsina_38

36. semnanense_50

37. MF543734_thomasianum

38. orbiculatum_33

39. saxatile_34

40. HM1454_Aethionema_S1865

41. Shirkuh_J2

42. W_0184833_Aethionema_ITS

43. S658_Sp_nova_HM478

44. Umbellatum_Archibold_J1

45. froedinii_11

46. AY254539_arabicum

47. arabicum_2

48. heterocarpum_15

49. EU938532_carneum_ITS1

50. turcicum_1

51. MW791189_turcicum

52. acarii_37

53. cordatum_6

54. MT799718_dumanii

55. MW791190_dumanii

56. dumanii_9

57. Lycium_13

58. fimbriatum_44

59. GQ284853_saxatile

60. rhodopaeum_24

61. syriacum_27

Bipartitions included in the consensus tree

1234567891111111111222222222233333333334444444444555555555566

0123456789012345678901234567890123456789012345678901

************************************************************* ( 1.0 )

--*********************************************************** ( 1.0 )

--******************************----------------------------- ( 1.0 )

--*******************---------------------------------------- ( 1.0 )

--****************------------------------------------------- ( 1.0 )

---***************------------------------------------------- ( 1.0 )

---**********------------------------------------------------ ( 1.0 )

---***------------------------------------------------------- ( 1.0 )

----**------------------------------------------------------- ( 1.0 )

------*******------------------------------------------------ ( 0.60078 )

-------******------------------------------------------------ ( 0.66499 )

-------***--------------------------------------------------- ( 1.0 )

--------**--------------------------------------------------- ( 1.0 )

----------***------------------------------------------------ ( 1.0 )

-----------**------------------------------------------------ ( 1.0 )

-------------*****------------------------------------------- ( 1.0 )

--------------****------------------------------------------- ( 1.0 )

---------------***------------------------------------------- ( 1.0 )

----------------**------------------------------------------- ( 1.0 )

------------------***---------------------------------------- ( 1.0 )

-------------------**---------------------------------------- ( 0.91844 )

---------------------***********----------------------------- ( 0.77098 )

---------------------********-------------------------------- ( 1.0 )

---------------------**-------------------------------------- ( 1.0 )

-----------------------******-------------------------------- ( 1.0 )

-----------------------**------------------------------------ ( 1.0 )

-------------------------****-------------------------------- ( 1.0 )

--------------------------***-------------------------------- ( 0.90773 )

---------------------------**-------------------------------- ( 1.0 )

-----------------------------***----------------------------- ( 0.77098 )

------------------------------**----------------------------- ( 1.0 )

--------------------------------***************************** ( 1.0 )

--------------------------------****------------------------- ( 0.70739 )

--------------------------------**--------------------------- ( 0.99783 )

----------------------------------**------------------------- ( 0.94673 )

------------------------------------************************* ( 0.60075 )

------------------------------------**********************--- ( 0.60075 )

------------------------------------********----------------- ( 0.60075 )

------------------------------------**----------------------- ( 1.0 )

--------------------------------------******----------------- ( 1.0 )

---------------------------------------*****----------------- ( 1.0 )

---------------------------------------***------------------- ( 0.90773 )

----------------------------------------**------------------- ( 1.0 )

------------------------------------------**----------------- ( 0.90773 )

--------------------------------------------**************--- ( 0.60075 )

--------------------------------------------************----- ( 0.62512 )

--------------------------------------------*****------------ ( 1.0 )

--------------------------------------------***-------------- ( 1.0 )

---------------------------------------------**-------------- ( 1.0 )

-----------------------------------------------**------------ ( 1.0 )

-------------------------------------------------*******----- ( 1.0 )

-------------------------------------------------***--------- ( 1.0 )

--------------------------------------------------**--------- ( 1.0 )

----------------------------------------------------****----- ( 1.0 )

-----------------------------------------------------***----- ( 1.0 )

------------------------------------------------------**----- ( 1.0 )

--------------------------------------------------------**--- ( 1.0 )

----------------------------------------------------------*** ( 1.0 )

-----------------------------------------------------------** ( 1.0 )

+--61 glaucinum_41

+-115

| +----60 DQ518386_elongatum

+-116

| +--59 spicatum_35

+-117

| | +--58 armenum_3

| | +-113

| | | +--57 MW791191_armenum

| +-114

| | +--56 alanyae_39

| | +-109

| | | +--55 subulatum_20

| | +-110

| | | +--54 demirizii_40

| | +-111

| | | +--53 schistosum_21

| +-112

| | +--52 karamanicum_43

| +-108

| +--51 MT799724_karamanicum

+-118

| | +--50 MT799725_cordifolium

| | +-105

| | | +--49 thomasianum_54

| | +-106

| | | +--48 coridifolium_7

| +-107

| | +--47 stenopterum_31

| | +--98

| | | +--46 MW791193_grandiflorum

| | +--99

| | | +--45 sintenisii_48

| | +-100

| | | | +--44 virgatum_26

| | | | +--96

| | | | | +--43 elongatum_12

| | | +--97

| | | +--42 Szowitsii_51

| | +-101

| | | +--41 eunomioides_10

| | +-102

| | | | +--40 MT799726_huber-morathii

| | | | +--94

| | | | | +--39 capitatumi_4

| | | +--95

| | | +--38 huber-morathii_42

| | +-103

| | | | +--37 membranaceum_33Copen

| | | | +--90

| | | | | +--36 edentulum_53

| | | | +--91

| | | | | +--35 grandiflorum_14

| | | | +--92

| | | | | +--34 umbellatum_32

| | | +--93

| | | +--33 MT799723_grandiflorum

| +-104

| +----32 FM180113

+---------------------119

| | +--31 dumanii_9

| | +--81

| | | +--30 MW791190_dumanii

| | +--82

| | | +--29 MT799718_dumanii

| | +--83

| | | +--28 cordatum_6

| | +--84

| | | | +--27 acarii_37

| | | | +--79

| | | | | +--26 MW791189_turcicum

| | | +--80

| | | +--25 turcicum_1

| | +--85

| | | | +--24 arabicum_2

| | | | +--76

| | | | | +--23 AY254539_arabicum

| | | | +--77

| | | | | +--22 froedinii_11

| | | +--78

| | | | +--21 EU938532_carneum_ITS1

| | | +--75

| | | +--20 heterocarpum_15

| | +--86

| | | | +--19 fimbriatum_44

| | | +--74

| | | +--18 Lycium_13

| | +--87

| | | | +--17 orbiculatum_33

| | | | +--72

| | | | | +--16 MF543734_thomasianum

| | | +--73

| | | | +--15 W_0184833_Aethionema_ITS

| | | | +--68

| | | | | +--14 Shirkuh_J2

| | | | +--69

| | | | | +--13 HM1454_Aethionema_S1865

| | | | +--70

| | | | | | +--12 Umbellatum_Archibold_J1

| | | | | +--67

| | | | | +--11 S658_Sp_nova_HM478

| | | +--71

| | | +--10 saxatile_34

| | +--88

| | | | +---9 syriacum_27

| | | | +--65

| | | | | +---8 rhodopaeum_24

| | | +--66

| | | +---7 GQ284853_saxatile

| +-----89

| | +----6 semnanense_50

| | +--63

| | | +-------5 retsina_38

| +--64

| | +---4 munzurense_46

| +--62

| +---3 stylosum_19

|

+---------2 spinosum_23

|

+----1 lepidioides_18

(lepidioides_18:0.0153694500,spinosum_23:0.0306465800,((((stylosum_19:0.0095275200,munzurense_46:0.0101188900):0.9978:0.0030104400,(retsina_38:0.0233554000,semnanense_50:0.0167365700):0.9467:0.0009086800):0.7074:0.0019411800,((GQ284853_saxatile:0.0102177100,(rhodopaeum_24:0.0000000100,syriacum_27:0.0000000100):1.0000:0.0049376400):1.0000:0.0022900300,(((saxatile_34:0.0087386400,((S658_Sp_nova_HM478:0.0072626600,Umbellatum_Archibold_J1:0.0021071900):0.9077:0.0000001100,(HM1454_Aethionema_S1865:0.0016975200,(Shirkuh_J2:0.0034352300,W_0184833_Aethionema_ITS:0.0016998900):1.0000:0.0034151500):0.9077:0.0000000900):1.0000:0.0016678900):1.0000:0.0017260000,(MF543734_thomasianum:0.0020712600,orbiculatum_33:0.0139939600):1.0000:0.0032467000):0.6007:0.0000001000,((Lycium_13:0.0122978000,fimbriatum_44:0.0105021300):1.0000:0.0015898800,(((heterocarpum_15:0.0101806300,EU938532_carneum_ITS1:0.0054701000):1.0000:0.0017279600,(froedinii_11:0.0017017600,(AY254539_arabicum:0.0034252100,arabicum_2:0.0017018400):1.0000:0.0017141300):1.0000:0.0037647000):1.0000:0.0083886200,((turcicum_1:0.0000001100,(MW791189_turcicum:0.0000000100,acarii_37:0.0068544000):1.0000:0.0034219800):1.0000:0.0016895500,(cordatum_6:0.0085484500,(MT799718_dumanii:0.0035652300,(MW791190_dumanii:0.0000000100,dumanii_9:0.0000000900):1.0000:0.0034141100):1.0000:0.0034618900):1.0000:0.0017178400):1.0000:0.0052086400):0.6251:0.0016641300):0.6007:0.0000001200):0.6007:0.0000001000):0.6007:0.0000001600):1.0000:0.0228297100,(((FM180113:0.0190924500,((MT799723_grandiflorum:0.0091121800,(umbellatum_32:0.0101447100,(grandiflorum_14:0.0017321400,(edentulum_53:0.0034725800,membranaceum_33Copen:0.0034666600):1.0000:0.0000001200):1.0000:0.0020983500):1.0000:0.0036241600):1.0000:0.0053958300,((huber-morathii_42:0.0000000100,(capitatumi_4:0.0000000100,MT799726_huber-morathii:0.0000000900):1.0000:0.0000000100):1.0000:0.0051887600,(eunomioides_10:0.0140643500,((Szowitsii_51:0.0000001200,(elongatum_12:0.0017223600,virgatum_26:0.0000001000):1.0000:0.0017225900):1.0000:0.0122674500,(sintenisii_48:0.0052337400,(MW791193_grandiflorum:0.0000002000,stenopterum_31:0.0052023500):1.0000:0.0017254200):1.0000:0.0052260500):0.6650:0.0000000800):0.6008:0.0000001100):1.0000:0.0000000800):1.0000:0.0024809300):1.0000:0.0095381300,(coridifolium_7:0.0000000100,(thomasianum_54:0.0000000100,MT799725_cordifolium:0.0000000800):0.9184:0.0000000100):1.0000:0.0090746500):1.0000:0.0019836800,((((MT799724_karamanicum:0.0017071600,karamanicum_43:0.0052833700):1.0000:0.0034859200,(schistosum_21:0.0034420800,(demirizii_40:0.0051487300,(subulatum_20:0.0017190100,alanyae_39:0.0017102500):1.0000:0.0017065100):0.9077:0.0000000800):1.0000:0.0017502300):1.0000:0.0017617400,(MW791191_armenum:0.0017731400,armenum_3:0.0052592400):1.0000:0.0124437200):1.0000:0.0045337600,(spicatum_35:0.0093470000,(DQ518386_elongatum:0.0176543000,glaucinum_41:0.0097077900):1.0000:0.0056399100):0.7710:0.0016420300):0.7710:0.0035205200):1.0000:0.0103587800):1.0000:0.0700180200);

Note: this tree is unrooted. Branch lengths are the expected number of substitutions per site. Labels next to parentheses represent phylogenetic uncertainty due to model selection (see documentation)

---------------------------------------------------------------

* *

* DECISION THEORY PERFORMANCE-BASED SELECTION (DT) *

* *

---------------------------------------------------------------

Sample size: 636.0

Model selected:

Model = TrNef+G

partition = 010020

-lnL = 2979.8108

K = 123

R(a) [AC] = 1.0000

R(b) [AG] = 3.1763

R(c) [AT] = 1.0000

R(d) [CG] = 1.0000

R(e) [CT] = 6.1648

R(f) [GT] = 1.0000

gamma shape = 0.3520

--

PAUP* Commands Block:

If you want to load the selected model and associated estimates in PAUP*,

attach the next block of commands after the data in your PAUP file:

[!

Likelihood settings from best-fit model (TrNef+G) selected by DT

with jModeltest 2.1.10 v20160303 on Thu Dec 29 23:05:45 PST 2022]

BEGIN PAUP;

Lset base=equal nst=6 rmat=(1.0000 3.1763 1.0000 1.0000 6.1648) rates=gamma shape=0.3520 ncat=4 pinvar=0;

END;

--

Tree for the best DT model = (lepidioides_18:0.01536945,spinosum_23:0.03064658,((((FM180113:0.01909245,(((huber-morathii_42:0.00000001,(capitatumi_4:0.00000001,MT799726_huber-morathii:0.00000009):0.00000001):0.00518876,(eunomioides_10:0.01406435,((Szowitsii_51:0.00000012,(elongatum_12:0.00172236,virgatum_26:0.00000010):0.00172259):0.01226745,(sintenisii_48:0.00523374,(MW791193_grandiflorum:0.00000020,stenopterum_31:0.00520235):0.00172542):0.00522605):0.00000008):0.00000011):0.00000008,(MT799723_grandiflorum:0.00911218,(umbellatum_32:0.01014471,(grandiflorum_14:0.00173214,(edentulum_53:0.00347258,membranaceum_33Copen:0.00346666):0.00000012):0.00209835):0.00362416):0.00539583):0.00248093):0.00953813,(coridifolium_7:0.00000001,(thomasianum_54:0.00000001,MT799725_cordifolium:0.00000008):0.00000001):0.00907465):0.00198368,(((MW791191_armenum:0.00177314,armenum_3:0.00525924):0.01244372,((MT799724_karamanicum:0.00170716,karamanicum_43:0.00528337):0.00348592,(schistosum_21:0.00344208,(demirizii_40:0.00514873,(subulatum_20:0.00171901,alanyae_39:0.00171025):0.00170651):0.00000008):0.00175023):0.00176174):0.00453376,(spicatum_35:0.00934700,(DQ518386_elongatum:0.01765430,glaucinum_41:0.00970779):0.00563991):0.00164203):0.00352052):0.01035878,(((stylosum_19:0.00952752,munzurense_46:0.01011889):0.00301044,(retsina_38:0.02335540,semnanense_50:0.01673657):0.00090868):0.00194118,((((MF543734_thomasianum:0.00207126,orbiculatum_33:0.01399396):0.00324670,(saxatile_34:0.00873864,((HM1454_Aethionema_S1865:0.00169752,(Shirkuh_J2:0.00343523,W_0184833_Aethionema_ITS:0.00169989):0.00341515):0.00000009,(S658_Sp_nova_HM478:0.00726266,Umbellatum_Archibold_J1:0.00210719):0.00000011):0.00166789):0.00172600):0.00000010,((((froedinii_11:0.00170176,(AY254539_arabicum:0.00342521,arabicum_2:0.00170184):0.00171413):0.00376470,(heterocarpum_15:0.01018063,EU938532_carneum_ITS1:0.00547010):0.00172796):0.00838862,((turcicum_1:0.00000011,(MW791189_turcicum:0.00000001,acarii_37:0.00685440):0.00342198):0.00168955,(cordatum_6:0.00854845,(MT799718_dumanii:0.00356523,(MW791190_dumanii:0.00000001,dumanii_9:0.00000009):0.00341411):0.00346189):0.00171784):0.00520864):0.00166413,(Lycium_13:0.01229780,fimbriatum_44:0.01050213):0.00158988):0.00000012):0.00000010,(GQ284853_saxatile:0.01021771,(rhodopaeum_24:0.00000001,syriacum_27:0.00000001):0.00493764):0.00229003):0.00000016):0.02282971):0.07001802);

* DT MODEL SELECTION : Selection uncertainty

Model -lnL K DT delta weight cumWeight

-------------------------------------------------------------------------

TrNef+G 2979.81085 123 0.027391 0.000000 0.024871 0.024871

TIM2ef+G 2977.60125 124 0.051873 0.024482 0.013133 0.038004

K80+I 3003.04891 122 0.052686 0.025295 0.012930 0.050935

TVM+G 2981.41844 128 0.054161 0.026770 0.012578 0.063513

TIM2+G 2977.38690 127 0.055327 0.027936 0.012313 0.075826

K80+G 2989.26834 122 0.055402 0.028012 0.012296 0.088122

TPM1+G 2989.23895 123 0.055407 0.028016 0.012295 0.100418

TIM3+G 2981.77005 127 0.056010 0.028619 0.012163 0.112581

HKY+G 2986.97194 125 0.056111 0.028720 0.012141 0.124722

TPM1uf+G 2986.93052 126 0.056116 0.028725 0.012140 0.136862

GTR+I 2987.71004 129 0.056185 0.028794 0.012125 0.148987

TIM3ef 3062.92466 123 0.056197 0.028806 0.012123 0.161110

TIM1ef 3063.36690 123 0.056236 0.028845 0.012114 0.173224

JC 3161.44041 120 0.056296 0.028905 0.012101 0.185325

TPM3+G 2988.59069 123 0.056590 0.029199 0.012038 0.197364

TPM2uf+G 2981.90626 126 0.056740 0.029349 0.012006 0.209370

JC+G 3078.63020 121 0.056795 0.029404 0.011995 0.221365

TPM2+G 2983.84973 123 0.056835 0.029445 0.011986 0.233351

TIM3ef+I 2996.39079 124 0.056984 0.029593 0.011955 0.245306

TIM1+I 2996.08350 127 0.058424 0.031033 0.011661 0.256967

TPM3uf+G 2986.52508 126 0.058943 0.031552 0.011558 0.268525

SYM 3059.45164 125 0.059267 0.031876 0.011495 0.280019

TVMef 3070.99524 124 0.059454 0.032063 0.011458 0.291478

TrN+I 2996.23495 126 0.059566 0.032175 0.011437 0.302915

TIM2ef 3060.66761 123 0.059580 0.032189 0.011434 0.314349

TrNef 3064.87776 122 0.059602 0.032211 0.011430 0.325779

TPM3 3075.23417 122 0.059612 0.032221 0.011428 0.337207

K80 3075.57205 121 0.059640 0.032249 0.011423 0.348630

TPM1 3075.54644 122 0.059645 0.032254 0.011422 0.360051

TIM1ef+I 2996.92577 124 0.059653 0.032262 0.011420 0.371472

F81+G 3076.81016 124 0.060014 0.032624 0.011351 0.382823

TPM2 3071.33843 122 0.060081 0.032690 0.011339 0.394162

TrNef+I 2996.10155 123 0.060207 0.032816 0.011315 0.405477

TPM3uf+I+G 2984.47662 127 0.060242 0.032851 0.011309 0.416786

TrN+I+G 2980.06770 127 0.060256 0.032865 0.011306 0.428092

SYM+I 2990.34617 126 0.060266 0.032875 0.011304 0.439396

TrNef+I+G 2980.96028 124 0.060386 0.032995 0.011282 0.450677

TPM3+I+G 2986.63599 124 0.060399 0.033008 0.011279 0.461957

TPM2uf+I+G 2980.12149 127 0.060449 0.033058 0.011270 0.473227

JC+I 3091.98176 121 0.060554 0.033163 0.011250 0.484477

TVMef+G 2983.13374 125 0.060727 0.033336 0.011218 0.495695

JC+I+G 3078.51345 122 0.061289 0.033898 0.011115 0.506810

F81+I+G 3073.21446 125 0.061337 0.033946 0.011107 0.517917

TIM2+I 2991.34522 127 0.061352 0.033962 0.011104 0.529021

TIM3+I 2995.99983 127 0.061430 0.034039 0.011090 0.540111

TIM2ef+I 2991.82038 124 0.061441 0.034051 0.011088 0.551199

TVM+I 2995.51843 128 0.061547 0.034156 0.011069 0.562267

TPM1uf+I 3000.82171 126 0.061567 0.034176 0.011065 0.573333

TPM2uf+I 2995.77105 126 0.061631 0.034240 0.011054 0.584386

HKY+I 3000.85853 125 0.061672 0.034281 0.011046 0.595433

TPM3uf+I 3000.63496 126 0.061721 0.034330 0.011038 0.606470

TVMef+I 2997.50920 125 0.061773 0.034382 0.011028 0.617498

TPM2+I 2997.97542 123 0.061803 0.034412 0.011023 0.628521

TPM1+I 3003.32454 123 0.061829 0.034438 0.011018 0.639540

GTR+G 2972.79037 129 0.061833 0.034442 0.011018 0.650557

TPM3+I 3002.91776 123 0.061857 0.034466 0.011013 0.661570

F81+I 3090.41888 124 0.062014 0.034623 0.010985 0.672556

HKY+I+G 2985.96738 126 0.062476 0.035085 0.010904 0.683460

TPM1uf+I+G 2985.93394 127 0.062479 0.035088 0.010904 0.694364

TIM2+I+G 2975.19220 128 0.062518 0.035127 0.010897 0.705260

TIM2ef+I+G 2975.35643 125 0.062537 0.035146 0.010894 0.716154

TPM1+I+G 2988.43516 124 0.062560 0.035169 0.010889 0.727043

K80+I+G 2988.45824 123 0.062568 0.035177 0.010888 0.737932

TVMef+I+G 2981.65942 126 0.062652 0.035262 0.010873 0.748805

TPM2+I+G 2982.20498 124 0.062984 0.035593 0.010816 0.759621

TIM1+I+G 2980.35709 128 0.062985 0.035594 0.010816 0.770438

SYM+I+G 2974.67032 127 0.063028 0.035637 0.010809 0.781246

SYM+G 2972.80480 126 0.063048 0.035658 0.010805 0.792051

TIM1ef+I+G 2981.12615 125 0.063083 0.035692 0.010799 0.802851

TIM3ef+I+G 2980.16928 125 0.063155 0.035764 0.010787 0.813638

GTR+I+G 2972.45694 130 0.064455 0.037064 0.010569 0.824207

TIM3 3058.19774 126 0.064575 0.037184 0.010550 0.834757

TrN 3058.52666 125 0.064600 0.037210 0.010546 0.845302

GTR 3054.70242 128 0.064606 0.037215 0.010545 0.855847

TIM2 3055.13104 126 0.064620 0.037230 0.010542 0.866389

TPM3uf 3066.06808 125 0.064702 0.037312 0.010529 0.876918

TVM 3062.42654 127 0.064721 0.037330 0.010526 0.887444

HKY 3066.35066 124 0.064725 0.037334 0.010525 0.897970

TPM1uf 3066.15339 125 0.064737 0.037346 0.010523 0.908493

TIM1ef+G 2979.78812 124 0.064863 0.037472 0.010503 0.918996

TIM3ef+G 2980.47692 124 0.064972 0.037581 0.010485 0.929481

F81 3157.11316 123 0.065526 0.038135 0.010397 0.939878

TrN+G 2980.38711 126 0.065798 0.038407 0.010354 0.950231

TIM1 3058.33235 126 0.065921 0.038530 0.010334 0.960566

TIM3+I+G 2979.19389 128 0.068288 0.040898 0.009976 0.970542

TPM2uf 3062.81331 125 0.068602 0.041211 0.009931 0.980472

TVM+I+G 2979.47930 129 0.069025 0.041634 0.009870 0.990342

TIM1+G 2979.67010 127 0.070537 0.043146 0.009658 1.000000

-------------------------------------------------------------------------

-lnL:t negative log likelihod

K: number of estimated parameters

DT: decision theory performance-based score

delta: DT difference

weight: DT weight* (calculated using 1/DT)

cumWeight: cumulative DT weight

* DT MODEL SELECTION : Confidence interval

There are 88 models in the 100% confidence interval: [ TrNef+G TIM2ef+G K80+I TVM+G TIM2+G K80+G TPM1+G TIM3+G HKY+G TPM1uf+G GTR+I TIM3ef TIM1ef JC TPM3+G TPM2uf+G JC+G TPM2+G TIM3ef+I TIM1+I TPM3uf+G SYM TVMef TrN+I TIM2ef TrNef TPM3 K80 TPM1 TIM1ef+I F81+G TPM2 TrNef+I TPM3uf+I+G TrN+I+G SYM+I TrNef+I+G TPM3+I+G TPM2uf+I+G JC+I TVMef+G JC+I+G F81+I+G TIM2+I TIM3+I TIM2ef+I TVM+I TPM1uf+I TPM2uf+I HKY+I TPM3uf+I TVMef+I TPM2+I TPM1+I GTR+G TPM3+I F81+I HKY+I+G TPM1uf+I+G TIM2+I+G TIM2ef+I+G TPM1+I+G K80+I+G TVMef+I+G TPM2+I+G TIM1+I+G SYM+I+G SYM+G TIM1ef+I+G TIM3ef+I+G GTR+I+G TIM3 TrN GTR TIM2 TPM3uf TVM HKY TPM1uf TIM1ef+G TIM3ef+G F81 TrN+G TIM1 TIM3+I+G TPM2uf TVM+I+G TIM1+G ]

* DT MODEL SELECTION : Parameter importance

Parameter Importance

----------------------

fA 0.4848

fC 0.4848

fG 0.4848

fT 0.4848

kappa 0.0922

titv 0.0922

rAC 0.5374

rAG 0.8175

rAT 0.5357

rCG 0.5342

rCT 0.8175

rGT 0.8175

pinv(I) 0.2490

alpha(G) 0.2689

pinv(IG) 0.2394

alpha(IG) 0.2394

----------------------

Values have been rounded.

(I): considers only +I models.

(G): considers only +G models.

(IG): considers only +I+G models.

* DT MODEL SELECTION : Best Model's command line

phyml -i /tmp/jmodeltest13117230865493229200.phy -d nt -n 1 -b 0 --run_id TrNef+G -m 010020 -f 0.25,0.25,0.25,0.25 -c 4 -a e --no_memory_check -o tlr -s BEST

---------------------------------------------------------------

* *

* MODEL AVERAGED PHYLOGENY *

* *

---------------------------------------------------------------

Selection criterion: . . . . DT

Confidence interval: . . . . 1.00

Consensus type:. . . . . . . 50% majority rule

Using 88 models in the 1.00 confidence interval = TrNef+G TIM2ef+G K80+I TVM+G TIM2+G K80+G TPM1+G TIM3+G HKY+G TPM1uf+G GTR+I TIM3ef TIM1ef JC TPM3+G TPM2uf+G JC+G TPM2+G TIM3ef+I TIM1+I TPM3uf+G SYM TVMef TrN+I TIM2ef TrNef TPM3 K80 TPM1 TIM1ef+I F81+G TPM2 TrNef+I TPM3uf+I+G TrN+I+G SYM+I TrNef+I+G TPM3+I+G TPM2uf+I+G JC+I TVMef+G JC+I+G F81+I+G TIM2+I TIM3+I TIM2ef+I TVM+I TPM1uf+I TPM2uf+I HKY+I TPM3uf+I TVMef+I TPM2+I TPM1+I GTR+G TPM3+I F81+I HKY+I+G TPM1uf+I+G TIM2+I+G TIM2ef+I+G TPM1+I+G K80+I+G TVMef+I+G TPM2+I+G TIM1+I+G SYM+I+G SYM+G TIM1ef+I+G TIM3ef+I+G GTR+I+G TIM3 TrN GTR TIM2 TPM3uf TVM HKY TPM1uf TIM1ef+G TIM3ef+G F81 TrN+G TIM1 TIM3+I+G TPM2uf TVM+I+G TIM1+G

Species in order:

1. lepidioides_18

2. spinosum_23

3. FM180113

4. huber-morathii_42

5. capitatumi_4

6. MT799726_huber-morathii

7. eunomioides_10

8. Szowitsii_51

9. elongatum_12

10. virgatum_26

11. sintenisii_48

12. MW791193_grandiflorum

13. stenopterum_31

14. MT799723_grandiflorum

15. umbellatum_32

16. grandiflorum_14

17. edentulum_53

18. membranaceum_33Copen

19. coridifolium_7

20. thomasianum_54

21. MT799725_cordifolium

22. MW791191_armenum

23. armenum_3

24. MT799724_karamanicum

25. karamanicum_43

26. schistosum_21

27. demirizii_40

28. subulatum_20

29. alanyae_39

30. spicatum_35

31. DQ518386_elongatum

32. glaucinum_41

33. stylosum_19

34. munzurense_46

35. retsina_38

36. semnanense_50

37. MF543734_thomasianum

38. orbiculatum_33

39. saxatile_34

40. HM1454_Aethionema_S1865

41. Shirkuh_J2

42. W_0184833_Aethionema_ITS

43. S658_Sp_nova_HM478

44. Umbellatum_Archibold_J1

45. froedinii_11

46. AY254539_arabicum

47. arabicum_2

48. heterocarpum_15

49. EU938532_carneum_ITS1

50. turcicum_1

51. MW791189_turcicum

52. acarii_37

53. cordatum_6

54. MT799718_dumanii

55. MW791190_dumanii

56. dumanii_9

57. Lycium_13

58. fimbriatum_44

59. GQ284853_saxatile

60. rhodopaeum_24

61. syriacum_27

Bipartitions included in the consensus tree

1234567891111111111222222222233333333334444444444555555555566

0123456789012345678901234567890123456789012345678901

************************************************************* ( 1.0 )

--*********************************************************** ( 1.0 )

--******************************----------------------------- ( 0.99035 )

--***************************-**----------------------------- ( 0.53366 )

--***************************-------------------------------- ( 0.52326 )

--*******************---------------------------------------- ( 0.88506 )

--****************------------------------------------------- ( 1.0 )

---***************------------------------------------------- ( 0.88506 )

---***------------------------------------------------------- ( 1.0 )

----**------------------------------------------------------- ( 0.75099 )

-------***--------------------------------------------------- ( 1.0 )

--------**--------------------------------------------------- ( 1.0 )

----------***------------------------------------------------ ( 1.0 )

-------------*****------------------------------------------- ( 0.88506 )

--------------****------------------------------------------- ( 1.0 )

---------------***------------------------------------------- ( 1.0 )

----------------**------------------------------------------- ( 0.86685 )

------------------***---------------------------------------- ( 1.0 )

-------------------**---------------------------------------- ( 0.70677 )

---------------------********-------------------------------- ( 1.0 )

---------------------**-------------------------------------- ( 1.0 )

-----------------------******-------------------------------- ( 1.0 )

-----------------------**------------------------------------ ( 1.0 )

-------------------------****-------------------------------- ( 1.0 )

---------------------------**-------------------------------- ( 1.0 )

------------------------------**----------------------------- ( 1.0 )

--------------------------------***************************** ( 1.0 )

--------------------------------**--************************* ( 0.521 )

--------------------------------**---------------*******----- ( 0.67141 )

--------------------------------**--------------------------- ( 0.83889 )

----------------------------------**------------------------- ( 0.63895 )

------------------------------------**----------------------- ( 1.0 )

--------------------------------------******----------------- ( 1.0 )

---------------------------------------*****----------------- ( 0.90631 )

----------------------------------------**------------------- ( 1.0 )

------------------------------------------**----------------- ( 0.52306 )

--------------------------------------------*****------------ ( 1.0 )

--------------------------------------------***-------------- ( 1.0 )

---------------------------------------------**-------------- ( 1.0 )

-----------------------------------------------**------------ ( 1.0 )

-------------------------------------------------*******----- ( 1.0 )

-------------------------------------------------***--------- ( 1.0 )

--------------------------------------------------**--------- ( 1.0 )

----------------------------------------------------****----- ( 1.0 )

-----------------------------------------------------***----- ( 1.0 )

------------------------------------------------------**----- ( 1.0 )

--------------------------------------------------------**--- ( 1.0 )

----------------------------------------------------------*** ( 1.0 )

-----------------------------------------------------------** ( 1.0 )

+--61 MT799725_cordifolium

+-103

| +--60 thomasianum_54

+-104

| +--59 coridifolium_7

+-105

| | +--58 membranaceum_33Copen

| | +--97

| | | +--57 edentulum_53

| | +--98

| | | +--56 grandiflorum_14

| | +--99

| | | +--55 umbellatum_32

| | +-100

| | | +--54 MT799723_grandiflorum

| | |

| | | +--53 stenopterum_31

| | | |

| | | +--52 MW791193_grandiflorum

| | +--96

| | | +--51 sintenisii_48

| | |

| | | +--50 virgatum_26

| | | +--94

| | | | +--49 elongatum_12

| | +--95

| | | +--48 Szowitsii_51

| | +-101

| | | | +--47 MT799726_huber-morathii

| | | | +--92

| | | | | +--46 capitatumi_4

| | | +--93

| | | | +--45 huber-morathii_42

| | | |

| | | +--44 eunomioides_10

| +-102

| +----43 FM180113

+-106

| | +--42 armenum_3

| | +--90

| | | +--41 MW791191_armenum

| +--91

| | +--40 alanyae_39

| | +--87

| | | +--39 subulatum_20

| | |

| | +--38 demirizii_40

| | +--88

| | | +--37 schistosum_21

| +--89

| | +--36 karamanicum_43

| +--86

| +--35 MT799724_karamanicum

+-107

| | +--34 glaucinum_41

| +--85

| +----33 DQ518386_elongatum

+-108

| +--32 spicatum_35

+--------------------109

| | +--31 munzurense_46

| | +--81

| | | +--30 stylosum_19

| | +--82

| | | | +--29 dumanii_9

| | | | +--77

| | | | | +--28 MW791190_dumanii

| | | | +--78

| | | | | +--27 MT799718_dumanii

| | | | +--79

| | | | | +--26 cordatum_6

| | | +--80

| | | | +--25 acarii_37

| | | | +--75

| | | | | +--24 MW791189_turcicum

| | | +--76

| | | +--23 turcicum_1

| | |

| | | +--22 fimbriatum_44

| | +--74

| | | +--21 Lycium_13

| | |

| | | +--20 orbiculatum_33

| | +--73

| | | +--19 MF543734_thomasianum

| | +--83

| | | | +--18 arabicum_2

| | | | +--70

| | | | | +--17 AY254539_arabicum

| | | | +--71

| | | | | +--16 froedinii_11

| | | +--72

| | | | | +--15 EU938532_carneum_ITS1

| | | | +--69

| | | | +--14 heterocarpum_15

| | | |

| | | | +--13 Umbellatum_Archibold_J1

| | | | +--66

| | | | | +--12 S658_Sp_nova_HM478

| | | | |

| | | | | +--11 W_0184833_Aethionema_ITS

| | | | +--65

| | | | | +--10 Shirkuh_J2

| | | | +--67

| | | | | +---9 HM1454_Aethionema_S1865

| | | +--68

| | | | +---8 saxatile_34

| | | |

| | | | +---7 syriacum_27

| | | | +--63

| | | | | +---6 rhodopaeum_24

| | | +--64

| | | +---5 GQ284853_saxatile

| +-----84

| | +----4 semnanense_50

| +--62

| +------3 retsina_38

|

+---------2 spinosum_23

|

+---1 lepidioides_18

(lepidioides_18:0.0141104700,spinosum_23:0.0306901700,(((retsina_38:0.0217993100,semnanense_50:0.0162713900):0.6389:0.0015688800,((GQ284853_saxatile:0.0100984200,(rhodopaeum_24:0.0000000100,syriacum_27:0.0000000100):1.0000:0.0048179300):1.0000:0.0022749500,(saxatile_34:0.0086362300,(HM1454_Aethionema_S1865:0.0016747400,(Shirkuh_J2:0.0033917900,W_0184833_Aethionema_ITS:0.0016769500):1.0000:0.0033729800,(S658_Sp_nova_HM478:0.0071609900,Umbellatum_Archibold_J1:0.0020922400):0.5231:0.0000001000):0.9063:0.0016581200):1.0000:0.0017042400,((heterocarpum_15:0.0100972900,EU938532_carneum_ITS1:0.0053545200):1.0000:0.0016962800,(froedinii_11:0.0016803400,(AY254539_arabicum:0.0033846800,arabicum_2:0.0016785700):1.0000:0.0016933300):1.0000:0.0036617500):1.0000:0.0090300400,(MF543734_thomasianum:0.0020047700,orbiculatum_33:0.0138252200):1.0000:0.0032031000,(Lycium_13:0.0121674400,fimbriatum_44:0.0103978900):1.0000:0.0015668400,(((turcicum_1:0.0000000700,(MW791189_turcicum:0.0000000100,acarii_37:0.0067604300):1.0000:0.0033773300):1.0000:0.0016650000,(cordatum_6:0.0084393600,(MT799718_dumanii:0.0035370600,(MW791190_dumanii:0.0000000100,dumanii_9:0.0000000900):1.0000:0.0033690300):1.0000:0.0034215500):1.0000:0.0016813200):1.0000:0.0044196300,(stylosum_19:0.0094518200,munzurense_46:0.0097483800):0.8389:0.0024377300):0.6714:0.0034131300):0.5210:0.0029869900):1.0000:0.0225543600,(spicatum_35:0.0073591000,((DQ518386_elongatum:0.0174814800,glaucinum_41:0.0094755400):1.0000:0.0053611500,((((MT799724_karamanicum:0.0017063500,karamanicum_43:0.0051532800):1.0000:0.0034256600,(schistosum_21:0.0033985500,demirizii_40:0.0050817900,(subulatum_20:0.0016954700,alanyae_39:0.0016886300):1.0000:0.0016826400):1.0000:0.0017148400):1.0000:0.0017068500,(MW791191_armenum:0.0017239200,armenum_3:0.0051671100):1.0000:0.0122481900):1.0000:0.0035706200,((FM180113:0.0184824400,(eunomioides_10:0.0138914000,(huber-morathii_42:0.0000000100,(capitatumi_4:0.0000000100,MT799726_huber-morathii:0.0000000800):0.7510:0.0000000100):1.0000:0.0051203400,(Szowitsii_51:0.0000000800,(elongatum_12:0.0017007100,virgatum_26:0.0000000900):1.0000:0.0017001700):1.0000:0.0121041800,(sintenisii_48:0.0053132300,MW791193_grandiflorum:0.0000001600,stenopterum_31:0.0051263700):1.0000:0.0051276400,(MT799723_grandiflorum:0.0090107800,(umbellatum_32:0.0098925000,(grandiflorum_14:0.0017083600,(edentulum_53:0.0034260800,membranaceum_33Copen:0.0034087300):0.8668:0.0000000800):1.0000:0.0021076000):1.0000:0.0034398800):0.8851:0.0053050700):0.8851:0.0031898300):1.0000:0.0091445800,(coridifolium_7:0.0000000100,(thomasianum_54:0.0000000100,MT799725_cordifolium:0.0000000900):0.7068:0.0000000100):1.0000:0.0089603300):0.8851:0.0049249400):0.5233:0.0020530100):0.5337:0.0031512700):0.9903:0.0094907100):1.0000:0.0667026700);

Note: this tree is unrooted. Branch lengths are the expected number of substitutions per site. Labels next to parentheses represent phylogenetic uncertainty due to model selection (see documentation)

---------------------------------------------------------------

* *

* SELECTION SUMMARY *

* *

---------------------------------------------------------------

::Optimized Topologies Summary::

There are 60 different topologies.

Topology Id: 1

Rank Weight RF AvgEucl VarEucl

AIC 1 68.56% 0 0.00e+00 0.00e+00

BIC 6 4.13% 16 6.99e-02 0.00e+00

AICc 1 57.28% 0 0.00e+00 0.00e+00

DT 6 1.08% 16 6.99e-02 0.00e+00

Models supporting: 1

SYM+G

Topology Id: 2

Rank Weight RF AvgEucl VarEucl

AIC 2 3.46% 6 1.78e-02 0.00e+00

BIC 7 0.00% 16 6.73e-02 0.00e+00

AICc 2 0.53% 6 1.78e-02 0.00e+00

DT 7 1.10% 16 6.73e-02 0.00e+00

Models supporting: 1

GTR+G

Topology Id: 3

Rank Weight RF AvgEucl VarEucl

AIC 3 0.24% 12 4.96e-02 0.00e+00

BIC 3 1.22% 14 7.19e-02 0.00e+00

AICc 3 0.60% 12 4.96e-02 0.00e+00

DT 3 1.05% 14 7.19e-02 0.00e+00

Models supporting: 1

TIM3ef+G

Topology Id: 4

Rank Weight RF AvgEucl VarEucl

AIC 4 1.25% 16 6.99e-02 0.00e+00

BIC 1 60.07% 0 0.00e+00 0.00e+00

AICc 4 5.42% 16 6.99e-02 0.00e+00

DT 1 2.49% 0 0.00e+00 0.00e+00

Models supporting: 1

TrNef+G

Topology Id: 5

Rank Weight RF AvgEucl VarEucl

AIC 5 0.02% 18 5.06e-02 1.13e-08

BIC 14 1.07% 20 6.21e-02 1.86e-08

AICc 5 0.10% 18 5.06e-02 1.13e-08

DT 14 2.40% 20 6.21e-02 1.86e-08

Models supporting: 2

TPM2+G TPM3+G

Topology Id: 6

Rank Weight RF AvgEucl VarEucl

AIC 6 0.47% 18 5.94e-02 0.00e+00

BIC 2 2.44% 12 7.00e-02 0.00e+00

AICc 6 1.19% 18 5.94e-02 0.00e+00

DT 2 1.05% 12 7.00e-02 0.00e+00

Models supporting: 1

TIM1ef+G

Topology Id: 7

Rank Weight RF AvgEucl VarEucl

AIC 7 0.00% 20 5.99e-02 0.00e+00

BIC 4 0.00% 16 6.24e-02 0.00e+00

AICc 7 0.00% 20 5.99e-02 0.00e+00

DT 4 1.20% 16 6.24e-02 0.00e+00

Models supporting: 1

JC+G

Topology Id: 8

Rank Weight RF AvgEucl VarEucl

AIC 8 0.03% 20 5.12e-02 0.00e+00

BIC 9 0.00% 18 7.01e-02 0.00e+00

AICc 8 0.03% 20 5.12e-02 0.00e+00

DT 9 1.04% 18 7.01e-02 0.00e+00

Models supporting: 1

TrN+G

Topology Id: 9

Rank Weight RF AvgEucl VarEucl

AIC 9 0.00% 22 6.65e-02 0.00e+00

BIC 12 0.00% 20 6.64e-02 0.00e+00

AICc 9 0.00% 22 6.65e-02 0.00e+00

DT 12 1.14% 20 6.64e-02 0.00e+00

Models supporting: 1

F81+G

Topology Id: 10

Rank Weight RF AvgEucl VarEucl

AIC 10 0.00% 22 5.63e-02 1.19e-12

BIC 17 0.12% 22 5.93e-02 2.77e-12

AICc 10 0.00% 22 5.63e-02 1.19e-12

DT 17 2.46% 22 5.93e-02 2.77e-12

Models supporting: 2

K80+G TPM1+G

Topology Id: 11

Rank Weight RF AvgEucl VarEucl

AIC 11 0.00% 22 6.42e-02 1.77e-12

BIC 18 0.00% 22 6.47e-02 3.72e-12

AICc 11 0.00% 22 6.42e-02 1.77e-12

DT 18 2.43% 22 6.47e-02 3.72e-12

Models supporting: 2

HKY+G TPM1uf+G

Topology Id: 12

Rank Weight RF AvgEucl VarEucl

AIC 12 0.01% 22 5.41e-02 0.00e+00

BIC 21 0.00% 22 6.04e-02 0.00e+00

AICc 12 0.01% 22 5.41e-02 0.00e+00

DT 21 1.20% 22 6.04e-02 0.00e+00

Models supporting: 1

TPM2uf+G

Topology Id: 13

Rank Weight RF AvgEucl VarEucl

AIC 13 4.18% 22 6.25e-02 0.00e+00

BIC 16 21.71% 20 6.75e-02 0.00e+00

AICc 13 10.56% 22 6.25e-02 0.00e+00

DT 16 1.31% 20 6.75e-02 0.00e+00

Models supporting: 1

TIM2ef+G

Topology Id: 14

Rank Weight RF AvgEucl VarEucl

AIC 14 0.00% 24 5.76e-02 6.82e-10

BIC 8 0.00% 18 6.52e-02 4.58e-10

AICc 14 0.00% 24 5.76e-02 6.82e-10

DT 8 2.22% 18 6.52e-02 4.58e-10

Models supporting: 2

JC+I+G F81+I+G

Topology Id: 15

Rank Weight RF AvgEucl VarEucl

AIC 15 0.85% 24 5.16e-02 3.04e-10

BIC 13 0.01% 20 7.10e-02 1.95e-08

AICc 15 0.23% 24 5.16e-02 3.04e-10

DT 13 5.45% 20 7.10e-02 1.95e-08

Models supporting: 5

K80+I+G HKY+I+G TPM1+I+G TPM1uf+I+G TIM2+I+G

Topology Id: 16

Rank Weight RF AvgEucl VarEucl

AIC 16 0.15% 24 5.91e-02 0.00e+00

BIC 19 0.75% 22 6.41e-02 0.00e+00

AICc 16 0.37% 24 5.91e-02 0.00e+00

DT 19 1.13% 22 6.41e-02 0.00e+00

Models supporting: 1

TrNef+I+G

Topology Id: 17

Rank Weight RF AvgEucl VarEucl

AIC 17 0.02% 24 5.89e-02 0.00e+00

BIC 20 0.00% 22 6.39e-02 0.00e+00

AICc 17 0.01% 24 5.89e-02 0.00e+00

DT 20 1.13% 22 6.39e-02 0.00e+00

Models supporting: 1

TrN+I+G

Topology Id: 18

Rank Weight RF AvgEucl VarEucl

AIC 18 0.00% 24 6.78e-02 0.00e+00

BIC 37 0.00% 26 6.37e-02 0.00e+00

AICc 18 0.00% 24 6.78e-02 0.00e+00

DT 37 1.13% 26 6.37e-02 0.00e+00

Models supporting: 1

TPM2

Topology Id: 19

Rank Weight RF AvgEucl VarEucl

AIC 19 0.02% 24 5.05e-02 6.24e-10

BIC 22 0.00% 22 6.21e-02 1.13e-08

AICc 19 0.01% 24 5.05e-02 6.24e-10

DT 22 3.39% 22 6.21e-02 1.13e-08

Models supporting: 3

TPM2uf+I+G TPM3+I+G TPM3uf+I+G

Topology Id: 20

Rank Weight RF AvgEucl VarEucl

AIC 20 0.00% 24 6.54e-02 0.00e+00

BIC 32 0.00% 24 6.65e-02 0.00e+00

AICc 20 0.00% 24 6.54e-02 0.00e+00

DT 32 1.16% 24 6.65e-02 0.00e+00

Models supporting: 1

TPM3uf+G

Topology Id: 21

Rank Weight RF AvgEucl VarEucl

AIC 21 0.17% 24 5.21e-02 2.26e-10

BIC 15 0.09% 20 7.10e-02 9.51e-09

AICc 21 0.24% 24 5.21e-02 2.26e-10

DT 15 3.24% 20 7.10e-02 9.51e-09

Models supporting: 3

TIM1ef+I+G TIM1+I+G TIM3ef+I+G

Topology Id: 22

Rank Weight RF AvgEucl VarEucl

AIC 22 0.03% 24 7.91e-02 0.00e+00

BIC 5 0.00% 16 7.10e-02 0.00e+00

AICc 22 0.01% 24 7.91e-02 0.00e+00

DT 5 0.97% 16 7.10e-02 0.00e+00

Models supporting: 1

TIM1+G

Topology Id: 23

Rank Weight RF AvgEucl VarEucl

AIC 23 0.26% 24 6.49e-02 0.00e+00

BIC 25 0.00% 22 6.54e-02 0.00e+00

AICc 23 0.12% 24 6.49e-02 0.00e+00

DT 25 1.23% 22 6.54e-02 0.00e+00

Models supporting: 1

TIM2+G

Topology Id: 24

Rank Weight RF AvgEucl VarEucl

AIC 24 0.00% 24 6.41e-02 0.00e+00

BIC 33 0.00% 24 6.46e-02 0.00e+00

AICc 24 0.00% 24 6.41e-02 0.00e+00

DT 33 1.22% 24 6.46e-02 0.00e+00

Models supporting: 1

TIM3+G

Topology Id: 25

Rank Weight RF AvgEucl VarEucl

AIC 25 0.02% 24 6.18e-02 0.00e+00

BIC 26 0.00% 22 7.37e-02 0.00e+00

AICc 25 0.00% 24 6.18e-02 0.00e+00

DT 26 1.00% 22 7.37e-02 0.00e+00

Models supporting: 1

TIM3+I+G

Topology Id: 26

Rank Weight RF AvgEucl VarEucl

AIC 26 0.01% 24 5.61e-02 0.00e+00

BIC 27 0.00% 22 6.72e-02 0.00e+00

AICc 26 0.01% 24 5.61e-02 0.00e+00

DT 27 1.12% 22 6.72e-02 0.00e+00

Models supporting: 1

TVMef+G

Topology Id: 27

Rank Weight RF AvgEucl VarEucl

AIC 27 0.01% 24 6.08e-02 0.00e+00

BIC 28 0.00% 22 6.62e-02 0.00e+00

AICc 27 0.01% 24 6.08e-02 0.00e+00

DT 28 1.09% 22 6.62e-02 0.00e+00

Models supporting: 1

TVMef+I+G

Topology Id: 28

Rank Weight RF AvgEucl VarEucl

AIC 28 0.00% 24 6.10e-02 0.00e+00

BIC 34 0.00% 24 5.58e-02 0.00e+00

AICc 28 0.00% 24 6.10e-02 0.00e+00

DT 34 1.26% 24 5.58e-02 0.00e+00

Models supporting: 1

TVM+G

Topology Id: 29

Rank Weight RF AvgEucl VarEucl

AIC 29 0.00% 24 6.13e-02 0.00e+00

BIC 29 0.00% 22 7.43e-02 0.00e+00

AICc 29 0.00% 24 6.13e-02 0.00e+00

DT 29 0.99% 22 7.43e-02 0.00e+00

Models supporting: 1

TVM+I+G

Topology Id: 30

Rank Weight RF AvgEucl VarEucl

AIC 30 0.00% 24 5.71e-02 0.00e+00

BIC 10 0.00% 18 6.24e-02 0.00e+00

AICc 30 0.00% 24 5.71e-02 0.00e+00

DT 10 1.15% 18 6.24e-02 0.00e+00

Models supporting: 1

SYM

Topology Id: 31

Rank Weight RF AvgEucl VarEucl

AIC 31 1.78% 24 5.20e-02 0.00e+00

BIC 11 0.00% 18 6.96e-02 0.00e+00

AICc 31 0.15% 24 5.20e-02 0.00e+00

DT 11 1.06% 18 6.96e-02 0.00e+00

Models supporting: 1

GTR+I+G

Topology Id: 32

Rank Weight RF AvgEucl VarEucl

AIC 32 0.04% 26 5.75e-02 0.00e+00

BIC 31 0.22% 24 6.79e-02 0.00e+00

AICc 32 0.11% 26 5.75e-02 0.00e+00

DT 31 1.08% 24 6.79e-02 0.00e+00

Models supporting: 1

TPM2+I+G

Topology Id: 33

Rank Weight RF AvgEucl VarEucl

AIC 33 14.53% 26 5.16e-02 0.00e+00

BIC 24 8.12% 22 7.13e-02 0.00e+00

AICc 33 21.16% 26 5.16e-02 0.00e+00

DT 24 1.09% 22 7.13e-02 0.00e+00

Models supporting: 1

TIM2ef+I+G

Topology Id: 34

Rank Weight RF AvgEucl VarEucl

AIC 34 3.90% 26 5.92e-02 0.00e+00

BIC 35 0.03% 24 6.67e-02 0.00e+00

AICc 34 1.86% 26 5.92e-02 0.00e+00

DT 35 1.08% 24 6.67e-02 0.00e+00

Models supporting: 1

SYM+I+G

Topology Id: 35

Rank Weight RF AvgEucl VarEucl

AIC 35 0.00% 28 6.41e-02 3.36e-10

BIC 39 0.00% 28 6.57e-02 1.39e-10

AICc 35 0.00% 28 6.41e-02 3.36e-10

DT 39 3.43% 28 6.57e-02 1.39e-10

Models supporting: 3

K80 TPM1 TPM3

Topology Id: 36

Rank Weight RF AvgEucl VarEucl

AIC 36 0.00% 28 6.37e-02 5.14e-10

BIC 36 0.00% 26 6.59e-02 1.91e-12

AICc 36 0.00% 28 6.37e-02 5.14e-10

DT 36 2.29% 26 6.59e-02 1.91e-12

Models supporting: 2

TrNef TIM2ef

Topology Id: 37

Rank Weight RF AvgEucl VarEucl

AIC 37 0.00% 28 5.86e-02 5.62e-10

BIC 23 0.00% 22 5.90e-02 3.57e-10

AICc 37 0.00% 28 5.86e-02 5.62e-10

DT 23 2.42% 22 5.90e-02 3.57e-10

Models supporting: 2

TIM1ef TIM3ef

Topology Id: 38

Rank Weight RF AvgEucl VarEucl

AIC 38 0.00% 28 6.38e-02 0.00e+00

BIC 40 0.00% 28 6.56e-02 0.00e+00

AICc 38 0.00% 28 6.38e-02 0.00e+00

DT 40 1.15% 28 6.56e-02 0.00e+00

Models supporting: 1

TVMef

Topology Id: 39

Rank Weight RF AvgEucl VarEucl

AIC 39 0.00% 30 5.88e-02 0.00e+00

BIC 30 0.00% 24 5.91e-02 0.00e+00

AICc 39 0.00% 30 5.88e-02 0.00e+00

DT 30 1.21% 24 5.91e-02 0.00e+00

Models supporting: 1

JC

Topology Id: 40

Rank Weight RF AvgEucl VarEucl

AIC 40 0.00% 32 5.13e-02 3.31e-09

BIC 41 0.00% 30 6.23e-02 2.56e-09

AICc 40 0.00% 32 5.13e-02 3.31e-09

DT 41 2.26% 30 6.23e-02 2.56e-09

Models supporting: 2

TrNef+I SYM+I

Topology Id: 41

Rank Weight RF AvgEucl VarEucl

AIC 41 0.00% 32 5.40e-02 0.00e+00

BIC 38 0.00% 26 5.52e-02 0.00e+00

AICc 41 0.00% 32 5.40e-02 0.00e+00

DT 38 1.21% 26 5.52e-02 0.00e+00

Models supporting: 1

GTR+I

Topology Id: 42

Rank Weight RF AvgEucl VarEucl

AIC 42 0.00% 34 6.50e-02 0.00e+00

BIC 42 0.00% 32 6.28e-02 0.00e+00

AICc 42 0.00% 34 6.50e-02 0.00e+00

DT 42 1.04% 32 6.28e-02 0.00e+00

Models supporting: 1

F81

Topology Id: 43

Rank Weight RF AvgEucl VarEucl

AIC 43 0.00% 34 5.39e-02 0.00e+00

BIC 43 0.00% 32 5.50e-02 0.00e+00

AICc 43 0.00% 34 5.39e-02 0.00e+00

DT 43 1.29% 32 5.50e-02 0.00e+00

Models supporting: 1

K80+I

Topology Id: 44

Rank Weight RF AvgEucl VarEucl

AIC 44 0.00% 34 6.84e-02 4.29e-09

BIC 44 0.00% 32 6.12e-02 3.31e-09

AICc 44 0.00% 34 6.84e-02 4.29e-09

DT 44 8.43% 32 6.12e-02 3.31e-09

Models supporting: 8

HKY TrN TPM1uf TPM3uf TIM2 TIM3 TVM GTR

Topology Id: 45

Rank Weight RF AvgEucl VarEucl

AIC 45 0.00% 34 7.16e-02 0.00e+00

BIC 49 0.00% 34 6.81e-02 0.00e+00

AICc 45 0.00% 34 7.16e-02 0.00e+00

DT 49 0.99% 34 6.81e-02 0.00e+00

Models supporting: 1

TPM2uf

Topology Id: 46

Rank Weight RF AvgEucl VarEucl

AIC 46 0.00% 34 7.07e-02 0.00e+00

BIC 51 0.00% 34 6.49e-02 0.00e+00

AICc 46 0.00% 34 7.07e-02 0.00e+00

DT 51 1.03% 34 6.49e-02 0.00e+00

Models supporting: 1

TIM1

Topology Id: 47

Rank Weight RF AvgEucl VarEucl

AIC 47 0.00% 36 6.13e-02 0.00e+00

BIC 48 0.00% 34 6.75e-02 0.00e+00

AICc 47 0.00% 36 6.13e-02 0.00e+00

DT 48 1.14% 34 6.75e-02 0.00e+00

Models supporting: 1

TrN+I

Topology Id: 48

Rank Weight RF AvgEucl VarEucl

AIC 48 0.00% 36 6.29e-02 0.00e+00

BIC 50 0.00% 34 6.85e-02 0.00e+00

AICc 48 0.00% 36 6.29e-02 0.00e+00

DT 50 1.11% 34 6.85e-02 0.00e+00

Models supporting: 1

TPM2uf+I

Topology Id: 49

Rank Weight RF AvgEucl VarEucl

AIC 49 0.00% 36 6.27e-02 0.00e+00

BIC 54 0.00% 36 6.87e-02 0.00e+00

AICc 49 0.00% 36 6.27e-02 0.00e+00

DT 54 1.10% 36 6.87e-02 0.00e+00

Models supporting: 1

TPM3+I

Topology Id: 50

Rank Weight RF AvgEucl VarEucl

AIC 50 0.00% 36 6.36e-02 0.00e+00

BIC 52 0.00% 34 6.81e-02 0.00e+00

AICc 50 0.00% 36 6.36e-02 0.00e+00

DT 52 1.11% 34 6.81e-02 0.00e+00

Models supporting: 1

TIM2ef+I

Topology Id: 51

Rank Weight RF AvgEucl VarEucl

AIC 51 0.00% 38 6.22e-02 1.01e-07

BIC 58 0.00% 38 6.85e-02 1.99e-08

AICc 51 0.00% 38 6.22e-02 1.01e-07

DT 58 4.42% 38 6.85e-02 1.99e-08

Models supporting: 4

HKY+I TPM1+I TPM1uf+I TVMef+I

Topology Id: 52

Rank Weight RF AvgEucl VarEucl

AIC 52 0.00% 38 6.14e-02 0.00e+00

BIC 59 0.00% 38 6.81e-02 0.00e+00

AICc 52 0.00% 38 6.14e-02 0.00e+00

DT 59 1.10% 38 6.81e-02 0.00e+00

Models supporting: 1

TPM2+I

Topology Id: 53

Rank Weight RF AvgEucl VarEucl

AIC 53 0.00% 38 6.49e-02 0.00e+00

BIC 60 0.00% 38 6.78e-02 0.00e+00

AICc 53 0.00% 38 6.49e-02 0.00e+00

DT 60 1.10% 38 6.78e-02 0.00e+00

Models supporting: 1

TPM3uf+I

Topology Id: 54

Rank Weight RF AvgEucl VarEucl

AIC 54 0.00% 38 4.56e-02 0.00e+00

BIC 45 0.00% 32 6.42e-02 0.00e+00

AICc 54 0.00% 38 4.56e-02 0.00e+00

DT 45 1.14% 32 6.42e-02 0.00e+00

Models supporting: 1

TIM1ef+I

Topology Id: 55

Rank Weight RF AvgEucl VarEucl

AIC 55 0.00% 38 6.33e-02 0.00e+00

BIC 46 0.00% 32 6.19e-02 0.00e+00

AICc 55 0.00% 38 6.33e-02 0.00e+00

DT 46 1.17% 32 6.19e-02 0.00e+00

Models supporting: 1

TIM1+I

Topology Id: 56

Rank Weight RF AvgEucl VarEucl

AIC 56 0.00% 38 6.26e-02 0.00e+00

BIC 55 0.00% 36 6.81e-02 0.00e+00

AICc 56 0.00% 38 6.26e-02 0.00e+00

DT 55 1.11% 36 6.81e-02 0.00e+00

Models supporting: 1

TIM2+I

Topology Id: 57

Rank Weight RF AvgEucl VarEucl

AIC 57 0.00% 38 6.18e-02 0.00e+00

BIC 47 0.00% 32 6.05e-02 0.00e+00

AICc 57 0.00% 38 6.18e-02 0.00e+00

DT 47 1.20% 32 6.05e-02 0.00e+00

Models supporting: 1

TIM3ef+I

Topology Id: 58

Rank Weight RF AvgEucl VarEucl

AIC 58 0.00% 38 6.29e-02 4.10e-07

BIC 56 0.00% 36 6.84e-02 7.78e-08

AICc 58 0.00% 38 6.29e-02 4.10e-07

DT 56 2.22% 36 6.84e-02 7.78e-08

Models supporting: 2

TIM3+I TVM+I

Topology Id: 59

Rank Weight RF AvgEucl VarEucl

AIC 59 0.00% 40 6.42e-02 0.00e+00

BIC 53 0.00% 36 6.51e-02 0.00e+00

AICc 59 0.00% 40 6.42e-02 0.00e+00

DT 53 1.13% 36 6.51e-02 0.00e+00

Models supporting: 1

JC+I

Topology Id: 60

Rank Weight RF AvgEucl VarEucl

AIC 60 0.00% 40 6.24e-02 0.00e+00

BIC 57 0.00% 38 6.81e-02 0.00e+00

AICc 60 0.00% 40 6.24e-02 0.00e+00

DT 57 1.10% 38 6.81e-02 0.00e+00

Models supporting: 1

F81+I

::Best Models::

Model f(a) f(c) f(g) f(t) kappa titv Ra Rb Rc Rd Re Rf pInv gamma

----------------------------------------------------------------------------------------------------------------------------------------

AIC SYM+G 0.25 0.25 0.25 0.25 0.00 0.00 1.533 4.121 2.091 0.754 8.014 1.000 N/A 0.35

BIC TrNef+G 0.25 0.25 0.25 0.25 0.00 0.00 1.000 3.176 1.000 1.000 6.165 1.000 N/A 0.35

AICc SYM+G 0.25 0.25 0.25 0.25 0.00 0.00 1.533 4.121 2.091 0.754 8.014 1.000 N/A 0.35

DT TrNef+G 0.25 0.25 0.25 0.25 0.00 0.00 1.000 3.176 1.000 1.000 6.165 1.000 N/A 0.35

Program is done.
