# Supplementary material for: Underestimated diversity in high elevations of a global biodiversity hotspot: two new endemic species of Aethionema (Brassicaceae) from the alpine zone of Iran
Source: Front Plant Sci. 2023 May 26;14:1182073. doi: 10.3389/fpls.2023.1182073 (PMC10250747; doi:10.3389/fpls.2023.1182073)
Supplement: Supplementary file 2 [file DataSheet_2.zip › Date Sheet 2/trnLF/Aethionema_ITS_ML_IQ-TREE_output.docx]

IQ-TREE 1.6.12 built Aug 15 2019

Input file name: trnL_Jalil_29_12_2022.fasta

Type of analysis: ModelFinder + tree reconstruction + ultrafast bootstrap (1000 replicates)

Random seed number: 515177

REFERENCES

----------

To cite ModelFinder please use:

Subha Kalyaanamoorthy, Bui Quang Minh, Thomas KF Wong, Arndt von Haeseler,

and Lars S Jermiin (2017) ModelFinder: Fast model selection for

accurate phylogenetic estimates. Nature Methods, 14:587–589.

https://doi.org/10.1038/nmeth.4285

To cite IQ-TREE please use:

Lam-Tung Nguyen, Heiko A. Schmidt, Arndt von Haeseler, and Bui Quang Minh

(2015) IQ-TREE: A fast and effective stochastic algorithm for estimating

maximum likelihood phylogenies. Mol Biol Evol, 32:268-274.

https://doi.org/10.1093/molbev/msu300

Since you used ultrafast bootstrap (UFBoot) please also cite:

Diep Thi Hoang, Olga Chernomor, Arndt von Haeseler, Bui Quang Minh,

and Le Sy Vinh (2017) UFBoot2: Improving the ultrafast bootstrap

approximation. Mol Biol Evol, in press.

https://doi.org/10.1093/molbev/msx281

SEQUENCE ALIGNMENT

------------------

Input data: 48 sequences with 699 nucleotide sites

Number of constant sites: 581 (= 83.1187% of all sites)

Number of invariant (constant or ambiguous constant) sites: 581 (= 83.1187% of all sites)

Number of parsimony informative sites: 62

Number of distinct site patterns: 203

ModelFinder

-----------

Best-fit model according to BIC: K3Pu+F+G4

List of models sorted by BIC scores:

Model LogL AIC w-AIC AICc w-AICc BIC w-BIC

K3Pu+F+G4 -1958.5388 4115.0777 + 0.3303 4148.1328 + 0.4449 4565.4931 + 0.7506

K3Pu+F+I -1960.0178 4118.0356 + 0.0753 4151.0907 + 0.1014 4568.4510 + 0.1710

K3Pu+F+I+G4 -1958.2837 4116.5674 + 0.1568 4150.3467 + 0.1471 4571.5325 - 0.0366

TIM+F+G4 -1958.4762 4116.9524 + 0.1294 4150.7317 + 0.1213 4571.9175 - 0.0302

TIM+F+I -1959.9498 4119.8996 - 0.0296 4153.6788 - 0.0278 4574.8647 - 0.0069

TVM+F+G4 -1957.8208 4117.6417 + 0.0916 4152.1542 + 0.0596 4577.1564 - 0.0022

TIM+F+I+G4 -1958.2238 4118.4476 + 0.0612 4152.9601 - 0.0398 4577.9623 - 0.0015

TVM+F+I -1959.3073 4120.6147 - 0.0207 4155.1272 - 0.0135 4580.1294 - 0.0005

K3Pu+F -1970.2995 4136.5990 - 0.0000 4168.9390 - 0.0000 4582.4648 - 0.0002

TVM+F+I+G4 -1957.5415 4119.0830 - 0.0446 4154.3380 - 0.0200 4583.1474 - 0.0001

GTR+F+G4 -1957.7739 4119.5478 - 0.0353 4154.8028 - 0.0158 4583.6122 - 0.0001

GTR+F+I -1959.2523 4122.5046 - 0.0081 4157.7597 - 0.0036 4586.5690 - 0.0000

TIM+F -1970.0946 4138.1892 - 0.0000 4171.2443 - 0.0000 4588.6046 - 0.0000

GTR+F+I+G4 -1957.5005 4121.0011 - 0.0171 4157.0078 - 0.0053 4589.6151 - 0.0000

TVM+F -1969.8051 4139.6101 - 0.0000 4173.3894 - 0.0000 4594.5752 - 0.0000

GTR+F -1969.6011 4141.2023 - 0.0000 4175.7149 - 0.0000 4600.7170 - 0.0000

F81+F+G4 -1983.1677 4160.3353 - 0.0000 4191.9693 - 0.0000 4601.6514 - 0.0000

F81+F+I -1984.4759 4162.9517 - 0.0000 4194.5857 - 0.0000 4604.2679 - 0.0000

TPM3+F+G4 -1979.3290 4156.6580 - 0.0000 4189.7131 - 0.0000 4607.0735 - 0.0000

TPM3u+F+G4 -1979.3290 4156.6581 - 0.0000 4189.7132 - 0.0000 4607.0735 - 0.0000

F81+F+I+G4 -1982.8364 4161.6729 - 0.0000 4194.0129 - 0.0000 4607.5386 - 0.0000

HKY+F+G4 -1983.1645 4162.3290 - 0.0000 4194.6690 - 0.0000 4608.1947 - 0.0000

TPM3u+F+I -1980.7256 4159.4512 - 0.0000 4192.5062 - 0.0000 4609.8666 - 0.0000

TPM3+F+I -1980.7256 4159.4512 - 0.0000 4192.5063 - 0.0000 4609.8666 - 0.0000

HKY+F+I -1984.4737 4164.9473 - 0.0000 4197.2873 - 0.0000 4610.8131 - 0.0000

TPM2+F+G4 -1981.9345 4161.8689 - 0.0000 4194.9240 - 0.0000 4612.2844 - 0.0000

TPM2u+F+G4 -1981.9346 4161.8692 - 0.0000 4194.9243 - 0.0000 4612.2847 - 0.0000

TPM3u+F+I+G4 -1979.0248 4158.0497 - 0.0000 4191.8290 - 0.0000 4613.0148 - 0.0000

TPM3+F+I+G4 -1979.0254 4158.0509 - 0.0000 4191.8301 - 0.0000 4613.0159 - 0.0000

TIM3+F+G4 -1979.2906 4158.5811 - 0.0000 4192.3604 - 0.0000 4613.5462 - 0.0000

HKY+F+I+G4 -1982.8333 4163.6665 - 0.0000 4196.7216 - 0.0000 4614.0820 - 0.0000

TN+F+G4 -1983.1067 4164.2135 - 0.0000 4197.2686 - 0.0000 4614.6289 - 0.0000

TPM2+F+I -1983.2987 4164.5974 - 0.0000 4197.6525 - 0.0000 4615.0129 - 0.0000

TPM2u+F+I -1983.2989 4164.5977 - 0.0000 4197.6528 - 0.0000 4615.0132 - 0.0000

TIM3+F+I -1980.6767 4161.3534 - 0.0000 4195.1327 - 0.0000 4616.3185 - 0.0000

TN+F+I -1984.4100 4166.8200 - 0.0000 4199.8751 - 0.0000 4617.2355 - 0.0000

TPM2u+F+I+G4 -1981.6418 4163.2835 - 0.0000 4197.0628 - 0.0000 4618.2486 - 0.0000

TPM2+F+I+G4 -1981.6423 4163.2847 - 0.0000 4197.0640 - 0.0000 4618.2498 - 0.0000

TIM2+F+G4 -1981.8650 4163.7301 - 0.0000 4197.5093 - 0.0000 4618.6952 - 0.0000

TIM3+F+I+G4 -1978.9889 4159.9778 - 0.0000 4194.4904 - 0.0000 4619.4925 - 0.0000

F81+F -1995.5449 4183.0898 - 0.0000 4214.0267 - 0.0000 4619.8563 - 0.0000

TN+F+I+G4 -1982.7832 4165.5664 - 0.0000 4199.3457 - 0.0000 4620.5315 - 0.0000

TIM2+F+I -1983.2260 4166.4521 - 0.0000 4200.2314 - 0.0000 4621.4172 - 0.0000

TIM2+F+I+G4 -1981.5765 4165.1531 - 0.0000 4199.6657 - 0.0000 4624.6678 - 0.0000

TPM3+F -1991.6447 4179.2893 - 0.0000 4211.6293 - 0.0000 4625.1551 - 0.0000

TPM3u+F -1991.6447 4179.2894 - 0.0000 4211.6294 - 0.0000 4625.1552 - 0.0000

HKY+F -1995.5300 4185.0601 - 0.0000 4216.6940 - 0.0000 4626.3762 - 0.0000

TPM2+F -1993.8016 4183.6033 - 0.0000 4215.9433 - 0.0000 4629.4691 - 0.0000

TPM2u+F -1993.8019 4183.6038 - 0.0000 4215.9438 - 0.0000 4629.4696 - 0.0000

TIM3+F -1991.4405 4180.8811 - 0.0000 4213.9362 - 0.0000 4631.2965 - 0.0000

TN+F -1995.3257 4186.6513 - 0.0000 4218.9913 - 0.0000 4632.5171 - 0.0000

TIM2+F -1993.5973 4185.1947 - 0.0000 4218.2498 - 0.0000 4635.6101 - 0.0000

K3P+G4 -2013.5125 4219.0250 - 0.0000 4249.9618 - 0.0000 4655.7914 - 0.0000

K3P+I -2014.7719 4221.5437 - 0.0000 4252.4806 - 0.0000 4658.3102 - 0.0000

K3P+I+G4 -2013.0704 4220.1408 - 0.0000 4251.7747 - 0.0000 4661.4569 - 0.0000

TIMe+G4 -2013.4633 4220.9266 - 0.0000 4252.5605 - 0.0000 4662.2427 - 0.0000

TIMe+I -2014.7182 4223.4363 - 0.0000 4255.0703 - 0.0000 4664.7524 - 0.0000

TVMe+G4 -2012.3951 4220.7901 - 0.0000 4253.1301 - 0.0000 4666.6559 - 0.0000

TIMe+I+G4 -2013.0276 4222.0552 - 0.0000 4254.3952 - 0.0000 4667.9210 - 0.0000

TVMe+I -2013.6274 4223.2547 - 0.0000 4255.5947 - 0.0000 4669.1205 - 0.0000

TVMe+I+G4 -2011.9151 4221.8302 - 0.0000 4254.8853 - 0.0000 4672.2456 - 0.0000

SYM+G4 -2012.3443 4222.6886 - 0.0000 4255.7436 - 0.0000 4673.1040 - 0.0000

SYM+I -2013.5719 4225.1437 - 0.0000 4258.1988 - 0.0000 4675.5592 - 0.0000

K3P -2027.2186 4244.4373 - 0.0000 4274.6860 - 0.0000 4676.6541 - 0.0000

SYM+I+G4 -2011.8723 4223.7445 - 0.0000 4257.5238 - 0.0000 4678.7096 - 0.0000

JC+G4 -2031.8799 4251.7598 - 0.0000 4281.3293 - 0.0000 4679.4270 - 0.0000

JC+I -2033.1132 4254.2264 - 0.0000 4283.7960 - 0.0000 4681.8936 - 0.0000

TIMe -2027.0030 4246.0060 - 0.0000 4276.9429 - 0.0000 4682.7725 - 0.0000

JC+I+G4 -2031.4538 4252.9077 - 0.0000 4283.1564 - 0.0000 4685.1245 - 0.0000

K2P+G4 -2031.7358 4253.4716 - 0.0000 4283.7204 - 0.0000 4685.6884 - 0.0000

K2P+I -2032.9686 4255.9372 - 0.0000 4286.1860 - 0.0000 4688.1540 - 0.0000

TVMe -2026.5776 4247.1553 - 0.0000 4278.7892 - 0.0000 4688.4714 - 0.0000

K2P+I+G4 -2031.3090 4254.6179 - 0.0000 4285.5548 - 0.0000 4691.3844 - 0.0000

TNe+G4 -2031.6849 4255.3699 - 0.0000 4286.3068 - 0.0000 4692.1363 - 0.0000

SYM -2026.3625 4248.7250 - 0.0000 4281.0650 - 0.0000 4694.5908 - 0.0000

TNe+I -2032.9134 4257.8268 - 0.0000 4288.7636 - 0.0000 4694.5932 - 0.0000

TNe+I+G4 -2031.2654 4256.5308 - 0.0000 4288.1647 - 0.0000 4697.8469 - 0.0000

TIM3e+G4 -2031.6070 4257.2139 - 0.0000 4288.8479 - 0.0000 4698.5300 - 0.0000

TIM2e+G4 -2031.6827 4257.3654 - 0.0000 4288.9994 - 0.0000 4698.6816 - 0.0000

JC -2045.4207 4276.8414 - 0.0000 4305.7406 - 0.0000 4699.9589 - 0.0000

TIM3e+I -2032.8420 4259.6840 - 0.0000 4291.3180 - 0.0000 4701.0001 - 0.0000

TIM2e+I -2032.9094 4259.8187 - 0.0000 4291.4527 - 0.0000 4701.1349 - 0.0000

TIM3e+I+G4 -2031.1966 4258.3933 - 0.0000 4290.7333 - 0.0000 4704.2591 - 0.0000

TIM2e+I+G4 -2031.2588 4258.5176 - 0.0000 4290.8576 - 0.0000 4704.3834 - 0.0000

K2P -2045.2812 4278.5625 - 0.0000 4308.1320 - 0.0000 4706.2297 - 0.0000

TNe -2045.0649 4280.1298 - 0.0000 4310.3786 - 0.0000 4712.3467 - 0.0000

TIM3e -2044.7689 4281.5378 - 0.0000 4312.4747 - 0.0000 4718.3043 - 0.0000

TIM2e -2045.0099 4282.0199 - 0.0000 4312.9568 - 0.0000 4718.7864 - 0.0000

AIC, w-AIC : Akaike information criterion scores and weights.

AICc, w-AICc : Corrected AIC scores and weights.

BIC, w-BIC : Bayesian information criterion scores and weights.

Plus signs denote the 95% confidence sets.

Minus signs denote significant exclusion.

SUBSTITUTION PROCESS

--------------------

Model of substitution: K3Pu+F+G4

Rate parameter R:

A-C: 1.0000

A-G: 0.5895

A-T: 0.2149

C-G: 0.2149

C-T: 0.5895

G-T: 1.0000

State frequencies: (empirical counts from alignment)

pi(A) = 0.3091

pi(C) = 0.1589

pi(G) = 0.1727

pi(T) = 0.3593

Rate matrix Q:

A -0.82 0.3856 0.2471 0.1873

C 0.7499 -1.354 0.09006 0.5139

G 0.4421 0.08286 -1.397 0.8719

T 0.1611 0.2273 0.4191 -0.8076

Model of rate heterogeneity: Gamma with 4 categories

Gamma shape alpha: 0.5115

Category Relative_rate Proportion

1 0.03553 0.25

2 0.2593 0.25

3 0.8284 0.25

4 2.877 0.25

Relative rates are computed as MEAN of the portion of the Gamma distribution falling in the category.

MAXIMUM LIKELIHOOD TREE

-----------------------

Log-likelihood of the tree: -1954.8111 (s.e. 89.5895)

Unconstrained log-likelihood (without tree): -2855.5433

Number of free parameters (#branches + #model parameters): 99

Akaike information criterion (AIC) score: 4107.6223

Corrected Akaike information criterion (AICc) score: 4140.6773

Bayesian information criterion (BIC) score: 4558.0377

Total tree length (sum of branch lengths): 0.2432

Sum of internal branch lengths: 0.1041 (42.8220% of tree length)

WARNING: 18 near-zero internal branches (<0.0014) should be treated with caution

Such branches are denoted by '**' in the figure below

NOTE: Tree is UNROOTED although outgroup taxon '_Shirkuh_J2_trnL' is drawn at root

Numbers in parentheses are SH-aLRT support (%) / ultrafast bootstrap support (%)

+**_Shirkuh_J2_trnL

|

| +**Umbellatum_Archibold_J1

| +----| (95.6/99)

| | +--HM1454_Oshrurankuh_S1857

| +--| (78.6/92)

| | | +--HM478_spec_nova_S645

| | | +--| (77.9/67)

| | | | | +**HM482_spec_nova_S648

| | | | | +**| (0/72)

| | | | | | +**HM479_spec._nova_S646__reversed_

| | | | +**| (0/76)

| | | | +**HM480_spec_nova_S647

| | +--| (77.1/89)

| | | +-----HM326_erinaceum_S572

| | | +**| (0/72)

| | | | +----Ae_spicatum__reversed_

| | | +------| (96.9/100)

| | | | | +--HM100_grandiflorum_S574__reversed_

| | | | | +**| (0/89)

| | | | | | +--Ae_huber_morathii__reversed_

| | | | +--| (0/93)

| | | | +--Ae_capitatum__reversed_

| | | +---| (90.8/94)

| | | | | +**HM86_transhyrcanum_S579__reversed_

| | | | +--| (88.3/96)

| | | | +--HM104_membranaceum_S573__reversed_

| | | +--| (74.4/76)

| | | | | +**DQ180216_elongatum__reversed_

| | | | | +**| (0/80)

| | | | | | +**Ae_glaucinum__reversed_

| | | | | +------| (84/97)

| | | | | | +**Ae_demirizii__reversed_

| | | | | +**| (0/61)

| | | | | | | +--Ae_armenum__reversed_

| | | | | | | +**| (0/60)

| | | | | | | | +**Ae_coridifolium__reversed_

| | | | | | | +------| (98.8/100)

| | | | | | | | +--Ae_umbellatum__reversed_

| | | | | | +--| (81.2/93)

| | | | | | +**Ae_diastrophis__reversed_

| | | | | +**| (0/72)

| | | | | | +--Ae_schistosum__reversed_

| | | | | +--| (84.9/83)

| | | | | | +**Ae_alanyae__reversed_

| | | | +---| (92.7/94)

| | | | +**Ae_karamanicum__reversed_

| | | +---| (88.8/88)

| | | | +--Ae_eunomioides__reversed_

| | | +---------| (97.7/100)

| | | | | +---------------------------Ae_lepidioides__reversed_

| | | | +-----------| (98.7/100)

| | | | +---HM95_spinosum_S592

| | | +**| (0/32)

| | | | +----Ae_cordatum__reversed_

| | | +**| (0/11)

| | | | +--Ae_lycium__reversed_

| | | +**| (0/29)

| | | | +---Ae_turcica__reversed_

| | | +**| (0/70)

| | | | +--Ae_thesiifolium__reversed_

| | | +---| (94.7/94)

| | | | | +**Ae_arabicum__reversed_

| | | | | +**| (0/81)

| | | | | | +**Ae_carneum__reversed_

| | | | | +**| (0/69)

| | | | | | | +**Ae_froedinii__reversed_

| | | | | | +--| (93/99)

| | | | | | +**Ae_syriacum__reversed_

| | | | | +--| (88.5/93)

| | | | | | +--Ae_heterocarpum__reversed_

| | | | | +--| (87.8/67)

| | | | | | +--Ae_stenopterum__reversed_

| | | | +--| (75/56)

| | | | +----Ae_dumanii__reversed_

| | +**| (0/53)

| | | +-------Ae_acarii__reversed_

| | | +--| (86.8/97)

| | | | +**Ae_saxatile__reversed_

| | | +--| (76/90)

| | | | | +--Ae_munzurense__reversed_

| | | | | +--| (93.3/96)

| | | | | | | +--Ae_papillosum__reversed_

| | | | | | +**| (0/84)

| | | | | | +--Ae_speciosum__reversed_

| | | | +--| (78.4/92)

| | | | | +------Ae_fimbriatum__reversed_

| | | | +**| (0/76)

| | | | +---Ae_stylosum__reversed_

| | +--| (86.5/75)

| | +**Ae_orbiculatum__reversed_

+**| (0/96)

| +**W_0184833_Aethionema_trnT-trnL-trnF__reversed_

|

+**HM1452_yazd_S1855

Tree in newick format:

(_Shirkuh_J2_trnL:0.0000023974,(((Umbellatum_Archibold_J1:0.0000021213,HM1454_Oshrurankuh_S1857:0.0015973062)95.6/99:0.0064495058,((HM478_spec_nova_S645:0.0015831865,((HM482_spec_nova_S648:0.0000023974,HM479_spec._nova_S646__reversed_:0.0000023974)0/72:0.0000023974,HM480_spec_nova_S647:0.0000023974)0/76:0.0000020077)77.9/67:0.0015872598,((((((((((((HM326_erinaceum_S572:0.0067393284,Ae_spicatum__reversed_:0.0063152050)0/72:0.0000020160,((HM100_grandiflorum_S574__reversed_:0.0031539139,Ae_huber_morathii__reversed_:0.0015760966)0/89:0.0000020584,Ae_capitatum__reversed_:0.0019860766)0/93:0.0016396402)96.9/100:0.0083228749,(HM86_transhyrcanum_S579__reversed_:0.0000023974,HM104_membranaceum_S573__reversed_:0.0015613162)88.3/96:0.0033437385)90.8/94:0.0052586565,((((((DQ180216_elongatum__reversed_:0.0000023974,Ae_glaucinum__reversed_:0.0000023974)0/80:0.0000020249,Ae_demirizii__reversed_:0.0000023974)84/97:0.0083698552,(((Ae_armenum__reversed_:0.0015596129,Ae_coridifolium__reversed_:0.0000023974)0/60:0.0000022629,Ae_umbellatum__reversed_:0.0031285479)98.8/100:0.0078987591,Ae_diastrophis__reversed_:0.0000023903)81.2/93:0.0015662838)0/61:0.0000025281,Ae_schistosum__reversed_:0.0031522853)0/72:0.0000022172,Ae_alanyae__reversed_:0.0000024404)84.9/83:0.0016553479,Ae_karamanicum__reversed_:0.0000023974)92.7/94:0.0050630712)74.4/76:0.0016493583,Ae_eunomioides__reversed_:0.0031926843)88.8/88:0.0049622636,(Ae_lepidioides__reversed_:0.0313919130,HM95_spinosum_S592:0.0051270688)98.7/100:0.0140481693)97.7/100:0.0118052915,Ae_cordatum__reversed_:0.0063447505)0/32:0.0000022544,Ae_lycium__reversed_:0.0015743704)0/11:0.0000023974,Ae_turcica__reversed_:0.0047626739)0/29:0.0000022046,Ae_thesiifolium__reversed_:0.0015747319)0/70:0.0000023224,(((((Ae_arabicum__reversed_:0.0000023974,Ae_carneum__reversed_:0.0000023974)0/81:0.0000022888,(Ae_froedinii__reversed_:0.0000025083,Ae_syriacum__reversed_:0.0000026525)93/99:0.0015790244)0/69:0.0000024776,Ae_heterocarpum__reversed_:0.0031673043)88.5/93:0.0031544738,Ae_stenopterum__reversed_:0.0031733009)87.8/67:0.0031520299,Ae_dumanii__reversed_:0.0064983481)75/56:0.0015683892)94.7/94:0.0047377442,(((Ae_acarii__reversed_:0.0089221207,Ae_saxatile__reversed_:0.0000026400)86.8/97:0.0036437863,((Ae_munzurense__reversed_:0.0031732465,(Ae_papillosum__reversed_:0.0015853248,Ae_speciosum__reversed_:0.0031700144)0/84:0.0000023974)93.3/96:0.0031742044,(Ae_fimbriatum__reversed_:0.0081937335,Ae_stylosum__reversed_:0.0048339044)0/76:0.0000025357)78.4/92:0.0015830770)76/90:0.0015822737,Ae_orbiculatum__reversed_:0.0000020939)86.5/75:0.0015799880)0/53:0.0000020395)77.1/89:0.0023235352)78.6/92:0.0023533921,W_0184833_Aethionema_trnT-trnL-trnF__reversed_:0.0000023974)0/96:0.0000025743,HM1452_yazd_S1855:0.0000023974);

CONSENSUS TREE

--------------

Consensus tree is constructed from 1000bootstrap trees

Log-likelihood of consensus tree: -1954.812070

Robinson-Foulds distance between ML tree and consensus tree: 6

Branches with support >0.000000% are kept (extended consensus)

Branch lengths are optimized by maximum likelihood on original alignment

Numbers in parentheses are bootstrap supports (%)

+--_Shirkuh_J2_trnL

|

| +--Umbellatum_Archibold_J1

| +----| (99)

| | +--HM1454_Oshrurankuh_S1857

| +--| (92)

| | | +--HM478_spec_nova_S645

| | | +--| (67)

| | | | | +--HM482_spec_nova_S648

| | | | | +--| (72)

| | | | | | +--HM479_spec._nova_S646__reversed_

| | | | +--| (76)

| | | | +--HM480_spec_nova_S647

| | +--| (89)

| | | +-----HM326_erinaceum_S572

| | | +--| (72)

| | | | +----Ae_spicatum__reversed_

| | | +------| (100)

| | | | | +--HM100_grandiflorum_S574__reversed_

| | | | | +--| (89)

| | | | | | +--Ae_huber_morathii__reversed_

| | | | +--| (93)

| | | | +--Ae_capitatum__reversed_

| | | +---| (94)

| | | | | +--HM86_transhyrcanum_S579__reversed_

| | | | +--| (96)

| | | | +--HM104_membranaceum_S573__reversed_

| | | +--| (76)

| | | | | +--DQ180216_elongatum__reversed_

| | | | | +--| (80)

| | | | | | +--Ae_glaucinum__reversed_

| | | | | +------| (97)

| | | | | | +--Ae_demirizii__reversed_

| | | | | +--| (61)

| | | | | | | +--Ae_armenum__reversed_

| | | | | | | +--| (60)

| | | | | | | | +--Ae_coridifolium__reversed_

| | | | | | | +------| (100)

| | | | | | | | +--Ae_umbellatum__reversed_

| | | | | | +--| (93)

| | | | | | +--Ae_diastrophis__reversed_

| | | | | +--| (72)

| | | | | | +--Ae_schistosum__reversed_

| | | | | +--| (83)

| | | | | | +--Ae_alanyae__reversed_

| | | | +---| (94)

| | | | +--Ae_karamanicum__reversed_

| | | +---| (88)

| | | | +--Ae_eunomioides__reversed_

| | | +---------| (100)

| | | | | +---------------------------Ae_lepidioides__reversed_

| | | | +-----------| (100)

| | | | +---HM95_spinosum_S592

| | | +--| (33)

| | | | +--Ae_thesiifolium__reversed_

| | | +--| (25)

| | | | +---Ae_turcica__reversed_

| | | +--| (28)

| | | | +--Ae_lycium__reversed_

| | | +--| (70)

| | | | +----Ae_cordatum__reversed_

| | | +---| (94)

| | | | | +--Ae_arabicum__reversed_

| | | | | +--| (81)

| | | | | | +--Ae_carneum__reversed_

| | | | | +--| (69)

| | | | | | | +--Ae_froedinii__reversed_

| | | | | | +--| (99)

| | | | | | +--Ae_syriacum__reversed_

| | | | | +--| (93)

| | | | | | +--Ae_heterocarpum__reversed_

| | | | | +--| (67)

| | | | | | +--Ae_stenopterum__reversed_

| | | | +--| (56)

| | | | +----Ae_dumanii__reversed_

| | +--| (53)

| | | +-------Ae_acarii__reversed_

| | | +--| (97)

| | | | +--Ae_saxatile__reversed_

| | | +--| (90)

| | | | | +--Ae_munzurense__reversed_

| | | | | +--| (96)

| | | | | | | +--Ae_papillosum__reversed_

| | | | | | +--| (84)

| | | | | | +--Ae_speciosum__reversed_

| | | | +--| (92)

| | | | | +------Ae_fimbriatum__reversed_

| | | | +--| (76)

| | | | +---Ae_stylosum__reversed_

| | +--| (75)

| | +--Ae_orbiculatum__reversed_

+--| (96)

| +--W_0184833_Aethionema_trnT-trnL-trnF__reversed_

|

+--HM1452_yazd_S1855

Consensus tree in newick format:

(_Shirkuh_J2_trnL:0.0000023974,(((Umbellatum_Archibold_J1:0.0000023974,HM1454_Oshrurankuh_S1857:0.0015971143)99:0.0064488040,((HM478_spec_nova_S645:0.0015831160,((HM482_spec_nova_S648:0.0000023974,HM479_spec._nova_S646__reversed_:0.0000023974)72:0.0000023974,HM480_spec_nova_S647:0.0000023974)76:0.0000023974)67:0.0015871648,((((((((((((HM326_erinaceum_S572:0.0067396679,Ae_spicatum__reversed_:0.0063155727)72:0.0000023974,((HM100_grandiflorum_S574__reversed_:0.0031528156,Ae_huber_morathii__reversed_:0.0015761474)89:0.0000025816,Ae_capitatum__reversed_:0.0019868303)93:0.0016389072)100:0.0083239110,(HM86_transhyrcanum_S579__reversed_:0.0000023974,HM104_membranaceum_S573__reversed_:0.0015606747)96:0.0033431605)94:0.0052586252,((((((DQ180216_elongatum__reversed_:0.0000023974,Ae_glaucinum__reversed_:0.0000023974)80:0.0000027545,Ae_demirizii__reversed_:0.0000023974)97:0.0083692554,(((Ae_armenum__reversed_:0.0015589299,Ae_coridifolium__reversed_:0.0000023974)60:0.0000023974,Ae_umbellatum__reversed_:0.0031286271)100:0.0078990571,Ae_diastrophis__reversed_:0.0000023974)93:0.0015655802)61:0.0000027545,Ae_schistosum__reversed_:0.0031517075)72:0.0000023165,Ae_alanyae__reversed_:0.0000023974)83:0.0016543106,Ae_karamanicum__reversed_:0.0000023974)94:0.0050630062)76:0.0016495453,Ae_eunomioides__reversed_:0.0031915882)88:0.0049623885,(Ae_lepidioides__reversed_:0.0313913229,HM95_spinosum_S592:0.0051264753)100:0.0140476687)100:0.0118058352,Ae_thesiifolium__reversed_:0.0015744159)33:0.0000023974,Ae_turcica__reversed_:0.0047622652)25:0.0000023974,Ae_lycium__reversed_:0.0015750766)28:0.0000023974,Ae_cordatum__reversed_:0.0063442839)70:0.0000023974,(((((Ae_arabicum__reversed_:0.0000023974,Ae_carneum__reversed_:0.0000023974)81:0.0000023974,(Ae_froedinii__reversed_:0.0000023974,Ae_syriacum__reversed_:0.0000023974)99:0.0015788588)69:0.0000023974,Ae_heterocarpum__reversed_:0.0031669675)93:0.0031541349,Ae_stenopterum__reversed_:0.0031729179)67:0.0031517087,Ae_dumanii__reversed_:0.0064976044)56:0.0015682496)94:0.0047371554,(((Ae_acarii__reversed_:0.0089213005,Ae_saxatile__reversed_:0.0000023974)97:0.0036440611,((Ae_munzurense__reversed_:0.0031733027,(Ae_papillosum__reversed_:0.0015853510,Ae_speciosum__reversed_:0.0031700802)84:0.0000023974)96:0.0031742266,(Ae_fimbriatum__reversed_:0.0081936443,Ae_stylosum__reversed_:0.0048337910)76:0.0000023974)92:0.0015830107)90:0.0015829178,Ae_orbiculatum__reversed_:0.0000023974)75:0.0015798399)53:0.0000023974)89:0.0023238761)92:0.0023526899,W_0184833_Aethionema_trnT-trnL-trnF__reversed_:0.0000023974)96:0.0000023974,HM1452_yazd_S1855:0.0000023974);

TIME STAMP

----------

Date and time: Thu Dec 29 20:18:46 2022

Total CPU time used: 35.12 seconds (0h:0m:35s)

Total wall-clock time used: 35.98193192 seconds (0h:0m:35s)
