# Supplementary material for: Underestimated diversity in high elevations of a global biodiversity hotspot: two new endemic species of Aethionema (Brassicaceae) from the alpine zone of Iran
Source: Front Plant Sci. 2023 May 26;14:1182073. doi: 10.3389/fpls.2023.1182073 (PMC10250747; doi:10.3389/fpls.2023.1182073)
Supplement: Supplementary file 2 [file DataSheet_2.zip › Date Sheet 2/trnLF/Aethionema_trnLF_MrBayes_consensus_trees.docx]

#NEXUS

[ID: 8348894888]

begin taxa;

dimensions ntax=48;

taxlabels

Shirkuh_J2_trnL

Umbellatum_Archibold_J1

W_0184833_Aethionema

HM1452_yazd_S1855

HM1454_Oshrurankuh

HM478_spec_nova_S645

HM482_spec_nova_S648

HM326_erinaceum_S572

HM480_spec_nova_S647

HM479_spec._nova_S646

DQ180216_elongatum

HM86_transhyrcanum_S579

HM100_grandiflorum_S574

Ae_acarii

Ae_alanyae

Ae_karamanicum

Ae_schistosum

Ae_armenum

Ae_coridifolium

Ae_umbellatum

Ae_diastrophis

Ae_demirizii

Ae_glaucinum

Ae_huber_morathii

Ae_spicatum

HM104_membranaceum_S573

Ae_eunomioides

Ae_capitatum

Ae_arabicum

Ae_carneum

Ae_thesiifolium

Ae_orbiculatum

Ae_froedinii

Ae_syriacum

Ae_heterocarpum

Ae_cordatum

Ae_munzurense

Ae_papillosum

Ae_lycium

Ae_turcica

Ae_fimbriatum

Ae_speciosum

Ae_stylosum

Ae_dumanii

Ae_stenopterum

Ae_saxatile

Ae_lepidioides

HM95_spinosum_S592

;

end;

begin trees;

translate

1 Shirkuh_J2_trnL,

2 Umbellatum_Archibold_J1,

3 W_0184833_Aethionema,

4 HM1452_yazd_S1855,

5 HM1454_Oshrurankuh,

6 HM478_spec_nova_S645,

7 HM482_spec_nova_S648,

8 HM326_erinaceum_S572,

9 HM480_spec_nova_S647,

10 HM479_spec._nova_S646,

11 DQ180216_elongatum,

12 HM86_transhyrcanum_S579,

13 HM100_grandiflorum_S574,

14 Ae_acarii,

15 Ae_alanyae,

16 Ae_karamanicum,

17 Ae_schistosum,

18 Ae_armenum,

19 Ae_coridifolium,

20 Ae_umbellatum,

21 Ae_diastrophis,

22 Ae_demirizii,

23 Ae_glaucinum,

24 Ae_huber_morathii,

25 Ae_spicatum,

26 HM104_membranaceum_S573,

27 Ae_eunomioides,

28 Ae_capitatum,

29 Ae_arabicum,

30 Ae_carneum,

31 Ae_thesiifolium,

32 Ae_orbiculatum,

33 Ae_froedinii,

34 Ae_syriacum,

35 Ae_heterocarpum,

36 Ae_cordatum,

37 Ae_munzurense,

38 Ae_papillosum,

39 Ae_lycium,

40 Ae_turcica,

41 Ae_fimbriatum,

42 Ae_speciosum,

43 Ae_stylosum,

44 Ae_dumanii,

45 Ae_stenopterum,

46 Ae_saxatile,

47 Ae_lepidioides,

48 HM95_spinosum_S592

;

tree con_50_majrule = [&U] (1[&prob=1.00000000e+00,prob_stddev=0.00000000e+00,prob_range={1.00000000e+00,1.00000000e+00},prob(percent)="100",prob+-sd="100+-0"]:1.406226e-03[&length_mean=3.69690760e-03,length_median=1.40622600e-03,length_95%HPD={5.62370500e-08,1.36950000e-02}],3[&prob=1.00000000e+00,prob_stddev=0.00000000e+00,prob_range={1.00000000e+00,1.00000000e+00},prob(percent)="100",prob+-sd="100+-0"]:1.431808e-03[&length_mean=3.76286567e-03,length_median=1.43180800e-03,length_95%HPD={1.46574300e-08,1.40440200e-02}],4[&prob=1.00000000e+00,prob_stddev=0.00000000e+00,prob_range={1.00000000e+00,1.00000000e+00},prob(percent)="100",prob+-sd="100+-0"]:1.414800e-03[&length_mean=3.69177793e-03,length_median=1.41480000e-03,length_95%HPD={1.31607900e-08,1.36194300e-02}],((2[&prob=1.00000000e+00,prob_stddev=0.00000000e+00,prob_range={1.00000000e+00,1.00000000e+00},prob(percent)="100",prob+-sd="100+-0"]:1.425685e-03[&length_mean=3.72754705e-03,length_median=1.42568500e-03,length_95%HPD={8.12422900e-08,1.36779400e-02}],5[&prob=1.00000000e+00,prob_stddev=0.00000000e+00,prob_range={1.00000000e+00,1.00000000e+00},prob(percent)="100",prob+-sd="100+-0"]:3.383157e-03[&length_mean=7.54921990e-03,length_median=3.38315700e-03,length_95%HPD={2.09766600e-05,2.64642900e-02}])[&prob=1.00000000e+00,prob_stddev=0.00000000e+00,prob_range={1.00000000e+00,1.00000000e+00},prob(percent)="100",prob+-sd="100+-0"]:9.316418e-03[&length_mean=1.93320088e-02,length_median=9.31641800e-03,length_95%HPD={5.17734100e-04,6.60662100e-02}],((6[&prob=1.00000000e+00,prob_stddev=0.00000000e+00,prob_range={1.00000000e+00,1.00000000e+00},prob(percent)="100",prob+-sd="100+-0"]:3.124597e-03[&length_mean=7.03484004e-03,length_median=3.12459700e-03,length_95%HPD={6.73525000e-08,2.46826200e-02}],7[&prob=1.00000000e+00,prob_stddev=0.00000000e+00,prob_range={1.00000000e+00,1.00000000e+00},prob(percent)="100",prob+-sd="100+-0"]:1.419588e-03[&length_mean=3.72255744e-03,length_median=1.41958800e-03,length_95%HPD={1.98930400e-08,1.37090500e-02}],9[&prob=1.00000000e+00,prob_stddev=0.00000000e+00,prob_range={1.00000000e+00,1.00000000e+00},prob(percent)="100",prob+-sd="100+-0"]:1.436743e-03[&length_mean=3.73746220e-03,length_median=1.43674300e-03,length_95%HPD={1.77044900e-08,1.37714700e-02}],10[&prob=1.00000000e+00,prob_stddev=0.00000000e+00,prob_range={1.00000000e+00,1.00000000e+00},prob(percent)="100",prob+-sd="100+-0"]:1.439600e-03[&length_mean=3.78480512e-03,length_median=1.43960000e-03,length_95%HPD={1.91180900e-08,1.38863700e-02}])[&prob=9.24254208e-01,prob_stddev=4.52096084e-03,prob_range={9.20559969e-01,9.30003889e-01},prob(percent)="92",prob+-sd="92+-0"]:3.370712e-03[&length_mean=7.54364659e-03,length_median=3.37071200e-03,length_95%HPD={4.88902800e-06,2.67800700e-02}],((((((8[&prob=1.00000000e+00,prob_stddev=0.00000000e+00,prob_range={1.00000000e+00,1.00000000e+00},prob(percent)="100",prob+-sd="100+-0"]:9.370135e-03[&length_mean=1.92913632e-02,length_median=9.37013500e-03,length_95%HPD={8.16151300e-04,6.57409800e-02}],(13[&prob=1.00000000e+00,prob_stddev=0.00000000e+00,prob_range={1.00000000e+00,1.00000000e+00},prob(percent)="100",prob+-sd="100+-0"]:5.281878e-03[&length_mean=1.13071269e-02,length_median=5.28187800e-03,length_95%HPD={1.16245900e-04,3.93006300e-02}],24[&prob=1.00000000e+00,prob_stddev=0.00000000e+00,prob_range={1.00000000e+00,1.00000000e+00},prob(percent)="100",prob+-sd="100+-0"]:3.367204e-03[&length_mean=7.39499400e-03,length_median=3.36720400e-03,length_95%HPD={1.06849700e-05,2.60337200e-02}])[&prob=7.38389534e-01,prob_stddev=8.86956832e-04,prob_range={7.37181268e-01,7.39181157e-01},prob(percent)="74",prob+-sd="74+-0"]:3.046377e-03[&length_mean=6.94223897e-03,length_median=3.04637700e-03,length_95%HPD={6.50042200e-07,2.48338400e-02}],25[&prob=1.00000000e+00,prob_stddev=0.00000000e+00,prob_range={1.00000000e+00,1.00000000e+00},prob(percent)="100",prob+-sd="100+-0"]:8.665931e-03[&length_mean=1.78059429e-02,length_median=8.66593100e-03,length_95%HPD={6.30725200e-04,6.21121900e-02}],28[&prob=1.00000000e+00,prob_stddev=0.00000000e+00,prob_range={1.00000000e+00,1.00000000e+00},prob(percent)="100",prob+-sd="100+-0"]:3.860387e-03[&length_mean=8.65835745e-03,length_median=3.86038700e-03,length_95%HPD={5.65575200e-06,3.06646200e-02}])[&prob=1.00000000e+00,prob_stddev=0.00000000e+00,prob_range={1.00000000e+00,1.00000000e+00},prob(percent)="100",prob+-sd="100+-0"]:1.115339e-02[&length_mean=2.30448371e-02,length_median=1.11533900e-02,length_95%HPD={1.09761900e-03,7.91161000e-02}],(12[&prob=1.00000000e+00,prob_stddev=0.00000000e+00,prob_range={1.00000000e+00,1.00000000e+00},prob(percent)="100",prob+-sd="100+-0"]:1.489212e-03[&length_mean=3.85545481e-03,length_median=1.48921200e-03,length_95%HPD={1.21530200e-08,1.42263400e-02}],26[&prob=1.00000000e+00,prob_stddev=0.00000000e+00,prob_range={1.00000000e+00,1.00000000e+00},prob(percent)="100",prob+-sd="100+-0"]:3.351768e-03[&length_mean=7.43328572e-03,length_median=3.35176800e-03,length_95%HPD={9.67274400e-06,2.64637100e-02}])[&prob=9.99319482e-01,prob_stddev=2.65937200e-04,prob_range={9.98944503e-01,9.99555580e-01},prob(percent)="100",prob+-sd="100+-0"]:5.510442e-03[&length_mean=1.17151521e-02,length_median=5.51044200e-03,length_95%HPD={1.22749400e-04,4.06807900e-02}])[&prob=9.99847231e-01,prob_stddev=5.31874400e-05,prob_range={9.99777790e-01,9.99888895e-01},prob(percent)="100",prob+-sd="100+-0"]:7.788339e-03[&length_mean=1.63954630e-02,length_median=7.78833900e-03,length_95%HPD={3.83666900e-04,5.62545800e-02}],(((11[&prob=1.00000000e+00,prob_stddev=0.00000000e+00,prob_range={1.00000000e+00,1.00000000e+00},prob(percent)="100",prob+-sd="100+-0"]:1.794769e-03[&length_mean=4.70443846e-03,length_median=1.79476900e-03,length_95%HPD={8.94887100e-08,1.70589500e-02}],22[&prob=1.00000000e+00,prob_stddev=0.00000000e+00,prob_range={1.00000000e+00,1.00000000e+00},prob(percent)="100",prob+-sd="100+-0"]:1.410600e-03[&length_mean=3.72016074e-03,length_median=1.41060000e-03,length_95%HPD={3.08816300e-08,1.37467700e-02}],23[&prob=1.00000000e+00,prob_stddev=0.00000000e+00,prob_range={1.00000000e+00,1.00000000e+00},prob(percent)="100",prob+-sd="100+-0"]:1.389519e-03[&length_mean=3.65332970e-03,length_median=1.38951900e-03,length_95%HPD={3.32133700e-08,1.34736500e-02}])[&prob=9.47447364e-01,prob_stddev=1.55811225e-03,prob_range={9.46003000e-01,9.49447253e-01},prob(percent)="95",prob+-sd="95+-0"]:7.598724e-03[&length_mean=1.61818907e-02,length_median=7.59872400e-03,length_95%HPD={5.19129600e-07,5.65920400e-02}],15[&prob=1.00000000e+00,prob_stddev=0.00000000e+00,prob_range={1.00000000e+00,1.00000000e+00},prob(percent)="100",prob+-sd="100+-0"]:1.419803e-03[&length_mean=3.74292303e-03,length_median=1.41980300e-03,length_95%HPD={3.23644700e-08,1.37498200e-02}],17[&prob=1.00000000e+00,prob_stddev=0.00000000e+00,prob_range={1.00000000e+00,1.00000000e+00},prob(percent)="100",prob+-sd="100+-0"]:5.139849e-03[&length_mean=1.09921261e-02,length_median=5.13984900e-03,length_95%HPD={5.32823900e-05,3.79528600e-02}],((18[&prob=1.00000000e+00,prob_stddev=0.00000000e+00,prob_range={1.00000000e+00,1.00000000e+00},prob(percent)="100",prob+-sd="100+-0"]:3.347468e-03[&length_mean=7.30792931e-03,length_median=3.34746800e-03,length_95%HPD={8.96544500e-06,2.58087900e-02}],19[&prob=1.00000000e+00,prob_stddev=0.00000000e+00,prob_range={1.00000000e+00,1.00000000e+00},prob(percent)="100",prob+-sd="100+-0"]:1.423283e-03[&length_mean=3.71848720e-03,length_median=1.42328300e-03,length_95%HPD={1.84566100e-08,1.36006600e-02}],20[&prob=1.00000000e+00,prob_stddev=0.00000000e+00,prob_range={1.00000000e+00,1.00000000e+00},prob(percent)="100",prob+-sd="100+-0"]:5.240103e-03[&length_mean=1.11092186e-02,length_median=5.24010300e-03,length_95%HPD={1.20435700e-04,3.84707700e-02}])[&prob=1.00000000e+00,prob_stddev=0.00000000e+00,prob_range={1.00000000e+00,1.00000000e+00},prob(percent)="100",prob+-sd="100+-0"]:1.099383e-02[&length_mean=2.24201670e-02,length_median=1.09938300e-02,length_95%HPD={8.45644700e-04,7.51858900e-02}],21[&prob=1.00000000e+00,prob_stddev=0.00000000e+00,prob_range={1.00000000e+00,1.00000000e+00},prob(percent)="100",prob+-sd="100+-0"]:1.467618e-03[&length_mean=3.86086028e-03,length_median=1.46761800e-03,length_95%HPD={1.79168900e-08,1.41777600e-02}])[&prob=9.43294817e-01,prob_stddev=2.31388983e-03,prob_range={9.39947781e-01,9.45169713e-01},prob(percent)="94",prob+-sd="94+-0"]:3.342425e-03[&length_mean=7.37472399e-03,length_median=3.34242500e-03,length_95%HPD={1.11137000e-06,2.60143200e-02}])[&prob=8.70854397e-01,prob_stddev=3.74430099e-03,prob_range={8.65729682e-01,8.74729182e-01},prob(percent)="87",prob+-sd="87+-0"]:3.434193e-03[&length_mean=7.71996627e-03,length_median=3.43419300e-03,length_95%HPD={1.07083700e-06,2.73148700e-02}],16[&prob=1.00000000e+00,prob_stddev=0.00000000e+00,prob_range={1.00000000e+00,1.00000000e+00},prob(percent)="100",prob+-sd="100+-0"]:1.605861e-03[&length_mean=4.12159315e-03,length_median=1.60586100e-03,length_95%HPD={3.88619200e-08,1.52265500e-02}])[&prob=1.00000000e+00,prob_stddev=0.00000000e+00,prob_range={1.00000000e+00,1.00000000e+00},prob(percent)="100",prob+-sd="100+-0"]:7.786113e-03[&length_mean=1.61180021e-02,length_median=7.78611300e-03,length_95%HPD={4.18653200e-04,5.59499700e-02}])[&prob=9.11921560e-01,prob_stddev=2.21491162e-03,prob_range={9.09449475e-01,9.14838065e-01},prob(percent)="91",prob+-sd="91+-0"]:3.437402e-03[&length_mean=7.64372953e-03,length_median=3.43740200e-03,length_95%HPD={8.23674600e-07,2.70328400e-02}],27[&prob=1.00000000e+00,prob_stddev=0.00000000e+00,prob_range={1.00000000e+00,1.00000000e+00},prob(percent)="100",prob+-sd="100+-0"]:5.368940e-03[&length_mean=1.14166024e-02,length_median=5.36894000e-03,length_95%HPD={1.31140900e-04,3.94314700e-02}])[&prob=9.98972279e-01,prob_stddev=1.84246697e-04,prob_range={9.98722293e-01,9.99166713e-01},prob(percent)="100",prob+-sd="100+-0"]:7.384475e-03[&length_mean=1.53917581e-02,length_median=7.38447500e-03,length_95%HPD={1.92496300e-04,5.31859800e-02}],(47[&prob=1.00000000e+00,prob_stddev=0.00000000e+00,prob_range={1.00000000e+00,1.00000000e+00},prob(percent)="100",prob+-sd="100+-0"]:3.615976e-02[&length_mean=7.34949134e-02,length_median=3.61597600e-02,length_95%HPD={7.62929100e-03,2.49569500e-01}],48[&prob=1.00000000e+00,prob_stddev=0.00000000e+00,prob_range={1.00000000e+00,1.00000000e+00},prob(percent)="100",prob+-sd="100+-0"]:7.777725e-03[&length_mean=1.61259150e-02,length_median=7.77772500e-03,length_95%HPD={1.17146000e-04,5.66495700e-02}])[&prob=1.00000000e+00,prob_stddev=0.00000000e+00,prob_range={1.00000000e+00,1.00000000e+00},prob(percent)="100",prob+-sd="100+-0"]:1.781636e-02[&length_mean=3.59825700e-02,length_median=1.78163600e-02,length_95%HPD={2.46610100e-03,1.21953000e-01}])[&prob=1.00000000e+00,prob_stddev=0.00000000e+00,prob_range={1.00000000e+00,1.00000000e+00},prob(percent)="100",prob+-sd="100+-0"]:1.522703e-02[&length_mean=3.09509858e-02,length_median=1.52270300e-02,length_95%HPD={1.95016300e-03,1.06057800e-01}],(((29[&prob=1.00000000e+00,prob_stddev=0.00000000e+00,prob_range={1.00000000e+00,1.00000000e+00},prob(percent)="100",prob+-sd="100+-0"]:1.458699e-03[&length_mean=3.84057669e-03,length_median=1.45869900e-03,length_95%HPD={3.00938000e-08,1.41199200e-02}],30[&prob=1.00000000e+00,prob_stddev=0.00000000e+00,prob_range={1.00000000e+00,1.00000000e+00},prob(percent)="100",prob+-sd="100+-0"]:1.437533e-03[&length_mean=3.79077476e-03,length_median=1.43753300e-03,length_95%HPD={9.76763700e-08,1.40271700e-02}],(33[&prob=1.00000000e+00,prob_stddev=0.00000000e+00,prob_range={1.00000000e+00,1.00000000e+00},prob(percent)="100",prob+-sd="100+-0"]:1.431244e-03[&length_mean=3.71837053e-03,length_median=1.43124400e-03,length_95%HPD={3.94200400e-08,1.38310100e-02}],34[&prob=1.00000000e+00,prob_stddev=0.00000000e+00,prob_range={1.00000000e+00,1.00000000e+00},prob(percent)="100",prob+-sd="100+-0"]:1.432621e-03[&length_mean=3.74958386e-03,length_median=1.43262100e-03,length_95%HPD={7.54301200e-08,1.37832200e-02}])[&prob=9.90014444e-01,prob_stddev=4.89313937e-04,prob_range={9.89333926e-01,9.90444975e-01},prob(percent)="99",prob+-sd="99+-0"]:3.360367e-03[&length_mean=7.45770682e-03,length_median=3.36036700e-03,length_95%HPD={1.78159600e-06,2.63933000e-02}],35[&prob=1.00000000e+00,prob_stddev=0.00000000e+00,prob_range={1.00000000e+00,1.00000000e+00},prob(percent)="100",prob+-sd="100+-0"]:5.286623e-03[&length_mean=1.11937995e-02,length_median=5.28662300e-03,length_95%HPD={1.09599700e-04,3.89164400e-02}])[&prob=9.99152825e-01,prob_stddev=1.83547466e-04,prob_range={9.98888951e-01,9.99277818e-01},prob(percent)="100",prob+-sd="100+-0"]:5.341860e-03[&length_mean=1.12951652e-02,length_median=5.34186000e-03,length_95%HPD={7.71043000e-05,3.95019100e-02}],45[&prob=1.00000000e+00,prob_stddev=0.00000000e+00,prob_range={1.00000000e+00,1.00000000e+00},prob(percent)="100",prob+-sd="100+-0"]:5.263454e-03[&length_mean=1.10714605e-02,length_median=5.26345400e-03,length_95%HPD={3.36124600e-06,3.86714900e-02}])[&prob=9.89917227e-01,prob_stddev=2.29048699e-04,prob_range={9.89667241e-01,9.90222765e-01},prob(percent)="99",prob+-sd="99+-0"]:5.501670e-03[&length_mean=1.16812736e-02,length_median=5.50167000e-03,length_95%HPD={9.14590100e-05,4.05362300e-02}],44[&prob=1.00000000e+00,prob_stddev=0.00000000e+00,prob_range={1.00000000e+00,1.00000000e+00},prob(percent)="100",prob+-sd="100+-0"]:9.436660e-03[&length_mean=1.94126631e-02,length_median=9.43666000e-03,length_95%HPD={7.03260200e-04,6.70892800e-02}])[&prob=8.54730293e-01,prob_stddev=8.12219404e-03,prob_range={8.49008388e-01,8.66729626e-01},prob(percent)="85",prob+-sd="85+-1"]:3.365798e-03[&length_mean=7.47187317e-03,length_median=3.36579800e-03,length_95%HPD={1.64123800e-06,2.63381400e-02}],31[&prob=1.00000000e+00,prob_stddev=0.00000000e+00,prob_range={1.00000000e+00,1.00000000e+00},prob(percent)="100",prob+-sd="100+-0"]:3.366341e-03[&length_mean=7.46515760e-03,length_median=3.36634100e-03,length_95%HPD={6.74825800e-07,2.67646500e-02}],36[&prob=1.00000000e+00,prob_stddev=0.00000000e+00,prob_range={1.00000000e+00,1.00000000e+00},prob(percent)="100",prob+-sd="100+-0"]:9.021536e-03[&length_mean=1.86175609e-02,length_median=9.02153600e-03,length_95%HPD={6.42346000e-04,6.38917200e-02}],39[&prob=1.00000000e+00,prob_stddev=0.00000000e+00,prob_range={1.00000000e+00,1.00000000e+00},prob(percent)="100",prob+-sd="100+-0"]:3.350320e-03[&length_mean=7.41319308e-03,length_median=3.35032000e-03,length_95%HPD={6.28362400e-06,2.58281500e-02}],40[&prob=1.00000000e+00,prob_stddev=0.00000000e+00,prob_range={1.00000000e+00,1.00000000e+00},prob(percent)="100",prob+-sd="100+-0"]:7.122947e-03[&length_mean=1.49451187e-02,length_median=7.12294700e-03,length_95%HPD={3.51251500e-04,5.09839300e-02}])[&prob=9.99111160e-01,prob_stddev=4.58097397e-04,prob_range={9.98722293e-01,9.99611133e-01},prob(percent)="100",prob+-sd="100+-0"]:7.076443e-03[&length_mean=1.47923922e-02,length_median=7.07644300e-03,length_95%HPD={1.05441100e-04,5.10581700e-02}],(((14[&prob=1.00000000e+00,prob_stddev=0.00000000e+00,prob_range={1.00000000e+00,1.00000000e+00},prob(percent)="100",prob+-sd="100+-0"]:1.151388e-02[&length_mean=2.36442178e-02,length_median=1.15138800e-02,length_95%HPD={9.49628800e-04,8.08066500e-02}],46[&prob=1.00000000e+00,prob_stddev=0.00000000e+00,prob_range={1.00000000e+00,1.00000000e+00},prob(percent)="100",prob+-sd="100+-0"]:2.094718e-03[&length_mean=5.25174677e-03,length_median=2.09471800e-03,length_95%HPD={6.17106700e-08,1.91242200e-02}])[&prob=9.85973001e-01,prob_stddev=9.30123760e-04,prob_range={9.84834176e-01,9.87111827e-01},prob(percent)="99",prob+-sd="99+-0"]:5.252091e-03[&length_mean=1.12894541e-02,length_median=5.25209100e-03,length_95%HPD={1.59712600e-06,3.92935800e-02}],((37[&prob=1.00000000e+00,prob_stddev=0.00000000e+00,prob_range={1.00000000e+00,1.00000000e+00},prob(percent)="100",prob+-sd="100+-0"]:5.307025e-03[&length_mean=1.12108258e-02,length_median=5.30702500e-03,length_95%HPD={1.18534500e-04,3.82354700e-02}],38[&prob=1.00000000e+00,prob_stddev=0.00000000e+00,prob_range={1.00000000e+00,1.00000000e+00},prob(percent)="100",prob+-sd="100+-0"]:3.345443e-03[&length_mean=7.46046718e-03,length_median=3.34544300e-03,length_95%HPD={5.76933800e-06,2.64475200e-02}],42[&prob=1.00000000e+00,prob_stddev=0.00000000e+00,prob_range={1.00000000e+00,1.00000000e+00},prob(percent)="100",prob+-sd="100+-0"]:5.311878e-03[&length_mean=1.11826786e-02,length_median=5.31187800e-03,length_95%HPD={1.20958700e-04,3.91522200e-02}])[&prob=9.99847231e-01,prob_stddev=9.48739030e-05,prob_range={9.99722238e-01,9.99944448e-01},prob(percent)="100",prob+-sd="100+-0"]:5.306799e-03[&length_mean=1.12430207e-02,length_median=5.30679900e-03,length_95%HPD={7.01238700e-05,3.90543500e-02}],41[&prob=1.00000000e+00,prob_stddev=0.00000000e+00,prob_range={1.00000000e+00,1.00000000e+00},prob(percent)="100",prob+-sd="100+-0"]:1.112648e-02[&length_mean=2.26985955e-02,length_median=1.11264800e-02,length_95%HPD={1.23990700e-03,7.84400800e-02}],43[&prob=1.00000000e+00,prob_stddev=0.00000000e+00,prob_range={1.00000000e+00,1.00000000e+00},prob(percent)="100",prob+-sd="100+-0"]:7.288259e-03[&length_mean=1.51078352e-02,length_median=7.28825900e-03,length_95%HPD={4.83476600e-04,5.27873500e-02}])[&prob=9.82737070e-01,prob_stddev=9.04186480e-04,prob_range={9.81612133e-01,9.83778679e-01},prob(percent)="98",prob+-sd="98+-0"]:3.374558e-03[&length_mean=7.51771348e-03,length_median=3.37455800e-03,length_95%HPD={2.89933200e-06,2.65652000e-02}])[&prob=9.89875562e-01,prob_stddev=9.70049620e-04,prob_range={9.88500639e-01,9.90611633e-01},prob(percent)="99",prob+-sd="99+-0"]:3.443884e-03[&length_mean=7.59157030e-03,length_median=3.44388400e-03,length_95%HPD={9.33930200e-07,2.67710200e-02}],32[&prob=1.00000000e+00,prob_stddev=0.00000000e+00,prob_range={1.00000000e+00,1.00000000e+00},prob(percent)="100",prob+-sd="100+-0"]:1.488587e-03[&length_mean=3.85966375e-03,length_median=1.48858700e-03,length_95%HPD={3.74604100e-08,1.43778200e-02}])[&prob=8.86881284e-01,prob_stddev=2.42842247e-03,prob_range={8.83784234e-01,8.89672796e-01},prob(percent)="89",prob+-sd="89+-0"]:3.272893e-03[&length_mean=7.32958896e-03,length_median=3.27289300e-03,length_95%HPD={1.76033500e-07,2.60816700e-02}])[&prob=9.56363535e-01,prob_stddev=2.41061874e-03,prob_range={9.53224821e-01,9.58391200e-01},prob(percent)="96",prob+-sd="96+-0"]:4.212855e-03[&length_mean=9.18688402e-03,length_median=4.21285500e-03,length_95%HPD={1.93550900e-06,3.23795200e-02}])[&prob=9.52266541e-01,prob_stddev=1.85325291e-03,prob_range={9.49669463e-01,9.54058108e-01},prob(percent)="95",prob+-sd="95+-0"]:4.217833e-03[&length_mean=9.26390412e-03,length_median=4.21783300e-03,length_95%HPD={2.04950500e-06,3.28585400e-02}]);

end;
