# Supplementary material for: Underestimated diversity in high elevations of a global biodiversity hotspot: two new endemic species of Aethionema (Brassicaceae) from the alpine zone of Iran
Source: Front Plant Sci. 2023 May 26;14:1182073. doi: 10.3389/fpls.2023.1182073 (PMC10250747; doi:10.3389/fpls.2023.1182073)
Supplement: Supplementary file 2 [file DataSheet_2.zip › Date Sheet 2/trnLF/Aethionema_trnLF_MrBayes_input_NEXUS.docx]

#NEXUS

BEGIN DATA;

DIMENSIONS NTAX=48 NCHAR=699;

FORMAT MISSING=? DATATYPE=DNA interleave=yes gap=- missing=?;

MATRIX

Shirkuh_J2_trnL AGAAACTAAAAAGTAGGAAATTCAAAAAGTTTTGGACCGGTAATTTTTTGGATCTTCGAAAAGAATACTTTCTAAG-----TTTTAAA------------ATTAAAATTGTATTGTTG--AATAGCTAAAAAAAACAAGGTACGGTGTCAAACAGACTTTTTGGGG-AGTACAGTTGGGGATAGAGGGACTTGAACCCTCACGATTTTAAAAGTCAACGGATTTTCATCTTACTATAAATTTCATTGG-TGTCAGTATTGACATGTAGAATGGGACTCTATCTTTATTCTCGTGTTATTAA-----------TAAGTTCCACCAAAGATCTATCAGACTATGAAGTGAATCATTTGA-TCAATGAATATTCGATTTTGTCTTCAACTTCGAATTGATTCACAACATTTCTATTTCTTATTAAATTTAAATAGAAAAAAAAAAAAGAAATAGAGATTCAGGTCGTCATTTTTGAGATCATTTTTTGTTACCTATCTTT-----ATGCAATATAGGTTTTTCTCCTTCATCC----TTTTTTATTTGAAGTTTCGATCGAAGGATTCCTTTATCAACACAA-GGTAGT-GAACTCCATTTGTTAGAACAGCTTCCATTGAGTCTCTGCACCTATCCCTTTTTTCTCGTTTTCTAAA-CTCGGGTTTGTTCGCGTAAACCGAGATTTGGCTCAGGATTGCCC

Umbellatum_Archibold_J1 AGAAACTAAAAAGTAGGAAATTCAAAAAGTTTTGGACCGGTAATTTTTTGGATCTTCGAAAAGAATACTTTCTAAG-----TTTTAAA------------ATTAAACTTGTATTGTTG--AATAGCTAAAAAAAACAAGGTAAGATGTCAAACAGACTTTTTGGGG-AGTACAGTTGGGGATAGAGGGACTTGAACCCTCACGATTTTAAAAGTCAACGGATTTTCATCTTACTATAAATTTCATTGG-TGTCAGTATTGACATGTAGAATGGGACTCTATCTTTATTCTCGTGTTATTAT------ATTAATAAGTTCCACCAAAGATCTATCAGACTATGAAGTGAATCATTTGA-TCAATGAATATTCGATTTTGTCTTCAACTTCAAATTGATTCACAACATTTCTATTTC--------TT-AAATAGA-AAAAAAAAAAGAAATAGAGATTCAGGTCGTCATTTTTGAGATCATTTTTTGTTACCTATCTTT-----ATGCAATATAGGTTTTTCTCCTTCATCC----TTTTTTATTTGAAGTTTCGATCGAAGGATTCCTTTATCAACACAA-GGTAGT-GAACTCCATTTGTTAGAACAGCTTCCATTGAGTCTCTGCACCTATCCCTTTTTTCTCGTTTTCTAAA----------GTTCGCGTAAACCGAGATTTGGCTCAGGATTGCCC

W_0184833_Aethionema AGAAACTAAAAAGTAGGAAATTCAAAAAGTTTTGGACCGGTAATTTTTTGGATCTTCGAAAAGAATACTTTCTAAG-----TTTTAAA------------ATTAAAATTGTATTGTTG--AATAGCTAAAAAAAACAAGGTACGGTGTCAAACAGACTTTTTGGGG-AGTACAGTTGGGGATAGAGGGACTTGAACCCTCACGATTTTAAAAGTCAACGGATTTTCATCTTACTATAAATTTCATTGG-TGTCAGTATTGACATGTAGAATGGGACTCTATCTTTATTCTCGTGTTATTAA-----------TAAGTTCCACCAAAGATCTATCAGACTATGAAGTGAATCATTTGA-TCAATGAATATTCGATTTTGTCTTCAACTTCGAATTGATTCACAACATTTCTATTTC--------TT-AAATAG---AAAAAAAAAGAAATAGAGATTCAGGTCGTCATTTTTGAGATCATTTTTTGTTACCTATCTTT-----ATGCAATATAGGTTTTTCTCCTTCATCC----TTTTTTATTTGAAGTTTCGATCGAAGGATTCCTTTATCAACACAA-GGTAGT-GAACTCCATTTGTTAGAACAGCTTCCATTGAGTCTCTGCACCTATCCCTTTTTTCTCGTTTTCTAAA-CTCGGGTTTGTTCGCGTAAACCGAGATTTGGCTCAGGATTGCCC

HM1452_yazd_S1855 AGAAACTAAAAAGTAGGAAATTCAAAAAGTTTTGGACCGGTAATTTTTTGGATCTTCGAAAAGAATACTTTCTAAG-----TTTTAAA------------ATTAAAATTGTATTGTTG--AATAGCTAAAAAAAACAAGGTACGGTGTCAAACAGACTTTTTGGGG-AGTACAGTTGGGGATAGAGGGACTTGAACCCTCACGATTTTAAAAGTCAACGGATTTTCATCTTACTATAAATTTCATTGG-TGTCAGTATTGACATGTAGAATGGGACTCTATCTTTATTCTCGTGTTATTAA-----------TAAGTTCCACCAAAGATCTATCAGACTATGAAGTGAATCATTTGA-TCAATGAATATTCGATTTTGTCTTCAACTTCGAATTGATTCACAACATTTCTATTTCTTATTAAATTTAAATAGAAAAAAAAAAAAGAAATAGAGATTCAGGTCGTCATTTTTGAGATCATTTTTTGTTACCTATCTTT-----ATGCAATATAGGTTTTTCTCCTTCATCC----TTTTTTATTTGAAGTTTCGATCGAAGGATTCCTTTATCAACACAA-GGTAGT-GAACTCCATTTGTTAGAACAGCTTCCATTGAGTCTCTGCACCTATCCCTTTTTTCTCGTTTTCTAAA-CTCGGGTTTGTTCGCGTAAACCGAGATTTGGCTCAGGATTGCCC

HM1454_Oshrurankuh AGAAACTAAAAAGTAGGAAATTCAAAAAGTTTTGGACCGGTAATTTTTTGGATCTTCGAAAAGAATACTTTCTAAG-----TTTTAAA------------ATTAAACTTGTATTGTTG--AATAGCTAAAAAAAACAAGGTAAGATGTCAAACAGACTTTTTGGGG-AGTACAGTTGGGGATAGAGGGACTTGAACCCTCACGATTTTAAAAGTCAACGGATTTTCATCTTACTATAAATTTCATTGG-TGTCAGTATTGACATGTAGAATGGGACTCTATCTTTATTCTCGTGTTATTAT------ATTAATAAGTTCCACCAAAGATCTATCAGACTATGAAGTGAATCATTTGA-TCAATGAATATTCGATTTTGTCTTCAACTTCAAATTGATTCACAACATTTCTATTTC--------TT-AAATAGA-AAAAAAAATAGAAATAGAGATTCAGGTCGTCATTTTTGAGATCATTTTTTGTTACCTATCTTT-----ATGCAATATAGGTTTTTCTCCTTCATCC----TTTTTTATTTGAAGTTTCGATCGAAGGATTCCTTTATCAACACAA-GGTAGT-GAACTCCATTTGTTAGAACAGCTTCCATTGAGTCTCTGCACCTATCCCTTTTTTCTCGTTTTCTAAA----------GTTCGCGTAAACCGAGATTTGGCTCAGGATTGCCC

HM478_spec_nova_S645 AGAAACTAAAAAGTAGGAAATTAAAAAAGTTTTGGACCGGTAATTTTTTGGATCTTCGAAAAGAATACTTTCTAAG-----TTTTAAA------------ATTAAAATTGTATTGTTG--AATAGCTAAAAAAAACAAGGTAAGGTGTCAAACAGACTTTTTGGGG-AGTACAGTTGGGGATAGAGGGACTTGAACCCTCACGATTTTAAAAGTCAACGGATTTTCATCTTACTATAAATTTCATTGG-TGTCAGTATTGACATGTAGAATGGGACTCTATCTTTATTCTCGTGTTATTAA-----------TAAGTTCCACCAAAGATCTATCAGACTATGAAGTGAATCATTTGA-TCAATCAATATTCGATTTTGTCTTCAACTTCGAATTGATTCACAACATTTCTATTTC--------TT-AAATAG---AAAAAAAAAGAAATAGAGATTCAGGTCGTCATTTTTGAGATCATTTTGTGTTACCTATCTTT-----ATGCAATATAGGTTTTTCTCCTTCATCC----TTTTTTATTTGAAGTTTCGATCGAAGGATTCCTTTATCAACACAA-GGTAGT-GAACTCCATTTGTTAGAACAGCTTCCATTGAGTCTCTGCACCTATCCCTTTTTTCTCGTTTTCTAAA-CTCGGGTTTGTTCGCGTAAACCGAGATTTGGCTCAGGATTGCCC

HM482_spec_nova_S648 AGAAACTAAAAAGTAGGAAATTCAAAAAGTTTTGGACCGGTAATTTTTTGGATCTTCGAAAAGAATACTTTCTAAG-----TTTTAAA------------ATTAAAATTGTATTGTTG--AATAGCTAAAAAAAACAAGGTAAGGTGTCAAACAGACTTTTTGGGG-AGTACAGTTGGGGATAGAGGGACTTGAACCCTCACGATTTTAAAAGTCAACGGATTTTCATCTTACTATAAATTTCATTGG-TGTCAGTATTGACATGTAGAATGGGACTCTATCTTTATTCTCGTGTTATTAA-----------TAAGTTCCACCAAAGATCTATCAGACTATGAAGTGAATCATTTGA-TCAATCAATATTCGATTTTGTCTTCAACTTCGAATTGATTCACAACATTTCTATTTC--------TT-AAATAG-AAAAAAAAAAAGAAATAGAGATTCAGGTCGTCATTTTTGAGATCATTTTGTGTTACCTATCTTT-----ATGCAATATAGGTTTTTCTCCTTCATCC----TTTTTTATTTGAAGTTTCGATCGAAGGATTCCTTTATCAACACAA-GGTAGT-GAACTCCATTTGTTAGAACAGCTTCCATTGAGTCTCTGCACCTATCCCTTTTTTCTCGTTTTCTAAA-CTCGGGTTTGTTCGCGTAAACCGAGATTTGGCTCAGGATTGCCC

HM326_erinaceum_S572 AGAAACTAAAAAGTAGTAAA----------------------------------TTCAAAAAGAATACTTTCTAAG-----TTTTAAA------------ATGTAAATTGTATTCTTG--AATAACTAAAAAAAACAAGGTAAGGTATCAAACAGACTTTTTGGGGGAGTACGTTTGGGGATAGAGGGACTTGAACCCTCACGCTTTGAAAAGTCAACGGATTTTCATCTTACTATAAATTTCATTGG-TGTCAGTATTGACATGTAGAATGGGACTCTATCTTTATTCTCGTCTTATTAA-----------TAAGTTCCACCAAAGATCTATCAGACTATGAAGTGAATCATTTGA-TCAATGAATATTCGATTTTGTCTTCAACTTCGAATTCATTCACAACATTTTTATTTC--------TTCAAATCG--AAAAAAAAAAGAAATAGAGATTCAGGTCGTCATTTTTGAGATCGTTTTGTGTTACCTATCTTT-----ATGCAATATAGGTTTTTCTCCTTCATCC----TTTTTGATTTGAAGTTTCGATCGAAGGATTCCTTTATCAACACAA-GGTGGTTGAACTCCATTTGTTAGAACAGCTTCCATTGAGTCTCTGCACCTATCCCTTTTTTATCGTTTTCTAAA-CTCGGGTTTGTTCGCGTAAACCGAGATTTGGCTCAGGATTGCCC

HM480_spec_nova_S647 AGAAACTAAAAAGTAGGAAATTCAAAAAGTTTTGGACCGGTAATTTTTTGGATCTTCGAAAAGAATACTTTCTAAG-----TTTTAAA------------ATTAAAATTGTATTGTTG--AATAGCTAAAAAAAACAAGGTAAGGTGTCAAACAGACTTTTTGGGG-AGTACAGTTGGGGATAGAGGGACTTGAACCCTCACGATTTTAAAAGTCAACGGATTTTCATCTTACTATAAATTTCATTGG-TGTCAGTATTGACATGTAGAATGGGACTCTATCTTTATTCTCGTGTTATTAA-----------TAAGTTCCACCAAAGATCTATCAGACTATGAAGTGAATCATTTGA-TCAATCAATATTCGATTTTGTCTTCAACTTCGAATTGATTCACAACATTTCTATTTC--------TT-AAATAG---AAAAAAAAAGAAATAGAGATTCAGGTCGTCATTTTTGAGATCATTTTGTGTTACCTATCTTT-----ATGCAATATAGGTTTTTCTCCTTCATCC----TTTTTTATTTGAAGTTTCGATCGAAGGATTCCTTTATCAACACAA-GGTAGT-GAACTCCATTTGTTAGAACAGCTTCCATTGAGTCTCTGCACCTATCCCTTTTTTCTCGTTTTCTAAA-CTCGGGTTTGTTCGCGTAAACCGAGATTTGGCTCAGGATTGCCC

HM479_spec._nova_S646 AGAAACTAAAAAGTAGGAAATTCAAAAAGTTTTGGACCGGTAATTTTTTGGATCTTCGAAAAGAATACTTTCTAAG-----TTTTAAA------------ATTAAAATTGTATTGTTG--AATAGCTAAAAAAAACAAGGTAAGGTGTCAAACAGACTTTTTGGGG-AGTACAGTTGGGGATAGAGGGACTTGAACCCTCACGATTTTAAAAGTCAACGGATTTTCATCTTACTATAAATTTCATTGG-TGTCAGTATTGACATGTAGAATGGGACTCTATCTTTATTCTCGTGTTATTAA-----------TAAGTTCCACCAAAGATCTATCAGACTATGAAGTGAATCATTTGA-TCAATCAATATTCGATTTTGTCTTCAACTTCGAATTGATTCACAACATTTCTATTTC--------TT-AAATAG-AAAAAAAAAAAGAAATAGAGATTCAGGTCGTCATTTTTGAGATCATTTTGTGTTACCTATCTTT-----ATGCAATATAGGTTTTTCTCCTTCATCC----TTTTTTATTTGAAGTTTCGATCGAAGGATTCCTTTATCAACACAA-GGTAGT-GAACTCCATTTGTTAGAACAGCTTCCATTGAGTCTCTGCACCTATCCCTTTTTTCTCGTTTTCTAAA-CTCGGGTTTGTTCGCGTAAACCGAGATTTGGCTCAGGATTGCCC

DQ180216_elongatum -----------------------------------------------------------------------------------------------------------------------------------------------------------------------------------------------------------------------------TTTTCATCTTACTATAAATTTCATTGGGTGTCAGTATTGACATGTAGAATGGGACTCTATCTTTATTCTCGTCTTATTAA-----------TAAGTTCCACCAAAGATCTATCAGACTATGAAGTGAATCATTTGATTCAATGAATATTCGATTTTGTCTTCAACTTCGAATTGATTCACAACATTTCTATTTC--------TTAAAATAG----AAAAAAAAGAAATAGAGATTCAGGTCGTCATTTTTGAGATCGTTTTGTGTTACCTATCTTTATGCAATGCAATATAGGTTTTTCTCCTTCATCC----TTTTTGAGTTGAAGTTTCGATCGAAGGATTCCTTTATCAACACAA-GGTGGTTGAACTCCATTTGTTAGAACAGCTTCCATTGAGTCTCTGCACCTATCTCTTTTTTATCGTTTTCTAAAACTCGGGTTTGTTCGCGTAAACCGAGATTTGGCTCAGGATTGCCC

HM86_transhyrcanum_S579 AGAAACTAAAAAGTAGTAAATTAAAAAAGTTTTGGACCGGGAATTTATTGGATCTTCAAAAAGAATACTTTCTAAG-----TTTTCAA------------ATGTAAATTGTATTCTTG--AATAACTAAAAAAAACAAGGTAAGGTGTCAAACAGACTTTTTGGGGGAGTACATTTGGGGATAGAGGGACTTGAACCCTCACGATTTTAAAAGTCAACGGATTTTCATCTTACTATAAATTTCATTGG-TGTCAGTATTGACATGTAGAATGGGACTCTATCTTTATTCTCGTCTTATTAA-----------TAAGTTCCACCAAAGATCTATCAGACTATGAAGTGAATCATTTGA-TCAATGAATATTCGATTTTGTCTTCAACTTCGAATTGATTCACAACATTTCTATTTC--------TTAAAATAG-AAAAAAAAAAAGAAATAGAGATTCAGGTCGTCATTTTTGAGATCGTTTTGTGTTACCTATCTTTTCTTTATGCAATATAGGTTTTTTTCCTTCATCCTTTTTTTTTGATTTGAAGTTTCGATCGAAGGATTCCTTTATCAACACAA-GGTGGTTGAACTCCATTTGTTAGAACAGCTTCCATTGAGTCTCTGCACCTATCCCTTTTTTATCGTTTTCTAAA-CTCGGGTTTGTTCGCGTAAACCGAGATTTGGCTCAGGATTGCCC

HM100_grandiflorum_S574 AGAAACTAAAAAGTAGTAAATTCAAAAAGTTTTGGACCGGGAATTTATTGGATCTTCAAAAAGAATACTTTCTAAG-----TTTGAAA------------ATGTAAATTGTATTCTTG--AATAACTAAAAAAAACAAGGTAAGGTATCAAACAGACTTTTTGGGGGAGTACATTTGGGGATAGAGGGACTTGAACCCTCACGATTTGAAAAGTCAACGGATTTTCATCTTACTATAAATTTCATTGG-TGTCAGTATTGACATGTAGAATGGGACTCTATCTTTATTCTCGTCTTATTAA-----------TAAGTTCTACCAAAGATCTATCAGACTATGAAGTGAATCATTTGA-TCAATGAATATTCGATTTTGTCTTCAACTTCGAATTGATTCACAACATTTTGATTTC--------TTCAAATAG-----AAAAAAAGAAATAGAGATTCAGGTCGTCATTTTTGAGATCGTTTTGTGTTACCTATCTTT-----ATGCAATATAGGTTTTTCTCCTTCATCC----TTTTTGATTTGAAGTTTCGATCGAAGGATTCCTTTATCAACACAA-GGTGGTTGAACTCCATTTGTTAGAACAGCTTCCATTGAGTCTCTGCACCTATCCCTTTTTTATCGTTTTCTAAA-CTCGGGTTTGTTCGCGTAAACCGAGATTTGGCTCAGGATTGCCC

Ae_acarii AGAAACTAAAAAGTAGGAAATTAAAAAAGTTTTGGACCGGTAATTTTTTGGATCTTCGAAAAGAATACTTTCTAAG-----TTTTAAA------------ATTCAAATTGTATTGTTG--AATAGCTAAAAAAAACAAGGTAAGGTGTCAAACAGACTTTTTGGGG-AGTACAGTTGGGGATAGAGGGACTTGAA---------------------------------------------------------------------------------------------------------------------TAAGTTCCACCAAAGATCTATCAGACTATGAAGTGAATCATTTGA-TCAATGAATATTCGATTTTGTCTTCAACTTCGAATTTATTCACAACATTTCTATTTC--------TTAAATATA----AAAAAAAA-AAATAGAGATTCAGGTCGTCGTTTTTGAGATCATTTTGTGTTACCTATCTTT-----ATGCAATATAGGTTTTTCTCCTTCATCC----TTTTTTATTTGAAGTTTCGATCGAAGGATTCCTTTATCAACACAA-GGTAGT-GAACTCCATTTGTTAGAACAGCTTCCATTGAGTCTCTGCACCTATCCCTTTTTTCTCGTTTTCTAAA-CTCGGGTTTGTTCGCGTAAACCGAGATTTGGCTCAGGATTGCCC

Ae_alanyae AGAAACTAAAAAGTAGTAAATTAAAAAAGTTTTGGACCGGGAATTTATTGGATCTTCGAAAAGAATACTTTATAAG-----TTTTAAA------------AAGTAAATTGTATTCTTG--AATACCTAAAAAAAACAAGGTAAGGTGTCAAACAGACTTTTTGGGGGAGTACAGTTGGGGATAGAGGGACTTGAACCCTCACGATTTTAAAAGTCAACGGATTTTCATCTTACTATAAATTTCATTGG-TGTCAGTATTGACATGTAGAATGGGACTCTATCTTTATTCTCGTCTTATTAA-----------TAAGTTCCACCAAAGATCTATCAGACTATGAAGTGAATCATTTGA-TCAATGAATATTCGATTTTGTCTTCAACTTCGAATTGATTCACAACATTTCTATTTC--------TTAAAATAG--AAAAAAAAAAGAAATAGAGATTCAGGTCGTCATTTTTGAGATCGTTTTGTGTTACCTATCTTT-CTTTATGCAATATAGGTTTTTCTCCTTCATCC----TTTTTGAGTTGAAGTTTCGATCGAAGGATTCCTTTATCAACACAA-GGTGGTTGAACTCCATTTGTTAGAACAGCTTCCATTGAGTCTCTGCACCTATCTCTTTTTTATCGTTTTCTAAAACTCGGGTTTGTTCGCGTAAACCGAGATTTGGCTCAGGATTGCCC

Ae_karamanicum AGAAACTAAAAAGTAGTAAATTAAAAAAGTTTTGGACCGGGAATTTATTGGATCTTCGAAAAGAATACTTTATAAG-----TTTTAAA------------AAGTAAATTGTATTCTTG--AATAACTAAAAAAAACAAGGTAAGGTGTCAAACAGACTTTTTGGGGGAGTACAGTTGGGGATAGAGGGACTTGAACCCTCACGATTTTAAAAGTCAACGGATTTTCATCTTACTATAAATTTCATTGG-TGTCAGTATTGACATGTAGAATGGGACTCTATCTTTATTCTCGTCTTATTAA-----------TAAGTTCCACCAAAGATCTATCAGACTATGAAGTGAATCATTTGA-TCAATGAATATTCGATTTTGTCTTCAACTTCGAATTGATTCACAACATTTCTATTTC--------TTAAAATAG---AAAAAAAAAGAAATAGAGATTCAGGTCGTCATTTTTGAGATCGTTTTGTGTTACCTATCTTT-----ATGCAATATAGGTTTTTCTCCTTCATCC----TTTTTGAGTTGAAGTTTCGATCGAAGGATTCCTTTATCAACACAA-GGTGGTTGAACTCCATTTGTTAGAACAGCTTCCATTGAGTCTCTGCACCTATCTCTTTTTTATCGTTTTCTAAAACTCGGGTTTGTTCGCGTAAACCGAGATTTGGCTCAGGATTGCCC

Ae_schistosum AGAAACTAAAAAGTAGTAAATTAAAAAAGTTTTGGACCGGGAATTTTTTGGATCTTCGAAAAGAATACTTTATAAG-----TTTTAAA------------AAGTAAATTGTATTCTTG--AATACCTAAAAAAAACAAGGTAAGGTGTCAAACAGACTTTTTGGGGGAGTACAGTTGGGGATAGAGGGACTTGAACCCTCACGATTTTAAAAGTCAACGGATTTTCATCTTACTATAAATTTCATTGG-TGTCAGTATTGACATGTAGAATGGGACTCTATCTTTATTCTCGTCTTATTAA-----------TAAGTTCCACCAAAGATCTATCAGACTATGAAGTGAATCATTTGA-TCAATGAATATTCGATTTTGTCTTCAACTTCGAATTGATTCACAACATTTCTCTTTC--------TTAAAATAG---AAAAAAAAAGAAATAGAGATTCAGGTCGTCATTTTTGAGATCGTTTTGTGTTACCTATCTTT-----ATGCAATATAGGTTTTTCTCCTTCATCC----TTTTTGAGTTGAAGTTTCGATCGAAGGATTCCTTTATCAACACAA-GGTGGTTGAACTCCATTTGTTAGAACAGCTTCCATTGAGTCTCTGCACCTATCTCTTTTTTATCGTTTTCTAAAACTCGGGTTTGTTCGCGTAAACCGAGATTTGGCTCAGGATTGCCC

Ae_armenum AGAAACTAAAAAGTAGTAAATTAAAAAAGTTTTGGACCGGGAATTTATTGGATCTTCGAAAAGAATACTTTATAAG-----TTTTAAA------------AAGTAAATTGTATTCTTG--AATACCTAAAAAAA-CAAGGTAAGGTGTCAAACAGACTTTTTGGGGGAGTACTGTTGGGGATAGAGGGACTTGAACCCTCACGATTTTAAAAGTCAACGGATTTTCATCTTACTATAAATTTCATTGG-TGTCAGTATTGACATGTAGAATGGGACTCTATCTTTATTCTCGTCTTATTAA-----------TAAGTTCCACCAAAGATCTATCAGACTATGAAGGGAATCATTTGA-TCAATGAATATTCGATTTTGTCTTCAACTTCGAATTGATTCACAACATTTCTATTTC--------TGAAAATAG--AAAAAAAACAGAAATAGAGATTCAGGTCGTCATTTTTGAGATCGTTTTGTGTTACCTATCTTT-----ATGCAATATAGGTTTTTCTCCTTCATCC----TTTTTGAGTTGAAGTTTCGATCGAAAGATTCCTTTATCAATACAA-GGTGGTTGAACTCCATTTGTTAGAACAGCTTCCATTGAGTCTCTGCACCTATCTCTTTTTTCTCGTTTTCTAAAACTCGGGTTTGTTCGCGTAAACCGAGATTTGGCTCAGGATTGCCC

Ae_coridifolium AGAAACTAAAAAGTAGTAAATTAAAAAAGTTTTGGACCGGGAATTTATTGGATCTTCGAAAAGAATACTTTATAAG-----TTTTAAA------------AAGTAAATTGTATTCTTG--AATACCTAAAAAAA-CAAGGTAAGGTGTCAAACAGACTTTTTGGGGGAGTACTGTTGGGGATAGAGGGACTTGAACCCTCACGATTTTAAAAGTCAACGGATTTTCATCTTACTATAAATTTCATTGG-TGTCAGTATTGACATGTAGAATGGGACTCTATCTTTATTCTCGTCTTATTAA-----------TAAGTTCCACCAAAGATCTATCAGACTATGAAGGGAATCATTTGA-TCAATGAATATTCGATTTTGTCTTCAACTTCGAATTGATTCACAACATTTCTATTTC--------TGAAAATAG--AAAAAAAACAGAAATAGAGATTCAGGTCGTCATTTTTGAGATCGTTTTGTGTTACCTATCTTT-----ATGCAATATAGGTTTTTCTCCTTCATCC----TTTTTGAGTTGAAGTTTCGATCGAAGGATTCCTTTATCAATACAA-GGTGGTTGAACTCCATTTGTTAGAACAGCTTCCATTGAGTCTCTGCACCTATCTCTTTTTTCTCGTTTTCTAAAACTCGGGTTTGTTCGCGTAAACCGAGATTTGGCTCAGGATTGCCC

Ae_umbellatum AGAAACTAAAAAGTAGTAAATTAAAAAAGTTTTGGACCGGGAATTTATTGGATCTTCGAAAAGAATACTTTATAAG-----TTTTAAA------------AAGTAAATTGTATTCTTG--AATACCTAAAAAAA-CAAGGTAAGGTGTCAAACAGACTTTTTGGGGGAGTACTGTTGGGGATAGAGGGACTTGAACCCTCACGATTTTTAAAGTCAACGGATTTTCATCTTACTATAAATTTCATTGG-TGTCAGTATTGACATGTAGAATGGGACTCTATCTTTATTCTCGTCTTATTAA-----------TAAGTTCCACCAAAGATCTATCAGACTATGAAGGGAATCGTTTGA-TCAATGAATATTCGATTTTGTCTTCAACTTCGAATTGATTCACAACATTTCTATTTC--------TGAAAATAG--AAAAAAAACAGAAATAGAGATTCAGGTCGTCATTTTTGAGATCGTTTTGTGTTACCTATCTTT-----ATGCAATATAGGTTTTTCTCCTTCATCC----TTTTTGAGTTGAAGTTTCGATCGAAGGATTCCTTTATCAATACAA-GGTGGTTGAACTCCATTTGTTAGAACAGCTTCCATTGAGTCTCTGCACCTATCTCTTTTTTCTCGTTTTCTAAAACTCGGGTTTGTTCGCGTAAACCGAGATTTGGCTCAGGATTGCCC

Ae_diastrophis AGAAACTAAAAAGTAGTAAATTAAAAAAGTTTTGGACCGGGAATTTATTGGATCTTCGAAAAGAATACTTTATAAG-----TTTTAAA------------AAGTAAATTGTATTCTTG--AATACCTAAAAAAA-CAAGGTAAGGTGTCAAACAGACTTTTTGGGGGAGTACAGTTGGGGATAGAGGGACTTGAACCCTCACGATTTTAAAAGTCAACGGATTTTCATCTTACTATAAATTTCATTGG-TGTCAGTATTGACATGTAGAATGGGACTCTATCTTTATTCTCGTCTTATTAA-----------TAAGTTCCACCAAAGATCTATCAGACTATGAAGGGAATCATTTGA-TCAATGAATATTCGATTTTGTCTTCAACTTCGAATTGATTCACAACATTTCTATTTC--------TTAAAATAG---AAAAAAAAAGAAATAGAGATTCAGGTCGTCATTTTTGAGATCGTTTTGTGTTACCTATCTTT-----ATGCAATATAGGTTTTTCTCCTTCATCC----TTTTTGAGTTGAAGTTTCGATCGAAGGATTCCTTTATCAACACAAAGGTGGTTGAACTCCATTTGTTAGAACAGCTTCCATTGAGTCTCTGCACCTATCTCTTTTTTATCGTTTTCTAAAACTCGGGTTTGTTCGCGTAAACCGAGATTTGGCTCAGGATTGCCC

Ae_demirizii AGAAACTAAAAAGTAGTAAATTAAAAAAGTTTTGGACCGGGAATTTATTGGATCTTCGAAAAGAATACTTTATAAG-----TTTTAAA------------AAGTCAATTGTATTCTTG--AATACCTAAAAAAAACAAGGTAAGGTGTCAAACAGACTTTTTGGGGGAGTACAGTTGGGGATAGAGGGACTTGAACCCTCACGATTTTAAAAGTCAACGGATTTTCATCTTACTATAAATTTCATTGG-TGTCAGTATTGACATGTAGAATGGGACTCTATCTTTATTCTCGTCTTATTAA-----------TAAGTTCCACCAAAGATCTATCAGACTATGAAGTGAATCATTTGATTCAATGAATATTCGATTTTGTCTTCAACTTCGAATTGATTCACAACATTTCTATTTC--------TTAAAATAG----AAAAAAAAGAAATAGAGATTCAGGTCGTCATTTTTGAGATCGTTTTGTGTTACCTATCTTT-----ATGCAATATAGGTTTTTCTCCTTCATCC----TTTTTGAGTTGAAGTTTCGATCGAAGGATTCCTTTATCAACACAA-GGTGGTTGAACTCCATTTGTTAGAACAGCTTCCATTGAGTCTCTGCACCTATCTCTTTTTTATCGTTTTCTAAAACTCGGGTTTGTTCGCGTAAACCGAGATTTGGCTCAGGATTGCCC

Ae_glaucinum AGAAACTAAAAAGTAGTAAATTAAAAAAGTTTTGGACCGGGAATTTATTGGATCTTCGAAAAGAATACTTTATAAG-----TTTTAAA------------AAGTCAATTGTATTCTTG--AATACCTAAAAAAAACAAGGTAAGGTGTCAAACAGACTTTTTGGGGGAGTACAGTTGGGGATAGAGGGACTTGAACCCTCACGATTTTAAAAGTCAACGGATTTTCATCTTACTATAAATTTCATTGG-TGTCAGTATTGACATGTAGAATGGGACTCTATCTTTATTCTCGTCTTATTAA-----------TAAGTTCCACCAAAGATCTATCAGACTATGAAGTGAATCATTTGATTCAATGAATATTCGATTTTGTCTTCAACTTCGAATTGATTCACAACATTTCTATTTC--------TTAAAATAG----AAAAAAAAGAAATAGAGATTCAGGTCGTCATTTTTGAGATCGTTTTGTGTTACCTATCTTTATGCAATGCAATATAGGTTTTTCTCCTTCATCC----TTTTTGAGTTGAAGTTTCGATCGAAGGATTCCTTTATCAACACAA-GGTGGTTGAACTCCATTTGTTAGAACAGCTTCCATTGAGTCTCTGCACCTATCTCTTTTTTATCGTTTTCTAAAACTCGGGTTTGTTCGCGTAAACCGAGATTTGGCTCAGGATTGCCC

Ae_huber_morathii AGAAACTAAAAAGTAGTAAATTCAAAAAGTTTTGGACCGGGAATTTATTGGATCTTCAAAAAGAATACTTTCTAAG-----TTTGAAA------------ATGTAAATTGTATTCTTG--AATAACTAAAAAAAACAAGGTAAGGTATCAAACAGACTTTTTGGGGGAGTACATTTGGGGATAGAGGGACTTGAACCCTCACGATTTGAAAAGTCAACGGATTTTCATCTTACTATAAATTTCATTGG-TATCAGTATTGACATGTAGAATGGGACTCTATCTTTATTCTCGTCTTATTAA-----------TAAGTTCCACCAAAGATCTATCAGACTATGAAGTGAATCATTTGA-TCAATGAATATTCGATTTTGTCTTCAACTTCGAATTGATTCACAACATTTTTATTTC--------TTCAAATAG----AAAAAAAAGAAATAGAGATTCAGGTCGTCATTTTTGAGATCGTTTTGTGTTACCTATCTTT-----ATGCAATATAGGTTTTTCTCCTTCATCC----TTTTTGATTTGAAGTTTCGATCGAAGGATTCCTTTATCAACACAA-GGTGGTTGAACTCCATTTGTTAGAACAGCTTCCATTGAGTCTCTGCACCTATCCCTTTTTTATCGTTTTCTAAA-CTCGGGTTTGTTCGCGTAAACCGAGATTTGGCTCAGGATTGCCC

Ae_spicatum AGAAACTAAAAGGTAGTAAATTCAAAAAGTTTTGGACCGGGAATTTCTTGGATCTTCAAAAAGAATACTTTCTAAG-----TTTTAAA------------ATGTAAATTGTATTCTTG--AATAACTAAAAAAAACAAGGTAAGGTATCAAACAGACTTTTTGGGGGAGTACATTTGGGGATAGAGGGACTTGAACCCTCACGATTTGAAAAGTCAACGGATTTTCATCTTACTATAAATTTCATTGG-TGTCAGTATTGACATGTAGAATGGGACTCTATCTTTATTCTCGTCTTATTAA-----------TAAGTTCCACCAAAGATCTATCAGACTATGAAGTGAATCATTTGA-TCAATGAATATTCGATTTTGTCTTCAACTTCGAATTGATTCACAACATTTTTATTTC--------TTCAAATAG----AAAAAAAAGAAATAGAGATTCAGGTCGTCATTTTTGAGATCGTTTTGTGTTACCTATCTTT-----ATGCAATATAGGTTTTTCTCCTTCATCC----TTTTTGATTTGAAGTTTCGATCGAAGGATTCCTTTATCAACACAA-GGTGGTTGAACTCCATTTGTTAGAACAGCTTCCATTGAGTCTCTGCACCTATCCCTTTTTTATCGTTTTCTAAA-CTCGGGTTTGCTCGAGTAAACCGAGATTTGGCTCAGGATTGCCC

HM104_membranaceum_S573 AGAAACTAAAAAGTAGTAAATTAAAAAAGTTTTGGACCGGGAATTTATTGGATCTTCAAAAAGAATACTTTCTAAG-----TTTTCAA------------ATGTAAATTGTATTCTTG--AATAACTAAAAAAAACAAGGTAAGGTGTCAAACAGACTTTTTGGGGGAGTACATTTGGGGATAGAGGGACTTGAACCCTCACGATTTTAAAAGTCAACGGATTTTCATCTTACTATAAATTTCATTGG-TGTCAGTATTGACATGTAGAATGGGACTCTATCTTTATTCTCGTCTTATTAA-----------TAAGTTCCACCAAAGATCTATCAGACTATGAAGTGAATCATTTGA-TCAATGAATATTCGATTTTGTCTTCAACTTCGAATTGATTCACAACATTTCTATTTC--------TTAAAATAGAAAAAAAAAAAAGAAATAGAGATTCAGGTCGTCATTTTTGAGATCGTTTTGTGTTACCTATCTTT-----ATGCAATATAGGTTTTTTTCCTTCATCCTTTTTCTTTGATTTGAAGTTTCGATCGAAGGATTCCTTTATCAACACAA-GGTGGTTGAACTCCATTTGTTAGAACAGCTTCCATTGAGTCTCTGCACCTATCCCTTTTTTATCGTTTTCTAAA-CTCGGGTTTGTTCGCGTAAACCGAGATTTGGCTCAGGATTGCCC

Ae_eunomioides AGAAACTAAAAAGTAGTAAATTAAAAAAGTTTTGGACCGGGAATTTTTTGGATCTTCGAAAAGAATACTTTATAAG-----TTTTAAA------------ATGTAAATTGTATTCTTG--AATAACTAAAAAAAACAAGGCAAGGTGTCAAACAGACTTTTTGGGGGAGTACAGTTGGGGATAGAGGGACTTGAACCCTCACGATTTTAAAAGTCAACGGATTTTCATCTTACTATAAATTTCATTGG-TGTCAGTATTGACATGTAGAATGGGACTCTATCTTTATTCTCGTCTTATTAA-----------TAAGTTCCACCAAAGATCTATCAGACTATGAAGTGAATCATTTGA-TCAATGAATATTCGATTTTGTCTTCAACTTCTAATTGATTCACAACATTTCTATTTC--------TTAAAATAG----AAAAAAAAGAAATAGAGATTCAGGTCGTCATTTTTGAGATCGTTTTGTGTTACCTATCTTT-----ATGCAATATAGGTTTTTCTCCTTCATCC----TTTTTGATTTGAAGTTTCGATCGAAGGATTCCTTTATCAACACAA-GGTGGTTGAACTCCATTTGTTAGAACAGCTTCCATTGAGTCTCTGCACCTATCCCTTTTTTATCGTTTTCTAAA-CTCGGGTTTGTTCGCGTAAACCGAGATTTGGCTCAGGATTGCCC

Ae_capitatum ----------------------------------------------------------------------------------------------------------------TTCTTG--AATAACTAAAAAAAACAAGGTAAGGTATCAAACAGACTTTTTTGGGGAGTACATTTGGGGATAGAGGGACTTGAACCCTCACGATTTGAAAAGTCAACGGATTTTCATCTTACTATAAATTTCATTGG-TGTCAGTATTGACATGTAGAATGGGACTCTATCTTTATTCTCGTCTTATTAATAATTAATAAATAAGTTCCACCAAAGATCTATCAGACTATGAAGTGAATCATTTGA-TCAATGAATATTCGATTTTGTCTTCAACTTCGAATTGATTCACAACATTTTTATTTC--------TTCAAATAG----AAAAAAAAGAAATAGAGATTCAGGTCGTCATTTTTGAGATCGTTTTGTGTTACCTATCTTT-----ATGCAATATAGGTTTTTCTCCTTCATCC----TTTTTGATTTGAAGTTTCGATCGAAGGATTCCTTTATCAACACAA-GGTGGTTGAACTCCATTTGTTAGAACAGCTTCCATTGAGTCTCTGCACCTATCCCTTTTTTATCGTTTTCTAAA-CTCGGGTTTGTTCGCGTAAACCGAGATTTGGCTCAGGATTGCCC

Ae_arabicum AGAAACTAAAAAGTAGGAAATTAAAAAAGTTTTGGACCGGTAATTTTTTGGATCTTCTAAAAGAATACTTTCTAAG-----TTTTAAA------------ATGAAAATTGTATTGTTG--AATAGCTAAAAAAAACAAGGTAAGGTGTCAAACAGACTTTTTGGGG-AGTACAGTTGGGGATAGAGGGACTTGAACCCTCACGATTTTAAAAGTCAACGGATTTTCATCTTACTATAAATTTCATTGG-TGTCAGTATTGACATGTAGAATGGGACTCTATCTTTATTCTCGTCTTATTAA-----------TAAGTTCCACCAAAGATCTATCAGACTATGAAGTGAATCATTTGA-TCAATGAATATTCGATTTTGTCTTCAACTTCGAATTGATTCACAACATTTCTATTTC--------TT-AAATAG----AAAAAAAAGAAATAGAGATTCAAGTCGTCATTTTTGAGATCATTTTGTGTTACCTATCCTT-----ATGCAATATAGGTTTTTCTCCTTCATCC----TTTTTGATTTGAAGTTTCGATCGAAGGATTCCGTTATCAACACAA-GGTAGT-GAACTCCATTTGTTAGAACAGCTTCCATTGAGTCTCTGCACCTATCCCTTTTTTCTCGTTTTCTAAA-CTCGGGTTTGTTCGCG----------------------------

Ae_carneum AGAAACTAAAAAGTAGGAAATTAAAAAAGTTTTGGACCGGTAATTTTTTGGATCTTCTAAAAGAATACTTTCTAAG-----TTTTAAA------------ATGAAAATTGTATTGTTG--AATAGCTAAAAAAAACAAGGTAAGGTGTCAAACAGACTTTTTGGGG-AGTACAGTTGGGGATAGAGGGACTTGAACCCTCACGATTTTAAAAGTCAACGGATTTTCATCTTACTATAAATTTCATTGG-TGTCAGTATTGACATGTAGAATGGGACTCTATCTTTATTCTCGTCTTATTAA-----------TAAGTTCCACCAAAGATCTATCAGACTATGAAGTGAATCATTTGA-TCAATGAATATTCGATTTTGTCTTCAACTTCGAATTGATTCACAACATTTCTATTTC--------TT-AAATAG--AAAAAAAAAAGAAATAGAGATTCAAGTCGTCATTTTTGAGATCATTTTGTGTTACCTATCCTT-----ATGCAATATAGGTTTTTCTCCTTCATCC----TTTTTGATTTGAAGTTTCGATCGAAGGATTCCGTTATCAACACAA-GGTAGT-GAACTCCATTTGTTAGAACAGCTTCCATTGAGTCTCTGCACCTATCCCTTTTTTCTCGTTTTCTAAA-CTCGGGTTTGTTCGCGTAAACCGAGATTTGGC------------

Ae_thesiifolium AGAAACTAAAAAGTAGGAAATTCAAAAAGTTTTGGACCGGTAATTTTTTGGATCTTCGAAAAGCATACTTTCTAAG-----TTTTAAA------------ATGAAAATTGTATTGTTG--AATAGCTAAAAAAAACAAGGTAAGGTGTCAAACAGACTTTTTGGGG-AGTACAGTTGGGGATAGAGGGACTTGAACCCTCACGATTTTAAAAGTCAACGGATTTTCATCTTACTATAAATTTCATTGG-TGTCAGTATTGACATGTAGAATGGGACTCTATCTTTATTCTCGTCTTATTAA-----------TAAGTTCCACCAAAGATCTATCAGACTATGAAGTGAATCATTTGA-TCAATGAATATTCGATTTTGTCTTCAACTTCGAATTGATTCACAACATTTCTATTTC--------TT-AAATAG----AAAAAAAAGAAATAGAGATTCAGGTCGTCATTTTTGAGATCATTTTGTGTTACCTATCTTT-----ATGCAATATAGGTTTTTCTCCTTCATCC----TTTTTGATTTGAAGTTTCGATCGAAGGATTCCTTTATCAACACAA-GGTAGT-GAACTCCATTTGTTAGAACAGCTTCCATTGAGTCTCTGCACCTATCCCTTTTTTCTCGTTTTCTAAA-CTCGGGTTTGTTCGCGTAAACCGAGATTTGGCTCAGGATTGCCC

Ae_orbiculatum AGAAACTAAAAAGTAGGAAATTAAAAAAGTTTTGGACCGGTAATTTTTTGGATCTTCGAAAAGAATACTTTCTAAG-----TTTTAAA------------ATTAAAATTGTATTGTTG--AATAGCTAAAAAAAACAAGGTAAGGTGTCAAACAGACTTTTTGGGG-AGTACAGTTGGGGATAGAGGGACTTGAACCCTCACGATTTTAAAAGTCAACGGATTTTCATCTTACTATAAATTTCATTGG-TGTCAGTATTGACATGTAGAATGGGACTCTATCTTTATTCTCGTGTTATTAA-----------TAAGTTCCACCAAAGATCTATCAGACTATGAAGTGAATCATTTGA-TCAATGAATATTCGATTTTGTCTTCAACTTCGAATTGATTCACAACATTTCTATTTC--------TT-AAATAG--AAAAAAAAAAGAAATAGAGATTCAGGTCGTCATTTTTGAGATCATTTTGTGTTACCTATCTTT-----ATGCAATATAGGTTTTTCTCCTTCATCC----TTTTTTATTTGAAGTTTCGATCGAAGGATTCCTTTATCAACACAA-GGTAGT-GAACTCCATTTGTTAGAACAGCTTCCATTGAGTCTCTGCACCTATCCCTTTTTTCTCGTTTTCTAAA-CTCGGGTTTGTTCGCGTAAACCGAGATTT---------------

Ae_froedinii AGAAACTAAAAAGTAGGAAATTAAAAAAGTTTTGGACCGGTAATTTTTTGGATCTTCTAAAAGAATACTTTCTAAG-----TTTTAAA------------ATGAAAATTGTATTGTTG--AATAGCTAAAAAAAACAAGGTAAGGTGTCAAACAGACTTTTTGGGG-AGTACAGTTGGGGATAGAGGGACTTGAACCCTCACGATTTTAAAAGTCAACGGATTTTCATCTTACTATAAATTTCATTGG-TGTCAGTATTGACATGTAGAATGGGACTCTATCTTTATTCTCGTCTTATTAA-----------TAAGTTCCACCAAAGATCTATCAGACTATGAAGTGAATCATTTGA-TCAATGAATATTCGATTTTGTCTTCAACTTCGAATTGATTCACAACATTTCTATTTC--------TT-AAATAG----AAAAAAAAGAAATAGAGATTCAAGTCGTCATTTTTGAGATCATTTTGTGTTACCTATCCTT-----ATGCAATATAGGTTTTTCTCCTTCATCC----TTTTTGATTTGAAGTTTCGAGCGAAGGATTCCGTTATCAACACAA-GGTAGT-GAACTCCATTTGTTAGAACAGCTTCCATTGAGTCTCTGCACCTATCCCTTTTTTCTCGTTTTCTAAA-CTCGGGTTTGTTCGCGTAAACCGAGATTTGGCTCAGGATTGCCC

Ae_syriacum AGAAACTAAAAAGTAGGAAATTAAAAAAGTTTTGGACCGGTAATTTTTTGGATCTTCTAAAAGAATACTTTCTAAG-----TTTTAAA------------ATGAAAATTGTATTGTTG--AATAGCTAAAAAAAACAAGGTAAGGTGTCAAACAGACTTTTTGGGG-AGTACAGTTGGGGATAGAGGGACTTGAACCCTCACGATTTTAAAAGTCAACGGATTTTCATCTTACTATAAATTTCATTGG-TGTCAGTATTGACATGTAGAATGGGACTCTATCTTTATTCTCGTCTTATTAA-----------TAAGTTCCACCAAAGATCTATCAGACTATGAAGTGAATCATTTGA-TCAATGAATATTCGATTTTGTCTTCAACTTCGAATTGATTCACAACATTTCTATTTC--------TT-AAATAG----AAAAAAAAGAAATAGAGATTCAAGTCGTCATTTTTGAGATCATTTTGTGTTACCTATCCTT-----ATGCAATATAGGTTTTTCTCCTTCATCC----TTTTTGATTTGAAGTTTCGAGCGAAGGATTCCGTTATCAACACAA-GGTAGT-GAACTCCATTTGTTAGAACAGCTTCCATTGAGTCTCTGCACCTATCCCTTTTTTCTCGTTTTCTAAA-CTCGGGTTTGTTCGCGTAAACCGAGATTTGGCTCAGGATTGCCC

Ae_heterocarpum AGAAACTAAAAAGTATGAAATTAAAAAAGTTTGGGACCGGTAATTTTTTGGATCTTCTAAAAGAATACTTTCTAAG-----TTTTAAA------------ATGAAAATTGTATTGTTG--AATAGCTAAAAAAAACAAGGTAAGGTGTCAAACAGACTTTTTGGGG-AGTACAGTTGGGGATAGAGGGACTTGAACCCTCACGATTTTAAAAGTCAACGGATTTTCATCTTACTATAAATTTCATTGG-TGTCAGTATTGACATGTAGAATGGGACTCTATCTTTATTCTCGTCTTATTAA-----------TAAGTTCCACCAAAGATCTATCAGACTATGAAGTGAATCATTTGA-TCAATGAATATTCGATTTTGTCTTCAACTTCGAATTGATTCACAACATTTCTATTTC--------TT-AAATAG----AAAAAAAAGAAATAGAGATTCAAGTCGTCATTTTTGAGATCATTTTGTGTTACCTATCCTT-----ATGCAATATAGGTTTTTCTCCTTCATCC----TTTTTGATTTGAAGTTTCGATCGAAGGATTCCGTTATCAACACAA-GGTAGT-GAACTCCATTTGTTAGAACAGCTTCCATTGAGTCTCTGCACCTATCCCTTTTTTCTCGTTTTCTAAA-CTCGGGTTTGTTCGCGTAAACCGAGATTTGGCTCAGGATTGCCC

Ae_cordatum AGAAACTAAAAAGTAGGAAATTCCAAAAGTTTTGGACCGGTAATTTTTTGGATCTTCGAAAAGAATACTTTCTAAG-----TTTTAAA------------ATGAAAATTGTATTGTTG--AATAGCTAAAAAAAACAAGGTAAGGTGTCAAACAGACTTTTTGGGA-AGTACAGTTGGGGATAGAGGGACTTGAACCCTCACGATTTTAAAAGTCAACGGATTTTCATCTTACTATAAATTTCATTGG-TGTCAGTATTGACATGTAGAATGGGACTCTATCTTTATTCTCGTCTTATTAA-----------GAAGTTCCACCAAAGATCTATCAGACTATGAAGTGAATCATTTGA-TCAATGAATATTCGATTTTGTCTTCAACTTCGAATTGATTCACAACATTTCTATTTC--------TT-AAATAG----AAAAAAAAGAAATAGAGATTCAGGTCGTCATTTTTGAGATCATTTTGTGTTACCTATCTTT-----ATGCAATATAGGTTTTTCTCCTTCATCC----TTTTTGATTTGAAGTTTCGATCGAAGGATTCCTTTATCAACACAA-GGTAGT-GAACTCCATTTGTTAGAACAGCTTCCATTGAGTCTCTGCACCTATCCCTTTTTTCTCGTTTTATAAA-CTCGGGTTTGTTCGCGTAAACCGAGATTTGGCTCAGGATTGCCC

Ae_munzurense AGAAACTAAAAAGTAGGAAATTAAAAAAGTTTGGGACCGGTAATTTTTTGGATCTTCGAAAAGAATACTTTCTAAG-----TTTTAAA------------ATTCAAATTGTATTGTTG--AATAGCTAAAAAAAACAAGGTAAGGTGTCAAACAGACTTTTTGGGG-AGTACAGTTGGGGATAGAGGGACTTGAACCCTCACGATTTTAAAAGTCAACGGATTTTCATCTTACTATAAATTTCATTGG-TGTCAGTATTGACATGTAGAATGGGACTCTATCTTTATTCTCGTGTTATTAA-----------TAAGTTTCACCAAAGATCTATCAGACTATGAAGTGAATCATTTGA-TCAATGAATATTCTATTTTGTCTTCAACTTCGAATTGATTCACAACATTTCTATTTC--------TT-AAATAG---AAAAAAAAAGAAATAGAGATTCAGGTCGTCGTTTTTGAGATCATTTTGTGTTACCTATCTTT-----ATGCAATATAGGTTTTTCTCCTTCATCC----TTTTTTATTTGAAGTTTTGATCGAAGGATTCCTTTATCAACACAA-GGTAGT-GAACTCCATTTGTTAGAACAGCTTCCATTGAGTCTCTGCACCTATCCCTTTTTTCTCGTTTTCTAAA-CTCGGGTTTGTTCGCGTAAACCGAGATTTGGCTCAGGATTGCCC

Ae_papillosum AGAAACTAAAAAGTAGGAAATTAAAAAAGTTTGGGACCGGTAATTTTTTGGATCTTCGAAAAGAATACTTTCTAAG-----TTTTAAA------------ATTAAAATTGTATTGTTG--AATAGCTAAAAAAAACAAGGTAAGGTGTCAAACAGACTTTTTGGGG-AGTACAGTTGGGGATAGAGGGACTTGAACCCTCACGATTTTAAAAGTCAACGGATTTTCATCTTACTATAAATTTCATTGG-TGTCAGTATTGACATGTAGAATGGGACTCTATCTTTATTCTCGTGTTATTAA-----------TAAGTTCCACCAAAGATCTATCAGACTATGAAGTGAATCATTTGA-TCAATGAATATTCTATTTTGTCTTCAACTTCGAATTGATTCACAACATTTCTATTTC--------TT-AAATAG-----AAAAAAAGAAATAGAGATTCCGGTCGTCGTTTTTGAGATCATTTTGTGTTACCTATCTTT-----ATGCAATATAGGTTTTTCTCCTTCATCC----TTTTTTATTTGAAGTTTTGATCGAAGGATTCCTTTATCAACACAA-GGTAGT-GAACTCCATTTGTTAGAACAGCTTCCATTGAGTCTCTGCACCTATCCCTTTTTTCTCGTTTTCTAAA-CTCGGGTTTGTTCGCGTAAACCGAGATTTGGCTCAGGATTGCCC

Ae_lycium AGAAACTAAAAAGTAGGAAATTCAAAAAGTTTTGGACCGGTAATTTTTTGGATCTTCGAAAAGAATACTTTCTAAG-----TTTTAAA------------ATGAAAATTGTATTGTTG--AATAGCTAAAAAAAACAAGGTAAGATGTCAAACAGACTTTTTGGGG-AGTACAGTTGGGGATAGAGGGACTTGAACCCTCACGATTTTAAAAGTCAACGGATTTTCATCTTACTATAAATTTCATTGG-TGTCAGTATTGACATGTAGAATGGGACTCTATCTTTATTCTCGTCTTATTAA-----------TAAGTTCCACCAAAGATCTATCAGACTATGAAGTGAATCATTTGATTCAATGAATATTCGATTTTGTCTTCAACTTCGAATTGATTCACAACATTTCTATTTC--------TT-AAATAG---AAAAAAAAAGAAATAGAGATTCAGGTCGTCATTTTTGAGATCATTTTGTGTTACCTATCTTT-----ATGCAATATAGGTTTTTCTCCTTCATCC----TTTTTGATTTGAAGTTTCGATCGAAGGATTCCTTTATCAACACAA-GGTAGT-GAACTCCATTTGTTAGAACAGCTTCCATTGAGTCTCTGCACCTATCCCTTTTTTCTCGTTTTCTAAA-CTCGGGTTTGTTCGCGTAAACCGAGATTTGGCTCAGGATTGCCC

Ae_turcica AGAAACTAAAAAGTAGGAAATTCAAAAAGTTTTGGACCGGTAATTTTTTGGATCTTCGAAAAGAATACTTTCTAAG-----TTTTAAA------------ATGAAAATTGTATTGTTG--AATAGCTAAAAAAAACAAGGTAAGGTGTCAAACAGACTTTTTGGGG-AGTACAGTTGGGGATAGAGGGACTTGAACCCTCACGATTTTAAAAGTCAACGGATTTTCATCTTACTATAAATTTCATTGG-TGTCAGTATTGACATGTAGAATGGGACTCTATCTTTATTCTCGTCTTATTAA-----------TAAGTTCCACCAAAGATCTATCAGACTATGAAGTGAATCATTTGATTCAATGAATATTCGATTTTGTCTTCAACTTCGAATTGATTCACAACATTTCTATTTC--------TT-AAATAG--AAAAAAAAAAGAAATATAGATTCAGGTCGTCATTTTTGAGATCATTTTGGGTTACCTATCTTT-----ATGCAATATAGGTTTTTCTCCTTCATCC----TTTTTGATTTGAAGTTTCGATCGAAGGATTCCTTTATCAACACAA-GGTAGT-GAACTCCATTTGTTAGAACAGCTTCCATTGAGTCTCTGCACCTATCCCTTTTTTCTCGTTTTCTAAA-CTCGGGTTTGTTCGCATAAACCGAGATTTGGCTCAGGATT----

Ae_fimbriatum ---AACTAAAAAGTCKGAAATTAAAAAAGTTTTGGACCGGTAATTTTTTGGATCTTCGAAAAGAATACTTTCTAAG-----TTTTCAA------------ATTAAAATTGTATTGTTG--AATAGTTAAAAAAAACAAGGTAAGGTGTCAAACAGACTTTTTGAGG-AGTACAGTTGGGGATAGAGGGACTTGAACCCTCACGATTTTAAAAGTCAACGGATTTTCATCTTACTATAAATTTCATTGG-TGTCAGTATTGACATGTAGAATGGGACTCTATCTTTATTCTCGTGTTATTAA-----------TAAGTTCCACCAAAGATCTATCAGACTATGAAGTGAATCATTTGA-TCAATGAATATTCGATTTTGTCTTCAACTTCGAATTGATTCACAACATTGCTATTTC--------TT-AAATAG----AAAAAAAAGAAATAGAGATTCAGGTCGTCGTTTTTGAGATCATTTTGTGTTACCTATCTTT-----ATGCAATATAGGTTTTTCTCCTTCATCC----TTTTTTATTTGAAGTTTTGATCGAAGGATTCCTTTATCAACACAA-GGTAGT-GAACTCCATTTGTTAGAACAGCTTCCATTGAGTCTCTGCACCTATCCCTTTTTTCTCGTTTTCTAAA-CTCGGGTTTGTTCGCGTAAACCGAGATTTGGC------------

Ae_speciosum AGAAACTAAAAAGTAGGAAATTCAAAAAGTTTGGGACCGGTAATTTTTTGGATCTTCGAAAAGAATACTTTCTAAG-----TTTTAAA------------ATTAAAATTGTATTGTTG--AATAGCTAAAAAAAACAAGGTAAGGTGTCAAACAGACTTTTTGGGG-AGTACAGTTGGGGATAGAGGGACTTGAACCCTCACGATTTTAAAAGTCAACGGATTTTCATCTTACTATAAATTTCATTGG-TGTCAGTATTGACATGTAGAATGGGACTCTATCTTTATTCTCGTGTTATTAA-----------TACGTTCCACCAAAGATCTATCAGACTATGAAGTGAATCATTTGA-TCAATGAATATTCTATTTTGTCTTCAACTTCGAATTGATTCACAACATTTCTATTTC--------TT-AAATAG----AAAAAAAAGAAATAGAGATTCAGGTCGTCGTTTTTGAGATCATTTTGTGTTACCTATCTTT-----ATGCAATATAGGTTTTTCTCCTTCATCC----TTTTTTATTTGAAGTTTTGATCGAAGGATTCCTTTATCAACACAA-GGTAGT-GAACTCCATTTGTTAGAACAGCTTCCATTGAGTCTCTGCACCTATCCCTTTTTTCTCGTTTTCTAAA-CTCGGGTTTGTTCGCGTAAACCGAGATTTGGCTCAGGATTGCCC

Ae_stylosum AGAAACTAAAAAGTAGGAAATTAAAAAAGTTTTGGACCGGTAATTTTTTGGATCTTCGAAAAGAATACTTTCTAAGCTAAGTTTTAAA------------ATGAAAATTGTATTGTTG--AATAGCTAAAAAAAACAAGGTAAGGTGTCAAACAGACTTTTTAGGG-AGTACAGTTGGGGATAGAGGGACTTGAACCCTCACGATTTTAAAAGTCAACGGATTTTCATCTTACTATAAATTTCATTGG-TGTCAGTATTGACATGTAGAATGGGACTCTATCTTTATTCTCGTGTTATTAA-----------TAAGTTCCACCAAAGATCTATCAGACTATGAAGTGAATCATTTGA-TCAATGCATATTCGATTTTGTCTTCAACTTCGAATTGATTCACAACATTTCTATTTC--------TT-AAATAG-AAAAAAAAAAAGAAATAGAGATTCAGGTCGTCGTTTTTGAGATCATTTTGTGTTACCTATCTTT-----ATGCAATATAGGTTTTTCTCCTTCATCC----TTTTTTATTTGAAGTTTTGATCGAAGGATTCCTTTATCAACACAA-GGTAGT-GAACTCCATTTGTTAGAACAGCTTCCATTGAGTCTCTGCACCTATCCCTTTTTTCTCGTTTTCTAAA-CTCGGGTTTGTTCGCGTAAACCGAGATTTGGC------------

Ae_dumanii -------AAAAAGTATGAAATTTAAAAAGTTTTGGACCGGTAATTTTTTGGATCTTCGAAAAGAATACTTTCTAAG-----TTTTAAA------------ATGAAAATTGTATTGTTG--AATAGCTAAAAAAAACAAGGTAAGGTGTCAAACAGACTTTTTGGGT-AGTACAGTTGGGGATAGAGGGACTTGAACCCTCACGATTTTAAAAGTCAACGGATTTTCATCTTACTATAAATTTCATTGG-TGTCAGTATTGACATGTAGAATGGGACTCTATCTTTATTCTCGTCTTATTAA-----------TAAGTTCCACCAAAGATC---CAGACTATGAAGTGAATCATTTTA-TCAATGAATATTCGATTTTGTCTTCAACTTCGAATTGATTCACAACATTTCTATTTC--------TT-AAATAG---AAAAAAAAAGAAATAGAGATTCAAGTCGTCATTTTTGAGATCATTTTGTGTTACCTATCTTT-----ATGCAATATAGGTTTTTCTCCTTCATCC----TTTTTGATTTGAAGTTTCGATCGAAGGATTCCTTTATCAACACAA-GGTAGT-GAACTCCATTTGTTAGAACAGCTTCCATTGAGTCTCTGCACCTATCCCTTTTTTCTCGTTTTCTAAA-CTCGGGTTTGTTCGCGTAAACCGAGATTTGGCTCAGGATTGCCC

Ae_stenopterum AGAAACTAAAAAGTAGGAAATTCAAAAAGTTTTGGACCGGTAATTTTTTGGATCTTCGAAAAGAATACTTTCTAAG-----TTTTAAACTAAGTTTTAAAATTAAAATTGTATTGTTG--AATAGCTAAAAAAAACAAGGTAAGGTGTCAAACAGACTTTTTGGGG-AGTACAGTTGGGGATAGAGGGACTTGAACCCTCACGATTTTAAAAGTCAACGGATTTTCATCTTACTATAAATTTCATTGG-TGTCAGTATTGACATGTAGAATGGGACTCTATCTTTATTCTCGTGTTATTAA-------TTAATAAGTTCCACCAAAGATCTATCAGACTATGAAGTGAATCATTTGA-TCAATGAATATTCGATTTTGTCTTCAACTTCGAATTGATTCACAACATTTCTATTTC--------TT-AAATAG---AAAAAAAAAGAAATAGAGATTCAAGTCGTCATTTTTGAGATCATTTTGTGTTACCTATCCTT-----ATGCAATATAGGTTTTTCTCCTTCATCC----TTTTTGATTTGAAGTTTCGATCGAAGGATTCCGTTATCAACACAA-GGTAGT-GAACTCCATTTGTTAGAACAGCTTCCATTGAGTCTCTGCACCTATCCCTTTTTTCTCGTTTTCTAAA-CTCGGGTTTGTTCGCGTAAACCGAGATTTGGCTCAGGATTGCCC

Ae_saxatile AGAAACTAAAAAGTAGGAAATTAAAAAAGTTTTGGACCGGTAATTTTTTGGATCTTC--------------------------------------------------TTGTATTGTTG--AATAGCTAAAAAAAACAAGGTAAGGTGTCAAACAGACTTTTTGGGG-AGTACAGTTGGGGATAGAGGGACTTGAACCCTCACGATTTTAAAAGTCAACGGATTTTCATCTTACTATAAATTTCATTGG-TGTCAGTATTGACATGTAGAATAGGACTCTATCTTTATTCTCGTGTTATTAA-----------TAAGTTCCACCAAAGATCTATCAGACTATGAAGTGAATCATTTGA-TCAATGAATATTCGATTTTGTCTTCAACTTCGAATTTATTCACAACATTTCTATTTC--------TT-AAATAG---AAAAAAAAAGAAATAGAGATTCAGGTCGTCGTTTTTGAGATCATTTTGTGTTACCTATCTTT-----ATGCAATATAGGTTTTTCTCCTTCATCC----TTTTTTATTTGAAGTTTCGATCGAAGGATTCCTTTATCAACACAA-GGTAGT-GAACTCCATTTGTTAGAACAGCTTCCATTGAGTCTCTGCACCTATCCCTTTTTTCTCGTTTTCTAAA-CTCGGGTTTGTTCGCGTAAACCGAGATTTGGCTCAGGATTGCCC

Ae_lepidioides CGAAACTCAAAAT---------------GTTTTGGACCGGGAATTTTTTGGATCTTCGAAAAGAAGACTTTAGAAG-----TTTTAAA------------ATGAAAATTGTCTTCTTTTCGAAGATCCAAAAAAAAAAGGTAAGGTGTCAAACAGACTTT----------ATAGTTGGGGATAGAGGGACTTGAACCCTCACGATTTTAAAAGTCAACGGATTTTCATCTTACTATAAATTTCATTGG-TGTCAGTATTGACATGTAAAATGGGACTCTATCTTTATTCTCGTCTTATTAA-----------TAAGTTCCACCAAAGATTTAGCAGACTATAAAGTGAATCATTTGA-TCAATGAATATTAGATTTTGTC----ACTTCGAATTGATTCACAACATTTCTATTTC--------T--AAAAA----AAAAAAAAAGAAATAGAGATTCAGGTCGTCAGTTTTGAGATCGTTTTGTGTTACCTATATTT-----ATGCAATATAGGTTTTTCTCCTTCATCC----TTTTTGATTTTAAGTTTCGATCGAAGGATTCCTTTATCAACACAA-GGTAGT-GAATTCCATTTGTTAGAACAGCTTCCATTGAGTCTCTGCACCTATCCCTTTTTTATCGTTTTCTAAA-CTCGGGTTTGTTCGCGTAAACCGAGATTTGGCTCAGGATTGCCC

HM95_spinosum_S592 CGAAACTCAAAAGTAGTAAATTCAAAAAGTTTTGGACCGGGAATTTTTTTGATCTTCGAAAAGAAGACTTTAGAAG-----TTTTAAA------------ATGAAAATTGTATTCTTG--AATAACTAAAAAAA--AAGGTAAGGTGTCAAACAGACTTTTTGGGGGAGTATAGTTGGGGATAGAGGGACTTGAACCCTCACGATTTTAAAAGTCAACGGATTTTCATCTTACTATAAATTTCATTGG-TGTCAGTATTGACATGTAGAATGGGACTCTATCTTTATTCTCGTCTTATTAA-----------TAAGTTCCACCAAAGATCTATCAGACTATGAAGTGAATCATTTGA-TCAATGAATAGTAGATTTTGTCTTTAACTTCGAATTGATTCACAACATTTCTATTTC--------T--AAA------AAAAAAAAAGAAATAGAGATTCAGGTCGTCATTTTTGAGATCGTTTTGTGTTACCTATATTT-----ATGCAATATAGGTTTTTCTCTTTCATCC----TTTTTGATTTGAAGTTTCGATCGAAGGATTCCTTTATCAACACAA-GGTAGT-GAACTCCATTTGTTAGAACAGCTTCCATTGAGTCTCTGCACCTATCCCTTTTTTATCGTTTTCTAAA-CTCGGGTTTGTTCGCGTAAACCGAGATTTGGCTCAGGATTGCCC

;

end;

begin mrbayes;

set autoclose=yes nowarn=yes;

lset nst=6 rates=gamma;

unlink statefreq=(all) revmat=(all) shape=(all) pinvar=(all);

prset applyto=(all) ratepr=variable;

mcmcp ngen=20000000 nruns=4 nchains=4 temp=0.2 samplefreq=1000 savebrlens=yes printfreq=1000;

mcmc;

sump burninfrac=0.1;

sumt burninfrac=0.1 contype=halfcompat conformat=Figtree outputname=Aethionema_30_12_2022_trnL_true.tre;

end;
