# Supplementary material for: Underestimated diversity in high elevations of a global biodiversity hotspot: two new endemic species of Aethionema (Brassicaceae) from the alpine zone of Iran
Source: Front Plant Sci. 2023 May 26;14:1182073. doi: 10.3389/fpls.2023.1182073 (PMC10250747; doi:10.3389/fpls.2023.1182073)
Supplement: Supplementary file 2 [file DataSheet_2.zip › Date Sheet 2/trnLF/Jmodeltest_output.docx]

-------------------------- jModeltest 2.1.10 v20160303 --------------------------

(c) 2011-onwards D. Darriba, G.L. Taboada, R. Doallo and D. Posada,

(1) Department of Biochemistry, Genetics and Immunology

University of Vigo, 36310 Vigo, Spain.

(2) Department of Electronics and Systems

University of A Coruna, 15071 A Coruna, Spain.

e-mail: ddarriba@udc.es, dposada@uvigo.es

--------------------------------------------------------------------------------

Thu Dec 29 23:00:10 PST 2022

Linux 4.18.0-348.23.1.el8_5.x86_64, arch: amd64, bits: 64, numcores: 32

--------------------------------------------------------------------------------

Citation: Darriba D, Taboada GL, Doallo R and Posada D. 2012.

"jModelTest 2: more models, new heuristics and parallel computing".

Nature Methods 9(8), 772.

--------------------------------------------------------------------------------

jModelTest 2.1.10 v20160303

Copyright (C) 2011 D. Darriba, G.L. Taboada, R. Doallo and D. Posada

This program comes with ABSOLUTELY NO WARRANTY

This is free software, and you are welcome to redistribute it under certain

conditions

Notice: This program may contain errors. Please inspect results carefully.

Arguments = -tr 32 --set-property log-dir=./ -d infile.phy -o output.txt -s 11 -f -i -g 4 -t ML -AIC -AICc -BIC -DT -S BEST -p -a -w

Reading data file "infile.phy"... OK.

number of sequences: 48

number of sites: 699

---------------------------------------------------------------

* *

* COMPUTATION OF LIKELIHOOD SCORES WITH PHYML *

* *

---------------------------------------------------------------

::Settings::

Phyml version = 20130103

Phyml binary = PhyML_3.0_linux64

Phyml path = /expanse/projects/ngbt/opt/expanse/jmodeltest2/2.1.10/exe/phyml/

Candidate models = 88

number of substitution schemes = 11

including models with equal/unequal base frequencies (+F)

including models with/without a proportion of invariable sites (+I)

including models with/without rate variation among sites (+G) (nCat = 4)

Optimized free parameters (K) = substitution parameters + 93 branch lengths + topology

Base tree for likelihood calculations = ML tree

Tree topology search operation = BEST

::Progress::

Model Exec. Time Total Time -lnL

-------------------------------------------------------------------------

F81+G 00h:00:17:03 00h:00:17:05 1978.6974 (1/88)

HKY+G 00h:00:17:06 00h:00:17:07 1978.6898 (2/88)

TrN+G 00h:00:17:09 00h:00:18:00 1978.5708 (3/88)

TPM2uf+G 00h:00:18:01 00h:00:18:02 1977.1756 (4/88)

TIM2+G 00h:00:18:04 00h:00:18:06 1977.0270 (5/88)

TIM1+G 00h:00:18:06 00h:00:18:07 1954.3187 (6/88)

TPM1uf+G 00h:00:18:08 00h:00:18:09 1954.4443 (7/88)

TPM3uf+G 00h:00:20:02 00h:00:20:03 1975.2044 (8/88)

TIM3+G 00h:00:20:00 00h:00:20:04 1975.1321 (9/88)

TVM+G 00h:00:22:05 00h:00:22:09 1953.9252 (10/88)

TPM1+I+G 00h:00:26:09 00h:00:27:00 2009.2134 (11/88)

TIM1ef+I+G 00h:00:26:09 00h:00:27:00 2009.1669 (12/88)

SYM+I+G 00h:00:27:02 00h:00:27:03 2008.0865 (13/88)

TPM2+I+G 00h:00:27:05 00h:00:27:06 2026.9684 (14/88)

TPM3+I+G 00h:00:27:09 00h:00:27:09 2026.9374 (15/88)

TIM2ef+I+G 00h:00:28:00 00h:00:28:01 2026.9204 (16/88)

TrNef+I+G 00h:00:28:00 00h:00:28:01 2026.9213 (17/88)

JC+I+G 00h:00:28:01 00h:00:28:02 2027.0866 (18/88)

TVM+I+G 00h:00:28:03 00h:00:28:03 1953.6242 (19/88)

K80+I+G 00h:00:28:06 00h:00:28:07 2026.9702 (20/88)

TPM3uf+I+G 00h:00:28:08 00h:00:28:08 1974.8852 (21/88)

F81+I+G 00h:00:29:01 00h:00:29:01 1978.3611 (22/88)

HKY+I+G 00h:00:29:03 00h:00:29:03 1978.3541 (23/88)

GTR+I+G 00h:00:30:00 00h:00:30:00 1953.5379 (24/88)

TIM1+I+G 00h:00:30:01 00h:00:30:01 1954.0317 (25/88)

TrN+I+G 00h:00:30:03 00h:00:30:03 1978.2428 (26/88)

TVMef+I+G 00h:00:30:03 00h:00:30:03 2008.1339 (27/88)

TPM1uf+I+G 00h:00:30:04 00h:00:30:04 1954.1512 (28/88)

TIM3ef+I+G 00h:00:30:03 00h:00:30:04 2026.8867 (29/88)

TPM2uf+I+G 00h:00:31:04 00h:00:31:04 1976.8670 (30/88)

TIM2+I+G 00h:00:31:07 00h:00:31:08 1976.7256 (31/88)

TIM3+I+G 00h:00:33:00 00h:00:33:01 1974.8205 (32/88)

JC+G 00h:00:17:02 00h:00:34:09 2027.4858 (33/88)

K80+G 00h:00:17:02 00h:00:35:02 2027.3698 (34/88)

TrNef+G 00h:00:17:07 00h:00:35:09 2027.3157 (35/88)

TPM1+G 00h:00:18:01 00h:00:36:06 2009.6251 (36/88)

GTR+G 00h:00:19:02 00h:00:36:08 1953.8321 (37/88)

TPM3+G 00h:00:17:09 00h:00:36:09 2027.3325 (38/88)

F81+I 00h:00:10:05 00h:00:37:08 1979.8439 (39/88)

TPM2+G 00h:00:19:01 00h:00:37:09 2027.3687 (40/88)

TIM2ef+G 00h:00:17:07 00h:00:38:00 2027.3147 (41/88)

HKY+I 00h:00:10:07 00h:00:38:03 1979.8377 (42/88)

TrN+I 00h:00:11:00 00h:00:39:00 1979.7105 (43/88)

TPM2uf+I 00h:00:10:09 00h:00:39:01 1978.3890 (44/88)

TPM3uf+I 00h:00:11:01 00h:00:39:03 1976.4350 (45/88)

TPM1uf+I 00h:00:11:04 00h:00:39:05 1955.7623 (46/88)

TIM3+I 00h:00:10:09 00h:00:39:07 1976.3496 (47/88)

TIM1+I 00h:00:11:04 00h:00:39:08 1955.6293 (48/88)

JC+I 00h:00:09:08 00h:00:39:09 2028.5764 (49/88)

TIM1ef+G 00h:00:19:07 00h:00:40:00 2009.5724 (50/88)

K80+I 00h:00:10:03 00h:00:40:04 2028.4603 (51/88)

TVM+I 00h:00:11:04 00h:00:40:05 1955.2524 (52/88)

TPM1+I 00h:00:10:02 00h:00:40:05 2010.7328 (53/88)

TIM3ef+G 00h:00:17:07 00h:00:40:06 2027.2755 (54/88)

TPM3+I 00h:00:10:05 00h:00:40:09 2028.4275 (55/88)

GTR+I 00h:00:11:09 00h:00:41:02 1955.1488 (56/88)

TrNef+I 00h:00:11:00 00h:00:41:03 2028.4017 (57/88)

TPM2+I 00h:00:11:01 00h:00:41:05 2028.4330 (58/88)

TIM1ef+I 00h:00:10:05 00h:00:42:00 2010.6759 (59/88)

TIM2+I 00h:00:14:03 00h:00:43:00 1978.2374 (60/88)

F81 00h:00:07:03 00h:00:43:01 1990.0650 (61/88)

TIM2ef+I 00h:00:12:04 00h:00:44:02 2028.3752 (62/88)

HKY 00h:00:07:06 00h:00:44:03 1990.0428 (63/88)

TrN 00h:00:07:08 00h:00:44:06 1989.7305 (64/88)

TVMef+G 00h:00:17:07 00h:00:44:07 2008.5714 (65/88)

TPM1uf 00h:00:07:08 00h:00:44:07 1965.1643 (66/88)

TIM3ef+I 00h:00:11:08 00h:00:44:09 2028.3659 (67/88)

SYM+G 00h:00:18:01 00h:00:45:01 2008.5193 (68/88)

TVMef+I 00h:00:10:05 00h:00:45:04 2009.6551 (69/88)

TPM3uf 00h:00:07:07 00h:00:45:06 1986.4982 (70/88)

TIM1 00h:00:07:09 00h:00:46:00 1964.8528 (71/88)

SYM+I 00h:00:10:07 00h:00:46:00 2009.5984 (72/88)

TPM2uf 00h:00:08:06 00h:00:46:05 1987.9786 (73/88)

JC 00h:00:07:02 00h:00:46:07 2039.9929 (74/88)

TIM3 00h:00:07:08 00h:00:46:07 1986.2188 (75/88)

K80 00h:00:07:01 00h:00:46:08 2039.8816 (76/88)

TrNef 00h:00:07:01 00h:00:46:09 2039.6663 (77/88)

TVM 00h:00:07:08 00h:00:46:09 1964.8159 (78/88)

GTR 00h:00:07:08 00h:00:47:01 1964.5188 (79/88)

TIM2 00h:00:08:08 00h:00:47:01 1987.6479 (80/88)

TPM1 00h:00:07:02 00h:00:47:01 2022.2779 (81/88)

TPM2 00h:00:07:03 00h:00:47:03 2039.6883 (82/88)

TIM1ef 00h:00:07:01 00h:00:47:06 2022.0630 (83/88)

TVMef 00h:00:06:08 00h:00:47:07 2021.6803 (84/88)

TIM2ef 00h:00:07:02 00h:00:47:07 2039.4725 (85/88)

TPM3 00h:00:07:03 00h:00:47:08 2039.6692 (86/88)

SYM 00h:00:06:07 00h:00:47:09 2021.4658 (87/88)

TIM3ef 00h:00:07:03 00h:00:48:00 2039.4543 (88/88)

Model = JC

partition = 000000

-lnL = 2039.9929

K = 94

Model = JC+I

partition = 000000

-lnL = 2028.5764

K = 95

p-inv = 0.5820

Model = JC+G

partition = 000000

-lnL = 2027.4858

K = 95

gamma shape = 0.4220

Model = JC+I+G

partition = 000000

-lnL = 2027.0866

K = 96

p-inv = 0.3960

gamma shape = 1.4000

Model = F81

partition = 000000

-lnL = 1990.0650

K = 97

freqA = 0.3055

freqC = 0.1649

freqG = 0.1689

freqT = 0.3607

Model = F81+I

partition = 000000

-lnL = 1979.8438

K = 98

freqA = 0.3055

freqC = 0.1652

freqG = 0.1701

freqT = 0.3593

p-inv = 0.5610

Model = F81+G

partition = 000000

-lnL = 1978.6974

K = 98

freqA = 0.3056

freqC = 0.1651

freqG = 0.1701

freqT = 0.3592

gamma shape = 0.4710

Model = F81+I+G

partition = 000000

-lnL = 1978.3611

K = 99

freqA = 0.3057

freqC = 0.1651

freqG = 0.1701

freqT = 0.3591

p-inv = 0.3590

gamma shape = 1.3640

Model = K80

partition = 010010

-lnL = 2039.8816

K = 95

kappa = 0.9200 (ti/tv = 0.4600)

Model = K80+I

partition = 010010

-lnL = 2028.4603

K = 96

kappa = 0.9177 (ti/tv = 0.4589)

p-inv = 0.5820

Model = K80+G

partition = 010010

-lnL = 2027.3698

K = 96

kappa = 0.9177 (ti/tv = 0.4589)

gamma shape = 0.4220

Model = K80+I+G

partition = 010010

-lnL = 2026.9702

K = 97

kappa = 0.9175 (ti/tv = 0.4587)

p-inv = 0.3960

gamma shape = 1.3990

Model = HKY

partition = 010010

-lnL = 1990.0428

K = 98

freqA = 0.3056

freqC = 0.1648

freqG = 0.1689

freqT = 0.3608

kappa = 1.0383 (ti/tv = 0.4624)

Model = HKY+I

partition = 010010

-lnL = 1979.8377

K = 99

freqA = 0.3055

freqC = 0.1651

freqG = 0.1700

freqT = 0.3593

kappa = 1.0206 (ti/tv = 0.4554)

p-inv = 0.5610

Model = HKY+G

partition = 010010

-lnL = 1978.6898

K = 99

freqA = 0.3057

freqC = 0.1650

freqG = 0.1700

freqT = 0.3593

kappa = 1.0225 (ti/tv = 0.4561)

gamma shape = 0.4720

Model = HKY+I+G

partition = 010010

-lnL = 1978.3541

K = 100

freqA = 0.3057

freqC = 0.1651

freqG = 0.1701

freqT = 0.3592

kappa = 1.0215 (ti/tv = 0.4557)

p-inv = 0.3580

gamma shape = 1.3630

Model = TrNef

partition = 010020

-lnL = 2039.6663

K = 96

R(a) [AC] = 1.0000

R(b) [AG] = 1.0115

R(c) [AT] = 1.0000

R(d) [CG] = 1.0000

R(e) [CT] = 0.8342

R(f) [GT] = 1.0000

Model = TrNef+I

partition = 010020

-lnL = 2028.4017

K = 97

R(a) [AC] = 1.0000

R(b) [AG] = 0.9659

R(c) [AT] = 1.0000

R(d) [CG] = 1.0000

R(e) [CT] = 0.8682

R(f) [GT] = 1.0000

p-inv = 0.5800

Model = TrNef+G

partition = 010020

-lnL = 2027.3157

K = 97

R(a) [AC] = 1.0000

R(b) [AG] = 0.9637

R(c) [AT] = 1.0000

R(d) [CG] = 1.0000

R(e) [CT] = 0.8704

R(f) [GT] = 1.0000

gamma shape = 0.4260

Model = TrNef+I+G

partition = 010020

-lnL = 2026.9213

K = 98

R(a) [AC] = 1.0000

R(b) [AG] = 0.9607

R(c) [AT] = 1.0000

R(d) [CG] = 1.0000

R(e) [CT] = 0.8727

R(f) [GT] = 1.0000

p-inv = 0.3890

gamma shape = 1.3720

Model = TrN

partition = 010020

-lnL = 1989.7305

K = 99

freqA = 0.3039

freqC = 0.1664

freqG = 0.1673

freqT = 0.3624

R(a) [AC] = 1.0000

R(b) [AG] = 1.1693

R(c) [AT] = 1.0000

R(d) [CG] = 1.0000

R(e) [CT] = 0.9205

R(f) [GT] = 1.0000

Model = TrN+I

partition = 010020

-lnL = 1979.7105

K = 100

freqA = 0.3042

freqC = 0.1662

freqG = 0.1690

freqT = 0.3606

R(a) [AC] = 1.0000

R(b) [AG] = 1.1045

R(c) [AT] = 1.0000

R(d) [CG] = 1.0000

R(e) [CT] = 0.9387

R(f) [GT] = 1.0000

p-inv = 0.5580

Model = TrN+G

partition = 010020

-lnL = 1978.5708

K = 100

freqA = 0.3044

freqC = 0.1661

freqG = 0.1690

freqT = 0.3605

R(a) [AC] = 1.0000

R(b) [AG] = 1.1032

R(c) [AT] = 1.0000

R(d) [CG] = 1.0000

R(e) [CT] = 0.9432

R(f) [GT] = 1.0000

gamma shape = 0.4790

Model = TrN+I+G

partition = 010020

-lnL = 1978.2427

K = 101

freqA = 0.3044

freqC = 0.1661

freqG = 0.1691

freqT = 0.3604

R(a) [AC] = 1.0000

R(b) [AG] = 1.0998

R(c) [AT] = 1.0000

R(d) [CG] = 1.0000

R(e) [CT] = 0.9443

R(f) [GT] = 1.0000

p-inv = 0.3600

gamma shape = 1.3990

Model = TPM1

partition = 012210

-lnL = 2022.2779

K = 96

R(a) [AC] = 1.0000

R(b) [AG] = 0.5857

R(c) [AT] = 0.2713

R(d) [CG] = 0.2713

R(e) [CT] = 0.5857

R(f) [GT] = 1.0000

Model = TPM1+I

partition = 012210

-lnL = 2010.7328

K = 97

R(a) [AC] = 1.0000

R(b) [AG] = 0.5831

R(c) [AT] = 0.2662

R(d) [CG] = 0.2662

R(e) [CT] = 0.5831

R(f) [GT] = 1.0000

p-inv = 0.5840

Model = TPM1+G

partition = 012210

-lnL = 2009.6251

K = 97

R(a) [AC] = 1.0000

R(b) [AG] = 0.5832

R(c) [AT] = 0.2656

R(d) [CG] = 0.2656

R(e) [CT] = 0.5832

R(f) [GT] = 1.0000

gamma shape = 0.4170

Model = TPM1+I+G

partition = 012210

-lnL = 2009.2134

K = 98

R(a) [AC] = 1.0000

R(b) [AG] = 0.5828

R(c) [AT] = 0.2650

R(d) [CG] = 0.2650

R(e) [CT] = 0.5828

R(f) [GT] = 1.0000

p-inv = 0.3850

gamma shape = 1.3050

Model = TPM1uf

partition = 012210

-lnL = 1965.1643

K = 99

freqA = 0.3128

freqC = 0.1596

freqG = 0.1619

freqT = 0.3658

R(a) [AC] = 1.0000

R(b) [AG] = 0.5880

R(c) [AT] = 0.2139

R(d) [CG] = 0.2139

R(e) [CT] = 0.5880

R(f) [GT] = 1.0000

Model = TPM1uf+I

partition = 012210

-lnL = 1955.7623

K = 100

freqA = 0.3131

freqC = 0.1598

freqG = 0.1628

freqT = 0.3643

R(a) [AC] = 1.0000

R(b) [AG] = 0.5880

R(c) [AT] = 0.2129

R(d) [CG] = 0.2129

R(e) [CT] = 0.5880

R(f) [GT] = 1.0000

p-inv = 0.5390

Model = TPM1uf+G

partition = 012210

-lnL = 1954.4443

K = 100

freqA = 0.3134

freqC = 0.1596

freqG = 0.1626

freqT = 0.3644

R(a) [AC] = 1.0000

R(b) [AG] = 0.5881

R(c) [AT] = 0.2111

R(d) [CG] = 0.2111

R(e) [CT] = 0.5881

R(f) [GT] = 1.0000

gamma shape = 0.5160

Model = TPM1uf+I+G

partition = 012210

-lnL = 1954.1512

K = 101

freqA = 0.3134

freqC = 0.1596

freqG = 0.1627

freqT = 0.3643

R(a) [AC] = 1.0000

R(b) [AG] = 0.5879

R(c) [AT] = 0.2110

R(d) [CG] = 0.2110

R(e) [CT] = 0.5879

R(f) [GT] = 1.0000

p-inv = 0.3370

gamma shape = 1.3800

Model = TPM2

partition = 010212

-lnL = 2039.6883

K = 96

R(a) [AC] = 0.8831

R(b) [AG] = 0.8587

R(c) [AT] = 0.8831

R(d) [CG] = 1.0000

R(e) [CT] = 0.8587

R(f) [GT] = 1.0000

Model = TPM2+I

partition = 010212

-lnL = 2028.4330

K = 97

R(a) [AC] = 0.9519

R(b) [AG] = 0.8933

R(c) [AT] = 0.9519

R(d) [CG] = 1.0000

R(e) [CT] = 0.8933

R(f) [GT] = 1.0000

p-inv = 0.5800

Model = TPM2+G

partition = 010212

-lnL = 2027.3687

K = 97

R(a) [AC] = 0.9902

R(b) [AG] = 0.9127

R(c) [AT] = 0.9902

R(d) [CG] = 1.0000

R(e) [CT] = 0.9127

R(f) [GT] = 1.0000

gamma shape = 0.4220

Model = TPM2+I+G

partition = 010212

-lnL = 2026.9684

K = 98

R(a) [AC] = 0.9944

R(b) [AG] = 0.9147

R(c) [AT] = 0.9944

R(d) [CG] = 1.0000

R(e) [CT] = 0.9147

R(f) [GT] = 1.0000

p-inv = 0.3830

gamma shape = 1.3170

Model = TPM2uf

partition = 010212

-lnL = 1987.9786

K = 99

freqA = 0.3116

freqC = 0.1647

freqG = 0.1631

freqT = 0.3606

R(a) [AC] = 0.6546

R(b) [AG] = 0.8082

R(c) [AT] = 0.6546

R(d) [CG] = 1.0000

R(e) [CT] = 0.8082

R(f) [GT] = 1.0000

Model = TPM2uf+I

partition = 010212

-lnL = 1978.3890

K = 100

freqA = 0.3112

freqC = 0.1649

freqG = 0.1649

freqT = 0.3591

R(a) [AC] = 0.6870

R(b) [AG] = 0.8230

R(c) [AT] = 0.6870

R(d) [CG] = 1.0000

R(e) [CT] = 0.8230

R(f) [GT] = 1.0000

p-inv = 0.5470

Model = TPM2uf+G

partition = 010212

-lnL = 1977.1756

K = 100

freqA = 0.3114

freqC = 0.1648

freqG = 0.1647

freqT = 0.3590

R(a) [AC] = 0.6810

R(b) [AG] = 0.8206

R(c) [AT] = 0.6810

R(d) [CG] = 1.0000

R(e) [CT] = 0.8206

R(f) [GT] = 1.0000

gamma shape = 0.5020

Model = TPM2uf+I+G

partition = 010212

-lnL = 1976.8670

K = 101

freqA = 0.3114

freqC = 0.1648

freqG = 0.1648

freqT = 0.3589

R(a) [AC] = 0.6830

R(b) [AG] = 0.8214

R(c) [AT] = 0.6830

R(d) [CG] = 1.0000

R(e) [CT] = 0.8214

R(f) [GT] = 1.0000

p-inv = 0.3470

gamma shape = 1.3990

Model = TPM3

partition = 012012

-lnL = 2039.6691

K = 96

R(a) [AC] = 1.1405

R(b) [AG] = 0.9716

R(c) [AT] = 1.0000

R(d) [CG] = 1.1405

R(e) [CT] = 0.9716

R(f) [GT] = 1.0000

Model = TPM3+I

partition = 012012

-lnL = 2028.4275

K = 97

R(a) [AC] = 1.0557

R(b) [AG] = 0.9391

R(c) [AT] = 1.0000

R(d) [CG] = 1.0557

R(e) [CT] = 0.9391

R(f) [GT] = 1.0000

p-inv = 0.5800

Model = TPM3+G

partition = 012012

-lnL = 2027.3325

K = 97

R(a) [AC] = 1.0598

R(b) [AG] = 0.9406

R(c) [AT] = 1.0000

R(d) [CG] = 1.0598

R(e) [CT] = 0.9406

R(f) [GT] = 1.0000

gamma shape = 0.4260

Model = TPM3+I+G

partition = 012012

-lnL = 2026.9373

K = 98

R(a) [AC] = 1.0550

R(b) [AG] = 0.9386

R(c) [AT] = 1.0000

R(d) [CG] = 1.0550

R(e) [CT] = 0.9386

R(f) [GT] = 1.0000

p-inv = 0.3800

gamma shape = 1.3120

Model = TPM3uf

partition = 012012

-lnL = 1986.4982

K = 99

freqA = 0.3054

freqC = 0.1571

freqG = 0.1687

freqT = 0.3688

R(a) [AC] = 1.7553

R(b) [AG] = 1.2840

R(c) [AT] = 1.0000

R(d) [CG] = 1.7553

R(e) [CT] = 1.2840

R(f) [GT] = 1.0000

Model = TPM3uf+I

partition = 012012

-lnL = 1976.4350

K = 100

freqA = 0.3052

freqC = 0.1564

freqG = 0.1697

freqT = 0.3687

R(a) [AC] = 1.7936

R(b) [AG] = 1.2839

R(c) [AT] = 1.0000

R(d) [CG] = 1.7936

R(e) [CT] = 1.2839

R(f) [GT] = 1.0000

p-inv = 0.5520

Model = TPM3uf+G

partition = 012012

-lnL = 1975.2044

K = 100

freqA = 0.3054

freqC = 0.1561

freqG = 0.1697

freqT = 0.3688

R(a) [AC] = 1.8092

R(b) [AG] = 1.2907

R(c) [AT] = 1.0000

R(d) [CG] = 1.8092

R(e) [CT] = 1.2907

R(f) [GT] = 1.0000

gamma shape = 0.4840

Model = TPM3uf+I+G

partition = 012012

-lnL = 1974.8852

K = 101

freqA = 0.3054

freqC = 0.1561

freqG = 0.1698

freqT = 0.3687

R(a) [AC] = 1.8097

R(b) [AG] = 1.2899

R(c) [AT] = 1.0000

R(d) [CG] = 1.8097

R(e) [CT] = 1.2899

R(f) [GT] = 1.0000

p-inv = 0.3410

gamma shape = 1.3040

Model = TIM1ef

partition = 012230

-lnL = 2022.0630

K = 97

R(a) [AC] = 1.0000

R(b) [AG] = 0.6437

R(c) [AT] = 0.2712

R(d) [CG] = 0.2712

R(e) [CT] = 0.5312

R(f) [GT] = 1.0000

Model = TIM1ef+I

partition = 012230

-lnL = 2010.6759

K = 98

R(a) [AC] = 1.0000

R(b) [AG] = 0.6132

R(c) [AT] = 0.2661

R(d) [CG] = 0.2661

R(e) [CT] = 0.5520

R(f) [GT] = 1.0000

p-inv = 0.5820

Model = TIM1ef+G

partition = 012230

-lnL = 2009.5724

K = 98

R(a) [AC] = 1.0000

R(b) [AG] = 0.6117

R(c) [AT] = 0.2655

R(d) [CG] = 0.2655

R(e) [CT] = 0.5536

R(f) [GT] = 1.0000

gamma shape = 0.4210

Model = TIM1ef+I+G

partition = 012230

-lnL = 2009.1669

K = 99

R(a) [AC] = 1.0000

R(b) [AG] = 0.6097

R(c) [AT] = 0.2651

R(d) [CG] = 0.2651

R(e) [CT] = 0.5550

R(f) [GT] = 1.0000

p-inv = 0.3800

gamma shape = 1.2930

Model = TIM1

partition = 012230

-lnL = 1964.8528

K = 100

freqA = 0.3110

freqC = 0.1611

freqG = 0.1604

freqT = 0.3675

R(a) [AC] = 1.0000

R(b) [AG] = 0.6619

R(c) [AT] = 0.2138

R(d) [CG] = 0.2138

R(e) [CT] = 0.5212

R(f) [GT] = 1.0000

Model = TIM1+I

partition = 012230

-lnL = 1955.6293

K = 101

freqA = 0.3117

freqC = 0.1609

freqG = 0.1617

freqT = 0.3656

R(a) [AC] = 1.0000

R(b) [AG] = 0.6372

R(c) [AT] = 0.2129

R(d) [CG] = 0.2129

R(e) [CT] = 0.5399

R(f) [GT] = 1.0000

p-inv = 0.5360

Model = TIM1+G

partition = 012230

-lnL = 1954.3187

K = 101

freqA = 0.3121

freqC = 0.1606

freqG = 0.1616

freqT = 0.3657

R(a) [AC] = 1.0000

R(b) [AG] = 0.6355

R(c) [AT] = 0.2111

R(d) [CG] = 0.2111

R(e) [CT] = 0.5414

R(f) [GT] = 1.0000

gamma shape = 0.5240

Model = TIM1+I+G

partition = 012230

-lnL = 1954.0317

K = 102

freqA = 0.3121

freqC = 0.1606

freqG = 0.1617

freqT = 0.3656

R(a) [AC] = 1.0000

R(b) [AG] = 0.6340

R(c) [AT] = 0.2110

R(d) [CG] = 0.2110

R(e) [CT] = 0.5424

R(f) [GT] = 1.0000

p-inv = 0.3310

gamma shape = 1.3730

Model = TIM2ef

partition = 010232

-lnL = 2039.4724

K = 97

R(a) [AC] = 0.8829

R(b) [AG] = 0.9442

R(c) [AT] = 0.8829

R(d) [CG] = 1.0000

R(e) [CT] = 0.7785

R(f) [GT] = 1.0000

Model = TIM2ef+I

partition = 010232

-lnL = 2028.3752

K = 98

R(a) [AC] = 0.9528

R(b) [AG] = 0.9406

R(c) [AT] = 0.9528

R(d) [CG] = 1.0000

R(e) [CT] = 0.8461

R(f) [GT] = 1.0000

p-inv = 0.5790

Model = TIM2ef+G

partition = 010232

-lnL = 2027.3147

K = 98

R(a) [AC] = 0.9907

R(b) [AG] = 0.9587

R(c) [AT] = 0.9907

R(d) [CG] = 1.0000

R(e) [CT] = 0.8660

R(f) [GT] = 1.0000

gamma shape = 0.4270

Model = TIM2ef+I+G

partition = 010232

-lnL = 2026.9204

K = 99

R(a) [AC] = 0.9952

R(b) [AG] = 0.9583

R(c) [AT] = 0.9952

R(d) [CG] = 1.0000

R(e) [CT] = 0.8704

R(f) [GT] = 1.0000

p-inv = 0.3780

gamma shape = 1.3040

Model = TIM2

partition = 010232

-lnL = 1987.6479

K = 100

freqA = 0.3098

freqC = 0.1663

freqG = 0.1616

freqT = 0.3623

R(a) [AC] = 0.6533

R(b) [AG] = 0.9123

R(c) [AT] = 0.6533

R(d) [CG] = 1.0000

R(e) [CT] = 0.7132

R(f) [GT] = 1.0000

Model = TIM2+I

partition = 010232

-lnL = 1978.2374

K = 101

freqA = 0.3098

freqC = 0.1661

freqG = 0.1637

freqT = 0.3605

R(a) [AC] = 0.6844

R(b) [AG] = 0.8958

R(c) [AT] = 0.6844

R(d) [CG] = 1.0000

R(e) [CT] = 0.7498

R(f) [GT] = 1.0000

p-inv = 0.5440

Model = TIM2+G

partition = 010232

-lnL = 1977.0270

K = 101

freqA = 0.3101

freqC = 0.1660

freqG = 0.1635

freqT = 0.3604

R(a) [AC] = 0.6782

R(b) [AG] = 0.8914

R(c) [AT] = 0.6782

R(d) [CG] = 1.0000

R(e) [CT] = 0.7482

R(f) [GT] = 1.0000

gamma shape = 0.5100

Model = TIM2+I+G

partition = 010232

-lnL = 1976.7256

K = 102

freqA = 0.3101

freqC = 0.1660

freqG = 0.1636

freqT = 0.3603

R(a) [AC] = 0.6801

R(b) [AG] = 0.8900

R(c) [AT] = 0.6801

R(d) [CG] = 1.0000

R(e) [CT] = 0.7507

R(f) [GT] = 1.0000

p-inv = 0.3430

gamma shape = 1.4070

Model = TIM3ef

partition = 012032

-lnL = 2039.4543

K = 97

R(a) [AC] = 1.1402

R(b) [AG] = 1.0681

R(c) [AT] = 1.0000

R(d) [CG] = 1.1402

R(e) [CT] = 0.8810

R(f) [GT] = 1.0000

Model = TIM3ef+I

partition = 012032

-lnL = 2028.3659

K = 98

R(a) [AC] = 1.0582

R(b) [AG] = 0.9908

R(c) [AT] = 1.0000

R(d) [CG] = 1.0582

R(e) [CT] = 0.8882

R(f) [GT] = 1.0000

p-inv = 0.5780

Model = TIM3ef+G

partition = 012032

-lnL = 2027.2755

K = 98

R(a) [AC] = 1.0621

R(b) [AG] = 0.9900

R(c) [AT] = 1.0000

R(d) [CG] = 1.0621

R(e) [CT] = 0.8918

R(f) [GT] = 1.0000

gamma shape = 0.4300

Model = TIM3ef+I+G

partition = 012032

-lnL = 2026.8867

K = 99

R(a) [AC] = 1.0574

R(b) [AG] = 0.9854

R(c) [AT] = 1.0000

R(d) [CG] = 1.0574

R(e) [CT] = 0.8926

R(f) [GT] = 1.0000

p-inv = 0.3760

gamma shape = 1.3090

Model = TIM3

partition = 012032

-lnL = 1986.2188

K = 100

freqA = 0.3039

freqC = 0.1586

freqG = 0.1672

freqT = 0.3704

R(a) [AC] = 1.7509

R(b) [AG] = 1.4353

R(c) [AT] = 1.0000

R(d) [CG] = 1.7509

R(e) [CT] = 1.1443

R(f) [GT] = 1.0000

Model = TIM3+I

partition = 012032

-lnL = 1976.3496

K = 101

freqA = 0.3041

freqC = 0.1574

freqG = 0.1689

freqT = 0.3696

R(a) [AC] = 1.7853

R(b) [AG] = 1.3671

R(c) [AT] = 1.0000

R(d) [CG] = 1.7853

R(e) [CT] = 1.1956

R(f) [GT] = 1.0000

p-inv = 0.5500

Model = TIM3+G

partition = 012032

-lnL = 1975.1321

K = 101

freqA = 0.3044

freqC = 0.1571

freqG = 0.1689

freqT = 0.3697

R(a) [AC] = 1.8013

R(b) [AG] = 1.3673

R(c) [AT] = 1.0000

R(d) [CG] = 1.8013

R(e) [CT] = 1.2091

R(f) [GT] = 1.0000

gamma shape = 0.4900

Model = TIM3+I+G

partition = 012032

-lnL = 1974.8205

K = 102

freqA = 0.3044

freqC = 0.1570

freqG = 0.1690

freqT = 0.3695

R(a) [AC] = 1.8013

R(b) [AG] = 1.3627

R(c) [AT] = 1.0000

R(d) [CG] = 1.8013

R(e) [CT] = 1.2113

R(f) [GT] = 1.0000

p-inv = 0.3500

gamma shape = 1.3770

Model = TVMef

partition = 012314

-lnL = 2021.6802

K = 98

R(a) [AC] = 1.0759

R(b) [AG] = 0.6066

R(c) [AT] = 0.3242

R(d) [CG] = 0.1940

R(e) [CT] = 0.6066

R(f) [GT] = 1.0000

Model = TVMef+I

partition = 012314

-lnL = 2009.6551

K = 99

R(a) [AC] = 1.0864

R(b) [AG] = 0.6063

R(c) [AT] = 0.3436

R(d) [CG] = 0.1679

R(e) [CT] = 0.6063

R(f) [GT] = 1.0000

p-inv = 0.5920

Model = TVMef+G

partition = 012314

-lnL = 2008.5714

K = 99

R(a) [AC] = 1.0873

R(b) [AG] = 0.6066

R(c) [AT] = 0.3425

R(d) [CG] = 0.1684

R(e) [CT] = 0.6066

R(f) [GT] = 1.0000

gamma shape = 0.3980

Model = TVMef+I+G

partition = 012314

-lnL = 2008.1339

K = 100

R(a) [AC] = 1.0878

R(b) [AG] = 0.6064

R(c) [AT] = 0.3435

R(d) [CG] = 0.1669

R(e) [CT] = 0.6064

R(f) [GT] = 1.0000

p-inv = 0.4110

gamma shape = 1.3860

Model = TVM

partition = 012314

-lnL = 1964.8159

K = 101

freqA = 0.3112

freqC = 0.1573

freqG = 0.1632

freqT = 0.3684

R(a) [AC] = 1.1829

R(b) [AG] = 0.6367

R(c) [AT] = 0.2202

R(d) [CG] = 0.2782

R(e) [CT] = 0.6367

R(f) [GT] = 1.0000

Model = TVM+I

partition = 012314

-lnL = 1955.2524

K = 102

freqA = 0.3109

freqC = 0.1566

freqG = 0.1646

freqT = 0.3679

R(a) [AC] = 1.2690

R(b) [AG] = 0.6578

R(c) [AT] = 0.2268

R(d) [CG] = 0.2803

R(e) [CT] = 0.6578

R(f) [GT] = 1.0000

p-inv = 0.5410

Model = TVM+G

partition = 012314

-lnL = 1953.9252

K = 102

freqA = 0.3112

freqC = 0.1563

freqG = 0.1644

freqT = 0.3680

R(a) [AC] = 1.2717

R(b) [AG] = 0.6587

R(c) [AT] = 0.2243

R(d) [CG] = 0.2822

R(e) [CT] = 0.6587

R(f) [GT] = 1.0000

gamma shape = 0.5080

Model = TVM+I+G

partition = 012314

-lnL = 1953.6242

K = 103

freqA = 0.3112

freqC = 0.1563

freqG = 0.1645

freqT = 0.3680

R(a) [AC] = 1.2765

R(b) [AG] = 0.6597

R(c) [AT] = 0.2245

R(d) [CG] = 0.2820

R(e) [CT] = 0.6597

R(f) [GT] = 1.0000

p-inv = 0.3260

gamma shape = 1.2870

Model = SYM

partition = 012345

-lnL = 2021.4658

K = 99

R(a) [AC] = 1.0755

R(b) [AG] = 0.6665

R(c) [AT] = 0.3244

R(d) [CG] = 0.1939

R(e) [CT] = 0.5501

R(f) [GT] = 1.0000

Model = SYM+I

partition = 012345

-lnL = 2009.5984

K = 100

R(a) [AC] = 1.0892

R(b) [AG] = 0.6383

R(c) [AT] = 0.3440

R(d) [CG] = 0.1682

R(e) [CT] = 0.5747

R(f) [GT] = 1.0000

p-inv = 0.5900

Model = SYM+G

partition = 012345

-lnL = 2008.5193

K = 100

R(a) [AC] = 1.0901

R(b) [AG] = 0.6371

R(c) [AT] = 0.3427

R(d) [CG] = 0.1689

R(e) [CT] = 0.5767

R(f) [GT] = 1.0000

gamma shape = 0.4020

Model = SYM+I+G

partition = 012345

-lnL = 2008.0865

K = 101

R(a) [AC] = 1.0908

R(b) [AG] = 0.6350

R(c) [AT] = 0.3438

R(d) [CG] = 0.1673

R(e) [CT] = 0.5785

R(f) [GT] = 1.0000

p-inv = 0.3880

gamma shape = 1.2610

Model = GTR

partition = 012345

-lnL = 1964.5188

K = 102

freqA = 0.3096

freqC = 0.1587

freqG = 0.1617

freqT = 0.3700

R(a) [AC] = 1.1787

R(b) [AG] = 0.7134

R(c) [AT] = 0.2197

R(d) [CG] = 0.2772

R(e) [CT] = 0.5652

R(f) [GT] = 1.0000

Model = GTR+I

partition = 012345

-lnL = 1955.1488

K = 103

freqA = 0.3098

freqC = 0.1577

freqG = 0.1636

freqT = 0.3690

R(a) [AC] = 1.2595

R(b) [AG] = 0.7038

R(c) [AT] = 0.2260

R(d) [CG] = 0.2789

R(e) [CT] = 0.6077

R(f) [GT] = 1.0000

p-inv = 0.5380

Model = GTR+G

partition = 012345

-lnL = 1953.8321

K = 103

freqA = 0.3102

freqC = 0.1574

freqG = 0.1635

freqT = 0.3690

R(a) [AC] = 1.2606

R(b) [AG] = 0.7015

R(c) [AT] = 0.2235

R(d) [CG] = 0.2805

R(e) [CT] = 0.6106

R(f) [GT] = 1.0000

gamma shape = 0.5150

Model = GTR+I+G

partition = 012345

-lnL = 1953.5379

K = 104

freqA = 0.3102

freqC = 0.1573

freqG = 0.1636

freqT = 0.3690

R(a) [AC] = 1.2646

R(b) [AG] = 0.7008

R(c) [AT] = 0.2237

R(d) [CG] = 0.2805

R(e) [CT] = 0.6128

R(f) [GT] = 1.0000

p-inv = 0.3150

gamma shape = 1.2580

Best-fit models should be reoptimized for comparison with unconstrained likelihood

Computation of likelihood scores completed. It took 00h:00:48:00.

---------------------------------------------------------------

* *

* AKAIKE INFORMATION CRITERION (AIC) *

* *

---------------------------------------------------------------

Model selected:

Model = TPM1uf+G

partition = 012210

-lnL = 1954.4443

K = 100

freqA = 0.3134

freqC = 0.1596

freqG = 0.1626

freqT = 0.3644

R(a) [AC] = 1.0000

R(b) [AG] = 0.5881

R(c) [AT] = 0.2111

R(d) [CG] = 0.2111

R(e) [CT] = 0.5881

R(f) [GT] = 1.0000

gamma shape = 0.5160

--

PAUP* Commands Block:

If you want to load the selected model and associated estimates in PAUP*,

attach the next block of commands after the data in your PAUP file:

[!

Likelihood settings from best-fit model (TPM1uf+G) selected by AIC

with jModeltest 2.1.10 v20160303 on Thu Dec 29 23:00:58 PST 2022]

BEGIN PAUP;

Lset base=(0.3134 0.1596 0.1626 ) nst=6 rmat=(1.0000 0.5881 0.2111 0.2111 0.5881) rates=gamma shape=0.5160 ncat=4 pinvar=0;

END;

--

Tree for the best AIC model = (Ae_lepidioides:0.03121337,HM95_spinosum_S592:0.00510022,((Ae_eunomioides:0.00317643,((Ae_karamanicum:0.00000008,((Ae_diastrophis:0.00000008,(Ae_umbellatum:0.00311010,(Ae_coridifolium:0.00000001,Ae_armenum:0.00154992):0.00000001):0.00785265):0.00155695,(Ae_alanyae:0.00000011,(Ae_schistosum:0.00313491,(Ae_demirizii:0.00000001,(DQ180216_elongatum:0.00000001,Ae_glaucinum:0.00000007):0.00000038):0.00832859):0.00000061):0.00000008):0.00164657):0.00503741,(((HM100_grandiflorum_S574:0.00313588,(Ae_huber_morathii:0.00156710,Ae_capitatum:0.00197456):0.00000008):0.00163355,(HM326_erinaceum_S572:0.00670476,Ae_spicatum:0.00628050):0.00000015):0.00828058,(HM86_transhyrcanum_S579:0.00000007,HM104_membranaceum_S573:0.00155300):0.00332673):0.00523236):0.00164100):0.00493522,(Ae_lycium:0.00156649,(Ae_turcica:0.00473694,((Ae_thesiifolium:0.00156565,(Ae_dumanii:0.00646413,(Ae_stenopterum:0.00315615,(Ae_arabicum:0.00000007,(Ae_heterocarpum:0.00314952,((Ae_froedinii:0.00000001,Ae_syriacum:0.00000001):0.00157013,Ae_carneum:0.00000006):0.00000006):0.00000007):0.00313606):0.00313359):0.00155965):0.00000006,(Ae_cordatum:0.00630882,(((Umbellatum_Archibold_J1:0.00000001,HM1454_Oshrurankuh_S1857:0.00159034):0.00641780,(W_0184833_Aethionema_trnT-trnL-trnF:0.00000001,(HM1452_yazd_S1855:0.00000001,_Shirkuh_J2_trnL:0.00000011):0.00000001):0.00234077):0.00231182,((Ae_orbiculatum:0.00000008,((Ae_acarii:0.00887534,Ae_saxatile:0.00000001):0.00362566,(Ae_fimbriatum:0.00815161,(Ae_stylosum:0.00480922,(Ae_speciosum:0.00315304,(Ae_papillosum:0.00157688,Ae_munzurense:0.00315658):0.00000008):0.00315773):0.00000008):0.00157443):0.00157476):0.00157161,(HM480_spec_nova_S647:0.00000008,(HM479_spec._nova_S646:0.00000009,(HM478_spec_nova_S645:0.00157488,HM482_spec_nova_S648:0.00000003):0.00000003):0.00000006):0.00157922):0.00000006):0.00471396):0.00000011):0.00000009):0.00000012):0.01174593):0.01396866);

* AIC MODEL SELECTION : Selection uncertainty

Model -lnL K AIC delta weight cumWeight

-------------------------------------------------------------------------

TPM1uf+G 1954.44432 100 4108.888640 0.000000 0.327052 0.327052

TPM1uf+I+G 1954.15119 101 4110.302380 1.413740 0.161297 0.488349

TIM1+G 1954.31869 101 4110.637380 1.748740 0.136421 0.624770

TPM1uf+I 1955.76233 100 4111.524660 2.636020 0.087541 0.712311

TVM+G 1953.92519 102 4111.850380 2.961740 0.074385 0.786696

TIM1+I+G 1954.03172 102 4112.063440 3.174800 0.066868 0.853564

TVM+I+G 1953.62423 103 4113.248460 4.359820 0.036974 0.890537

TIM1+I 1955.62933 101 4113.258660 4.370020 0.036786 0.927323

GTR+G 1953.83207 103 4113.664140 4.775500 0.030035 0.957358

TVM+I 1955.25236 102 4114.504720 5.616080 0.019729 0.977087

GTR+I+G 1953.53790 104 4115.075800 6.187160 0.014828 0.991915

GTR+I 1955.14881 103 4116.297620 7.408980 0.008050 0.999965

TPM1uf 1965.16426 99 4128.328520 19.439880 1.96e-005 0.999984

TIM1 1964.85284 100 4129.705680 20.817040 9.87e-006 0.999994

TVM 1964.81588 101 4131.631760 22.743120 3.77e-006 0.999998

GTR 1964.51881 102 4133.037620 24.148980 1.87e-006 1.000000

TPM3uf+G 1975.20442 100 4150.408840 41.520200 3.15e-010 1.000000

TPM3uf+I+G 1974.88523 101 4151.770460 42.881820 1.60e-010 1.000000

TIM3+G 1975.13208 101 4152.264160 43.375520 1.25e-010 1.000000

TPM3uf+I 1976.43500 100 4152.870000 43.981360 9.21e-011 1.000000

F81+G 1978.69742 98 4153.394840 44.506200 7.08e-011 1.000000

TIM3+I+G 1974.82046 102 4153.640920 44.752280 6.26e-011 1.000000

TPM2uf+G 1977.17561 100 4154.351220 45.462580 4.39e-011 1.000000

TIM3+I 1976.34961 101 4154.699220 45.810580 3.69e-011 1.000000

F81+I+G 1978.36107 99 4154.722140 45.833500 3.65e-011 1.000000

HKY+G 1978.68979 99 4155.379580 46.490940 2.63e-011 1.000000

F81+I 1979.84385 98 4155.687700 46.799060 2.25e-011 1.000000

TPM2uf+I+G 1976.86696 101 4155.733920 46.845280 2.20e-011 1.000000

TIM2+G 1977.02700 101 4156.054000 47.165360 1.87e-011 1.000000

HKY+I+G 1978.35406 100 4156.708120 47.819480 1.35e-011 1.000000

TPM2uf+I 1978.38903 100 4156.778060 47.889420 1.30e-011 1.000000

TrN+G 1978.57075 100 4157.141500 48.252860 1.09e-011 1.000000

TIM2+I+G 1976.72556 102 4157.451120 48.562480 9.32e-012 1.000000

HKY+I 1979.83768 99 4157.675360 48.786720 8.33e-012 1.000000

TIM2+I 1978.23742 101 4158.474840 49.586200 5.59e-012 1.000000

TrN+I+G 1978.24275 101 4158.485500 49.596860 5.56e-012 1.000000

TrN+I 1979.71054 100 4159.421080 50.532440 3.48e-012 1.000000

TPM3uf 1986.49823 99 4170.996460 62.107820 1.07e-014 1.000000

TIM3 1986.21875 100 4172.437500 63.548860 5.19e-015 1.000000

TPM2uf 1987.97857 99 4173.957140 65.068500 2.43e-015 1.000000

F81 1990.06503 97 4174.130060 65.241420 2.23e-015 1.000000

TIM2 1987.64789 100 4175.295780 66.407140 1.24e-015 1.000000

HKY 1990.04277 98 4176.085540 67.196900 8.38e-016 1.000000

TrN 1989.73049 99 4177.460980 68.572340 4.21e-016 1.000000

TPM1+G 2009.62507 97 4213.250140 104.361500 7.13e-024 1.000000

TPM1+I+G 2009.21342 98 4214.426840 105.538200 3.96e-024 1.000000

TVMef+G 2008.57144 99 4215.142880 106.254240 2.77e-024 1.000000

TIM1ef+G 2009.57238 98 4215.144760 106.256120 2.76e-024 1.000000

TPM1+I 2010.73276 97 4215.465520 106.576880 2.35e-024 1.000000

TVMef+I+G 2008.13387 100 4216.267740 107.379100 1.58e-024 1.000000

TIM1ef+I+G 2009.16693 99 4216.333860 107.445220 1.52e-024 1.000000

SYM+G 2008.51934 100 4217.038680 108.150040 1.07e-024 1.000000

TVMef+I 2009.65512 99 4217.310240 108.421600 9.36e-025 1.000000

TIM1ef+I 2010.67594 98 4217.351880 108.463240 9.16e-025 1.000000

SYM+I+G 2008.08649 101 4218.172980 109.284340 6.08e-025 1.000000

SYM+I 2009.59843 100 4219.196860 110.308220 3.64e-025 1.000000

TPM1 2022.27793 96 4236.555860 127.667220 6.19e-029 1.000000

TIM1ef 2022.06300 97 4238.126000 129.237360 2.83e-029 1.000000

TVMef 2021.68025 98 4239.360500 130.471860 1.52e-029 1.000000

SYM 2021.46580 99 4240.931600 132.042960 6.95e-030 1.000000

JC+G 2027.48582 95 4244.971640 136.083000 9.22e-031 1.000000

JC+I+G 2027.08664 96 4246.173280 137.284640 5.05e-031 1.000000

K80+G 2027.36975 96 4246.739500 137.850860 3.81e-031 1.000000

JC+I 2028.57639 95 4247.152780 138.264140 3.10e-031 1.000000

K80+I+G 2026.97015 97 4247.940300 139.051660 2.09e-031 1.000000

TrNef+G 2027.31565 97 4248.631300 139.742660 1.48e-031 1.000000

TPM3+G 2027.33247 97 4248.664940 139.776300 1.45e-031 1.000000

TPM2+G 2027.36867 97 4248.737340 139.848700 1.40e-031 1.000000

K80+I 2028.46030 96 4248.920600 140.031960 1.28e-031 1.000000

TrNef+I+G 2026.92131 98 4249.842620 140.953980 8.07e-032 1.000000

TPM3+I+G 2026.93735 98 4249.874700 140.986060 7.94e-032 1.000000

TPM2+I+G 2026.96837 98 4249.936740 141.048100 7.70e-032 1.000000

TIM3ef+G 2027.27553 98 4250.551060 141.662420 5.66e-032 1.000000

TIM2ef+G 2027.31470 98 4250.629400 141.740760 5.45e-032 1.000000

TrNef+I 2028.40172 97 4250.803440 141.914800 4.99e-032 1.000000

TPM3+I 2028.42747 97 4250.854940 141.966300 4.86e-032 1.000000

TPM2+I 2028.43296 97 4250.865920 141.977280 4.84e-032 1.000000

TIM3ef+I+G 2026.88670 99 4251.773400 142.884760 3.07e-032 1.000000

TIM2ef+I+G 2026.92040 99 4251.840800 142.952160 2.97e-032 1.000000

TIM3ef+I 2028.36586 98 4252.731720 143.843080 1.90e-032 1.000000

TIM2ef+I 2028.37515 98 4252.750300 143.861660 1.89e-032 1.000000

JC 2039.99294 94 4267.985880 159.097240 9.27e-036 1.000000

K80 2039.88158 95 4269.763160 160.874520 3.81e-036 1.000000

TrNef 2039.66627 96 4271.332540 162.443900 1.74e-036 1.000000

TPM3 2039.66915 96 4271.338300 162.449660 1.73e-036 1.000000

TPM2 2039.68826 96 4271.376520 162.487880 1.70e-036 1.000000

TIM3ef 2039.45433 97 4272.908660 164.020020 7.91e-037 1.000000

TIM2ef 2039.47245 97 4272.944900 164.056260 7.77e-037 1.000000

-------------------------------------------------------------------------

-lnL: negative log likelihod

K: number of estimated parameters

AIC: Akaike Information Criterion

delta: AIC difference

weight: AIC weight

cumWeight: cumulative AIC weight

* AIC MODEL SELECTION : Confidence interval

There are 88 models in the 100% confidence interval: [ TPM1uf+G TPM1uf+I+G TIM1+G TPM1uf+I TVM+G TIM1+I+G TVM+I+G TIM1+I GTR+G TVM+I GTR+I+G GTR+I TPM1uf TIM1 TVM GTR TPM3uf+G TPM3uf+I+G TIM3+G TPM3uf+I F81+G TIM3+I+G TPM2uf+G TIM3+I F81+I+G HKY+G F81+I TPM2uf+I+G TIM2+G HKY+I+G TPM2uf+I TrN+G TIM2+I+G HKY+I TIM2+I TrN+I+G TrN+I TPM3uf TIM3 TPM2uf F81 TIM2 HKY TrN TPM1+G TPM1+I+G TVMef+G TIM1ef+G TPM1+I TVMef+I+G TIM1ef+I+G SYM+G TVMef+I TIM1ef+I SYM+I+G SYM+I TPM1 TIM1ef TVMef SYM JC+G JC+I+G K80+G JC+I K80+I+G TrNef+G TPM3+G TPM2+G K80+I TrNef+I+G TPM3+I+G TPM2+I+G TIM3ef+G TIM2ef+G TrNef+I TPM3+I TPM2+I TIM3ef+I+G TIM2ef+I+G TIM3ef+I TIM2ef+I JC K80 TrNef TPM3 TPM2 TIM3ef TIM2ef ]

* AIC MODEL SELECTION : Parameter importance

Parameter Importance

----------------------

fA 1.0000

fC 1.0000

fG 1.0000

fT 1.0000

kappa 0.0000

titv 0.0000

rAC 0.1840

rAG 1.0000

rAT 1.0000

rCG 1.0000

rCT 1.0000

rGT 1.0000

pinv(I) 0.1521

alpha(G) 0.5679

pinv(IG) 0.2800

alpha(IG) 0.2800

----------------------

Values have been rounded.

(I): considers only +I models.

(G): considers only +G models.

(IG): considers only +I+G models.

* AIC MODEL SELECTION : Best Model's command line

phyml -i /tmp/jmodeltest13390012638257739752.phy -d nt -n 1 -b 0 --run_id TPM1uf+G -m 012210 -f m -c 4 -a e --no_memory_check -o tlr -s BEST

---------------------------------------------------------------

* *

* MODEL AVERAGED PHYLOGENY *

* *

---------------------------------------------------------------

Selection criterion: . . . . AIC

Confidence interval: . . . . 1.00

Consensus type:. . . . . . . 50% majority rule

Using 88 models in the 1.00 confidence interval = TPM1uf+G TPM1uf+I+G TIM1+G TPM1uf+I TVM+G TIM1+I+G TVM+I+G TIM1+I GTR+G TVM+I GTR+I+G GTR+I TPM1uf TIM1 TVM GTR TPM3uf+G TPM3uf+I+G TIM3+G TPM3uf+I F81+G TIM3+I+G TPM2uf+G TIM3+I F81+I+G HKY+G F81+I TPM2uf+I+G TIM2+G HKY+I+G TPM2uf+I TrN+G TIM2+I+G HKY+I TIM2+I TrN+I+G TrN+I TPM3uf TIM3 TPM2uf F81 TIM2 HKY TrN TPM1+G TPM1+I+G TVMef+G TIM1ef+G TPM1+I TVMef+I+G TIM1ef+I+G SYM+G TVMef+I TIM1ef+I SYM+I+G SYM+I TPM1 TIM1ef TVMef SYM JC+G JC+I+G K80+G JC+I K80+I+G TrNef+G TPM3+G TPM2+G K80+I TrNef+I+G TPM3+I+G TPM2+I+G TIM3ef+G TIM2ef+G TrNef+I TPM3+I TPM2+I TIM3ef+I+G TIM2ef+I+G TIM3ef+I TIM2ef+I JC K80 TrNef TPM3 TPM2 TIM3ef TIM2ef

Species in order:

1. Ae_lepidioides

2. HM95_spinosum_S592

3. Ae_eunomioides

4. Ae_karamanicum

5. Ae_diastrophis

6. Ae_umbellatum

7. Ae_coridifolium

8. Ae_armenum

9. Ae_alanyae

10. Ae_schistosum

11. Ae_demirizii

12. DQ180216_elongatum

13. Ae_glaucinum

14. HM100_grandiflorum_S574

15. Ae_huber_morathii

16. Ae_capitatum

17. HM326_erinaceum_S572

18. Ae_spicatum

19. HM86_transhyrcanum_S579

20. HM104_membranaceum_S573

21. Ae_lycium

22. Ae_turcica

23. Ae_thesiifolium

24. Ae_dumanii

25. Ae_stenopterum

26. Ae_arabicum

27. Ae_heterocarpum

28. Ae_froedinii

29. Ae_syriacum

30. Ae_carneum

31. Ae_cordatum

32. Umbellatum_Archibold_J1

33. HM1454_Oshrurankuh_S1857

34. W_0184833_Aethionema_trnT-trnL-trnF

35. HM1452_yazd_S1855

36. _Shirkuh_J2_trnL

37. Ae_orbiculatum

38. Ae_acarii

39. Ae_saxatile

40. Ae_fimbriatum

41. Ae_stylosum

42. Ae_speciosum

43. Ae_papillosum

44. Ae_munzurense

45. HM480_spec_nova_S647

46. HM479_spec._nova_S646

47. HM478_spec_nova_S645

48. HM482_spec_nova_S648

Bipartitions included in the consensus tree

123456789111111111122222222223333333333444444444

012345678901234567890123456789012345678

************************************************ ( 1.0 )

--********************************************** ( 1.0 )

--******************---------------------------- ( 1.0 )

---*****************---------------------------- ( 1.0 )

---**********----------------------------------- ( 1.0 )

----*********----------------------------------- ( 1.0 )

----****---------------------------------------- ( 1.0 )

-----***---------------------------------------- ( 1.0 )

------**---------------------------------------- ( 0.56793 )

--------*****----------------------------------- ( 0.56793 )

---------****----------------------------------- ( 0.56793 )

----------***----------------------------------- ( 1.0 )

-----------**----------------------------------- ( 0.86763 )

-------------*******---------------------------- ( 1.0 )

-------------*****------------------------------ ( 1.0 )

-------------***-------------------------------- ( 1.0 )

--------------**-------------------------------- ( 0.8479 )

----------------**------------------------------ ( 0.61273 )

------------------**---------------------------- ( 1.0 )

--------------------**************************** ( 1.0 )

-----------------------*******------------------ ( 1.0 )

------------------------******------------------ ( 1.0 )

-------------------------*****------------------ ( 1.0 )

--------------------------****------------------ ( 0.72004 )

---------------------------***------------------ ( 0.56793 )

---------------------------**------------------- ( 1.0 )

-------------------------------***************** ( 1.0 )

-------------------------------*************---- ( 0.53175 )

-------------------------------*****------------ ( 1.0 )

-------------------------------**--------------- ( 1.0 )

---------------------------------***------------ ( 1.0 )

----------------------------------**------------ ( 1.0 )

------------------------------------********---- ( 1.0 )

-------------------------------------*******---- ( 1.0 )

-------------------------------------**--------- ( 1.0 )

---------------------------------------*****---- ( 1.0 )

----------------------------------------****---- ( 0.72004 )

-----------------------------------------***---- ( 1.0 )

------------------------------------------**---- ( 0.68325 )

--------------------------------------------**** ( 1.0 )

---------------------------------------------*** ( 0.56793 )

+-48 Ae_glaucinum

+-80

| +-47 DQ180216_elongatum

+-----81

| +-46 Ae_demirizii

+-82

| +-45 Ae_schistosum

+-83

| +-44 Ae_alanyae

+-84

| | +-43 Ae_armenum

| | +-77

| | | +-42 Ae_coridifolium

| | +----78

| | | +-41 Ae_umbellatum

| +-79

| +-40 Ae_diastrophis

+--85

| +-39 Ae_karamanicum

+-86

| | +-38 HM104_membranaceum_S573

| | +-75

| | | +-37 HM86_transhyrcanum_S579

| +--76

| | +---36 Ae_spicatum

| | +-73

| | | +---35 HM326_erinaceum_S572

| +-----74

| | +-34 Ae_capitatum

| | +-71

| | | +-33 Ae_huber_morathii

| +-72

| +-32 HM100_grandiflorum_S574

+--87

| +-31 Ae_eunomioides

+----------88

| | +-30 _Shirkuh_J2_trnL

| | +-65

| | | +-29 HM1452_yazd_S1855

| | +-66

| | | +-28 W_0184833_Aethionema_trnT-trnL-trnF

| | +-67

| | | | +-27 HM1454_Oshrurankuh_S1857

| | | +---64

| | | +-26 Umbellatum_Archibold_J1

| | +-68

| | | | +-25 Ae_munzurense

| | | | +-58

| | | | | +-24 Ae_papillosum

| | | | +-59

| | | | | +-23 Ae_speciosum

| | | | +-60

| | | | | +--22 Ae_stylosum

| | | | +-61

| | | | | +-----21 Ae_fimbriatum

| | | | +-62

| | | | | | +-20 Ae_saxatile

| | | | | +-57

| | | | | +-----19 Ae_acarii

| | | +-63

| | | +-18 Ae_orbiculatum

| | +--69

| | | | +-17 HM482_spec_nova_S648

| | | | |

| | | | +-16 HM478_spec_nova_S645

| | | | +-55

| | | | | +-15 HM479_spec._nova_S646

| | | +-56

| | | +-14 HM480_spec_nova_S647

| | |

| | | +-13 Ae_syriacum

| | | +-49

| | | | +-12 Ae_froedinii

| | | +-50

| | | | +-11 Ae_carneum

| | | +-51

| | | | +-10 Ae_heterocarpum

| | | +-52

| | | | +--9 Ae_arabicum

| | | +-53

| | | | +--8 Ae_stenopterum

| | +-54

| | | +----7 Ae_dumanii

| | |

| | +----6 Ae_cordatum

| +--------70

| +--5 Ae_thesiifolium

| |

| +---4 Ae_turcica

| |

| +--3 Ae_lycium

|

+---2 HM95_spinosum_S592

|

+--------------------------1 Ae_lepidioides

(Ae_lepidioides:0.0312133700,HM95_spinosum_S592:0.0050993700,((Ae_lycium:0.0015661800,Ae_turcica:0.0047349000,Ae_thesiifolium:0.0015652400,Ae_cordatum:0.0063088200,(Ae_dumanii:0.0064641300,(Ae_stenopterum:0.0031560200,(Ae_arabicum:0.0000000700,(Ae_heterocarpum:0.0031495200,(Ae_carneum:0.0000000600,(Ae_froedinii:0.0000000100,Ae_syriacum:0.0000000100):1.0000:0.0015701300):0.5679:0.0000000600):0.7200:0.0000000700):1.0000:0.0031360600):1.0000:0.0031335900):1.0000:0.0015588300,((HM480_spec_nova_S647:0.0000000700,(HM479_spec._nova_S646:0.0000000900,HM478_spec_nova_S645:0.0015748400,HM482_spec_nova_S648:0.0000000500):0.5679:0.0000000600):1.0000:0.0015791700,((Ae_orbiculatum:0.0000000800,((Ae_acarii:0.0088720300,Ae_saxatile:0.0000000100):1.0000:0.0036256600,(Ae_fimbriatum:0.0081516100,(Ae_stylosum:0.0048092200,(Ae_speciosum:0.0031530400,(Ae_papillosum:0.0015768800,Ae_munzurense:0.0031565800):0.6832:0.0000000800):1.0000:0.0031577300):0.7200:0.0000000800):1.0000:0.0015744300):1.0000:0.0015747600):1.0000:0.0015716100,((Umbellatum_Archibold_J1:0.0000000100,HM1454_Oshrurankuh_S1857:0.0015889300):1.0000:0.0064178000,(W_0184833_Aethionema_trnT-trnL-trnF:0.0000000100,(HM1452_yazd_S1855:0.0000000100,_Shirkuh_J2_trnL:0.0000001100):1.0000:0.0000000100):1.0000:0.0023404000):1.0000:0.0023117100):0.5317:0.0000000600):1.0000:0.0047139600):1.0000:0.0117459300,(Ae_eunomioides:0.0031764300,((((HM100_grandiflorum_S574:0.0031356200,(Ae_huber_morathii:0.0015668500,Ae_capitatum:0.0019745600):0.8479:0.0000000800):1.0000:0.0016326200,(HM326_erinaceum_S572:0.0067018300,Ae_spicatum:0.0062796200):0.6127:0.0000001500):1.0000:0.0082805800,(HM86_transhyrcanum_S579:0.0000000700,HM104_membranaceum_S573:0.0015530000):1.0000:0.0033267300):1.0000:0.0052323600,(Ae_karamanicum:0.0000000800,((Ae_diastrophis:0.0000000800,(Ae_umbellatum:0.0031101000,(Ae_coridifolium:0.0000000100,Ae_armenum:0.0015497500):0.5679:0.0000000100):1.0000:0.0078526500):1.0000:0.0015569500,(Ae_alanyae:0.0000001100,(Ae_schistosum:0.0031349100,(Ae_demirizii:0.0000000100,(DQ180216_elongatum:0.0000000100,Ae_glaucinum:0.0000000700):0.8676:0.0000003800):1.0000:0.0083285900):0.5679:0.0000006100):0.5679:0.0000000800):1.0000:0.0016449200):1.0000:0.0050374100):1.0000:0.0016405700):1.0000:0.0049263800):1.0000:0.0139686600);

Note: this tree is unrooted. Branch lengths are the expected number of substitutions per site. Labels next to parentheses represent phylogenetic uncertainty due to model selection (see documentation)

---------------------------------------------------------------

* *

* CORRECTED AKAIKE INFORMATION CRITERION (AICc) *

* *

---------------------------------------------------------------

Sample size: 699.0

Model selected:

Model = TPM1uf+G

partition = 012210

-lnL = 1954.4443

K = 100

freqA = 0.3134

freqC = 0.1596

freqG = 0.1626

freqT = 0.3644

R(a) [AC] = 1.0000

R(b) [AG] = 0.5881

R(c) [AT] = 0.2111

R(d) [CG] = 0.2111

R(e) [CT] = 0.5881

R(f) [GT] = 1.0000

gamma shape = 0.5160

--

PAUP* Commands Block:

If you want to load the selected model and associated estimates in PAUP*,

attach the next block of commands after the data in your PAUP file:

[!

Likelihood settings from best-fit model (TPM1uf+G) selected by AICc

with jModeltest 2.1.10 v20160303 on Thu Dec 29 23:00:58 PST 2022]

BEGIN PAUP;

Lset base=(0.3134 0.1596 0.1626 ) nst=6 rmat=(1.0000 0.5881 0.2111 0.2111 0.5881) rates=gamma shape=0.5160 ncat=4 pinvar=0;

END;

--

Tree for the best AICc model = (Ae_lepidioides:0.03121337,HM95_spinosum_S592:0.00510022,((Ae_eunomioides:0.00317643,((Ae_karamanicum:0.00000008,((Ae_diastrophis:0.00000008,(Ae_umbellatum:0.00311010,(Ae_coridifolium:0.00000001,Ae_armenum:0.00154992):0.00000001):0.00785265):0.00155695,(Ae_alanyae:0.00000011,(Ae_schistosum:0.00313491,(Ae_demirizii:0.00000001,(DQ180216_elongatum:0.00000001,Ae_glaucinum:0.00000007):0.00000038):0.00832859):0.00000061):0.00000008):0.00164657):0.00503741,(((HM100_grandiflorum_S574:0.00313588,(Ae_huber_morathii:0.00156710,Ae_capitatum:0.00197456):0.00000008):0.00163355,(HM326_erinaceum_S572:0.00670476,Ae_spicatum:0.00628050):0.00000015):0.00828058,(HM86_transhyrcanum_S579:0.00000007,HM104_membranaceum_S573:0.00155300):0.00332673):0.00523236):0.00164100):0.00493522,(Ae_lycium:0.00156649,(Ae_turcica:0.00473694,((Ae_thesiifolium:0.00156565,(Ae_dumanii:0.00646413,(Ae_stenopterum:0.00315615,(Ae_arabicum:0.00000007,(Ae_heterocarpum:0.00314952,((Ae_froedinii:0.00000001,Ae_syriacum:0.00000001):0.00157013,Ae_carneum:0.00000006):0.00000006):0.00000007):0.00313606):0.00313359):0.00155965):0.00000006,(Ae_cordatum:0.00630882,(((Umbellatum_Archibold_J1:0.00000001,HM1454_Oshrurankuh_S1857:0.00159034):0.00641780,(W_0184833_Aethionema_trnT-trnL-trnF:0.00000001,(HM1452_yazd_S1855:0.00000001,_Shirkuh_J2_trnL:0.00000011):0.00000001):0.00234077):0.00231182,((Ae_orbiculatum:0.00000008,((Ae_acarii:0.00887534,Ae_saxatile:0.00000001):0.00362566,(Ae_fimbriatum:0.00815161,(Ae_stylosum:0.00480922,(Ae_speciosum:0.00315304,(Ae_papillosum:0.00157688,Ae_munzurense:0.00315658):0.00000008):0.00315773):0.00000008):0.00157443):0.00157476):0.00157161,(HM480_spec_nova_S647:0.00000008,(HM479_spec._nova_S646:0.00000009,(HM478_spec_nova_S645:0.00157488,HM482_spec_nova_S648:0.00000003):0.00000003):0.00000006):0.00157922):0.00000006):0.00471396):0.00000011):0.00000009):0.00000012):0.01174593):0.01396866);

* AICc MODEL SELECTION : Selection uncertainty

Model -lnL K AICc delta weight cumWeight

-------------------------------------------------------------------------

TPM1uf+G 1954.44432 100 4142.667904 0.000000 0.435254 0.435254

TPM1uf+I+G 1954.15119 101 4144.814943 2.147039 0.148771 0.584025

TIM1+G 1954.31869 101 4145.149943 2.482039 0.125827 0.709853

TPM1uf+I 1955.76233 100 4145.303924 2.636020 0.116503 0.826356

TVM+G 1953.92519 102 4147.105414 4.437509 0.047331 0.873687

TIM1+I+G 1954.03172 102 4147.318474 4.650569 0.042549 0.916236

TIM1+I 1955.62933 101 4147.771223 5.103319 0.033929 0.950165

TVM+I+G 1953.62423 103 4149.255183 6.587278 0.016156 0.966321

GTR+G 1953.83207 103 4149.670863 7.002958 0.013124 0.979445

TVM+I 1955.25236 102 4149.759754 7.091849 0.012554 0.991998

GTR+I+G 1953.53790 104 4151.843477 9.175573 0.004429 0.996427

GTR+I 1955.14881 103 4152.304343 9.636438 0.003517 0.999945

TPM1uf 1965.16426 99 4161.383612 18.715708 3.76e-005 0.999982

TIM1 1964.85284 100 4163.484944 20.817040 1.31e-005 0.999995

TVM 1964.81588 101 4166.144323 23.476419 3.47e-006 0.999999

GTR 1964.51881 102 4168.292654 25.624749 1.19e-006 1.000000

TPM3uf+G 1975.20442 100 4184.188104 41.520200 4.20e-010 1.000000

F81+G 1978.69742 98 4185.734840 43.066936 1.94e-010 1.000000

TPM3uf+I+G 1974.88523 101 4186.283023 43.615119 1.47e-010 1.000000

TPM3uf+I 1976.43500 100 4186.649264 43.981360 1.23e-010 1.000000

TIM3+G 1975.13208 101 4186.776723 44.108819 1.15e-010 1.000000

F81+I+G 1978.36107 99 4187.777232 45.109328 6.97e-011 1.000000

F81+I 1979.84385 98 4188.027700 45.359796 6.15e-011 1.000000

TPM2uf+G 1977.17561 100 4188.130484 45.462580 5.84e-011 1.000000

HKY+G 1978.68979 99 4188.434672 45.766768 5.02e-011 1.000000

TIM3+I+G 1974.82046 102 4188.895954 46.228049 3.99e-011 1.000000

TIM3+I 1976.34961 101 4189.211783 46.543879 3.40e-011 1.000000

TPM2uf+I+G 1976.86696 101 4190.246483 47.578579 2.03e-011 1.000000

HKY+I+G 1978.35406 100 4190.487384 47.819480 1.80e-011 1.000000

TPM2uf+I 1978.38903 100 4190.557324 47.889420 1.74e-011 1.000000

TIM2+G 1977.02700 101 4190.566563 47.898659 1.73e-011 1.000000

HKY+I 1979.83768 99 4190.730452 48.062548 1.59e-011 1.000000

TrN+G 1978.57075 100 4190.920764 48.252860 1.45e-011 1.000000

TIM2+I+G 1976.72556 102 4192.706154 50.038249 5.93e-012 1.000000

TIM2+I 1978.23742 101 4192.987403 50.319499 5.15e-012 1.000000

TrN+I+G 1978.24275 101 4192.998063 50.330159 5.12e-012 1.000000

TrN+I 1979.71054 100 4193.200344 50.532440 4.63e-012 1.000000

TPM3uf 1986.49823 99 4204.051552 61.383648 2.04e-014 1.000000

F81 1990.06503 97 4205.764003 63.096099 8.66e-015 1.000000

TIM3 1986.21875 100 4206.216764 63.548860 6.91e-015 1.000000

TPM2uf 1987.97857 99 4207.012232 64.344328 4.64e-015 1.000000

HKY 1990.04277 98 4208.425540 65.757636 2.29e-015 1.000000

TIM2 1987.64789 100 4209.075044 66.407140 1.65e-015 1.000000

TrN 1989.73049 99 4210.516072 67.848168 8.05e-016 1.000000

TPM1+G 2009.62507 97 4244.884083 102.216179 2.77e-023 1.000000

TPM1+I+G 2009.21342 98 4246.766840 104.098936 1.08e-023 1.000000

TPM1+I 2010.73276 97 4247.099463 104.431559 9.16e-024 1.000000

TIM1ef+G 2009.57238 98 4247.484760 104.816856 7.55e-024 1.000000

TVMef+G 2008.57144 99 4248.197972 105.530068 5.29e-024 1.000000

TIM1ef+I+G 2009.16693 99 4249.388952 106.721048 2.91e-024 1.000000

TIM1ef+I 2010.67594 98 4249.691880 107.023976 2.50e-024 1.000000

TVMef+I+G 2008.13387 100 4250.047004 107.379100 2.10e-024 1.000000

TVMef+I 2009.65512 99 4250.365332 107.697428 1.79e-024 1.000000

SYM+G 2008.51934 100 4250.817944 108.150040 1.43e-024 1.000000

SYM+I+G 2008.08649 101 4252.685543 110.017639 5.61e-025 1.000000

SYM+I 2009.59843 100 4252.976124 110.308220 4.85e-025 1.000000

TPM1 2022.27793 96 4267.492737 124.824833 3.41e-028 1.000000

TIM1ef 2022.06300 97 4269.759943 127.092039 1.10e-028 1.000000

TVMef 2021.68025 98 4271.700500 129.032596 4.17e-029 1.000000

SYM 2021.46580 99 4273.986692 131.318788 1.33e-029 1.000000

JC+G 2027.48582 95 4275.220396 132.552492 7.17e-030 1.000000

JC+I+G 2027.08664 96 4277.110157 134.442253 2.79e-030 1.000000

JC+I 2028.57639 95 4277.401536 134.733632 2.41e-030 1.000000

K80+G 2027.36975 96 4277.676377 135.008473 2.10e-030 1.000000

K80+I+G 2026.97015 97 4279.574243 136.906339 8.13e-031 1.000000

K80+I 2028.46030 96 4279.857477 137.189573 7.05e-031 1.000000

TrNef+G 2027.31565 97 4280.265243 137.597339 5.75e-031 1.000000

TPM3+G 2027.33247 97 4280.298883 137.630979 5.66e-031 1.000000

TPM2+G 2027.36867 97 4280.371283 137.703379 5.46e-031 1.000000

TrNef+I+G 2026.92131 98 4282.182620 139.514716 2.21e-031 1.000000

TPM3+I+G 2026.93735 98 4282.214700 139.546796 2.17e-031 1.000000

TPM2+I+G 2026.96837 98 4282.276740 139.608836 2.10e-031 1.000000

TrNef+I 2028.40172 97 4282.437383 139.769479 1.94e-031 1.000000

TPM3+I 2028.42747 97 4282.488883 139.820979 1.89e-031 1.000000

TPM2+I 2028.43296 97 4282.499863 139.831959 1.88e-031 1.000000

TIM3ef+G 2027.27553 98 4282.891060 140.223156 1.55e-031 1.000000

TIM2ef+G 2027.31470 98 4282.969400 140.301496 1.49e-031 1.000000

TIM3ef+I+G 2026.88670 99 4284.828492 142.160588 5.87e-032 1.000000

TIM2ef+I+G 2026.92040 99 4284.895892 142.227988 5.68e-032 1.000000

TIM3ef+I 2028.36586 98 4285.071720 142.403816 5.20e-032 1.000000

TIM2ef+I 2028.37515 98 4285.090300 142.422396 5.15e-032 1.000000

JC 2039.99294 94 4297.555416 154.887512 1.01e-034 1.000000

K80 2039.88158 95 4300.011916 157.344012 2.96e-035 1.000000

TrNef 2039.66627 96 4302.269417 159.601513 9.59e-036 1.000000

TPM3 2039.66915 96 4302.275177 159.607273 9.56e-036 1.000000

TPM2 2039.68826 96 4302.313397 159.645493 9.38e-036 1.000000

TIM3ef 2039.45433 97 4304.542603 161.874699 3.08e-036 1.000000

TIM2ef 2039.47245 97 4304.578843 161.910939 3.02e-036 1.000000

-------------------------------------------------------------------------

-lnL: negative log likelihod

K: number of estimated parameters

AICc: Corrected Akaike Information Criterion

delta: AICc difference

weight: AICc weight

cumWeight: cumulative AICc weight

* AICc MODEL SELECTION : Confidence interval

There are 88 models in the 100% confidence interval: [ TPM1uf+G TPM1uf+I+G TIM1+G TPM1uf+I TVM+G TIM1+I+G TIM1+I TVM+I+G GTR+G TVM+I GTR+I+G GTR+I TPM1uf TIM1 TVM GTR TPM3uf+G F81+G TPM3uf+I+G TPM3uf+I TIM3+G F81+I+G F81+I TPM2uf+G HKY+G TIM3+I+G TIM3+I TPM2uf+I+G HKY+I+G TPM2uf+I TIM2+G HKY+I TrN+G TIM2+I+G TIM2+I TrN+I+G TrN+I TPM3uf F81 TIM3 TPM2uf HKY TIM2 TrN TPM1+G TPM1+I+G TPM1+I TIM1ef+G TVMef+G TIM1ef+I+G TIM1ef+I TVMef+I+G TVMef+I SYM+G SYM+I+G SYM+I TPM1 TIM1ef TVMef SYM JC+G JC+I+G JC+I K80+G K80+I+G K80+I TrNef+G TPM3+G TPM2+G TrNef+I+G TPM3+I+G TPM2+I+G TrNef+I TPM3+I TPM2+I TIM3ef+G TIM2ef+G TIM3ef+I+G TIM2ef+I+G TIM3ef+I TIM2ef+I JC K80 TrNef TPM3 TPM2 TIM3ef TIM2ef ]

* AICc MODEL SELECTION : Parameter importance

Parameter Importance

----------------------

fA 1.0000

fC 1.0000

fG 1.0000

fT 1.0000

kappa 0.0000

titv 0.0000

rAC 0.0971

rAG 1.0000

rAT 1.0000

rCG 1.0000

rCT 1.0000

rGT 1.0000

pinv(I) 0.1665

alpha(G) 0.6215

pinv(IG) 0.2119

alpha(IG) 0.2119

----------------------

Values have been rounded.

(I): considers only +I models.

(G): considers only +G models.

(IG): considers only +I+G models.

* AICc MODEL SELECTION : Best Model's command line

phyml -i /tmp/jmodeltest13390012638257739752.phy -d nt -n 1 -b 0 --run_id TPM1uf+G -m 012210 -f m -c 4 -a e --no_memory_check -o tlr -s BEST

---------------------------------------------------------------

* *

* MODEL AVERAGED PHYLOGENY *

* *

---------------------------------------------------------------

Selection criterion: . . . . AICc

Confidence interval: . . . . 1.00

Consensus type:. . . . . . . 50% majority rule

Using 88 models in the 1.00 confidence interval = TPM1uf+G TPM1uf+I+G TIM1+G TPM1uf+I TVM+G TIM1+I+G TIM1+I TVM+I+G GTR+G TVM+I GTR+I+G GTR+I TPM1uf TIM1 TVM GTR TPM3uf+G F81+G TPM3uf+I+G TPM3uf+I TIM3+G F81+I+G F81+I TPM2uf+G HKY+G TIM3+I+G TIM3+I TPM2uf+I+G HKY+I+G TPM2uf+I TIM2+G HKY+I TrN+G TIM2+I+G TIM2+I TrN+I+G TrN+I TPM3uf F81 TIM3 TPM2uf HKY TIM2 TrN TPM1+G TPM1+I+G TPM1+I TIM1ef+G TVMef+G TIM1ef+I+G TIM1ef+I TVMef+I+G TVMef+I SYM+G SYM+I+G SYM+I TPM1 TIM1ef TVMef SYM JC+G JC+I+G JC+I K80+G K80+I+G K80+I TrNef+G TPM3+G TPM2+G TrNef+I+G TPM3+I+G TPM2+I+G TrNef+I TPM3+I TPM2+I TIM3ef+G TIM2ef+G TIM3ef+I+G TIM2ef+I+G TIM3ef+I TIM2ef+I JC K80 TrNef TPM3 TPM2 TIM3ef TIM2ef

Species in order:

1. Ae_lepidioides

2. HM95_spinosum_S592

3. Ae_eunomioides

4. Ae_karamanicum

5. Ae_diastrophis

6. Ae_umbellatum

7. Ae_coridifolium

8. Ae_armenum

9. Ae_alanyae

10. Ae_schistosum

11. Ae_demirizii

12. DQ180216_elongatum

13. Ae_glaucinum

14. HM100_grandiflorum_S574

15. Ae_huber_morathii

16. Ae_capitatum

17. HM326_erinaceum_S572

18. Ae_spicatum

19. HM86_transhyrcanum_S579

20. HM104_membranaceum_S573

21. Ae_lycium

22. Ae_turcica

23. Ae_thesiifolium

24. Ae_dumanii

25. Ae_stenopterum

26. Ae_arabicum

27. Ae_heterocarpum

28. Ae_froedinii

29. Ae_syriacum

30. Ae_carneum

31. Ae_cordatum

32. Umbellatum_Archibold_J1

33. HM1454_Oshrurankuh_S1857

34. W_0184833_Aethionema_trnT-trnL-trnF

35. HM1452_yazd_S1855

36. _Shirkuh_J2_trnL

37. Ae_orbiculatum

38. Ae_acarii

39. Ae_saxatile

40. Ae_fimbriatum

41. Ae_stylosum

42. Ae_speciosum

43. Ae_papillosum

44. Ae_munzurense

45. HM480_spec_nova_S647

46. HM479_spec._nova_S646

47. HM478_spec_nova_S645

48. HM482_spec_nova_S648

Bipartitions included in the consensus tree

123456789111111111122222222223333333333444444444

012345678901234567890123456789012345678

************************************************ ( 1.0 )

--********************************************** ( 1.0 )

--******************---------------------------- ( 1.0 )

---*****************---------------------------- ( 1.0 )

---**********----------------------------------- ( 1.0 )

----*********----------------------------------- ( 1.0 )

----****---------------------------------------- ( 1.0 )

-----***---------------------------------------- ( 1.0 )

------**---------------------------------------- ( 0.6216 )

--------*****----------------------------------- ( 0.6216 )

---------****----------------------------------- ( 0.6216 )

----------***----------------------------------- ( 1.0 )

-----------**----------------------------------- ( 0.84606 )

-------------*******---------------------------- ( 1.0 )

-------------*****------------------------------ ( 1.0 )

-------------***-------------------------------- ( 1.0 )

--------------**-------------------------------- ( 0.8335 )

----------------**------------------------------ ( 0.65899 )

------------------**---------------------------- ( 1.0 )

--------------------**************************** ( 1.0 )

-----------------------*******------------------ ( 1.0 )

------------------------******------------------ ( 1.0 )

-------------------------*****------------------ ( 1.0 )

--------------------------****------------------ ( 0.7881 )

---------------------------***------------------ ( 0.6216 )

---------------------------**------------------- ( 1.0 )

-------------------------------***************** ( 1.0 )

-------------------------------*****------------ ( 1.0 )

-------------------------------**--------------- ( 1.0 )

---------------------------------***------------ ( 1.0 )

----------------------------------**------------ ( 1.0 )

------------------------------------************ ( 0.52964 )

------------------------------------********---- ( 1.0 )

-------------------------------------*******---- ( 1.0 )

-------------------------------------**--------- ( 1.0 )

---------------------------------------*****---- ( 1.0 )

----------------------------------------****---- ( 0.7881 )

-----------------------------------------***---- ( 1.0 )

------------------------------------------**---- ( 0.75417 )

--------------------------------------------**** ( 1.0 )

---------------------------------------------*** ( 0.6216 )

+-48 Ae_glaucinum

+-80

| +-47 DQ180216_elongatum

+-----81

| +-46 Ae_demirizii

+-82

| +-45 Ae_schistosum

+-83

| +-44 Ae_alanyae

+-84

| | +-43 Ae_armenum

| | +-77

| | | +-42 Ae_coridifolium

| | +----78

| | | +-41 Ae_umbellatum

| +-79

| +-40 Ae_diastrophis

+--85

| +-39 Ae_karamanicum

+-86

| | +-38 HM104_membranaceum_S573

| | +-75

| | | +-37 HM86_transhyrcanum_S579

| +--76

| | +---36 Ae_spicatum

| | +-73

| | | +---35 HM326_erinaceum_S572

| +-----74

| | +-34 Ae_capitatum

| | +-71

| | | +-33 Ae_huber_morathii

| +-72

| +-32 HM100_grandiflorum_S574

+--87

| +-31 Ae_eunomioides

+----------88

| | +-30 Ae_munzurense

| | +-62

| | | +-29 Ae_papillosum

| | +-63

| | | +-28 Ae_speciosum

| | +-64

| | | +--27 Ae_stylosum

| | +-65

| | | +-----26 Ae_fimbriatum

| | +-66

| | | | +-25 Ae_saxatile

| | | +-61

| | | +-----24 Ae_acarii

| | +-67

| | | +-23 Ae_orbiculatum

| | +-68

| | | | +-22 HM482_spec_nova_S648

| | | | |

| | | | +-21 HM478_spec_nova_S645

| | | | +-59

| | | | | +-20 HM479_spec._nova_S646

| | | +-60

| | | +-19 HM480_spec_nova_S647

| | +--69

| | | | +-18 _Shirkuh_J2_trnL

| | | | +-56

| | | | | +-17 HM1452_yazd_S1855

| | | | +-57

| | | | | +-16 W_0184833_Aethionema_trnT-trnL-trnF

| | | +-58

| | | | +-15 HM1454_Oshrurankuh_S1857

| | | +---55

| | | +-14 Umbellatum_Archibold_J1

| | |

| | | +-13 Ae_syriacum

| | | +-49

| | | | +-12 Ae_froedinii

| | | +-50

| | | | +-11 Ae_carneum

| | | +-51

| | | | +-10 Ae_heterocarpum

| | | +-52

| | | | +--9 Ae_arabicum

| | | +-53

| | | | +--8 Ae_stenopterum

| | +-54

| | | +----7 Ae_dumanii

| | |

| | +----6 Ae_cordatum

| +--------70

| +--5 Ae_thesiifolium

| |

| +---4 Ae_turcica

| |

| +--3 Ae_lycium

|

+---2 HM95_spinosum_S592

|

+--------------------------1 Ae_lepidioides

(Ae_lepidioides:0.0312133700,HM95_spinosum_S592:0.0051002200,((Ae_lycium:0.0015664900,Ae_turcica:0.0047369400,Ae_thesiifolium:0.0015656500,Ae_cordatum:0.0063088200,(Ae_dumanii:0.0064641300,(Ae_stenopterum:0.0031561500,(Ae_arabicum:0.0000000700,(Ae_heterocarpum:0.0031495200,(Ae_carneum:0.0000000600,(Ae_froedinii:0.0000000100,Ae_syriacum:0.0000000100):1.0000:0.0015701300):0.6216:0.0000000600):0.7881:0.0000000700):1.0000:0.0031360600):1.0000:0.0031335900):1.0000:0.0015588300,(((Umbellatum_Archibold_J1:0.0000000100,HM1454_Oshrurankuh_S1857:0.0015903400):1.0000:0.0064178000,(W_0184833_Aethionema_trnT-trnL-trnF:0.0000000100,(HM1452_yazd_S1855:0.0000000100,_Shirkuh_J2_trnL:0.0000001100):1.0000:0.0000000100):1.0000:0.0023404000):1.0000:0.0023118200,((HM480_spec_nova_S647:0.0000000700,(HM479_spec._nova_S646:0.0000000900,HM478_spec_nova_S645:0.0015748800,HM482_spec_nova_S648:0.0000000500):0.6216:0.0000000600):1.0000:0.0015791700,(Ae_orbiculatum:0.0000000800,((Ae_acarii:0.0088753400,Ae_saxatile:0.0000000100):1.0000:0.0036256600,(Ae_fimbriatum:0.0081516100,(Ae_stylosum:0.0048092200,(Ae_speciosum:0.0031530400,(Ae_papillosum:0.0015768800,Ae_munzurense:0.0031565800):0.7542:0.0000000800):1.0000:0.0031577300):0.7881:0.0000000800):1.0000:0.0015744300):1.0000:0.0015747600):1.0000:0.0015716100):0.5296:0.0000000600):1.0000:0.0047139600):1.0000:0.0117459300,(Ae_eunomioides:0.0031764300,((((HM100_grandiflorum_S574:0.0031356200,(Ae_huber_morathii:0.0015668500,Ae_capitatum:0.0019745600):0.8335:0.0000000800):1.0000:0.0016335500,(HM326_erinaceum_S572:0.0067047600,Ae_spicatum:0.0062796200):0.6590:0.0000001500):1.0000:0.0082805800,(HM86_transhyrcanum_S579:0.0000000700,HM104_membranaceum_S573:0.0015530000):1.0000:0.0033267300):1.0000:0.0052323600,(Ae_karamanicum:0.0000000800,((Ae_diastrophis:0.0000000800,(Ae_umbellatum:0.0031101000,(Ae_coridifolium:0.0000000100,Ae_armenum:0.0015499200):0.6216:0.0000000100):1.0000:0.0078526500):1.0000:0.0015569500,(Ae_alanyae:0.0000001100,(Ae_schistosum:0.0031349100,(Ae_demirizii:0.0000000100,(DQ180216_elongatum:0.0000000100,Ae_glaucinum:0.0000000700):0.8461:0.0000003800):1.0000:0.0083285900):0.6216:0.0000006100):0.6216:0.0000000800):1.0000:0.0016465700):1.0000:0.0050374100):1.0000:0.0016410000):1.0000:0.0049263800):1.0000:0.0139686600);

Note: this tree is unrooted. Branch lengths are the expected number of substitutions per site. Labels next to parentheses represent phylogenetic uncertainty due to model selection (see documentation)

---------------------------------------------------------------

* *

* BAYESIAN INFORMATION CRITERION (BIC) *

* *

---------------------------------------------------------------

Sample size: 699.0

Model selected:

Model = TPM1uf+G

partition = 012210

-lnL = 1954.4443

K = 100

freqA = 0.3134

freqC = 0.1596

freqG = 0.1626

freqT = 0.3644

R(a) [AC] = 1.0000

R(b) [AG] = 0.5881

R(c) [AT] = 0.2111

R(d) [CG] = 0.2111

R(e) [CT] = 0.5881

R(f) [GT] = 1.0000

gamma shape = 0.5160

--

PAUP* Commands Block:

If you want to load the selected model and associated estimates in PAUP*,

attach the next block of commands after the data in your PAUP file:

[!

Likelihood settings from best-fit model (TPM1uf+G) selected by BIC

with jModeltest 2.1.10 v20160303 on Thu Dec 29 23:00:58 PST 2022]

BEGIN PAUP;

Lset base=(0.3134 0.1596 0.1626 ) nst=6 rmat=(1.0000 0.5881 0.2111 0.2111 0.5881) rates=gamma shape=0.5160 ncat=4 pinvar=0;

END;

--

Tree for the best BIC model = (Ae_lepidioides:0.03121337,HM95_spinosum_S592:0.00510022,((Ae_eunomioides:0.00317643,((Ae_karamanicum:0.00000008,((Ae_diastrophis:0.00000008,(Ae_umbellatum:0.00311010,(Ae_coridifolium:0.00000001,Ae_armenum:0.00154992):0.00000001):0.00785265):0.00155695,(Ae_alanyae:0.00000011,(Ae_schistosum:0.00313491,(Ae_demirizii:0.00000001,(DQ180216_elongatum:0.00000001,Ae_glaucinum:0.00000007):0.00000038):0.00832859):0.00000061):0.00000008):0.00164657):0.00503741,(((HM100_grandiflorum_S574:0.00313588,(Ae_huber_morathii:0.00156710,Ae_capitatum:0.00197456):0.00000008):0.00163355,(HM326_erinaceum_S572:0.00670476,Ae_spicatum:0.00628050):0.00000015):0.00828058,(HM86_transhyrcanum_S579:0.00000007,HM104_membranaceum_S573:0.00155300):0.00332673):0.00523236):0.00164100):0.00493522,(Ae_lycium:0.00156649,(Ae_turcica:0.00473694,((Ae_thesiifolium:0.00156565,(Ae_dumanii:0.00646413,(Ae_stenopterum:0.00315615,(Ae_arabicum:0.00000007,(Ae_heterocarpum:0.00314952,((Ae_froedinii:0.00000001,Ae_syriacum:0.00000001):0.00157013,Ae_carneum:0.00000006):0.00000006):0.00000007):0.00313606):0.00313359):0.00155965):0.00000006,(Ae_cordatum:0.00630882,(((Umbellatum_Archibold_J1:0.00000001,HM1454_Oshrurankuh_S1857:0.00159034):0.00641780,(W_0184833_Aethionema_trnT-trnL-trnF:0.00000001,(HM1452_yazd_S1855:0.00000001,_Shirkuh_J2_trnL:0.00000011):0.00000001):0.00234077):0.00231182,((Ae_orbiculatum:0.00000008,((Ae_acarii:0.00887534,Ae_saxatile:0.00000001):0.00362566,(Ae_fimbriatum:0.00815161,(Ae_stylosum:0.00480922,(Ae_speciosum:0.00315304,(Ae_papillosum:0.00157688,Ae_munzurense:0.00315658):0.00000008):0.00315773):0.00000008):0.00157443):0.00157476):0.00157161,(HM480_spec_nova_S647:0.00000008,(HM479_spec._nova_S646:0.00000009,(HM478_spec_nova_S645:0.00157488,HM482_spec_nova_S648:0.00000003):0.00000003):0.00000006):0.00157922):0.00000006):0.00471396):0.00000011):0.00000009):0.00000012):0.01174593):0.01396866);

* BIC MODEL SELECTION : Selection uncertainty

Model -lnL K BIC delta weight cumWeight

-------------------------------------------------------------------------

TPM1uf+G 1954.44432 100 4563.853714 0.000000 0.725217 0.725217

TPM1uf+I 1955.76233 100 4566.489734 2.636020 0.194117 0.919333

TPM1uf+I+G 1954.15119 101 4569.817105 5.963391 0.036773 0.956107

TIM1+G 1954.31869 101 4570.152105 6.298391 0.031102 0.987209

TIM1+I 1955.62933 101 4572.773385 8.919671 0.008387 0.995595

TVM+G 1953.92519 102 4575.914756 12.061041 0.001744 0.997339

TIM1+I+G 1954.03172 102 4576.127816 12.274101 0.001567 0.998906

TVM+I 1955.25236 102 4578.569096 14.715381 0.000462 0.999369

TPM1uf 1965.16426 99 4578.743943 14.890229 0.000424 0.999793

TVM+I+G 1953.62423 103 4581.862486 18.008772 8.91e-005 0.999882

GTR+G 1953.83207 103 4582.278166 18.424452 7.24e-005 0.999954

TIM1 1964.85284 100 4584.670754 20.817040 2.19e-005 0.999976

GTR+I 1955.14881 103 4584.911646 21.057932 1.94e-005 0.999995

GTR+I+G 1953.53790 104 4588.239477 24.385763 3.67e-006 0.999999

TVM 1964.81588 101 4591.146485 27.292771 8.59e-007 1.000000

GTR 1964.51881 102 4597.101996 33.248281 4.37e-008 1.000000

F81+G 1978.69742 98 4599.260613 35.406899 1.49e-008 1.000000

F81+I 1979.84385 98 4601.553473 37.699759 4.72e-009 1.000000

F81+I+G 1978.36107 99 4605.137563 41.283849 7.87e-010 1.000000

TPM3uf+G 1975.20442 100 4605.373914 41.520200 6.99e-010 1.000000

HKY+G 1978.68979 99 4605.795003 41.941289 5.66e-010 1.000000

TPM3uf+I 1976.43500 100 4607.835074 43.981360 2.04e-010 1.000000

HKY+I 1979.83768 99 4608.090783 44.237069 1.80e-010 1.000000

TPM2uf+G 1977.17561 100 4609.316294 45.462580 9.74e-011 1.000000

TPM3uf+I+G 1974.88523 101 4611.285185 47.431471 3.64e-011 1.000000

HKY+I+G 1978.35406 100 4611.673194 47.819480 3.00e-011 1.000000

TPM2uf+I 1978.38903 100 4611.743134 47.889420 2.89e-011 1.000000

TIM3+G 1975.13208 101 4611.778885 47.925171 2.84e-011 1.000000

TrN+G 1978.57075 100 4612.106574 48.252860 2.41e-011 1.000000

TIM3+I 1976.34961 101 4614.213945 50.360231 8.41e-012 1.000000

TrN+I 1979.71054 100 4614.386154 50.532440 7.72e-012 1.000000

TPM2uf+I+G 1976.86696 101 4615.248645 51.394931 5.01e-012 1.000000

F81 1990.06503 97 4615.446182 51.592468 4.54e-012 1.000000

TIM2+G 1977.02700 101 4615.568725 51.715011 4.27e-012 1.000000

TIM3+I+G 1974.82046 102 4617.705296 53.851581 1.47e-012 1.000000

TIM2+I 1978.23742 101 4617.989565 54.135851 1.27e-012 1.000000

TrN+I+G 1978.24275 101 4618.000225 54.146511 1.27e-012 1.000000

TPM3uf 1986.49823 99 4621.411883 57.558169 2.30e-013 1.000000

TIM2+I+G 1976.72556 102 4621.515496 57.661781 2.18e-013 1.000000

HKY 1990.04277 98 4621.951313 58.097599 1.76e-013 1.000000

TPM2uf 1987.97857 99 4624.372563 60.518849 5.24e-014 1.000000

TIM3 1986.21875 100 4627.402574 63.548860 1.15e-014 1.000000

TrN 1989.73049 99 4627.876403 64.022689 9.08e-015 1.000000

TIM2 1987.64789 100 4630.260854 66.407140 2.76e-015 1.000000

TPM1+G 2009.62507 97 4654.566262 90.712548 1.45e-020 1.000000

TPM1+I 2010.73276 97 4656.781642 92.927928 4.80e-021 1.000000

TPM1+I+G 2009.21342 98 4660.292613 96.438899 8.30e-022 1.000000

TIM1ef+G 2009.57238 98 4661.010533 97.156819 5.80e-022 1.000000

TIM1ef+I 2010.67594 98 4663.217653 99.363939 1.92e-022 1.000000

TVMef+G 2008.57144 99 4665.558303 101.704589 5.96e-023 1.000000

TIM1ef+I+G 2009.16693 99 4666.749283 102.895569 3.29e-023 1.000000

TVMef+I 2009.65512 99 4667.725663 103.871949 2.02e-023 1.000000

TVMef+I+G 2008.13387 100 4671.232814 107.379100 3.49e-024 1.000000

SYM+G 2008.51934 100 4672.003754 108.150040 2.38e-024 1.000000

TPM1 2022.27793 96 4673.322331 109.468617 1.23e-024 1.000000

SYM+I 2009.59843 100 4674.161934 110.308220 8.08e-025 1.000000

JC+G 2027.48582 95 4677.188461 113.334746 1.78e-025 1.000000

SYM+I+G 2008.08649 101 4677.687705 113.833991 1.39e-025 1.000000

JC+I 2028.57639 95 4679.369601 115.515886 5.98e-026 1.000000

TIM1ef 2022.06300 97 4679.442122 115.588408 5.76e-026 1.000000

JC+I+G 2027.08664 96 4682.939751 119.086037 1.00e-026 1.000000

K80+G 2027.36975 96 4683.505971 119.652257 7.56e-027 1.000000

TVMef 2021.68025 98 4685.226273 121.372559 3.20e-027 1.000000

K80+I 2028.46030 96 4685.687071 121.833357 2.54e-027 1.000000

K80+I+G 2026.97015 97 4689.256422 125.402708 4.26e-028 1.000000

TrNef+G 2027.31565 97 4689.947422 126.093708 3.02e-028 1.000000

TPM3+G 2027.33247 97 4689.981062 126.127348 2.97e-028 1.000000

TPM2+G 2027.36867 97 4690.053462 126.199748 2.86e-028 1.000000

SYM 2021.46580 99 4691.347023 127.493309 1.50e-028 1.000000

TrNef+I 2028.40172 97 4692.119562 128.265848 1.02e-028 1.000000

TPM3+I 2028.42747 97 4692.171062 128.317348 9.92e-029 1.000000

TPM2+I 2028.43296 97 4692.182042 128.328328 9.87e-029 1.000000

JC 2039.99294 94 4695.653050 131.799336 1.74e-029 1.000000

TrNef+I+G 2026.92131 98 4695.708393 131.854679 1.69e-029 1.000000

TPM3+I+G 2026.93735 98 4695.740473 131.886759 1.67e-029 1.000000

TPM2+I+G 2026.96837 98 4695.802513 131.948799 1.61e-029 1.000000

TIM3ef+G 2027.27553 98 4696.416833 132.563119 1.19e-029 1.000000

TIM2ef+G 2027.31470 98 4696.495173 132.641459 1.14e-029 1.000000

TIM3ef+I 2028.36586 98 4698.597493 134.743779 3.99e-030 1.000000

TIM2ef+I 2028.37515 98 4698.616073 134.762359 3.96e-030 1.000000

K80 2039.88158 95 4701.979981 138.126266 7.36e-031 1.000000

TIM3ef+I+G 2026.88670 99 4702.188823 138.335109 6.63e-031 1.000000

TIM2ef+I+G 2026.92040 99 4702.256223 138.402509 6.41e-031 1.000000

TrNef 2039.66627 96 4708.099011 144.245297 3.45e-032 1.000000

TPM3 2039.66915 96 4708.104771 144.251057 3.44e-032 1.000000

TPM2 2039.68826 96 4708.142991 144.289277 3.38e-032 1.000000

TIM3ef 2039.45433 97 4714.224782 150.371068 1.61e-033 1.000000

TIM2ef 2039.47245 97 4714.261022 150.407308 1.58e-033 1.000000

-------------------------------------------------------------------------

-lnL: negative log likelihod

K: number of estimated parameters

BIC: Bayesian Information Criterion

delta: BIC difference

weight: BIC weight

cumWeight: cumulative BIC weight

* BIC MODEL SELECTION : Confidence interval

There are 88 models in the 100% confidence interval: [ TPM1uf+G TPM1uf+I TPM1uf+I+G TIM1+G TIM1+I TVM+G TIM1+I+G TVM+I TPM1uf TVM+I+G GTR+G TIM1 GTR+I GTR+I+G TVM GTR F81+G F81+I F81+I+G TPM3uf+G HKY+G TPM3uf+I HKY+I TPM2uf+G TPM3uf+I+G HKY+I+G TPM2uf+I TIM3+G TrN+G TIM3+I TrN+I TPM2uf+I+G F81 TIM2+G TIM3+I+G TIM2+I TrN+I+G TPM3uf TIM2+I+G HKY TPM2uf TIM3 TrN TIM2 TPM1+G TPM1+I TPM1+I+G TIM1ef+G TIM1ef+I TVMef+G TIM1ef+I+G TVMef+I TVMef+I+G SYM+G TPM1 SYM+I JC+G SYM+I+G JC+I TIM1ef JC+I+G K80+G TVMef K80+I K80+I+G TrNef+G TPM3+G TPM2+G SYM TrNef+I TPM3+I TPM2+I JC TrNef+I+G TPM3+I+G TPM2+I+G TIM3ef+G TIM2ef+G TIM3ef+I TIM2ef+I K80 TIM3ef+I+G TIM2ef+I+G TrNef TPM3 TPM2 TIM3ef TIM2ef ]

* BIC MODEL SELECTION : Parameter importance

Parameter Importance

----------------------

fA 1.0000

fC 1.0000

fG 1.0000

fT 1.0000

kappa 0.0000

titv 0.0000

rAC 0.0024

rAG 1.0000

rAT 1.0000

rCG 1.0000

rCT 1.0000

rGT 1.0000

pinv(I) 0.2030

alpha(G) 0.7581

pinv(IG) 0.0384

alpha(IG) 0.0384

----------------------

Values have been rounded.

(I): considers only +I models.

(G): considers only +G models.

(IG): considers only +I+G models.

* BIC MODEL SELECTION : Best Model's command line

phyml -i /tmp/jmodeltest13390012638257739752.phy -d nt -n 1 -b 0 --run_id TPM1uf+G -m 012210 -f m -c 4 -a e --no_memory_check -o tlr -s BEST

---------------------------------------------------------------

* *

* MODEL AVERAGED PHYLOGENY *

* *

---------------------------------------------------------------

Selection criterion: . . . . BIC

Confidence interval: . . . . 1.00

Consensus type:. . . . . . . 50% majority rule

Using 88 models in the 1.00 confidence interval = TPM1uf+G TPM1uf+I TPM1uf+I+G TIM1+G TIM1+I TVM+G TIM1+I+G TVM+I TPM1uf TVM+I+G GTR+G TIM1 GTR+I GTR+I+G TVM GTR F81+G F81+I F81+I+G TPM3uf+G HKY+G TPM3uf+I HKY+I TPM2uf+G TPM3uf+I+G HKY+I+G TPM2uf+I TIM3+G TrN+G TIM3+I TrN+I TPM2uf+I+G F81 TIM2+G TIM3+I+G TIM2+I TrN+I+G TPM3uf TIM2+I+G HKY TPM2uf TIM3 TrN TIM2 TPM1+G TPM1+I TPM1+I+G TIM1ef+G TIM1ef+I TVMef+G TIM1ef+I+G TVMef+I TVMef+I+G SYM+G TPM1 SYM+I JC+G SYM+I+G JC+I TIM1ef JC+I+G K80+G TVMef K80+I K80+I+G TrNef+G TPM3+G TPM2+G SYM TrNef+I TPM3+I TPM2+I JC TrNef+I+G TPM3+I+G TPM2+I+G TIM3ef+G TIM2ef+G TIM3ef+I TIM2ef+I K80 TIM3ef+I+G TIM2ef+I+G TrNef TPM3 TPM2 TIM3ef TIM2ef

Species in order:

1. Ae_lepidioides

2. HM95_spinosum_S592

3. Ae_eunomioides

4. Ae_karamanicum

5. Ae_diastrophis

6. Ae_umbellatum

7. Ae_coridifolium

8. Ae_armenum

9. Ae_alanyae

10. Ae_schistosum

11. Ae_demirizii

12. DQ180216_elongatum

13. Ae_glaucinum

14. HM100_grandiflorum_S574

15. Ae_huber_morathii

16. Ae_capitatum

17. HM326_erinaceum_S572

18. Ae_spicatum

19. HM86_transhyrcanum_S579

20. HM104_membranaceum_S573

21. Ae_lycium

22. Ae_turcica

23. Ae_thesiifolium

24. Ae_dumanii

25. Ae_stenopterum

26. Ae_arabicum

27. Ae_heterocarpum

28. Ae_froedinii

29. Ae_syriacum

30. Ae_carneum

31. Ae_cordatum

32. Umbellatum_Archibold_J1

33. HM1454_Oshrurankuh_S1857

34. W_0184833_Aethionema_trnT-trnL-trnF

35. HM1452_yazd_S1855

36. _Shirkuh_J2_trnL

37. Ae_orbiculatum

38. Ae_acarii

39. Ae_saxatile

40. Ae_fimbriatum

41. Ae_stylosum

42. Ae_speciosum

43. Ae_papillosum

44. Ae_munzurense

45. HM480_spec_nova_S647

46. HM479_spec._nova_S646

47. HM478_spec_nova_S645

48. HM482_spec_nova_S648

Bipartitions included in the consensus tree

123456789111111111122222222223333333333444444444

012345678901234567890123456789012345678

************************************************ ( 1.0 )

--********************************************** ( 1.0 )

--******************---------------------------- ( 1.0 )

---*****************---------------------------- ( 1.0 )

---**********----------------------------------- ( 1.0 )

----*********----------------------------------- ( 1.0 )

----****---------------------------------------- ( 1.0 )

-----***---------------------------------------- ( 1.0 )

------**---------------------------------------- ( 0.75859 )

--------*****----------------------------------- ( 0.75859 )

---------****----------------------------------- ( 0.75859 )

----------***----------------------------------- ( 1.0 )

-----------**----------------------------------- ( 0.79748 )

-------------*******---------------------------- ( 1.0 )

-------------*****------------------------------ ( 1.0 )

-------------***-------------------------------- ( 1.0 )

--------------**-------------------------------- ( 0.79702 )

----------------**------------------------------ ( 0.76655 )

------------------**---------------------------- ( 1.0 )

--------------------**************************** ( 1.0 )

---------------------*************************** ( 0.72704 )

----------------------************************** ( 0.72704 )

----------------------********------------------ ( 0.72704 )

-----------------------*******------------------ ( 1.0 )

------------------------******------------------ ( 1.0 )

-------------------------*****------------------ ( 1.0 )

--------------------------****------------------ ( 0.96157 )

---------------------------***------------------ ( 0.75859 )

---------------------------**------------------- ( 1.0 )

------------------------------****************** ( 0.72704 )

-------------------------------***************** ( 1.0 )

-------------------------------*****------------ ( 1.0 )

-------------------------------**--------------- ( 1.0 )

---------------------------------***------------ ( 1.0 )

----------------------------------**------------ ( 1.0 )

------------------------------------************ ( 0.73542 )

------------------------------------********---- ( 1.0 )

-------------------------------------*******---- ( 1.0 )

-------------------------------------**--------- ( 1.0 )

---------------------------------------*****---- ( 1.0 )

----------------------------------------****---- ( 0.96157 )

-----------------------------------------***---- ( 1.0 )

------------------------------------------**---- ( 0.95318 )

--------------------------------------------**** ( 1.0 )

---------------------------------------------*** ( 0.75859 )

----------------------------------------------** ( 0.72704 )

+-48 Ae_glaucinum

+-85

| +-47 DQ180216_elongatum

+-----86

| +-46 Ae_demirizii

+-87

| +-45 Ae_schistosum

+-88

| +-44 Ae_alanyae

+-89

| | +-43 Ae_armenum

| | +-82

| | | +-42 Ae_coridifolium

| | +----83

| | | +-41 Ae_umbellatum

| +-84

| +-40 Ae_diastrophis

+--90

| +-39 Ae_karamanicum

+-91

| | +-38 HM104_membranaceum_S573

| | +-80

| | | +-37 HM86_transhyrcanum_S579

| +--81

| | +---36 Ae_spicatum

| | +-78

| | | +---35 HM326_erinaceum_S572

| +-----79

| | +-34 Ae_capitatum

| | +-76

| | | +-33 Ae_huber_morathii

| +-77

| +-32 HM100_grandiflorum_S574

+--92

| +-31 Ae_eunomioides

+----------93

| | +-30 Ae_syriacum

| | +-66

| | | +-29 Ae_froedinii

| | +-67

| | | +-28 Ae_carneum

| | +-68

| | | +-27 Ae_heterocarpum

| | +-69

| | | +-26 Ae_arabicum

| | +-70

| | | +-25 Ae_stenopterum

| | +-71

| | | +---24 Ae_dumanii

| | +-72

| | | +-23 Ae_thesiifolium

| | +-73

| | | | +-22 Ae_munzurense

| | | | +-57

| | | | | +-21 Ae_papillosum

| | | | +-58

| | | | | +-20 Ae_speciosum

| | | | +-59

| | | | | +--19 Ae_stylosum

| | | | +-60

| | | | | +-----18 Ae_fimbriatum

| | | | +-61

| | | | | | +-17 Ae_saxatile

| | | | | +-56

| | | | | +-----16 Ae_acarii

| | | | +-62

| | | | | +-15 Ae_orbiculatum

| | | | +-63

| | | | | | +-14 HM482_spec_nova_S648

| | | | | | +-53

| | | | | | | +-13 HM478_spec_nova_S645

| | | | | | +-54

| | | | | | | +-12 HM479_spec._nova_S646

| | | | | +-55

| | | | | +-11 HM480_spec_nova_S647

| | | | +--64

| | | | | | +-10 _Shirkuh_J2_trnL

| | | | | | +-50

| | | | | | | +--9 HM1452_yazd_S1855

| | | | | | +-51

| | | | | | | +--8 W_0184833_Aethionema_trnT-trnL-trnF

| | | | | +-52

| | | | | | +--7 HM1454_Oshrurankuh_S1857

| | | | | +---49

| | | | | +--6 Umbellatum_Archibold_J1

| | | +-65

| | | +----5 Ae_cordatum

| | +-74

| | | +---4 Ae_turcica

| +--------75

| +--3 Ae_lycium

|

+---2 HM95_spinosum_S592

|

+--------------------------1 Ae_lepidioides

(Ae_lepidioides:0.0312133700,HM95_spinosum_S592:0.0051002200,((Ae_lycium:0.0015664900,(Ae_turcica:0.0047369400,((Ae_cordatum:0.0063088200,(((Umbellatum_Archibold_J1:0.0000000100,HM1454_Oshrurankuh_S1857:0.0015903400):1.0000:0.0064178000,(W_0184833_Aethionema_trnT-trnL-trnF:0.0000000100,(HM1452_yazd_S1855:0.0000000100,_Shirkuh_J2_trnL:0.0000001100):1.0000:0.0000000100):1.0000:0.0023407700):1.0000:0.0023118200,((HM480_spec_nova_S647:0.0000000800,(HM479_spec._nova_S646:0.0000000900,(HM478_spec_nova_S645:0.0015748800,HM482_spec_nova_S648:0.0000000300):0.7270:0.0000000300):0.7586:0.0000000600):1.0000:0.0015792200,(Ae_orbiculatum:0.0000000800,((Ae_acarii:0.0088753400,Ae_saxatile:0.0000000100):1.0000:0.0036256600,(Ae_fimbriatum:0.0081516100,(Ae_stylosum:0.0048092200,(Ae_speciosum:0.0031530400,(Ae_papillosum:0.0015768800,Ae_munzurense:0.0031565800):0.9532:0.0000000800):1.0000:0.0031577300):0.9616:0.0000000800):1.0000:0.0015744300):1.0000:0.0015747600):1.0000:0.0015716100):0.7354:0.0000000600):1.0000:0.0047139600):0.7270:0.0000001100,(Ae_thesiifolium:0.0015656500,(Ae_dumanii:0.0064641300,(Ae_stenopterum:0.0031561500,(Ae_arabicum:0.0000000700,(Ae_heterocarpum:0.0031495200,(Ae_carneum:0.0000000600,(Ae_froedinii:0.0000000100,Ae_syriacum:0.0000000100):1.0000:0.0015701300):0.7586:0.0000000600):0.9616:0.0000000700):1.0000:0.0031360600):1.0000:0.0031335900):1.0000:0.0015596500):0.7270:0.0000000600):0.7270:0.0000000900):0.7270:0.0000001200):1.0000:0.0117459300,(Ae_eunomioides:0.0031764300,((((HM100_grandiflorum_S574:0.0031358800,(Ae_huber_morathii:0.0015671000,Ae_capitatum:0.0019745600):0.7970:0.0000000800):1.0000:0.0016335500,(HM326_erinaceum_S572:0.0067047600,Ae_spicatum:0.0062805000):0.7665:0.0000001500):1.0000:0.0082805800,(HM86_transhyrcanum_S579:0.0000000700,HM104_membranaceum_S573:0.0015530000):1.0000:0.0033267300):1.0000:0.0052323600,(Ae_karamanicum:0.0000000800,((Ae_diastrophis:0.0000000800,(Ae_umbellatum:0.0031101000,(Ae_coridifolium:0.0000000100,Ae_armenum:0.0015499200):0.7586:0.0000000100):1.0000:0.0078526500):1.0000:0.0015569500,(Ae_alanyae:0.0000001100,(Ae_schistosum:0.0031349100,(Ae_demirizii:0.0000000100,(DQ180216_elongatum:0.0000000100,Ae_glaucinum:0.0000000700):0.7975:0.0000003800):1.0000:0.0083285900):0.7586:0.0000006100):0.7586:0.0000000800):1.0000:0.0016465700):1.0000:0.0050374100):1.0000:0.0016410000):1.0000:0.0049352200):1.0000:0.0139686600);

Note: this tree is unrooted. Branch lengths are the expected number of substitutions per site. Labels next to parentheses represent phylogenetic uncertainty due to model selection (see documentation)

---------------------------------------------------------------

* *

* DECISION THEORY PERFORMANCE-BASED SELECTION (DT) *

* *

---------------------------------------------------------------

Sample size: 699.0

Model selected:

Model = TPM1uf+G

partition = 012210

-lnL = 1954.4443

K = 100

freqA = 0.3134

freqC = 0.1596

freqG = 0.1626

freqT = 0.3644

R(a) [AC] = 1.0000

R(b) [AG] = 0.5881

R(c) [AT] = 0.2111

R(d) [CG] = 0.2111

R(e) [CT] = 0.5881

R(f) [GT] = 1.0000

gamma shape = 0.5160

--

PAUP* Commands Block:

If you want to load the selected model and associated estimates in PAUP*,

attach the next block of commands after the data in your PAUP file:

[!

Likelihood settings from best-fit model (TPM1uf+G) selected by DT

with jModeltest 2.1.10 v20160303 on Thu Dec 29 23:00:58 PST 2022]

BEGIN PAUP;

Lset base=(0.3134 0.1596 0.1626 ) nst=6 rmat=(1.0000 0.5881 0.2111 0.2111 0.5881) rates=gamma shape=0.5160 ncat=4 pinvar=0;

END;

--

Tree for the best DT model = (Ae_lepidioides:0.03121337,HM95_spinosum_S592:0.00510022,((Ae_eunomioides:0.00317643,((Ae_karamanicum:0.00000008,((Ae_diastrophis:0.00000008,(Ae_umbellatum:0.00311010,(Ae_coridifolium:0.00000001,Ae_armenum:0.00154992):0.00000001):0.00785265):0.00155695,(Ae_alanyae:0.00000011,(Ae_schistosum:0.00313491,(Ae_demirizii:0.00000001,(DQ180216_elongatum:0.00000001,Ae_glaucinum:0.00000007):0.00000038):0.00832859):0.00000061):0.00000008):0.00164657):0.00503741,(((HM100_grandiflorum_S574:0.00313588,(Ae_huber_morathii:0.00156710,Ae_capitatum:0.00197456):0.00000008):0.00163355,(HM326_erinaceum_S572:0.00670476,Ae_spicatum:0.00628050):0.00000015):0.00828058,(HM86_transhyrcanum_S579:0.00000007,HM104_membranaceum_S573:0.00155300):0.00332673):0.00523236):0.00164100):0.00493522,(Ae_lycium:0.00156649,(Ae_turcica:0.00473694,((Ae_thesiifolium:0.00156565,(Ae_dumanii:0.00646413,(Ae_stenopterum:0.00315615,(Ae_arabicum:0.00000007,(Ae_heterocarpum:0.00314952,((Ae_froedinii:0.00000001,Ae_syriacum:0.00000001):0.00157013,Ae_carneum:0.00000006):0.00000006):0.00000007):0.00313606):0.00313359):0.00155965):0.00000006,(Ae_cordatum:0.00630882,(((Umbellatum_Archibold_J1:0.00000001,HM1454_Oshrurankuh_S1857:0.00159034):0.00641780,(W_0184833_Aethionema_trnT-trnL-trnF:0.00000001,(HM1452_yazd_S1855:0.00000001,_Shirkuh_J2_trnL:0.00000011):0.00000001):0.00234077):0.00231182,((Ae_orbiculatum:0.00000008,((Ae_acarii:0.00887534,Ae_saxatile:0.00000001):0.00362566,(Ae_fimbriatum:0.00815161,(Ae_stylosum:0.00480922,(Ae_speciosum:0.00315304,(Ae_papillosum:0.00157688,Ae_munzurense:0.00315658):0.00000008):0.00315773):0.00000008):0.00157443):0.00157476):0.00157161,(HM480_spec_nova_S647:0.00000008,(HM479_spec._nova_S646:0.00000009,(HM478_spec_nova_S645:0.00157488,HM482_spec_nova_S648:0.00000003):0.00000003):0.00000006):0.00157922):0.00000006):0.00471396):0.00000011):0.00000009):0.00000012):0.01174593):0.01396866);

* DT MODEL SELECTION : Selection uncertainty

Model -lnL K DT delta weight cumWeight

-------------------------------------------------------------------------

TPM1uf+G 1954.44432 100 0.007564 0.000000 0.035102 0.035102

GTR+G 1953.83207 103 0.007614 0.000050 0.034870 0.069971

TVM+G 1953.92519 102 0.007665 0.000101 0.034640 0.104611

TIM2+G 1977.02700 101 0.021816 0.014252 0.012170 0.116782

TPM2uf+G 1977.17561 100 0.021831 0.014267 0.012162 0.128943

TIM1+G 1954.31869 101 0.021831 0.014267 0.012162 0.141105

TrN+G 1978.57075 100 0.021863 0.014299 0.012144 0.153249

TIM3+G 1975.13208 101 0.021867 0.014303 0.012142 0.165391

HKY+G 1978.68979 99 0.021881 0.014317 0.012134 0.177525

F81+G 1978.69742 98 0.021881 0.014317 0.012134 0.189659

TPM3uf+G 1975.20442 100 0.021884 0.014320 0.012132 0.201792

TIM1+I 1955.62933 101 0.023133 0.015569 0.011477 0.213269

TIM2+I 1978.23742 101 0.023175 0.015611 0.011457 0.224725

TPM1uf+I 1955.76233 100 0.023182 0.015618 0.011453 0.236179

TPM2uf+I 1978.38903 100 0.023192 0.015628 0.011448 0.247627

GTR+I 1955.14881 103 0.023204 0.015640 0.011442 0.259069

TVM+I 1955.25236 102 0.023224 0.015660 0.011432 0.270501

TIM2 1987.64789 100 0.023325 0.015761 0.011383 0.281884

TPM2uf 1987.97857 99 0.023337 0.015773 0.011377 0.293261

GTR 1964.51881 102 0.023502 0.015938 0.011297 0.304558

TIM1 1964.85284 100 0.023505 0.015941 0.011296 0.315854

TVM 1964.81588 101 0.023511 0.015947 0.011293 0.327147

TPM1uf 1965.16426 99 0.023516 0.015952 0.011291 0.338437

TIM3 1986.21875 100 0.023521 0.015957 0.011288 0.349725

TPM3uf 1986.49823 99 0.023527 0.015963 0.011285 0.361010

TrN 1989.73049 99 0.023547 0.015983 0.011276 0.372286

F81 1990.06503 97 0.023558 0.015994 0.011270 0.383556

HKY 1990.04277 98 0.023558 0.015994 0.011270 0.394827

TIM3+I 1976.34961 101 0.023869 0.016305 0.011124 0.405950

TPM3uf+I 1976.43500 100 0.023885 0.016321 0.011116 0.417066

K80 2039.88158 95 0.024681 0.017117 0.010758 0.427824

JC 2039.99294 94 0.024681 0.017117 0.010757 0.438581

TrNef 2039.66627 96 0.024689 0.017125 0.010754 0.449335

TPM2 2039.68826 96 0.024736 0.017172 0.010734 0.460069

TIM2ef 2039.47245 97 0.024745 0.017181 0.010730 0.470798

TPM3 2039.66915 96 0.024768 0.017204 0.010720 0.481518

TIM3ef 2039.45433 97 0.024776 0.017213 0.010716 0.492234

TIM2+I+G 1976.72556 102 0.025464 0.017900 0.010427 0.502661

TPM2uf+I+G 1976.86696 101 0.025483 0.017919 0.010419 0.513079

TIM1+I+G 1954.03172 102 0.025489 0.017925 0.010417 0.523496

GTR+I+G 1953.53790 104 0.025505 0.017941 0.010410 0.533906

TPM1uf+I+G 1954.15119 101 0.025509 0.017945 0.010408 0.544314

TrN+I+G 1978.24275 101 0.025514 0.017950 0.010406 0.554721

TIM3+I+G 1974.82046 102 0.025518 0.017954 0.010405 0.565125

TVM+I+G 1953.62423 103 0.025528 0.017964 0.010401 0.575526

TPM3uf+I+G 1974.88523 101 0.025532 0.017968 0.010399 0.585925

HKY+I+G 1978.35406 100 0.025535 0.017971 0.010398 0.596323

F81+I+G 1978.36107 99 0.025535 0.017971 0.010398 0.606720

TrN+I 1979.71054 100 0.025650 0.018087 0.010351 0.617071

F81+I 1979.84385 98 0.025671 0.018107 0.010343 0.627414

TVMef 2021.68025 98 0.025848 0.018284 0.010272 0.637685

SYM 2021.46580 99 0.025856 0.018292 0.010268 0.647954

TIM2ef+I 2028.37515 98 0.025884 0.018320 0.010258 0.658212

TPM1 2022.27793 96 0.025946 0.018382 0.010233 0.668445

TIM1ef 2022.06300 97 0.025956 0.018392 0.010229 0.678674

SYM+G 2008.51934 100 0.025979 0.018415 0.010220 0.688894

TVMef+G 2008.57144 99 0.025982 0.018418 0.010219 0.699113

TrNef+G 2027.31565 97 0.026075 0.018511 0.010183 0.709295

JC+G 2027.48582 95 0.026078 0.018514 0.010181 0.719476

K80+G 2027.36975 96 0.026078 0.018514 0.010181 0.729658

TIM2ef+G 2027.31470 98 0.026079 0.018515 0.010181 0.739838

TPM2+G 2027.36867 97 0.026083 0.018519 0.010179 0.750018

TIM1ef+G 2009.57238 98 0.026112 0.018548 0.010168 0.760186

TPM1+G 2009.62507 97 0.026116 0.018552 0.010166 0.770352

TIM3ef+G 2027.27553 98 0.026122 0.018558 0.010164 0.780516

TPM3+G 2027.33247 97 0.026124 0.018560 0.010163 0.790680

TPM2+I 2028.43296 97 0.026153 0.018590 0.010152 0.800832

TVMef+I 2009.65512 99 0.026684 0.019120 0.009950 0.810782

HKY+I 1979.83768 99 0.026700 0.019136 0.009944 0.820726

TIM1ef+I 2010.67594 98 0.026743 0.019179 0.009928 0.830654

JC+I 2028.57639 95 0.026924 0.019360 0.009861 0.840515

K80+I 2028.46030 96 0.026925 0.019361 0.009861 0.850376

TIM3ef+I 2028.36586 98 0.026967 0.019403 0.009846 0.860222

TPM3+I 2028.42747 97 0.026997 0.019433 0.009835 0.870056

TPM1+I 2010.73276 97 0.027036 0.019472 0.009821 0.879877

TrNef+I 2028.40172 97 0.027105 0.019541 0.009796 0.889672

SYM+I 2009.59843 100 0.028350 0.020786 0.009365 0.899037

SYM+I+G 2008.08649 101 0.028820 0.021256 0.009212 0.908250

TVMef+I+G 2008.13387 100 0.028840 0.021276 0.009206 0.917456

TIM2ef+I+G 2026.92040 99 0.028925 0.021361 0.009179 0.926635

TPM2+I+G 2026.96837 98 0.028930 0.021366 0.009178 0.935813

TrNef+I+G 2026.92131 98 0.028932 0.021368 0.009177 0.944990

JC+I+G 2027.08664 96 0.028939 0.021375 0.009175 0.954164

K80+I+G 2026.97015 97 0.028939 0.021375 0.009175 0.963339

TIM1ef+I+G 2009.16693 99 0.028965 0.021401 0.009167 0.972506

TIM3ef+I+G 2026.88670 99 0.028969 0.021405 0.009165 0.981671

TPM1+I+G 2009.21342 98 0.028970 0.021406 0.009165 0.990836

TPM3+I+G 2026.93735 98 0.028972 0.021408 0.009164 1.000000

-------------------------------------------------------------------------

-lnL:t negative log likelihod

K: number of estimated parameters

DT: decision theory performance-based score

delta: DT difference

weight: DT weight* (calculated using 1/DT)

cumWeight: cumulative DT weight

* DT MODEL SELECTION : Confidence interval

There are 88 models in the 100% confidence interval: [ TPM1uf+G GTR+G TVM+G TIM2+G TPM2uf+G TIM1+G TrN+G TIM3+G HKY+G F81+G TPM3uf+G TIM1+I TIM2+I TPM1uf+I TPM2uf+I GTR+I TVM+I TIM2 TPM2uf GTR TIM1 TVM TPM1uf TIM3 TPM3uf TrN F81 HKY TIM3+I TPM3uf+I K80 JC TrNef TPM2 TIM2ef TPM3 TIM3ef TIM2+I+G TPM2uf+I+G TIM1+I+G GTR+I+G TPM1uf+I+G TrN+I+G TIM3+I+G TVM+I+G TPM3uf+I+G HKY+I+G F81+I+G TrN+I F81+I TVMef SYM TIM2ef+I TPM1 TIM1ef SYM+G TVMef+G TrNef+G JC+G K80+G TIM2ef+G TPM2+G TIM1ef+G TPM1+G TIM3ef+G TPM3+G TPM2+I TVMef+I HKY+I TIM1ef+I JC+I K80+I TIM3ef+I TPM3+I TPM1+I TrNef+I SYM+I SYM+I+G TVMef+I+G TIM2ef+I+G TPM2+I+G TrNef+I+G JC+I+G K80+I+G TIM1ef+I+G TIM3ef+I+G TPM1+I+G TPM3+I+G ]

* DT MODEL SELECTION : Parameter importance

Parameter Importance

----------------------

fA 0.5622

fC 0.5622

fG 0.5622

fT 0.5622

kappa 0.0837

titv 0.0837

rAC 0.5556

rAG 0.8322

rAT 0.5784

rCG 0.5766

rCT 0.8322

rGT 0.8322

pinv(I) 0.2303

alpha(G) 0.3138

pinv(IG) 0.2154

alpha(IG) 0.2154

----------------------

Values have been rounded.

(I): considers only +I models.

(G): considers only +G models.

(IG): considers only +I+G models.

* DT MODEL SELECTION : Best Model's command line

phyml -i /tmp/jmodeltest13390012638257739752.phy -d nt -n 1 -b 0 --run_id TPM1uf+G -m 012210 -f m -c 4 -a e --no_memory_check -o tlr -s BEST

---------------------------------------------------------------

* *

* MODEL AVERAGED PHYLOGENY *

* *

---------------------------------------------------------------

Selection criterion: . . . . DT

Confidence interval: . . . . 1.00

Consensus type:. . . . . . . 50% majority rule

Using 88 models in the 1.00 confidence interval = TPM1uf+G GTR+G TVM+G TIM2+G TPM2uf+G TIM1+G TrN+G TIM3+G HKY+G F81+G TPM3uf+G TIM1+I TIM2+I TPM1uf+I TPM2uf+I GTR+I TVM+I TIM2 TPM2uf GTR TIM1 TVM TPM1uf TIM3 TPM3uf TrN F81 HKY TIM3+I TPM3uf+I K80 JC TrNef TPM2 TIM2ef TPM3 TIM3ef TIM2+I+G TPM2uf+I+G TIM1+I+G GTR+I+G TPM1uf+I+G TrN+I+G TIM3+I+G TVM+I+G TPM3uf+I+G HKY+I+G F81+I+G TrN+I F81+I TVMef SYM TIM2ef+I TPM1 TIM1ef SYM+G TVMef+G TrNef+G JC+G K80+G TIM2ef+G TPM2+G TIM1ef+G TPM1+G TIM3ef+G TPM3+G TPM2+I TVMef+I HKY+I TIM1ef+I JC+I K80+I TIM3ef+I TPM3+I TPM1+I TrNef+I SYM+I SYM+I+G TVMef+I+G TIM2ef+I+G TPM2+I+G TrNef+I+G JC+I+G K80+I+G TIM1ef+I+G TIM3ef+I+G TPM1+I+G TPM3+I+G

Species in order:

1. Ae_lepidioides

2. HM95_spinosum_S592

3. Ae_eunomioides

4. Ae_karamanicum

5. Ae_diastrophis

6. Ae_umbellatum

7. Ae_coridifolium

8. Ae_armenum

9. Ae_alanyae

10. Ae_schistosum

11. Ae_demirizii

12. DQ180216_elongatum

13. Ae_glaucinum

14. HM100_grandiflorum_S574

15. Ae_huber_morathii

16. Ae_capitatum

17. HM326_erinaceum_S572

18. Ae_spicatum

19. HM86_transhyrcanum_S579

20. HM104_membranaceum_S573

21. Ae_lycium

22. Ae_turcica

23. Ae_thesiifolium

24. Ae_dumanii

25. Ae_stenopterum

26. Ae_arabicum

27. Ae_heterocarpum

28. Ae_froedinii

29. Ae_syriacum

30. Ae_carneum

31. Ae_cordatum

32. Umbellatum_Archibold_J1

33. HM1454_Oshrurankuh_S1857

34. W_0184833_Aethionema_trnT-trnL-trnF

35. HM1452_yazd_S1855

36. _Shirkuh_J2_trnL

37. Ae_orbiculatum

38. Ae_acarii

39. Ae_saxatile

40. Ae_fimbriatum

41. Ae_stylosum

42. Ae_speciosum

43. Ae_papillosum

44. Ae_munzurense

45. HM480_spec_nova_S647

46. HM479_spec._nova_S646

47. HM478_spec_nova_S645

48. HM482_spec_nova_S648

Bipartitions included in the consensus tree

123456789111111111122222222223333333333444444444

012345678901234567890123456789012345678

************************************************ ( 1.0 )

--********************************************** ( 1.0 )

--******************---------------------------- ( 1.0 )

---*****************---------------------------- ( 1.0 )

---**********----------------------------------- ( 1.0 )

----*********----------------------------------- ( 1.0 )

----****---------------------------------------- ( 1.0 )

-----***---------------------------------------- ( 1.0 )

------**---------------------------------------- ( 0.69361 )

--------*****----------------------------------- ( 0.5543 )

---------****----------------------------------- ( 0.5543 )

----------***----------------------------------- ( 1.0 )

-----------**----------------------------------- ( 0.93486 )

-------------*******---------------------------- ( 1.0 )

-------------*****------------------------------ ( 1.0 )

-------------***-------------------------------- ( 1.0 )

--------------**-------------------------------- ( 0.75682 )

----------------**------------------------------ ( 0.56821 )

------------------**---------------------------- ( 1.0 )

--------------------**************************** ( 1.0 )

-----------------------*******------------------ ( 1.0 )

------------------------******------------------ ( 1.0 )

-------------------------*****------------------ ( 1.0 )

--------------------------****------------------ ( 0.67523 )

---------------------------***------------------ ( 0.5543 )

---------------------------**------------------- ( 1.0 )

-------------------------------***************** ( 1.0 )

-------------------------------*****------------ ( 1.0 )

-------------------------------**--------------- ( 1.0 )

---------------------------------***------------ ( 1.0 )

----------------------------------**------------ ( 1.0 )

------------------------------------********---- ( 0.60156 )

-------------------------------------*******---- ( 1.0 )

-------------------------------------**--------- ( 1.0 )

---------------------------------------*****---- ( 1.0 )

----------------------------------------****---- ( 0.68464 )

-----------------------------------------***---- ( 1.0 )

------------------------------------------**---- ( 0.61153 )

--------------------------------------------**** ( 0.85523 )

+-48 Ae_glaucinum

+-78

| +-47 DQ180216_elongatum

+-----79

| +-46 Ae_demirizii

+-80

| +-45 Ae_schistosum

+-81

| +-44 Ae_alanyae

+-82

| | +-43 Ae_armenum

| | +-75

| | | +-42 Ae_coridifolium

| | +----76

| | | +-41 Ae_umbellatum

| +-77

| +-40 Ae_diastrophis

+--83

| +-39 Ae_karamanicum

+-84

| | +-38 HM104_membranaceum_S573

| | +-73

| | | +-37 HM86_transhyrcanum_S579

| +--74

| | +---36 Ae_spicatum

| | +-71

| | | +---35 HM326_erinaceum_S572

| +-----72

| | +-34 Ae_capitatum

| | +-69

| | | +-33 Ae_huber_morathii

| +-70

| +-32 HM100_grandiflorum_S574

+--85

| +-31 Ae_eunomioides

+----------86

| | +-30 Ae_munzurense

| | +-61

| | | +-29 Ae_papillosum

| | +-62

| | | +-28 Ae_speciosum

| | +-63

| | | +--27 Ae_stylosum

| | +-64

| | | +-----26 Ae_fimbriatum

| | +-65

| | | | +-25 Ae_saxatile

| | | +-60

| | | +-----24 Ae_acarii

| | +-66

| | | +-23 Ae_orbiculatum

| | |

| | | +-22 HM482_spec_nova_S648

| | | |

| | | +-21 HM478_spec_nova_S645

| | +-59

| | | +-20 HM479_spec._nova_S646

| | | |

| | | +-19 HM480_spec_nova_S647

| | +--67

| | | | +-18 _Shirkuh_J2_trnL

| | | | +-56

| | | | | +-17 HM1452_yazd_S1855

| | | | +-57

| | | | | +-16 W_0184833_Aethionema_trnT-trnL-trnF

| | | +-58

| | | | +-15 HM1454_Oshrurankuh_S1857

| | | +---55

| | | +-14 Umbellatum_Archibold_J1

| | |

| | | +-13 Ae_syriacum

| | | +-49

| | | | +-12 Ae_froedinii

| | | +-50

| | | | +-11 Ae_carneum

| | | +-51

| | | | +-10 Ae_heterocarpum

| | | +-52

| | | | +--9 Ae_arabicum

| | | +-53

| | | | +--8 Ae_stenopterum

| | +-54

| | | +----7 Ae_dumanii

| | |

| | +----6 Ae_cordatum

| +--------68

| +--5 Ae_thesiifolium

| |

| +---4 Ae_turcica

| |

| +--3 Ae_lycium

|

+---2 HM95_spinosum_S592

|

+--------------------------1 Ae_lepidioides

(Ae_lepidioides:0.0311758900,HM95_spinosum_S592:0.0051485500,((Ae_lycium:0.0015676400,Ae_turcica:0.0047403000,Ae_thesiifolium:0.0015692100,Ae_cordatum:0.0063221000,(Ae_dumanii:0.0064700600,(Ae_stenopterum:0.0031509200,(Ae_arabicum:0.0000000600,(Ae_heterocarpum:0.0031527400,(Ae_carneum:0.0000000600,(Ae_froedinii:0.0000000100,Ae_syriacum:0.0000000100):1.0000:0.0015720400):0.5543:0.0000000700):0.6752:0.0000000600):1.0000:0.0031413600):1.0000:0.0031383200):1.0000:0.0015595800,(((Umbellatum_Archibold_J1:0.0000000100,HM1454_Oshrurankuh_S1857:0.0015804700):1.0000:0.0063894100,(W_0184833_Aethionema_trnT-trnL-trnF:0.0000000100,(HM1452_yazd_S1855:0.0000000100,_Shirkuh_J2_trnL:0.0000001100):1.0000:0.0000000100):1.0000:0.0023252300):1.0000:0.0023007900,(HM480_spec_nova_S647:0.0000000800,HM479_spec._nova_S646:0.0000000800,HM478_spec_nova_S645:0.0015626200,HM482_spec_nova_S648:0.0000000500):0.8552:0.0015776800,(Ae_orbiculatum:0.0000000800,((Ae_acarii:0.0088482400,Ae_saxatile:0.0000000100):1.0000:0.0036350000,(Ae_fimbriatum:0.0081567300,(Ae_stylosum:0.0048102100,(Ae_speciosum:0.0031578400,(Ae_papillosum:0.0015796700,Ae_munzurense:0.0031617500):0.6115:0.0000000900):1.0000:0.0031622300):0.6846:0.0000000800):1.0000:0.0015768500):1.0000:0.0015754600):0.6016:0.0015716100):1.0000:0.0047086800):1.0000:0.0117459300,(Ae_eunomioides:0.0031705000,((((HM100_grandiflorum_S574:0.0031398600,(Ae_huber_morathii:0.0015671000,Ae_capitatum:0.0019781400):0.7568:0.0000000800):1.0000:0.0016322800,(HM326_erinaceum_S572:0.0066853900,Ae_spicatum:0.0062897700):0.5682:0.0000001500):1.0000:0.0082680500,(HM86_transhyrcanum_S579:0.0000000700,HM104_membranaceum_S573:0.0015530000):1.0000:0.0033151600):1.0000:0.0052131000,(Ae_karamanicum:0.0000000800,((Ae_diastrophis:0.0000000800,(Ae_umbellatum:0.0031191300,(Ae_coridifolium:0.0000000100,Ae_armenum:0.0015533400):0.6936:0.0000000100):1.0000:0.0078557300):1.0000:0.0015585700,(Ae_alanyae:0.0000000800,(Ae_schistosum:0.0031349100,(Ae_demirizii:0.0000000100,(DQ180216_elongatum:0.0000000100,Ae_glaucinum:0.0000000700):0.9349:0.0000003800):1.0000:0.0083285900):0.5543:0.0000005200):0.5543:0.0000000800):1.0000:0.0016449200):1.0000:0.0050088600):1.0000:0.0016106500):1.0000:0.0048843000):1.0000:0.0139465600);

Note: this tree is unrooted. Branch lengths are the expected number of substitutions per site. Labels next to parentheses represent phylogenetic uncertainty due to model selection (see documentation)

---------------------------------------------------------------

* *

* SELECTION SUMMARY *

* *

---------------------------------------------------------------

::Optimized Topologies Summary::

There are 26 different topologies.

Topology Id: 1

Rank Weight RF AvgEucl VarEucl

AIC 1 43.15% 0 6.48e-05 2.71e-09

BIC 1 72.70% 0 6.48e-05 2.71e-09

AICc 1 49.57% 0 6.48e-05 2.71e-09

DT 1 10.46% 0 6.48e-05 2.71e-09

Models supporting: 3

TPM1uf+G TVM+G GTR+G

Topology Id: 2

Rank Weight RF AvgEucl VarEucl

AIC 2 0.00% 6 2.52e-02 6.97e-09

BIC 2 0.00% 6 2.52e-02 6.97e-09

AICc 2 0.00% 6 2.52e-02 6.97e-09

DT 2 15.30% 6 2.52e-02 6.97e-09

Models supporting: 15

JC+G K80+G TrNef+G TPM1 TPM1+G TPM2+G TPM3+G TIM1ef TIM1ef+G TIM2ef+G TIM3ef+G TVMef

TVMef+G SYM SYM+G

Topology Id: 3

Rank Weight RF AvgEucl VarEucl

AIC 3 0.00% 10 2.39e-02 1.25e-09

BIC 3 0.00% 10 2.39e-02 1.25e-09

AICc 3 0.00% 10 2.39e-02 1.25e-09

DT 3 7.52% 10 2.39e-02 1.25e-09

Models supporting: 7

JC K80 TrNef TPM2 TPM3 TIM2ef TIM3ef

Topology Id: 4

Rank Weight RF AvgEucl VarEucl

AIC 4 13.64% 12 2.24e-02 5.37e-10

BIC 4 3.11% 12 2.24e-02 5.37e-10

AICc 4 12.58% 12 2.24e-02 5.37e-10

DT 4 9.72% 12 2.24e-02 5.37e-10

Models supporting: 8

F81+G HKY+G TrN+G TPM2uf+G TPM3uf+G TIM1+G TIM2+G TIM3+G

Topology Id: 5

Rank Weight RF AvgEucl VarEucl

AIC 5 0.00% 14 2.41e-02 4.01e-10

BIC 5 0.04% 14 2.41e-02 4.01e-10

AICc 5 0.01% 14 2.41e-02 4.01e-10

DT 5 10.16% 14 2.41e-02 4.01e-10

Models supporting: 9

F81 HKY TrN TPM1uf TPM3uf TIM1 TIM3 TVM GTR

Topology Id: 6

Rank Weight RF AvgEucl VarEucl

AIC 6 0.00% 16 2.39e-02 4.20e-11

BIC 6 0.00% 16 2.39e-02 4.20e-11

AICc 6 0.00% 16 2.39e-02 4.20e-11

DT 6 2.28% 16 2.39e-02 4.20e-11

Models supporting: 2

TPM2uf TIM2

Topology Id: 7

Rank Weight RF AvgEucl VarEucl

AIC 7 0.00% 22 2.68e-02 0.00e+00

BIC 7 0.00% 22 2.68e-02 0.00e+00

AICc 7 0.00% 22 2.68e-02 0.00e+00

DT 7 0.99% 22 2.68e-02 0.00e+00

Models supporting: 1

TIM1ef+I

Topology Id: 8

Rank Weight RF AvgEucl VarEucl

AIC 8 0.00% 24 2.62e-02 0.00e+00

BIC 8 0.00% 24 2.62e-02 0.00e+00

AICc 8 0.00% 24 2.62e-02 0.00e+00

DT 8 1.14% 24 2.62e-02 0.00e+00

Models supporting: 1

TPM2uf+I

Topology Id: 9

Rank Weight RF AvgEucl VarEucl

AIC 9 0.00% 26 2.70e-02 0.00e+00

BIC 9 0.00% 26 2.70e-02 0.00e+00

AICc 9 0.00% 26 2.70e-02 0.00e+00

DT 9 0.98% 26 2.70e-02 0.00e+00

Models supporting: 1

TPM1+I

Topology Id: 10

Rank Weight RF AvgEucl VarEucl

AIC 10 0.00% 26 2.28e-02 6.46e-11

BIC 10 0.00% 26 2.28e-02 6.46e-11

AICc 10 0.00% 26 2.28e-02 6.46e-11

DT 10 2.22% 26 2.28e-02 6.46e-11

Models supporting: 2

TPM3uf+I TIM3+I

Topology Id: 11

Rank Weight RF AvgEucl VarEucl

AIC 11 3.68% 26 2.57e-02 0.00e+00

BIC 11 0.84% 26 2.57e-02 0.00e+00

AICc 11 3.39% 26 2.57e-02 0.00e+00

DT 11 1.15% 26 2.57e-02 0.00e+00

Models supporting: 1

TIM1+I

Topology Id: 12

Rank Weight RF AvgEucl VarEucl

AIC 12 0.80% 26 2.62e-02 3.48e-10

BIC 12 0.00% 26 2.62e-02 3.48e-10

AICc 12 0.35% 26 2.62e-02 3.48e-10

DT 12 2.29% 26 2.62e-02 3.48e-10

Models supporting: 2

TIM2+I GTR+I

Topology Id: 13

Rank Weight RF AvgEucl VarEucl

AIC 13 0.00% 26 2.67e-02 0.00e+00

BIC 13 0.00% 26 2.67e-02 0.00e+00

AICc 13 0.00% 26 2.67e-02 0.00e+00

DT 13 1.00% 26 2.67e-02 0.00e+00

Models supporting: 1

TVMef+I

Topology Id: 14

Rank Weight RF AvgEucl VarEucl

AIC 14 1.97% 26 2.91e-02 0.00e+00

BIC 14 0.05% 26 2.91e-02 0.00e+00

AICc 14 1.26% 26 2.91e-02 0.00e+00

DT 14 1.14% 26 2.91e-02 0.00e+00

Models supporting: 1

TVM+I

Topology Id: 15

Rank Weight RF AvgEucl VarEucl

AIC 15 0.00% 28 2.62e-02 1.15e-10

BIC 15 0.00% 28 2.62e-02 1.15e-10

AICc 15 0.00% 28 2.62e-02 1.15e-10

DT 15 2.07% 28 2.62e-02 1.15e-10

Models supporting: 2

F81+I TrN+I

Topology Id: 16

Rank Weight RF AvgEucl VarEucl

AIC 16 0.00% 28 2.76e-02 0.00e+00

BIC 16 0.00% 28 2.76e-02 0.00e+00

AICc 16 0.00% 28 2.76e-02 0.00e+00

DT 16 0.99% 28 2.76e-02 0.00e+00

Models supporting: 1

HKY+I

Topology Id: 17

Rank Weight RF AvgEucl VarEucl

AIC 17 0.00% 28 2.76e-02 0.00e+00

BIC 17 0.00% 28 2.76e-02 0.00e+00

AICc 17 0.00% 28 2.76e-02 0.00e+00

DT 17 0.98% 28 2.76e-02 0.00e+00

Models supporting: 1

TrNef+I

Topology Id: 18

Rank Weight RF AvgEucl VarEucl

AIC 18 8.75% 28 2.91e-02 0.00e+00

BIC 18 19.41% 28 2.91e-02 0.00e+00

AICc 18 11.65% 28 2.91e-02 0.00e+00

DT 18 1.15% 28 2.91e-02 0.00e+00

Models supporting: 1

TPM1uf+I

Topology Id: 19

Rank Weight RF AvgEucl VarEucl

AIC 19 0.00% 28 2.67e-02 0.00e+00

BIC 19 0.00% 28 2.67e-02 0.00e+00

AICc 19 0.00% 28 2.67e-02 0.00e+00

DT 19 1.02% 28 2.67e-02 0.00e+00

Models supporting: 1

TPM2+I

Topology Id: 20

Rank Weight RF AvgEucl VarEucl

AIC 20 0.00% 28 2.95e-02 0.00e+00

BIC 20 0.00% 28 2.95e-02 0.00e+00

AICc 20 0.00% 28 2.95e-02 0.00e+00

DT 20 0.94% 28 2.95e-02 0.00e+00

Models supporting: 1

SYM+I

Topology Id: 21

Rank Weight RF AvgEucl VarEucl

AIC 21 0.00% 30 2.72e-02 5.16e-14

BIC 21 0.00% 30 2.72e-02 5.16e-14

AICc 21 0.00% 30 2.72e-02 5.16e-14

DT 21 1.97% 30 2.72e-02 5.16e-14

Models supporting: 2

JC+I K80+I

Topology Id: 22

Rank Weight RF AvgEucl VarEucl

AIC 22 0.00% 30 2.88e-02 2.32e-09

BIC 22 0.00% 30 2.88e-02 2.32e-09

AICc 22 0.00% 30 2.88e-02 2.32e-09

DT 22 10.10% 30 2.88e-02 2.32e-09

Models supporting: 11

JC+I+G K80+I+G TrNef+I+G TPM1+I+G TPM2+I+G TPM3+I+G TIM1ef+I+G TIM2ef+I+G TIM3ef+I+G

TVMef+I+G SYM+I+G

Topology Id: 23

Rank Weight RF AvgEucl VarEucl

AIC 23 28.00% 30 2.55e-02 5.29e-10

BIC 23 3.84% 30 2.55e-02 5.29e-10

AICc 23 21.19% 30 2.55e-02 5.29e-10

DT 23 11.45% 30 2.55e-02 5.29e-10

Models supporting: 11

F81+I+G HKY+I+G TrN+I+G TPM1uf+I+G TPM2uf+I+G TPM3uf+I+G TIM1+I+G TIM2+I+G TIM3+I+G

TVM+I+G GTR+I+G

Topology Id: 24

Rank Weight RF AvgEucl VarEucl

AIC 24 0.00% 30 2.69e-02 0.00e+00

BIC 24 0.00% 30 2.69e-02 0.00e+00

AICc 24 0.00% 30 2.69e-02 0.00e+00

DT 24 0.98% 30 2.69e-02 0.00e+00

Models supporting: 1

TPM3+I

Topology Id: 25

Rank Weight RF AvgEucl VarEucl

AIC 25 0.00% 30 2.75e-02 0.00e+00

BIC 25 0.00% 30 2.75e-02 0.00e+00

AICc 25 0.00% 30 2.75e-02 0.00e+00

DT 25 1.03% 30 2.75e-02 0.00e+00

Models supporting: 1

TIM2ef+I

Topology Id: 26

Rank Weight RF AvgEucl VarEucl

AIC 26 0.00% 32 2.73e-02 0.00e+00

BIC 26 0.00% 32 2.73e-02 0.00e+00

AICc 26 0.00% 32 2.73e-02 0.00e+00

DT 26 0.98% 32 2.73e-02 0.00e+00

Models supporting: 1

TIM3ef+I

::Best Models::

Model f(a) f(c) f(g) f(t) kappa titv Ra Rb Rc Rd Re Rf pInv gamma

----------------------------------------------------------------------------------------------------------------------------------------

AIC TPM1uf+G 0.31 0.16 0.16 0.36 0.00 0.00 1.000 0.588 0.211 0.211 0.588 1.000 N/A 0.52

BIC TPM1uf+G 0.31 0.16 0.16 0.36 0.00 0.00 1.000 0.588 0.211 0.211 0.588 1.000 N/A 0.52

AICc TPM1uf+G 0.31 0.16 0.16 0.36 0.00 0.00 1.000 0.588 0.211 0.211 0.588 1.000 N/A 0.52

DT TPM1uf+G 0.31 0.16 0.16 0.36 0.00 0.00 1.000 0.588 0.211 0.211 0.588 1.000 N/A 0.52

Program is done.
